# Revealing two dynamic dengue epidemic clusters in Thailand

**DOI:** 10.1186/s12879-020-05666-4

**Published:** 2020-12-04

**Authors:** Jue Tao Lim, Yiting Han, Borame Sue Lee Dickens, Esther Li Wen Choo, Lawrence Zheng Xiong Chew, Alex R. Cook

**Affiliations:** 1 Saw Swee Hock School of Public Health, National University Health Systems, National University of Singapore, Singapore, Singapore; 2 School of Pharmacy, Fudan University, Shanghai, China; 3 Department of Biological Sciences, Faculty of Science, National University of Singapore, Singapore, Singapore; 4 Department of Geography, Faculty of Arts and Social Sciences, National University of Singapore, Singapore, Singapore

**Keywords:** Dengue, Outbreaks, Clusters, Thailand

## Abstract

**Background:**

Thailand is home to around 69 million individuals. Dengue is hyper-endemic and all 4 serotypes are in active circulation in the country. Dengue outbreaks occur almost annually within Thailand in at least one province but the spatio-temporal and environmental interface of these outbreaks has not been studied.

**Methods:**

We develop Bayesian regime switching (BRS) models to characterize outbreaks, their persistence and infer their likelihood of occurrence across time for each administrative province where dengue case counts are collected. BRS was compared against two other classification tools and their agreement is assessed. We further examine how these spatio-temporal clusters of outbreak clusters arise by comparing reported dengue case counts, urban population, urban land cover, climate and flight volumes on the province level.

**Results:**

Two dynamic dengue epidemic clusters were found nationally. One cluster consists of 47 provinces and is highly outbreak prone. Provinces with a large number of case counts, urban population, urban land cover and incoming flight passengers are associated to the epidemic prone cluster of dengue. Climate has an effect on determining the probability of outbreaks over time within provinces, but have less influence on whether provinces belong to the epidemic prone cluster. BRS found high agreement with other classification tools.

**Conclusions:**

Importation and urbanization drives the risk of outbreaks across regions strongly. In provinces estimated to have high epidemic persistence, more resource allocation to vector control should be applied to those localities as heightened transmission counts are likely to occur over a longer period of time. Clustering of epidemic and non-epidemic prone areas also highlights the need for prioritization of resource allocation for disease mitigation over provinces in Thailand.

**Supplementary Information:**

**Supplementary information** accompanies this paper at 10.1186/s12879-020-05666-4.

## Background

Thailand is home to around 69 million individuals [1], with dengue considered to be hyper-endemic due to all four serotypes being in active circulation within the country. Each of the 77 provinces in Thailand have on average, non-zero reported dengue case counts over the past 10 years, which create considerable health and economic burdens. Widespread urbanization, favourable climatic conditions and increased human mobility across provinces are ideal for dengue transmission [2]. Vector control has been and continues to be the primary control method for the two dominant dengue mosquito vectors, *Aedes aegypti* and *Aedes albopictus* as other control methods such as Dengvaxia (CYD-TDV) vaccination face issues of low seropostive rates, especially among the younger age groups [3]. The resources allocated for vector control are unfortunately limited even for the case of high income countries. A deep understanding of the epidemiological factors leading to dengue epidemics is therefore necessary, for appropriate resource planning and effective public health policy making. The identification of high risk, epidemic prone areas with frequent outbreaks would help allocate resources to these zones to reduce the public health burdens of dengue – which is especially important for countries which are vast, such as Thailand.

Considerable work has been conducted to understand the temporal behaviour of dengue in Thailand, ranging from peri-urban settings [4, 5], provincial level studies analyzing province specific trends [6–8] as well as national level transmission patterns [3, 9–13]. At the national level, studies often split reported counts at the province level in order to reconstruct suitable inference on how dengue is transmitted and the ways surveillance can be implemented on different frequencies [9]. A substantial number of studies also focus on understanding predicting dengue incidence in Thailand [14–16].

A key gap however, is understanding the patterns of disease outbreaks, instead of the number of reported cases. This is less frequently attempted due to the inherent difficulty in classifying what an outbreak is, or when an outbreak starts or ends. This difficulty in ascertaining outbreak patterns and understanding the drivers of outbreaks, is further compounded for a hyper-endemic disease such as dengue in Thailand, where case counts are consistently reported over time. Yet, as is estimated that early detection and in-time clinical care will drastically reduce the dengue fatality rates [17], the identification of outbreaks, their patterns and where they occur is essential to public health decision making for medical resource allocation and staffing. This will help optimize medical care delivery in an outbreak scenario and provide substantial gains in population health.

In this study, we therefore aim to understand the patterns and biological reasons for dengue outbreaks in Thailand, by first developing suitable methods to characterize outbreaks and infer their likelihood of occurrence across time for each administrative province where dengue case counts are collected. We further examined whether spatio-temporal clusters of outbreak clusters arise, and whether human population and biologically relevant factors such as reporting rate, urban land cover, population and climatic forcing affect these outbreaks over a large spatial scale.

## Methods

### Dengue case count data

Monthly dengue case count data in Thailand is collected by the Ministry of Public Health, Thailand with mandatory notification of virologically confirmed or laboratory-confirmed cases. To account for difference in population sizes among different regions and further allow interpretability of the regression coefficients, we normalized each province’s dengue case count time series to a scale of 0 to 1 for the analysis by subtracting each province’s timepoint by the province’s minimum observed value and divide them by the range of values observed across time. Data is available for 76 regions in Thailand from 2007 to 2018. We removed Bueng Kan from analysis as it was split from Nong Khai province in 2011. Dengue case count data was used to infer dengue outbreak patterns across provinces from 2007 to 2018.

### Climate data

Climate data was obtained from ERA5, published by the European Centre for Medium-Range Weather Forecasts. ERA5 provides hourly estimates across a 30 km grid [18], which we have aggregated over a monthly timescale and spatially averaged for provinces of Thailand. Mean, minimum and maximum air temperature at 2 m was calculated to represent thermal forcing and stress on vector population growth, and total rainfall for the weekly interval obtained for breeding site availability [19]. Air temperature and dewpoint temperature were utilized to calculate saturation vapor pressure and actual vapor pressure using Teten’s formula, where relative and absolute humidity could then be estimated using standard formula [20]. Absolute and relative humidity are used to represent the impacts of ambient moisture on breeding potential [21]. Climate data was used to determine whether climate is a driver of dengue outbreaks.

### Human population data

Human population data for Thailand was obtained from Thailand’s Official Statistics Registration Systems website, published by the Office of Registration Administration, Department of Local Administration. The population data provided is available for 77 regions in Thailand from 2007 to 2018, with the inclusion of Bueng Kan only after 2011. The yearly total population and births for each province were obtained for the required time period to determine the reporting rate of dengue via time series susceptible-infected recovered analysis [22]. Urban population and land cover data was used to determine whether outbreak prone clusters have higher rates of urbanization.

### Flight data

Monthly number of air ticket bookings during 2015–6 was obtained from OAG for every origin-destination route with up to two connections. The number of incoming flight passenger numbers for each airport within each province was then used to determine whether outbreak prone clusters have higher numbers of incoming flight passengers.

### Identifying outbreaks

Bayesian regime switching models (BRS) (1–2) are used to analyse recorded disease case counts over time which have characteristic changes in transmission behavior. It has been successfully applied for outbreak inference for seasonal and endemic diseases [23, 24]. This method provides a classification of the time series as separable regimes, as well as the probabilities of each regime to stay or transition to another regime over time. BRS is used to determine when dengue outbreaks occur for each province.

1 $$ {Y}_t={\beta}_{s_t,0}+{\sum}_{i=1}^p{\beta}_{s_t,i}{Y}_{t-i}+{\epsilon}_{s_t} $$

BRS (1) first models the observed dengue case counts *Y*_*t*_ as an evolution of past *i* month dengue case counts *Y*_*t* − *i*_ and an autoregressive factor ${\beta}_{S_t,i}$. With ${\epsilon}_{S_t}\sim N\left(0,{\sigma}_{S_t}^2\right)$ representing white noise. The intercept term ${\beta}_{S_t,0}$, denotes the mean level of dengue transmission over time and may vary within each regime *S*_*t*_, as well as the autoregressive and variance terms ${\beta}_{S_t,i}$ for a maximum of *p* lags and ${\sigma}_{S_t}^2$ respectively. Separate models are thus fitted depending on the regime classification at the current time point. The labelling of *S*_*t*_ as an epidemic or endemic regime is done post-hoc after estimation of *S*_*t*_ over all time points, based on each province’s dengue case count behaviour at the corresponding time points.

2 $$ P\left({S}_t|{S}_{t-1}\right)=\left[\begin{array}{ll}P\left({S}_t=0|{S}_{t-1}=0\right)=P& P\left({S}_t=0|{S}_{t-1}=1\right)=1-P\\ {}P\left({S}_t=1|{S}_{t-1}=0\right)=1-Q& P\left({S}_t=1|{S}_{t-1}=1\right)=Q\end{array}\right] $$

In the case of dengue, the transition probability matrix (2) describes the persistence and evolution of separable endemic (*S*_*t*_ = 0) and epidemic regimes (*S*_*t*_ = 1) – these correspond to non-outbreak and outbreak periods of dengue respectively. The likelihood of transitioning from a non-outbreak period to an outbreak period is given by 1 − *P* and the likelihood of staying within an outbreak period given that the past month is classified as an outbreak period is *Q*. All parameters, including the regimes, are estimated using a Gibbs sampling framework, with full computational details provided in the Additional file 1.

The ability for BRS to classify outbreaks is assessed in a rolling manner, where we fit the BRS specification (1) sequentially from around 1/3 of the data set at the 40th month onwards and increase the information set provided to the BRS by 1 more month in each refitting. The contemporaneously classified regime from the regime fitted to the final timepoint in each model iteration is compared against the classification where BRS is estimated on the full dataset. Two other commonly used outbreak classification tools were also used to assess classification stability of the BRS model. Namely, the cumulative sum framework of [25] and simple regression framework of [26] were used to further validate the results of BRS classification. Agreement between classification tools is measured using the area under the receiver operating curve, across 76 provinces of Thailand.

### Identifying dynamic clusters

We then assessed the tendency for outbreaks to be clustered across space and time, by using the graphical dissimilarity approach [27]. After which, we classified provinces into dynamic clusters using the partitioning around medoids (PAM) algorithm. This analysis allows us determine whether province-level dynamic clusters of epidemic prone areas exist over time. Dynamic time warped distances are used to determine the clusters each province are in and to account for possible temporal dependence between outbreak probabilities between provinces. We preset the number of clusters to 2 to 20 sequentially and ran the PAM algorithm, computing cluster validity indices to determine the optimal number of clusters for the classified outbreaks. The full assessment details for the optimal number of dynamic clusters is described in the Additional file 1.

### Inferring climatic forcing on outbreak probability

Finally, we looked at whether climate affects the probability for outbreaks to occur. However, as climatic variables are numerous, highly collinear and may affect outbreaks only after long time lags, inefficient parameter estimation may result using standard least squares approach [28]. We thus conduct inference using the least absolute shrinkage and selection operator (LASSO) for each province *p*, due to its ability to provide both model parsimony and regularization to enhance predictive accuracy and interpretability. In brief, the LASSO (3) was fitted with independent, province-level climatic variables *X*_*t* − *i*, *j*, *p*_ on *p*(*S*_*t*, *p*_ = 1), the estimated epidemic probability obtained from (1). Where *j* denotes climate variables for *i* number of lags across *p* provinces. Factors considered were previous air temperature, precipitation, absolute and relative humidity of up to 4 months so that possibly long-term climatic fluctuations could be taken into account. These factors were normalized 0 to 1 by subtracting each factor by its maximum value and dividing each differenced factor by the range of values each factor observes. Normalization was conducted to account for the different units of measurement and the non-invariance of LASSO regularization to scale [28].

3 $$ p\left({S}_{t,p}=1\right)={\beta}_{0,p}+\sum \limits_{j\in Climate}\sum \limits_{i\in Lags}{\beta}_{i,j,p}{X}_{t-i,j,p}, $$

$$ \mathrm{subject}\ \mathrm{to}\parallel {\beta}_p\parallel \le \lambda, \mathrm{for}\ \mathrm{some}\ \mathrm{penalty}\ \mathrm{term}\ \lambda $$

Five-fold cross validation was first conducted to yield test error rates which do not suffer from unreasonably high bias or variance [28]. The cross-validation step optimizes the regularization parameter *λ* using the area under curve of the receiving operator characteristic as the tuning criterion. We then refitted our data using the optimal regularization parameter *λ*^∗^ to produce probabilities for being in each regime at each timepoint. Next, bootstrapping was conducted over 200 iterations to recover bootstrap confidence intervals and bootstrap mean estimates [29] for each of our LASSO dependent variables. The bootstrap also allows computation of LASSO inclusion probabilities, which provides a measure of each climate variable’s importance in influencing outbreak probabilities. Analyses were done in R version 3.6.2.

## Results

### 2 dynamic dengue epidemic clusters revealed

Two dynamic dengue epidemic clusters were found, after conducting cluster analysis and assessment of the optimal number of clusters. The full results of these assessment checks are reported in the Additional file 1.

**Fig. 1:**
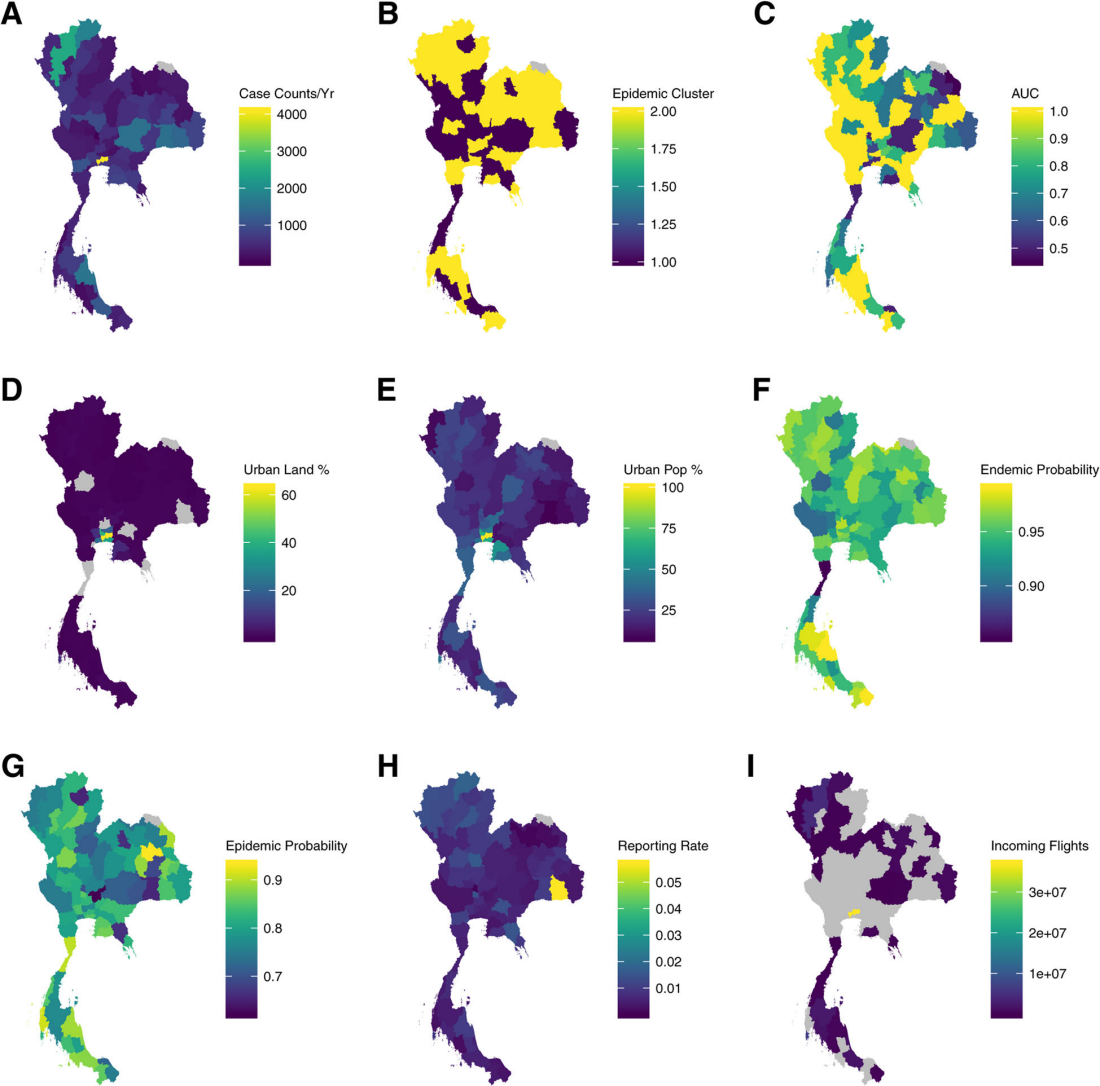
**a** Dengue case counts from 2008 to 2017. **b** Baseline (C1) and epidemic cluster (C2) classifications from cluster analysis. **c** AUC-ROC from post-hoc classification exercise for the BRS model. **d** Urban land cover percentage. **e** Municipal population percentage. **f** Endemic regime persistence probabilities estimated from the BRS model. **g** Epidemic regime persistence probabilities estimated from the BRS model. **h** Reporting rate of dengue as estimated from the canonical time series subsceptible-infected-recovered model. **i** Number of incoming flight passengers in 2017

**Fig. 2:**
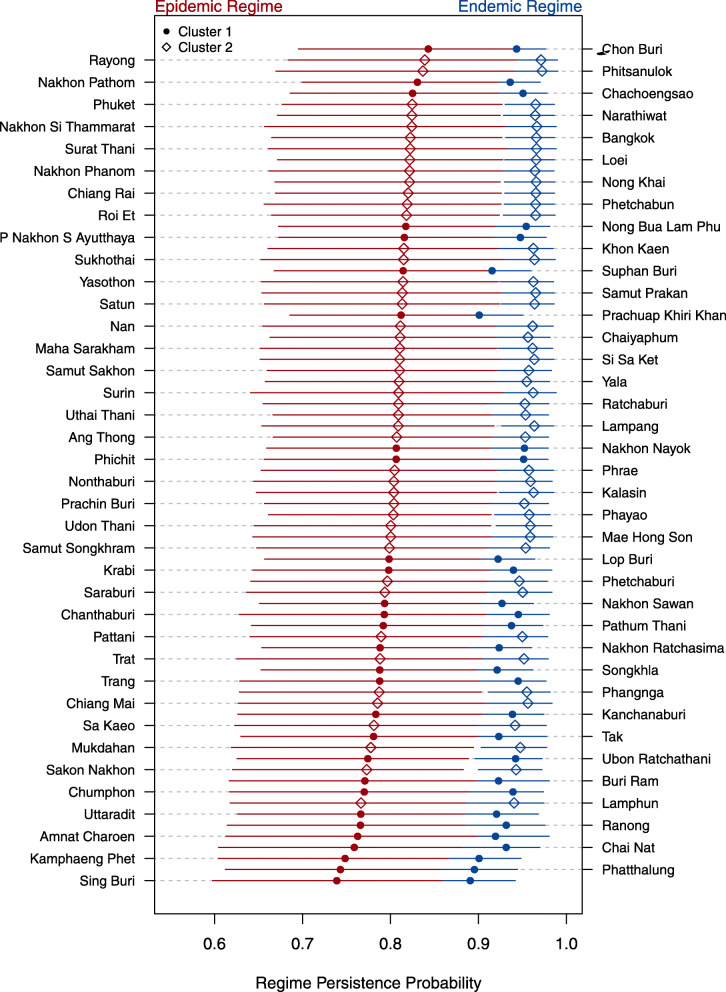
Probabilities of transitioning to an endemic regime if the past month estimated regime was endemic and the corresponding probabilities of transitioning to an epidemic regime if the past month estimated regime was epidemic. Lines represent 95% credible intervals, points represent the posterior mean estimate of persistence probabilities across all provinces of Thailand. Red and blue lines which overlap denote crossing 95% credible intervals. Red and blue points which are closer to each other denote more similar epidemic and endemic persistence probabilties

Cluster analysis indicates that cluster 1 (C1) consists of 29 provinces compared to cluster 2 (C2), which has 47. C1 is less prone to outbreaks compared to C2, with a concentration of outbreaks for C1 around 2013 to 2014, while C2 has outbreaks occurring almost yearly, asides from 2017 (Additional file 1). Spatially, the outbreak prone C2 is concentrated around the Northern region of Thailand, while the less outbreak prone C1 dispersed around the central and southern regions of Thailand (Fig. 1). Substantial variation in the persistence of the epidemics is estimated at the province-level, varying from 82% for Chon Buri province (C1) to 73% for Sing Buri province (C1) for staying in an epidemic. Likewise, for the baseline endemic regime, Phitsanulok (C2) province has the highest level of persistence at 99% and Prachuap Khiri Khan (C1) has the lowest level of persistence at 87%. In general, the likelihood of transitions from an epidemic regime into the endemic regime are in general higher compared to the likelihood of transitions from an endemic regime to an epidemic regime. The likelihood of staying within an epidemic regime is also lower, as compared to the likelihood of staying within an endemic regime. Jointly, this indicates that the epidemic regimes are less persistent compared to the endemic regimes across provinces (Fig. 2). C2 which is more outbreak prone, is also observed to have a larger group of provinces having a higher likelihood of staying within an epidemic regime, compared to the less outbreak prone C1 group (Fig. 2, Additional file 1).

Geographically, Chiang Rai, Chiang Mai, Mae Hong Son, Lamphun and Nan form a high confidence continuous spatial north western aggregation for C2 (Fig. 1, AUC-ROC: 0.76–0.94). This clustering continues across northern Thailand to the East at Nakhon Phnom (Fig. 1, AUC-ROC: 0.74–0.98) with less confidence observed at Khon Kaen (Fig. 1, AUC-ROC = 0.67). At the very East, Amnat Charoen and Ubon Ratchathani forms another discrete cluster (Fig. 1, C1, ROC: 0.88–0.94). Overall, Uttaradist and Non Bua Lamphu are distinct outliers within the dominant C2 zone in Northern Thailand (Fig. 1, C1 AUC-ROC 0.76, 0.90). A C1 belt exists across the central region, interspersed with C2 anomalies, notably Uthaithani, Ang Thong and Saraburi (Fig. 1, ROC: 0.86, 0.94 and 0.9). A mid C2 exists with Samut Prakan, Bangkok, Nonthaburi, Samut Sakhon and Samut Songkhram (Fig. 1, AUC-ROC: 0.87–0.93) where Phetchaburi is part of this cluster with weaker confidence (Fig. 1, AUC-ROC: 0.66). Prachuap Khiri Khan and Chumphon are a distinct C1 located in the mid corridor (Fig. 1, AUC-ROC: 0.76–0.98). In the south, Phang Nga, Surat Thani and Nakhon Si Thammarat form a C2 group with low confidence (Fig. 1, AUC-ROC = 0.44), with Pattani, Yala and Narathiwat at the very southern tip (Fig. 1, 0.63–0.65). In between these two C1 groupings, Krabi, Trang, Songkhla and Phattalung exist as a group (Fig. 1, AUC-ROC: 0.51–0.88).

### Dynamic clusters associated to endogenous and exogenous factors

The epidemic prone C2 cluster consists of regions which have the highest number of reported dengue case counts over 2007 to 2018, including that of Bangkok (Fig. 1a, Case Counts/Yr: 4080), Chiang Mai (Fig. 1a, Case Counts/Yr: 2478), Chiang Rai (Fig. 1a, Case Counts/Yr: 1809) and Si Sa Ket (Fig. 1a, Case Counts/Yr: 1335). These regions also have estimated reporting rates which are highly spread out compared to the mean levels experienced throughout the whole of Thailand. Bangkok province for example, has a dengue reporting rate of 0.5%, which does not deviate from the mean reporting rate of 0.7%, as compared to Si Sa Ket (C2), at the highest reporting rate of 5.7% (Fig. 1h). These 3 regions, also have the largest number of incoming flight passengers yearly as compared to other provinces in Thailand, at over 36 million for Bangkok, 4 million for Chiang Mai and 1 million for Chiang Rai (Fig. 1i).

Notably, the epidemic prone C2 consists of Bangkok and its adjacent/nearby provinces, Samut Prakan, Samut Songkhram, Nonthaburi and Ayutthaya which has the highest percentage of urban land cover in Thailand, at 63, 36, 41 and 19% respectively (Fig. 1d). It also corresponds to the highest percentage of each province’s population residing in urban regions at 100, 63, 23.4, 66 and 46% respectively (Fig. 1e). Conversely, the north-western corridor with a large number of provinces within the less outbreak prone C1 consists of the least urbanized regions. Namely, Tak, Kachanaburi and Mae Hong Son have urban population percentages at 24, 23 and 10% respectively (Fig. 1e), with only 23, 19 and 14% of urban land cover (Fig. 1d). This trend persists for a large proportion of provinces, where lower levels of urban land cover and urban populations correlate to lower levels of dengue case counts and epidemic persistence probabilities (Fig. 1a, Fig. 2).

**Fig. 3:**
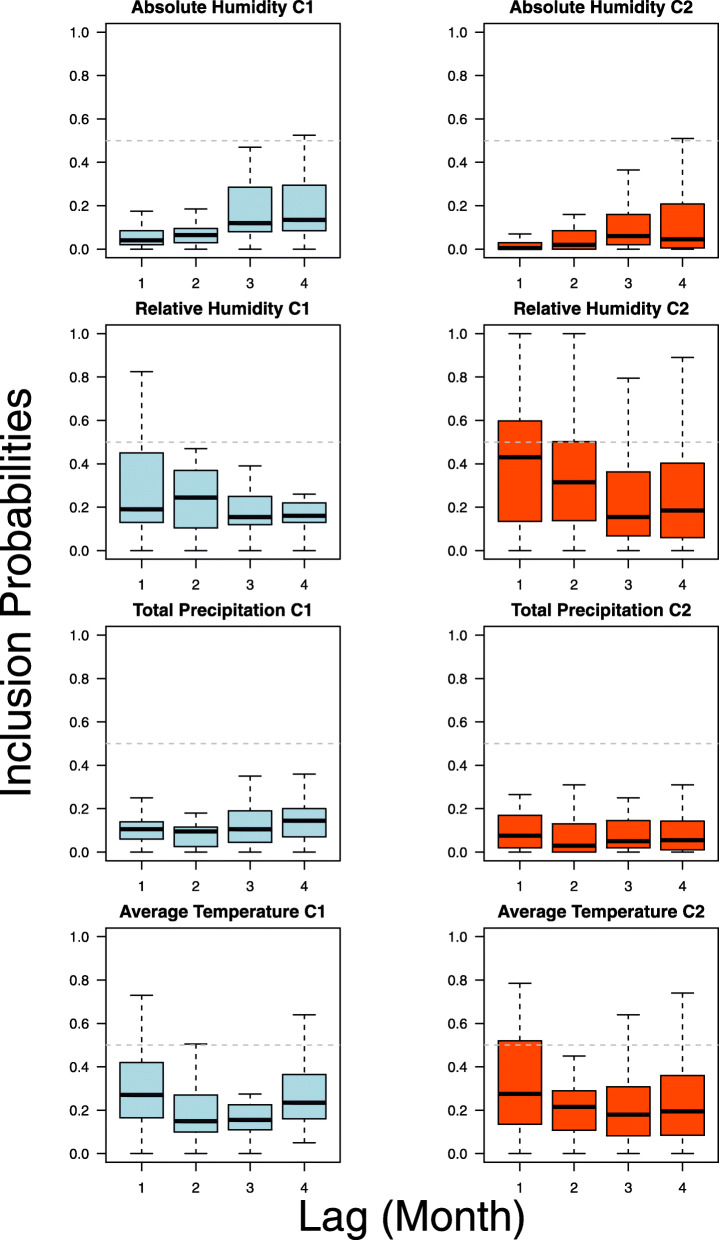
Variable importance of climatic variables on their effects on dengue epidemic potential across all provinces by epidemic cluster

Lastly, a duality in climatic forcing on regimes is indicated between the two clusters, with climatic forcing on dengue epidemics more notable in the epidemic prone C2 compared to the baseline C1 under LASSO analysis. To belabor, in C1, inclusion probabilities for climatic variables cross the 0.5 bound less than variables in C2, however, the inclusion probability interval for C2 were far wider which may be due to more provinces being classified within the cluster. In particular, absolute humidity, total precipitation, relative humidity, average temperature followed by total precipitation were included in increasing order across 1 to 4 lags for C1, while total precipitation, absolute humidity, average temperature, followed by relative humidity were included in increasing order across 1 to 4 lags for C2 (Fig. 3). Absolute humidity was included the least, with only 7 provinces having inclusion probabilities above the 0.5 cutoff, followed by relative humidity, total precipitation and average temperature. Average inclusion probabilities between clusters also indicate that absolute and relative humidity are more important in C1 for explaining epidemics, while average temperature and total precipitation are more important in explaining epidemics in C2 (Fig. 3). Lastly, reconstructing LASSO to only in-sample performance indicates good in-sample fit, with an average area under the ROC being 0.82 across provinces.

### Model assessment

Fitting the BRS indicated that a 3 lag specification was sufficient to account for residual autocorrelation in dengue time series across all regions. In general, the BRS specification was able to detect breakpoints which precede large rises in dengue case counts across provinces when estimated using the full set. It was able to detect these breakpoints even when restricting observations to only contemporaneous and past timepoints (Fig. 1c, Additional file 1). Concordance as measured by the area under the receiver operating characteristic curve (AUC-ROC) showed that there is agreement between other outbreak detection measures with an average AUC-ROC of 0.840 across provinces with more than 60% provinces producing an AUC-ROC larger than 0.8, indicating good outbreak classification concordance in the majority of provinces [30]. The full results for classification stability are provided in the Additional file 1.

## Discussion

Results indicate two dynamic epidemic clusters in Thailand from 2007 to 2018; a baseline cluster 1 (C1), with a large majority of epidemics occurring sporadically, and an epidemic prone cluster 2 (C2), where outbreaks occur on an almost yearly basis. In epidemic prone C2, results indicate that epidemics are more persistent and estimated epidemic persistence probabilities are higher (Fig. 2), compared to the baseline C1, where epidemics are less likely to persist over time. This result is consistent with a study analysing dengue outbreaks in dengue hyper-endemic Singapore, where epidemics are found to be less persistent compared to the baseline state [24].

For Thailand, central to policy planners are four suggestions. In provinces estimated to have high epidemic persistence, more resource allocation to vector control should be applied to those localities as heightened transmission counts are likely to occur in C2 over a longer period of time compared to C1. Minimizing dengue transmission in areas which have higher urban land cover and possess larger importation risk is also important, due to their epidemic prone nature, as elaborated below.

Clusters as estimated in this study showed key endogenous and exogenous drivers. Urban land cover and urban population, are especially prominent in Bangkok and its adjacent areas, where it is assigned to the epidemic prone C2 and there may be several ecological reasons for its occurrence. Urbanization is strongly associated with increased dengue incidence [31], and increased population density from urbanization leads to an increase in viral diversity and the number of possible dengue transmission chains [32]. Urban practices such as storing water in plastic containers also increase the breeding potential for the *Aedes aegypti* vector [31], which thereby increases the vector’s viability in these regions. The tendency of *Aedes aegypti* to live in human homes [31], also compounds transmissibility, due to the higher density of urban centers as compared to peri-urban and rural settings in other provinces. Our results corroborate with trends which have emerged from other dengue endemic countries such as Vietnam, where dengue transmission is concentrated in large, urbanized areas such as Ha Noi and Ho Chi Minh [33]. In totality, these factors may have induced the epidemic prone conditions in Bangkok and the surrounding locations.

The classification of Bangkok, Chiang Mai and Chiang Rai to epidemic prone C2, which are the provinces which correspondingly have the largest number of incoming flight passengers and largest number of case counts reported is also notable. Several key pathways may have led to elevated dengue transmission and outbreak risk within the region. Increased risk of dengue epidemics may result from importation of viraemic mosquitoes due to air travel from Thailand’s status as an air traffic hub [34]. Viraemic individuals may similarly result in dengue importation, as showed from the co-circulation of dengue serotypes within South East Asia and the importation of different serotypes into Thailand [34, 35]. As a result, the status of Bangkok, Chiang Mai and Chiang provinces being air travel hubs may have further heightened the importation risk of new dengue serotypes or genotypes, increase viral diversity, cause selection of strains of higher fitness which the local population are not exposed to previously and potentially seed new epidemics [36].

The influence of climate on mosquito breeding potential and the transmissibility of dengue is well-studied [37, 38]. In this paper, varying levels of importance are ascribed to climatic factors in determining dengue epidemic probability, with 1–4 months lagged average temperature and total precipitation having the highest level of inclusion. This constrasts prior results of climate not being able to substantially explain dengue epidemics in Singapore [24] and may be due to the limited geographical range of Singapore as compared to Thailand. In Thailand, the importance of climate on dengue epidemics is observed across both epidemic prone C2 and baseline C1, which suggests that climatic factors do affect probabilities of epidemics occurring with some degree (Fig. 3, Additional file 1). However, as inclusion probability intervals overlap across both C1 and C2 for variables on all lags, there is only evidence that climate provides a signal to dengue epidemic risk temporally, rather than affect said risk through the epidemic clusters as classified. Studies have also shown the effects of longitude and latitude on dengue transmission potential, through climate and thus vector breeding potential [14]. Although the epidemic clusters detected do have some geographical patterns (Fig. 1), the dispersion of provinces through the entirety of the Thailand may have written off potential distinguishing climatic factors between clusters.

Our study centrally extends previous work conducted on dengue in Thailand [9, 14] by examining dengue epidemic probabilities and outbreaks over time as the quantity of interest, rather than dengue case counts. Compared to standard outbreak classification tools, BRS as developed in this paper, can be used to infer separable regimes and crucially, provide information on the duration and persistence of epidemics without pre-specified thresholds, even when only limited information on reported dengue cases at the provincial level is provided. It can capture the growth and decline of monthly dengue transmission counts across provinces in Thailand, and outbreak classification using BRS is also concordant with other benchmark methods in outbreak classification, which further validates the framework (Additional file 1). Cluster analysis is also robust, according to majority consensus of cluster validity indices in our analysis, which then allows us to draw links between our analysis and biologically relevant factors. Lastly, climate is associated with dengue epidemics but with varying levels of importance; there are inclusions of at least one lagged climatic variable being associated to epidemics across time in all provinces.

Several limitations are present in this paper. First, using monthly dengue case counts as demonstrated in this paper, would not be suitable for vector control purposes as policy planners would want to ramp up vector control as soon as an outbreak is classified or forecasted. Forecasting outbreaks under only 1 step or more ahead monthly frequencies is only possible due to monthly frequencies being the model input for BRS in this study and hence would not be timely enough for surveillance purposes. However, BRS could easily be applied to weekly nowcasts, forecasts and inference as demonstrated by other infectious disease applications [23, 24]. The reporting rate of dengue case counts may have been driven by province level variations in healthcare accessibility, which may affect our estimates of epidemic persistence and classifications. However, these estimates are likely skewed towards the null due to under-reporting likely to occur rather than over-reporting of dengue case counts. The variations in reporting rate are also minimal as estimated within this paper. Number of asymptomatic dengue cases are also not available, but is not likely to affect the results as the primary parameters of interest are regimes, rather than predicted case counts. Reported number of dengue haemorrhagic fever and dengue shock syndrome cases, while also available, was not used as a dependent due to zero-inflation. Further work should develop and assess models which can generalize the BRS to such settings. Serotype composition and number of individuals travelling across land routes are important factors which may influence dengue outbreaks, but are not available. Future studies should attempt to study the relation between outbreaks and these covariates. Lastly, as data on biologically relevant factors such as urban land cover, urban population, and flight passengers are not available at the same frequency as dengue case counts, their effects on regimes and outbreak clusters can only be conducted post estimation of BRS.

## Conclusion

Being able to classify and infer the risk and persistence of dengue outbreaks is important, as significant health risks surface from these events. Contemporaneously classifying outbreaks, understanding outbreak characteristics and the drivers of these outbreaks is important for public health policy, as it provides policy makers with an indicator to ramp up the delivery of care and vector control to those localities. Our study found that provinces with a large number of case counts, urban population, urban land cover and incoming flight passengers are associated to the outbreak prone cluster of dengue. Climate has an effect on determining outbreak probabilities, but have less influence on whether they belong to the outbreak prone cluster. To the best of the authors’ knowledge, this is the first study to classify dengue outbreaks, estimate transition probabilities and show the clustering of epidemics on a national level in Thailand. The methods developed are also easily extendable to any infectious disease with case counts recorded over a substantial period of time.

## Supplementary Information

**Additional file 1.**

## Data Availability

The datasets used and/or analysed during the current study available from https://github.com/juetaoLim/regimeSwitchTH.

## Abbreviations

BRS: Bayesian Regime Switching
LASSO: Least Absolute Shrinkage and Selection Operator
AUC: Area Under the Curve
ROC: Receiver Operating Characteristics curve
PAM: Partitioning Around Medoids
