# Supplementary material for: Revealing two dynamic dengue epidemic clusters in Thailand
Source: BMC Infect Dis. 2020 Dec 4;20:927. doi: 10.1186/s12879-020-05666-4 (PMC7718674; doi:10.1186/s12879-020-05666-4)
Supplement: Supplementary file 1 — Additional file 1. [file 12879_2020_5666_MOESM1_ESM.pdf]

Technical Appendix

October 11, 2020

Contents

1 Model Specification (Bayesian Regime Switching) 1

2 Model Estimation (Bayesian Regime Switching) 2

3 Alternative Classification Schemes 3

4 Clustering Assessment Metrics 4

5 Covariate Values 6

6 Model Assessment: Cluster Validity 13

7 Model Assessment: Dissimilarity Matrix 14

8 Summary of Regime Probabilities across Time 15

9 Epidemic Classification by Provinces 16

10 Receiver Operating Characteristic Curves by Provinces 92

11 Climatic Epidemic Potential by Provinces 157

1 Model Specification (Bayesian Regime Switching)

We consider a 2 state fixed transition probability Markov switching model of order p with switching autoregressive parameters as follows,

$$y_t = \beta_{1,s_t} + y_{t-1}\beta_{2,s_t} + \cdots + y_{t-p}\beta_{p,s_t} + \epsilon_t$$

$$\epsilon_t \sim N\left(0, \sigma^2_{s_t}\right)$$

The transition matrix $\boldsymbol{\xi}$ characterizes probabilities of switching between two states at each time point:

$$\boldsymbol{\xi}_t = \begin{bmatrix} p_{11,t} & p_{12,t} \\ p_{21,t} & p_{22,t} \end{bmatrix}$$

We estimate sequentially $\theta = \{\beta_{s_t}, \sigma^2_s, s_t, \boldsymbol{\xi}\}$ by placing the following priors on parameters, states and transition matrix:

$$\beta_s \sim N\left(\beta_0, \sigma^2_s \mathbf{P}_0\right)$$

with $\beta_0 = 0$ and $\sigma_s^2 \mathbf{P}_0 = \text{diag}(100)$ for our parameters to be centered around 0 and having a wide variance to impose noninformativeness.

$$\sigma_s^2 \sim IG\left(\frac{v_0}{2}, \frac{v_0 \sigma_0^2}{2}\right)$$

with $v_0 = v_0$, $\sigma_0^2 = 1$ to yield a non-informative inverse-gamma prior distribution for σ_s

$$\boldsymbol{\xi} \sim Dir(e_{j1}, \ldots, e_{jk})$$

with j denoting number of states and $e_{11} = 5, e_{12} = 15$ for the two state BRS to impose the belief that dengue transmissions are more likely to stay within their own regime rather than move to another regime.

2 Model Estimation (Bayesian Regime Switching)

To estimate our model, we placed normal-inverse gamma priors on our regression and variance parameters $\beta_{s_t} \sim N(0, 100)$ and $\sigma_{s_t} \sim IG(0.5, 0.5)$. Regimes are sampled using multi-move Gibbs sampling via the Carter-Kohn recursion (Albert and Chib 1993) with up to 3 regimes considered. The recursion first conducts a forward pass filtering step to infer the probability of arriving at a regime given the first t observations for all $t \in \{1, \ldots, T\}$, where T denotes the final time point. Next, the backward pass smoothing step provides the probabilities of being in a regime at t given $\{t + 1, \ldots, T\}$. The second step allows the recursion to consider the full data likelihood and provides assignment of datapoints to each regime, which were then post-hoc labelled based on their behavior. The Dirichlet prior $\zeta_1 \sim Dir(25, 5), \zeta_2 \sim Dir(5, 25)$ was also placed on each row of the transition matrix, dictating the belief that the probability of staying within one regime is higher than the probability of transitioning to another. We impose the identifiability constraint $\sigma_{s_t=\text{epidemic}} > \sigma_{s_t=\text{endemic}}$ to account for label switching, as dengue transmission counts are expected to fluctuate more in absolute numbers within an epidemic compared to endemic period. These steps are nested within a Gibbs sampling framework due to prior-likelihood conditional conjugacy. Gibbs sampling for BRS is run with 50000 iterations with a burn-in of 5000.

The Gibbs sampler proceeds by sequentially sampling the conditional posteriors for each of our parameters specified above.

First, we conduct multi-move sampling on s_t with the conditional posteriors given by (3), with $\Pr(\mathbf{S}_{1:t} \mid Y_t)$ obtained using the Hamilton filter and $\prod_{k=1}^{T-1} \Pr(S_k \mid S_{k+1}, Y_t)$ through the Carter-Kohn recursion as described by Kim and Nelson 1999 Kim and Nelson 1999

$$\Pr(\mathbf{S}_{1:t} \mid \theta_{-S}) = \Pr(S_t \mid Y_t) \prod_{k=1}^{T-1} \Pr(S_k \mid S_{k+1}, Y_t) \tag{1}$$

Next, to sample the time varying transition probability matrix $\xi_\mathbf{t}$, we note that for each time point $\mathbf{S} = s_1 \ldots s_j$ has the complete likelihood

$$p(\mathbf{S} \mid \xi_t) \ = \prod_{j=1}^{K} \prod_{k=1}^{K} \xi_{jkr}^{N_{jk,t}}(s)$$

where $N_{jk,t}$ denotes counts for the transition from j to k states at time t, we derive:

$$p(\xi_t \mid \mathbf{S}) \propto Dir(e_{j1} + N_{j1,t}(s), \ldots, e_{jk} + N_{jk,t}(s)) \tag{2}$$

For σ_s^2, given the state sampled from (1) conjugacy between the inverse gamma prior and normally distributed likelihood yields an inverse gamma posterior with shape and rate parameters $v_1/2$ and v_1/σ_1^2 respectively,

$$\begin{aligned} p\left(\sigma_s^2 \mid \theta_{-\sigma^2}\right) &\propto p\left(Y \mid \theta_{-Y}\right) p\left(\beta \mid \sigma_s^2, Y\right) p\left(\sigma_s^2\right) \\ &\sim IG\left(v_1/2, v_1 \sigma_1^2\right) \end{aligned} \tag{3}$$

$$
\begin{aligned}
v_1 &= v_0 + (n-p) \\
\sigma_1^2 &= v_1^{-1}\left(v_0\sigma_0^2 + \mathbf{A}_s + \mathbf{B}_s\right) \\
\mathbf{A}_s &= (y - X\beta_s^*)'\,(y - X\beta_s^*) \\
\mathbf{B}_s &= (\beta_s^* - \beta_0)'\,\mathbf{P_0}\,(\beta_s^* - \beta_0)
\end{aligned}
$$

Similarly for β, given the state sampled from (1) conjugacy between the normally distributed prior and normally distributed likelihood yields a normally distributed posterior with mean β_s^* and variance $\sigma_\beta^2\mathbf{P}_1$

$$
\begin{aligned}
p\left(\beta_s \mid \theta_{-\beta_s}\right) &\propto p\left(Y \mid \beta_s, \sigma^2, s_t\right) p\left(\beta_s \mid s_t, \sigma^2\right) \\
&\sim N\left(\beta_s^*, \sigma_\beta^2\mathbf{P}_1\right) \qquad (4)\\
\mathbf{P}_1 &= (\mathbf{P}_0^{-1} + X_s'X_s)^{-1} \\
\beta_s^* &= \mathbf{P}_1\left(Y_s'X_s + \mathbf{P}_0\beta_{0,s}\right)
\end{aligned}
$$

Lastly, to prevent label switching, we place an identification restriction on the sigma parameter such that $\sigma_{1,S=2} > \sigma_{1,S=1}$

Steps (1) - (4) are iterated until convergence

3 Alternative Classification Schemes

The CUSUM framework may detect changes in the incidence of a health event across time. The d-month upper CUSUM incorporates the recent 7 months data, representing possible short to long term dependence in dengue transmission dynamics, using a specific control statistic, C_t^+ (5) with $C_{t-d}^+ = 0$ and $\tilde{y_{(7)}}$ and $\tilde{s_{(7)}}$ referring to running mean and sample variance of dengue counts from y_{t-d-7},...,y_{t-d-1}. The parameter k represents the minimum standardized difference, which must be exceeded for a data point being included in the CUSUM calculation (Cowling et al. 2006). When the control statistic is greater than $\Phi_{1-\alpha}$, the $1-\alpha$ quantile of the standard normal cumulative distribution function, the current timepoint is classified as an epidemic.

$$
C_t^+ = \max\{0, \frac{y_t - \tilde{y_{(7)}}}{\tilde{s_{(7)}}} - k + C_{t-1}^+\} \qquad (5)
$$

The simple regression model classifies outbreaks by using the running mean and sample variance from the preceding m months. An epidemic alert is generated if the t statistic exceeds the $100(1-\alpha)^{th}$ percentile of the Student's t-distribution with m - 1 degrees of freedom. (6)

$$
t^* = \frac{y_t - \tilde{y_m}}{\tilde{s_m} * \sqrt{1 + 1/m}} \sim t_{m-1}(1-\alpha) \qquad (6)
$$

Assessment on measure agreement were done by pairwise comparison and constructing the receiver operating characteristic (ROC) curve for each province separately. The area under the ROC for each province is then computed to provide a general measure on classification concordance between the CUMSUM, simple regression model as well as BRS fitted on the full dataset.

Comparing CUSUM(d=2, k=1) and BRS, there is an average AUC-ROC of 0.840 across provinces with more than 60% provinces producing an AUC-ROC larger than 0.8, indicating good outbreak concordance in the majority of provinces (Fawcett 2006).AUC-ROC is highest in Samut Sakhon (0.989), Chumphon (0.978) and lowest for Phang Nga (0.438) and Phayao (0.451). The simple regression model was also run sequentially with data inputs increasing from 5 to 11 months, with outbreak classification against BRS conducted at each step. The AUC-ROC aggregated from all provinces increases from 0.780 to 0.869 when data input for the simple regression model increased from 5 to 11 months respectively. Increasing the data input into the simple regression may have increased the concordance with BRS as the BRS accounts for the full history

of dengue cases in each province. Using the simple regression model with dengue counts of the preceding 5 months, we ascribe the highest concordance between simple regression and BRS in Ang Thong (0.956) and Chiang Rai (0.951), while Khon Kaen and Phayao showed lowest concordance producing AUC-ROC 0.523 and 0.550 respectively. However, the overall concordance is good with the average AUC-ROC across provinces being 0.815. Simple regression classifies outbreaks with less similarity compared to CUSUM when compared the the BRS.

4 Clustering Assessment Metrics

Clustering validity indices were used to determine the optimal number of clusters. Namely, we used the Silhouette index, Calinski-Harabasz index, Davies-Bouldin index, Dunn Index and COP index (Table 1). The optimal number of epidemic clusters were then determined by majority consensus across these indices. Temporal cluster stability was conducted by subsetting our dataset to different blocks. Namely, a starting point was randomly selected and we applied the DTW-PAM clustering algorithm for 1 to 10 clusters and 20 to 50 observations after the starting point. CVI was evaluated for each subsetted time series, with the classified clusters for each region matched against the classified clusters on the full time series.

Graphical analysis of the dissimilarity matrix indicates that the estimated epidemic probabilities across regions have clustering tendencies across time. 2 to 10 clusters were pre-specified and initially fit using DTW-PAM, with cluster validity indices computed on each iteration. Among the 6 cluster validity indices computed, majority consensus indicates that 2 was the optimal amount of clusters. Only the COP index indicated 10 as the optimal number of clusters. Cross-validating over 30 repetitions for cluster stability together with computing the cluster validity indices (CVIs) for each repetition showed that 2 clusters were the optimal number across samples under majority consensus of the CVIs.

Index	Metric	
Silhouette index	$s(i) = \frac{b(i)-a(i)}{max\{a(i),b(i)\}}$, if $\|C_i\| > 1$	(Rousseeuw 1987)
Calinski-Harabasz index	$\frac{\text{SS}_b}{\text{SS}_w} \times \frac{\text{N-K}}{\text{K-1}}$	(Caliński and Harabasz 1974)
Davies-Bouldin index	$\frac{1}{N} \sum_{i=1}^{N} D_i$	(Davies and Bouldin 1979)
Dunn index	$\frac{min_{1<i<j<m} \delta(C_i, C_j)}{max_{1<k<m} \Delta_k}$	(Dunn 1973)
COP index	$\frac{1}{\|Y\|} \sum_{C \in P^Y} \|C\| \frac{\text{intra}_{cop}}{\text{inter}_{cop}}$	(Gurrutxaga et al. 2010)

Table 1: Cluster Validity Indexes

5 Covariate Values

	Province	Municipal Population	Non-Municipal Population	Percentage Municpal
1	Amnat Charoen	64092.00	292683.00	17.96
2	Ang Thong	72543.00	196273.00	26.99
3	Bangkok	6320174.00	0.00	100.00
4	Buri Ram	204716.00	1283562.00	13.76
5	Chai Nat	46244.00	312636.00	12.89
6	Chaiyaphum	176838.00	917485.00	16.16
7	Chanthaburi	156317.00	317904.00	32.96
8	Chachoengsao	131600.00	500933.00	20.80
9	Chiang Mai	387537.00	1084866.00	26.32
10	Chiang Rai	196249.00	938345.00	17.30
11	Chon Buri	555285.00	463291.00	54.52
12	Chumphon	82649.00	362466.00	18.57
13	Kalasin	187426.00	731806.00	20.39
14	Kamphaeng Phet	86788.00	583412.00	12.95
15	Kanchanaburi	171834.00	561421.00	23.43
16	Khon Kaen	375058.00	1352406.00	21.71
17	Krabi	54816.00	279594.00	16.39
18	Lampang	223624.00	555591.00	28.70
19	Lamphun	98728.00	312503.00	24.01
20	Loei	101162.00	502697.00	16.75
21	Lop Buri	119183.00	623850.00	16.04
22	Mae Hong Son	21031.00	187804.00	10.07
23	Maha Sarakham	114236.00	827935.00	12.12
24	Mukdahan	46854.00	263101.00	15.12
25	Nakhon Nayok	30401.00	210529.00	12.62
26	Nakhon Pathom	218441.00	590621.00	27.00
27	Nakhon Phanom	93977.00	589715.00	13.75
28	Nakhon Ratchasima	535459.00	2014745.00	21.00
29	Nakhon Sawan	229647.00	859012.00	21.09
30	Nakhon Si Thammarat	259190.00	1258741.00	17.07
31	Nan	61345.00	393265.00	13.49
32	Narathiwat	162031.00	499082.00	24.51
33	Nong Bua Lam Phu	110026.00	371476.00	22.85
34	Nong Khai	182530.00	699181.00	20.70
35	Nonthaburi	538235.00	272019.00	66.43
36	Pathum Thani	310484.00	358558.00	46.41
37	Pattani	115640.00	478727.00	19.46
38	Phangnga	33907.00	200126.00	14.49
39	Phatthalung	72575.00	423664.00	14.62
40	Phayao	110612.00	391203.00	22.04
41	Phetchabun	158840.00	799297.00	16.58
42	Phetchaburi	155707.00	275775.00	36.09
43	Phichit	117217.00	454716.00	20.50
44	Phitsanulok	154643.00	637009.00	19.53
45	Phra Nakhon Si Ayutthaya	238233.00	483408.00	33.01
46	Phrae	106537.00	384979.00	21.68
47	Phuket	100224.00	138773.00	41.94
48	Prachin Buri	67955.00	337336.00	16.77
49	Prachuap Kiri Khan	162630.00	280490.00	36.70
50	Ranong	30321.00	130639.00	18.84
51	Ratchaburi	241572.00	546953.00	30.64

52	Rayong	205140.00	314942.00	39.44
53	Roi Et	151518.00	1100598.00	12.10
54	Sa Kaeo	71235.00	412633.00	14.72
55	Sakon Nakhon	145609.00	890569.00	14.05
56	Samut Prakarn	640311.00	374138.00	63.12
57	Samut Sakhon	191973.00	265105.00	42.00
58	Samut Songhkram	47763.00	156261.00	23.41
59	Saraburi	223313.00	345064.00	39.29
60	Satun	39887.00	207440.00	16.13
61	Si Sa Ket	148293.00	1254525.00	10.57
62	Sing Buri	68506.00	164192.00	29.44
63	Songkhla	404105.00	846798.00	32.30
64	Sukhothai	115698.00	476226.00	19.55
65	Suphanburi	147902.00	706614.00	17.31
66	Surat Thani	268075.00	599313.00	30.91
67	Surin	103864.00	1221830.00	7.83
68	Tak	116714.00	367642.00	24.10
69	Trang	118649.00	475094.00	19.98
70	Trat	49746.00	168031.00	22.84
71	Udon Ratchathani	266448.00	1419852.00	15.80
72	Udon Thani	396016.00	1063081.00	27.14
73	Uttaradit	98870.00	364244.00	21.35
74	Utthai Thani	54184.00	249448.00	17.84
75	Yala	114621.00	300371.00	27.62
76	Yasothon	60734.00	497796.00	10.87

Table 2: Values for key urbanisation metrics across provinces

	Province	Urban Land Percentage	Incoming Flights	Domestic Flights
1	Amnat Charoen	0.19		
2	Ang Thong	1.25		
3	Bangkok	63.17	36974652.00	12400214.00
4	Buri Ram	0.87	86189.00	83500.00
5	Chai Nat	0.57		
6	Chaiyaphum	0.37		
7	Chanthaburi	0.23		
8	Chachoengsao	1.04		
9	Chiang Mai	0.72	4283603.00	2963344.00
10	Chiang Rai	1.28	1082499.00	931965.00
11	Chon Buri	7.22		
12	Chumphon	0.39	38424.00	38420.00
13	Kalasin	0.37		
14	Kamphaeng Phet			
15	Kanchanaburi	0.19		
16	Khon Kaen	0.74	739110.00	720742.00
17	Krabi	0.25	1767276.00	925567.00
18	Lampang	0.59	135299.00	132660.00
19	Lamphun	1.31		
20	Loei	0.18	114020.00	114009.00
21	Lop Buri	0.95		
22	Mae Hong Son	0.14	43063.00	42865.00
23	Maha Sarakham	0.55		
24	Mukdahan	0.21		

25	Nakhon Nayok	0.05		
26	Nakhon Pathom	8.25		
27	Nakhon Phanom	0.41	308883.00	304443.00
28	Nakhon Ratchasima	0.84	122.00	122.00
29	Nakhon Sawan	0.82		
30	Nakhon Si Thammarat	0.36	591074.00	565780.00
31	Nan	0.15		
32	Narathiwat	0.67	260331.00	260233.00
33	Nong Bua Lam Phu	0.10		
34	Nong Khai	0.43		
35	Nonthaburi	40.69		
36	Pathum Thani	18.80		
37	Pattani	1.20		
38	Phangnga	0.02		
39	Phatthalung	0.23		
40	Phayao	0.47		
41	Phetchabun	0.32		
42	Phetchaburi	0.54		
43	Phichit	0.53		
44	Phitsanulok	0.55	252074.00	245692.00
45	Phra Nakhon Si Ayutthaya			
46	Phrae	0.54	36742.00	36742.00
47	Phuket	8.04	7007893.00	2671719.00
48	Prachin Buri			
49	Prachuap Kiri Khan		4122.00	4113.00
50	Ranong	0.33	43535.00	42526.00
51	Ratchaburi	1.05		
52	Rayong	2.59	469579.00	315045.00
53	Roi Et	0.88	171816.00	171784.00
54	Sa Kaeo	0.55		
55	Sakon Nakhon	0.35		
56	Samut Prakarn	35.70		
57	Samut Sakhon	22.24		
58	Samut Songhkram			
59	Saraburi	2.50		
60	Satun	0.33		
61	Si Sa Ket			
62	Sing Buri	2.13		
63	Songkhla	1.27	1806617.00	1612036.00
64	Sukhothai	0.65	49727.00	48609.00
65	Suphanburi	1.12		
66	Surat Thani	0.48	2268480.00	1524558.00
67	Surin	0.67		
68	Tak	0.23	81978.00	80268.00
69	Trang	0.59	368976.00	368933.00
70	Trat		51243.00	41182.00
71	Udon Ratchathani	0.69	813033.00	789740.00
72	Udon Thani	0.69	1083855.00	1045890.00
73	Uttaradit	0.54		
74	Utthai Thani	0.10		
75	Yala	0.58		
76	Yasothon	0.47		

Table 3: Values for key urbanisation and transport metrics across provinces

	Province	Reporting Rate	Reporting Rate (Sensitivity 1)	Reporting Rate (Sensitivity 2)
1	Amnat Charoen	0.00	0.01	0.01
2	Ang Thong	0.01	0.01	0.01
3	Bangkok	0.00	0.00	0.00
4	Buri Ram	0.00	0.00	0.00
5	Chai Nat	0.01	0.01	0.01
6	Chaiyaphum	0.01	0.01	0.01
7	Chanthaburi	0.01	0.01	0.01
8	Chachoengsao	0.01	0.01	0.01
9	Chiang Mai	0.01	0.01	0.01
10	Chiang Rai	0.01	0.02	0.02
11	Chon Buri	0.00	0.00	0.00
12	Chumphon	0.01	0.01	0.01
13	Kalasin	0.01	0.01	0.01
14	Kamphaeng Phet	0.01	0.01	0.01
15	Kanchanaburi	0.00	0.00	0.00
16	Khon Kaen	0.00	0.00	0.00
17	Krabi	0.01	0.01	0.01
18	Lampang	0.01	0.01	0.01
19	Lamphun	0.01	0.01	0.01
20	Loei	0.01	0.01	0.01
21	Lop Buri	0.01	0.01	0.01
22	Mae Hong Son	0.01	0.01	0.01
23	Maha Sarakham	0.01	0.01	0.02
24	Mukdahan	0.01	0.01	0.01
25	Nakhon Nayok	0.00	0.00	0.00
26	Nakhon Pathom	0.01	0.01	0.01
27	Nakhon Phanom	0.00	0.00	0.01
28	Nakhon Ratchasima	0.01	0.01	0.01
29	Nakhon Sawan	0.01	0.01	0.01
30	Nakhon Si Thammarat	0.01	0.01	0.01
31	Nan	0.01	0.01	0.01
32	Narathiwat	0.00	0.00	0.00
33	Nong Bua Lam Phu	0.00	0.00	0.00
34	Nong Khai	0.00	0.00	0.00
35	Nonthaburi	0.01	0.01	0.01
36	Pathum Thani	0.00	0.01	0.00
37	Pattani	0.00	0.00	0.00
38	Phangnga	0.00	0.00	0.00
39	Phatthalung	0.01	0.01	0.01
40	Phayao	0.01	0.01	0.01
41	Phetchabun	0.01	0.01	0.01
42	Phetchaburi	0.00	0.01	0.01
43	Phichit	0.01	0.02	0.02
44	Phitsanulok	0.01	0.01	0.01
45	Phra Nakhon Si Ayutthaya	0.01	0.01	0.01
46	Phrae	0.01	0.01	0.01
47	Phuket	0.00	0.01	0.01
48	Prachin Buri	0.01	0.01	0.01

49	Prachuap Kiri Khan	0.00	0.01	0.01
50	Ranong	0.00	0.00	0.00
51	Ratchaburi	0.01	0.01	0.01
52	Rayong	0.01	0.01	0.01
53	Roi Et	0.01	0.01	0.01
54	Sa Kaeo	0.01	0.01	0.01
55	Sakon Nakhon	0.00	0.00	0.00
56	Samut Prakarn	0.00	0.00	0.00
57	Samut Sakhon	0.00	0.00	0.00
58	Samut Songhkram	0.01	0.01	0.01
59	Saraburi	0.00	0.00	0.00
60	Satun	0.00	0.01	0.00
61	Si Sa Ket	0.05	0.06	0.06
62	Sing Buri	0.00	0.00	0.00
63	Songkhla	0.00	0.01	0.01
64	Sukhothai	0.01	0.01	0.00
65	Suphanburi	0.00	0.00	0.01
66	Surat Thani	0.00	0.00	0.00
67	Surin	0.01	0.01	0.01
68	Tak	0.01	0.01	0.01
69	Trang	0.00	0.00	0.00
70	Trat	0.01	0.01	0.01
71	Udon Ratchathani	0.00	0.00	0.01
72	Udon Thani	0.00	0.00	0.00
73	Uttaradit	0.01	0.01	0.01
74	Utthai Thani	0.01	0.01	0.01
75	Yala	0.00	0.00	0.00
76	Yasothon	0.00	0.01	0.01

Table 4: Values for dengue reporting rate using the time-series subsceptible-infected-recovered model

		Endemic (Persist)	Epidemic (Persist)	Epidemic (Exit)	Endemic (Exit)
1	Amnat Charoen	0.94	0.85	0.15	0.06
2	Ang Thong	0.94	0.85	0.15	0.06
3	Bangkok	0.96	0.83	0.17	0.04
4	Buri Ram	0.92	0.70	0.30	0.08
5	Chai Nat	0.93	0.80	0.20	0.07
6	Chaiyaphum	0.95	0.82	0.18	0.05
7	Chanthaburi	0.95	0.78	0.22	0.05
8	Chachoengsao	0.94	0.66	0.34	0.06
9	Chiang Mai	0.95	0.77	0.23	0.05
10	Chiang Rai	0.96	0.82	0.18	0.04
11	Chon Buri	0.95	0.85	0.15	0.05
12	Chumphon	0.91	0.78	0.22	0.09
13	Kalasin	0.97	0.93	0.07	0.03
14	Kamphaeng Phet	0.90	0.87	0.13	0.10
15	Kanchanaburi	0.90	0.75	0.25	0.10
16	Khon Kaen	0.93	0.81	0.19	0.07
17	Krabi	0.95	0.76	0.24	0.05
18	Lampang	0.97	0.83	0.17	0.03
19	Lamphun	0.96	0.86	0.14	0.04
20	Loei	0.97	0.79	0.21	0.03

21	Lop Buri	0.95	0.76	0.24	0.05
22	Mae Hong Son	0.97	0.75	0.25	0.03
23	Maha Sarakham	0.96	0.88	0.12	0.04
24	Mukdahan	0.94	0.81	0.19	0.06
25	Nakhon Nayok	0.97	0.83	0.17	0.03
26	Nakhon Pathom	0.93	0.84	0.16	0.07
27	Nakhon Phanom	0.96	0.90	0.10	0.04
28	Nakhon Ratchasima	0.94	0.72	0.28	0.06
29	Nakhon Sawan	0.95	0.77	0.23	0.05
30	Nakhon Si Thammarat	0.99	0.89	0.11	0.01
31	Nan	0.94	0.80	0.20	0.06
32	Narathiwat	0.99	0.75	0.25	0.01
33	Nong Bua Lam Phu	0.95	0.66	0.34	0.05
34	Nong Khai	0.98	0.74	0.26	0.02
35	Nonthaburi	0.97	0.76	0.24	0.03
36	Pathum Thani	0.95	0.77	0.23	0.05
37	Pattani	0.97	0.72	0.28	0.03
38	Phangnga	0.95	0.91	0.09	0.05
39	Phatthalung	0.92	0.80	0.20	0.08
40	Phayao	0.91	0.65	0.35	0.09
41	Phetchabun	0.97	0.74	0.26	0.03
42	Phetchaburi	0.95	0.80	0.20	0.05
43	Phichit	0.93	0.80	0.20	0.07
44	Phitsanulok	0.94	0.71	0.29	0.06
45	Phra Nakhon Si Ayutthaya	0.98	0.83	0.17	0.02
46	Phrae	0.96	0.87	0.13	0.04
47	Phuket	0.96	0.82	0.18	0.04
48	Prachin Buri	0.95	0.84	0.16	0.05
49	Prachuap Kiri Khan	0.85	0.90	0.10	0.15
50	Ranong	0.94	0.85	0.15	0.06
51	Ratchaburi	0.96	0.81	0.19	0.04
52	Rayong	0.96	0.87	0.13	0.04
53	Roi Et	0.95	0.69	0.31	0.05
54	Sa Kaeo	0.93	0.83	0.17	0.07
55	Sakon Nakhon	0.95	0.76	0.24	0.05
56	Samut Prakarn	0.96	0.85	0.15	0.04
57	Samut Sakhon	0.95	0.76	0.24	0.05
58	Samut Songhkram	0.97	0.81	0.19	0.03
59	Saraburi	0.93	0.62	0.38	0.07
60	Satun	0.98	0.88	0.12	0.02
61	Si Sa Ket	0.97	0.84	0.16	0.03
62	Sing Buri	0.93	0.70	0.30	0.07
63	Songkhla	0.95	0.88	0.12	0.05
64	Sukhothai	0.97	0.83	0.17	0.03
65	Suphanburi	0.91	0.82	0.18	0.09
66	Surat Thani	0.98	0.77	0.23	0.02
67	Surin	0.95	0.66	0.34	0.05
68	Tak	0.96	0.80	0.20	0.04
69	Trang	0.95	0.84	0.16	0.05
70	Trat	0.94	0.82	0.18	0.06
71	Udon Ratchathani	0.96	0.80	0.20	0.04
72	Udon Thani	0.95	0.78	0.22	0.05
73	Uttaradit	0.93	0.82	0.18	0.07
74	Utthai Thani	0.92	0.73	0.27	0.08

75	Yala	0.98	0.86	0.14	0.02
76	Yasothon	0.91	0.87	0.13	0.09

Table 5: Values for estimated outbreak statistics across provinces

6 Model Assessment: Cluster Validity

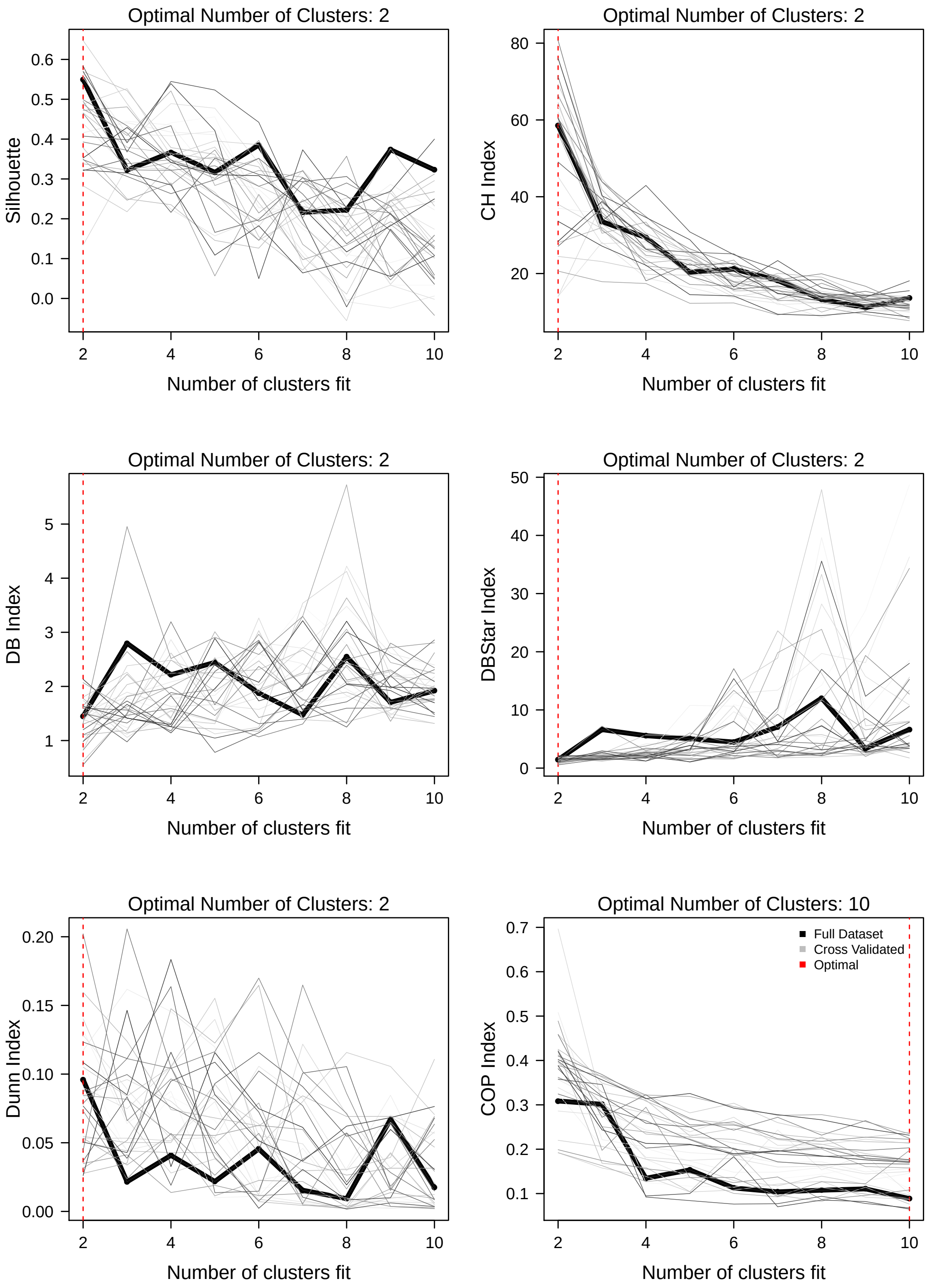

Figure 1: Cluster Validity Indices

7 Model Assessment: Dissimilarity Matrix

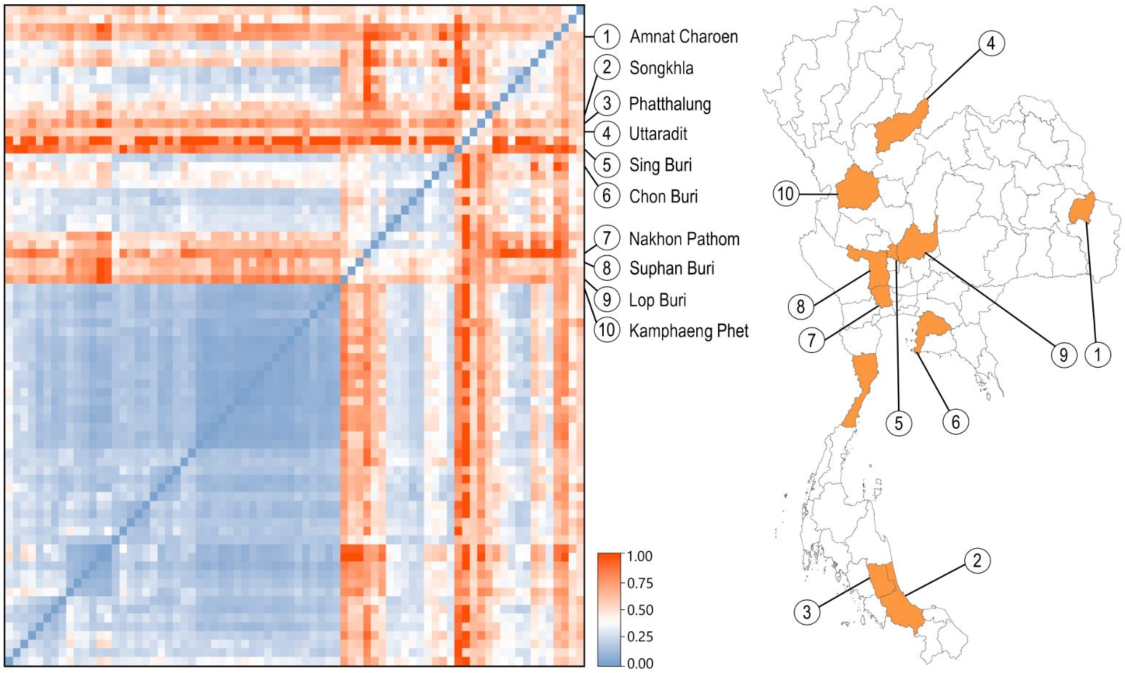

Figure 2: Dissimilarity Matrix

8 Summary of Regime Probabilities across Time

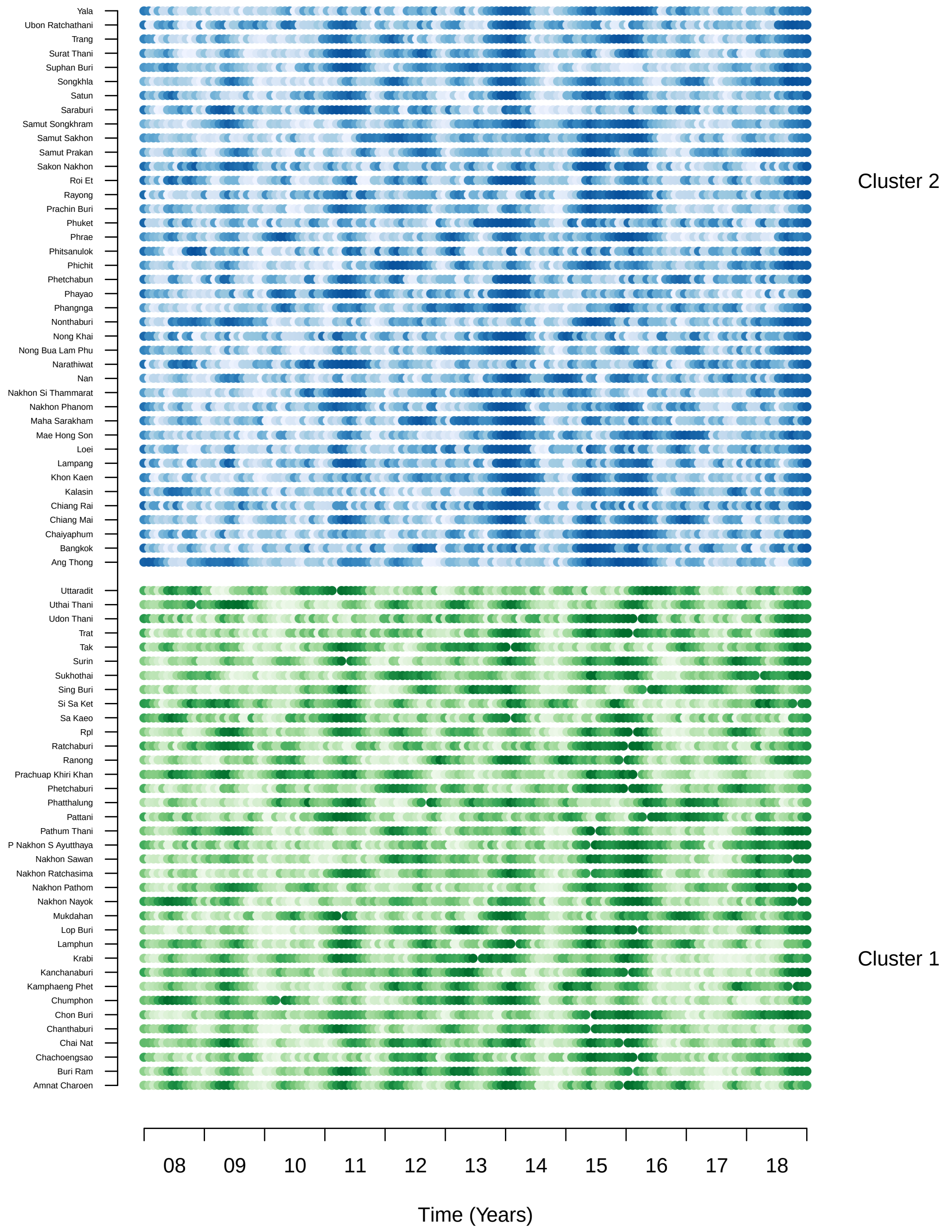

Figure 3: Hovmoller plot of epidemic regime probabilities across time

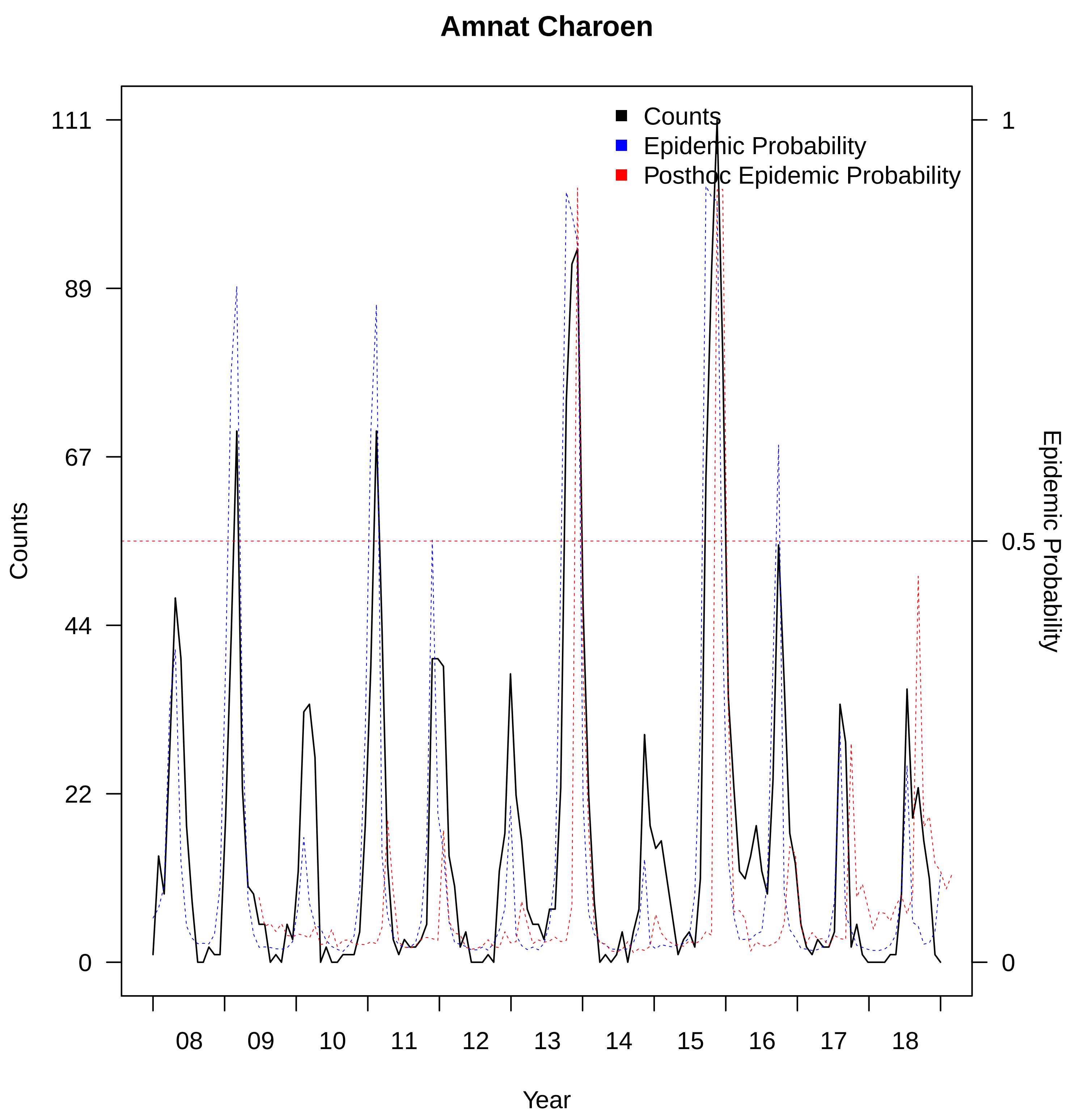

Amnat Charoen
Counts
Epidemic Probability
Posthoc Epidemic Probability
111
89
67
44
22
0
1
0.5
0
Counts
Epidemic Probability
08
09
10
11
12
13
14
15
16
17
18
Year

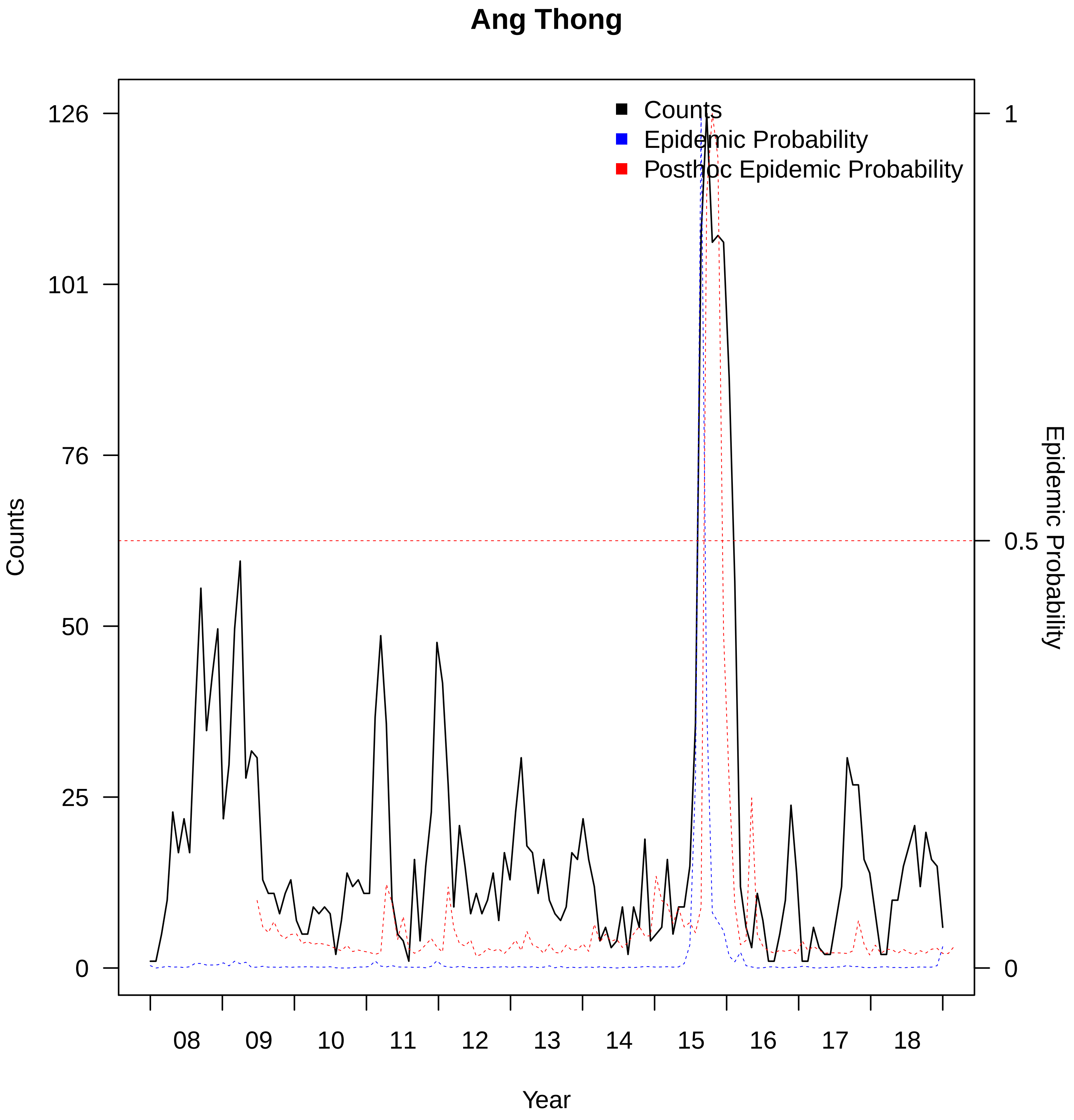

Ang Thong
Counts
Epidemic Probability
Posthoc Epidemic Probability
126
101
76
50
25
0
1
0.5
0
08
09
10
11
12
13
14
15
16
17
18
Year
Counts
Epidemic Probability

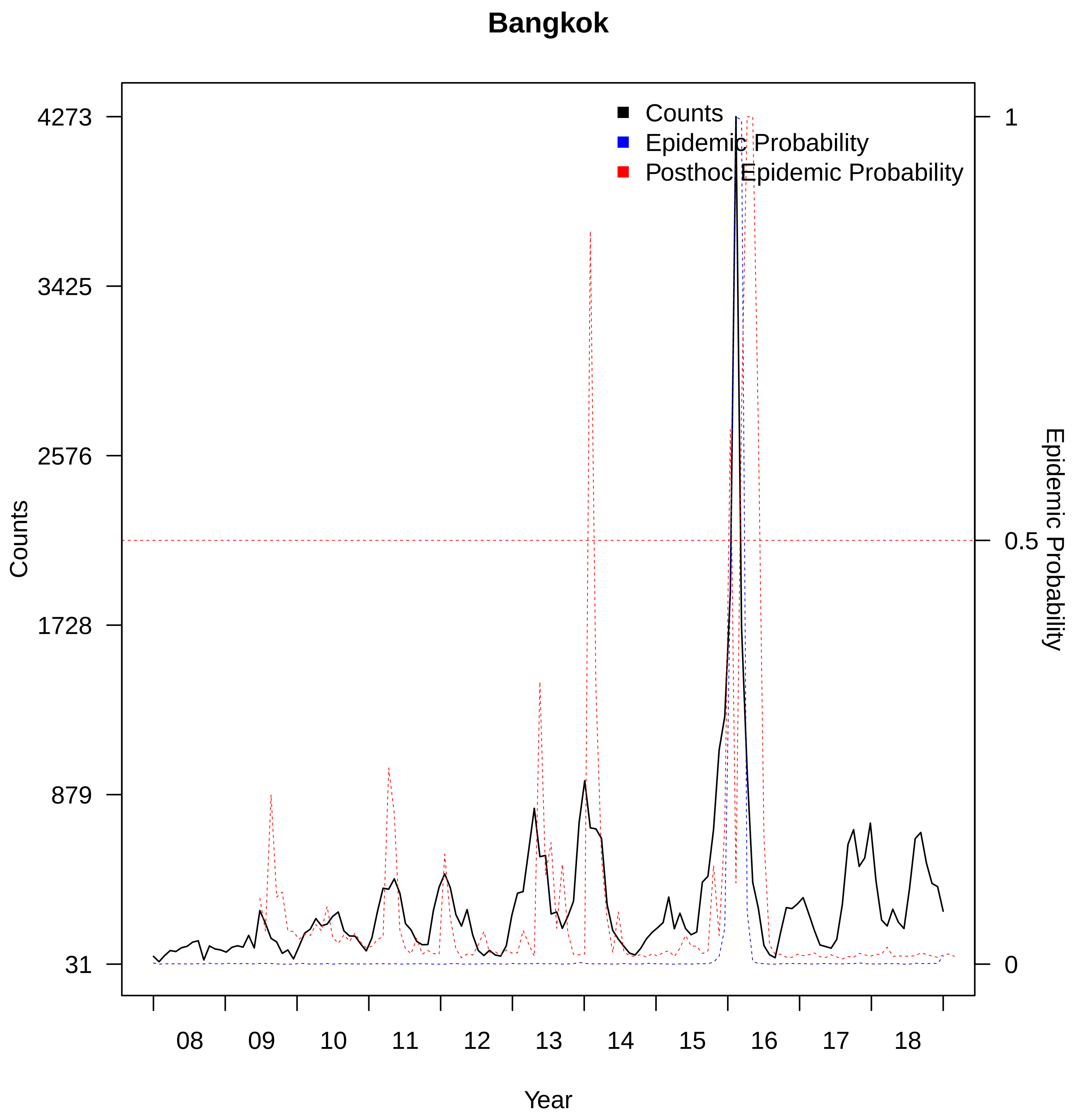

Bangkok
Counts
Epidemic Probability
Posthoc Epidemic Probability
4273
3425
2576
1728
879
31
Counts
1
0.5
0
Epidemic Probability
08
09
10
11
12
13
14
15
16
17
18
Year

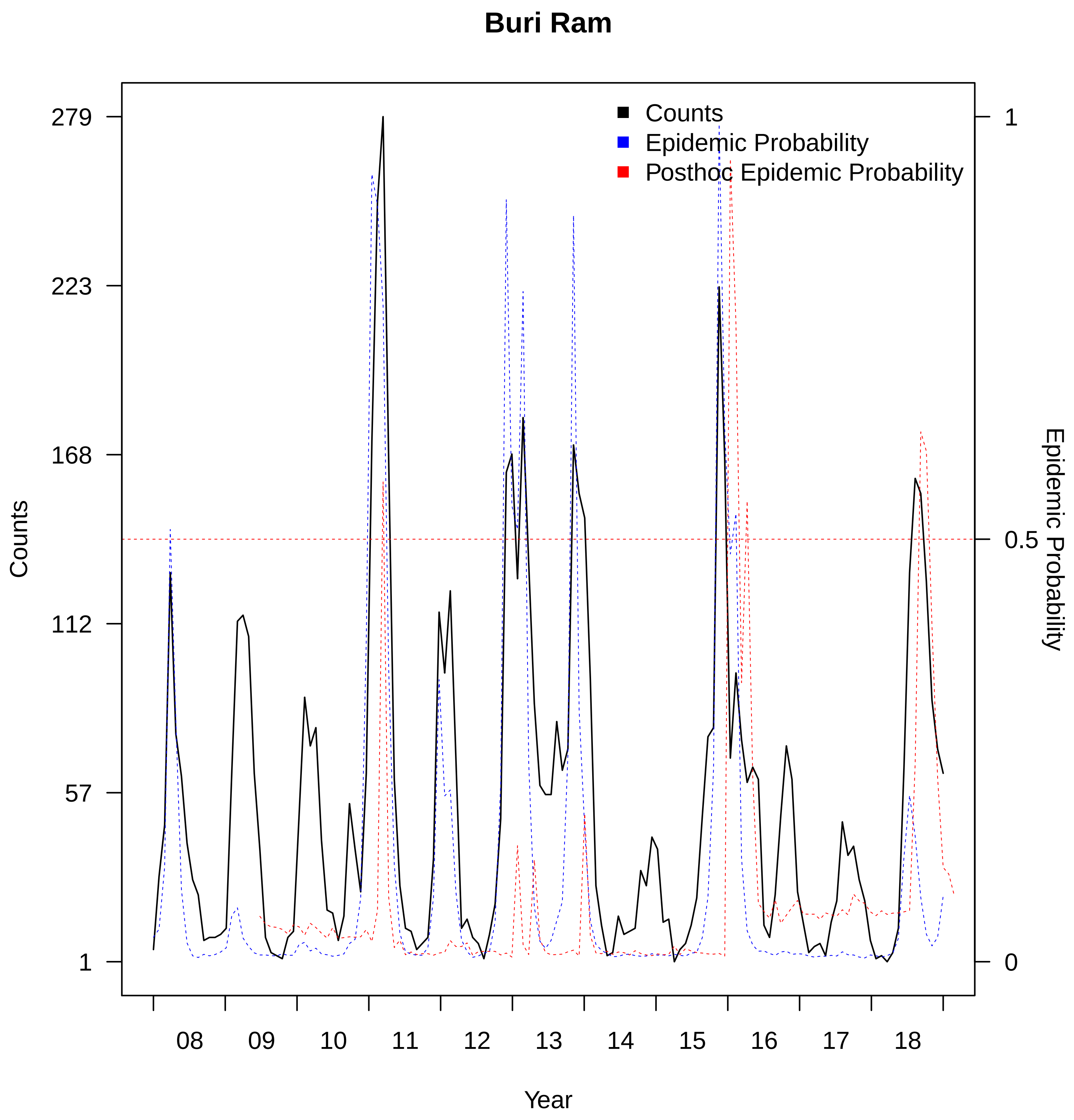

Buri Ram
Counts
Epidemic Probability
Posthoc Epidemic Probability
279
223
168
112
57
1
1
0.5
0
08
09
10
11
12
13
14
15
16
17
18
Year
Counts
Epidemic Probability

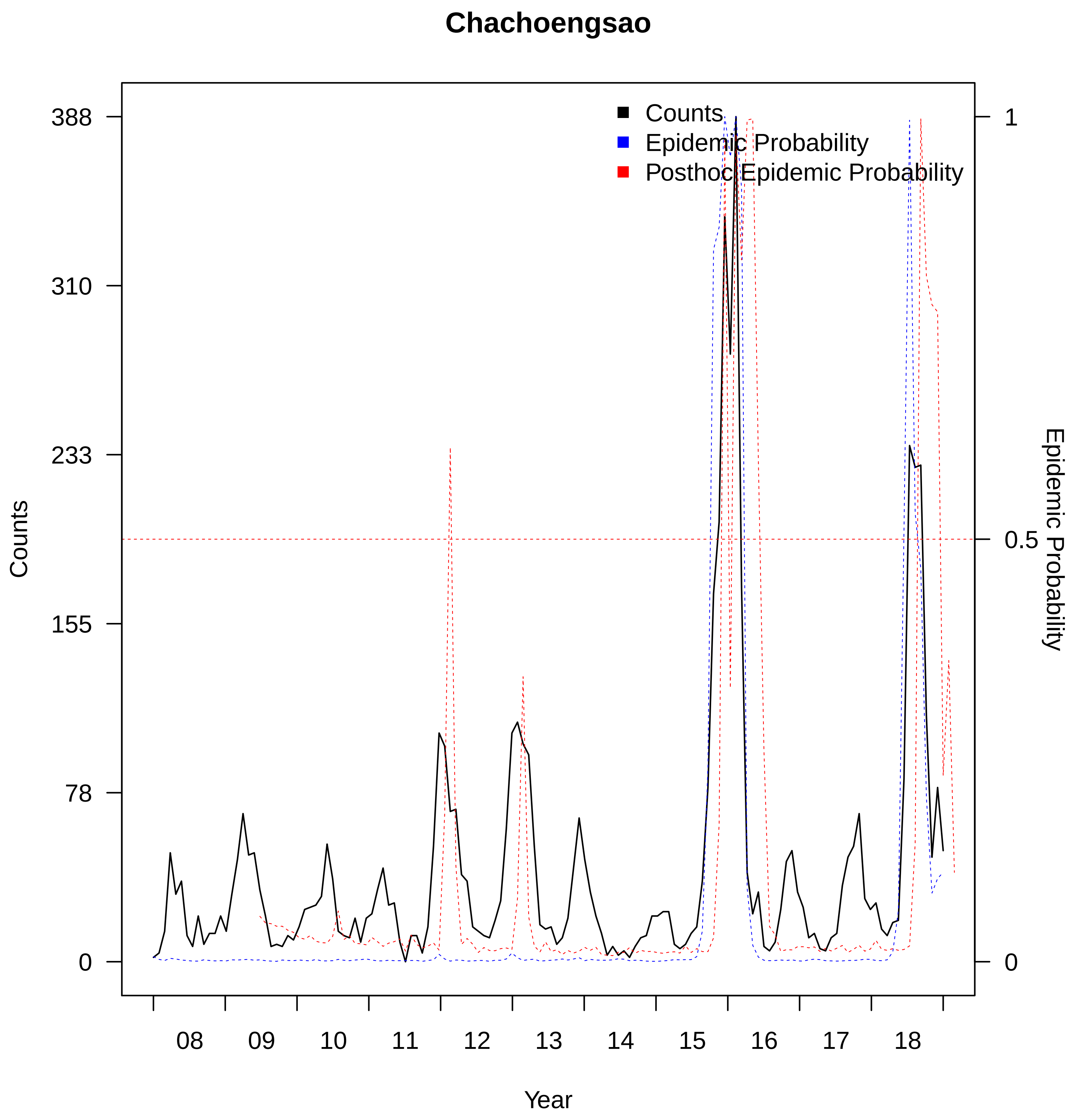

Chachoengsao
Counts
Epidemic Probability
Posthoc Epidemic Probability
388
310
233
155
78
0
1
0.5
0
Counts
Epidemic Probability
08
09
10
11
12
13
14
15
16
17
18
Year

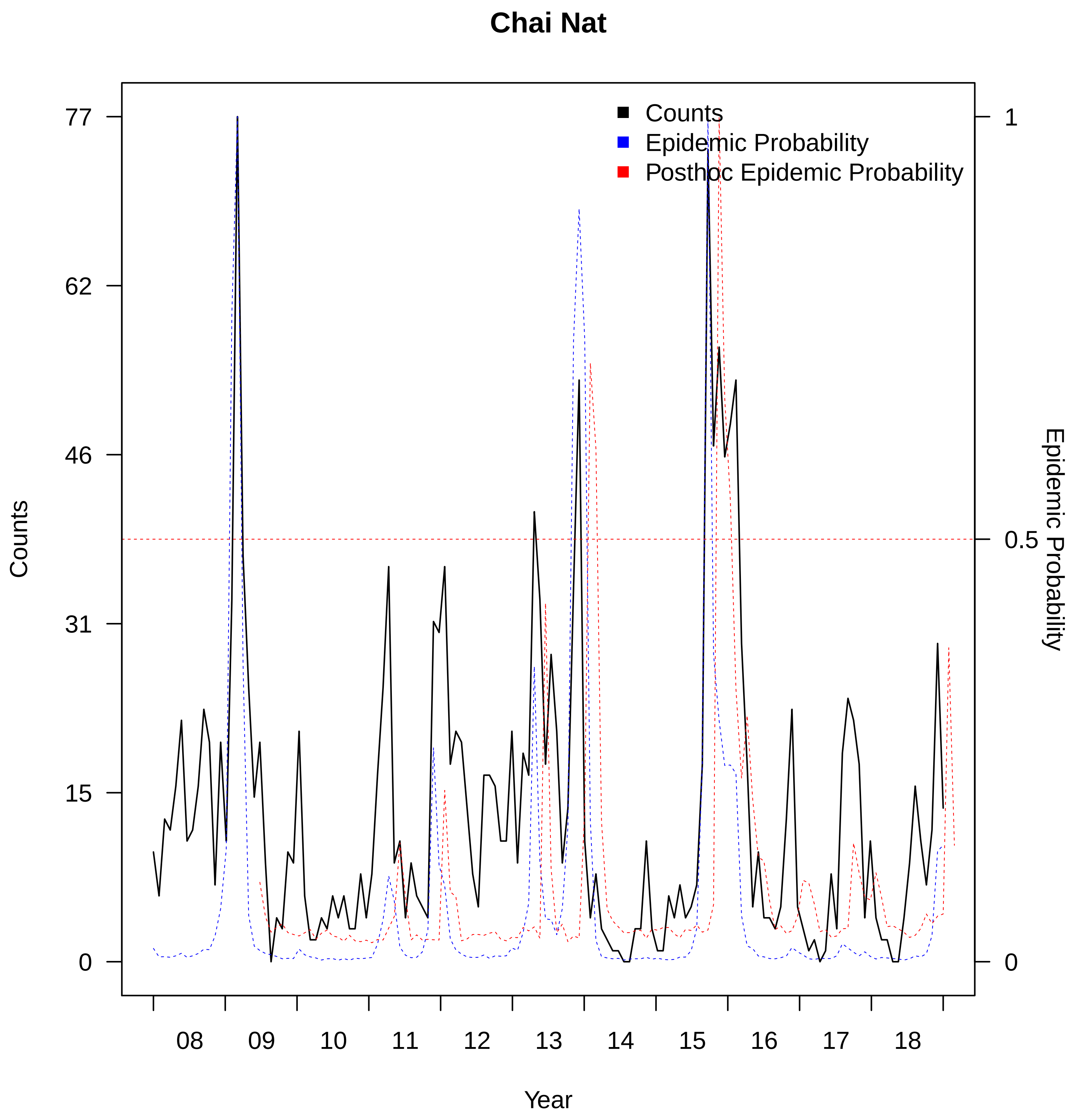

Chai Nat
Counts
Epidemic Probability
Posthoc Epidemic Probability
77
62
46
31
15
0
1
0.5
0
Counts
Epidemic Probability
08
09
10
11
12
13
14
15
16
17
18
Year

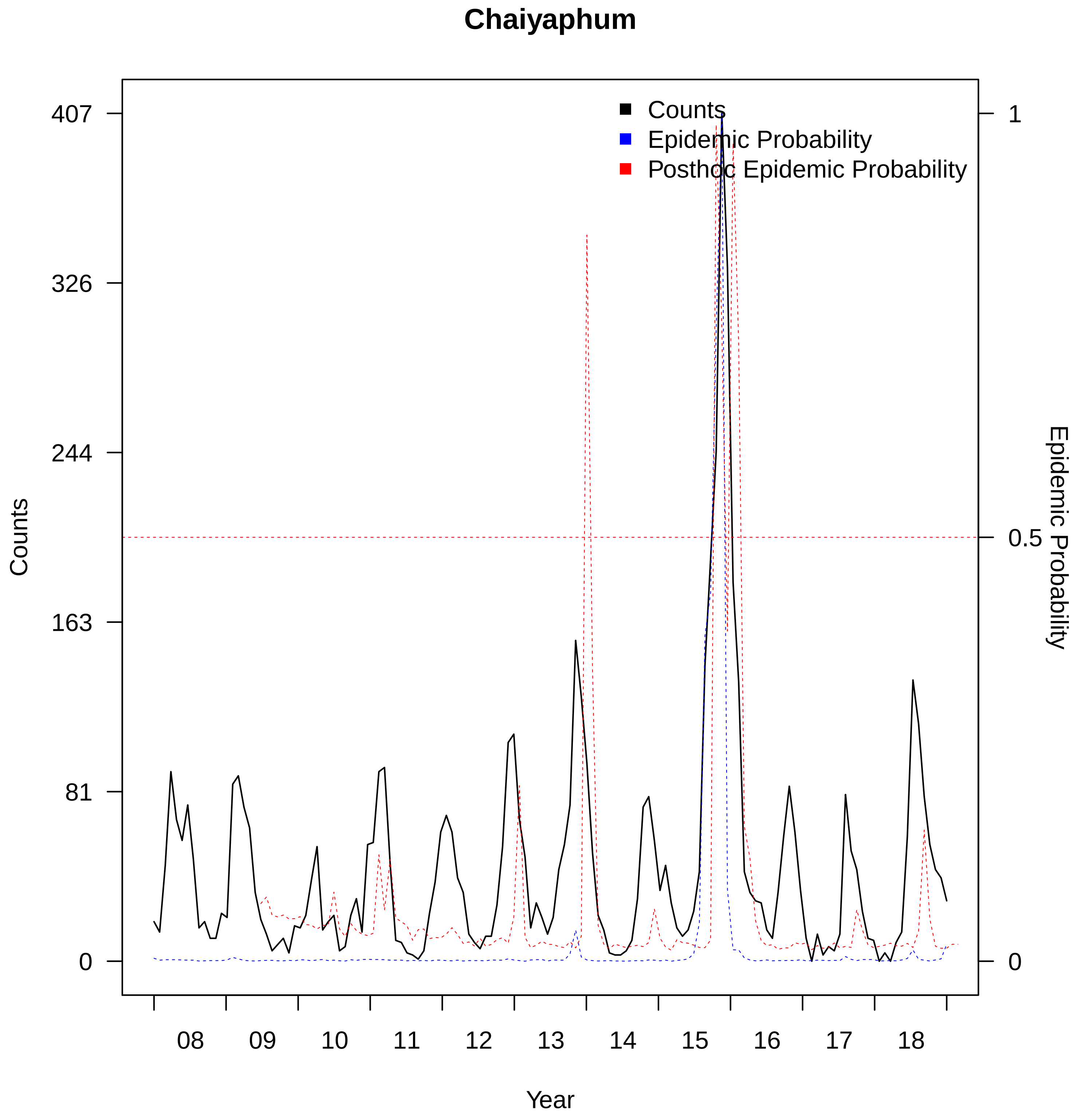
Chaiyaphum
Counts
Epidemic Probability
Posthoc Epidemic Probability
407
326
244
163
81
0
1
0.5
0
Counts
Epidemic Probability
08
09
10
11
12
13
14
15
16
17
18
Year

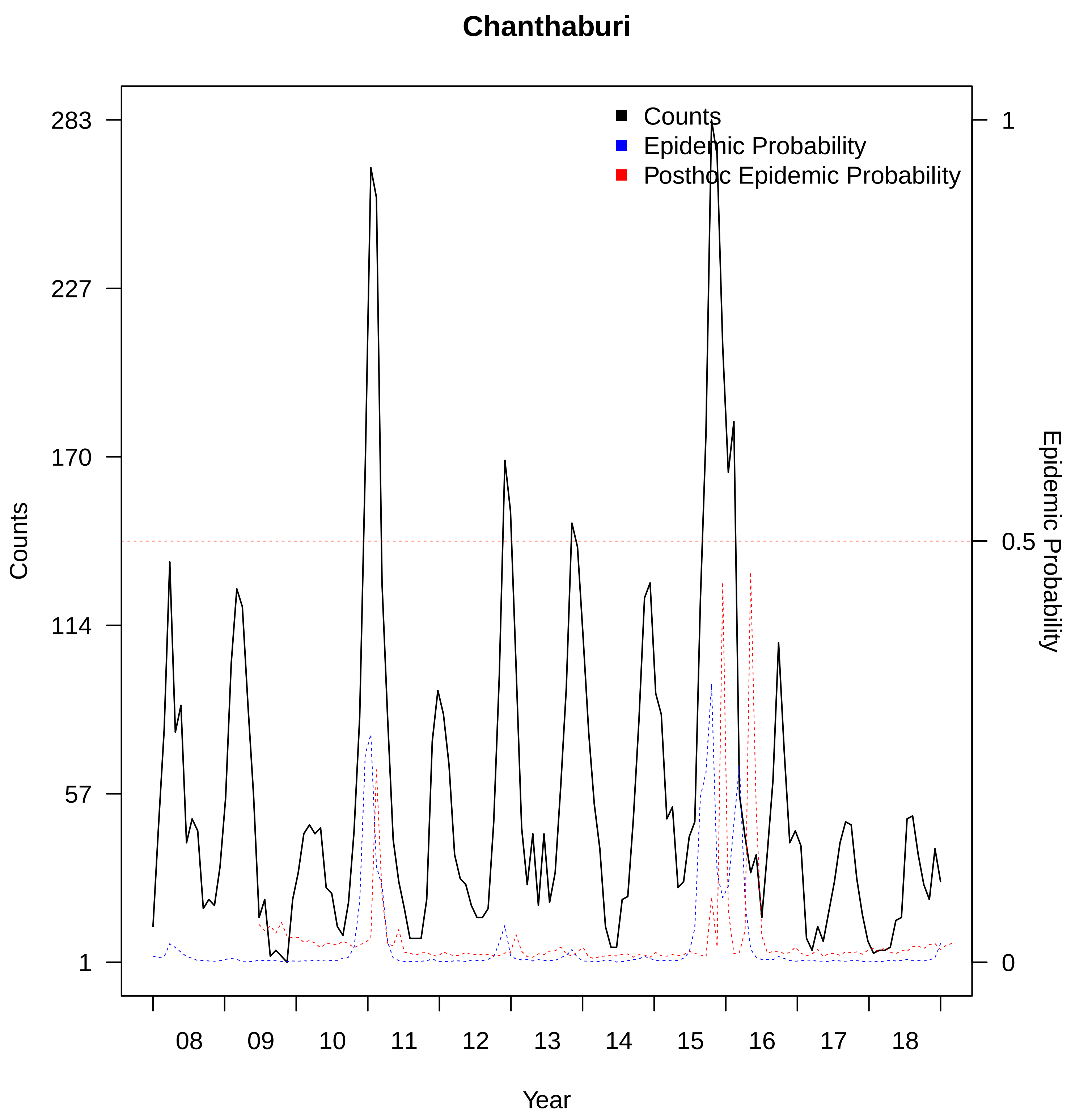

Chanthaburi
Counts
Epidemic Probability
Posthoc Epidemic Probability
283
227
170
114
57
1
Counts
1
0.5
0
Epidemic Probability
08
09
10
11
12
13
14
15
16
17
18
Year

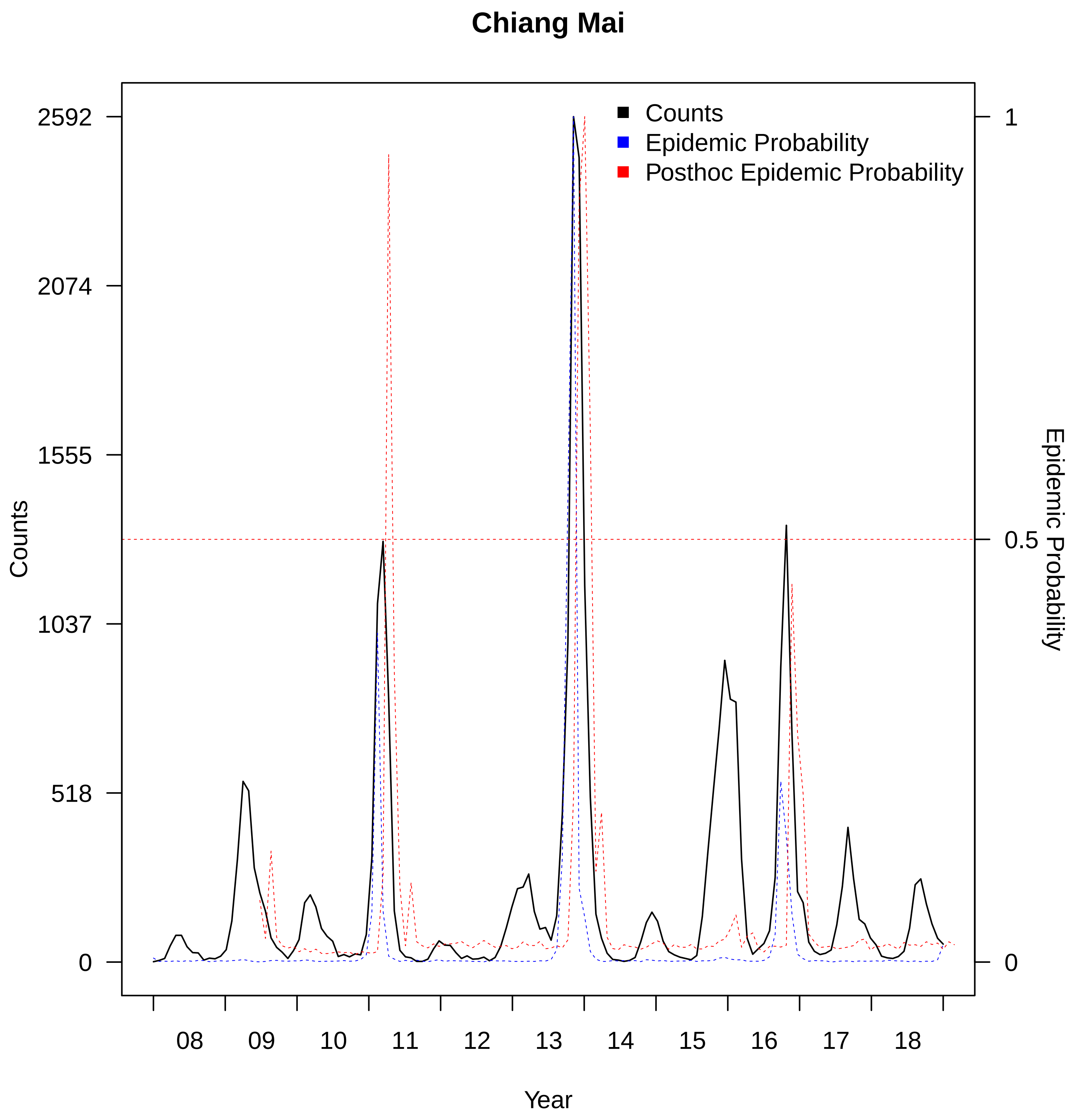

Chiang Mai
Counts
Epidemic Probability
Posthoc Epidemic Probability
2592
2074
1555
1037
518
0
Counts
1
0.5
0
Epidemic Probability
08
09
10
11
12
13
14
15
16
17
18
Year

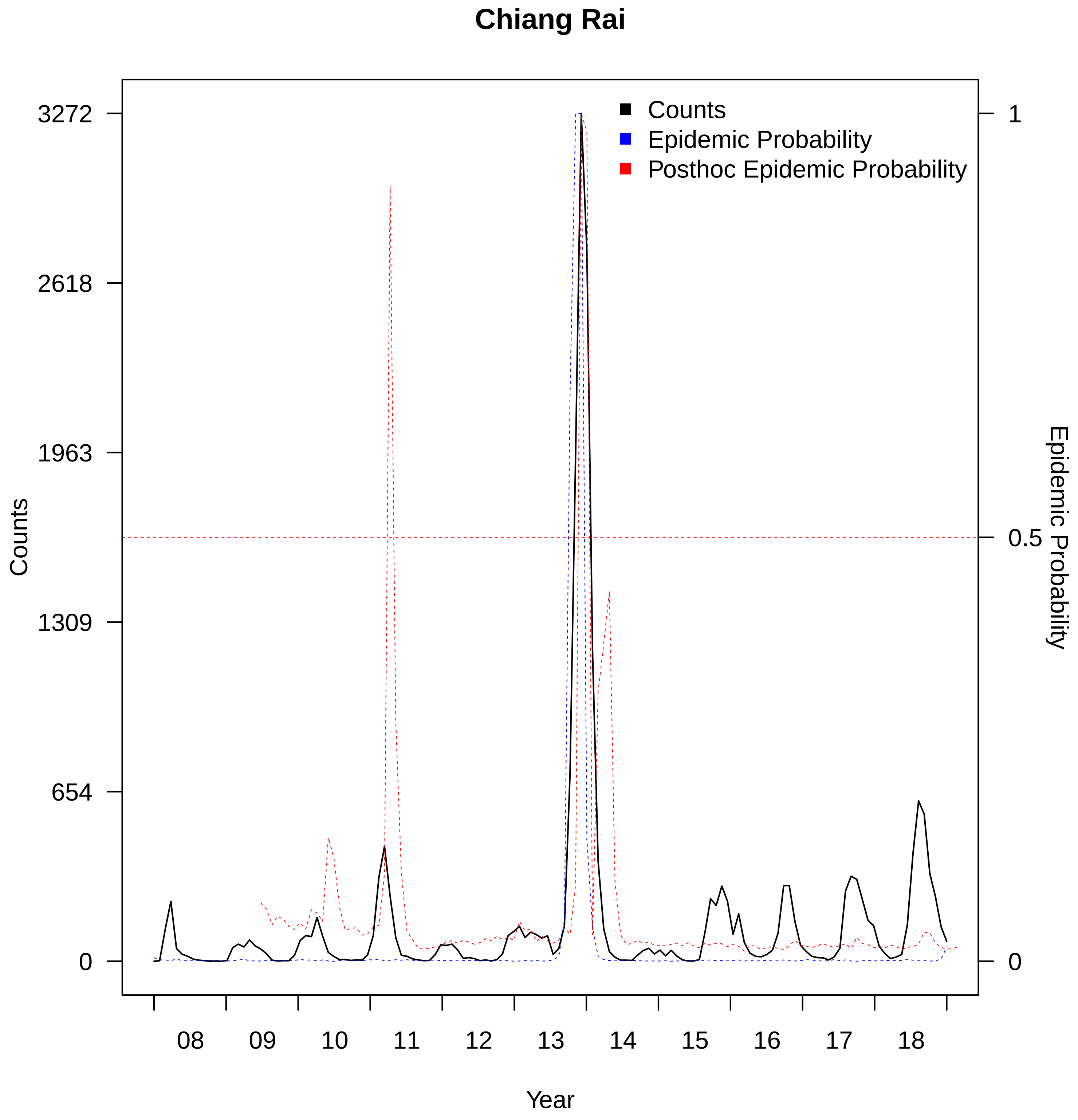
Chiang Rai
Counts
Epidemic Probability
Posthoc Epidemic Probability
3272
2618
1963
1309
654
0
1
0.5
0
Counts
Epidemic Probability
08
09
10
11
12
13
14
15
16
17
18
Year

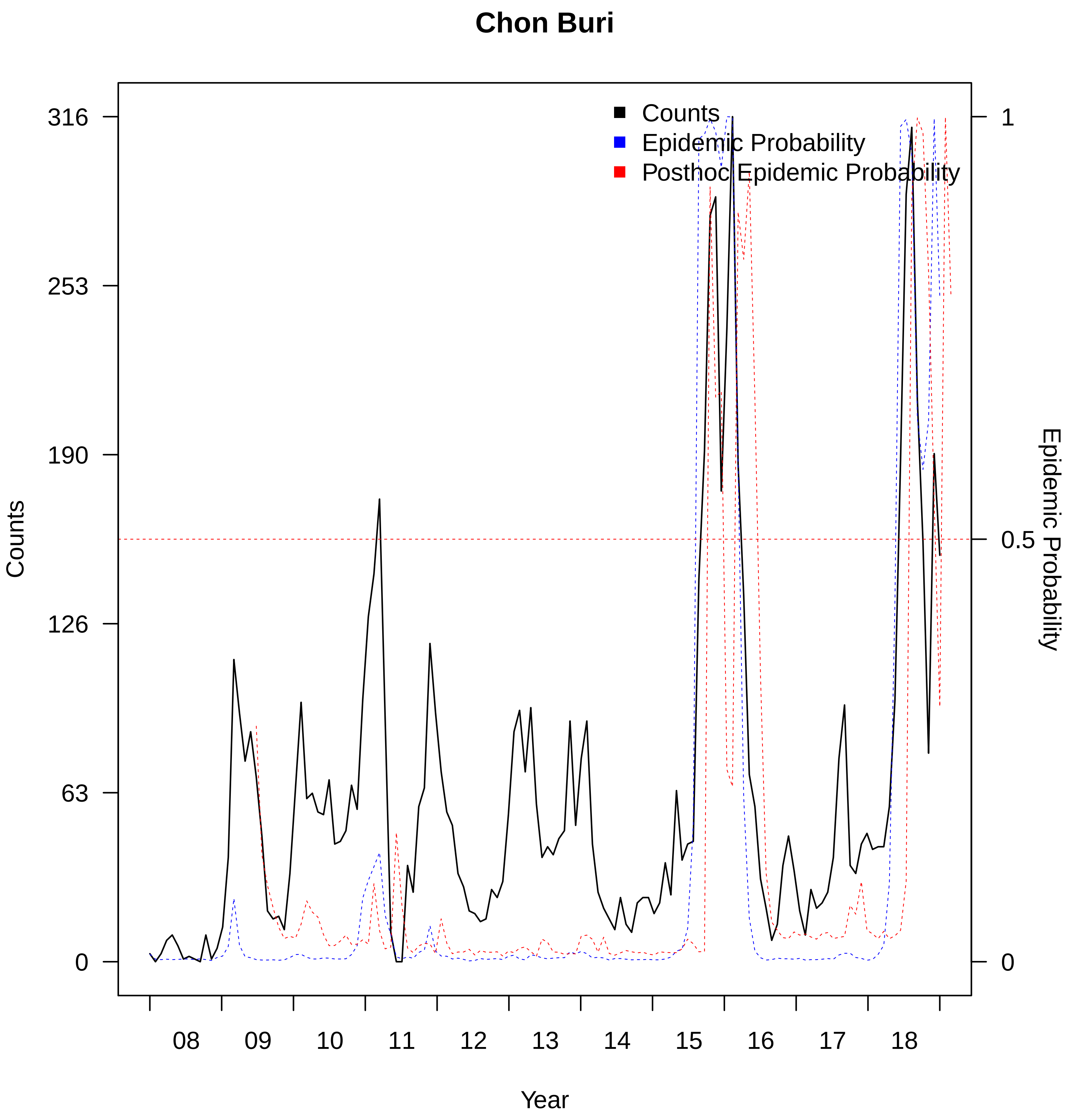
Chon Buri
Counts
Epidemic Probability
Posthoc Epidemic Probability
316
253
190
126
63
0
1
0.5
0
08
09
10
11
12
13
14
15
16
17
18
Year
Counts
Epidemic Probability

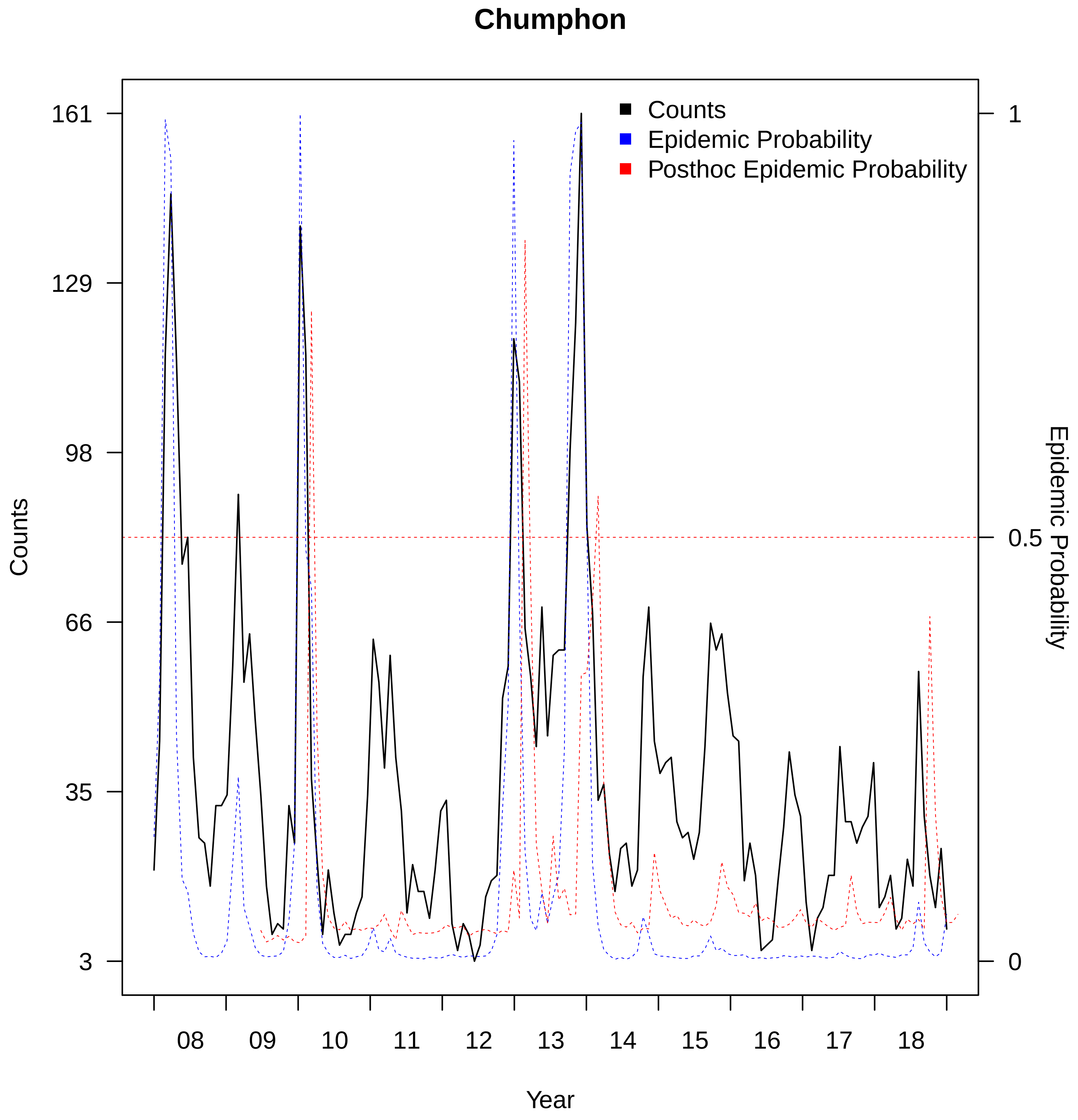

Chumphon
Counts
Epidemic Probability
Posthoc Epidemic Probability
161
129
98
66
35
3
1
0.5
0
Counts
Epidemic Probability
08
09
10
11
12
13
14
15
16
17
18
Year

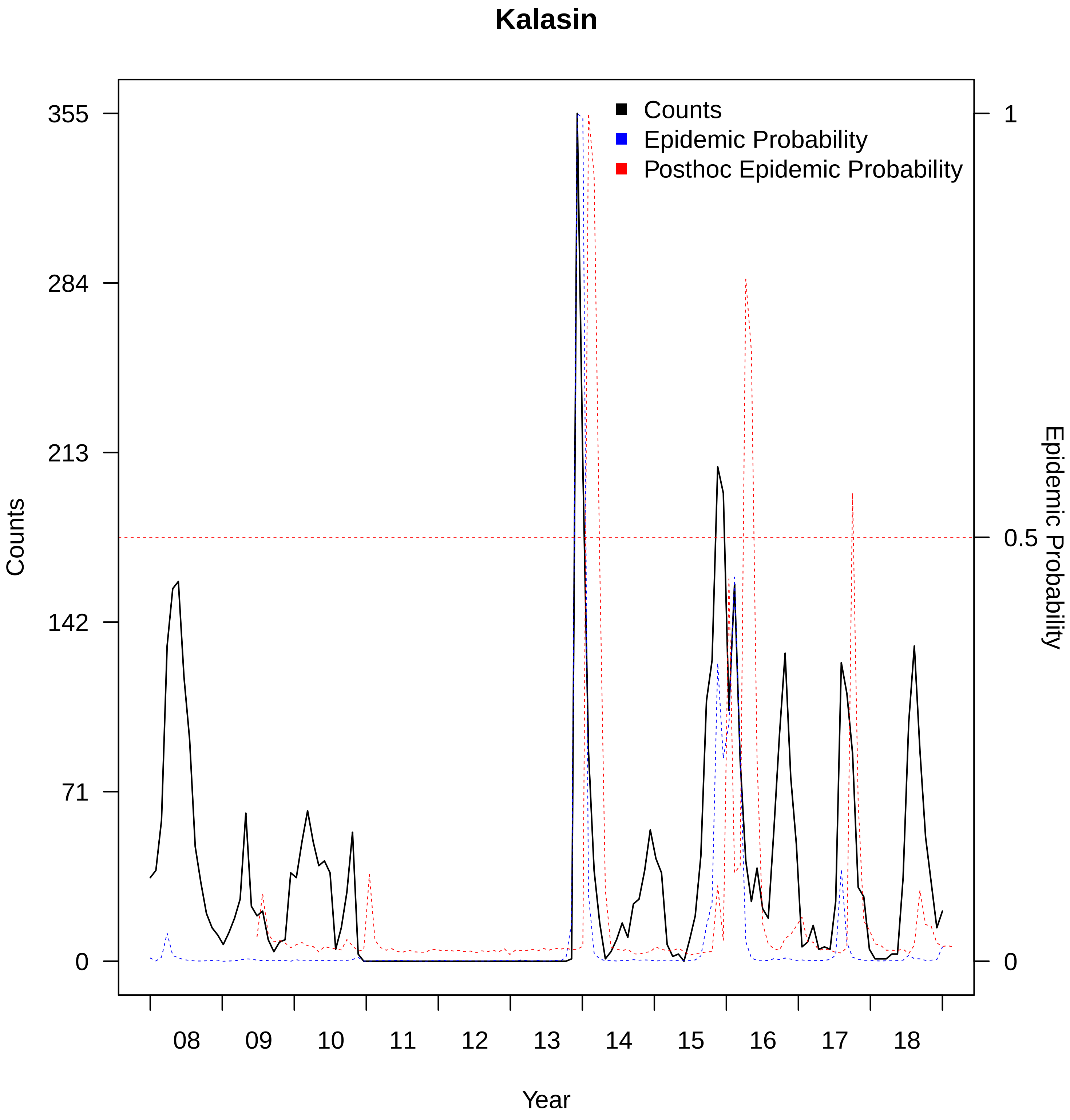

Kalasin
Counts
Epidemic Probability
Posthoc Epidemic Probability
355
284
213
142
71
0
1
0.5
0
08
09
10
11
12
13
14
15
16
17
18
Year
Counts
Epidemic Probability

Kamphaeng Phet

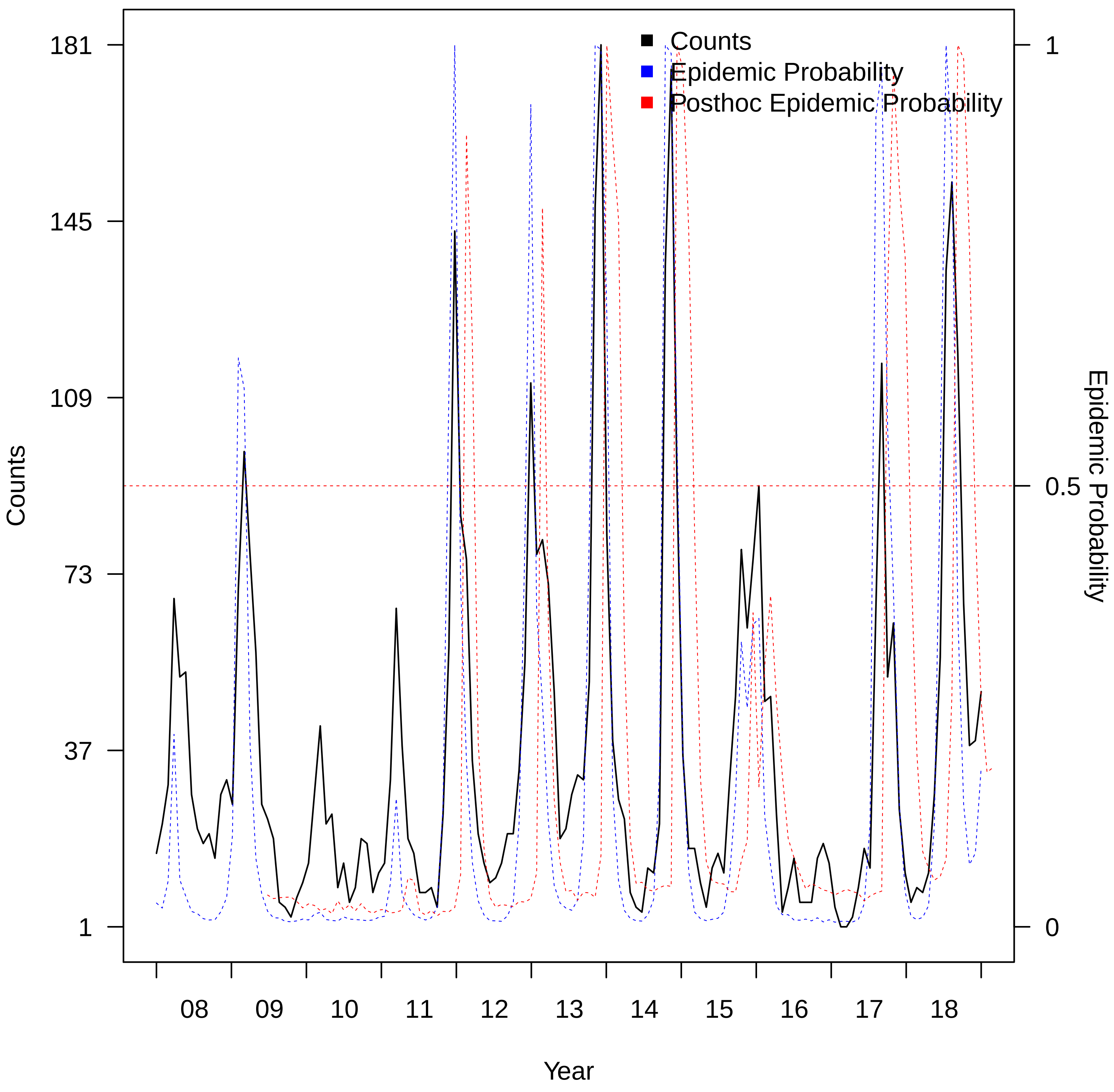

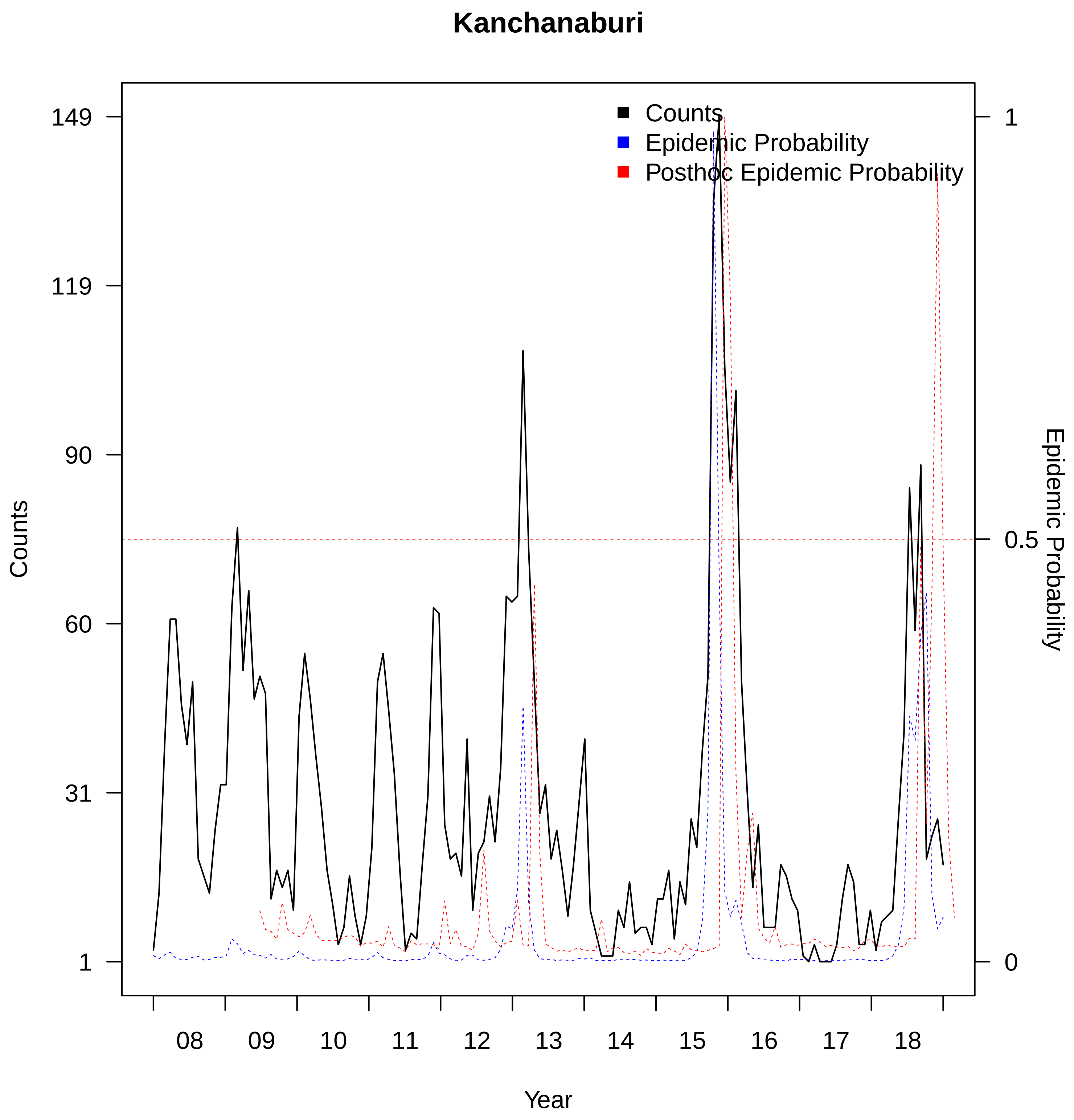

Kanchanaburi
Counts
Epidemic Probability
Posthoc Epidemic Probability
149
119
90
60
31
1
1
0.5
0
Counts
Epidemic Probability
08
09
10
11
12
13
14
15
16
17
18
Year

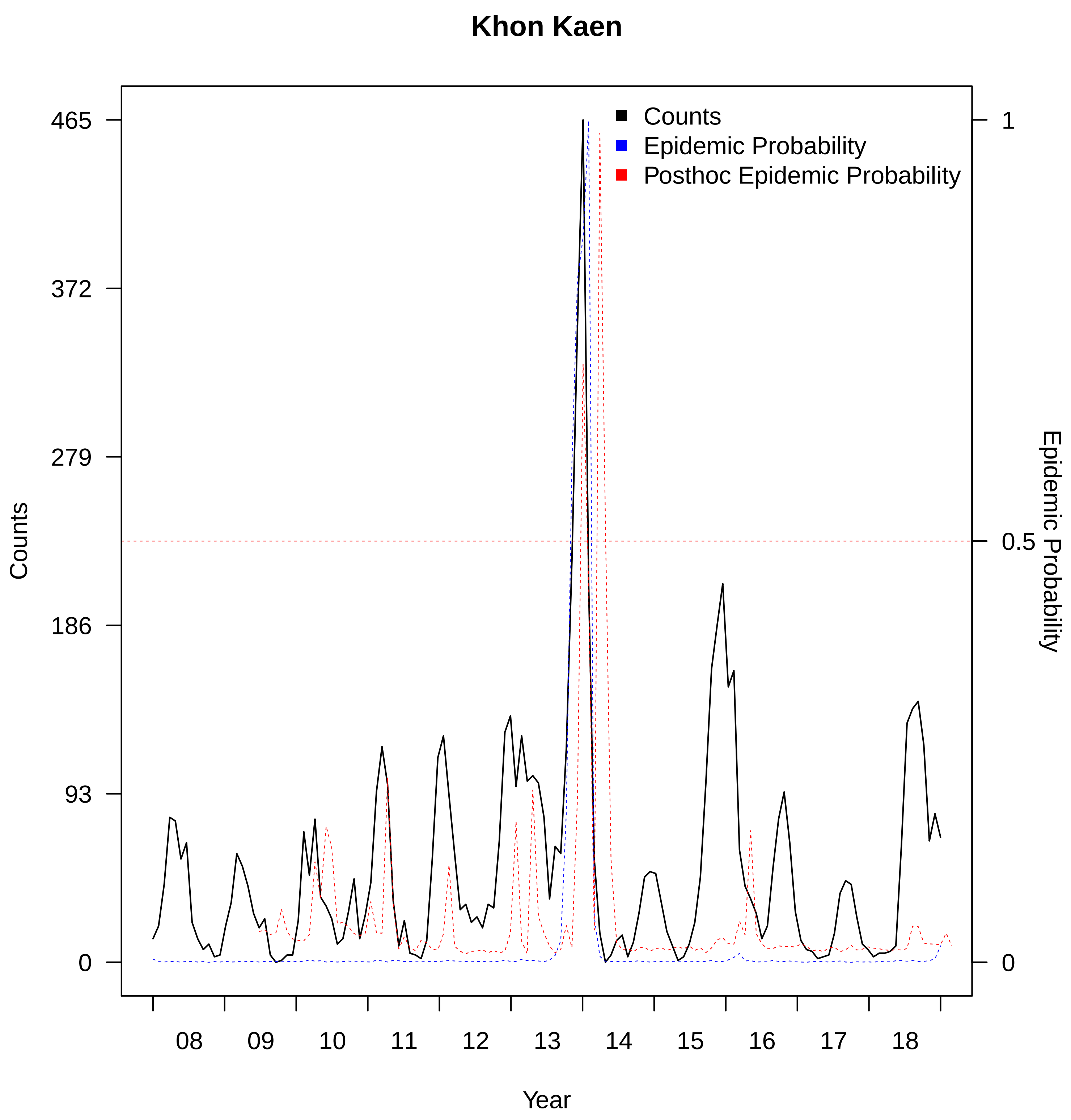

Khon Kaen
Counts
Epidemic Probability
Posthoc Epidemic Probability
465
372
279
186
93
0
1
0.5
0
Counts
Epidemic Probability
08
09
10
11
12
13
14
15
16
17
18
Year

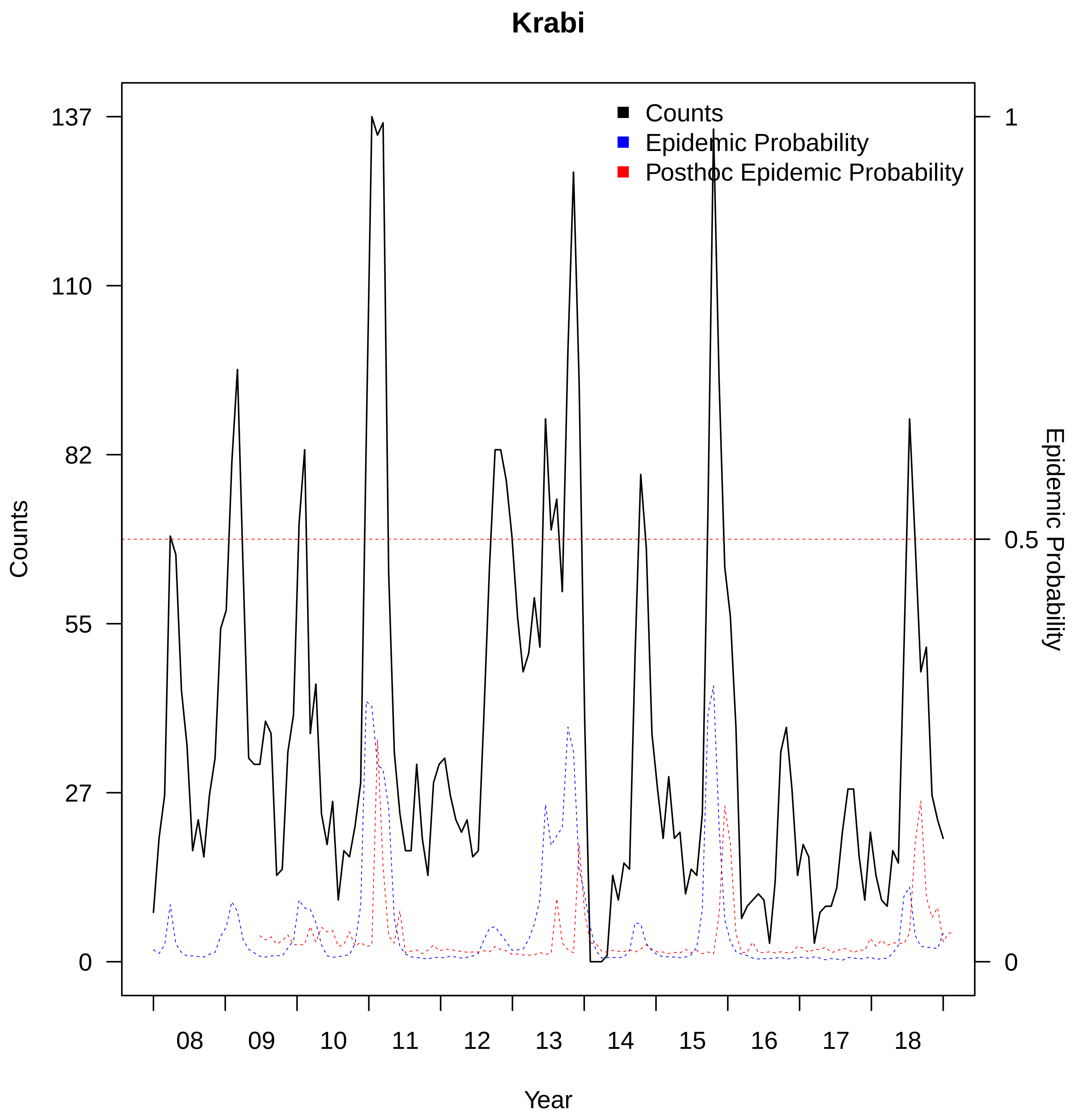

Krabi
Counts
Epidemic Probability
Posthoc Epidemic Probability
137
110
82
55
27
0
1
0.5
0
Counts
Epidemic Probability
08
09
10
11
12
13
14
15
16
17
18
Year

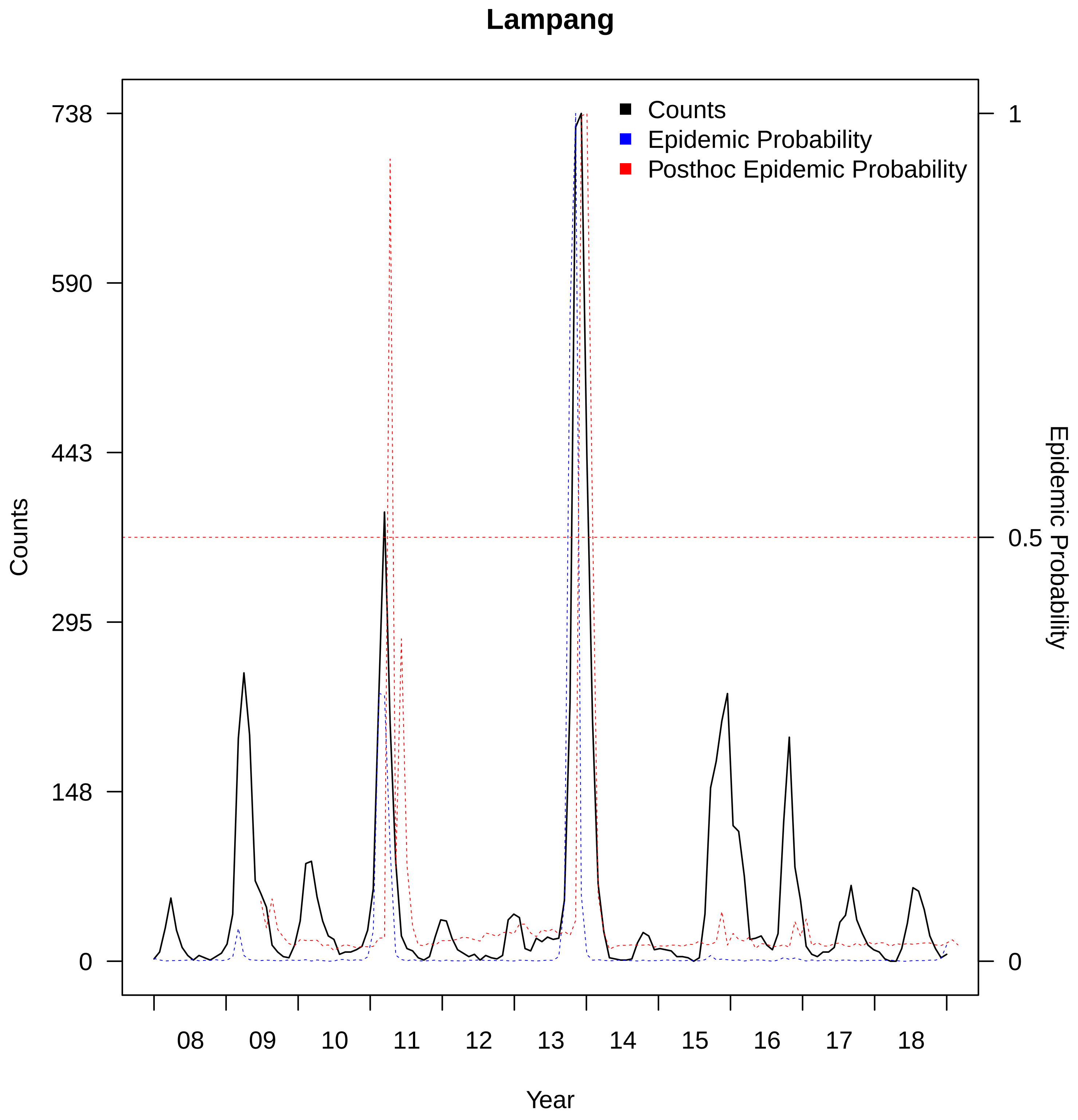
Lampang
Counts
Epidemic Probability
Posthoc Epidemic Probability
738
590
443
295
148
0
1
0.5
0
Counts
Epidemic Probability
08
09
10
11
12
13
14
15
16
17
18
Year

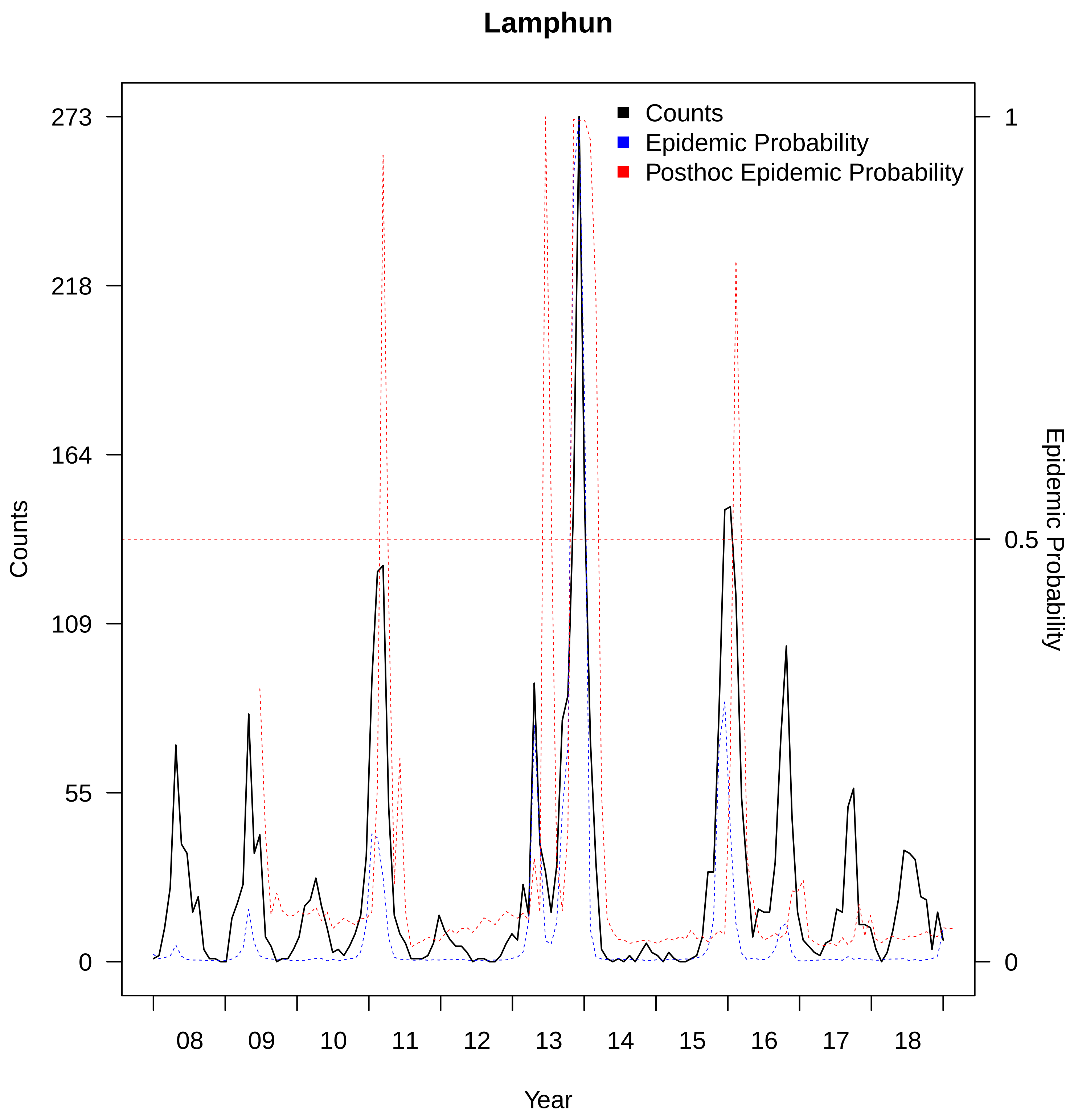

Lamphun
Counts
Epidemic Probability
Posthoc Epidemic Probability
273
218
164
109
55
0
1
0.5
0
Counts
Epidemic Probability
08
09
10
11
12
13
14
15
16
17
18
Year

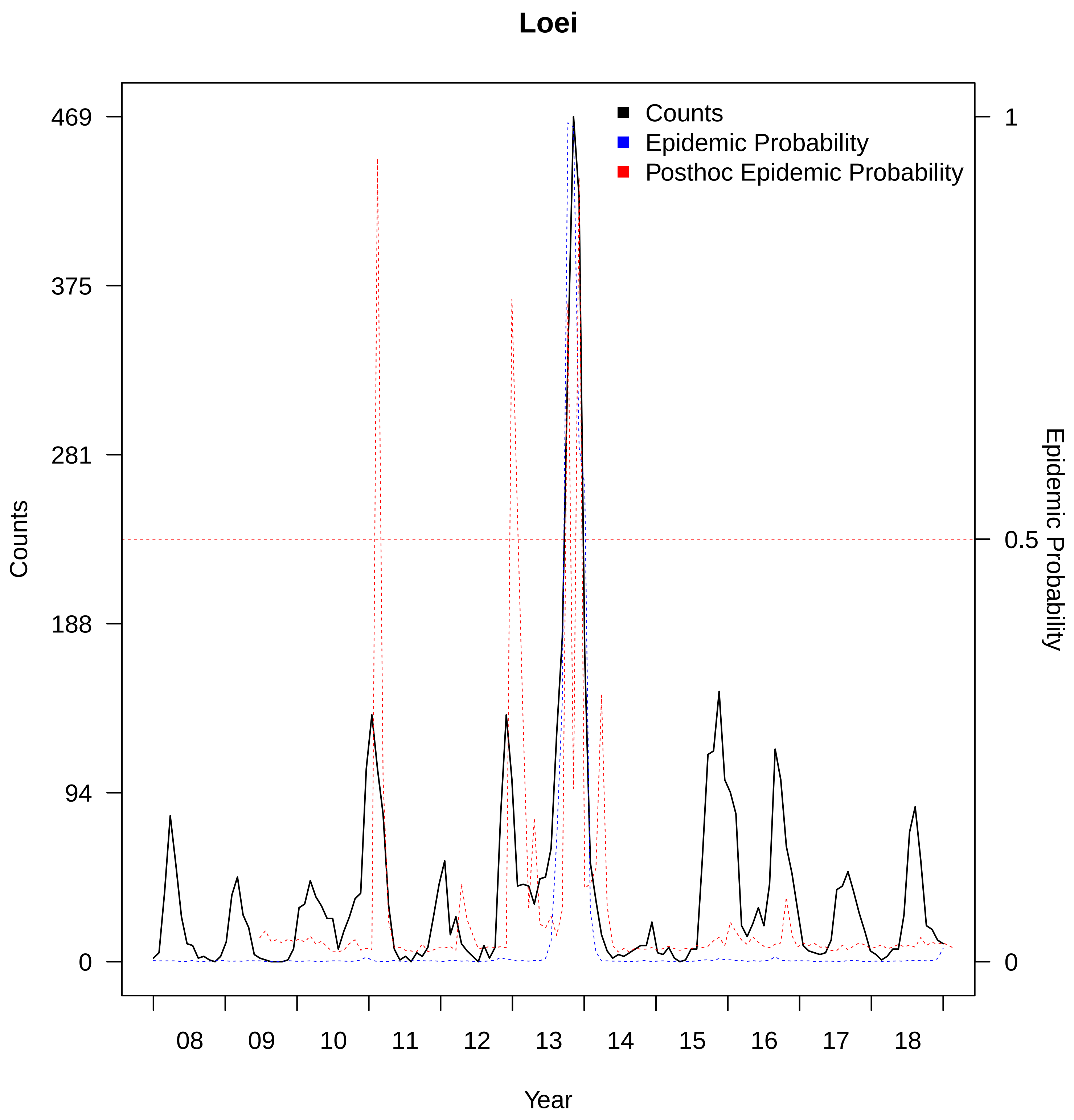

Loei
Counts
Epidemic Probability
Posthoc Epidemic Probability
469
375
281
188
94
0
Counts
1
0.5
0
Epidemic Probability
08
09
10
11
12
13
14
15
16
17
18
Year

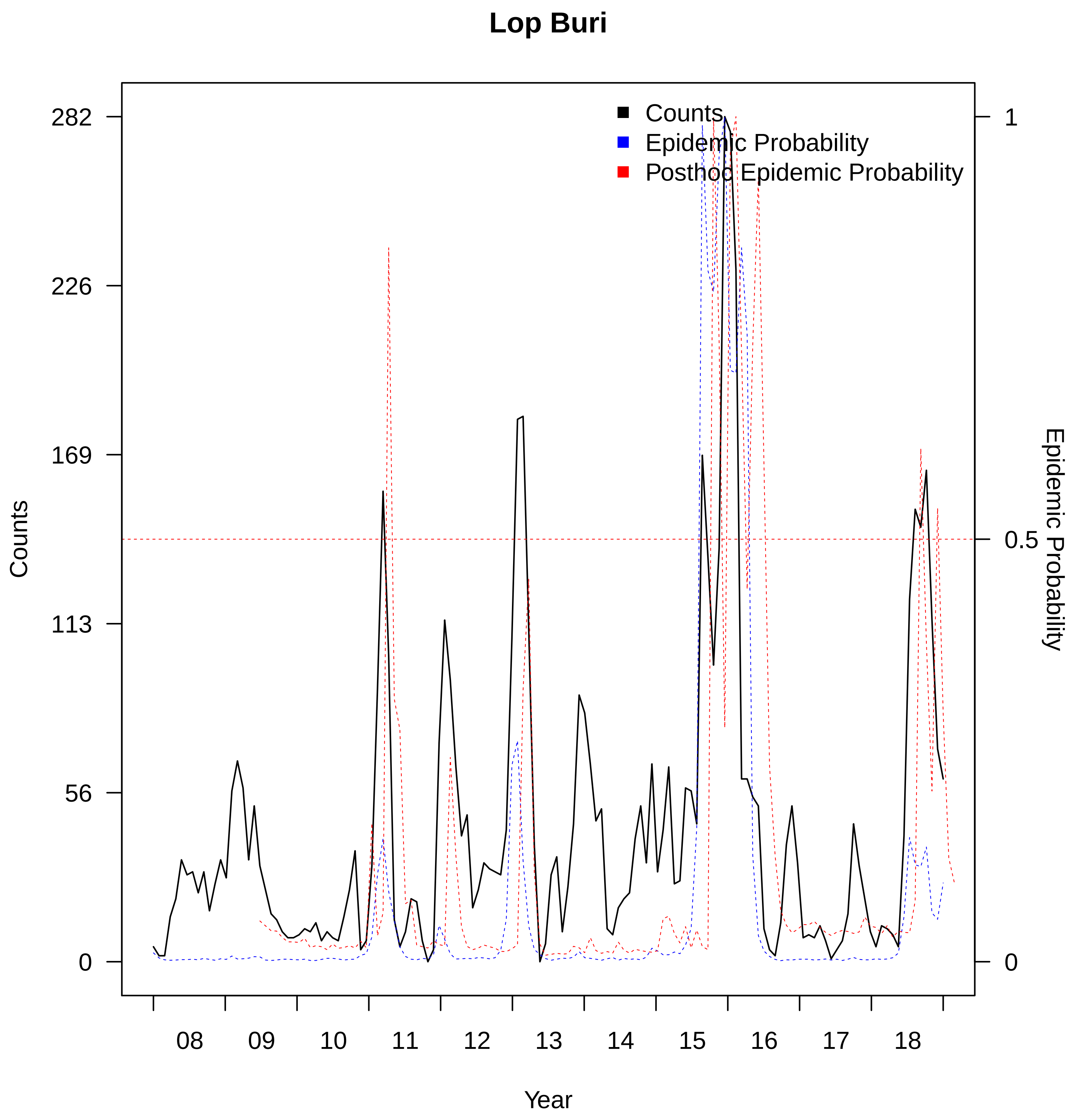

Lop Buri
Counts
Epidemic Probability
Posthoc Epidemic Probability
282
226
169
113
56
0
1
0.5
0
Counts
Epidemic Probability
08
09
10
11
12
13
14
15
16
17
18
Year

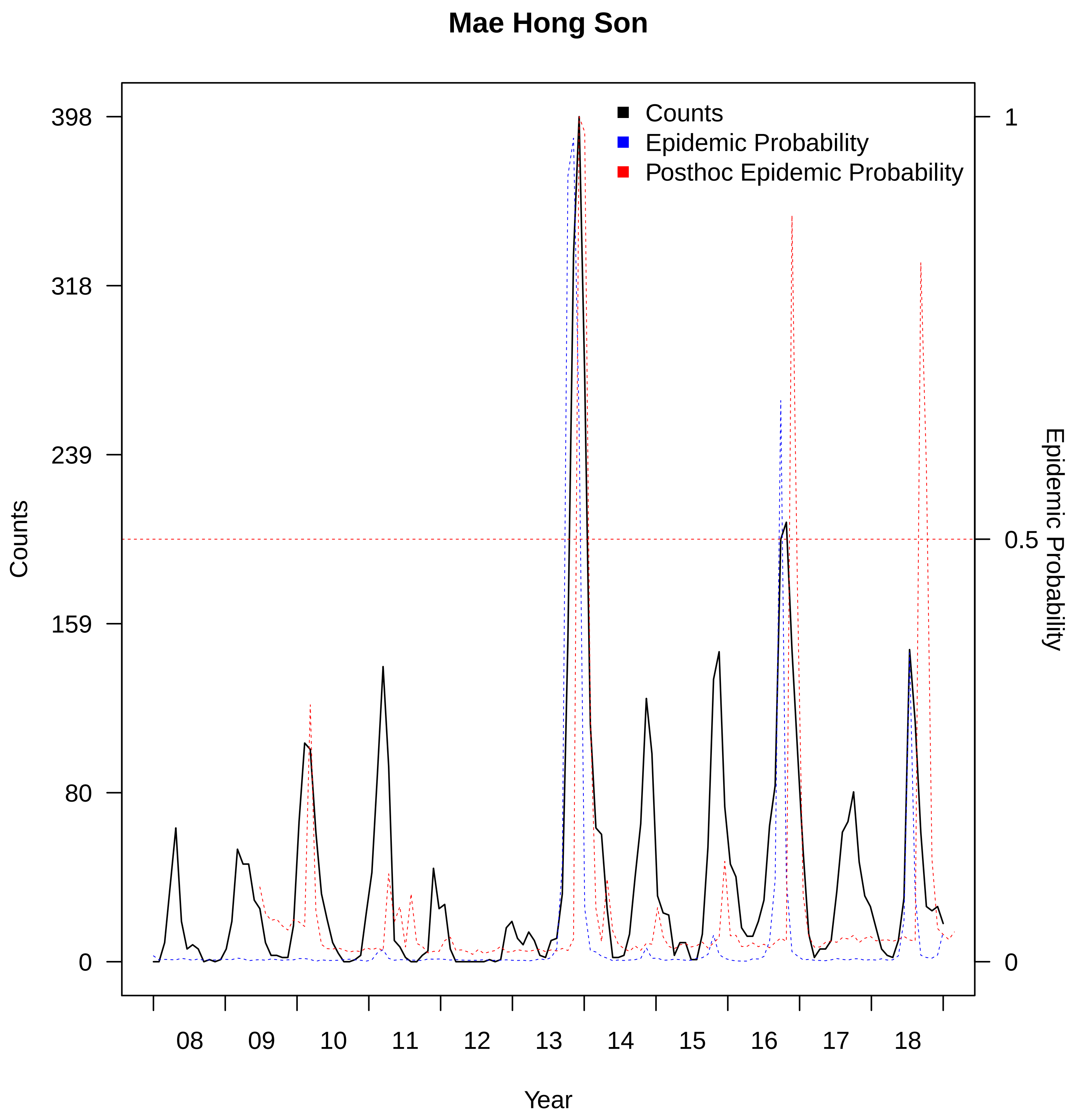

Mae Hong Son
Counts
Epidemic Probability
Posthoc Epidemic Probability
398
318
239
159
80
0
1
0.5
0
Counts
Epidemic Probability
08
09
10
11
12
13
14
15
16
17
18
Year

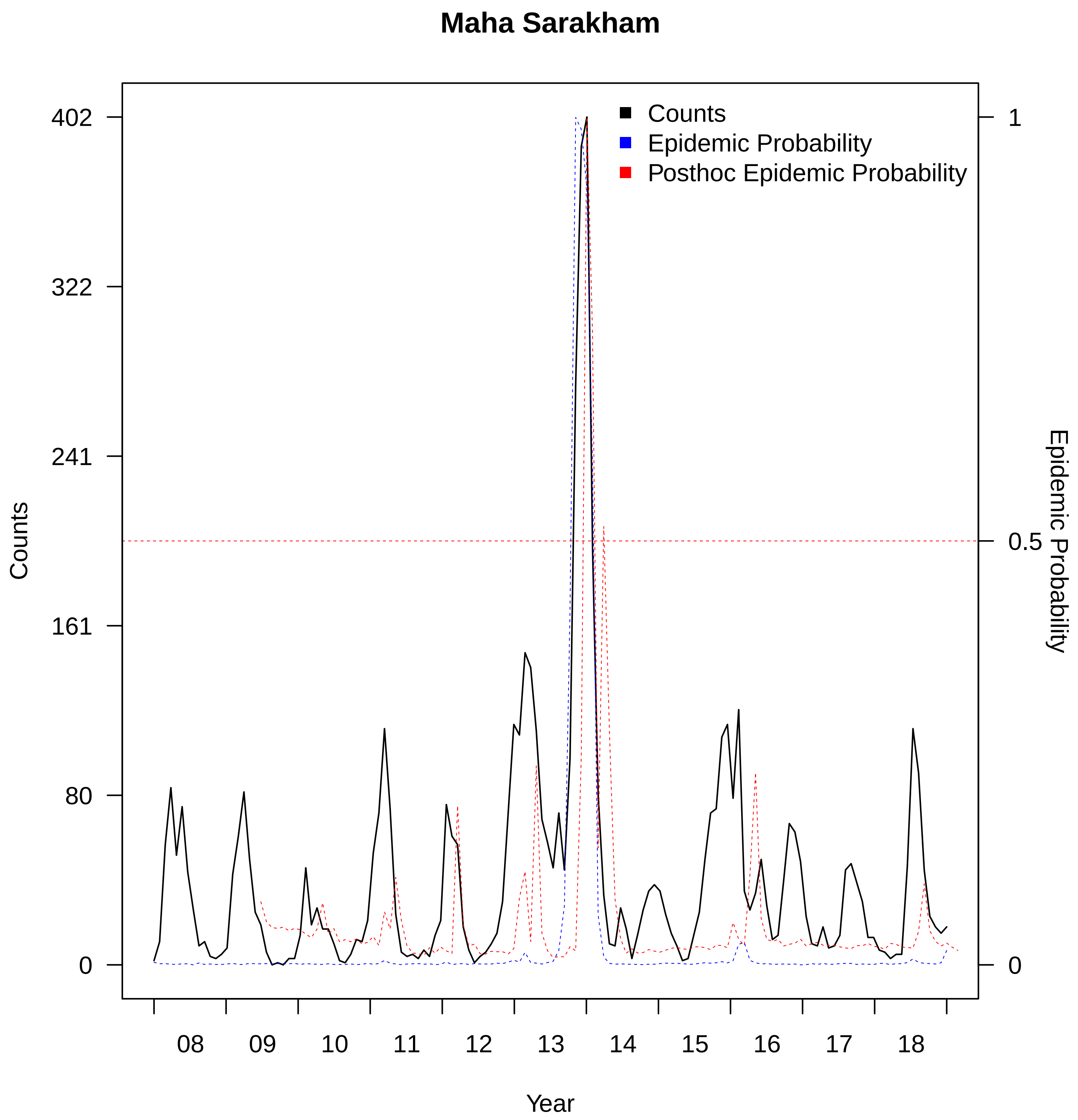

Maha Sarakham
Counts
Epidemic Probability
Posthoc Epidemic Probability
402
322
241
161
80
0
1
0.5
0
08
09
10
11
12
13
14
15
16
17
18
Year
Counts
Epidemic Probability

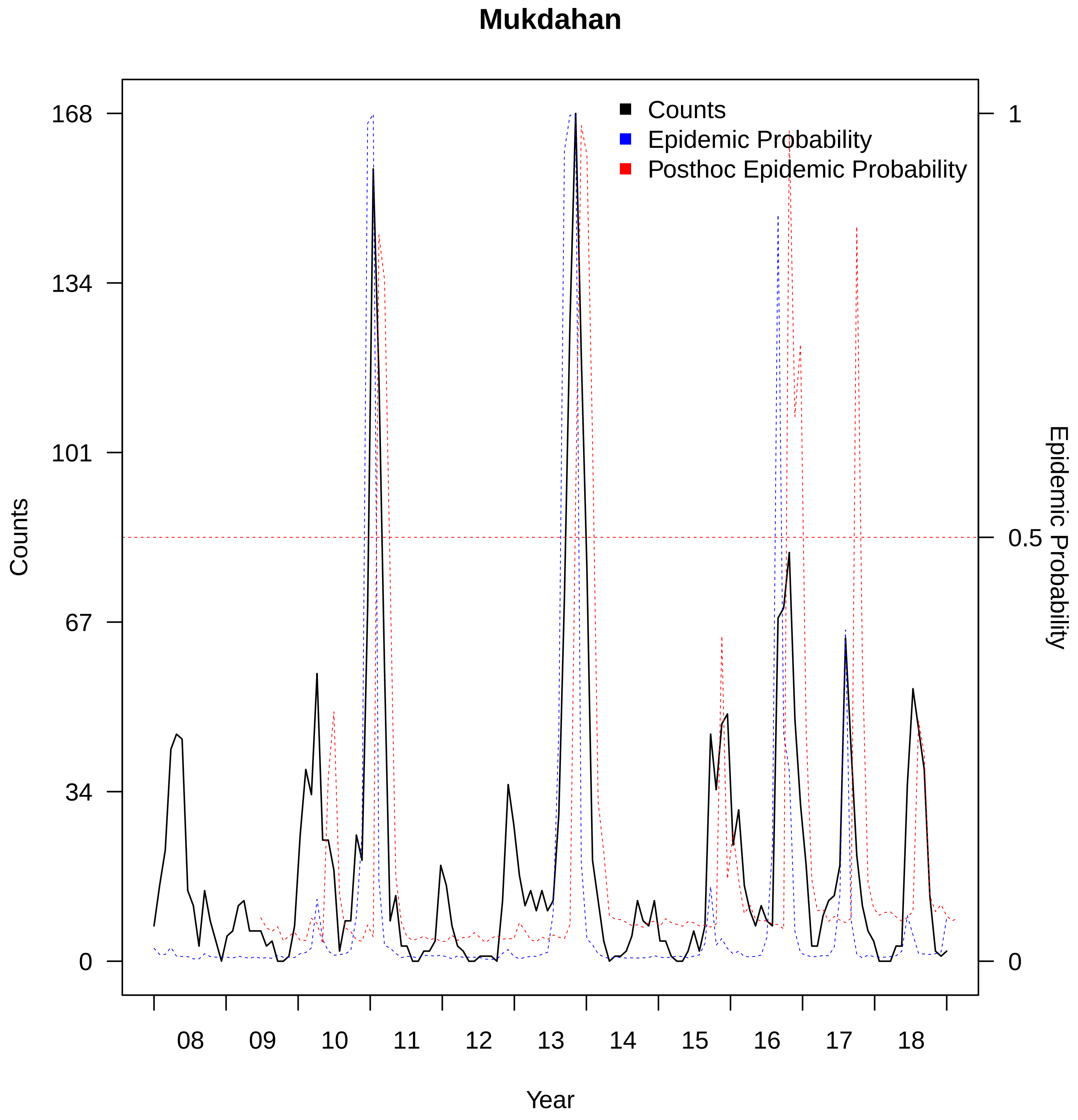
Mukdahan
Counts
Epidemic Probability
Posthoc Epidemic Probability
168
134
101
67
34
0
Counts
1
0.5
0
Epidemic Probability
08
09
10
11
12
13
14
15
16
17
18
Year

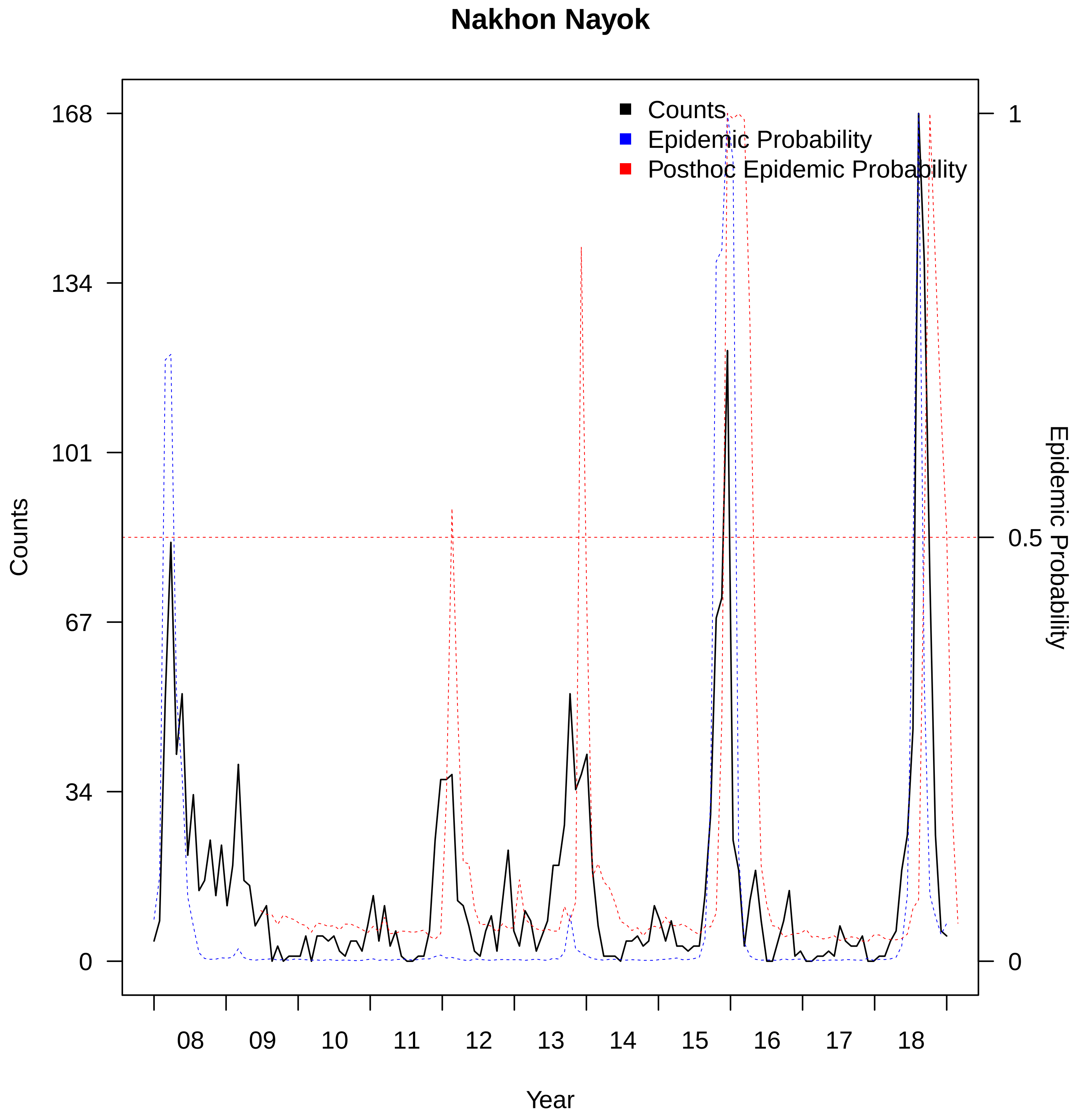
Nakhon Nayok
Counts
Epidemic Probability
Posthoc Epidemic Probability
168
134
101
67
34
0
1
0.5
0
Counts
Epidemic Probability
08
09
10
11
12
13
14
15
16
17
18
Year

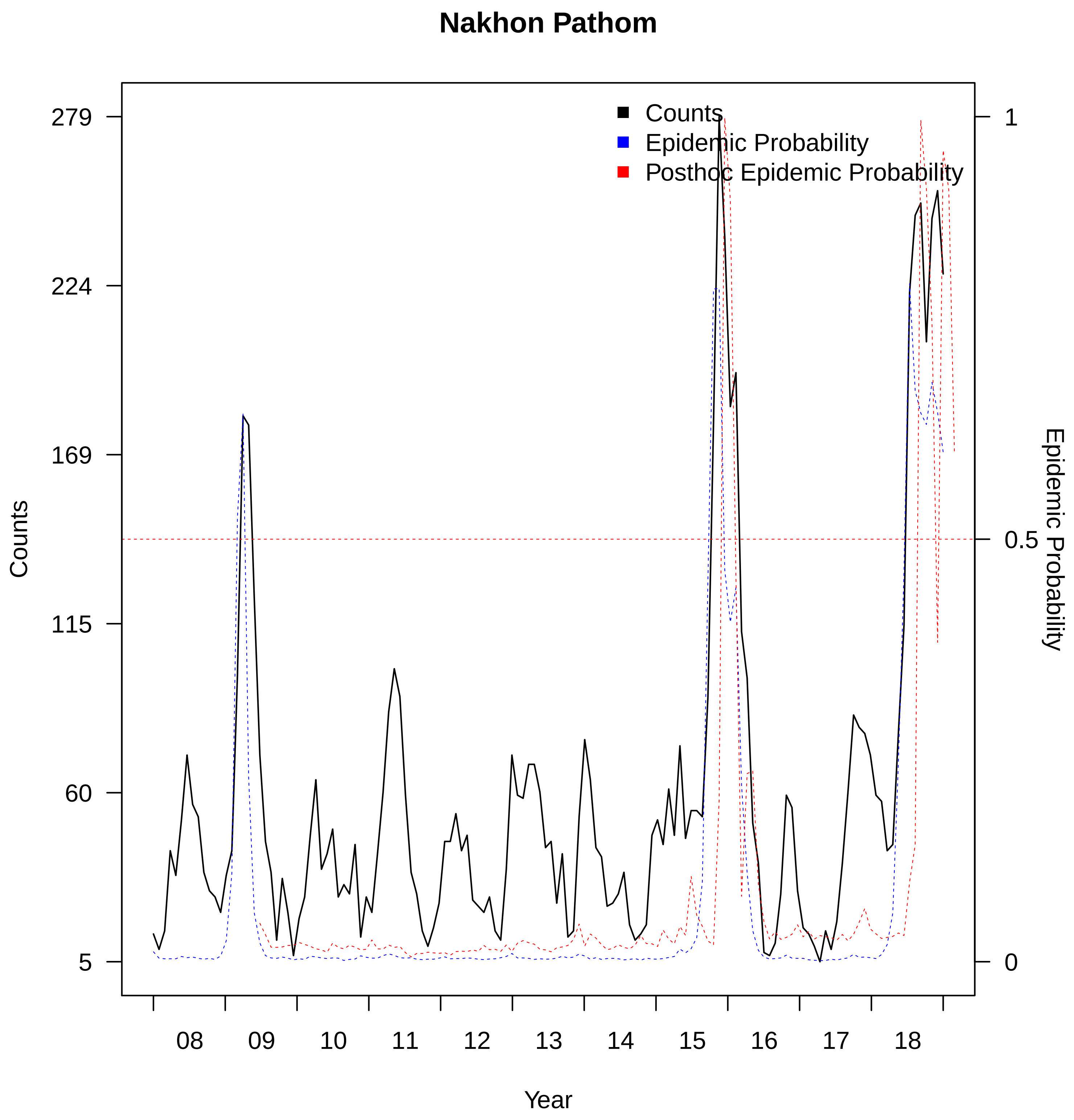

Nakhon Pathom
Counts
Epidemic Probability
Posthoc Epidemic Probability
279
224
169
115
60
5
1
0.5
0
08
09
10
11
12
13
14
15
16
17
18
Year
Counts
Epidemic Probability

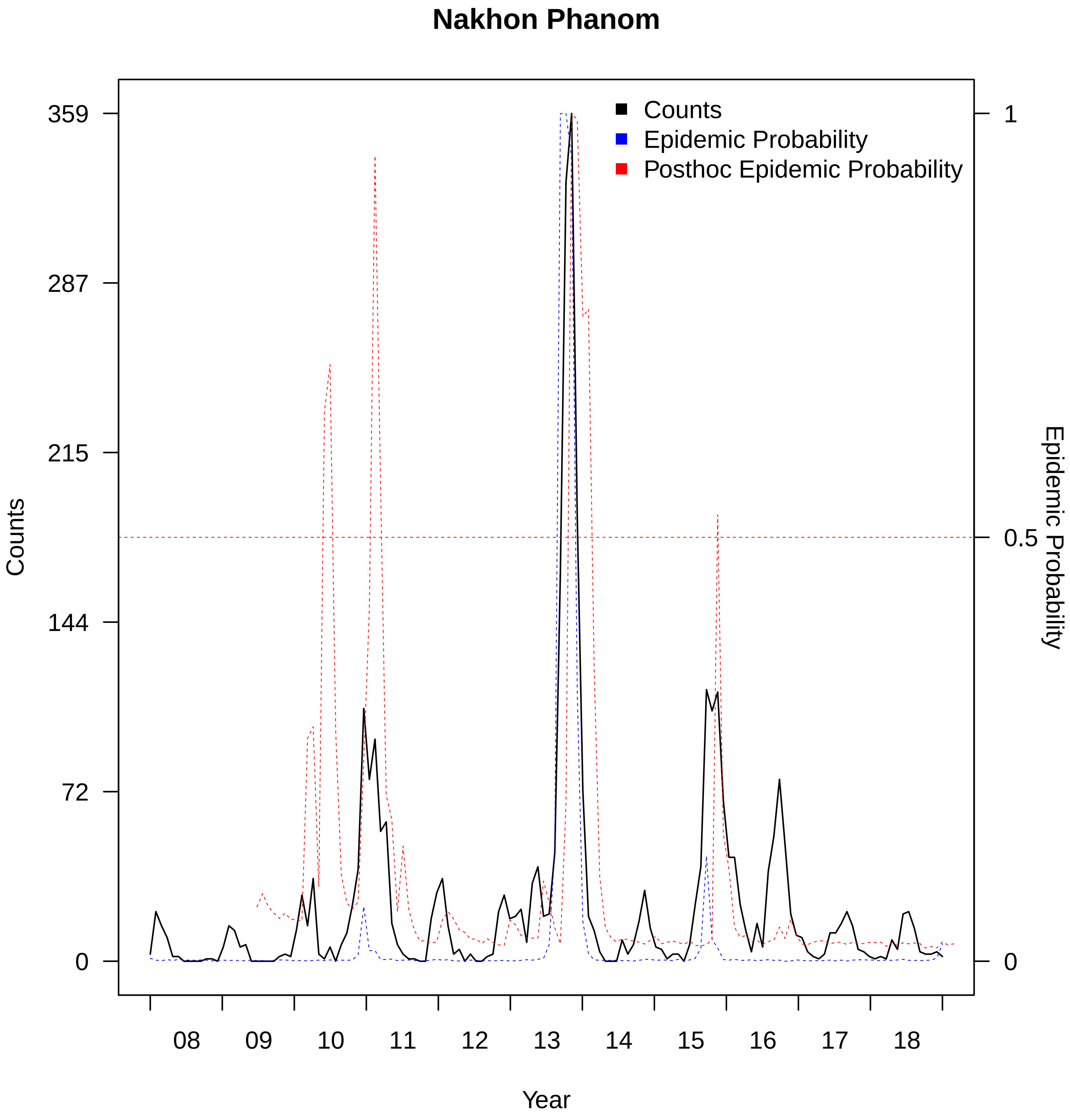
Nakhon Phanom
Counts
Epidemic Probability
Posthoc Epidemic Probability
359
287
215
144
72
0
1
0.5
0
Counts
Epidemic Probability
08
09
10
11
12
13
14
15
16
17
18
Year

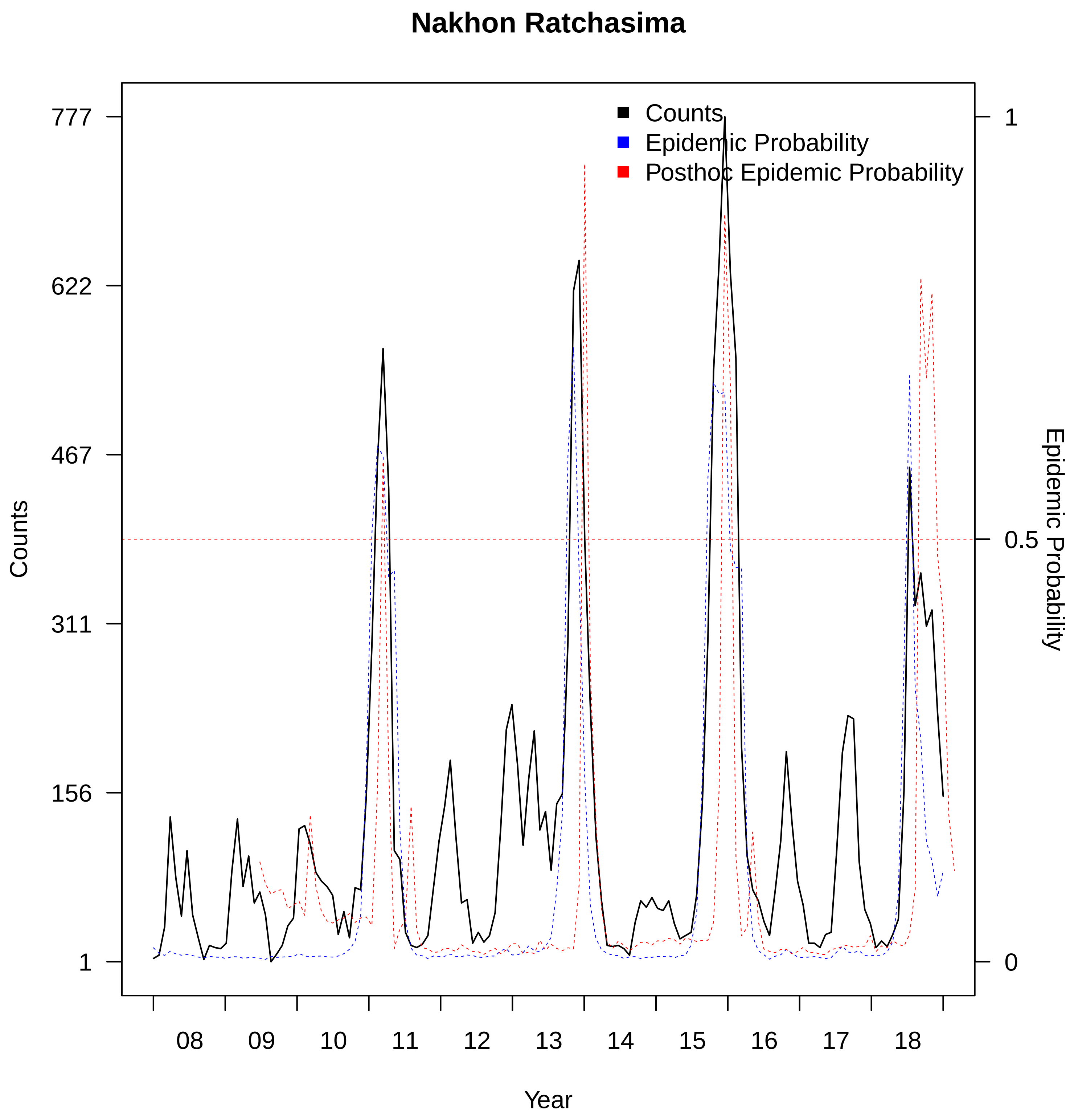
Nakhon Ratchasima
Counts
Epidemic Probability
Posthoc Epidemic Probability
777
622
467
311
156
1
1
0.5
0
Counts
Epidemic Probability
08
09
10
11
12
13
14
15
16
17
18
Year

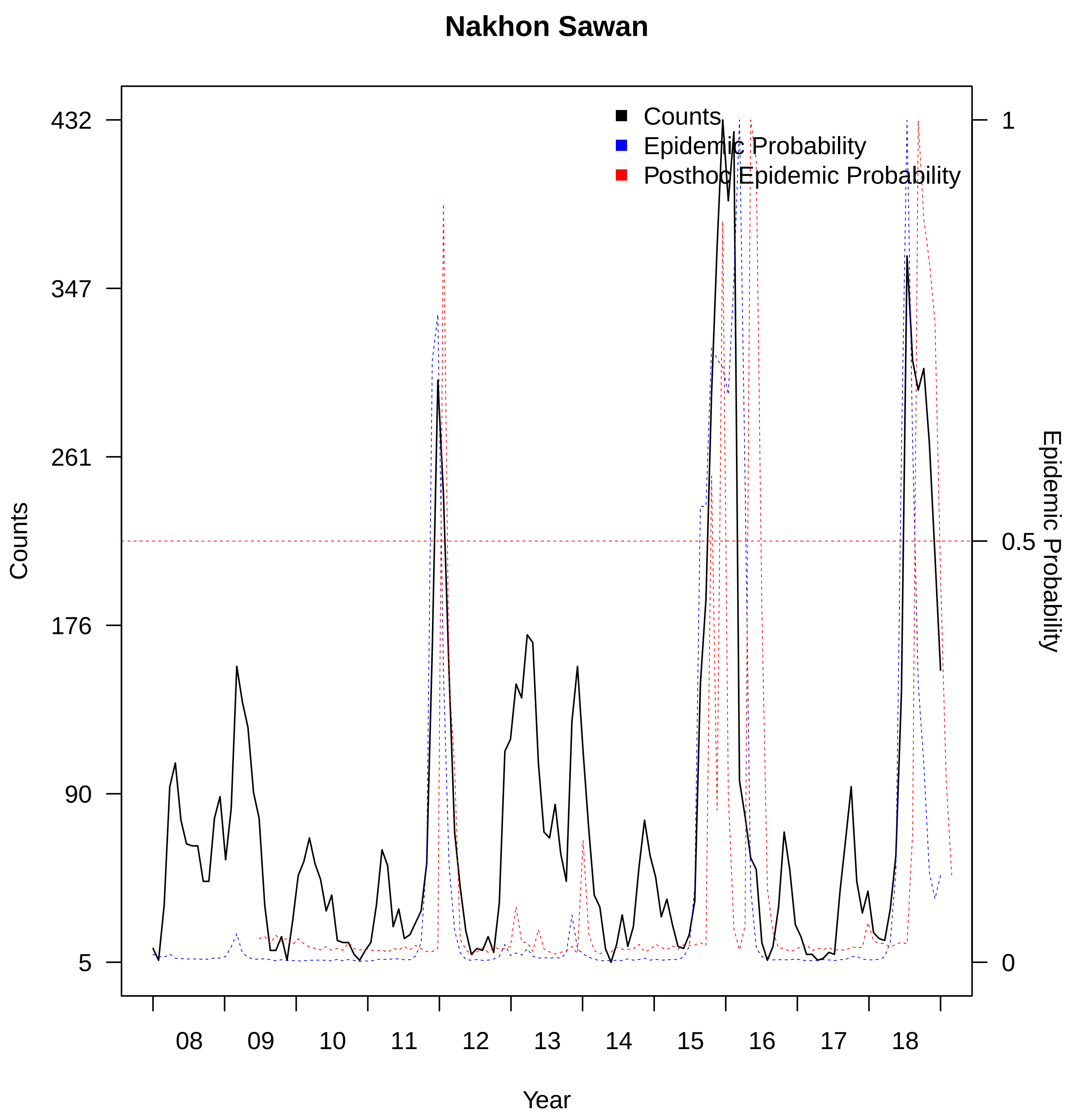

Nakhon Sawan
Counts
Epidemic Probability
Posthoc Epidemic Probability
432
347
261
176
90
5
1
0.5
0
Counts
Epidemic Probability
08
09
10
11
12
13
14
15
16
17
18
Year

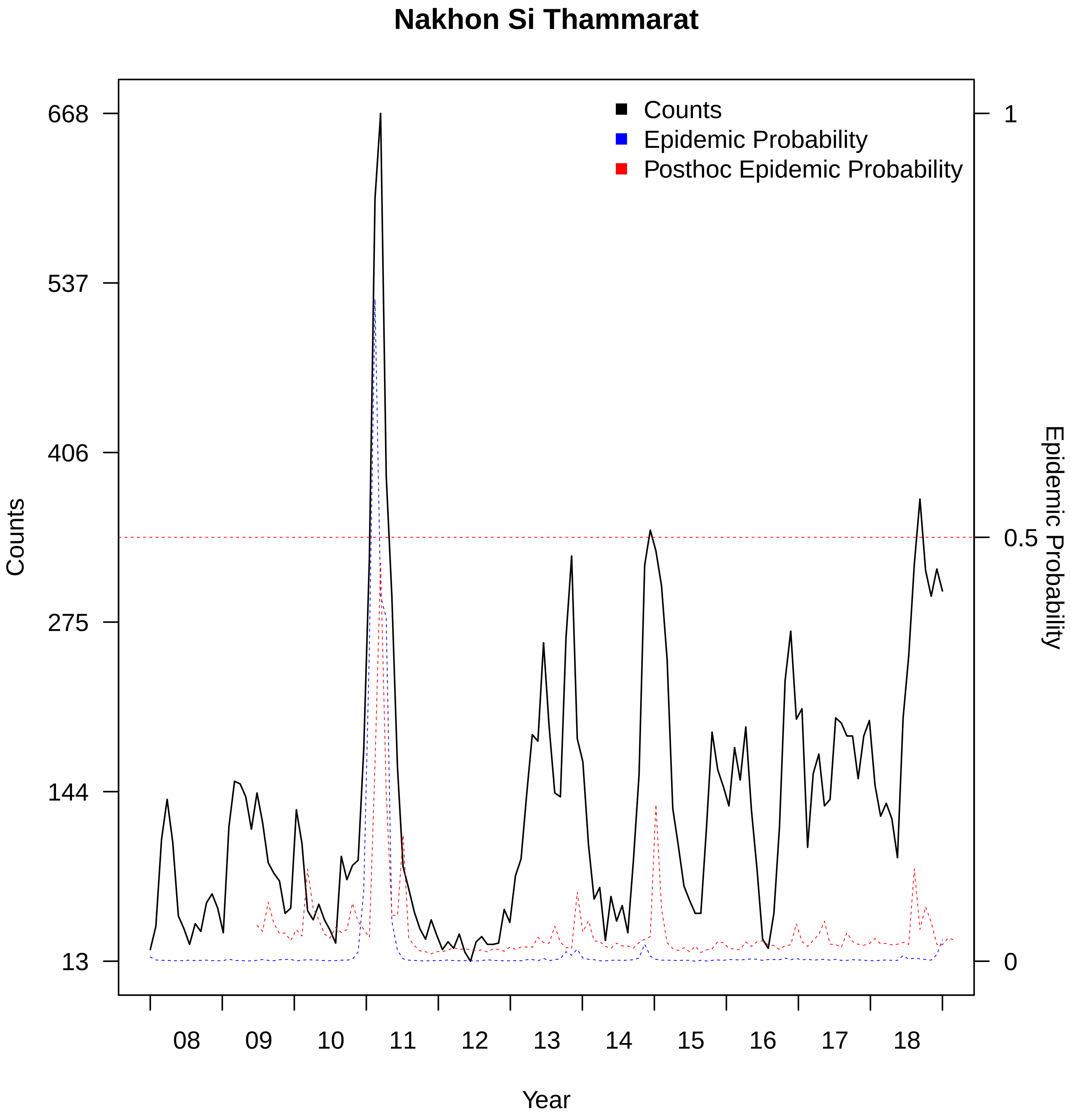
Nakhon Si Thammarat
Counts
Epidemic Probability
Posthoc Epidemic Probability
668
537
406
275
144
13
1
0.5
0
08
09
10
11
12
13
14
15
16
17
18
Year
Counts
Epidemic Probability

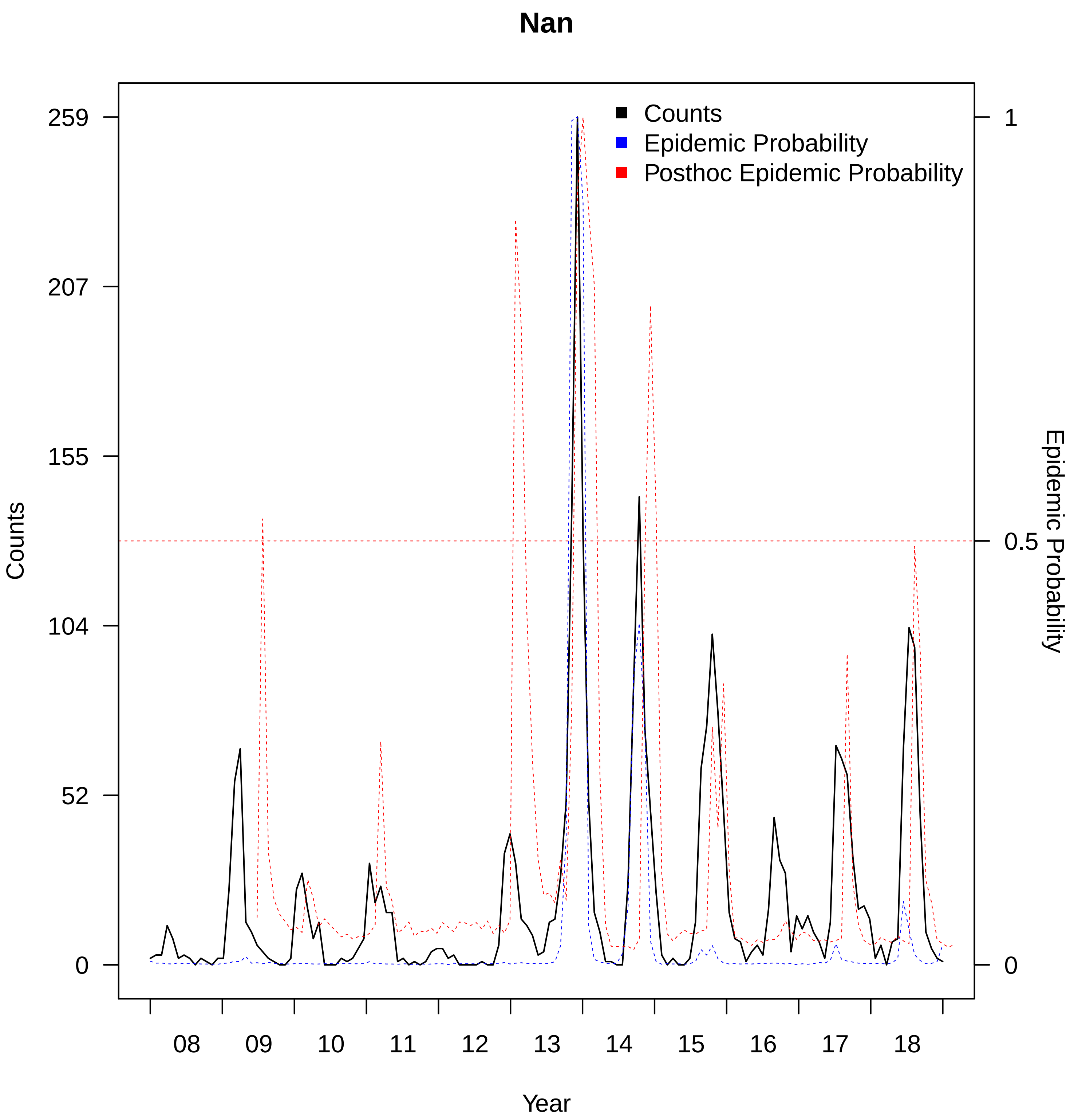
Nan
Counts
Epidemic Probability
Posthoc Epidemic Probability
259
207
155
104
52
0
1
0.5
0
Counts
Epidemic Probability
08
09
10
11
12
13
14
15
16
17
18
Year

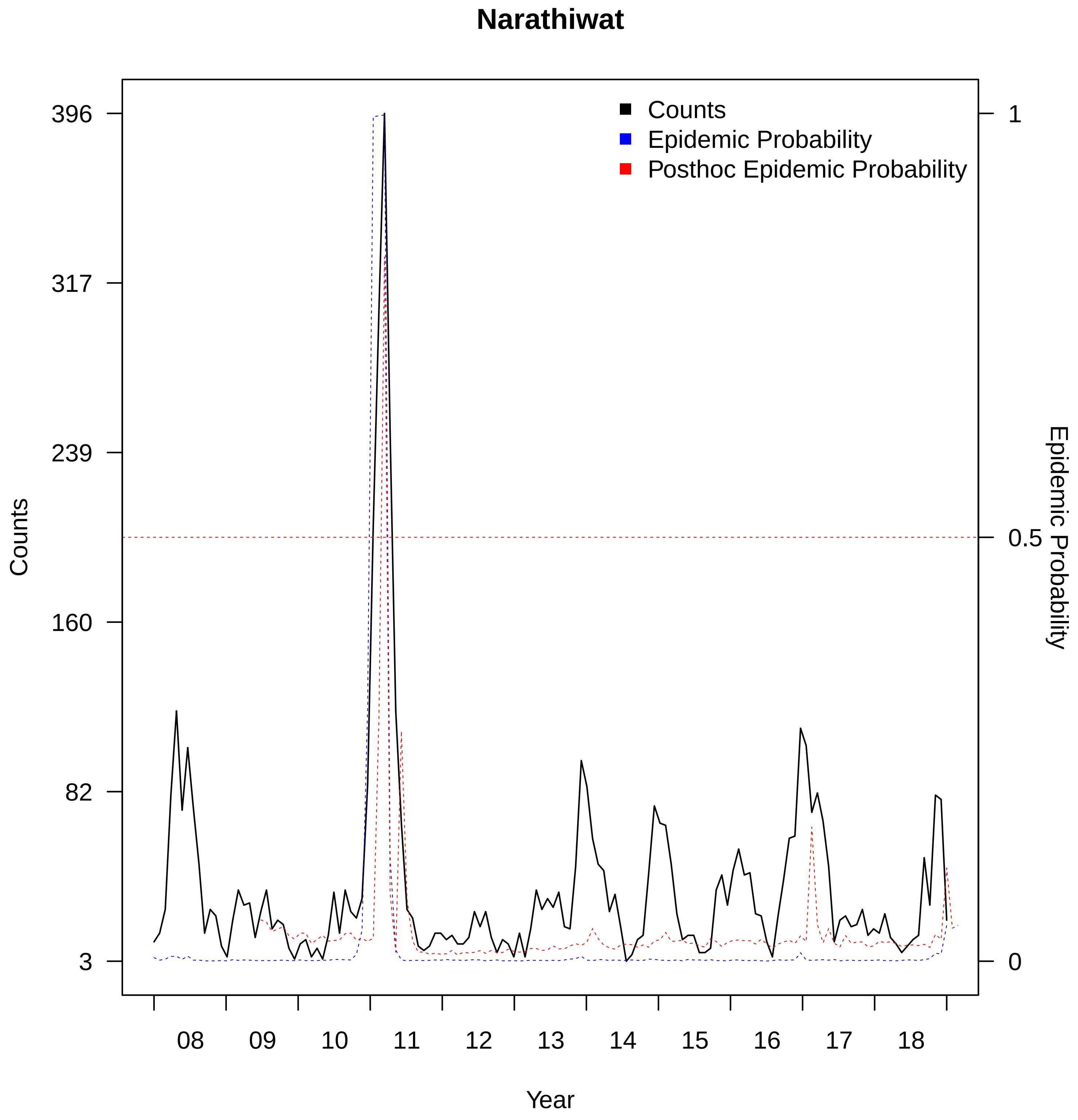

Narathiwat
Counts
Epidemic Probability
Posthoc Epidemic Probability
396
317
239
160
82
3
Counts
1
0.5
0
Epidemic Probability
08
09
10
11
12
13
14
15
16
17
18
Year

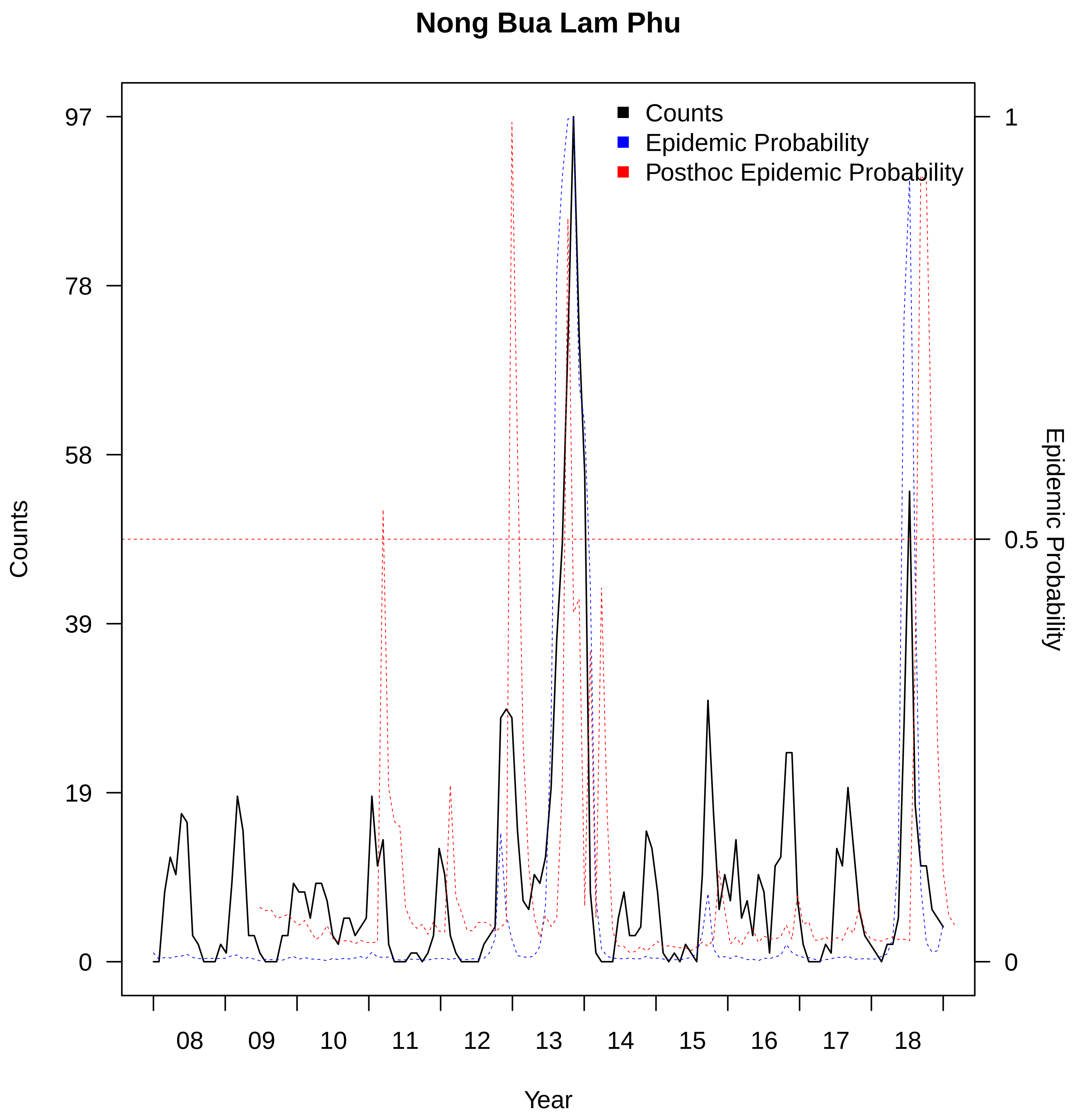
Nong Bua Lam Phu
Counts
Epidemic Probability
Posthoc Epidemic Probability
97
78
58
39
19
0
1
0.5
0
08
09
10
11
12
13
14
15
16
17
18
Year
Counts
Epidemic Probability

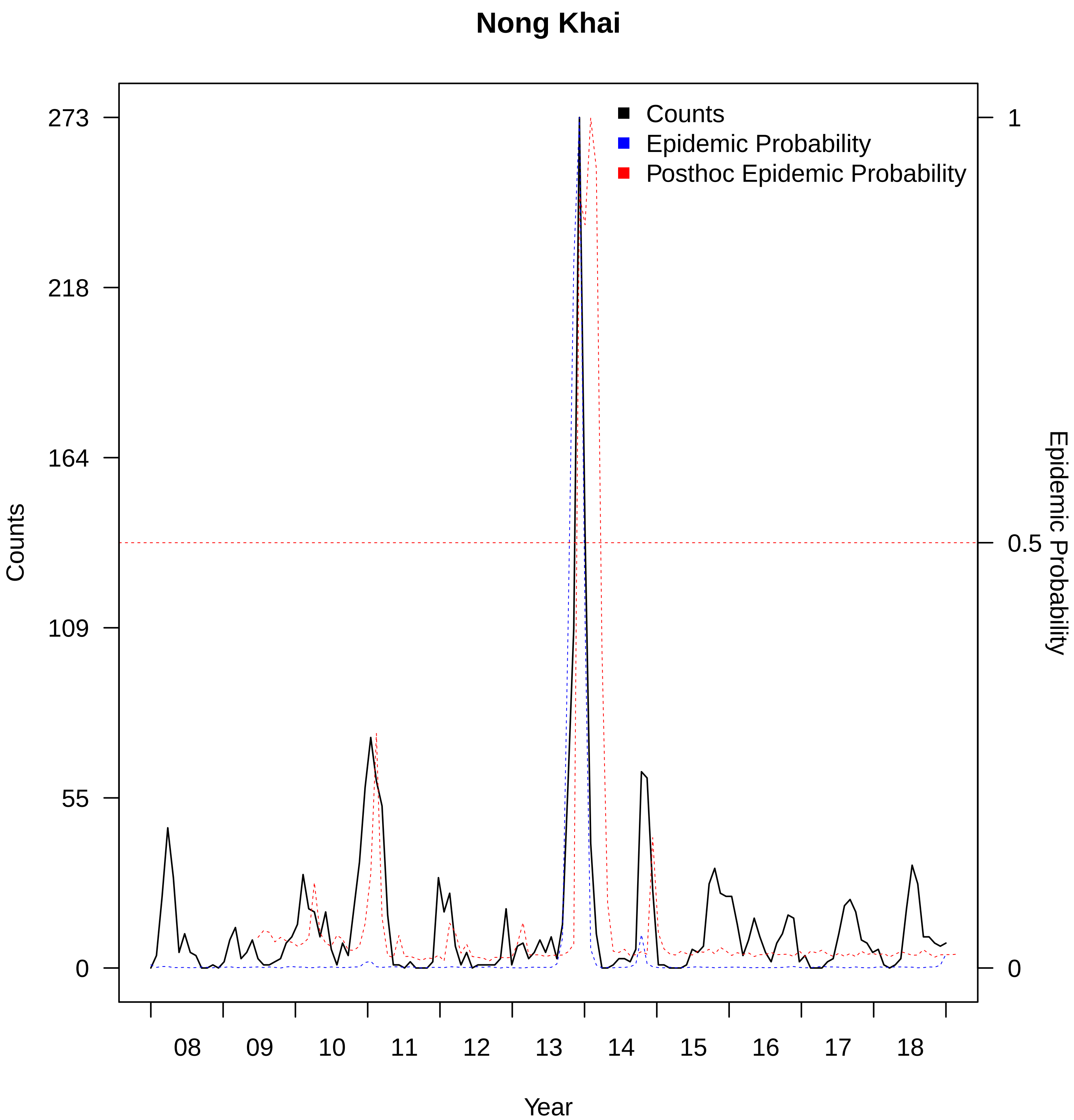
Nong Khai
Counts
Epidemic Probability
Posthoc Epidemic Probability
273
218
164
109
55
0
1
0.5
0
08
09
10
11
12
13
14
15
16
17
18
Year
Counts
Epidemic Probability

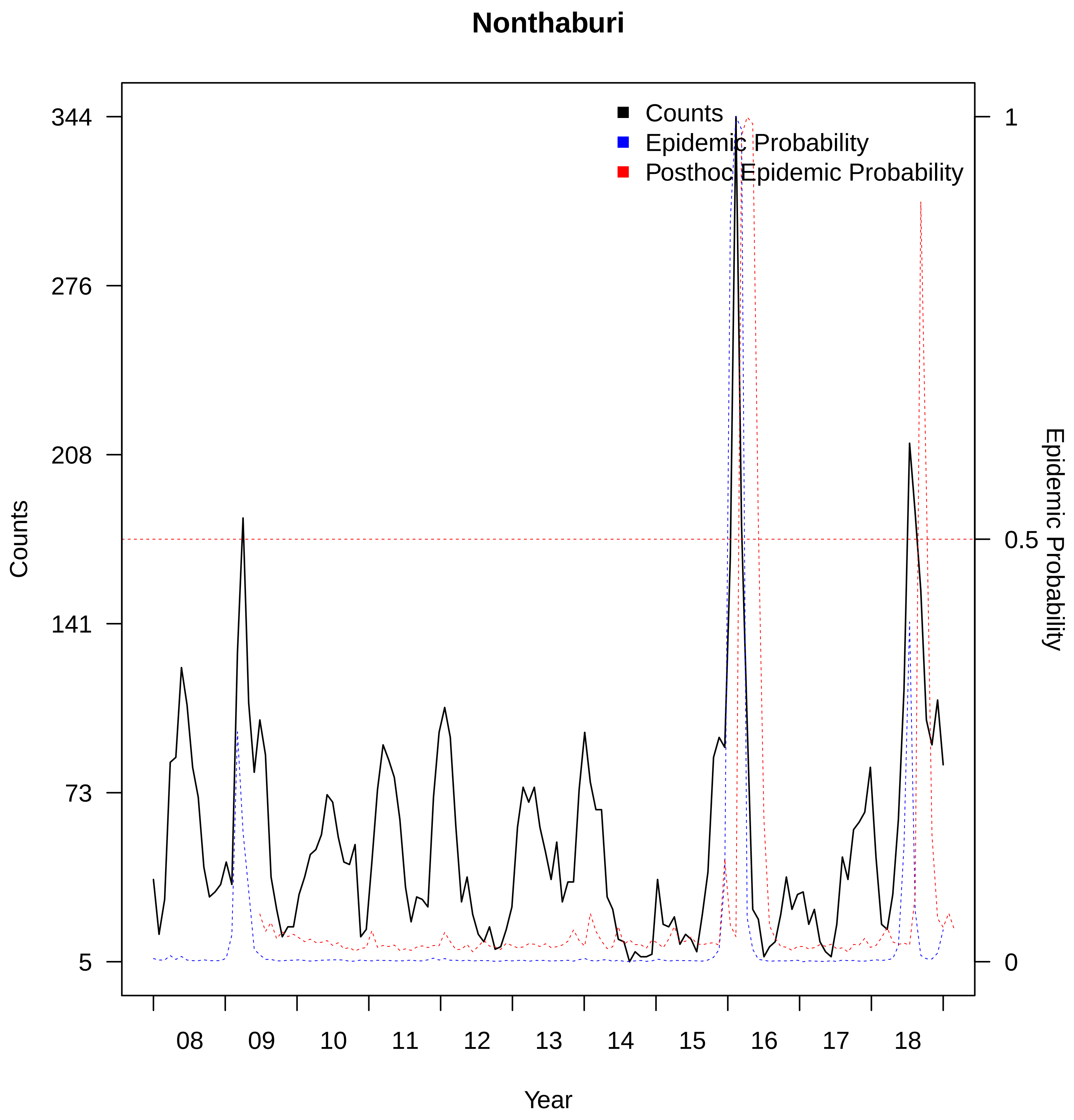
Nonthaburi
Counts
Epidemic Probability
Posthoc Epidemic Probability
344
276
208
141
73
5
1
0.5
0
08
09
10
11
12
13
14
15
16
17
18
Year
Counts
Epidemic Probability

P Nakhon S Ayutthaya

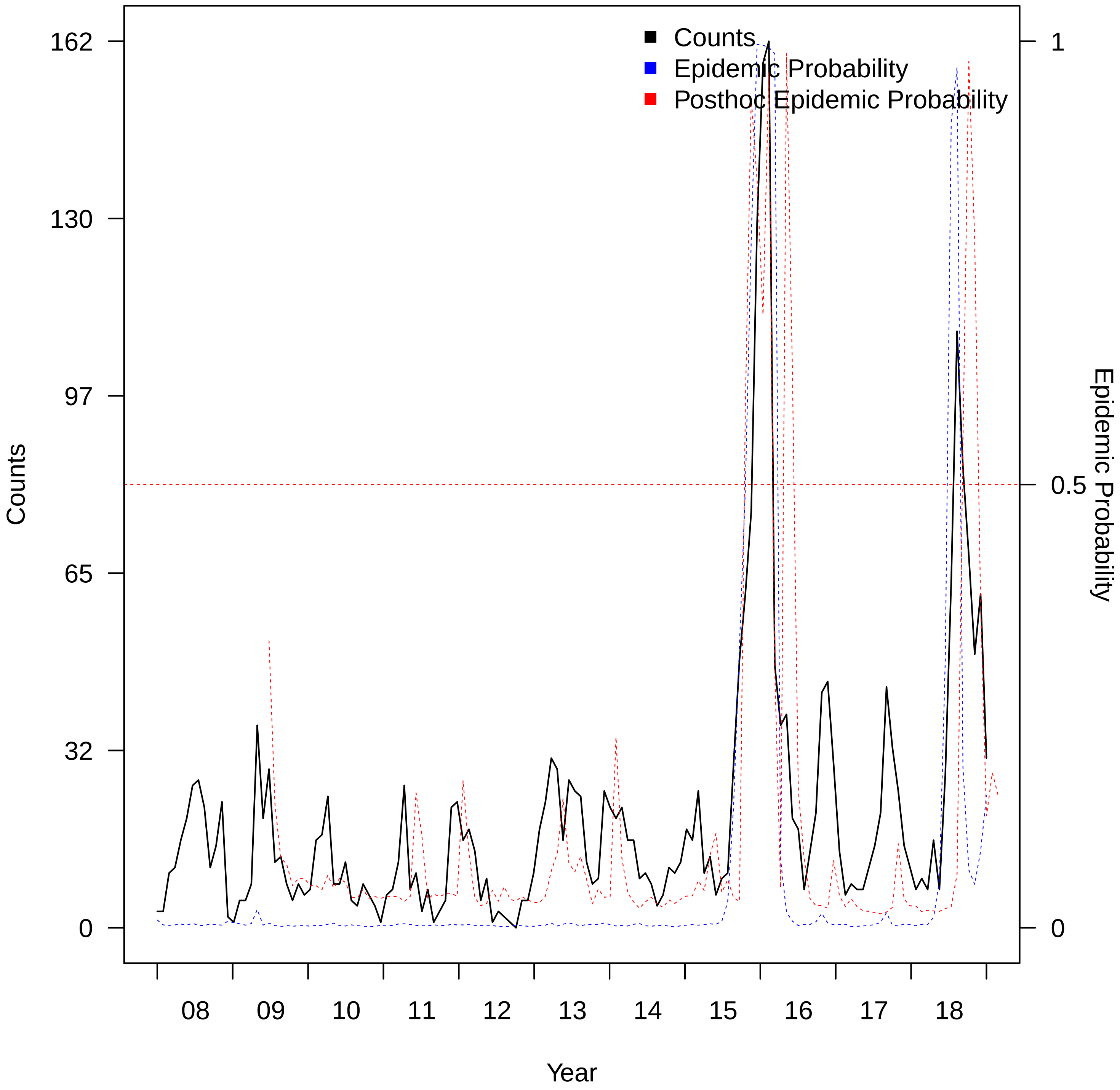

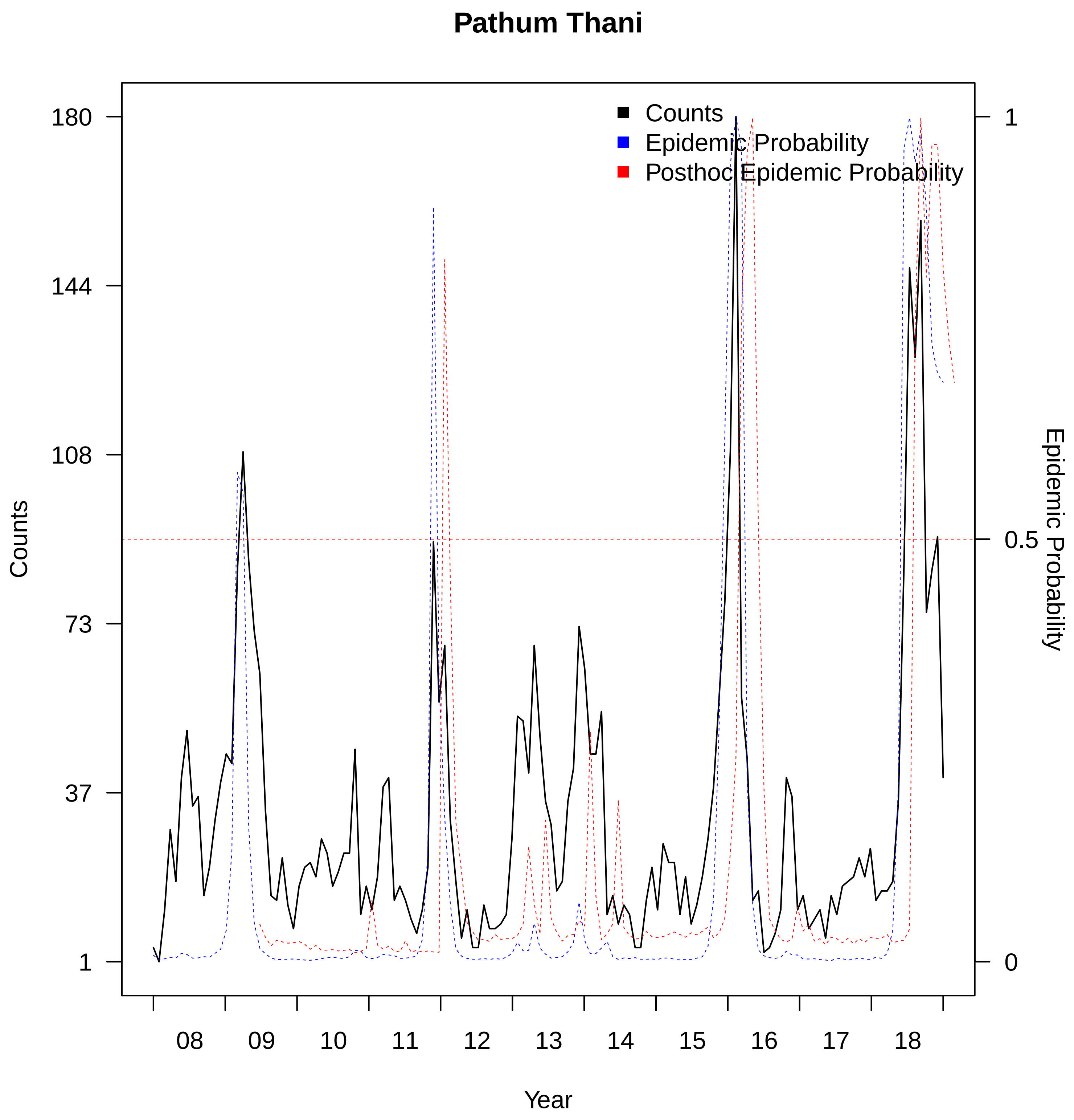
Pathum Thani
Counts
Epidemic Probability
Posthoc Epidemic Probability
180
144
108
73
37
1
1
0.5
0
Counts
Epidemic Probability
08
09
10
11
12
13
14
15
16
17
18
Year

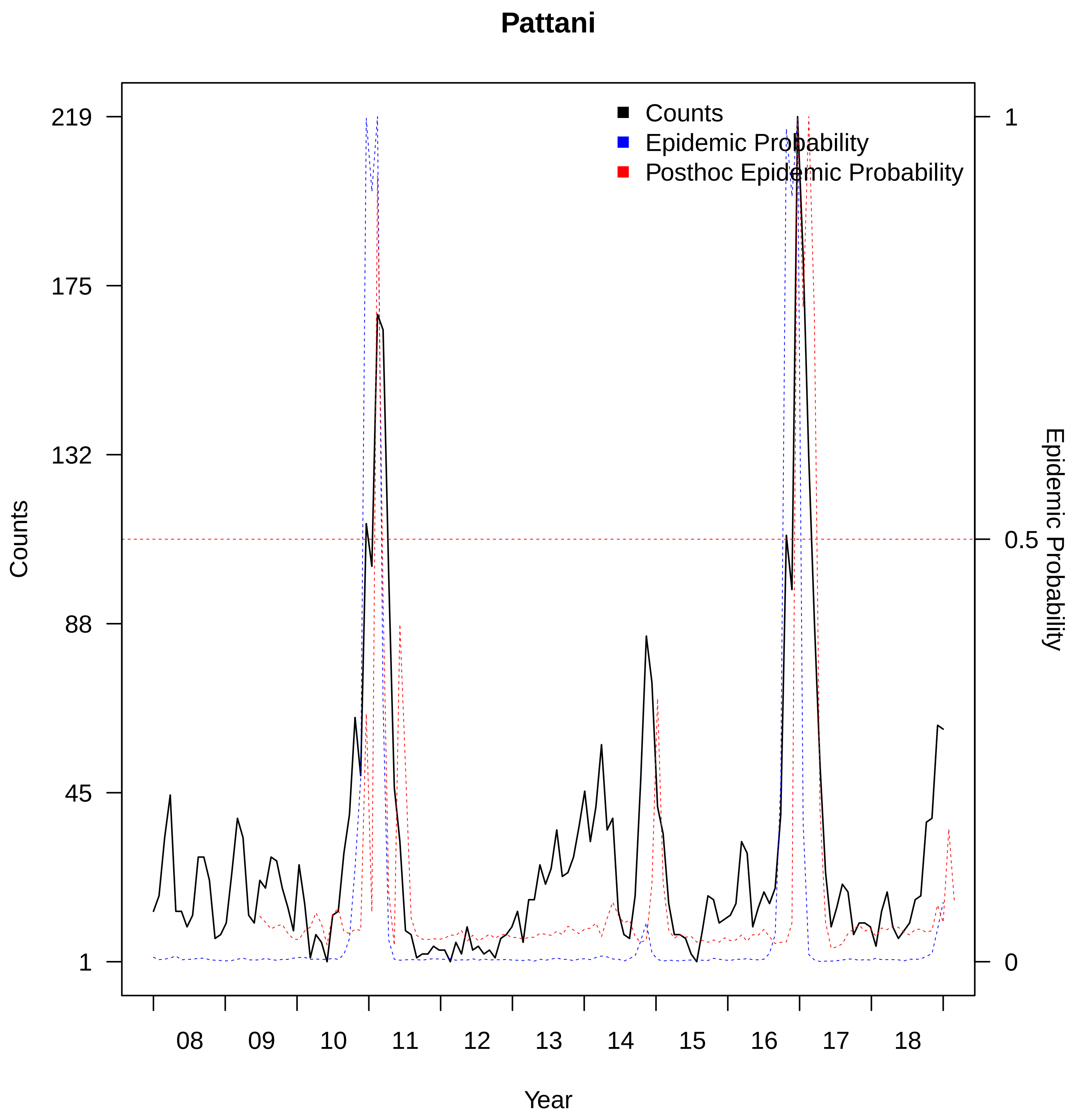

Pattani
Counts
Epidemic Probability
Posthoc Epidemic Probability
219
175
132
88
45
1
1
0.5
0
Counts
Epidemic Probability
08
09
10
11
12
13
14
15
16
17
18
Year

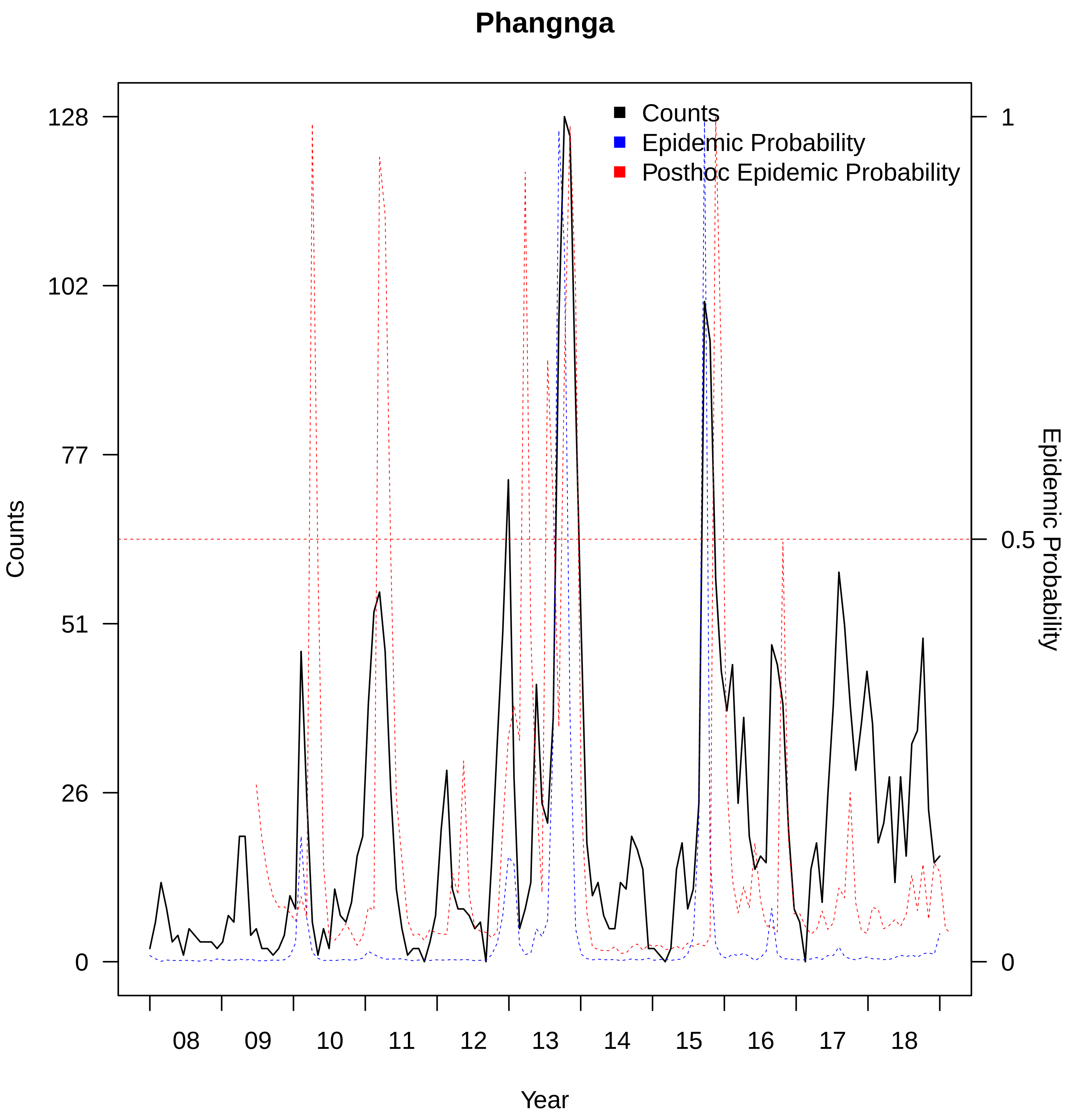
Phangnga
Counts
Epidemic Probability
Posthoc Epidemic Probability
128
102
77
51
26
0
1
0.5
0
08
09
10
11
12
13
14
15
16
17
18
Year
Counts
Epidemic Probability

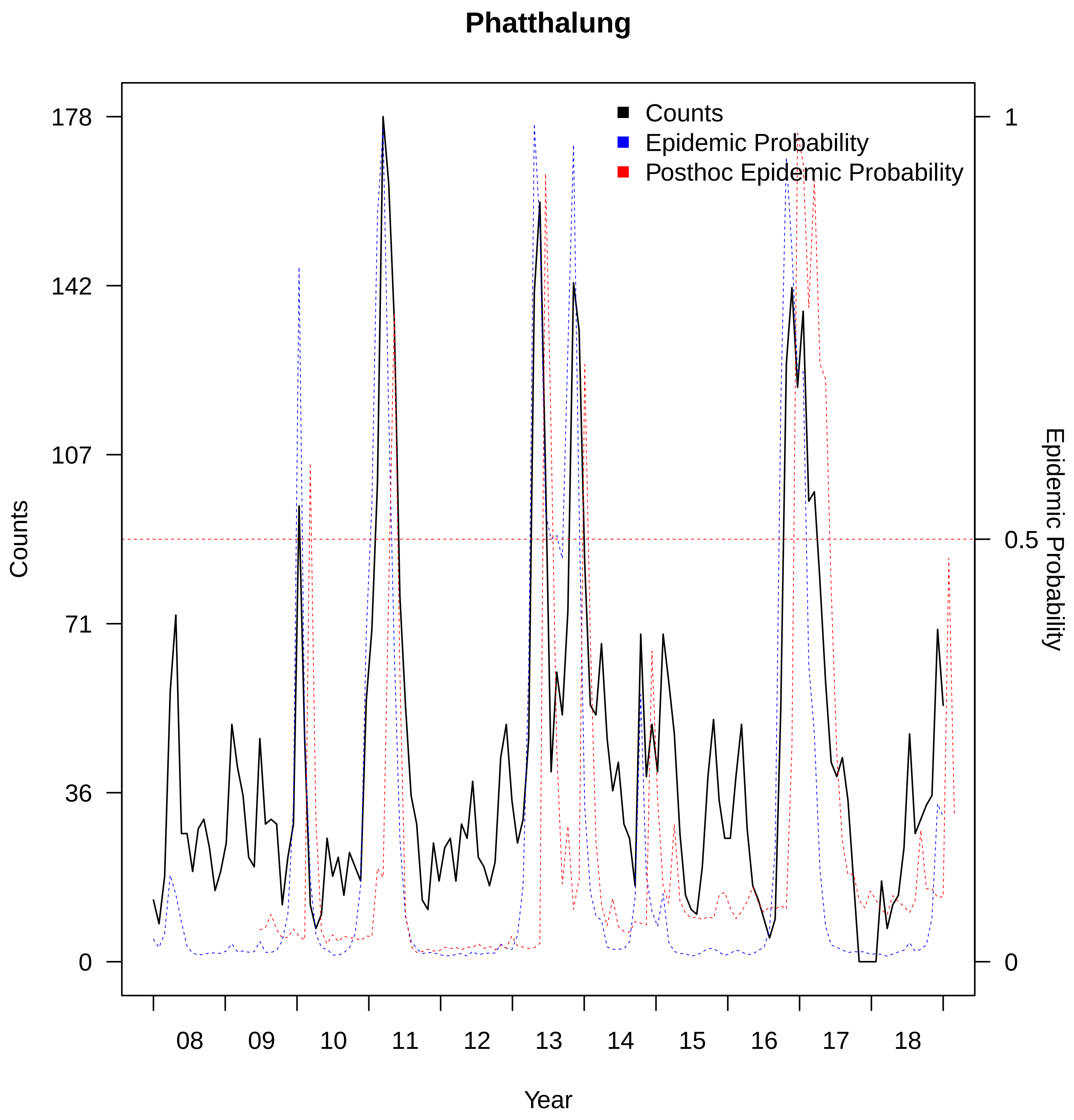

Phatthalung
Counts
Epidemic Probability
Posthoc Epidemic Probability
178
142
107
71
36
0
1
0.5
0
Counts
Epidemic Probability
08
09
10
11
12
13
14
15
16
17
18
Year

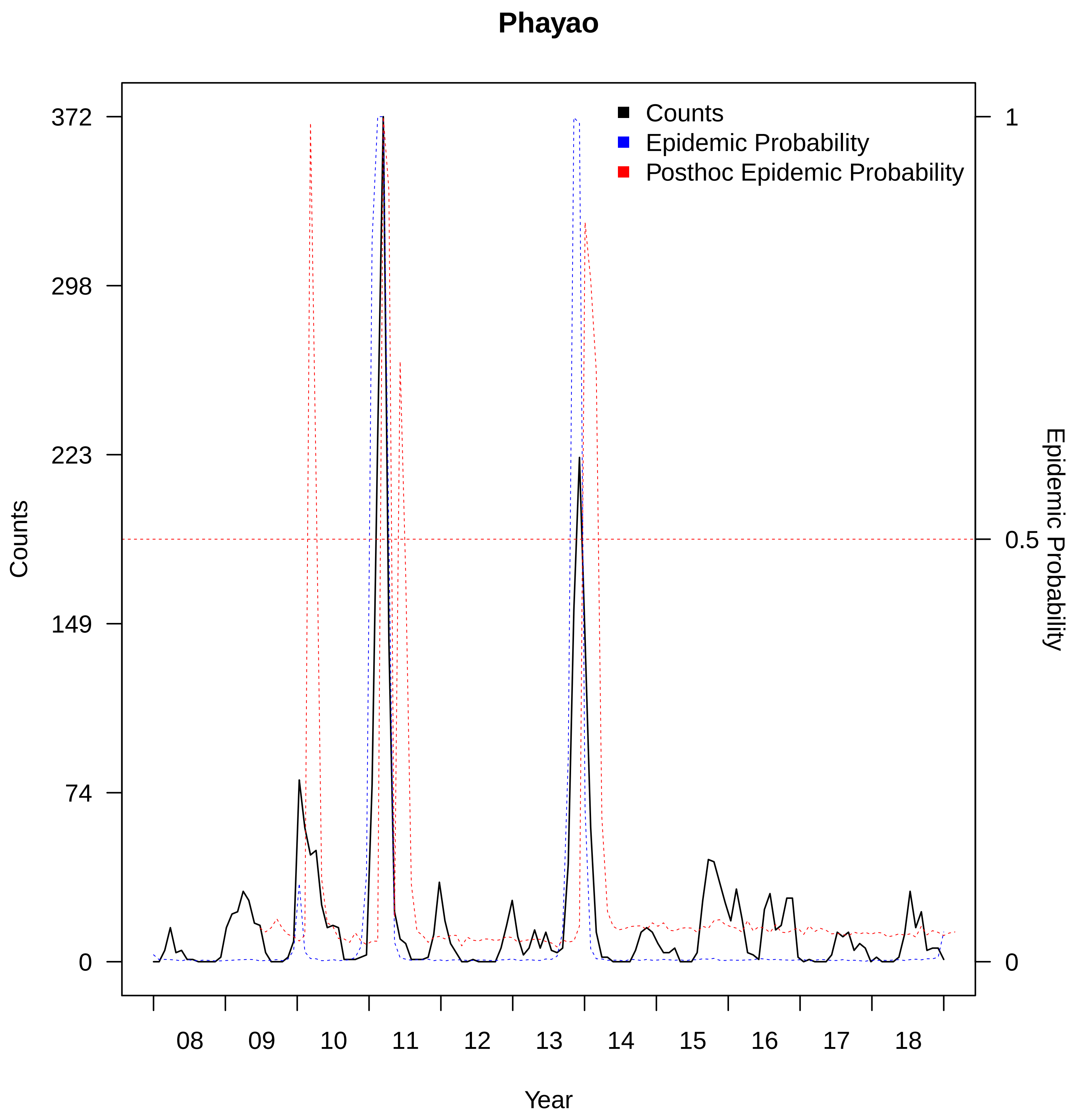
Phayao
Counts
Epidemic Probability
Posthoc Epidemic Probability
372
298
223
149
74
0
1
0.5
0
08
09
10
11
12
13
14
15
16
17
18
Year
Counts
Epidemic Probability

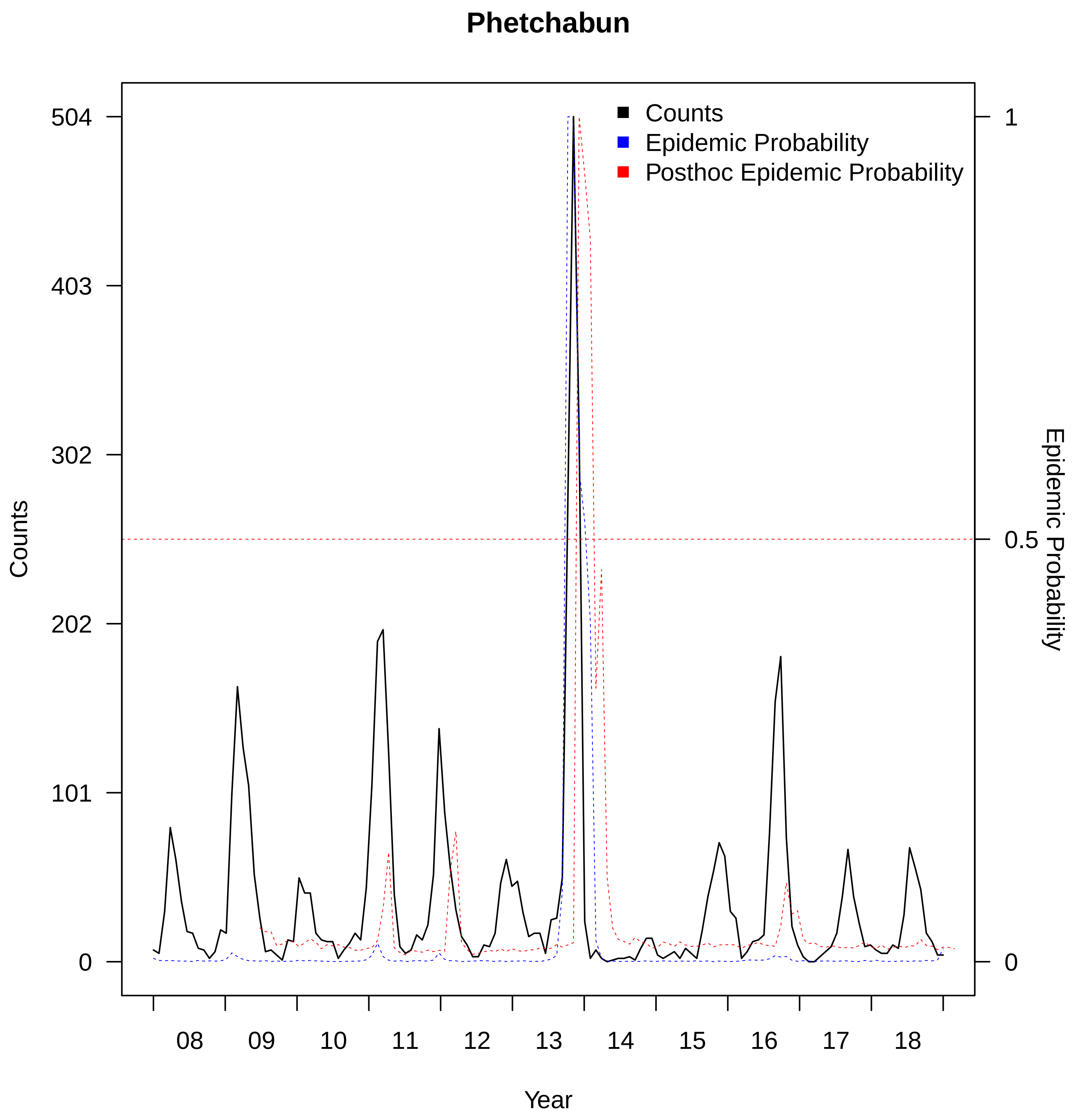

Phetchabun
Counts
Epidemic Probability
Posthoc Epidemic Probability
504
403
302
202
101
0
1
0.5
0
08
09
10
11
12
13
14
15
16
17
18
Year
Counts
Epidemic Probability

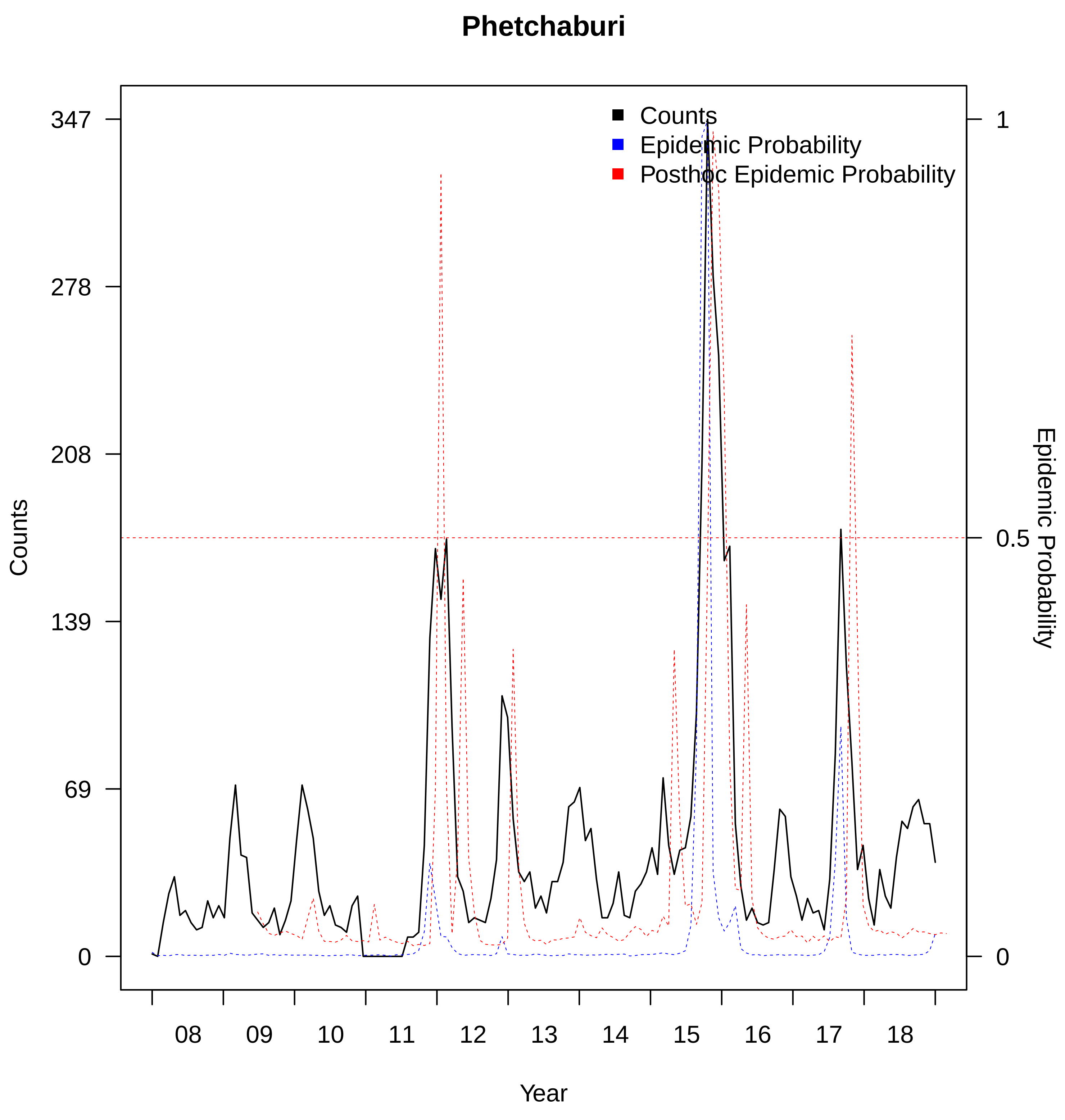
Phetchaburi
Counts
Epidemic Probability
Posthoc Epidemic Probability
347
278
208
139
69
0
1
0.5
0
08
09
10
11
12
13
14
15
16
17
18
Year
Counts
Epidemic Probability

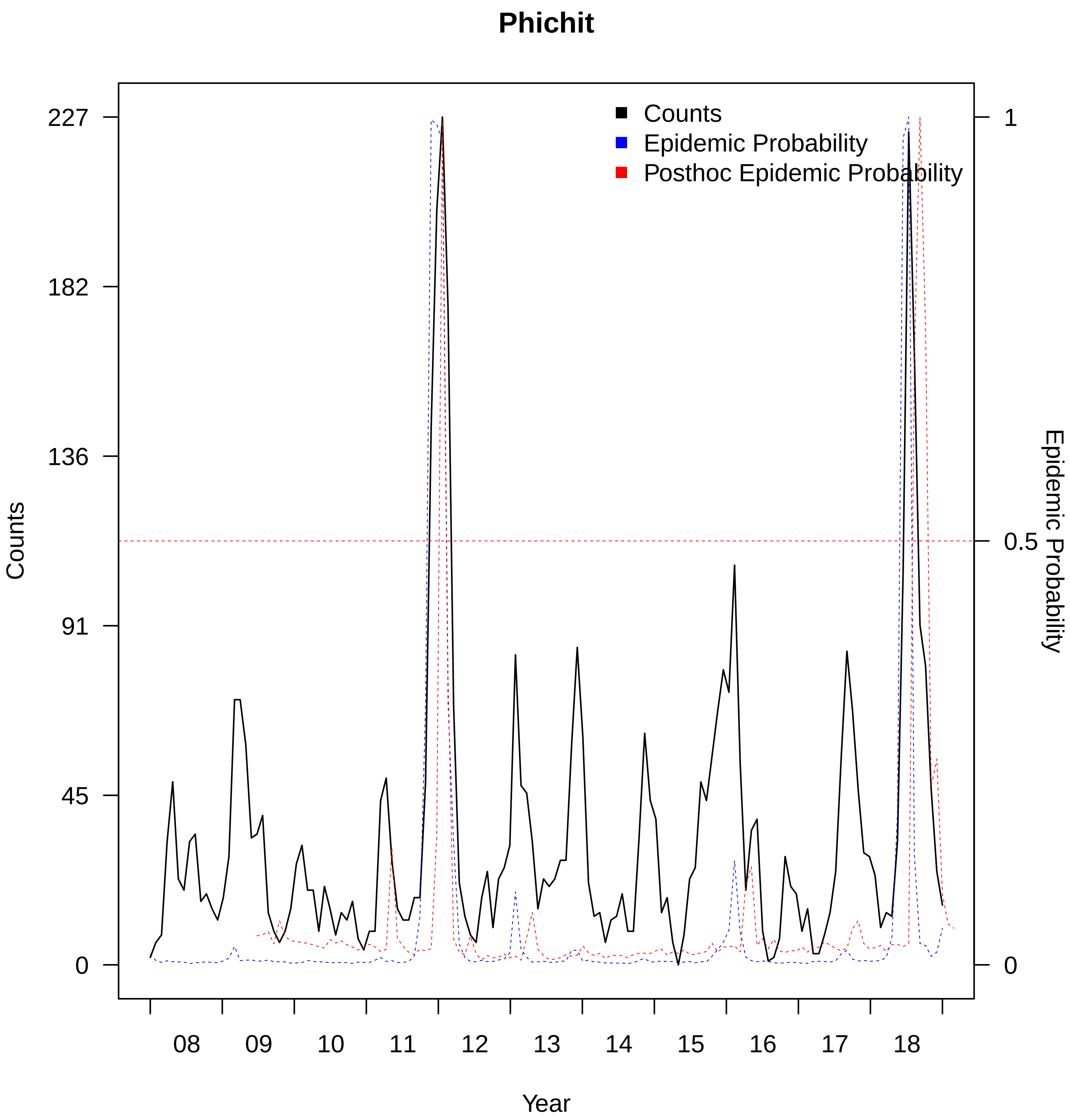

Phichit
Counts
Epidemic Probability
Posthoc Epidemic Probability
227
182
136
91
45
0
1
0.5
0
Counts
Epidemic Probability
08
09
10
11
12
13
14
15
16
17
18
Year

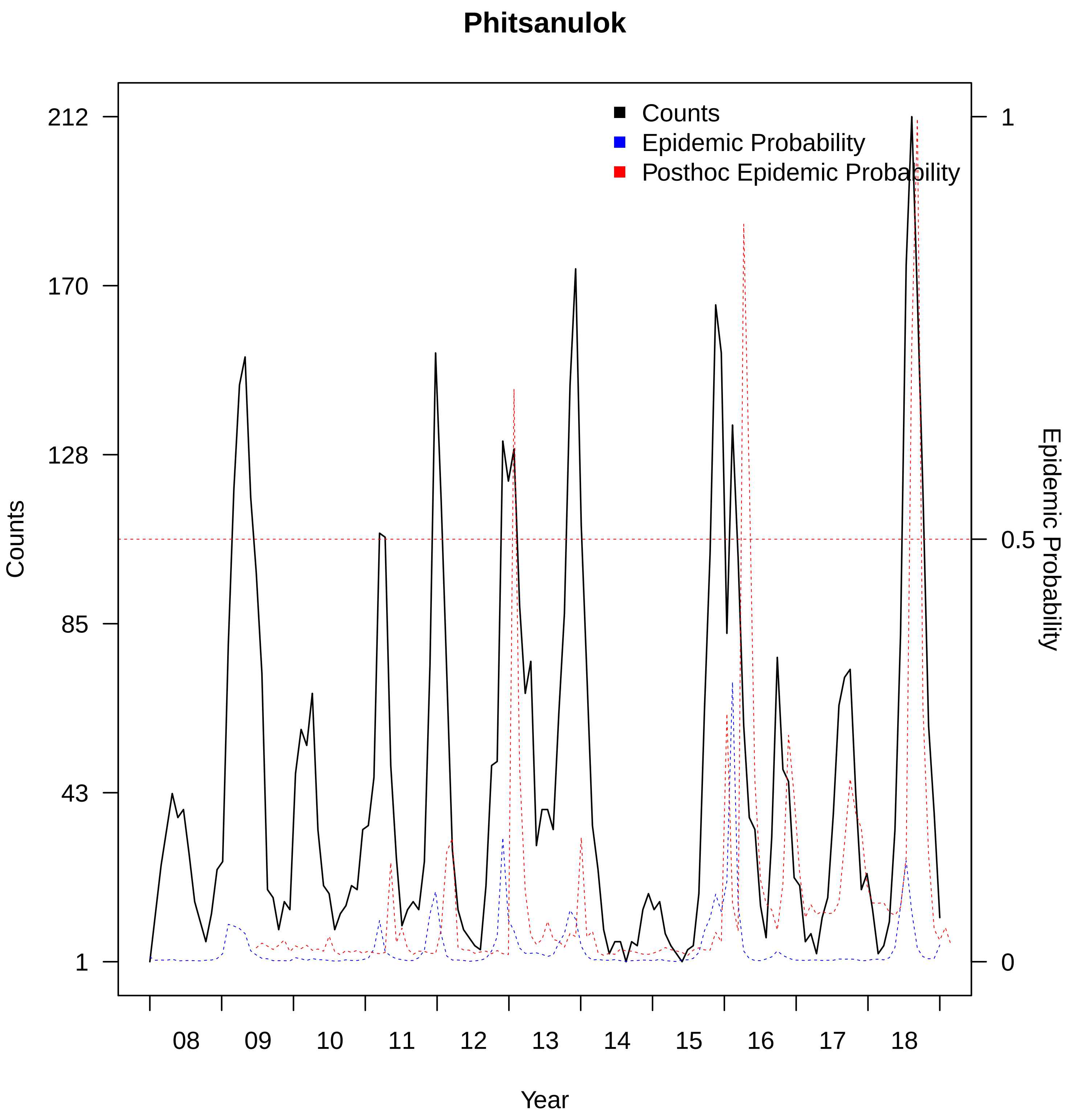

Phitsanulok
Counts
Epidemic Probability
Posthoc Epidemic Probability
212
170
128
85
43
1
1
0.5
0
08
09
10
11
12
13
14
15
16
17
18
Year
Counts
Epidemic Probability

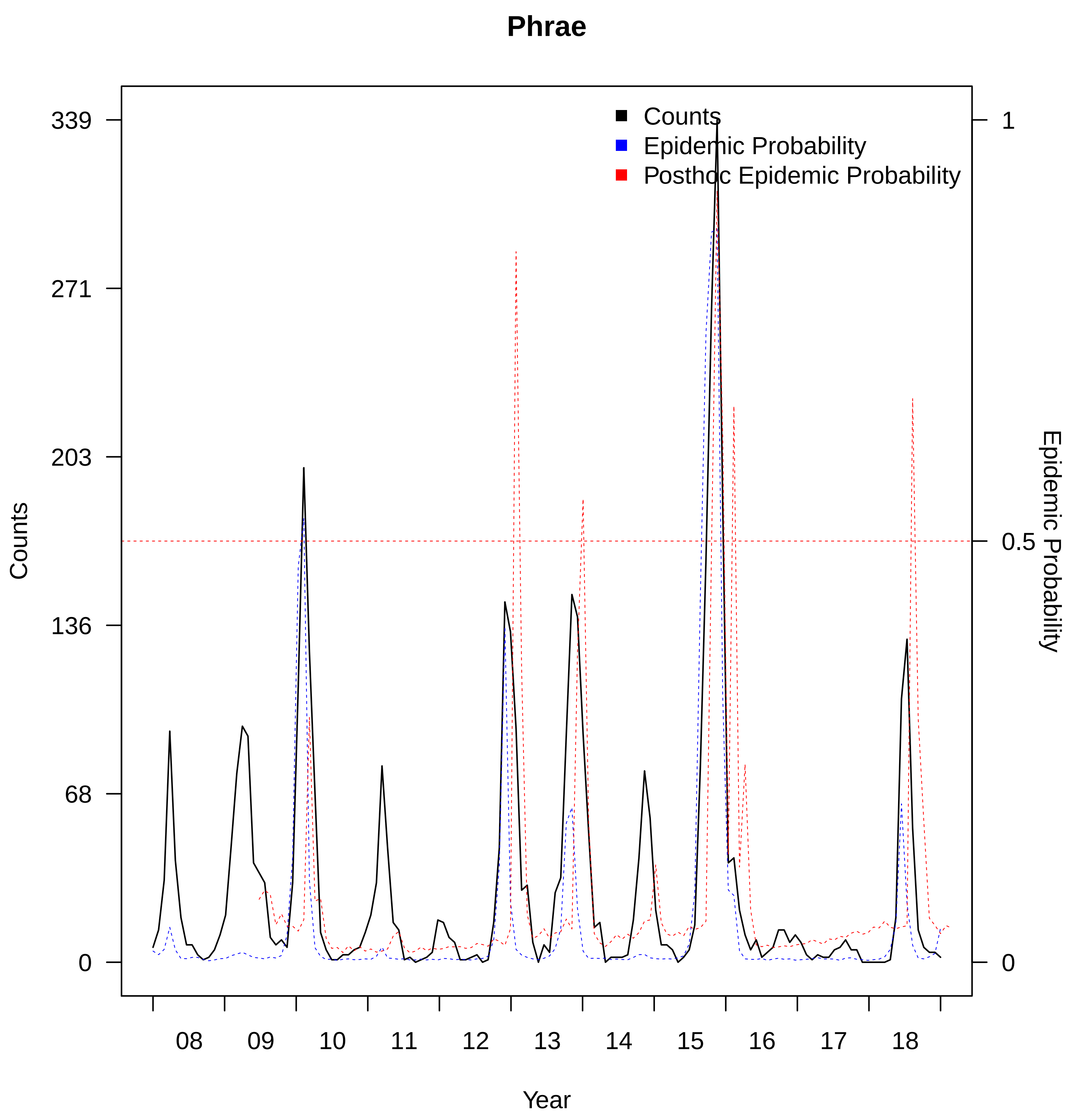

Phrae
Counts
Epidemic Probability
Posthoc Epidemic Probability
339
271
203
136
68
0
1
0.5
0
08
09
10
11
12
13
14
15
16
17
18
Year
Counts
Epidemic Probability

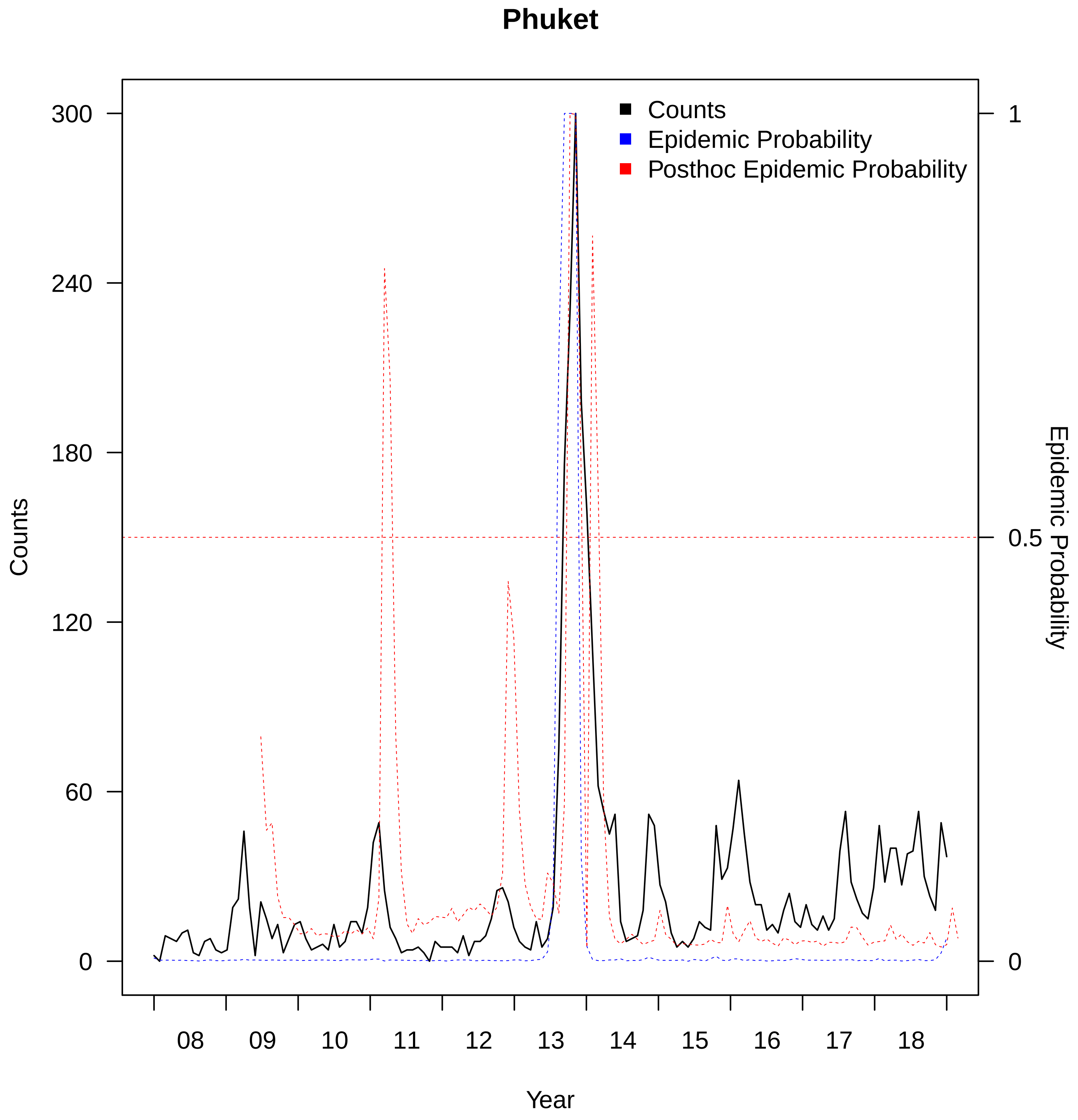
Phuket
Counts
Epidemic Probability
Posthoc Epidemic Probability
300
240
180
120
60
0
1
0.5
0
08
09
10
11
12
13
14
15
16
17
18
Year
Counts
Epidemic Probability

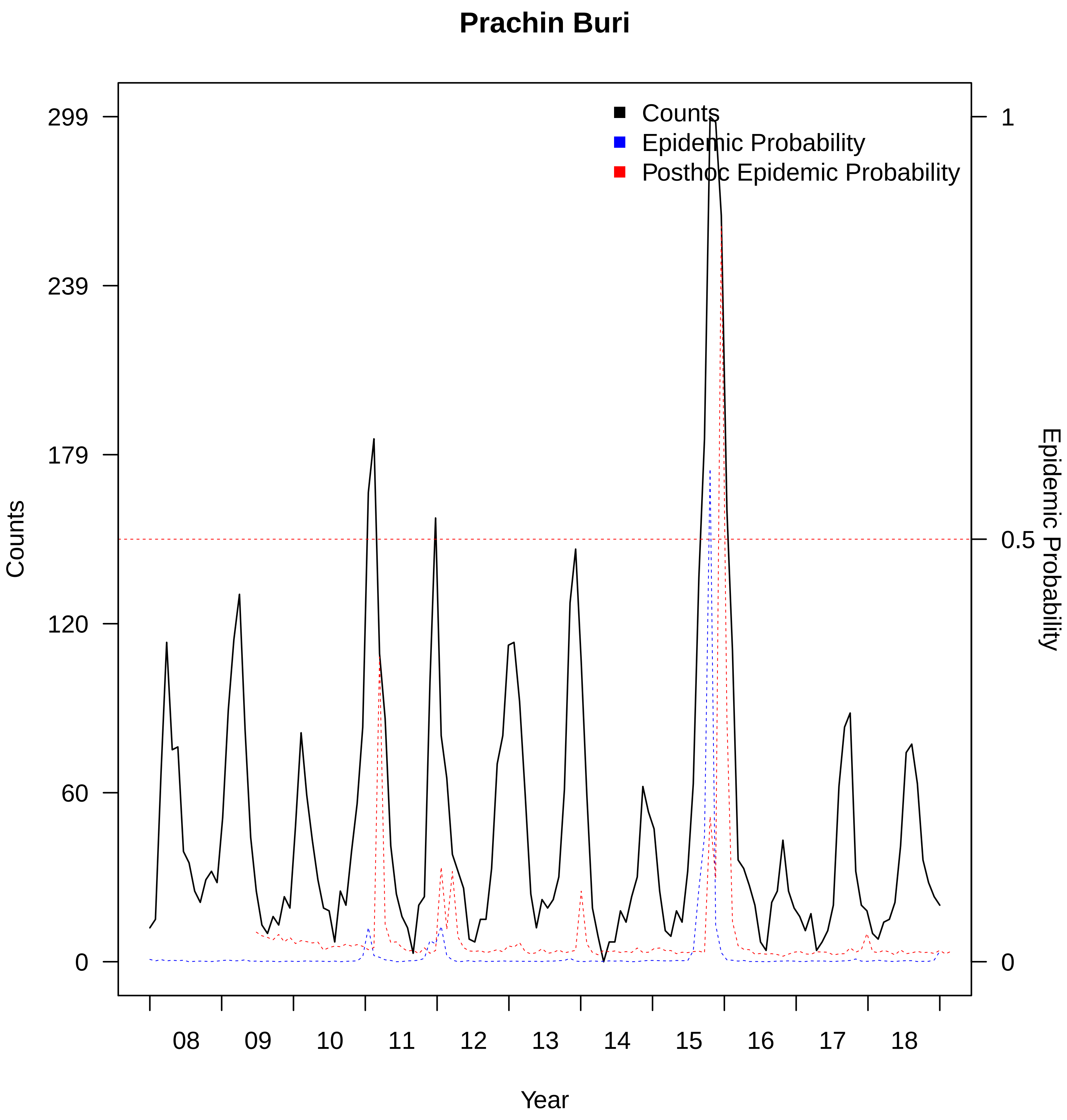

Prachin Buri
Counts
Epidemic Probability
Posthoc Epidemic Probability
299
239
179
120
60
0
1
0.5
0
08
09
10
11
12
13
14
15
16
17
18
Year
Counts
Epidemic Probability

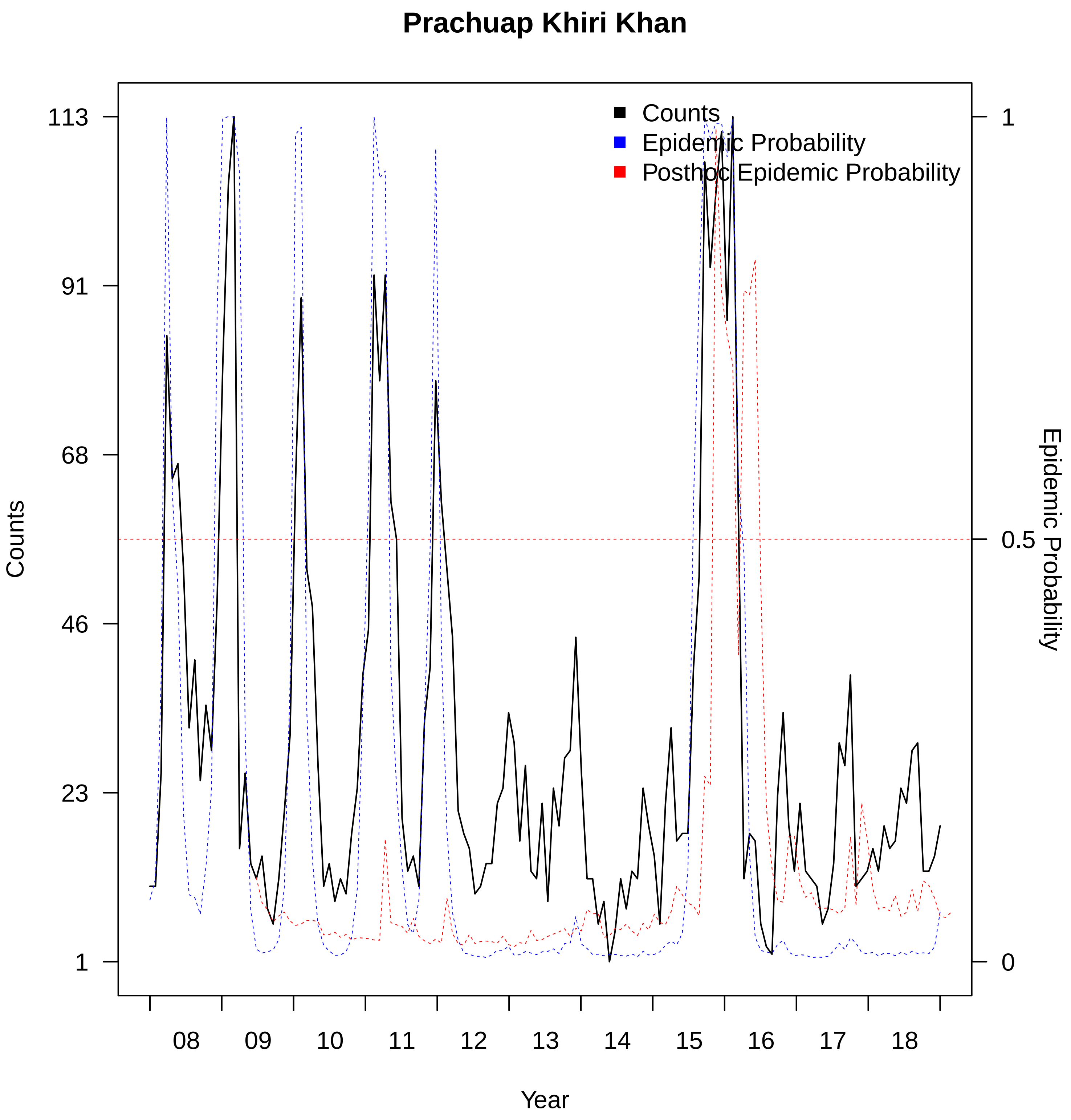
Prachuap Khiri Khan
Counts
Epidemic Probability
Posthoc Epidemic Probability
113
91
68
46
23
1
1
0.5
0
Counts
Epidemic Probability
08
09
10
11
12
13
14
15
16
17
18
Year

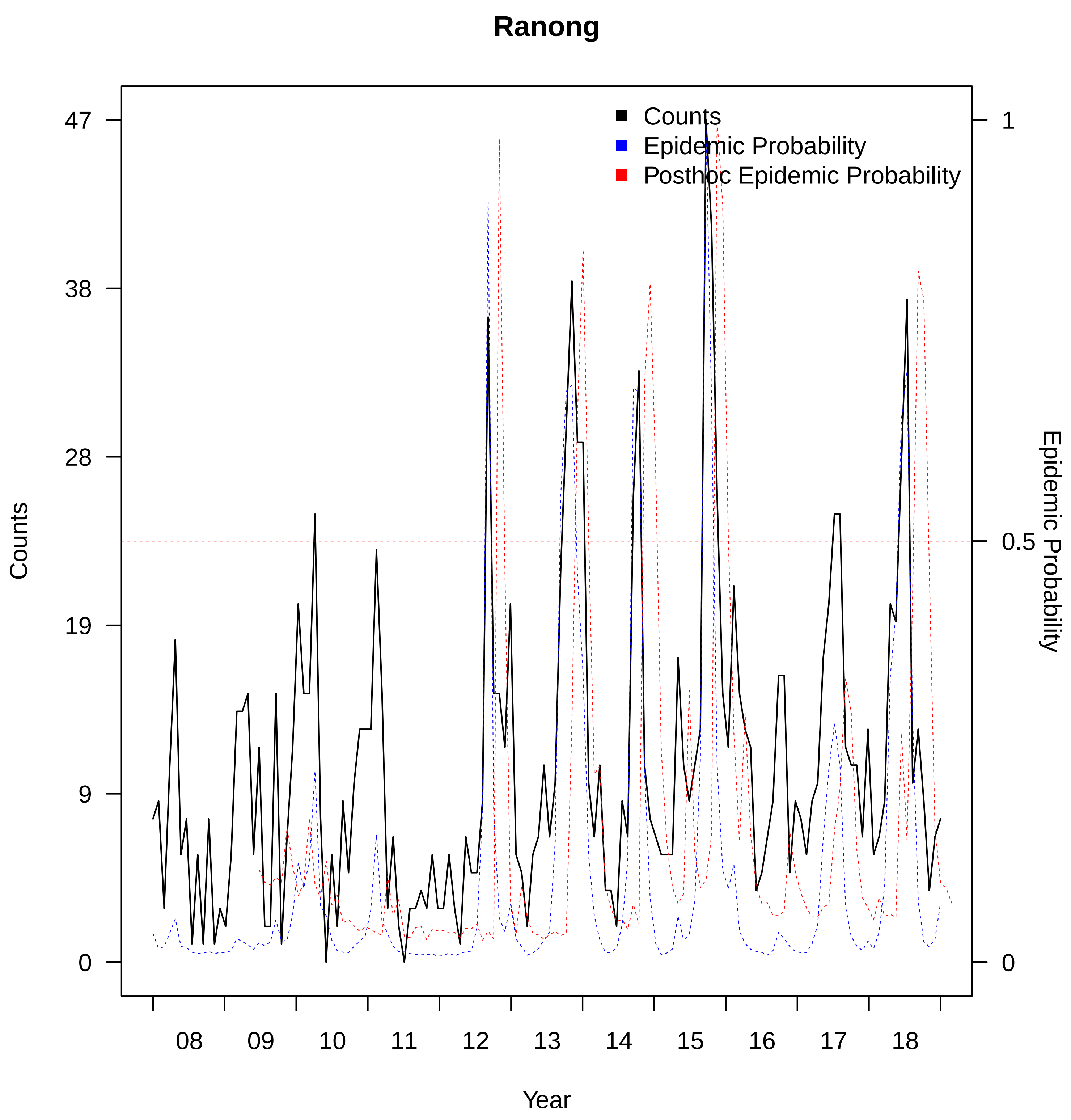
Ranong
Counts
Epidemic Probability
Posthoc Epidemic Probability
Counts
47
38
28
19
9
0
Epidemic Probability
1
0.5
0
08
09
10
11
12
13
14
15
16
17
18
Year

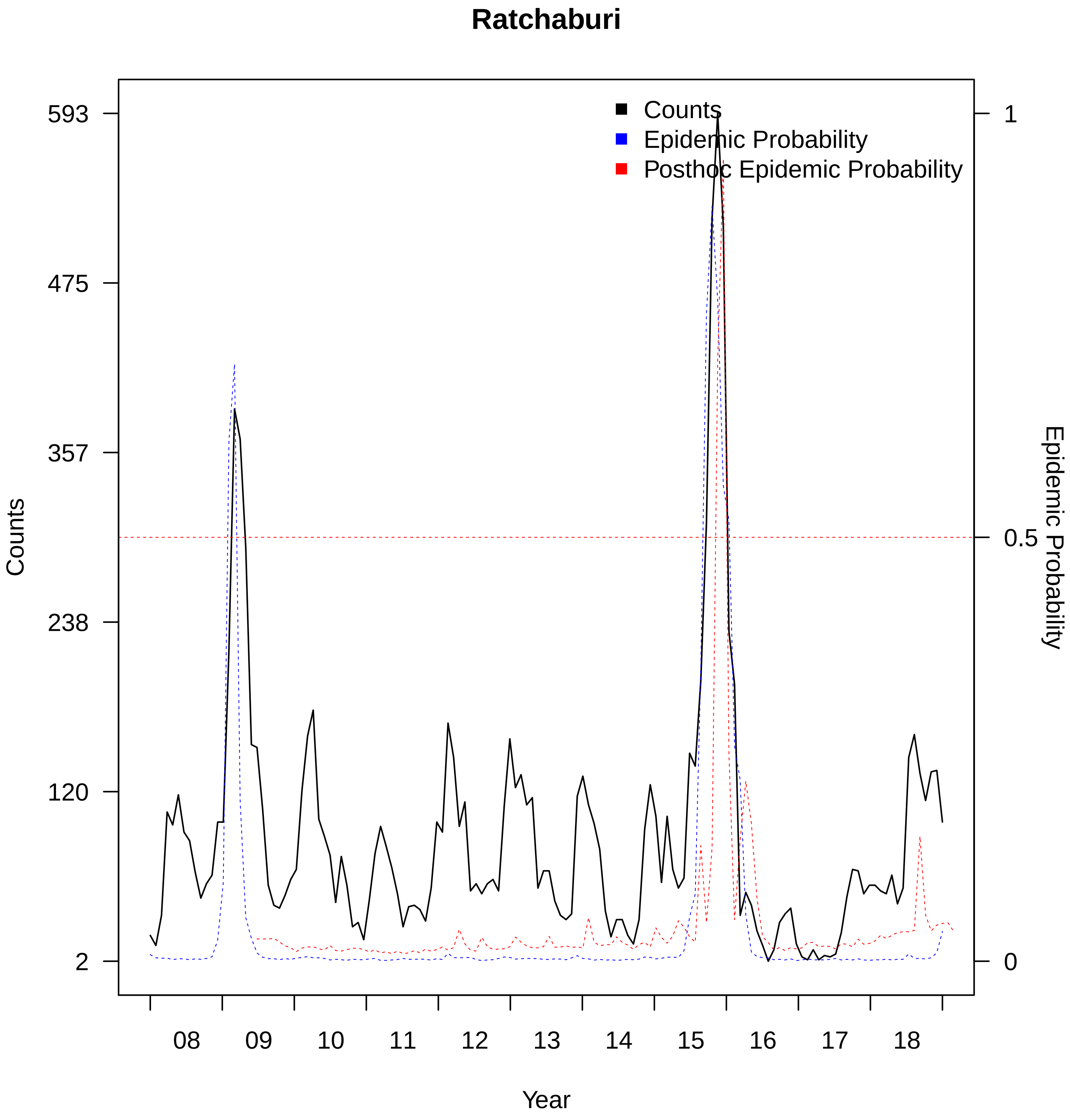

Ratchaburi
Counts
Epidemic Probability
Posthoc Epidemic Probability
593
475
357
238
120
2
1
0.5
0
Counts
Epidemic Probability
08
09
10
11
12
13
14
15
16
17
18
Year

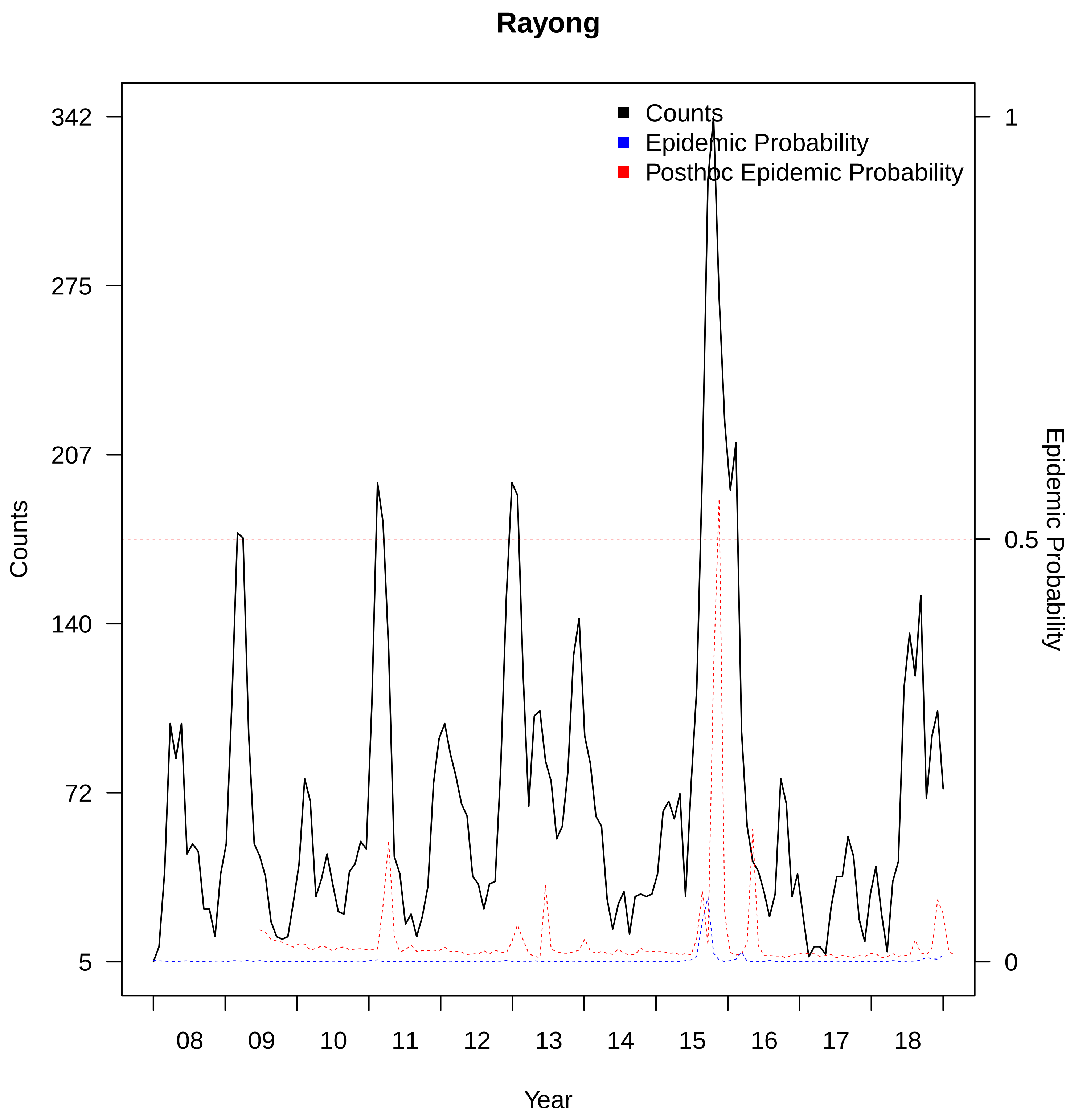

Rayong
Counts
Epidemic Probability
Posthoc Epidemic Probability
342
275
207
140
72
5
1
0.5
0
Counts
Epidemic Probability
08
09
10
11
12
13
14
15
16
17
18
Year

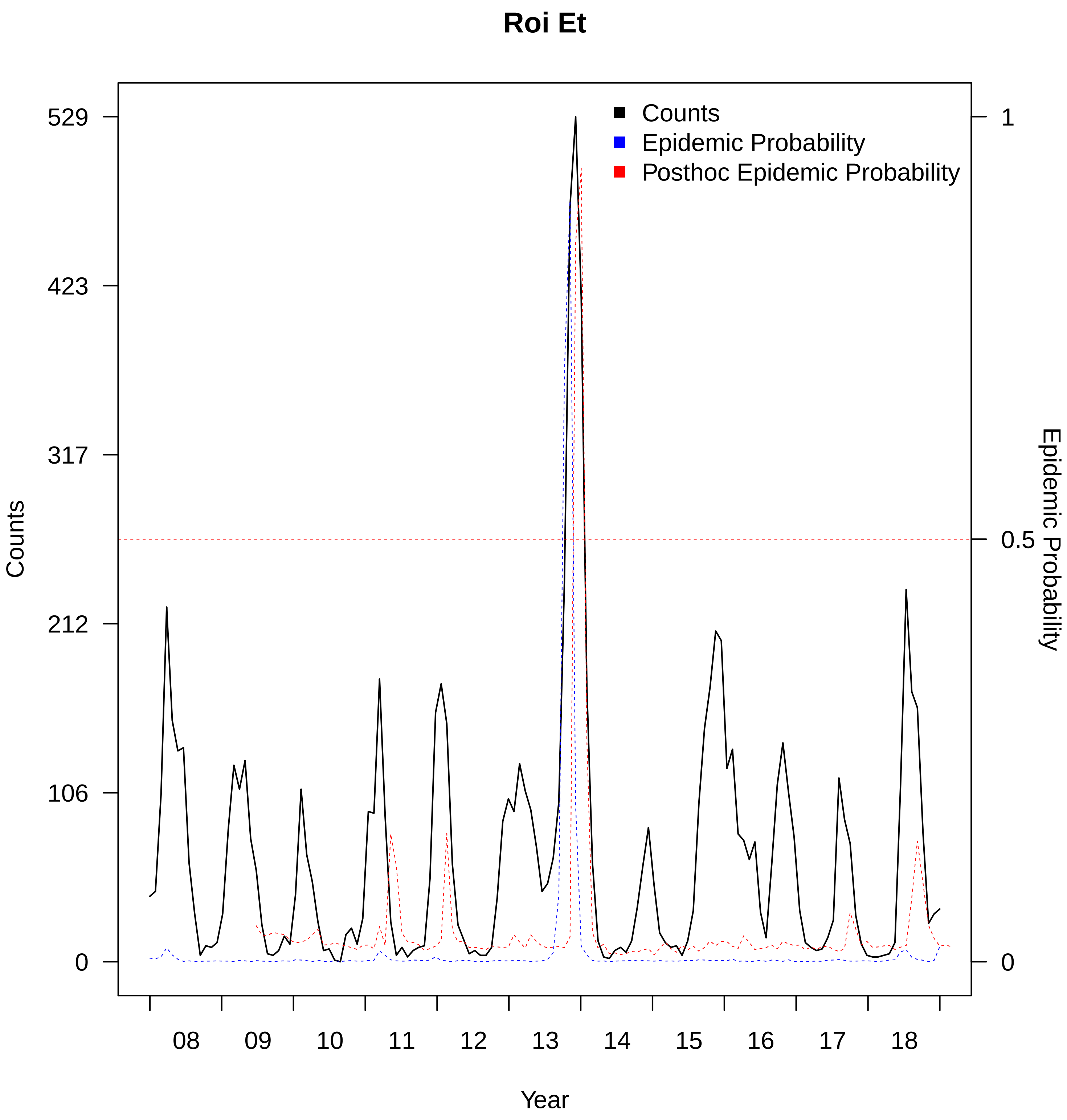

Roi Et
Counts
Epidemic Probability
Posthoc Epidemic Probability
529
423
317
212
106
0
1
0.5
0
Counts
Epidemic Probability
08
09
10
11
12
13
14
15
16
17
18
Year

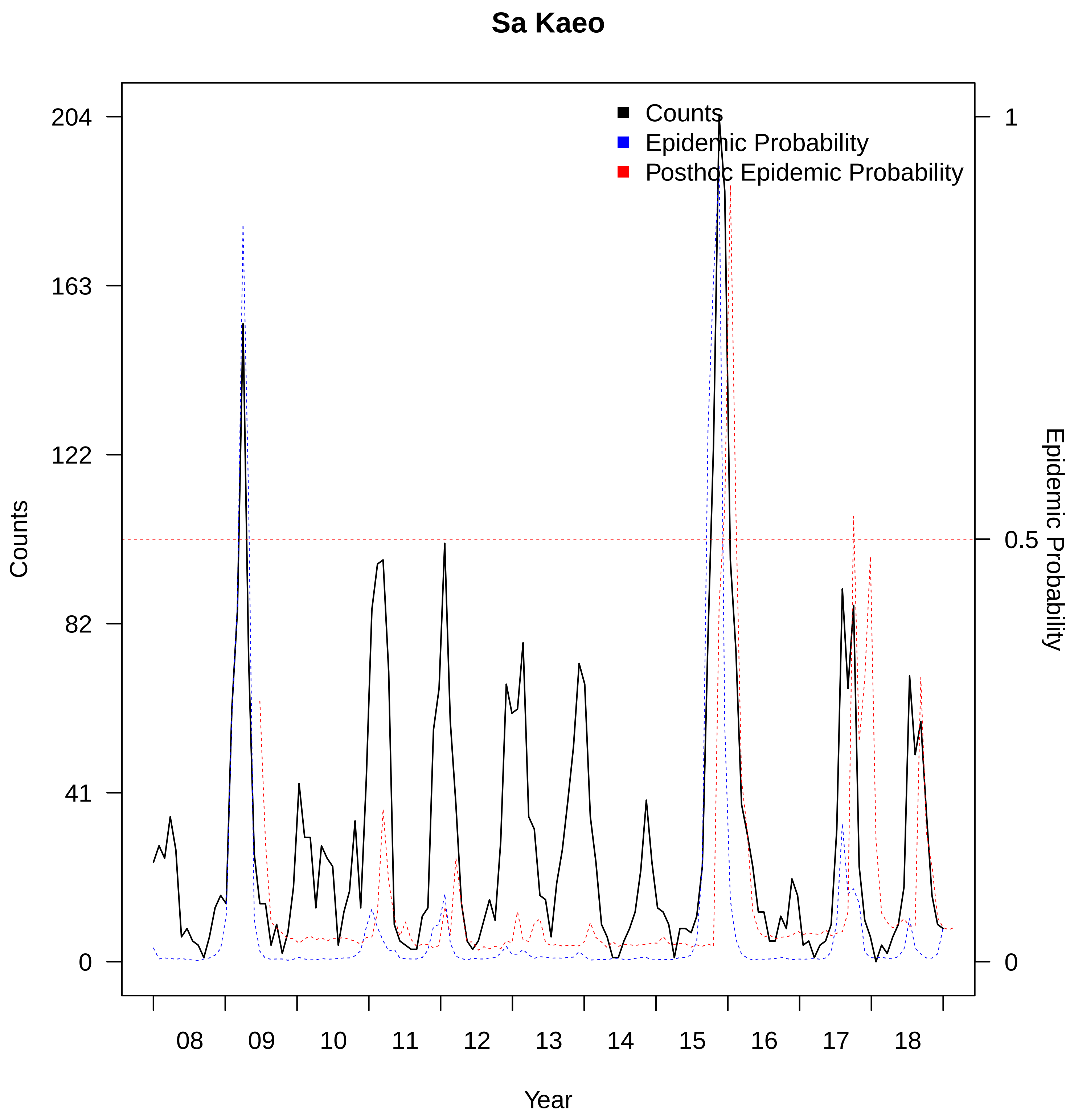

Sa Kaeo
Counts
Epidemic Probability
Posthoc Epidemic Probability
204
163
122
82
41
0
1
0.5
0
Counts
Epidemic Probability
08
09
10
11
12
13
14
15
16
17
18
Year

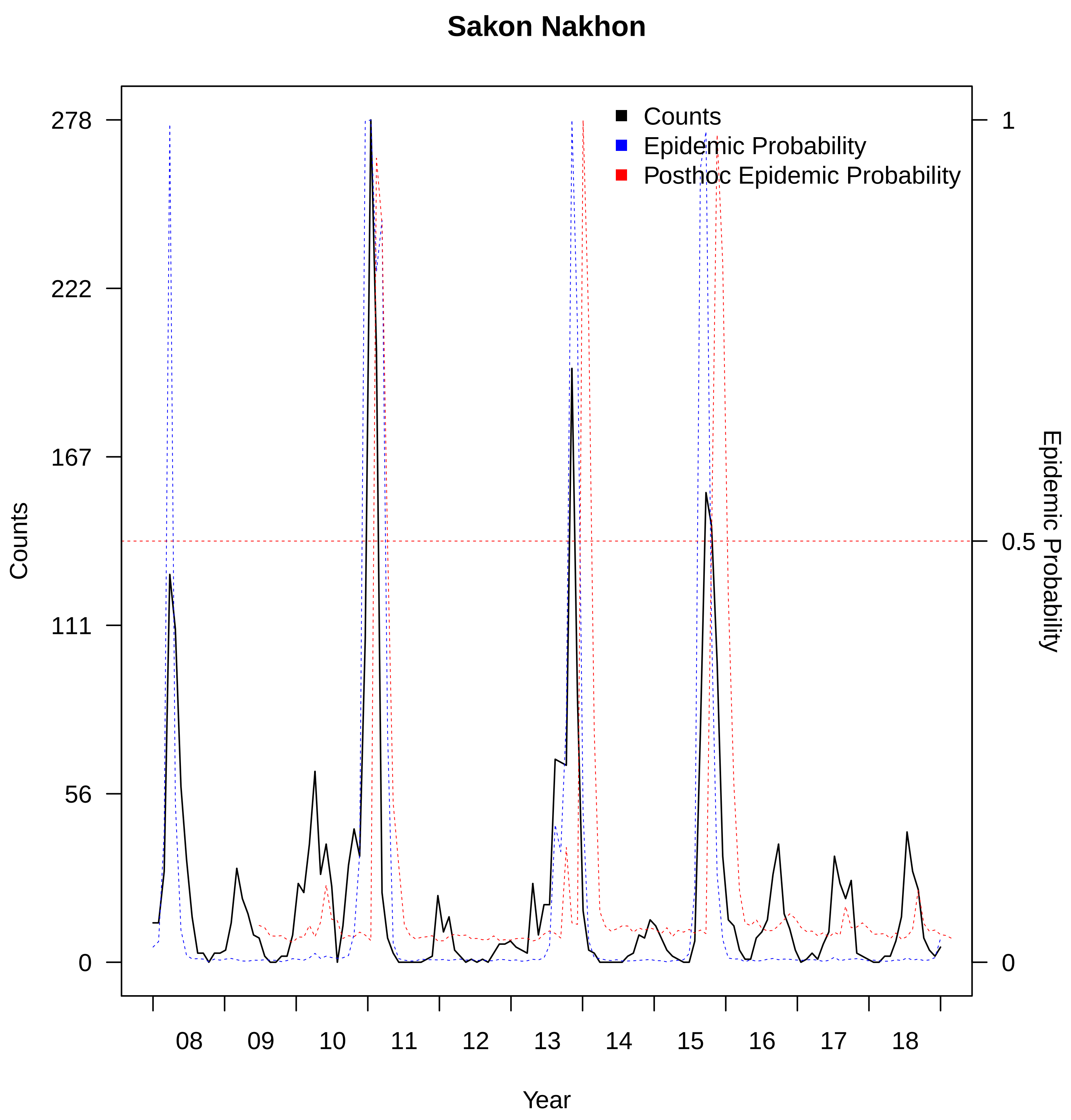

Sakon Nakhon
Counts
Epidemic Probability
Posthoc Epidemic Probability
278
222
167
111
56
0
1
0.5
0
08
09
10
11
12
13
14
15
16
17
18
Year
Counts
Epidemic Probability

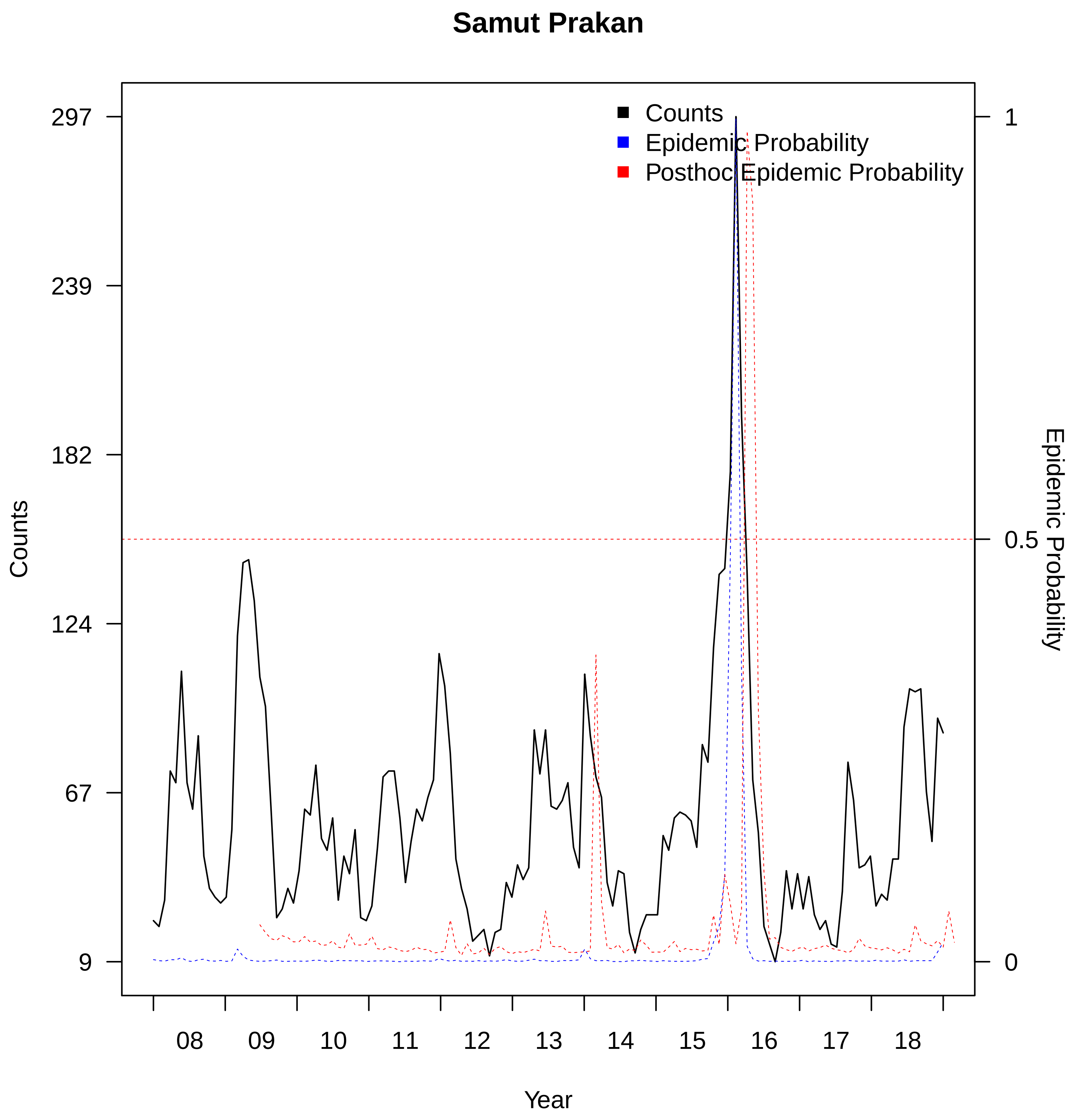
Samut Prakan
Counts
Epidemic Probability
Posthoc Epidemic Probability
297
239
182
124
67
9
1
0.5
0
08
09
10
11
12
13
14
15
16
17
18
Year
Counts
Epidemic Probability

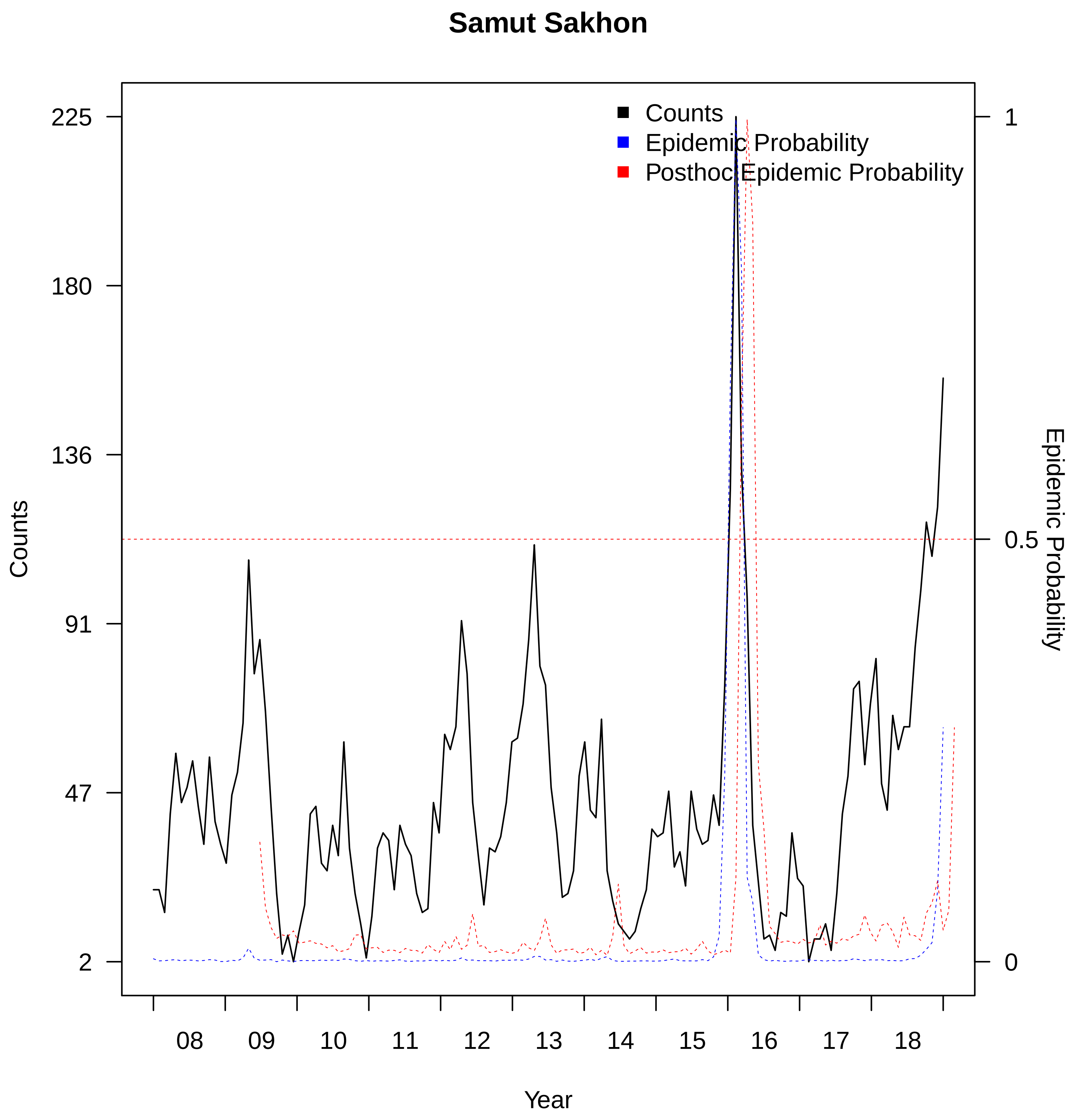
Samut Sakhon
Counts
Epidemic Probability
Posthoc Epidemic Probability
225
180
136
91
47
2
1
0.5
0
08
09
10
11
12
13
14
15
16
17
18
Year
Counts
Epidemic Probability

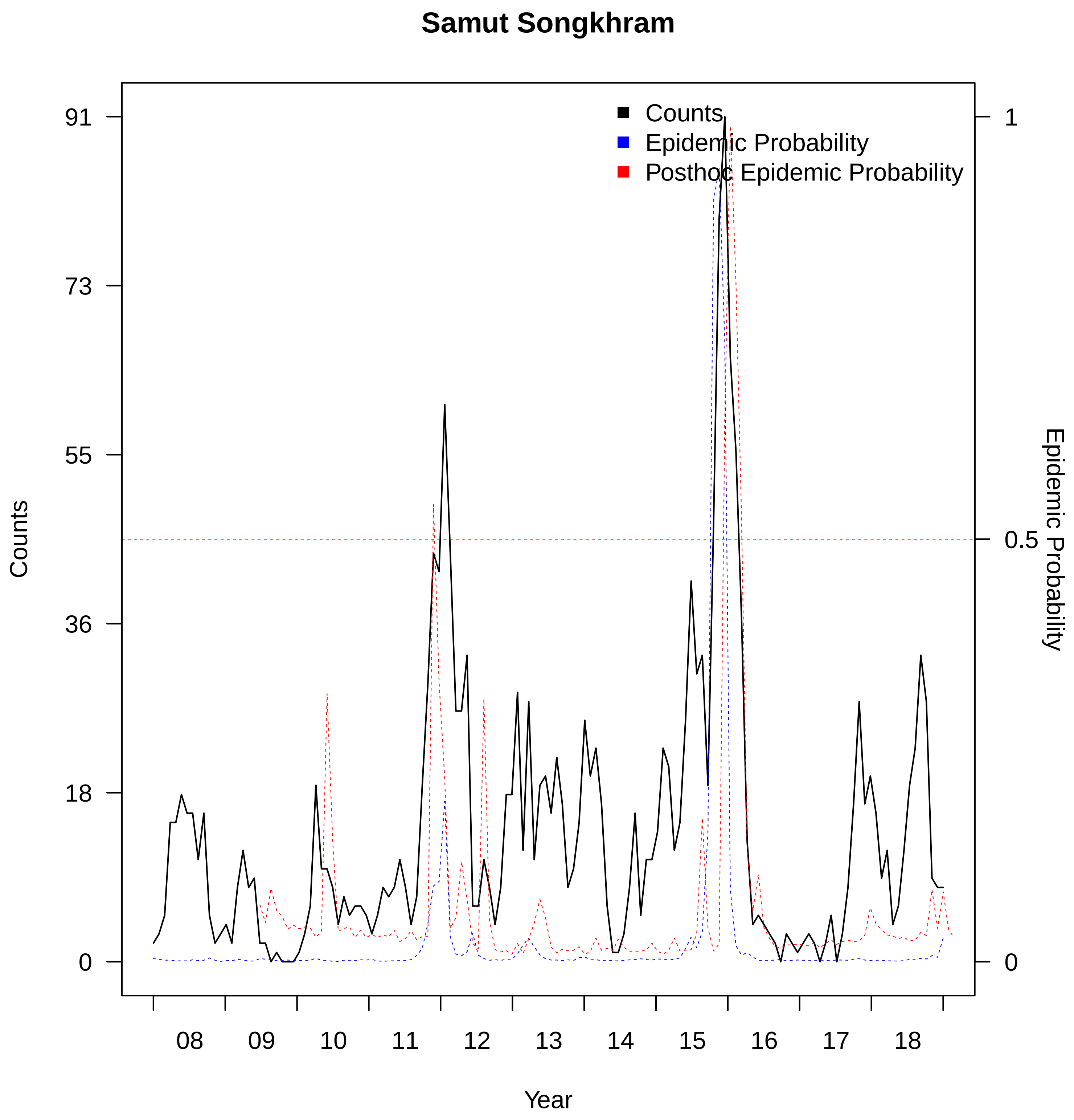

Samut Songkhram
Counts
Epidemic Probability
Posthoc Epidemic Probability
91
73
55
36
18
0
1
0.5
0
08
09
10
11
12
13
14
15
16
17
18
Year
Counts
Epidemic Probability

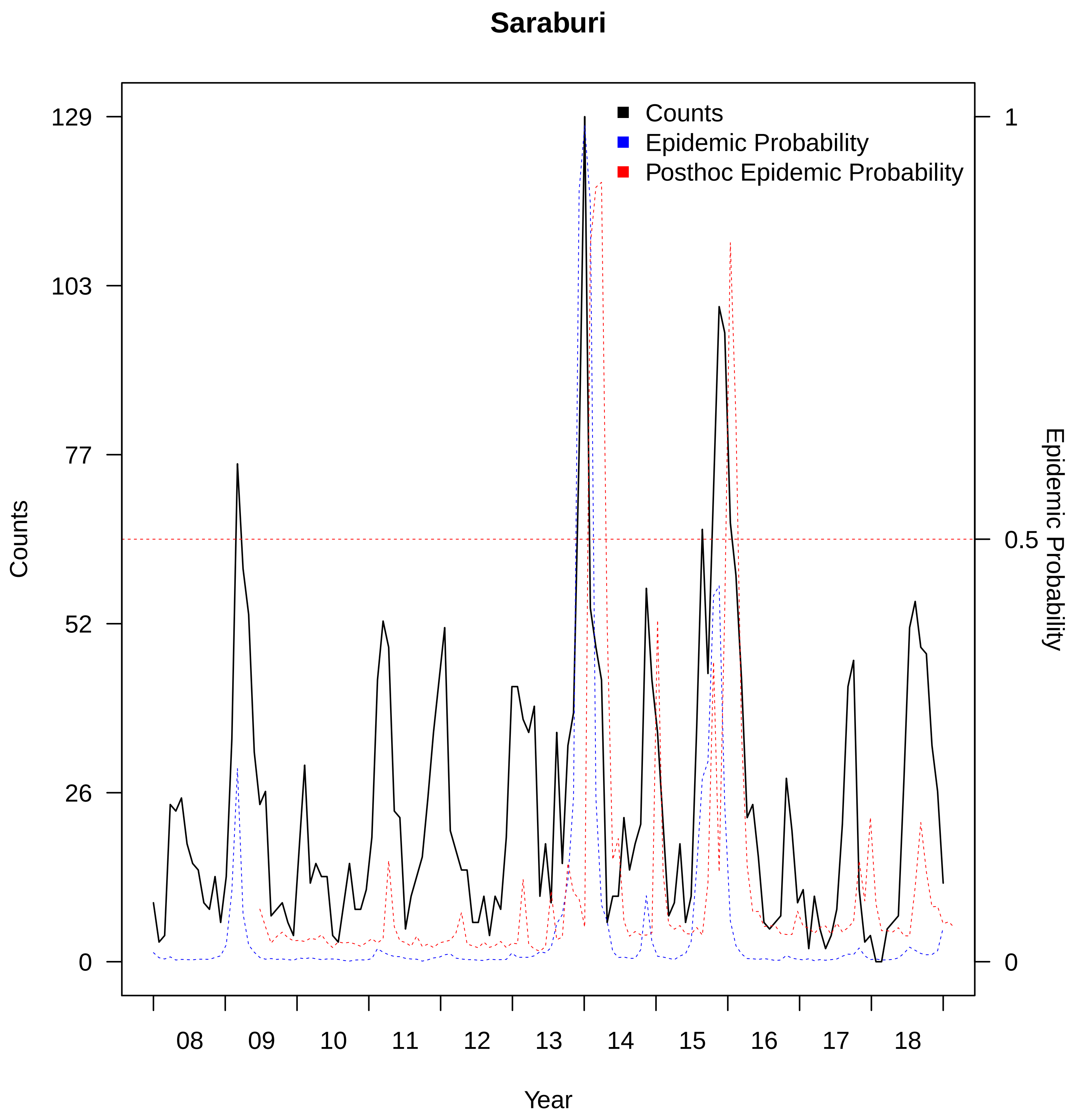

Saraburi
Counts
Epidemic Probability
Posthoc Epidemic Probability
129
103
77
52
26
0
1
0.5
0
Counts
Epidemic Probability
08
09
10
11
12
13
14
15
16
17
18
Year

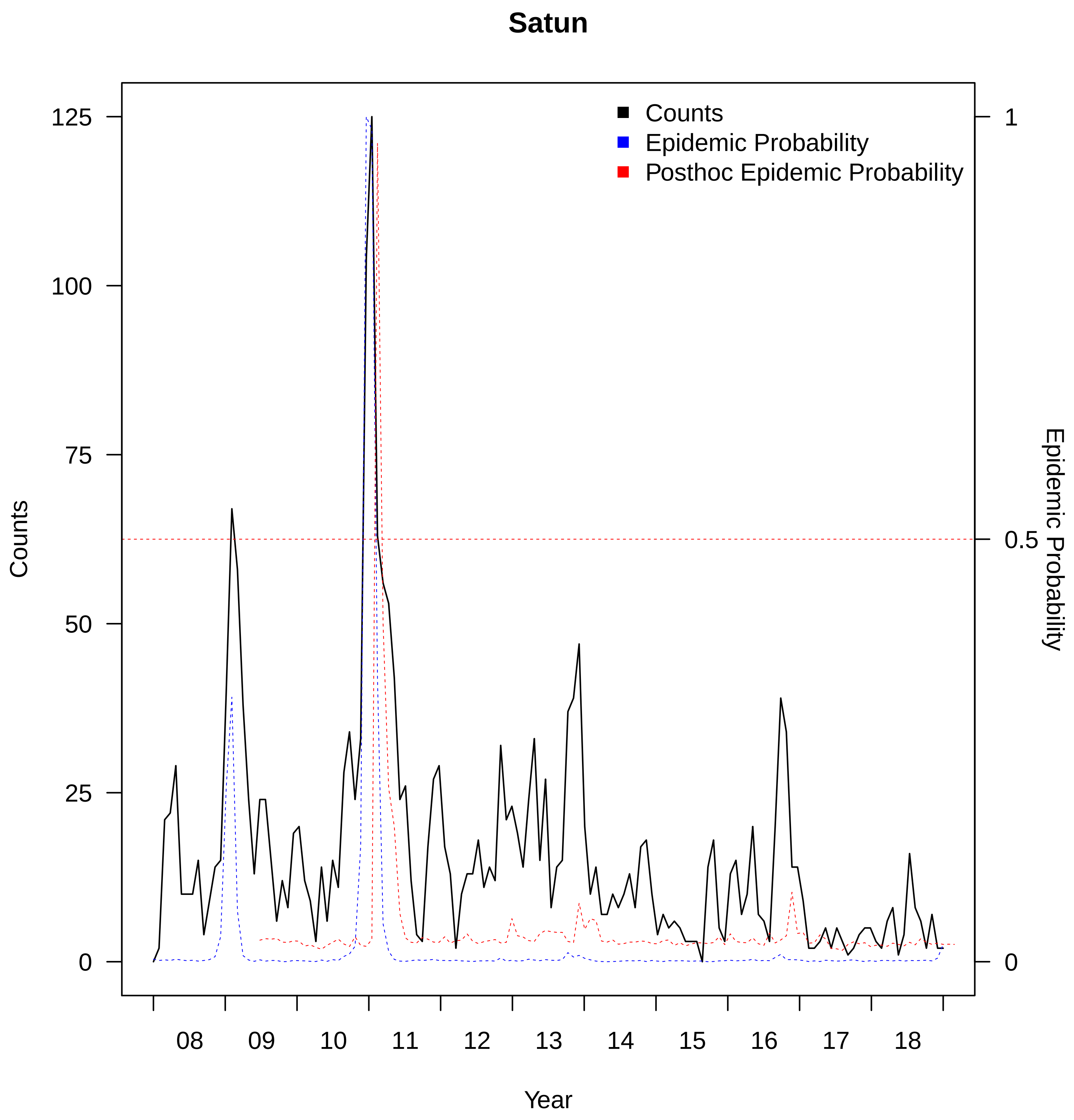
Satun
Counts
Epidemic Probability
Posthoc Epidemic Probability
125
100
75
50
25
0
1
0.5
0
08
09
10
11
12
13
14
15
16
17
18
Year
Counts
Epidemic Probability

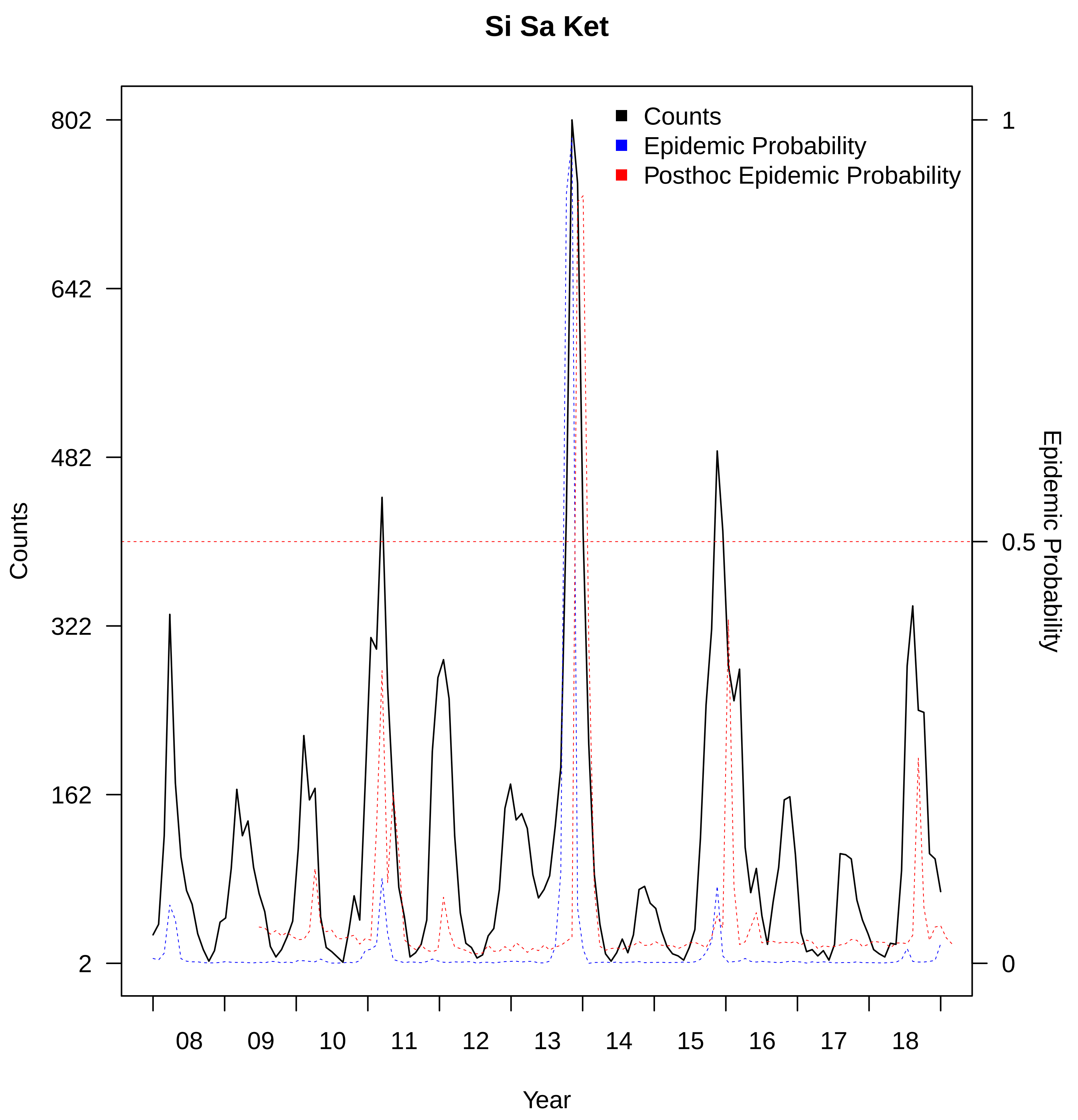

Si Sa Ket
Counts
Epidemic Probability
Posthoc Epidemic Probability
802
642
482
322
162
2
1
0.5
0
08
09
10
11
12
13
14
15
16
17
18
Year
Counts
Epidemic Probability

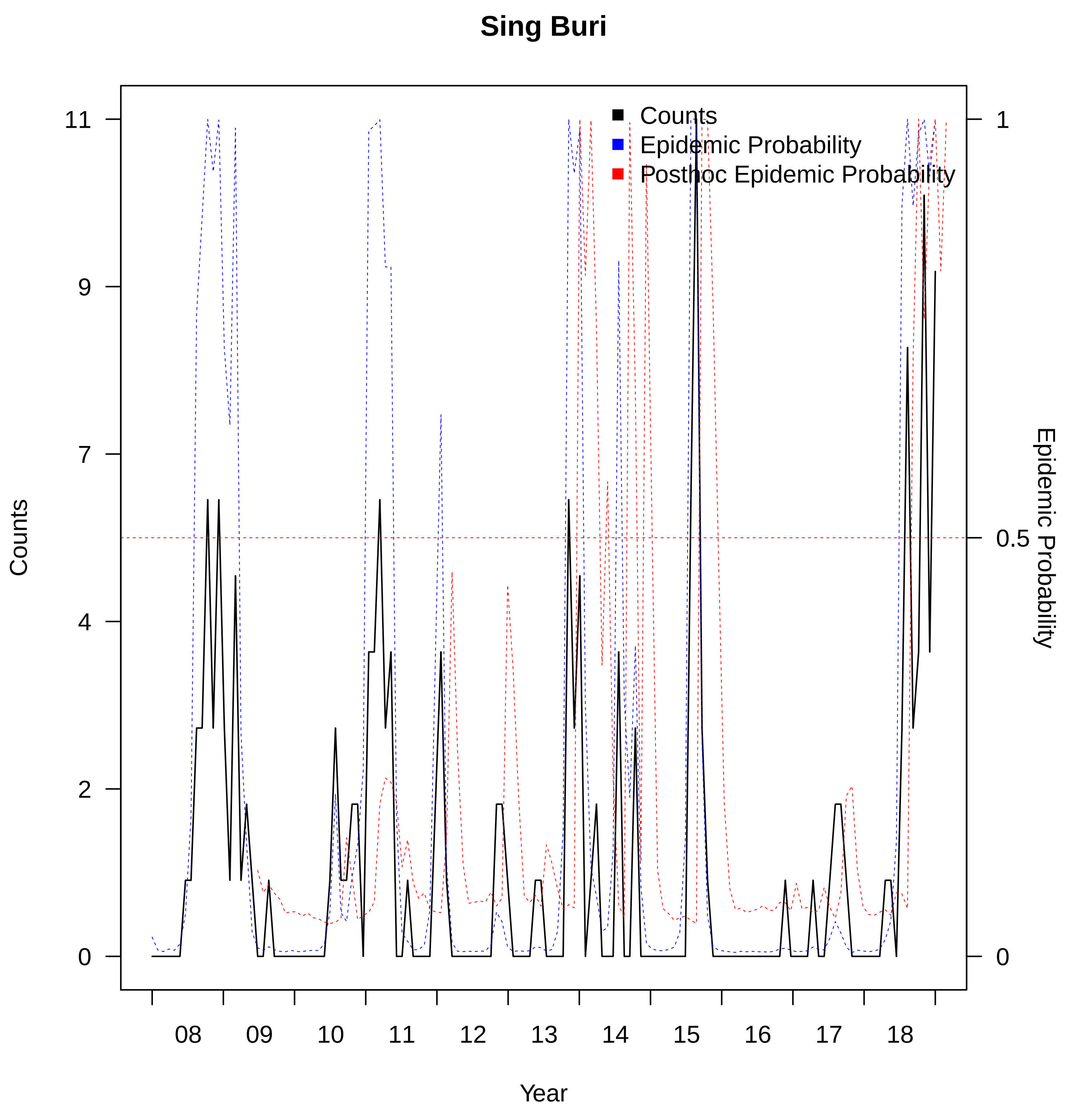
Sing Buri
Counts
Epidemic Probability
Posthoc Epidemic Probability
11
9
7
4
2
0
1
0.5
0
08
09
10
11
12
13
14
15
16
17
18
Counts
Year
Epidemic Probability

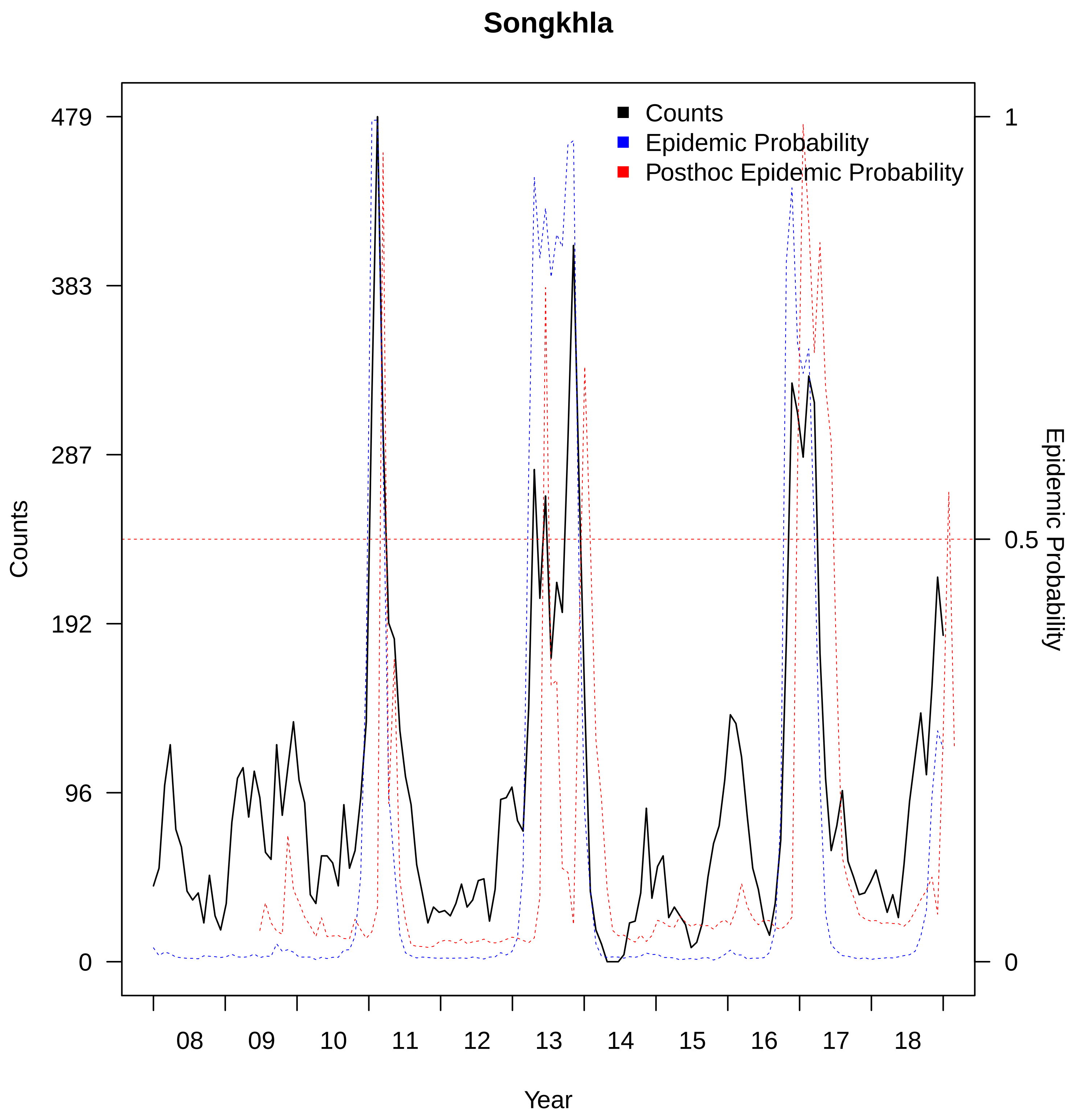

Songkhla
Counts
Epidemic Probability
Posthoc Epidemic Probability
479
383
287
192
96
0
1
0.5
0
08
09
10
11
12
13
14
15
16
17
18
Year

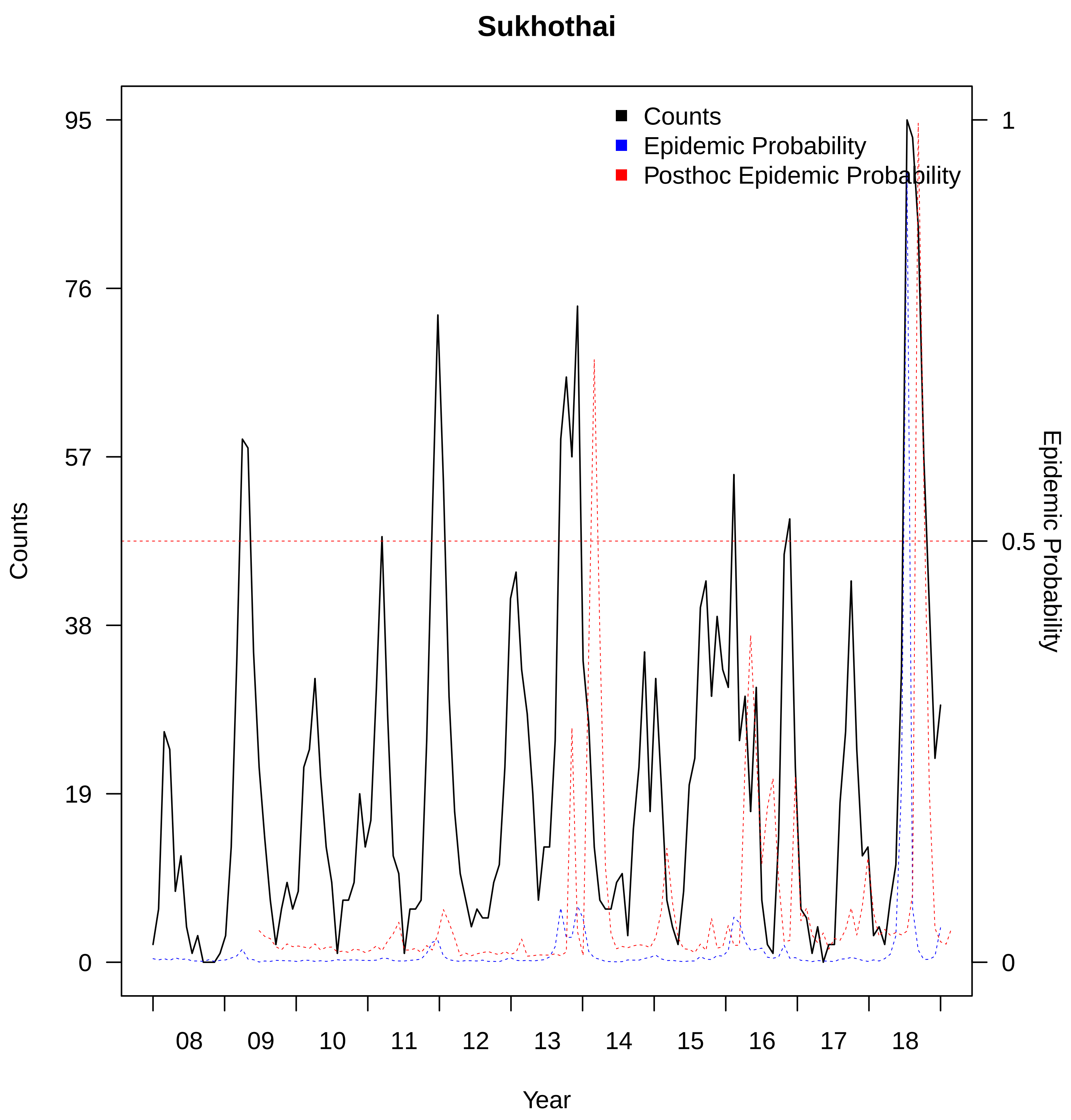

Sukhothai
Counts
Epidemic Probability
Posthoc Epidemic Probability
95
76
57
38
19
0
1
0.5
0
Counts
Epidemic Probability
08
09
10
11
12
13
14
15
16
17
18
Year

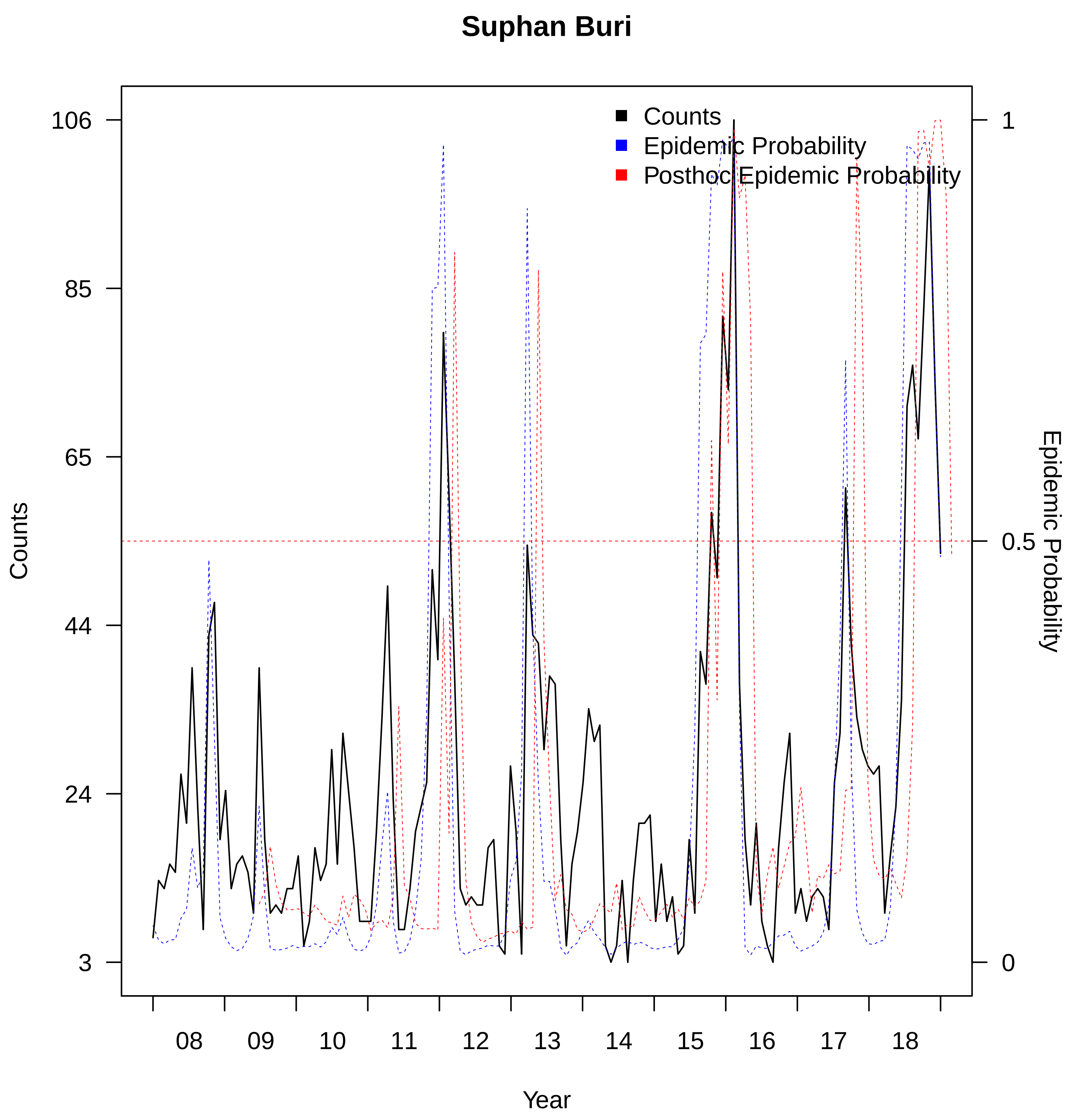

Suphan Buri
Counts
Epidemic Probability
Posthoc Epidemic Probability
106
85
65
44
24
3
1
0.5
0
08
09
10
11
12
13
14
15
16
17
18
Year

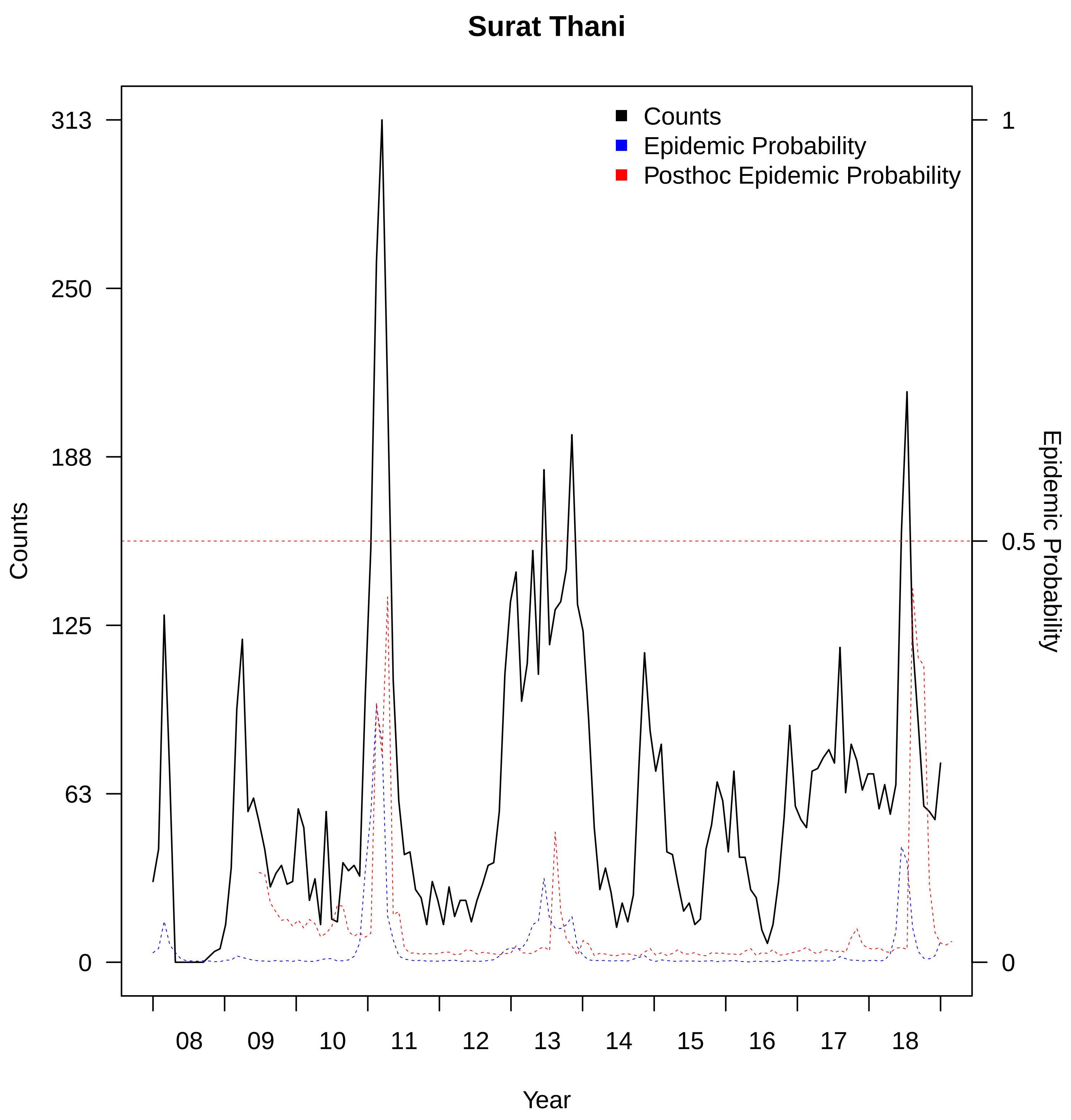

Surat Thani
Counts
Epidemic Probability
Posthoc Epidemic Probability
313
250
188
125
63
0
1
0.5
0
Counts
Epidemic Probability
08
09
10
11
12
13
14
15
16
17
18
Year

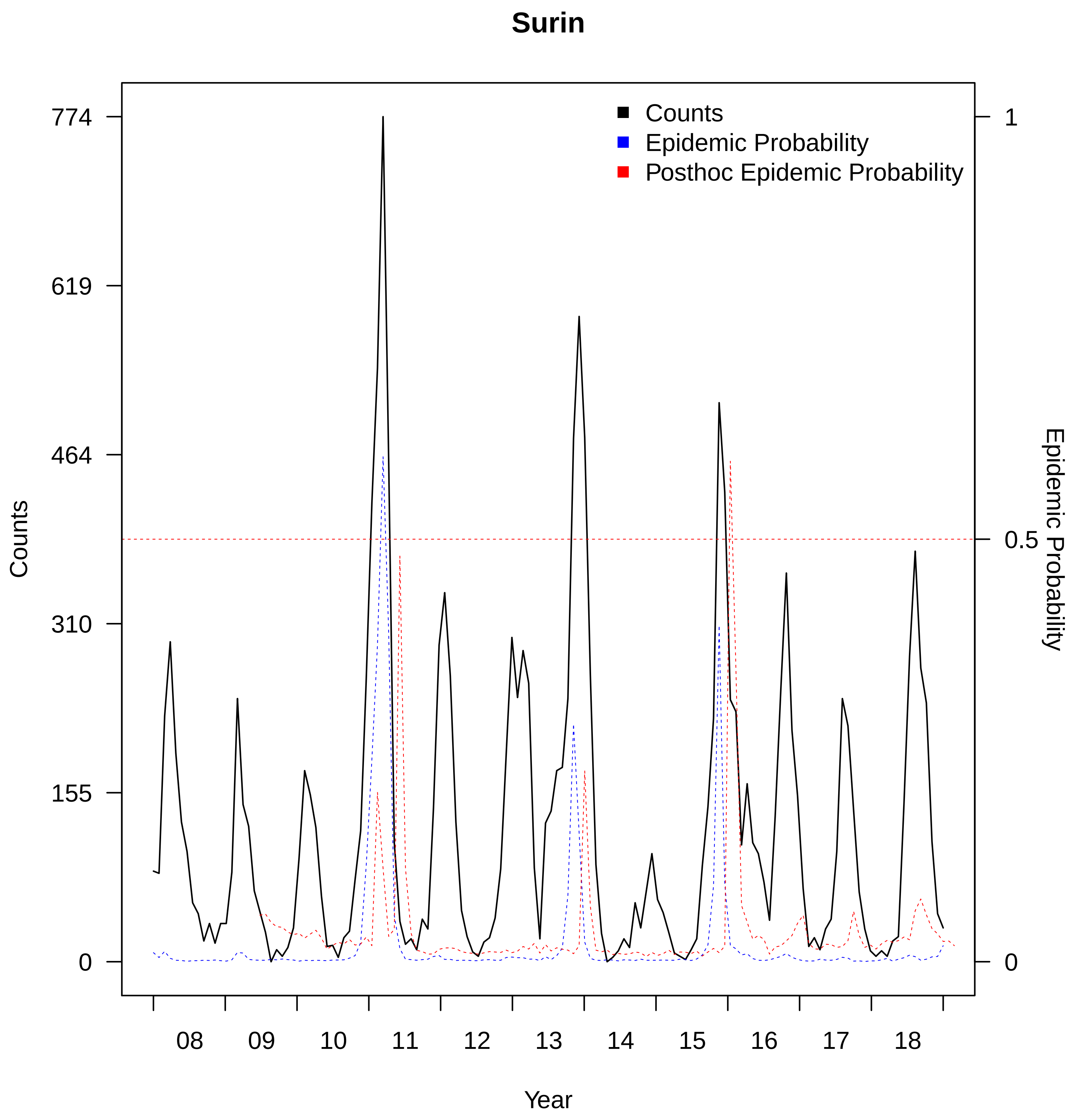
Surin
Counts
Epidemic Probability
Posthoc Epidemic Probability
774
619
464
310
155
0
1
0.5
0
Counts
Epidemic Probability
08
09
10
11
12
13
14
15
16
17
18
Year

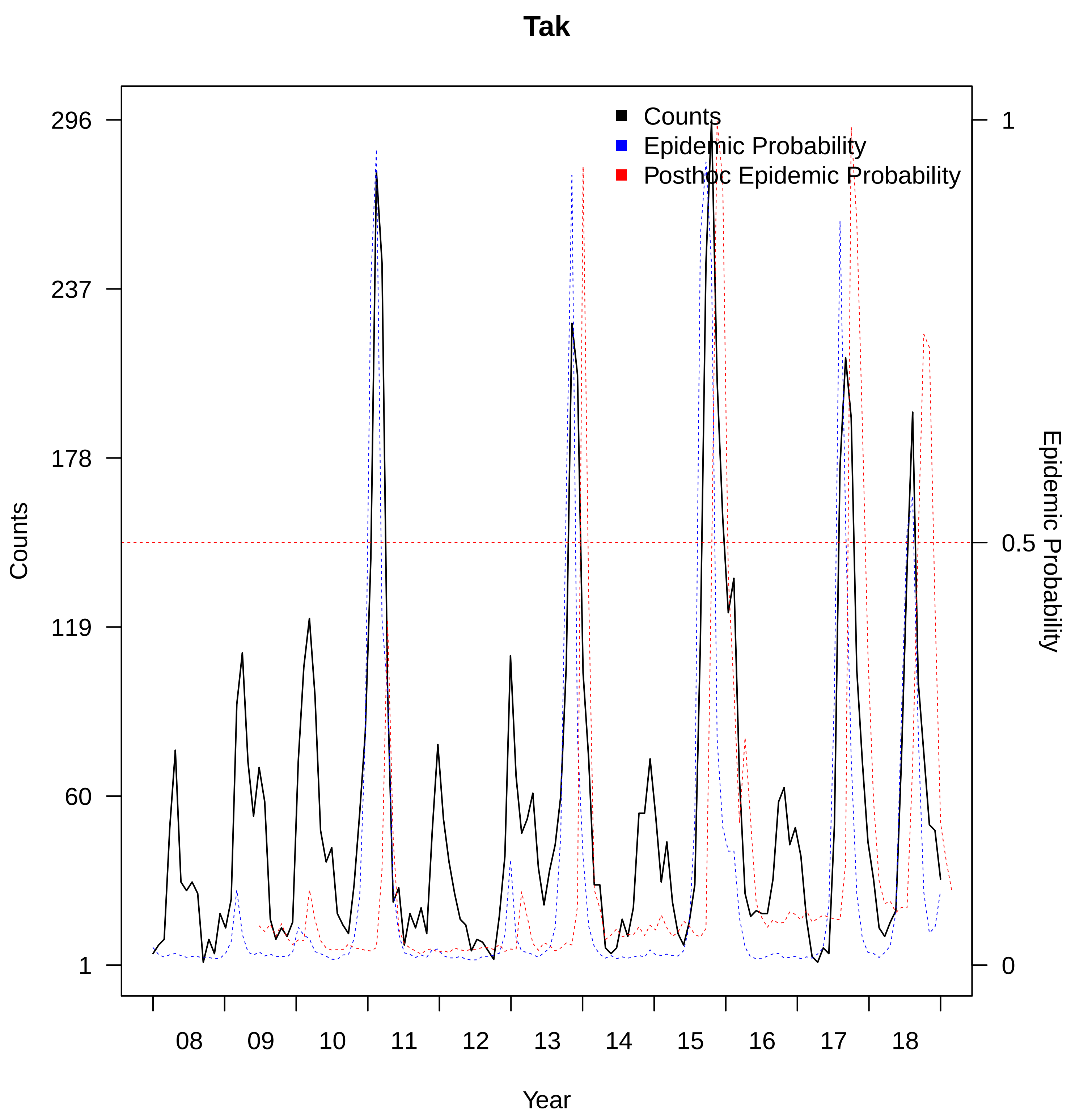

Tak
Counts
Epidemic Probability
Posthoc Epidemic Probability
296
237
178
119
60
1
1
0.5
0
08
09
10
11
12
13
14
15
16
17
18
Year
Counts
Epidemic Probability

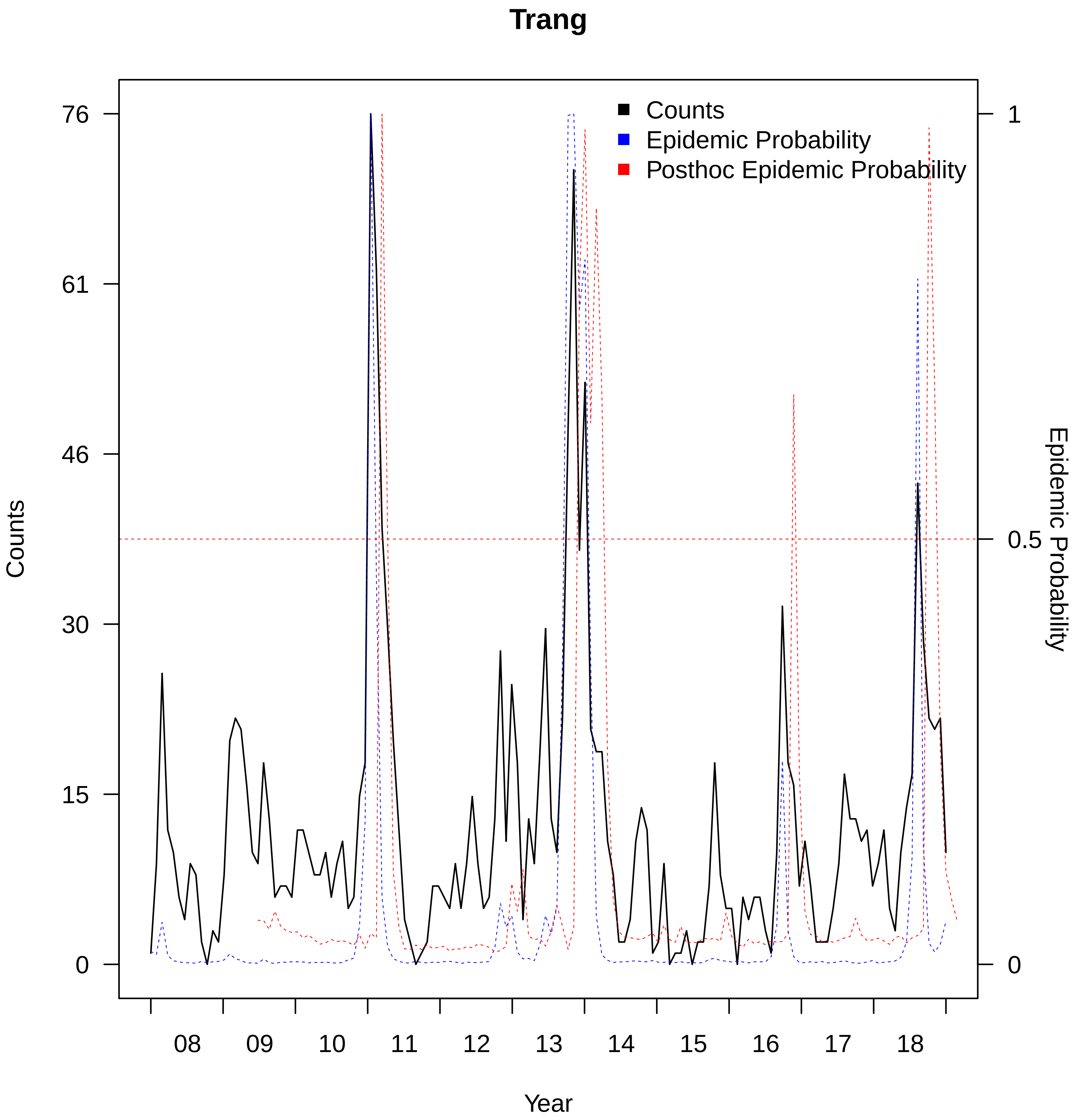
Trang
Counts
Epidemic Probability
Posthoc Epidemic Probability
76
61
46
30
15
0
1
0.5
0
Counts
Epidemic Probability
08
09
10
11
12
13
14
15
16
17
18
Year

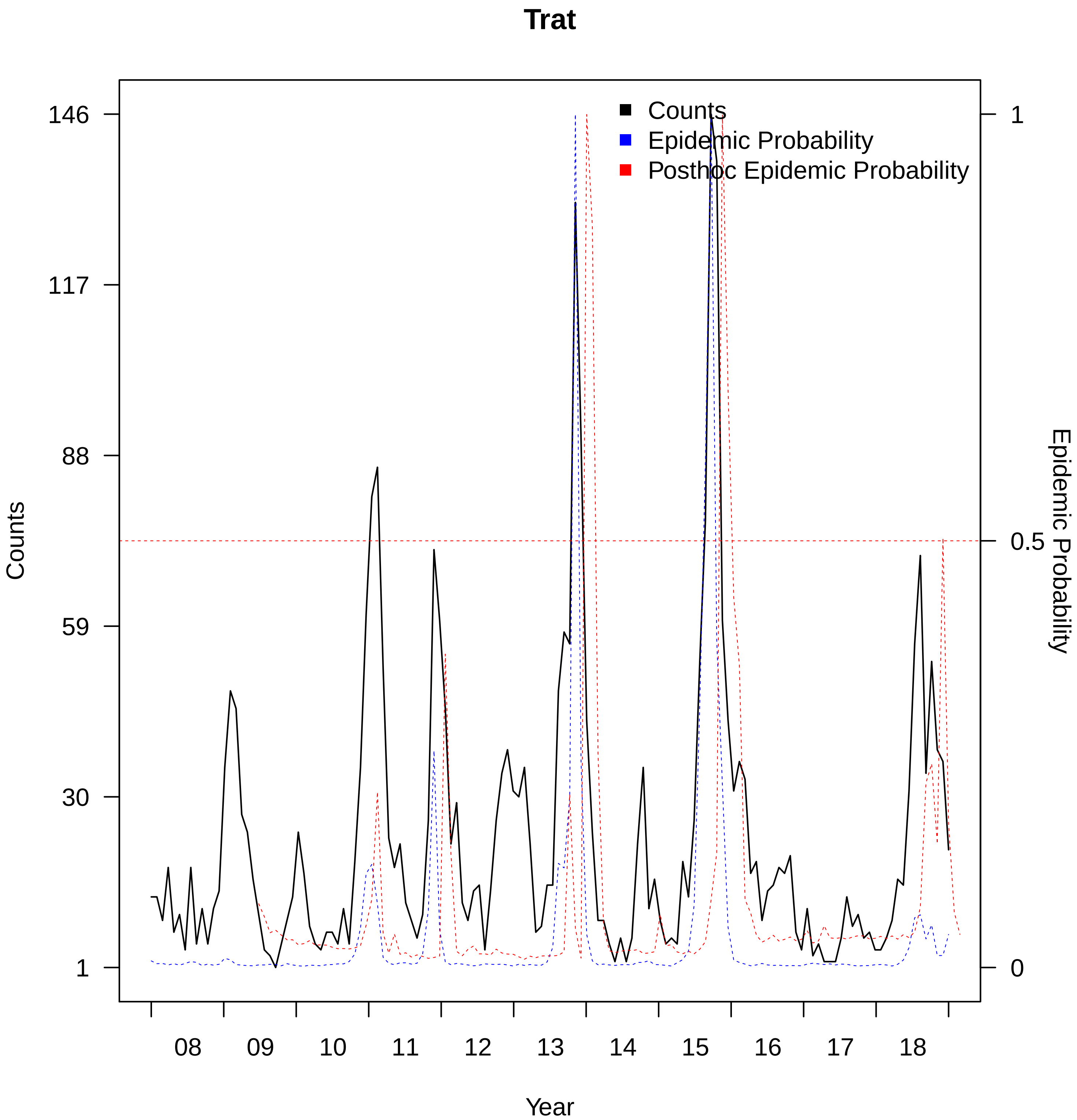

Trat
Counts
Epidemic Probability
Posthoc Epidemic Probability
146
117
88
59
30
1
Counts
1
0.5
0
Epidemic Probability
08
09
10
11
12
13
14
15
16
17
18
Year

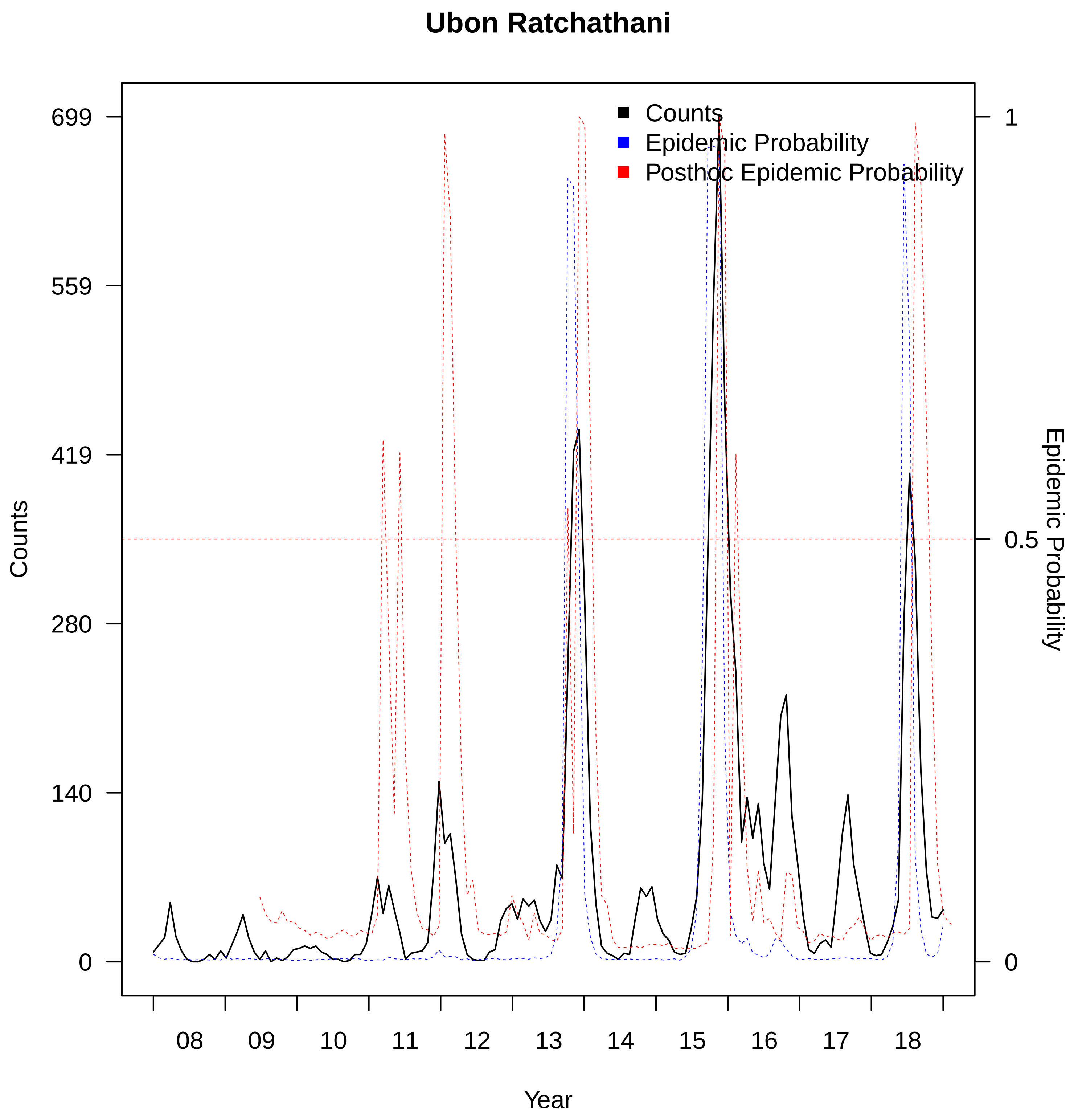

Ubon Ratchathani
Counts
Epidemic Probability
Posthoc Epidemic Probability
699
559
419
280
140
0
1
0.5
0
Counts
Epidemic Probability
08
09
10
11
12
13
14
15
16
17
18
Year

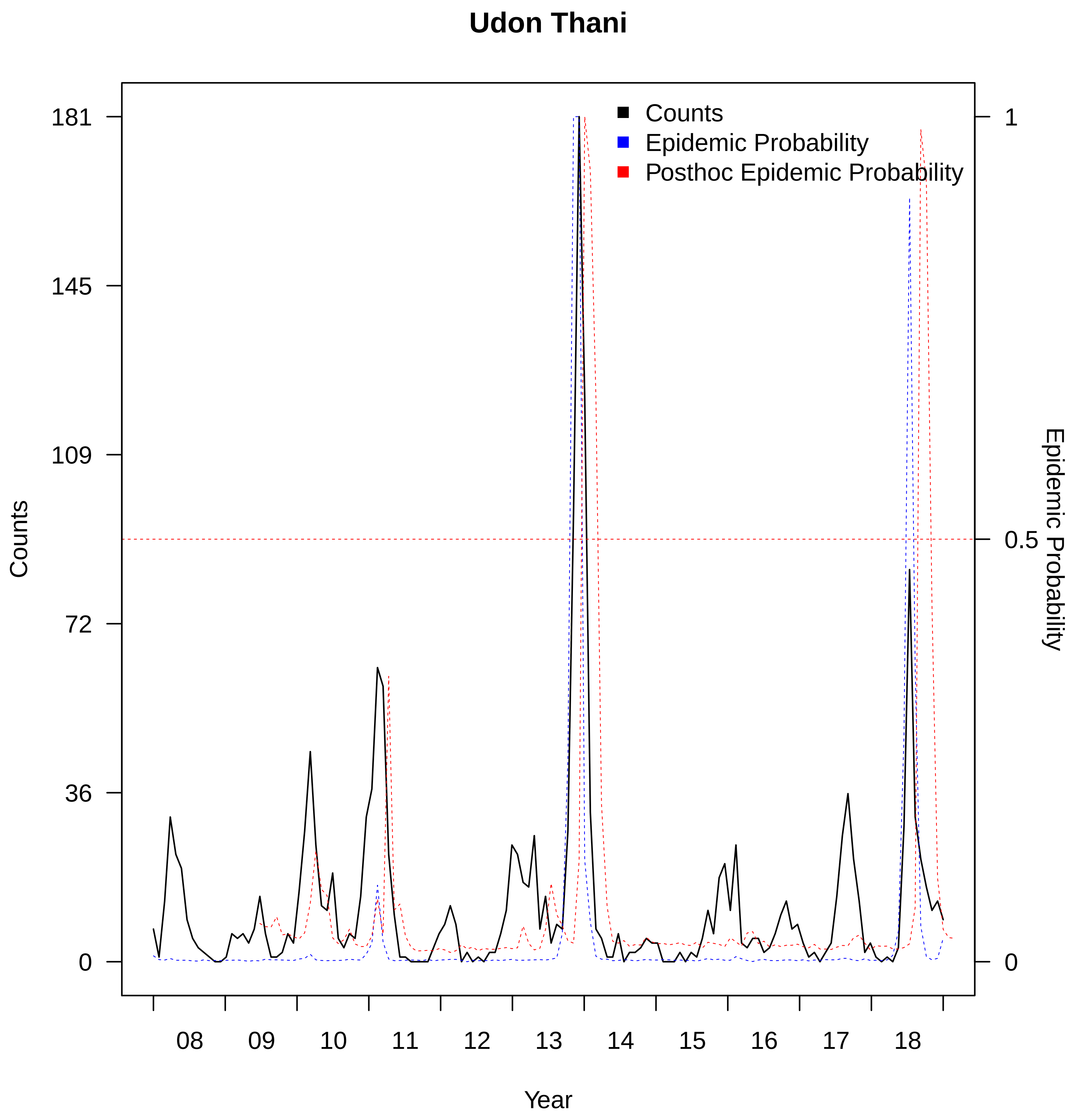
Udon Thani
Counts
Epidemic Probability
Posthoc Epidemic Probability
181
145
109
72
36
0
1
0.5
0
Counts
Epidemic Probability
08
09
10
11
12
13
14
15
16
17
18
Year

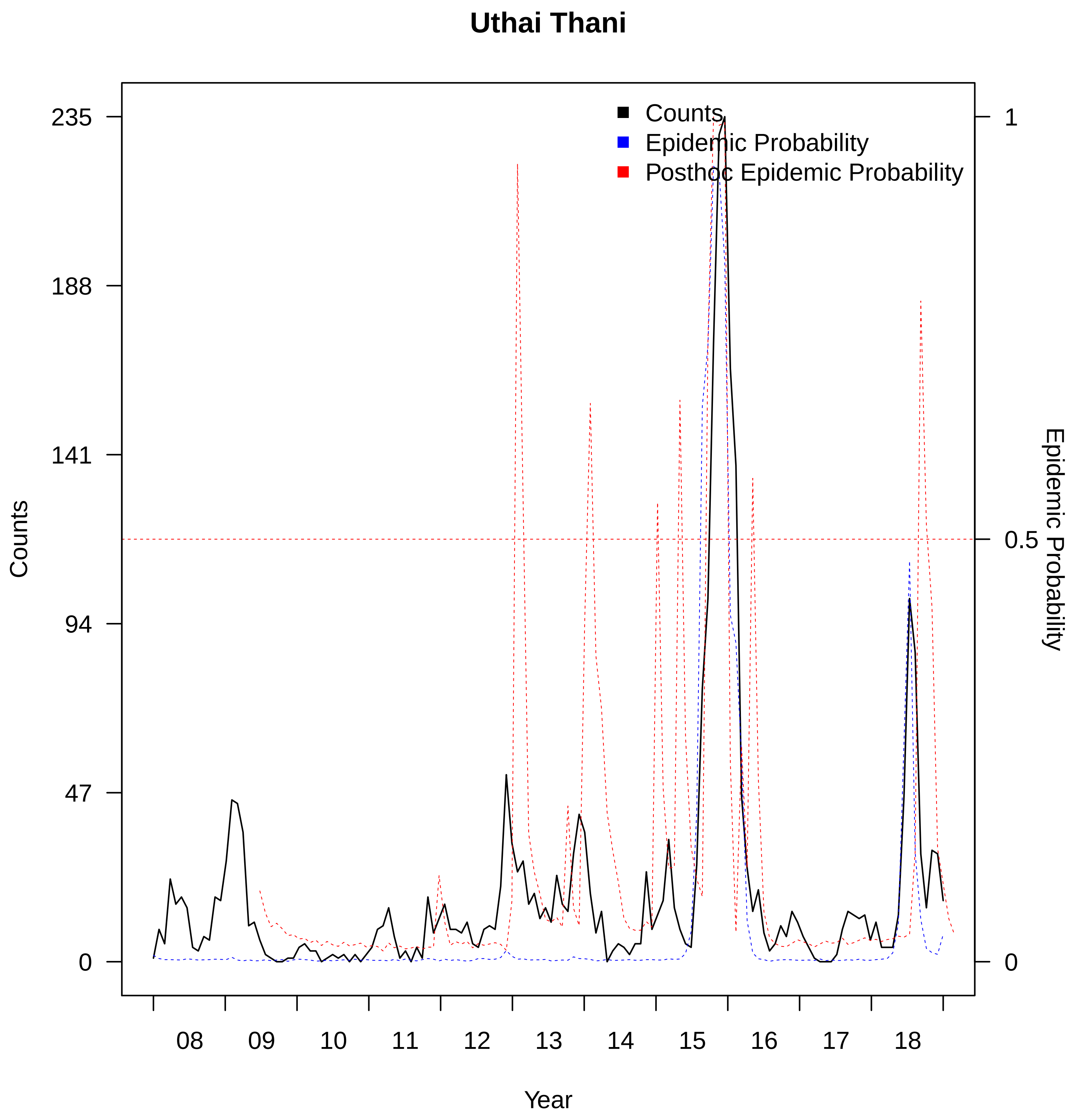
Uthai Thani
Counts
Epidemic Probability
Posthoc Epidemic Probability
235
188
141
94
47
0
1
0.5
0
Counts
Epidemic Probability
08
09
10
11
12
13
14
15
16
17
18
Year

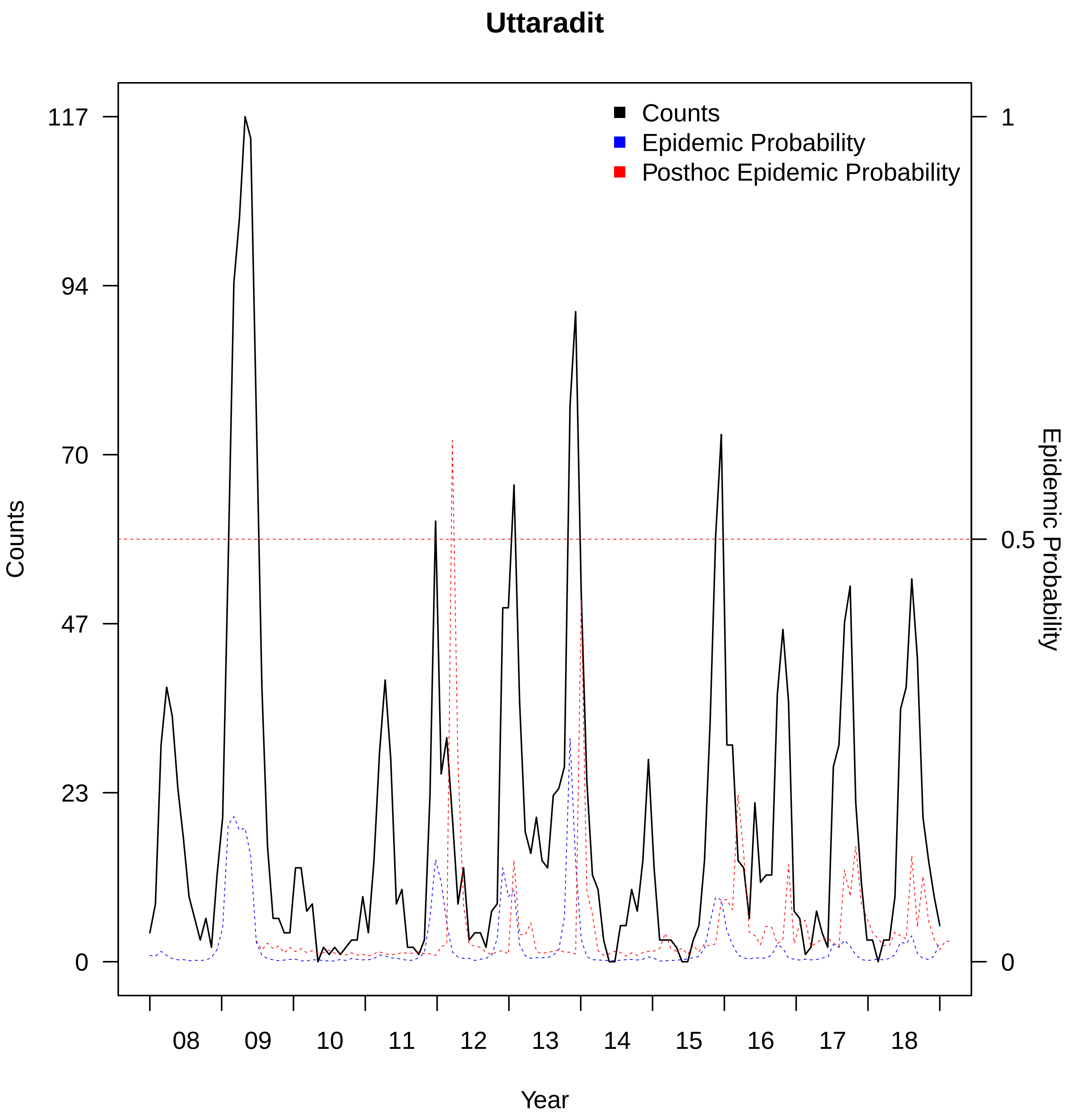
Uttaradit
Counts
Epidemic Probability
Posthoc Epidemic Probability
117
94
70
47
23
0
Counts
1
0.5
0
Epidemic Probability
08
09
10
11
12
13
14
15
16
17
18
Year

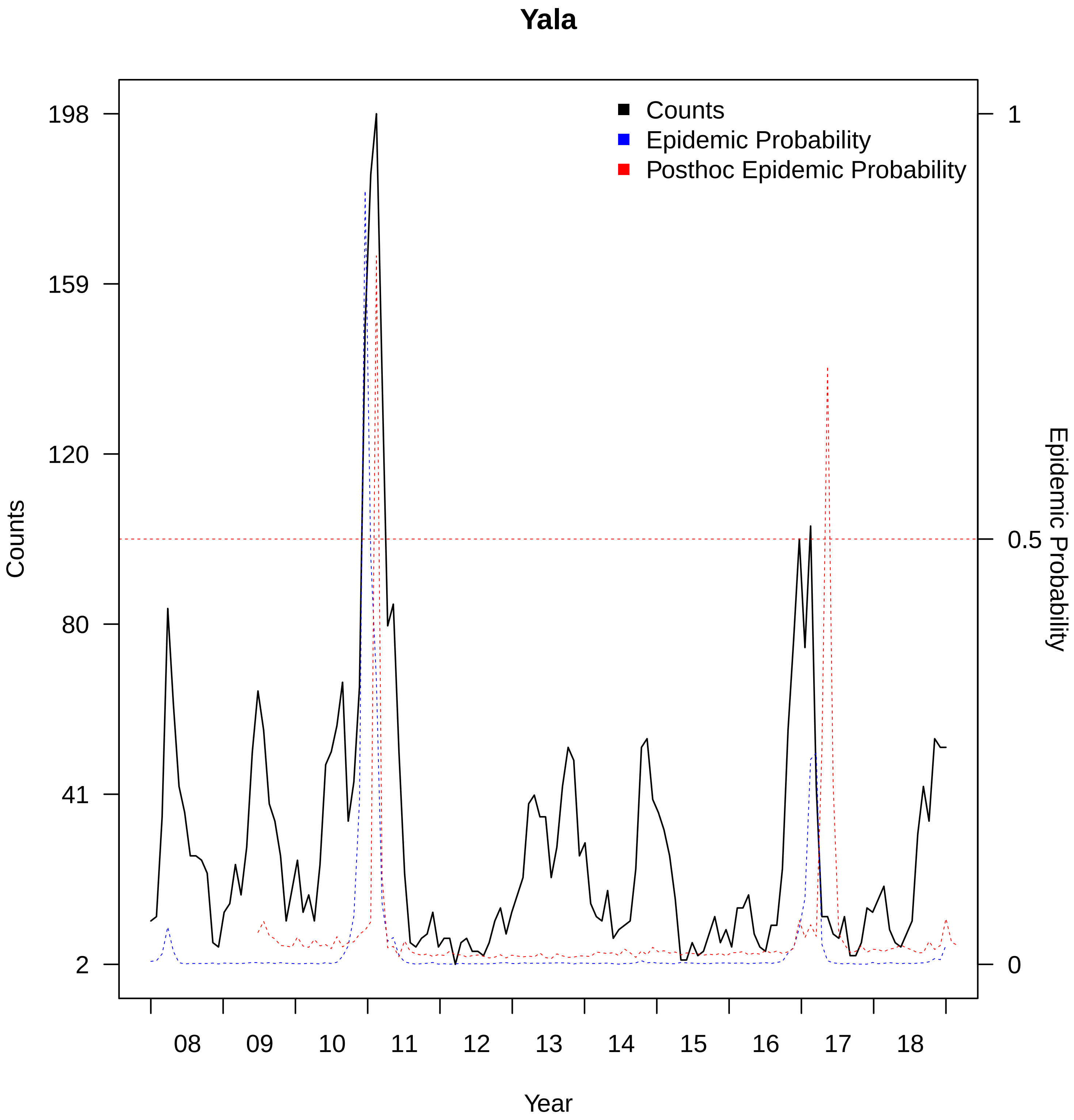
Yala
Counts
Epidemic Probability
Posthoc Epidemic Probability
198
159
120
80
41
2
1
0.5
0
Counts
Epidemic Probability
08
09
10
11
12
13
14
15
16
17
18
Year

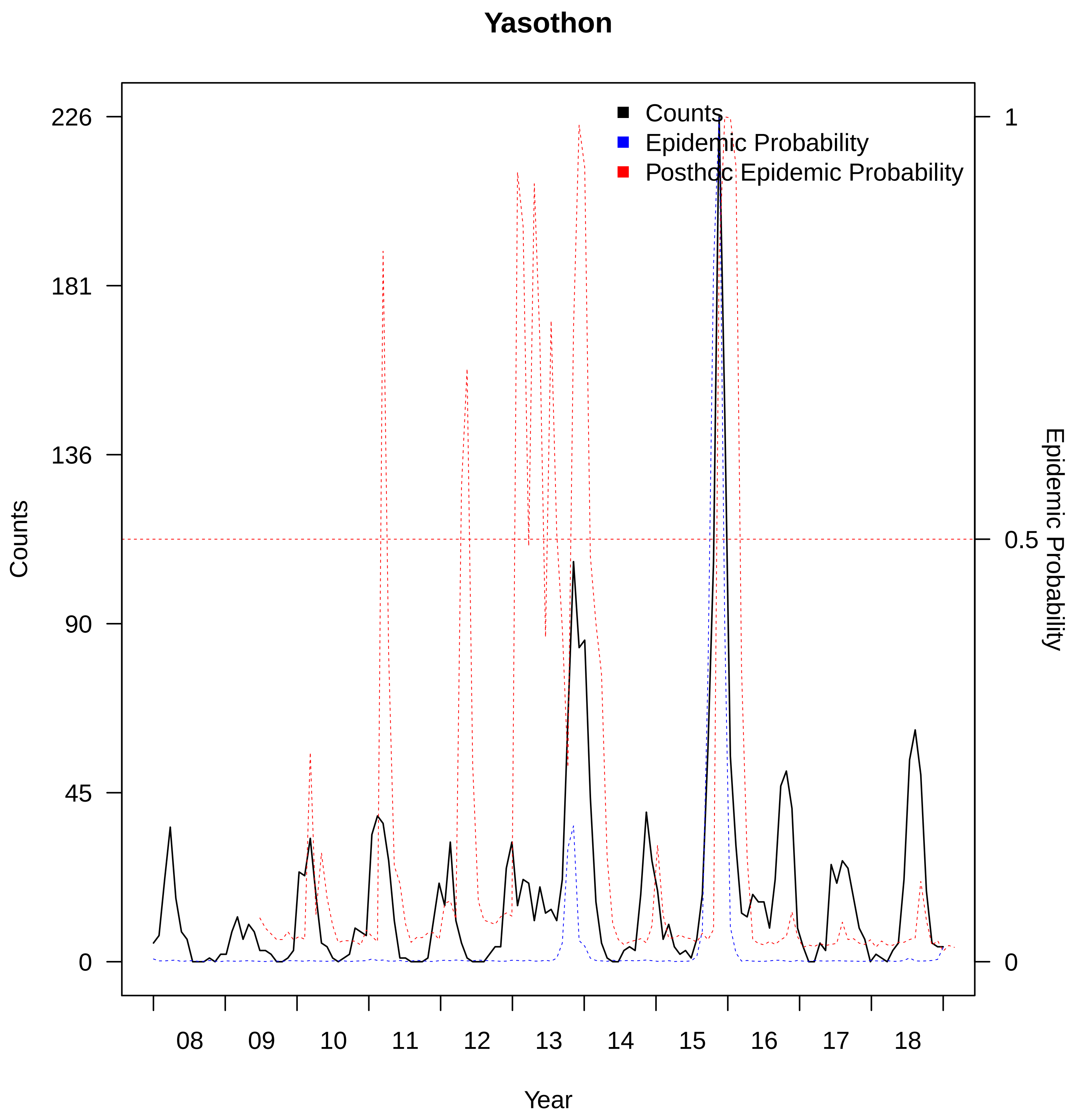

Yasothon
Counts
Epidemic Probability
Posthoc Epidemic Probability
226
181
136
90
45
0
1
0.5
0
Counts
Epidemic Probability
08
09
10
11
12
13
14
15
16
17
18
Year

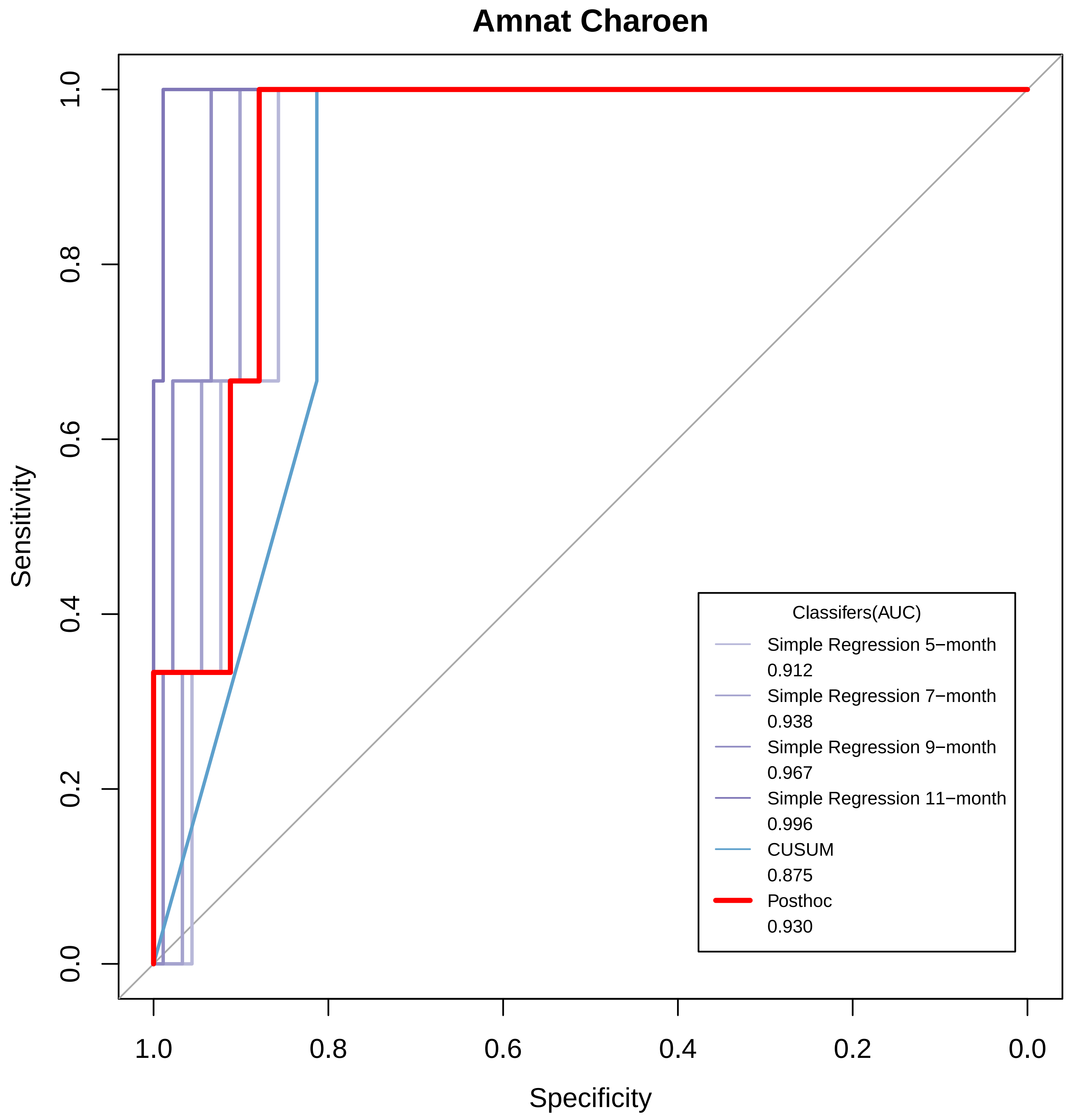
Amnat Charoen
Sensitivity
Specificity
1.0
0.8
0.6
0.4
0.2
0.0
Classifers(AUC)
Simple Regression 5−month
0.912
Simple Regression 7−month
0.938
Simple Regression 9−month
0.967
Simple Regression 11−month
0.996
CUSUM
0.875
Posthoc
0.930

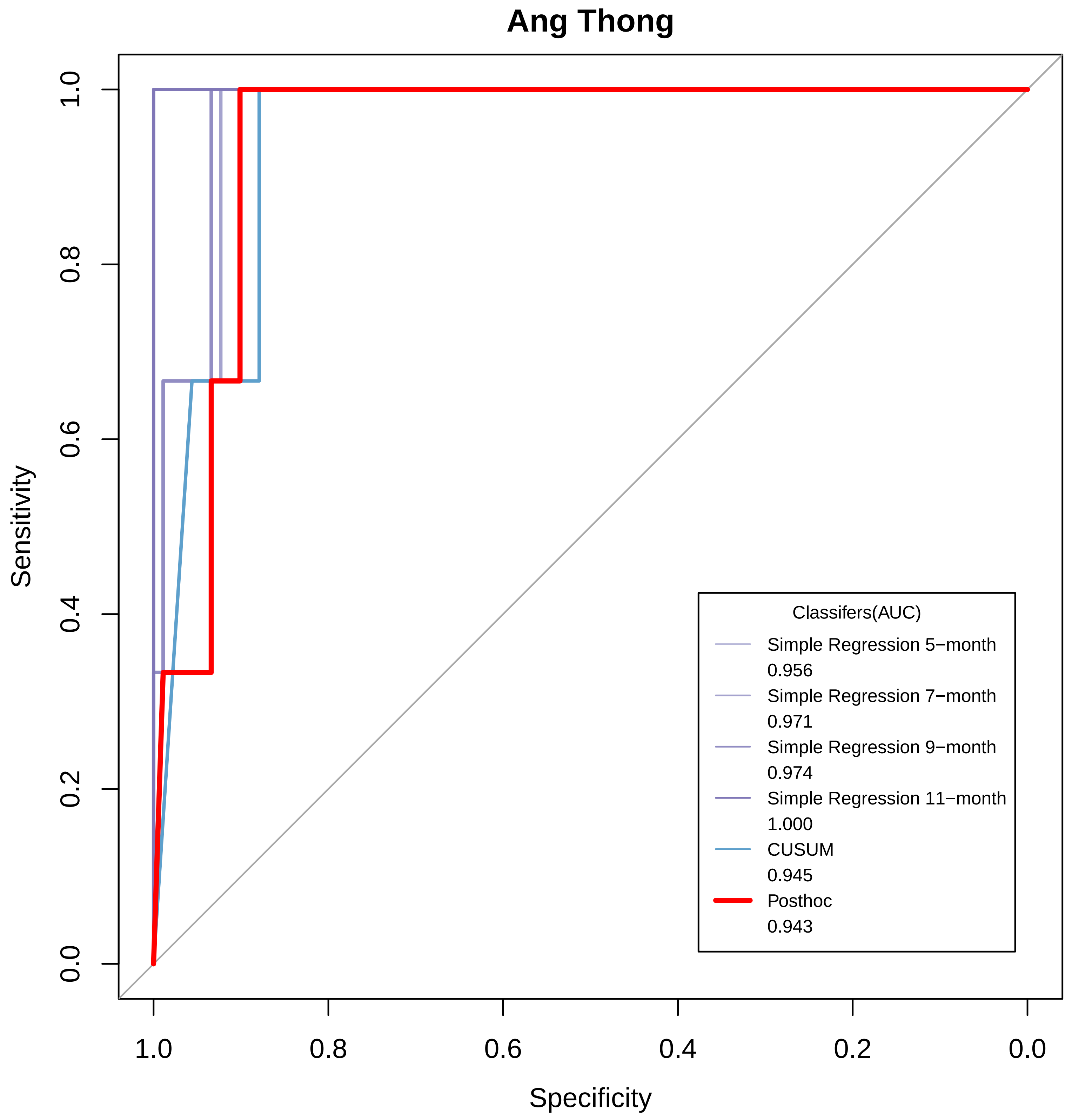
Ang Thong
Sensitivity
Specificity
1.0
0.8
0.6
0.4
0.2
0.0
Classifers(AUC)
Simple Regression 5−month
0.956
Simple Regression 7−month
0.971
Simple Regression 9−month
0.974
Simple Regression 11−month
1.000
CUSUM
0.945
Posthoc
0.943

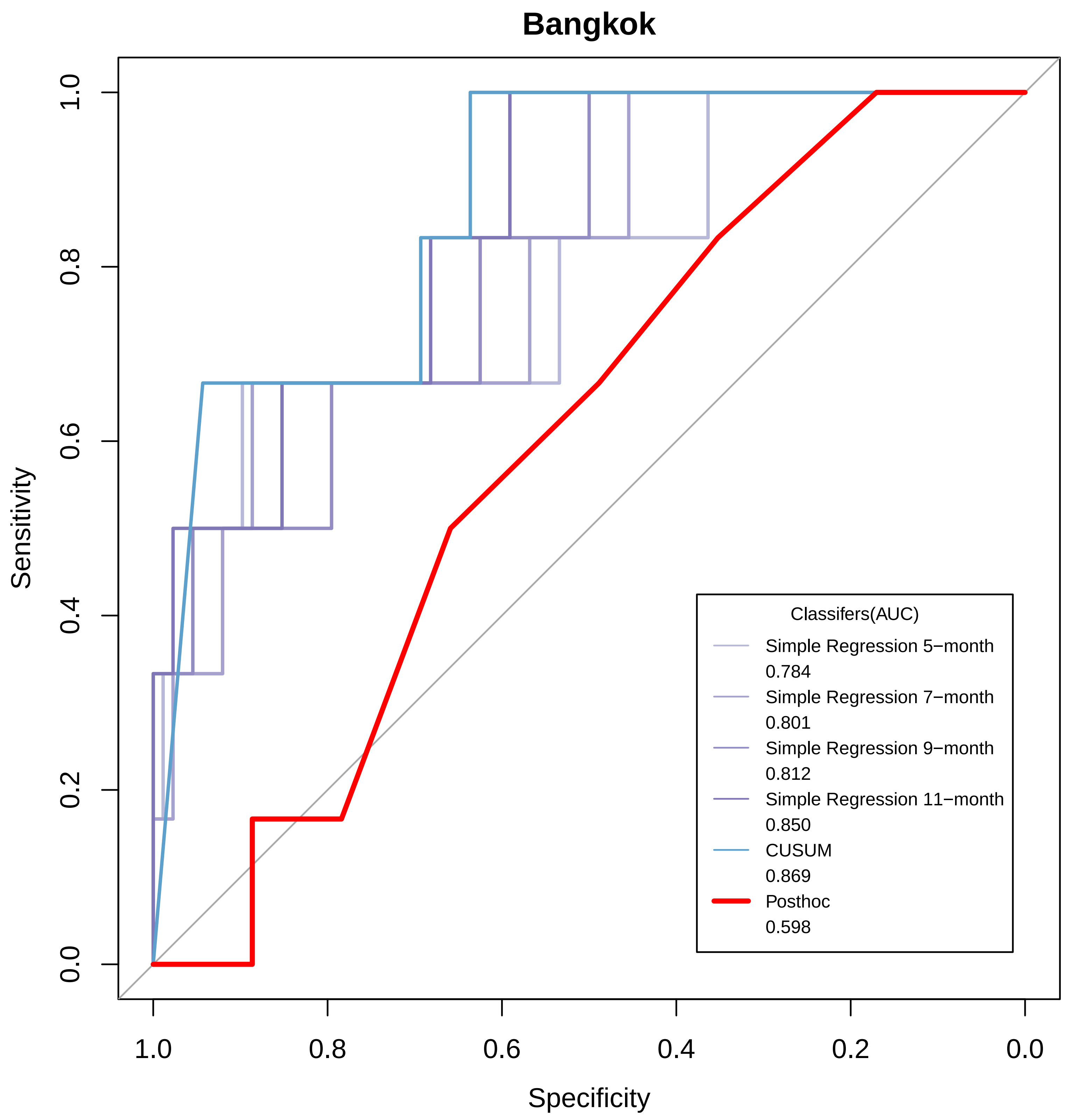

Bangkok
Sensitivity
Specificity
0.0
0.2
0.4
0.6
0.8
1.0
1.0
0.8
0.6
0.4
0.2
0.0
Classifers(AUC)
Simple Regression 5−month
0.784
Simple Regression 7−month
0.801
Simple Regression 9−month
0.812
Simple Regression 11−month
0.850
CUSUM
0.869
Posthoc
0.598

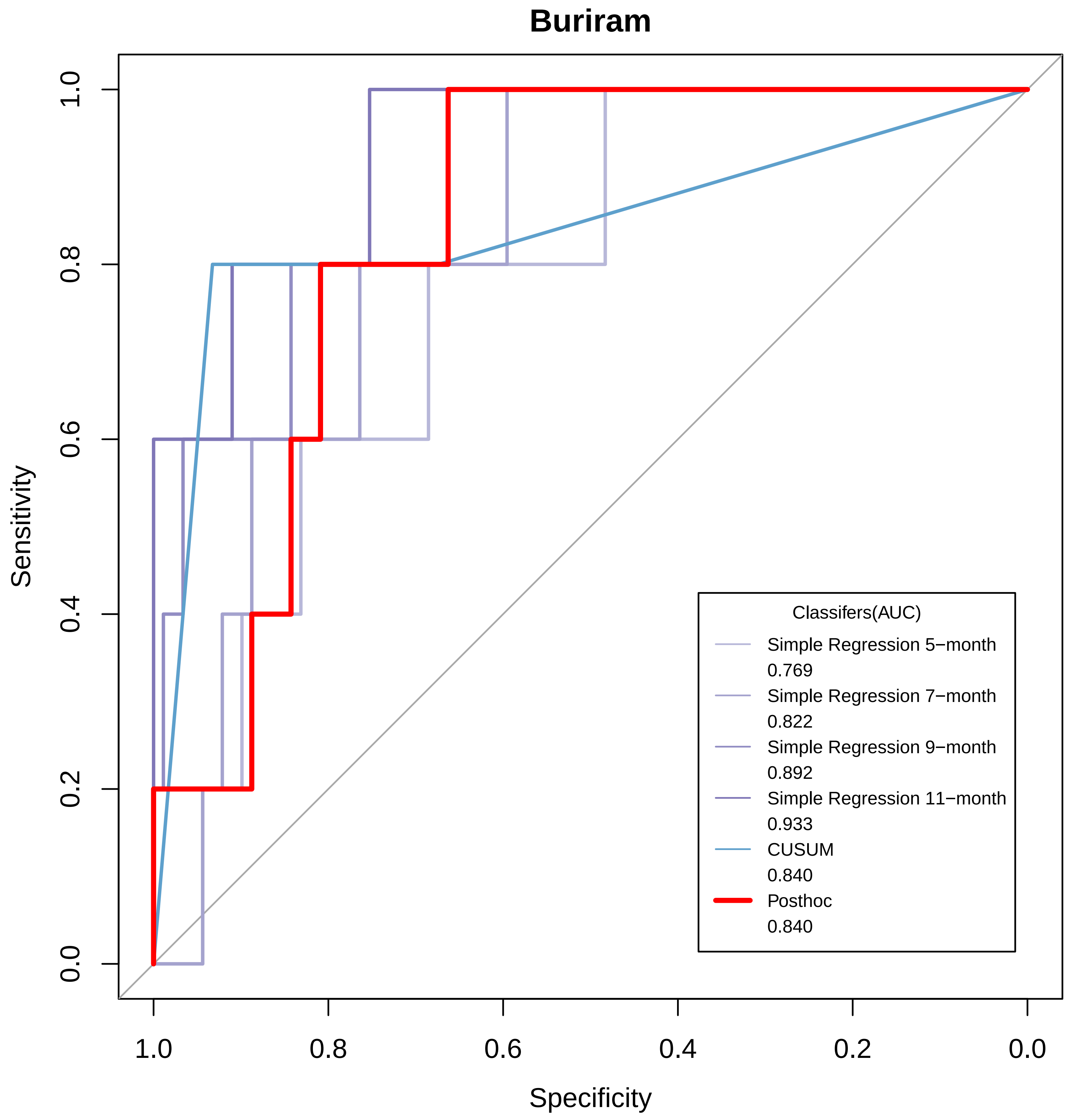

Buriram
Sensitivity
Specificity
1.0
0.8
0.6
0.4
0.2
0.0
Classifers(AUC)
Simple Regression 5−month
0.769
Simple Regression 7−month
0.822
Simple Regression 9−month
0.892
Simple Regression 11−month
0.933
CUSUM
0.840
Posthoc
0.840

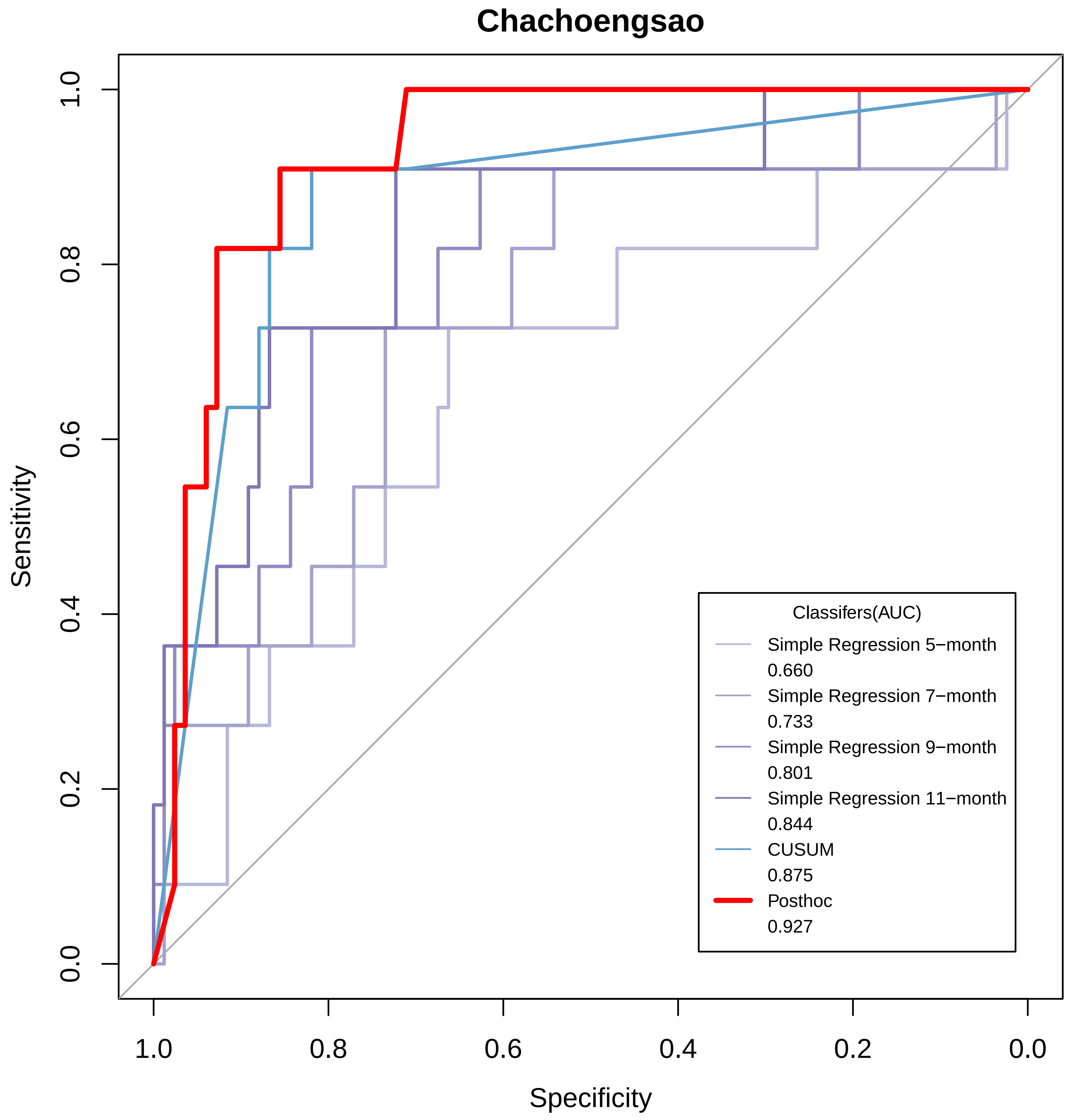
Chachoengsao
Sensitivity
Specificity
1.0
0.8
0.6
0.4
0.2
0.0
Classifers(AUC)
Simple Regression 5−month
0.660
Simple Regression 7−month
0.733
Simple Regression 9−month
0.801
Simple Regression 11−month
0.844
CUSUM
0.875
Posthoc
0.927

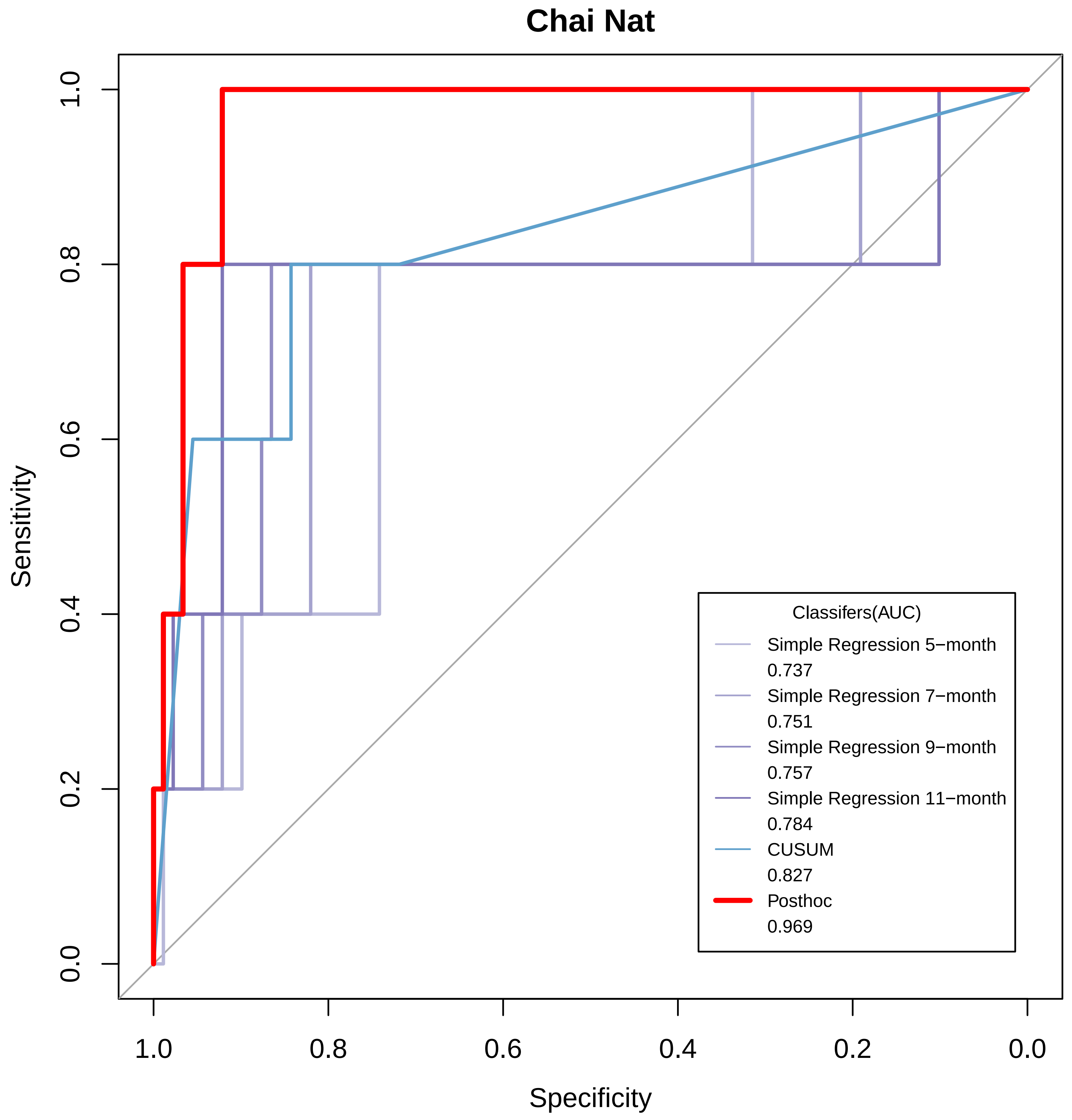
Chai Nat
Sensitivity
Specificity
1.0
0.8
0.6
0.4
0.2
0.0
Classifers(AUC)
Simple Regression 5−month
0.737
Simple Regression 7−month
0.751
Simple Regression 9−month
0.757
Simple Regression 11−month
0.784
CUSUM
0.827
Posthoc
0.969

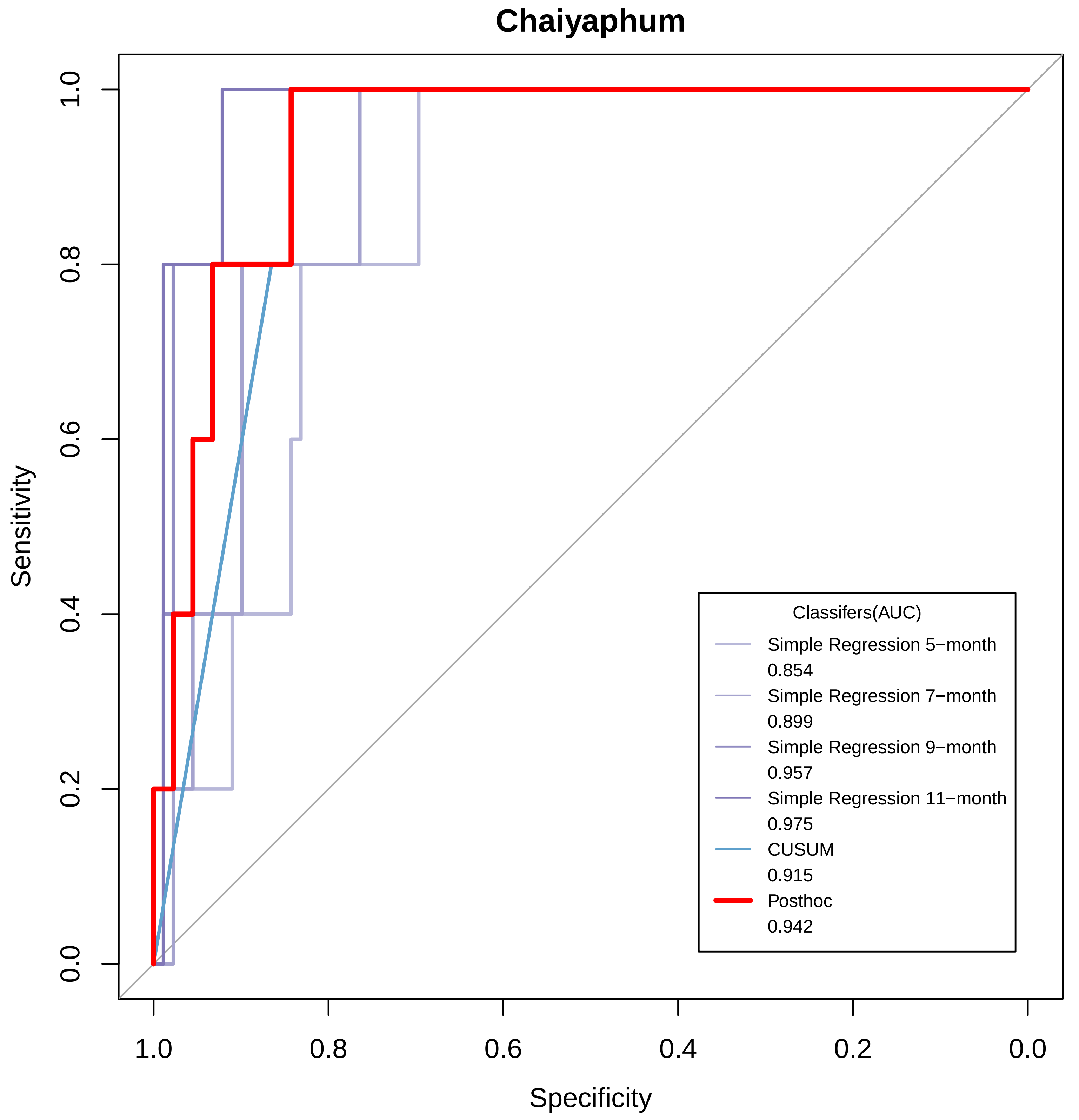

Chaiyaphum
Sensitivity
Specificity
1.0
0.8
0.6
0.4
0.2
0.0
Classifers(AUC)
Simple Regression 5−month
0.854
Simple Regression 7−month
0.899
Simple Regression 9−month
0.957
Simple Regression 11−month
0.975
CUSUM
0.915
Posthoc
0.942

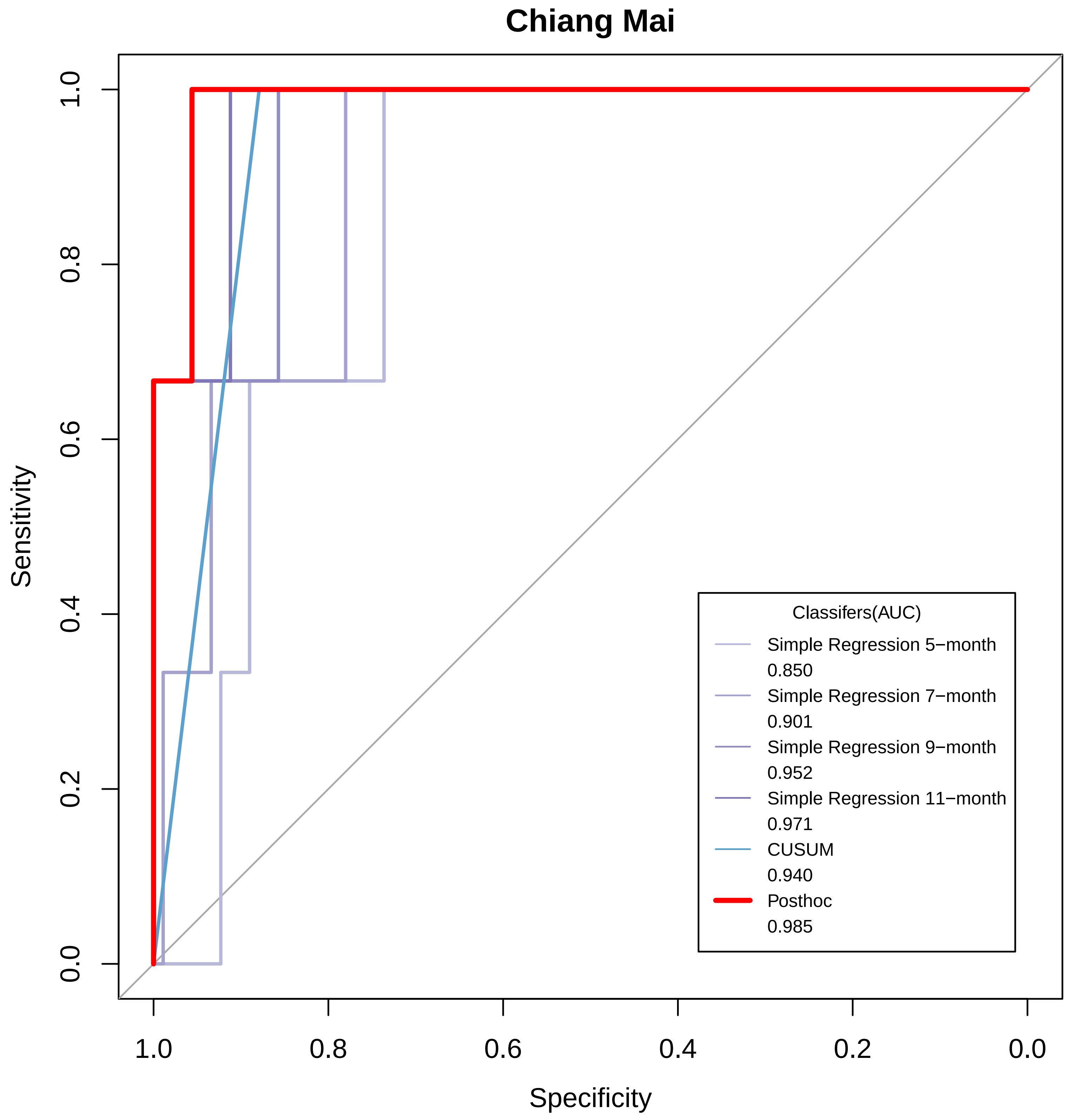
Chiang Mai
Sensitivity
Specificity
1.0
0.8
0.6
0.4
0.2
0.0
Classifers(AUC)
Simple Regression 5−month
0.850
Simple Regression 7−month
0.901
Simple Regression 9−month
0.952
Simple Regression 11−month
0.971
CUSUM
0.940
Posthoc
0.985

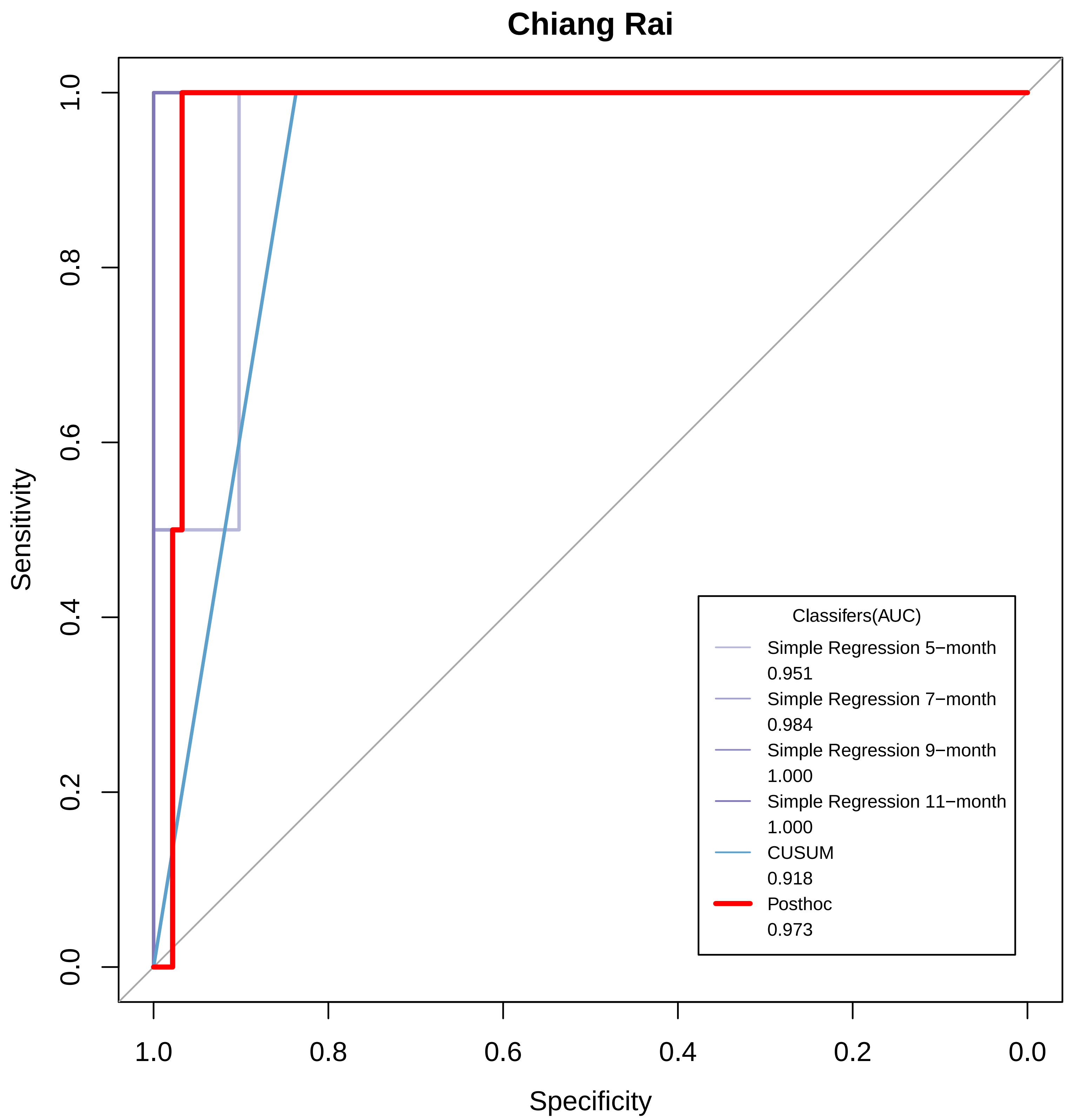

Chiang Rai
Sensitivity
Specificity
1.0
0.8
0.6
0.4
0.2
0.0
Classifers(AUC)
Simple Regression 5−month
0.951
Simple Regression 7−month
0.984
Simple Regression 9−month
1.000
Simple Regression 11−month
1.000
CUSUM
0.918
Posthoc
0.973

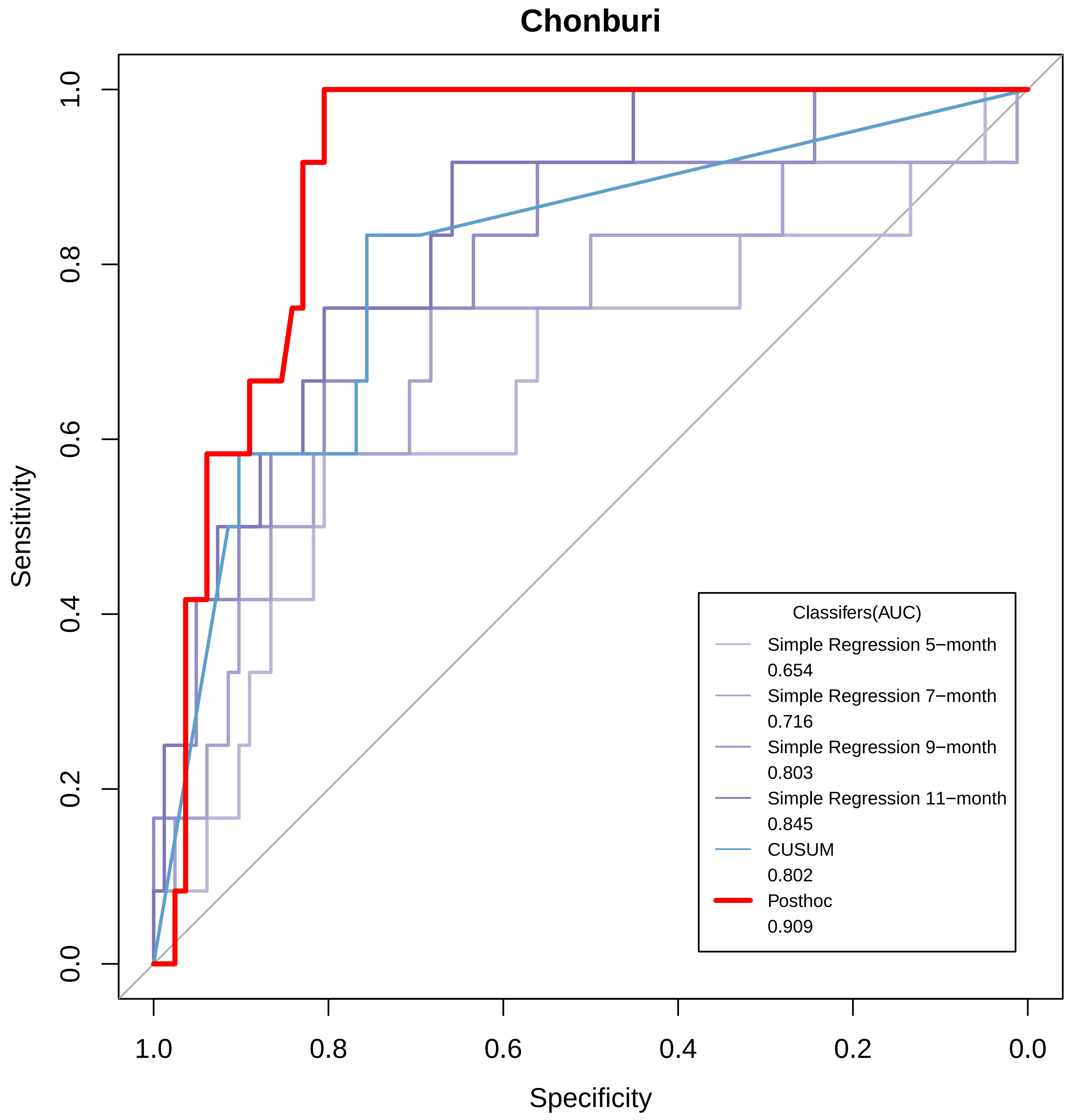
Chonburi
Sensitivity
Specificity
1.0
0.8
0.6
0.4
0.2
0.0
Classifers(AUC)
Simple Regression 5−month
0.654
Simple Regression 7−month
0.716
Simple Regression 9−month
0.803
Simple Regression 11−month
0.845
CUSUM
0.802
Posthoc
0.909

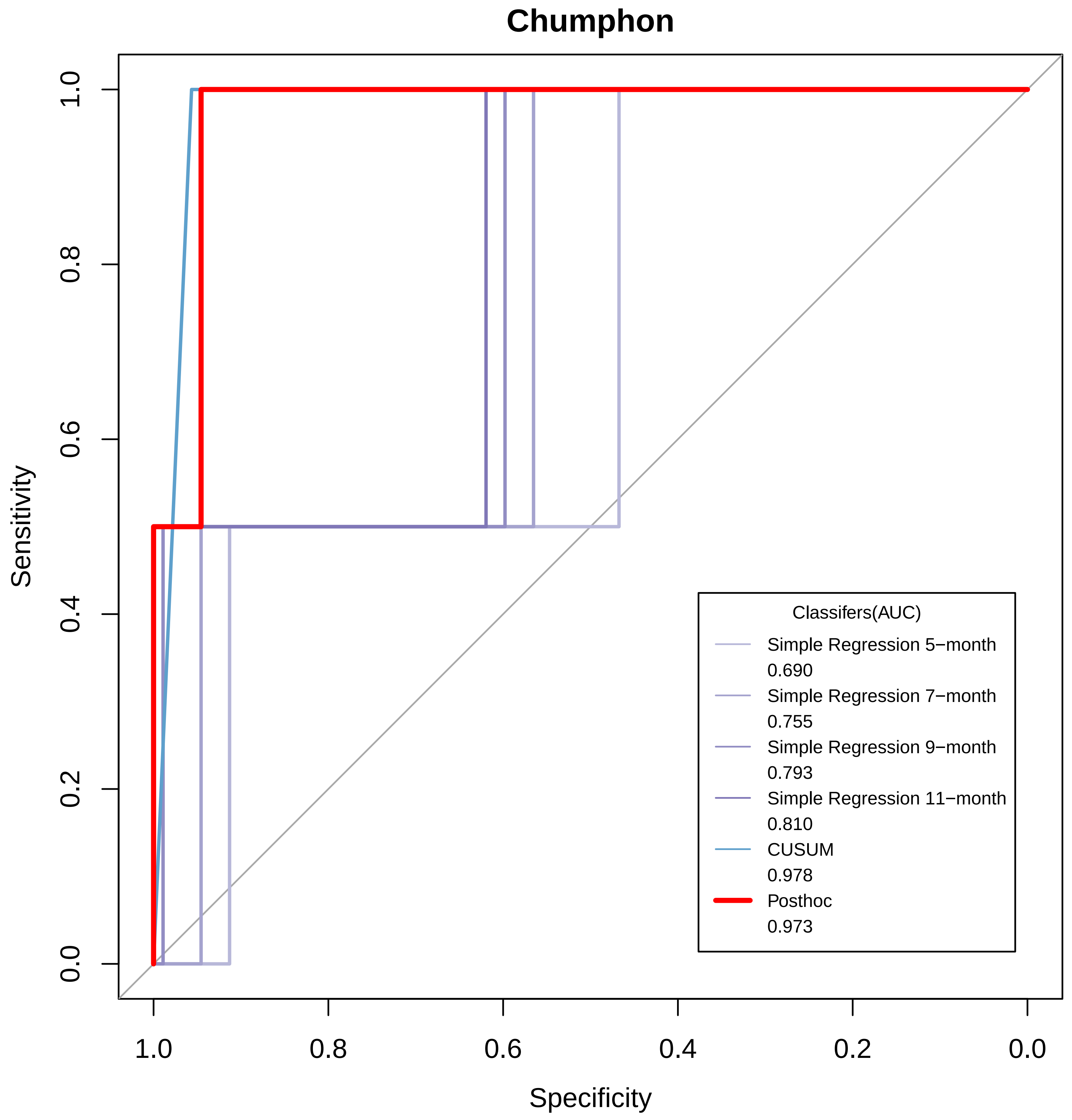

Chumphon
Sensitivity
Specificity
1.0
0.8
0.6
0.4
0.2
0.0
Classifers(AUC)
Simple Regression 5−month
0.690
Simple Regression 7−month
0.755
Simple Regression 9−month
0.793
Simple Regression 11−month
0.810
CUSUM
0.978
Posthoc
0.973

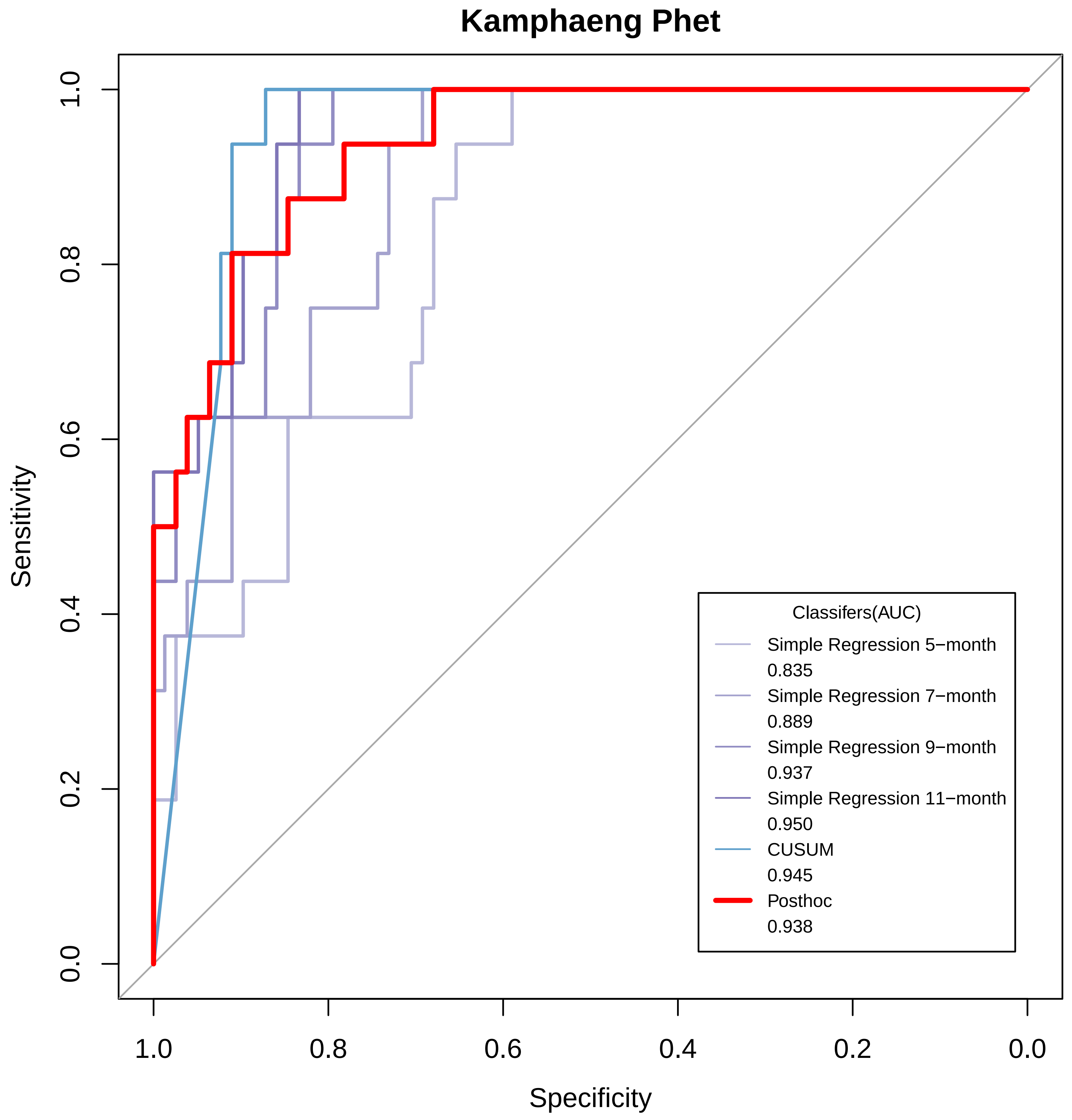

Kamphaeng Phet
Sensitivity
Specificity
0.0
0.2
0.4
0.6
0.8
1.0
1.0
0.8
0.6
0.4
0.2
0.0
Classifers(AUC)
Simple Regression 5−month
0.835
Simple Regression 7−month
0.889
Simple Regression 9−month
0.937
Simple Regression 11−month
0.950
CUSUM
0.945
Posthoc
0.938

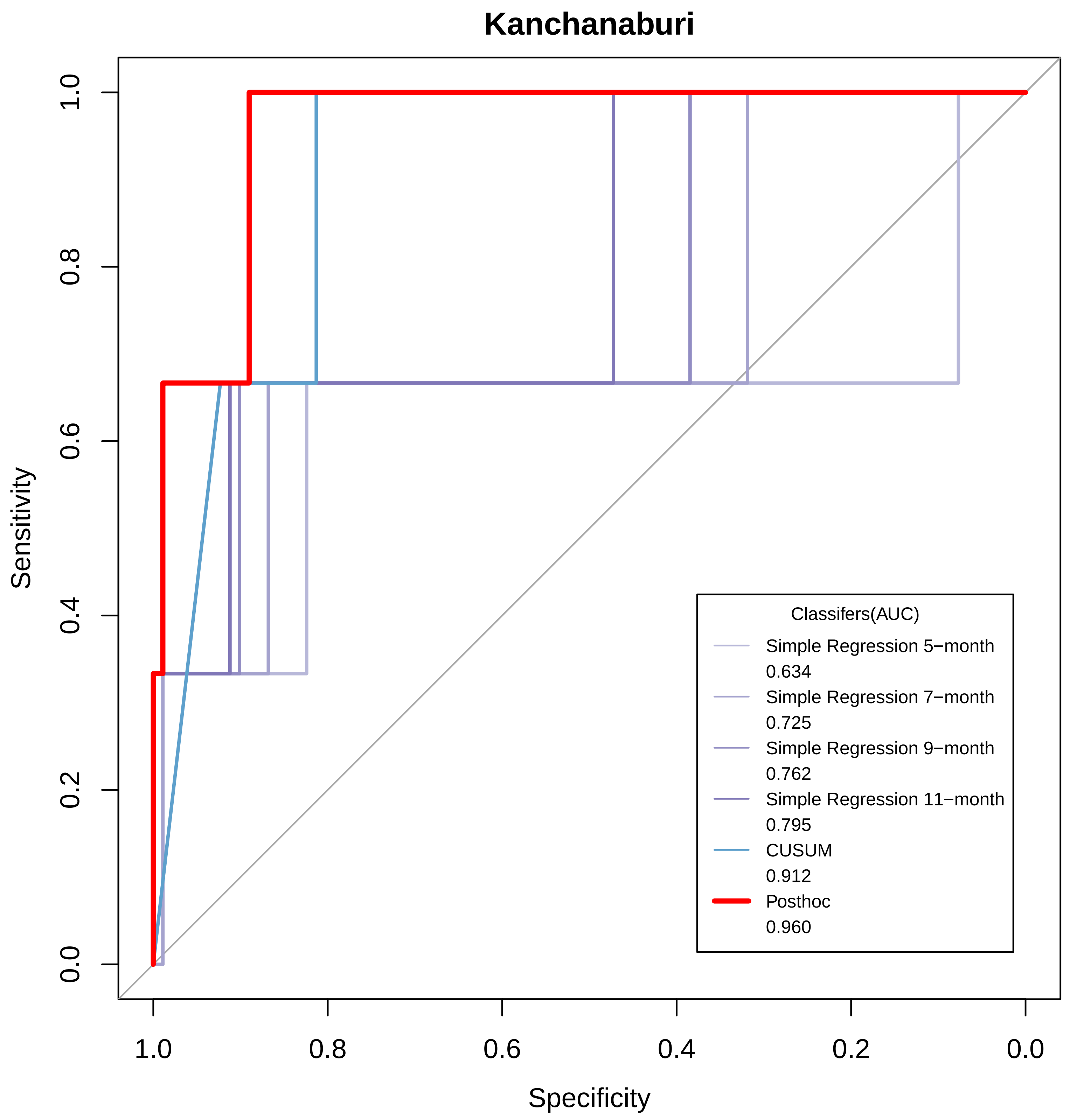

Kanchanaburi
Sensitivity
Specificity
1.0
0.8
0.6
0.4
0.2
0.0
Classifers(AUC)
Simple Regression 5−month
0.634
Simple Regression 7−month
0.725
Simple Regression 9−month
0.762
Simple Regression 11−month
0.795
CUSUM
0.912
Posthoc
0.960

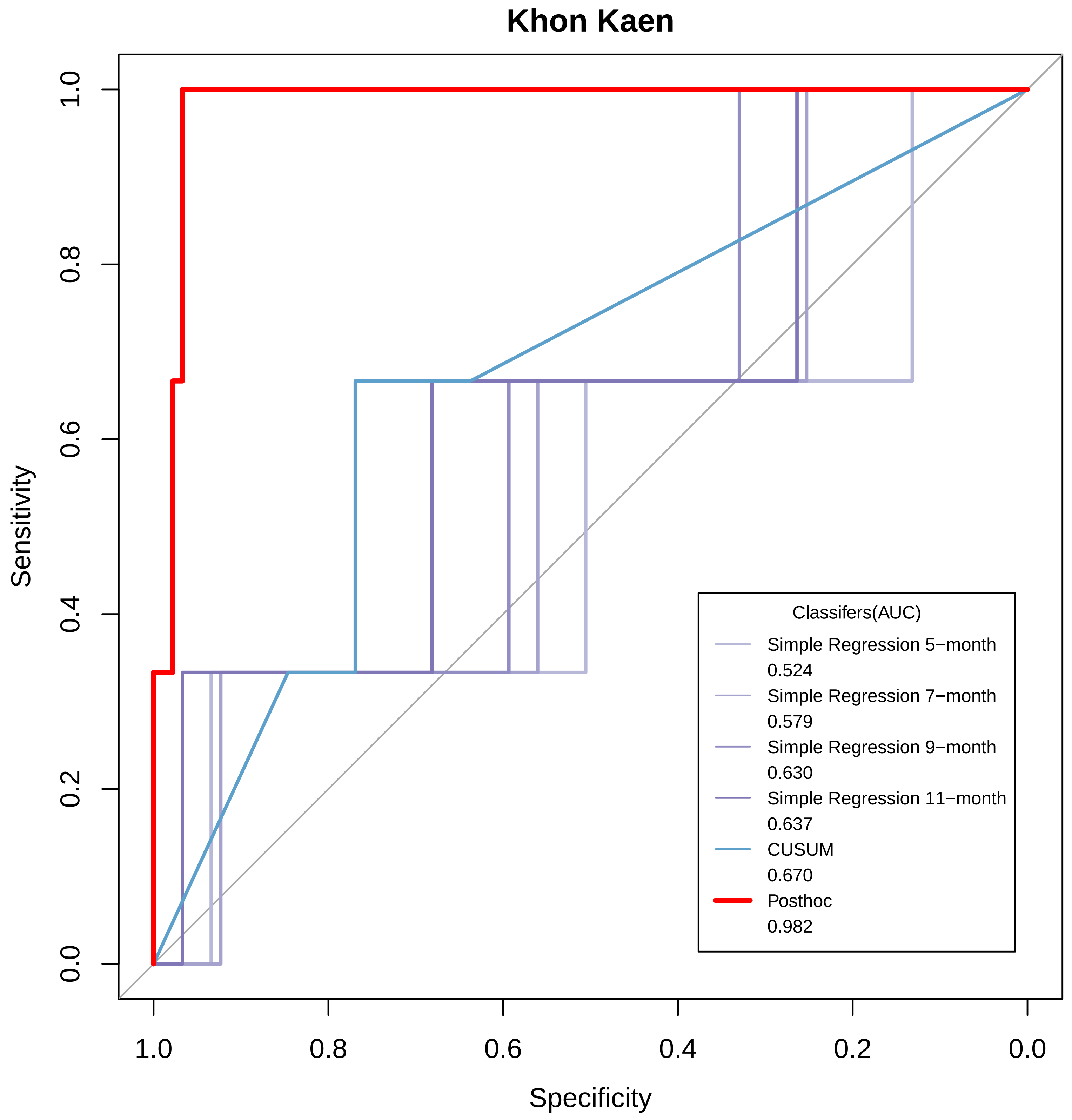
Khon Kaen
Sensitivity
Specificity
1.0
0.8
0.6
0.4
0.2
0.0
Classifers(AUC)
Simple Regression 5−month
0.524
Simple Regression 7−month
0.579
Simple Regression 9−month
0.630
Simple Regression 11−month
0.637
CUSUM
0.670
Posthoc
0.982

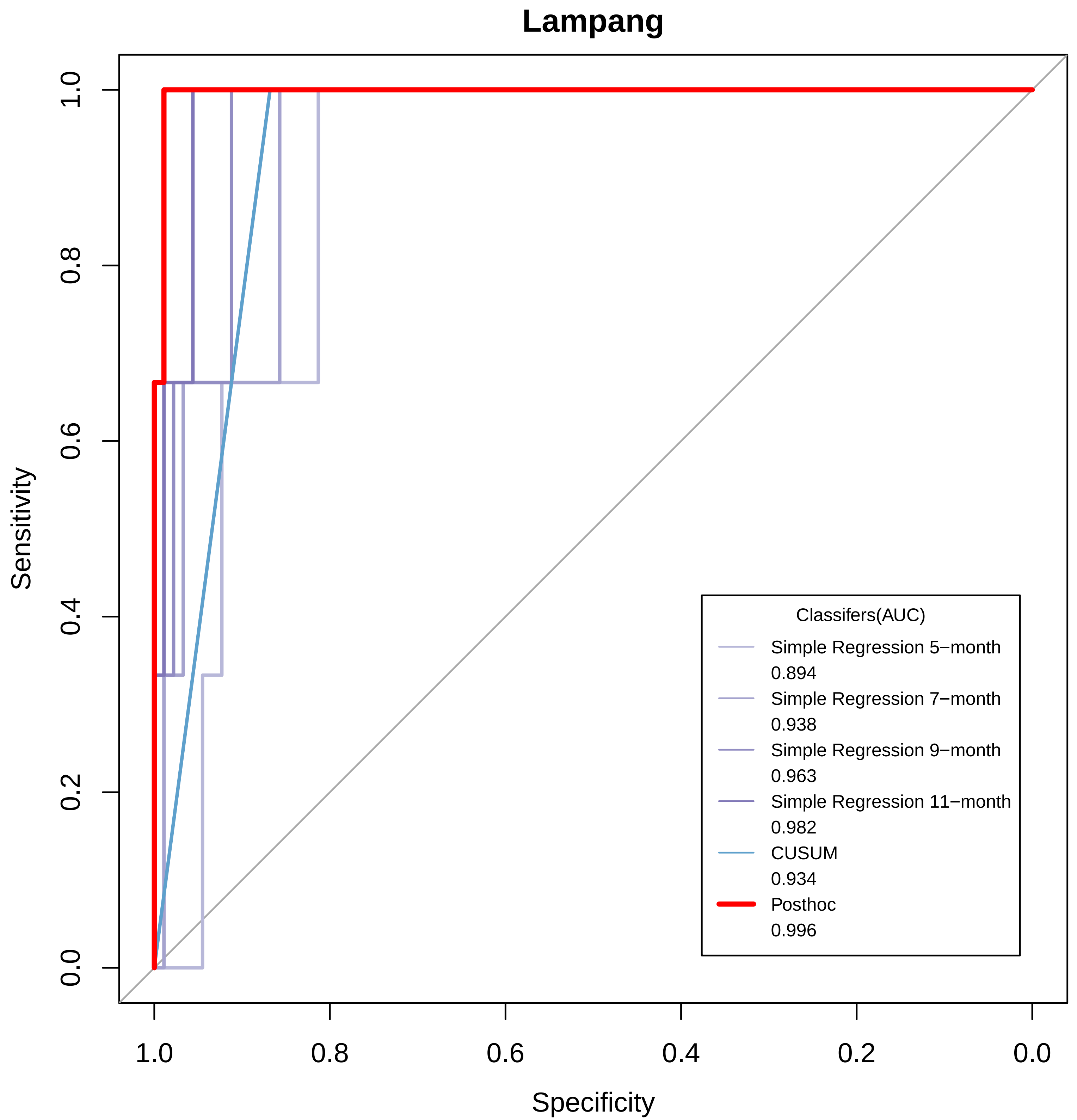

Lampang
Sensitivity
Specificity
1.0
0.8
0.6
0.4
0.2
0.0
Classifers(AUC)
Simple Regression 5−month
0.894
Simple Regression 7−month
0.938
Simple Regression 9−month
0.963
Simple Regression 11−month
0.982
CUSUM
0.934
Posthoc
0.996

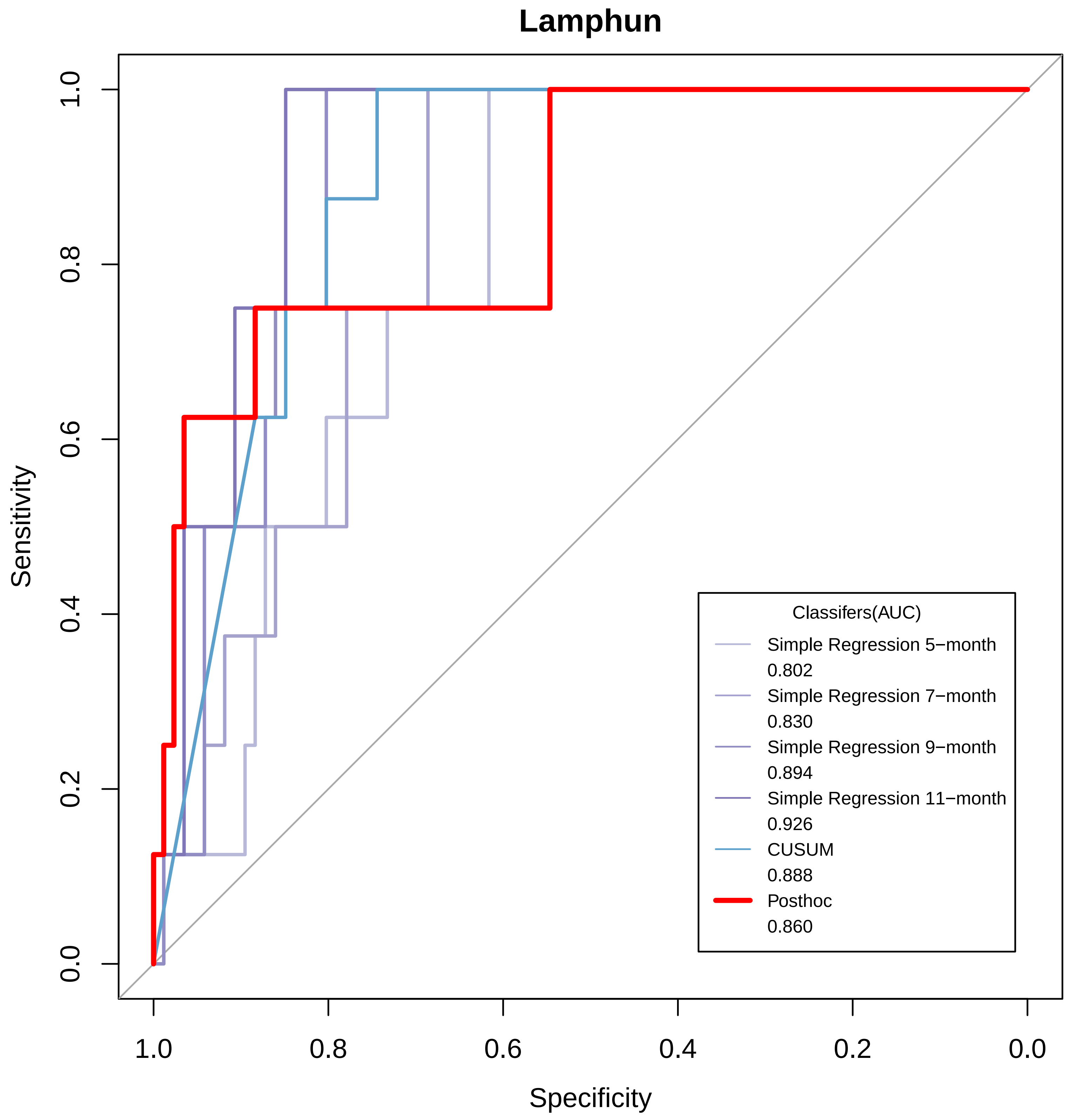
Lamphun
Sensitivity
Specificity
0.0
0.2
0.4
0.6
0.8
1.0
1.0
0.8
0.6
0.4
0.2
0.0
Classifers(AUC)
Simple Regression 5−month
0.802
Simple Regression 7−month
0.830
Simple Regression 9−month
0.894
Simple Regression 11−month
0.926
CUSUM
0.888
Posthoc
0.860

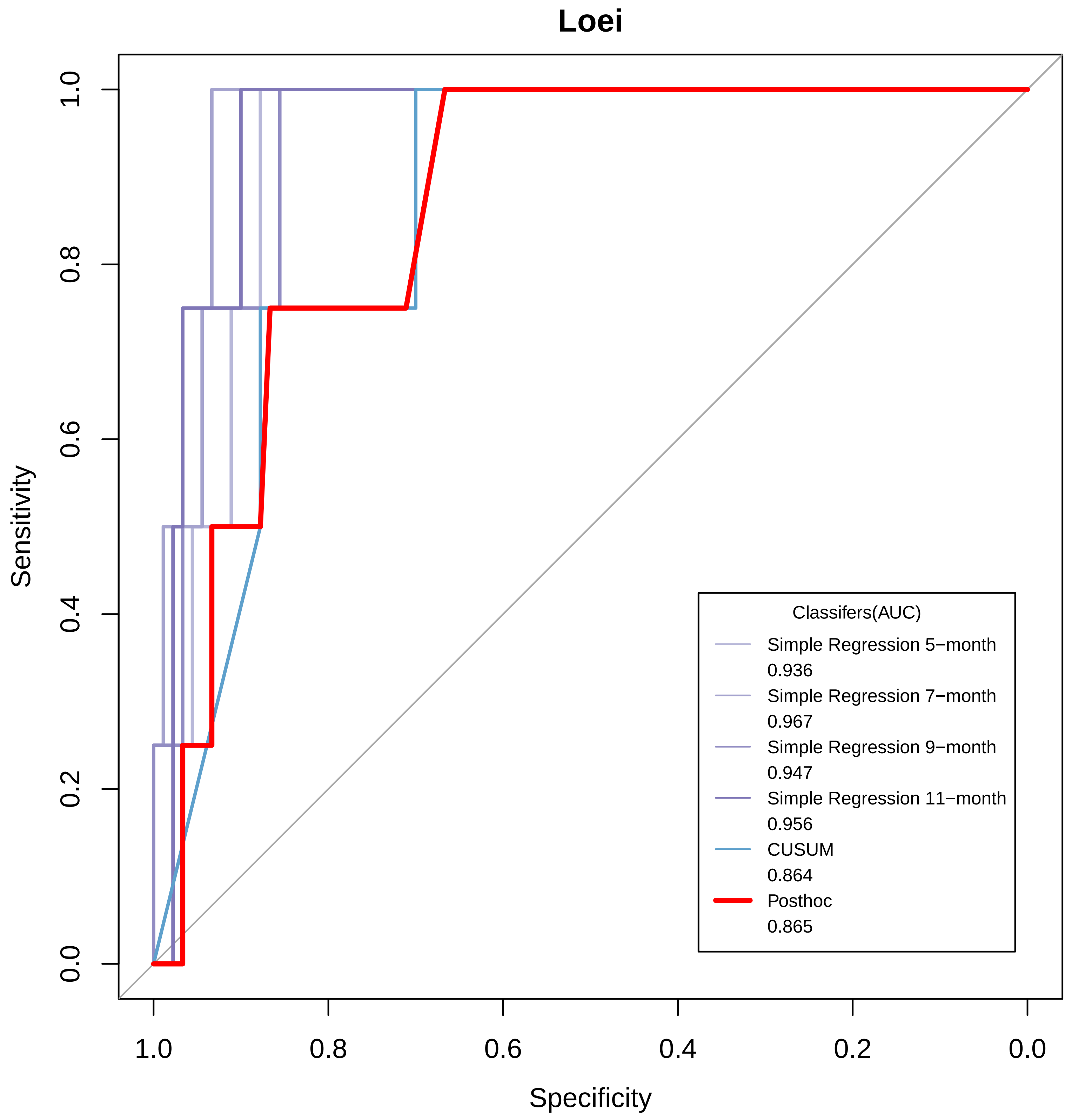

Loei
Sensitivity
Specificity
1.0
0.8
0.6
0.4
0.2
0.0
Classifers(AUC)
Simple Regression 5−month
0.936
Simple Regression 7−month
0.967
Simple Regression 9−month
0.947
Simple Regression 11−month
0.956
CUSUM
0.864
Posthoc
0.865

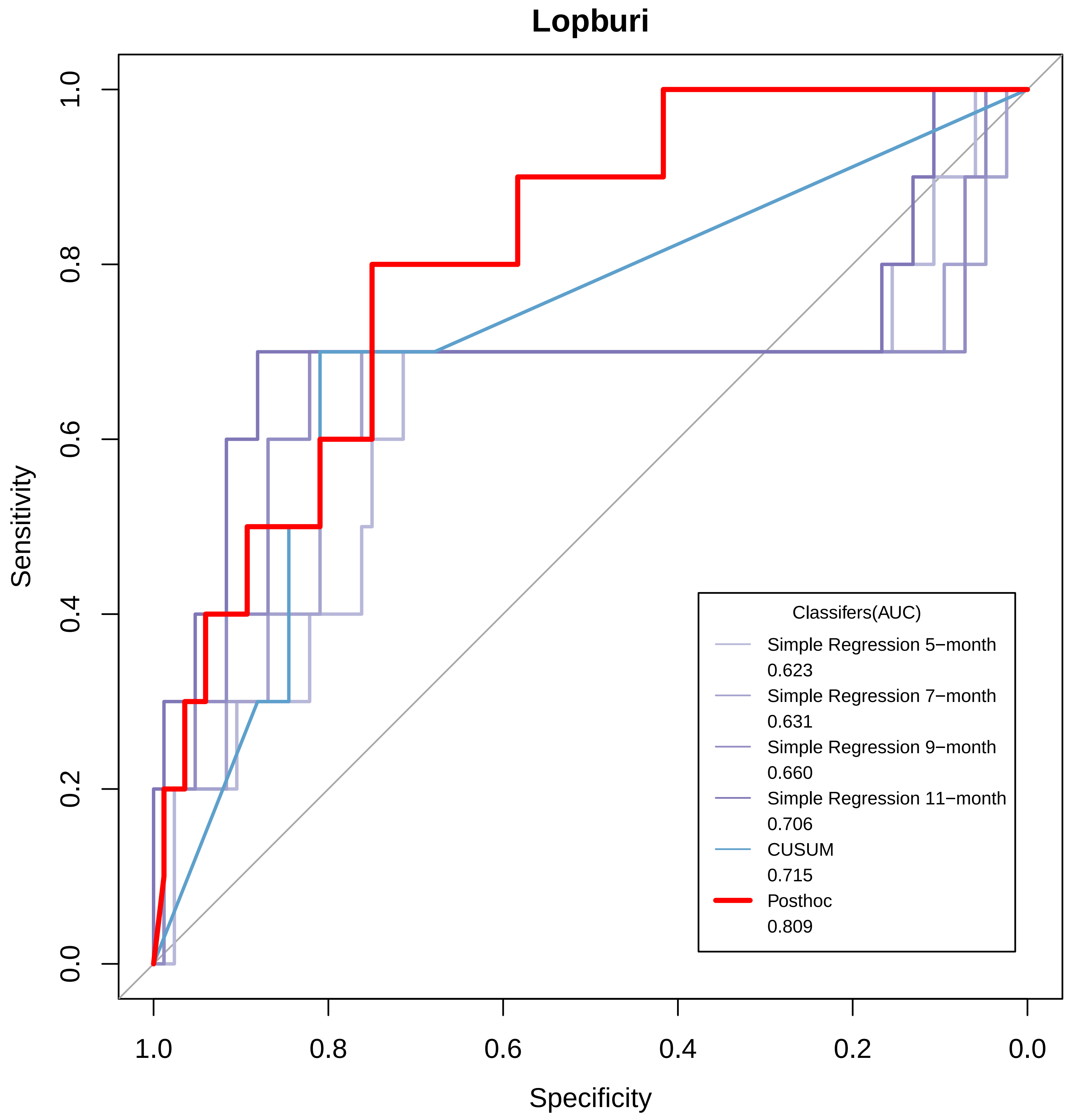
Lopburi
Sensitivity
Specificity
1.0
0.8
0.6
0.4
0.2
0.0
Classifers(AUC)
Simple Regression 5−month
0.623
Simple Regression 7−month
0.631
Simple Regression 9−month
0.660
Simple Regression 11−month
0.706
CUSUM
0.715
Posthoc
0.809

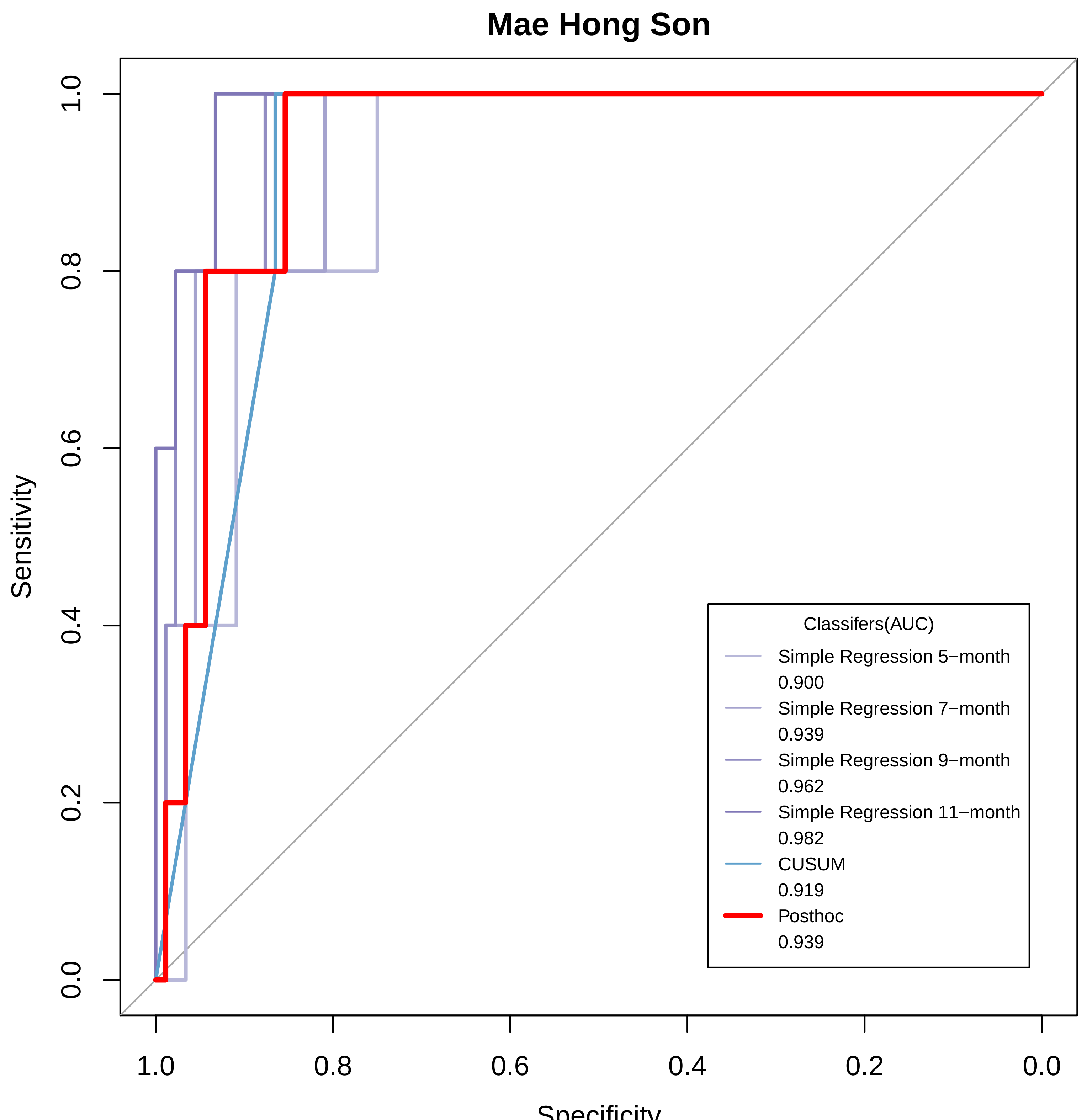

Mae Hong Son
Sensitivity
Specificity
1.0
0.8
0.6
0.4
0.2
0.0
Classifers(AUC)
Simple Regression 5−month
0.900
Simple Regression 7−month
0.939
Simple Regression 9−month
0.962
Simple Regression 11−month
0.982
CUSUM
0.919
Posthoc
0.939

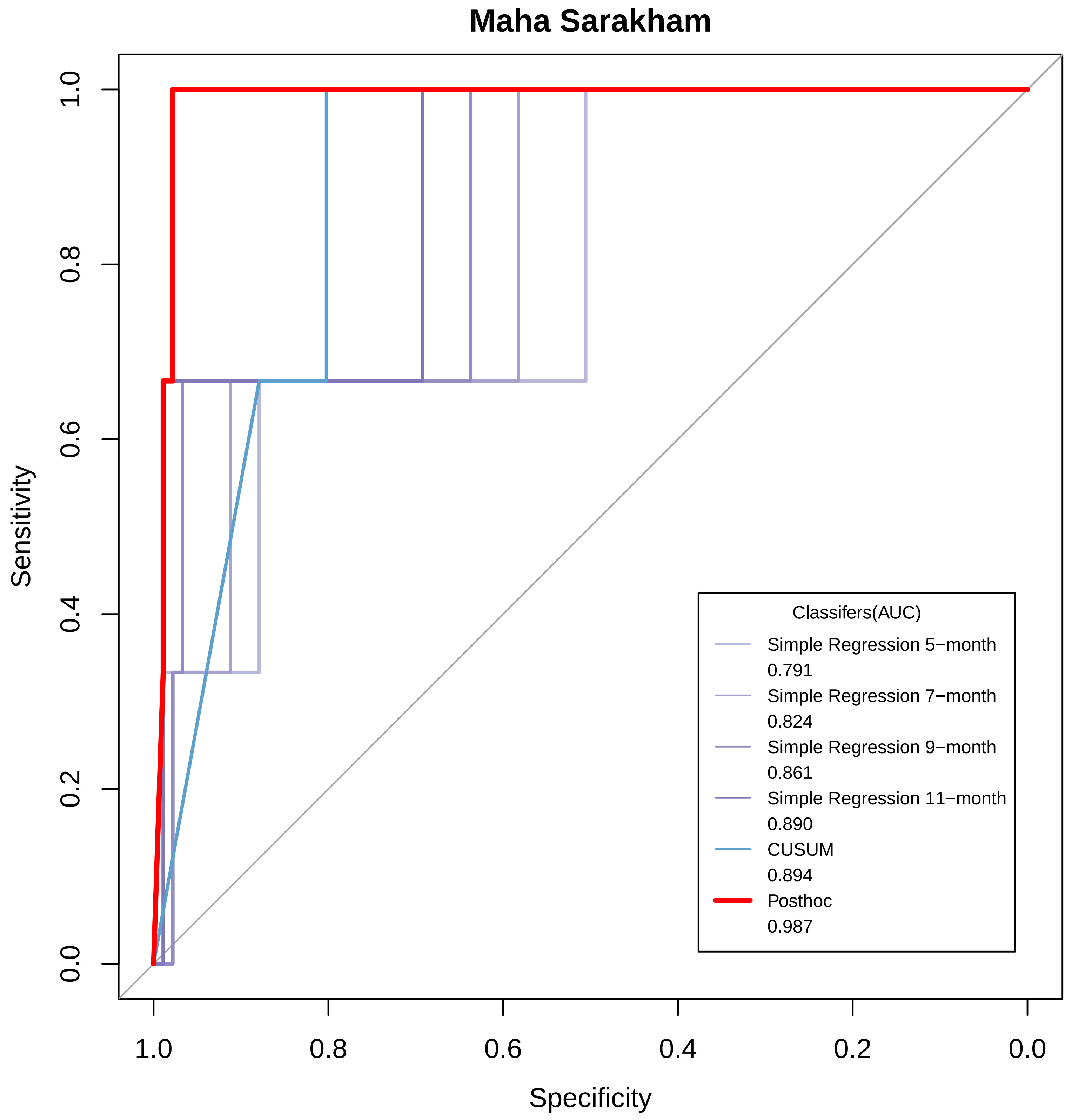

Maha Sarakham
Sensitivity
Specificity
1.0
0.8
0.6
0.4
0.2
0.0
Classifers(AUC)
Simple Regression 5−month
0.791
Simple Regression 7−month
0.824
Simple Regression 9−month
0.861
Simple Regression 11−month
0.890
CUSUM
0.894
Posthoc
0.987

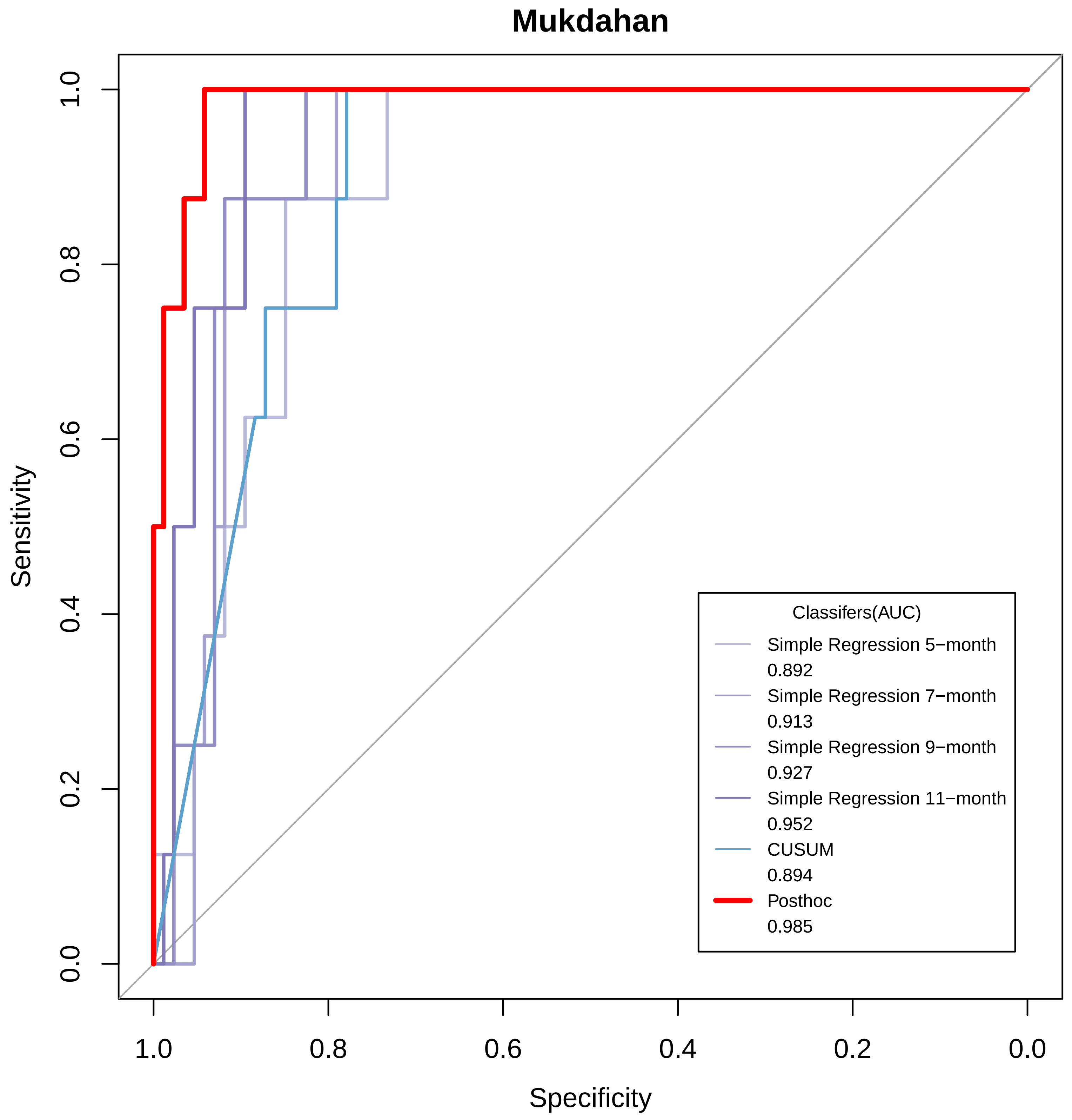
Mukdahan
Sensitivity
Specificity
1.0
0.8
0.6
0.4
0.2
0.0
Classifers(AUC)
Simple Regression 5−month
0.892
Simple Regression 7−month
0.913
Simple Regression 9−month
0.927
Simple Regression 11−month
0.952
CUSUM
0.894
Posthoc
0.985

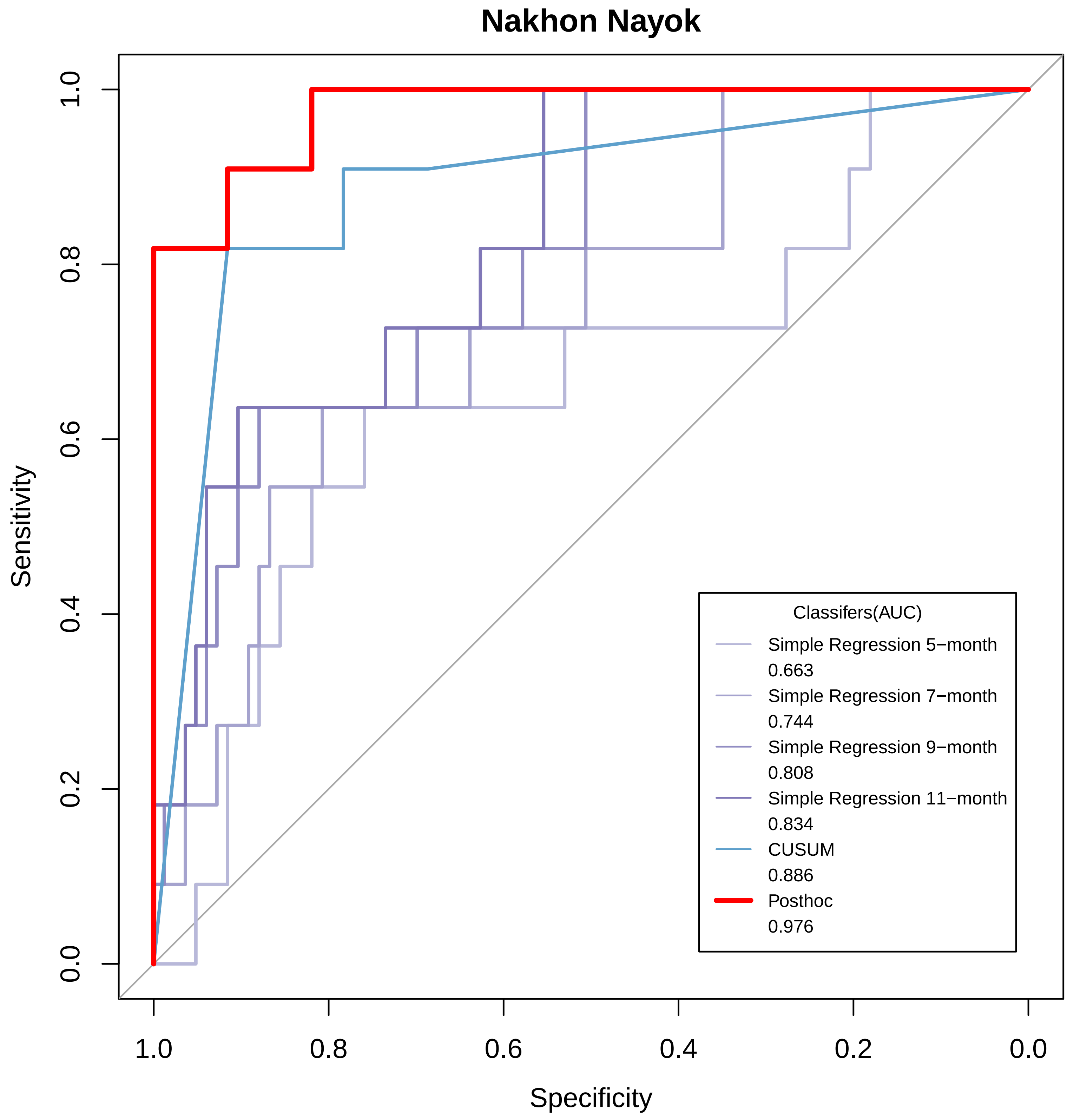
Nakhon Nayok
Sensitivity
Specificity
1.0
0.8
0.6
0.4
0.2
0.0
Classifers(AUC)
Simple Regression 5−month
0.663
Simple Regression 7−month
0.744
Simple Regression 9−month
0.808
Simple Regression 11−month
0.834
CUSUM
0.886
Posthoc
0.976

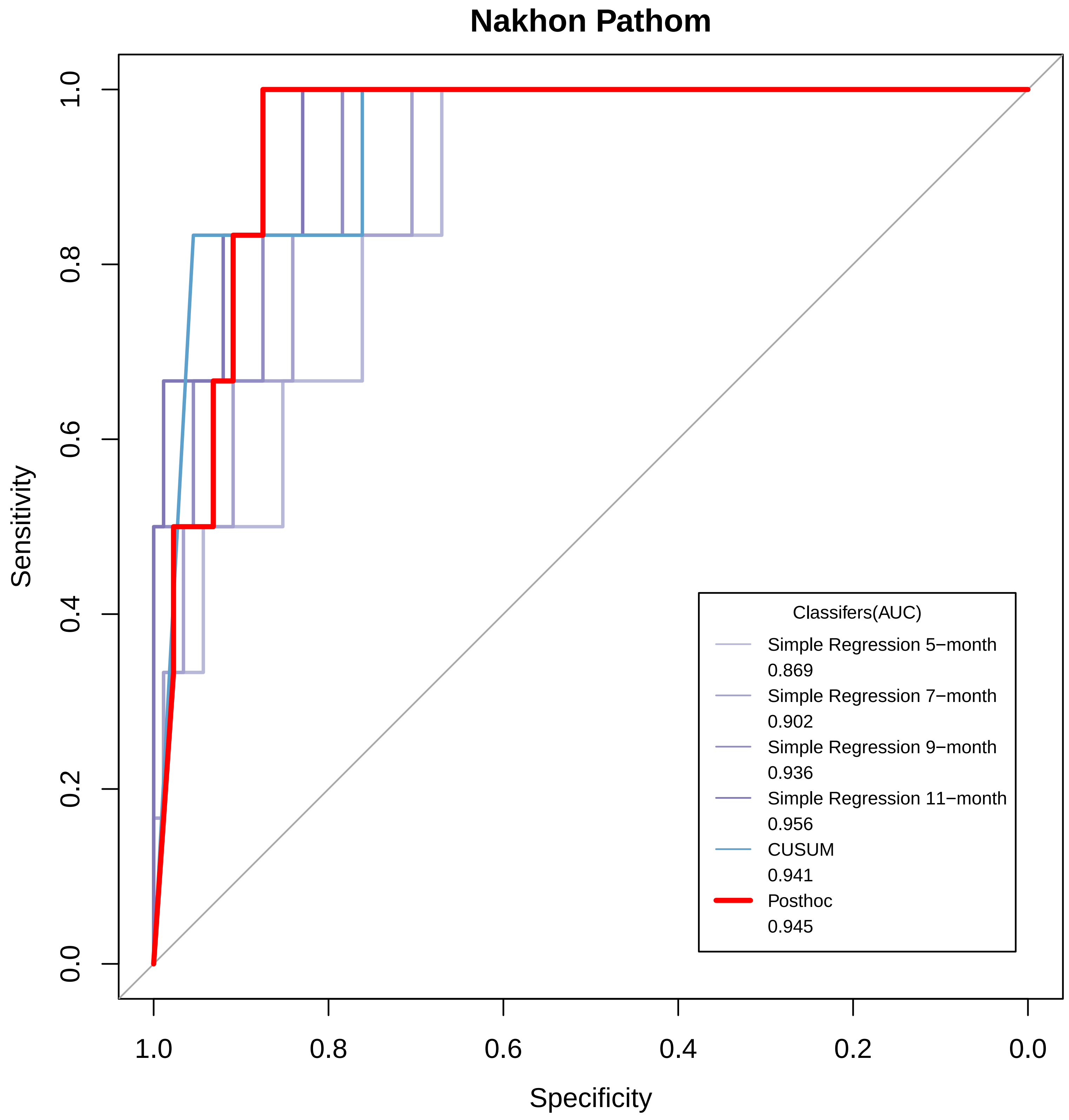
Nakhon Pathom
Sensitivity
Specificity
1.0
0.8
0.6
0.4
0.2
0.0
Classifers(AUC)
Simple Regression 5−month
0.869
Simple Regression 7−month
0.902
Simple Regression 9−month
0.936
Simple Regression 11−month
0.956
CUSUM
0.941
Posthoc
0.945

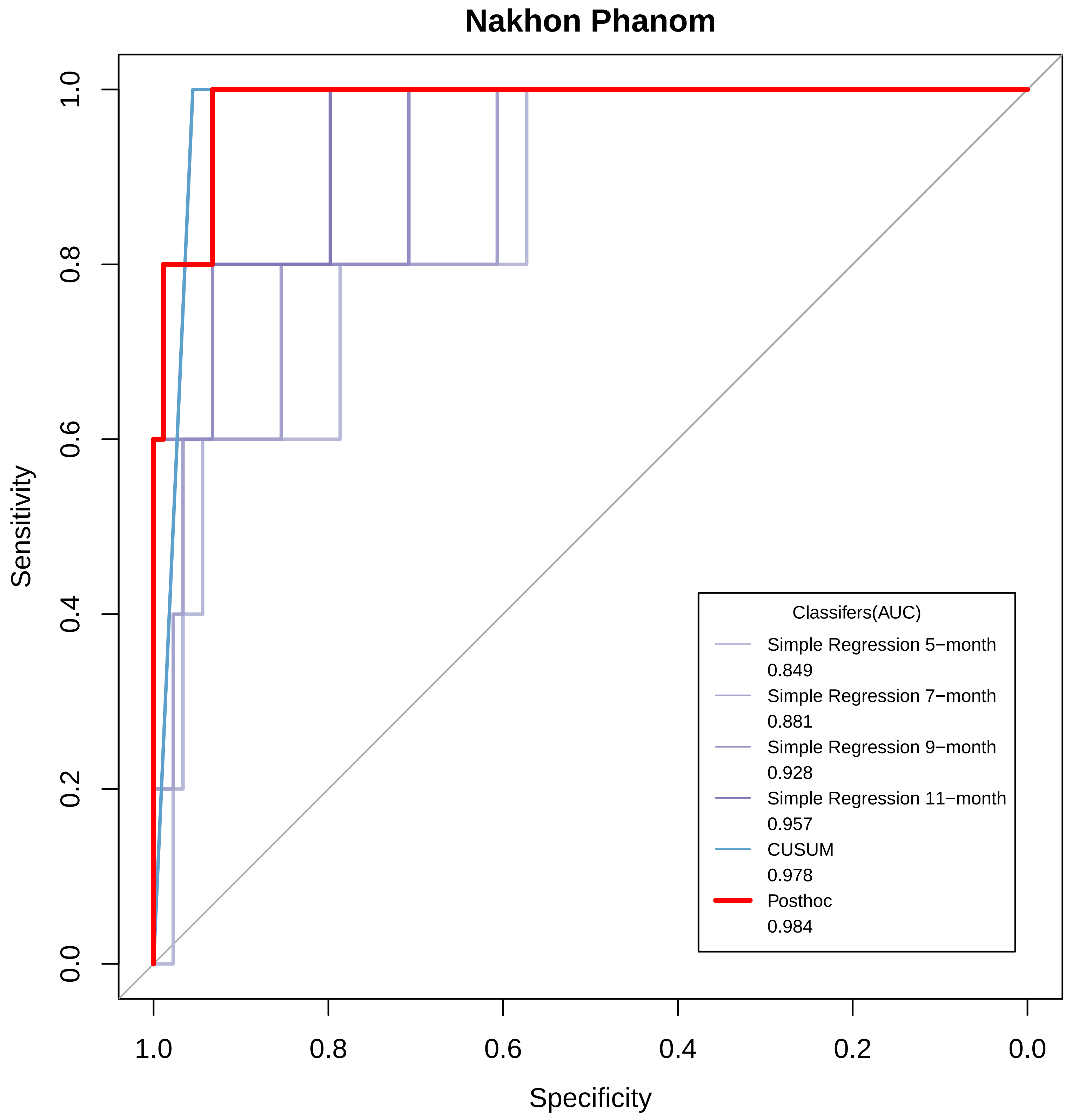

Nakhon Phanom
Sensitivity
Specificity
1.0
0.8
0.6
0.4
0.2
0.0
Classifers(AUC)
Simple Regression 5−month
0.849
Simple Regression 7−month
0.881
Simple Regression 9−month
0.928
Simple Regression 11−month
0.957
CUSUM
0.978
Posthoc
0.984

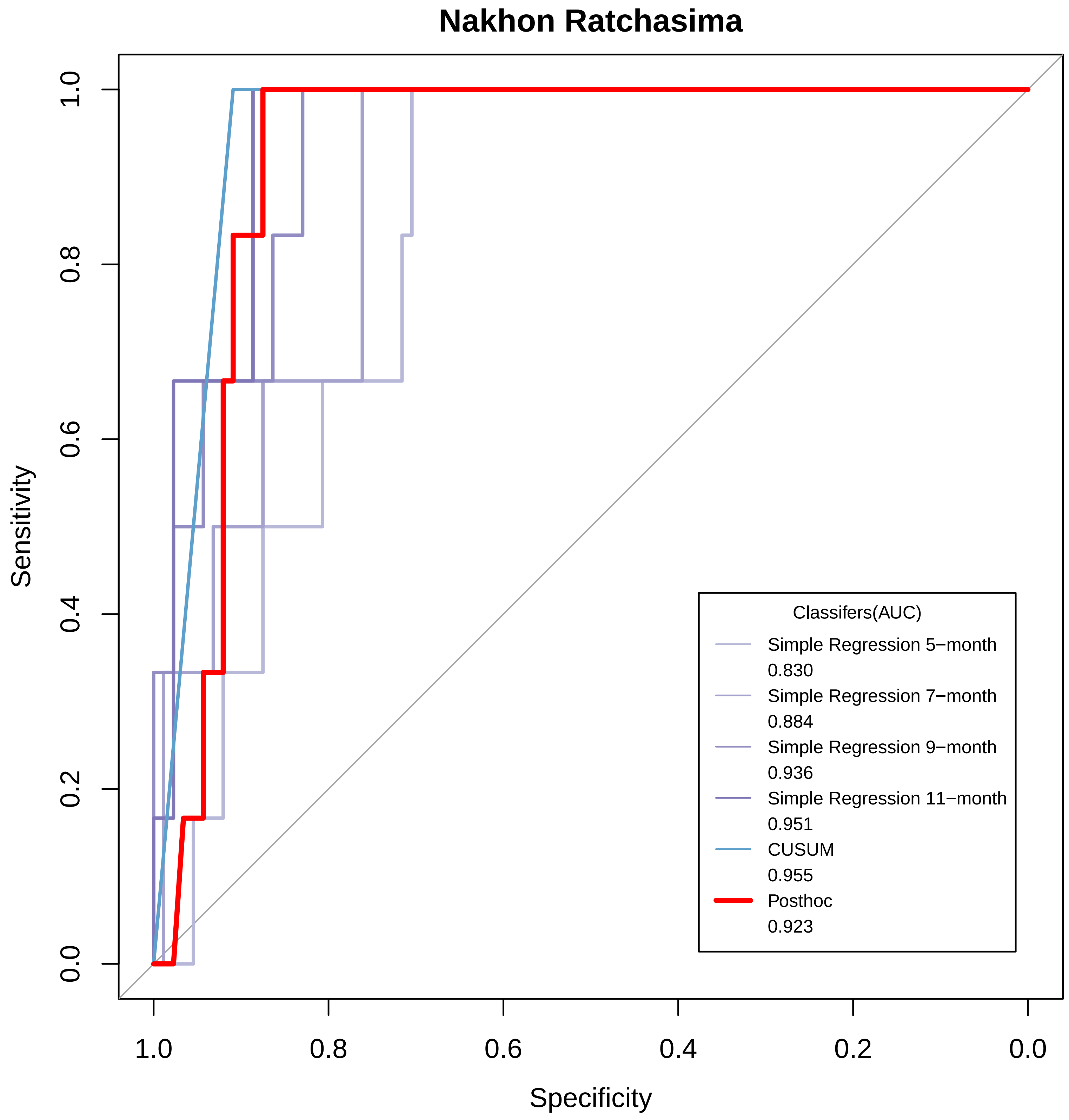

Nakhon Ratchasima
Sensitivity
Specificity
1.0
0.8
0.6
0.4
0.2
0.0
Classifers(AUC)
Simple Regression 5−month
0.830
Simple Regression 7−month
0.884
Simple Regression 9−month
0.936
Simple Regression 11−month
0.951
CUSUM
0.955
Posthoc
0.923

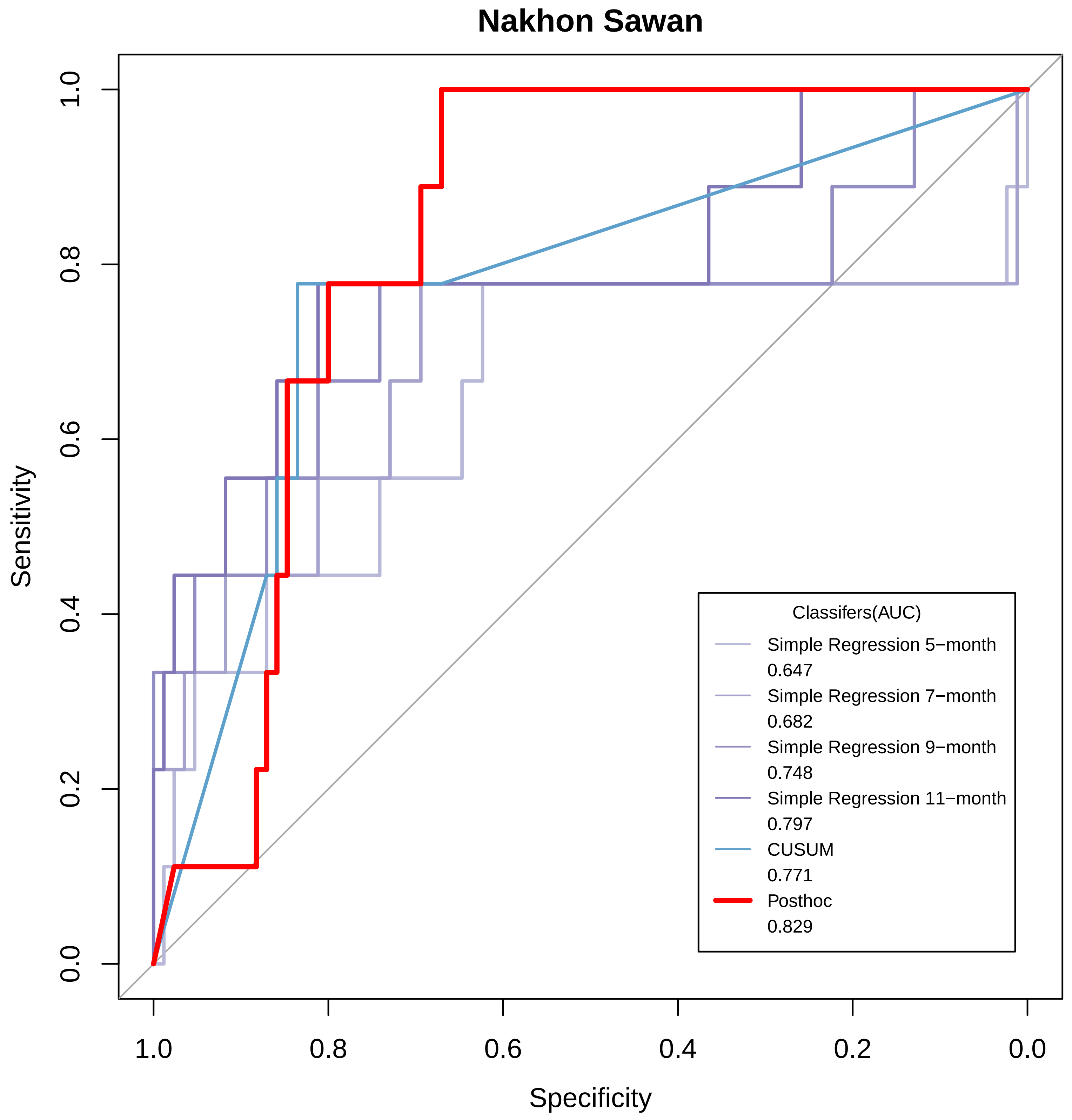
Nakhon Sawan
Sensitivity
Specificity
1.0
0.8
0.6
0.4
0.2
0.0
Classifers(AUC)
Simple Regression 5−month
0.647
Simple Regression 7−month
0.682
Simple Regression 9−month
0.748
Simple Regression 11−month
0.797
CUSUM
0.771
Posthoc
0.829

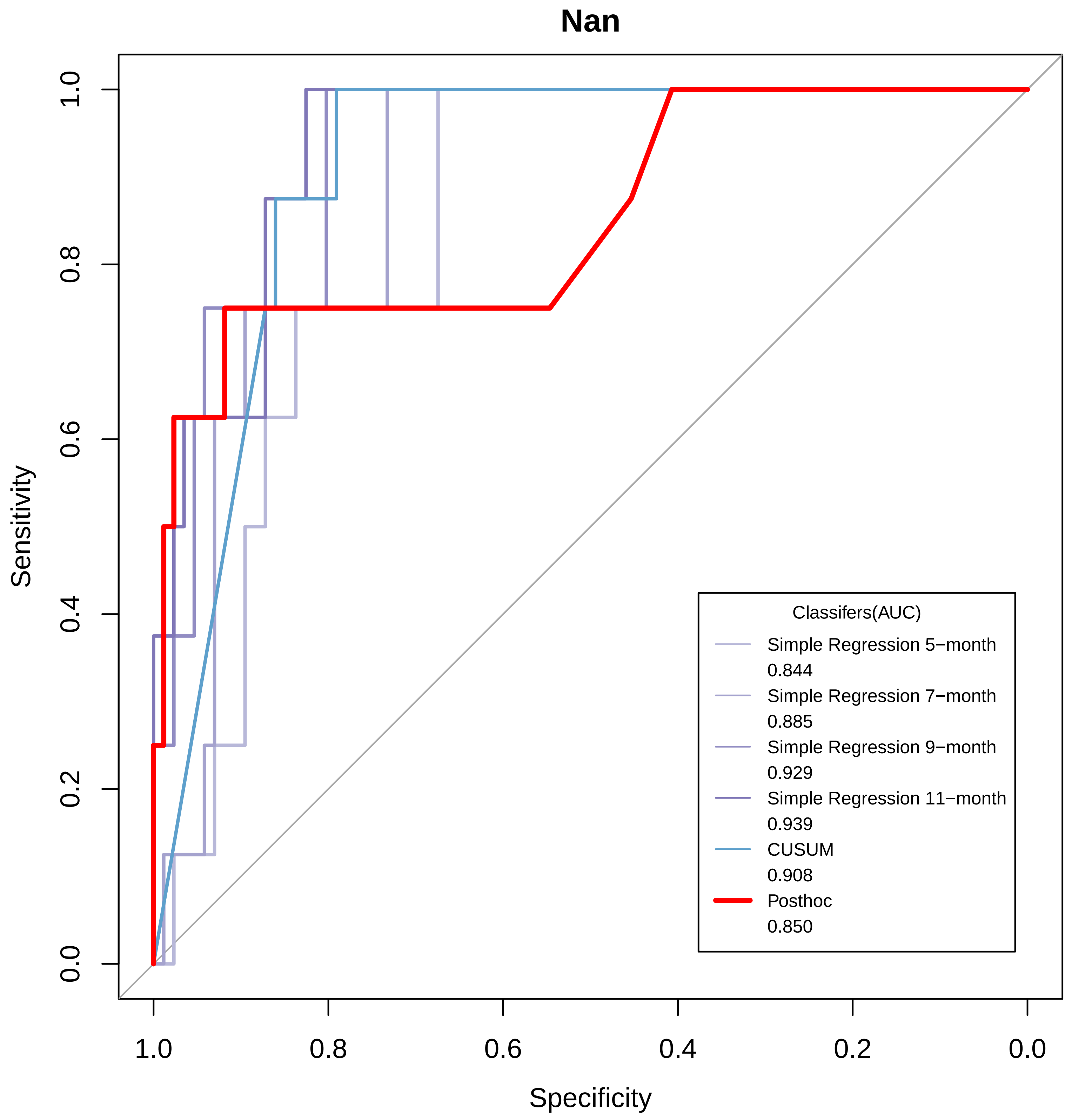
Nan
Sensitivity
Specificity
1.0
0.8
0.6
0.4
0.2
0.0
Classifers(AUC)
Simple Regression 5−month
0.844
Simple Regression 7−month
0.885
Simple Regression 9−month
0.929
Simple Regression 11−month
0.939
CUSUM
0.908
Posthoc
0.850

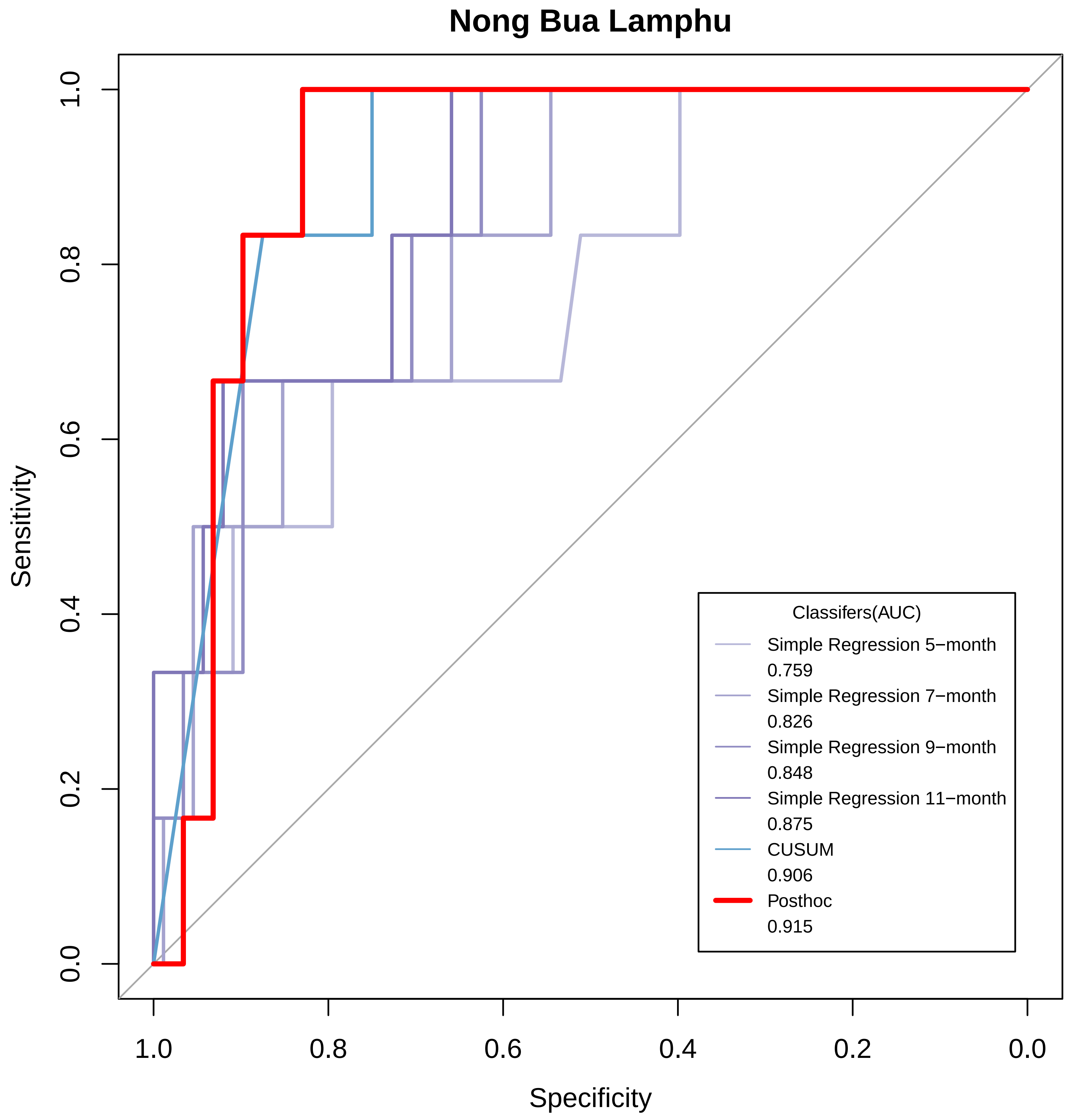

Nong Bua Lamphu
Sensitivity
Specificity
1.0
0.8
0.6
0.4
0.2
0.0
Classifers(AUC)
Simple Regression 5−month
0.759
Simple Regression 7−month
0.826
Simple Regression 9−month
0.848
Simple Regression 11−month
0.875
CUSUM
0.906
Posthoc
0.915

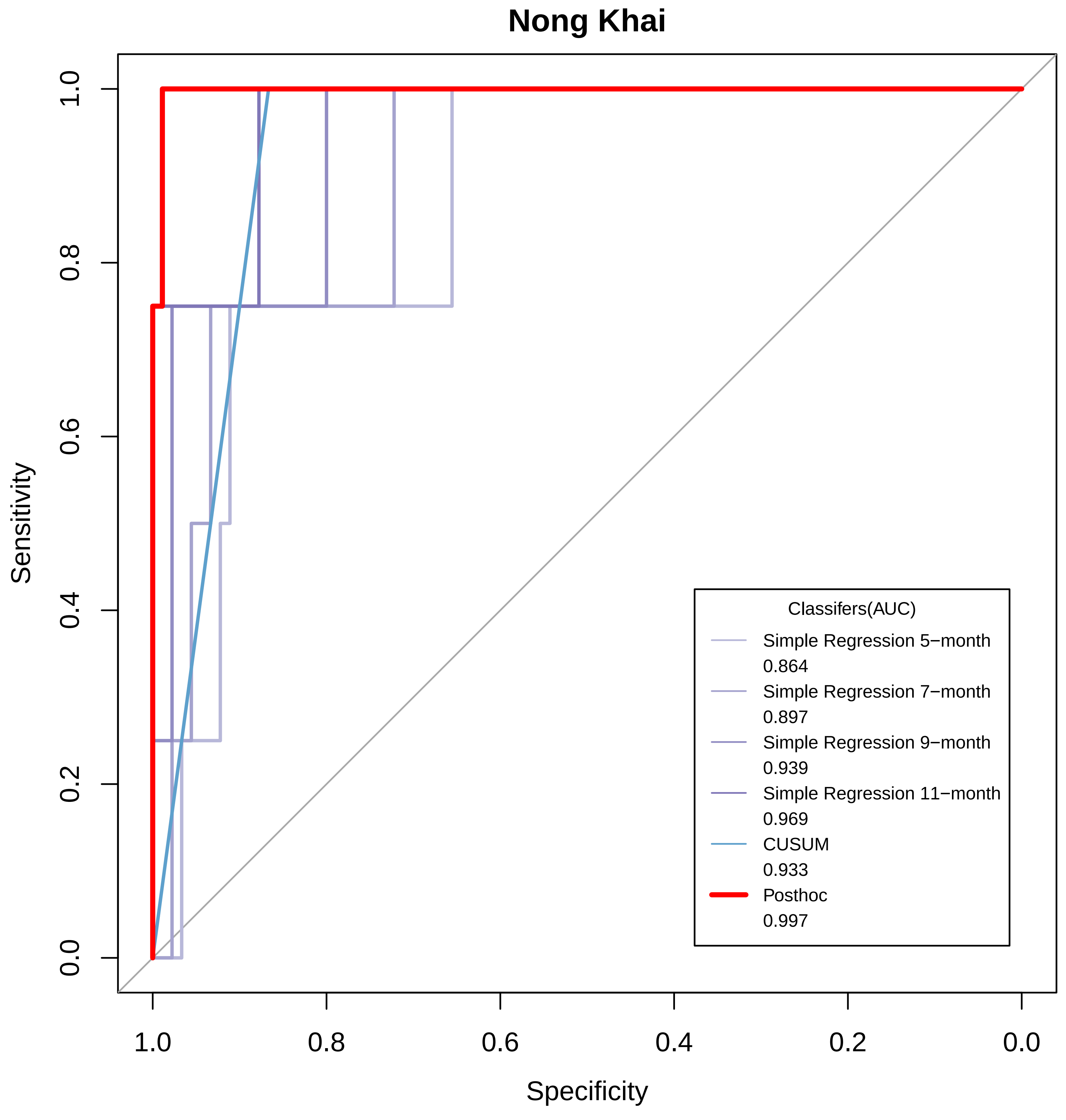
Nong Khai
Sensitivity
Specificity
1.0
0.8
0.6
0.4
0.2
0.0
Classifers(AUC)
Simple Regression 5−month
0.864
Simple Regression 7−month
0.897
Simple Regression 9−month
0.939
Simple Regression 11−month
0.969
CUSUM
0.933
Posthoc
0.997

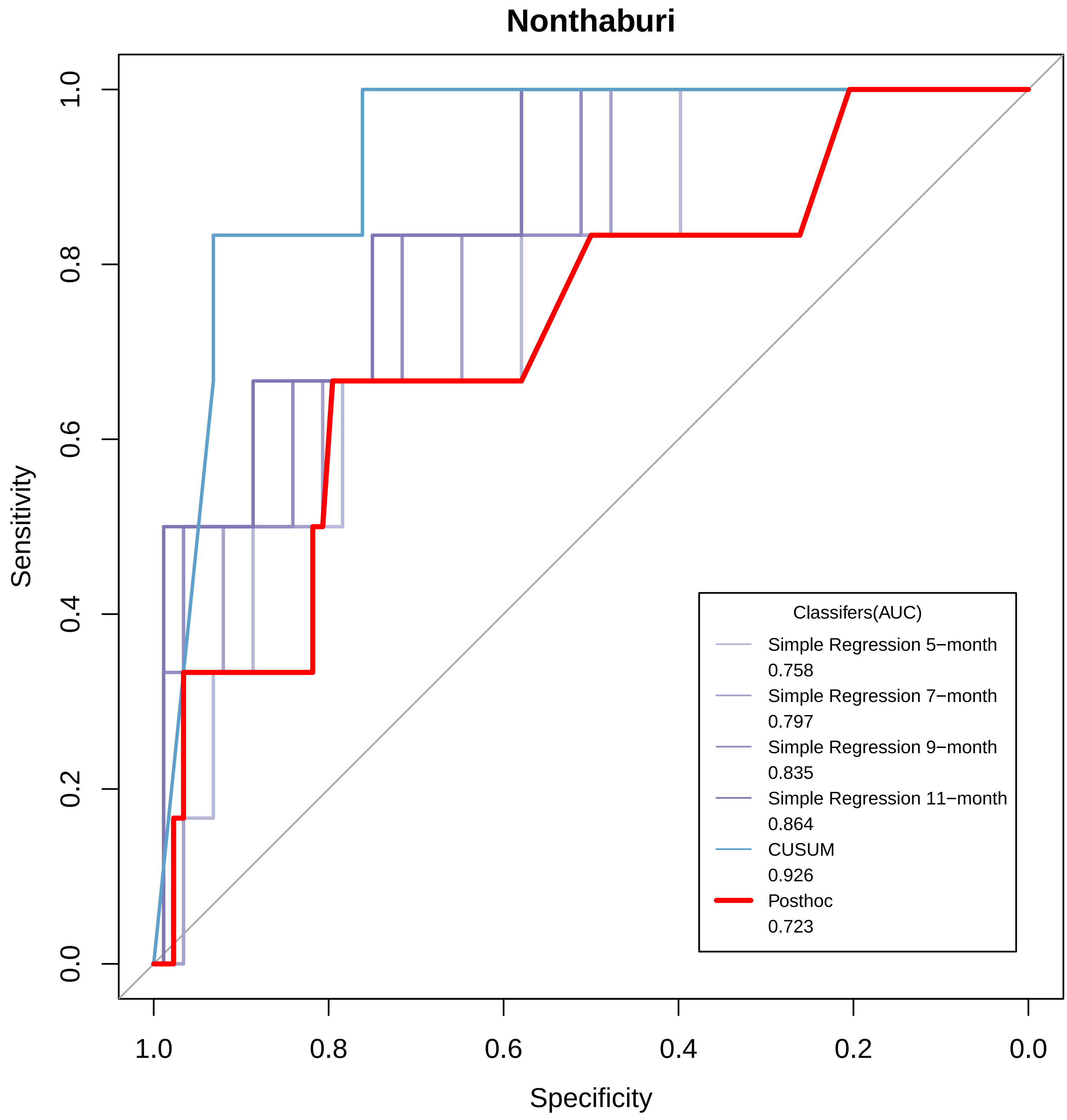

Nonthaburi
Sensitivity
Specificity
1.0
0.8
0.6
0.4
0.2
0.0
Classifers(AUC)
Simple Regression 5−month
0.758
Simple Regression 7−month
0.797
Simple Regression 9−month
0.835
Simple Regression 11−month
0.864
CUSUM
0.926
Posthoc
0.723

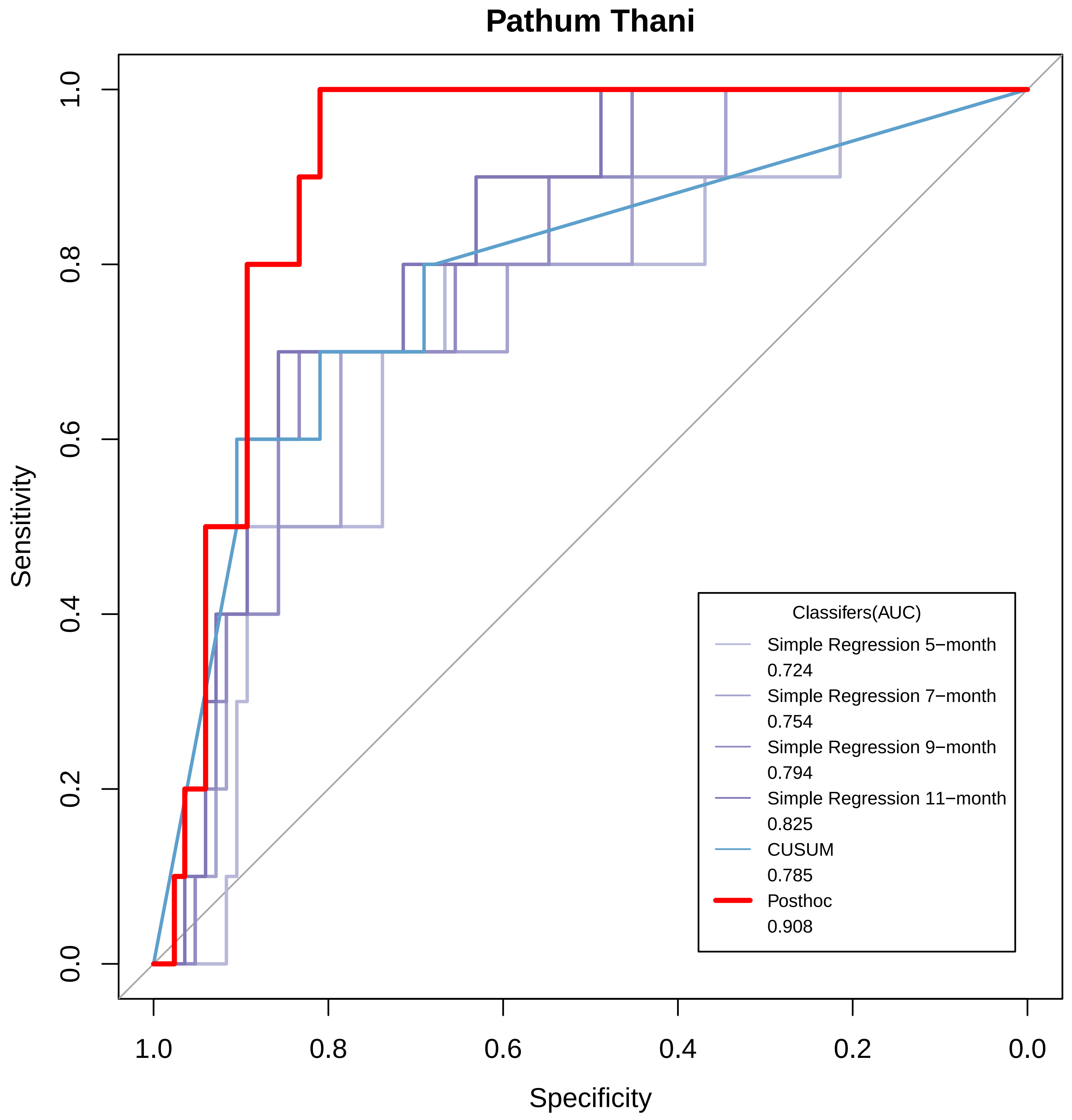

Pathum Thani
Sensitivity
Specificity
1.0
0.8
0.6
0.4
0.2
0.0
Classifers(AUC)
Simple Regression 5−month
0.724
Simple Regression 7−month
0.754
Simple Regression 9−month
0.794
Simple Regression 11−month
0.825
CUSUM
0.785
Posthoc
0.908

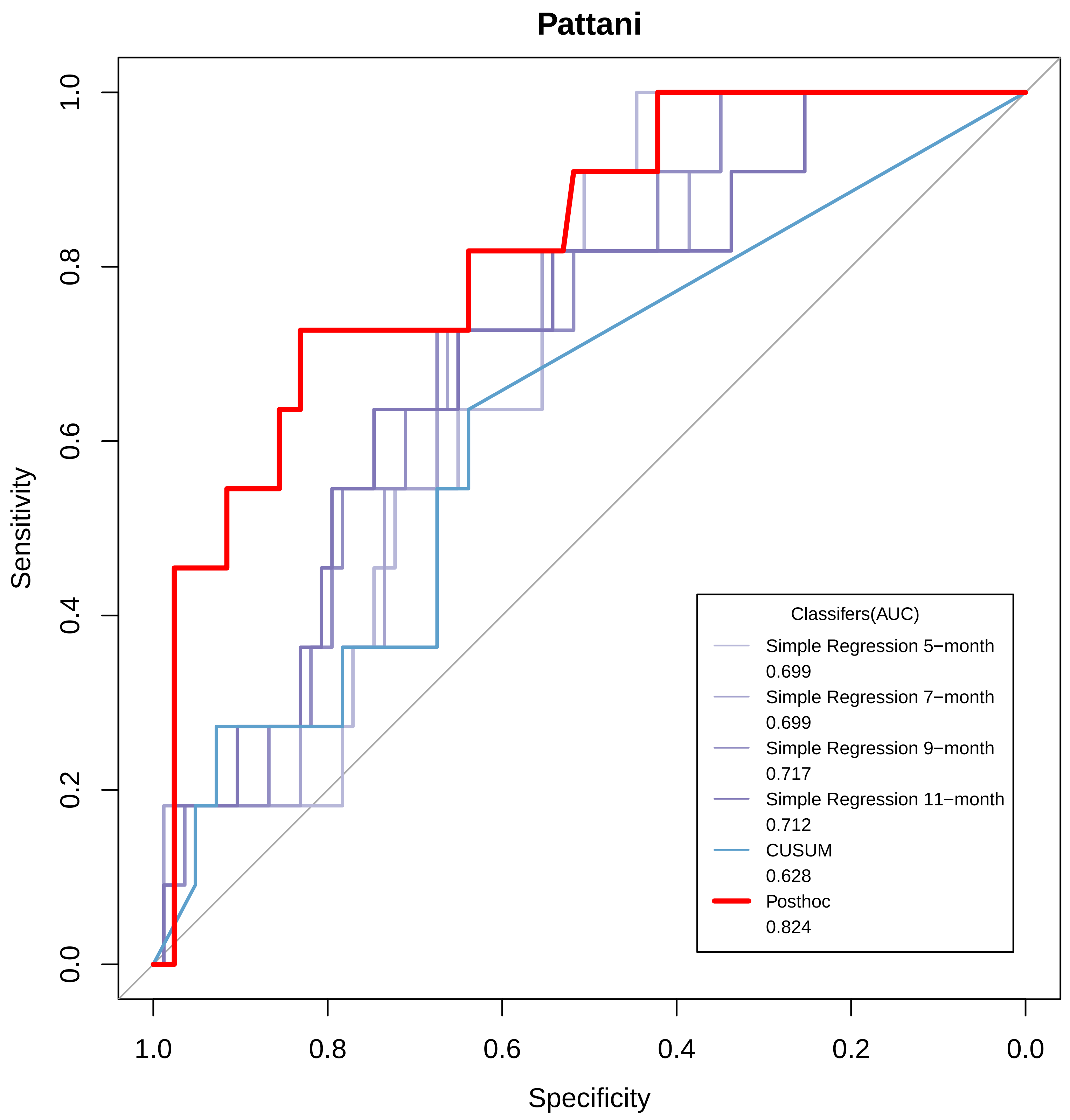
Pattani
Sensitivity
Specificity
1.0
0.8
0.6
0.4
0.2
0.0
Classifers(AUC)
Simple Regression 5−month
0.699
Simple Regression 7−month
0.699
Simple Regression 9−month
0.717
Simple Regression 11−month
0.712
CUSUM
0.628
Posthoc
0.824

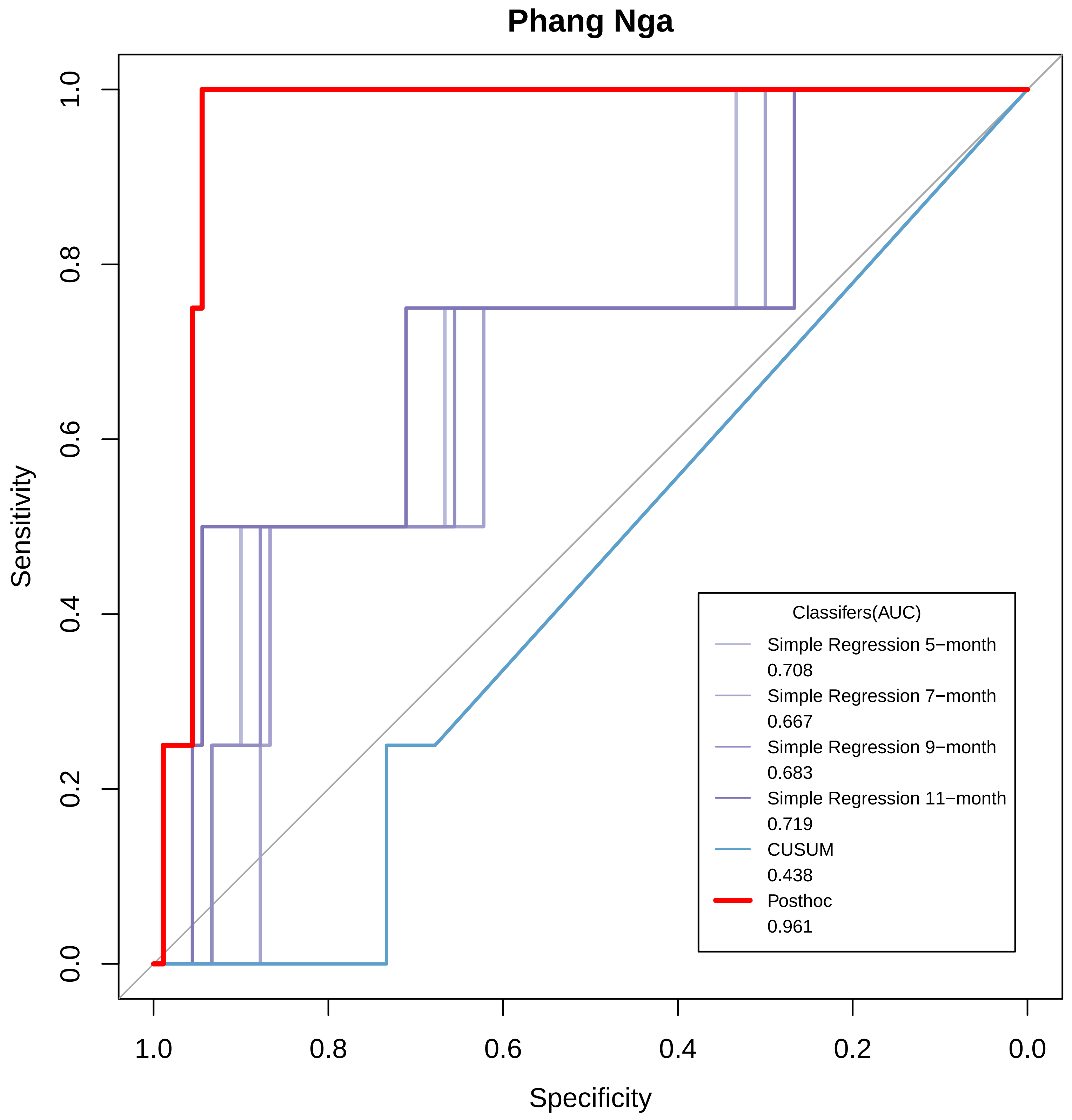
Phang Nga
Sensitivity
Specificity
1.0
0.8
0.6
0.4
0.2
0.0
Classifers(AUC)
Simple Regression 5−month
0.708
Simple Regression 7−month
0.667
Simple Regression 9−month
0.683
Simple Regression 11−month
0.719
CUSUM
0.438
Posthoc
0.961

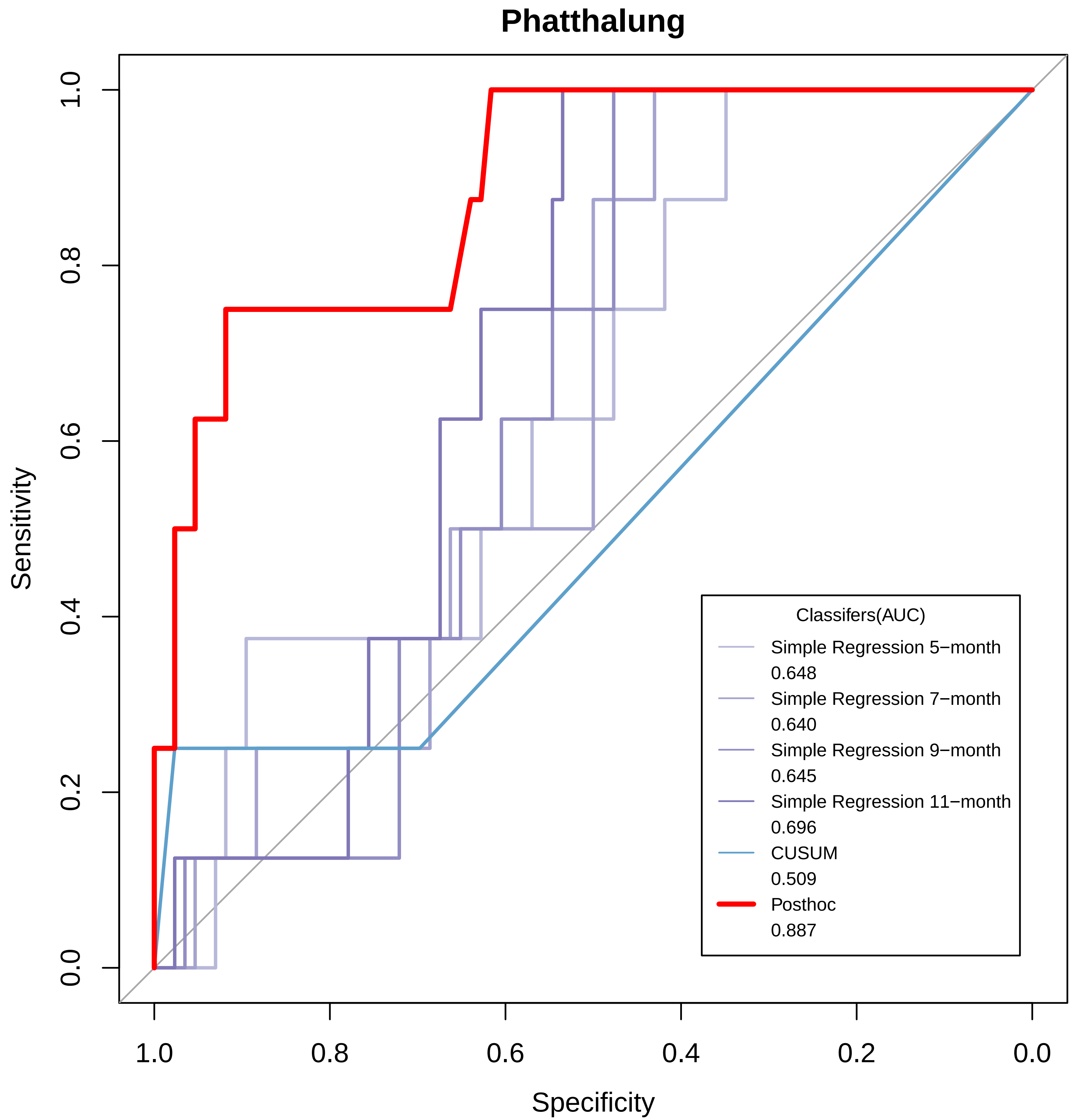

Phatthalung
Sensitivity
Specificity
1.0
0.8
0.6
0.4
0.2
0.0
Classifers(AUC)
Simple Regression 5−month
0.648
Simple Regression 7−month
0.640
Simple Regression 9−month
0.645
Simple Regression 11−month
0.696
CUSUM
0.509
Posthoc
0.887

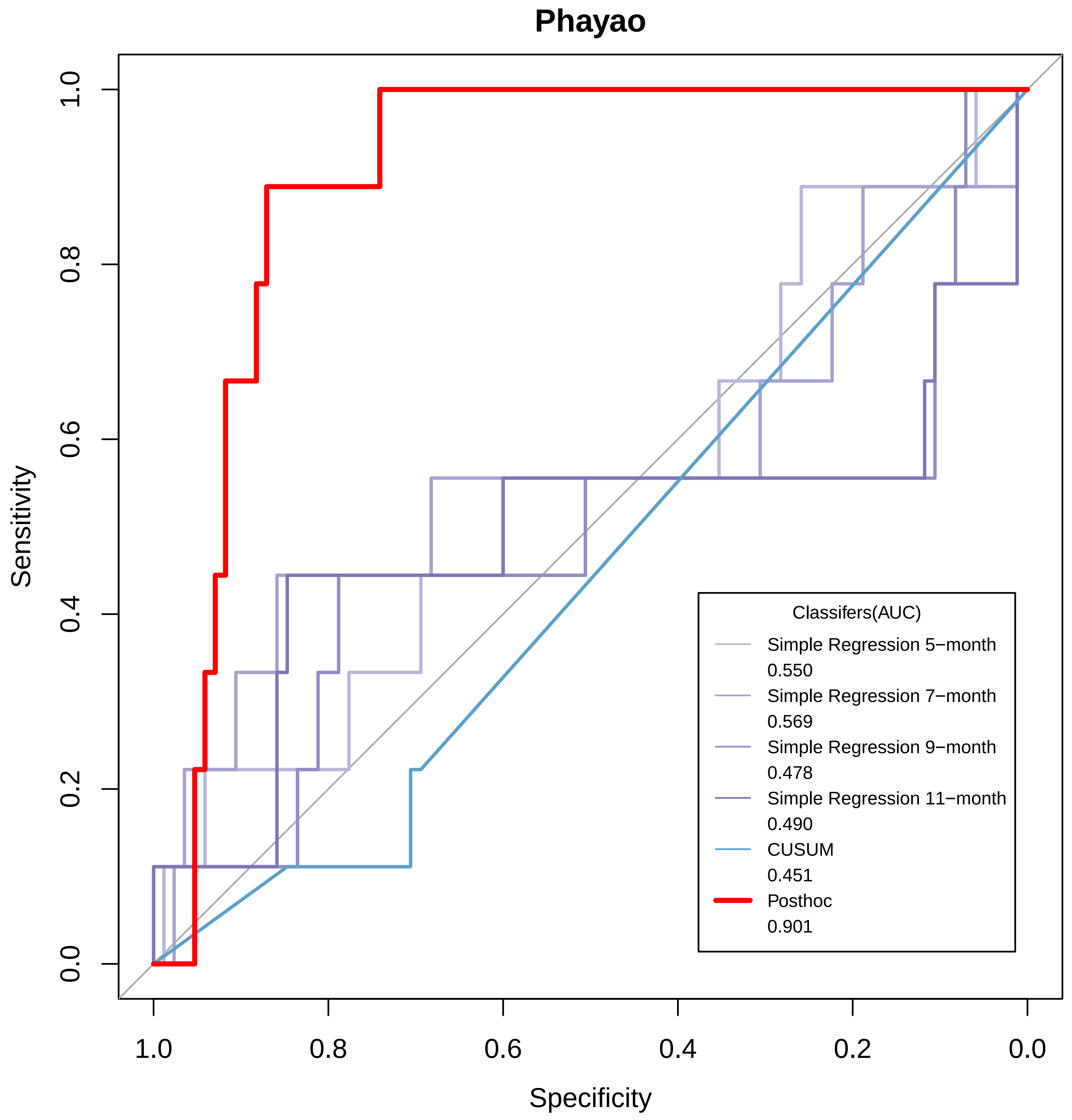

Phayao
Sensitivity
Specificity
1.0
0.8
0.6
0.4
0.2
0.0
Classifers(AUC)
Simple Regression 5−month
0.550
Simple Regression 7−month
0.569
Simple Regression 9−month
0.478
Simple Regression 11−month
0.490
CUSUM
0.451
Posthoc
0.901

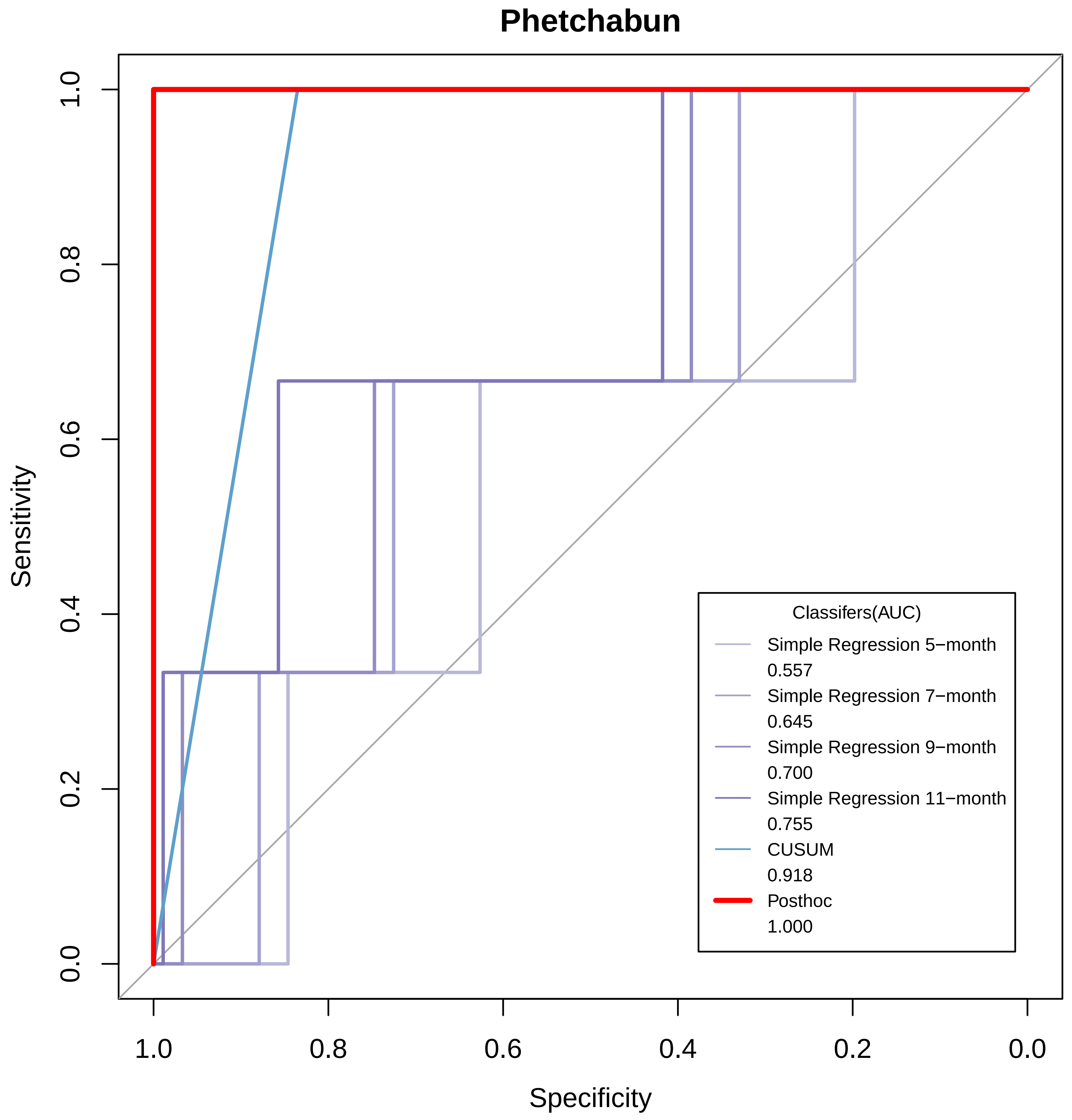

Phetchabun
Sensitivity
Specificity
1.0
0.8
0.6
0.4
0.2
0.0
Classifers(AUC)
Simple Regression 5−month
0.557
Simple Regression 7−month
0.645
Simple Regression 9−month
0.700
Simple Regression 11−month
0.755
CUSUM
0.918
Posthoc
1.000

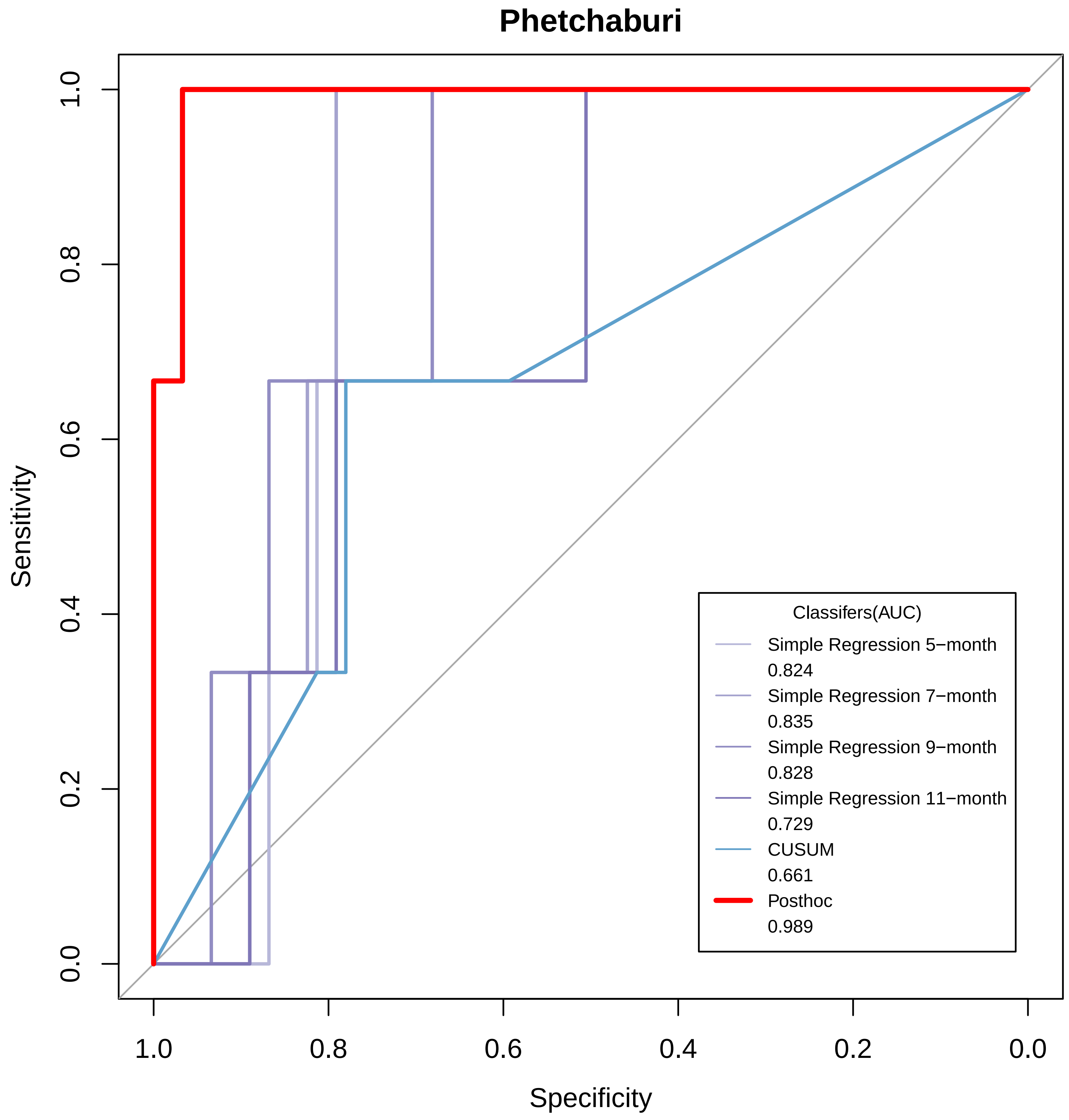

Phetchaburi
Sensitivity
Specificity
1.0
0.8
0.6
0.4
0.2
0.0
Classifers(AUC)
Simple Regression 5−month
0.824
Simple Regression 7−month
0.835
Simple Regression 9−month
0.828
Simple Regression 11−month
0.729
CUSUM
0.661
Posthoc
0.989

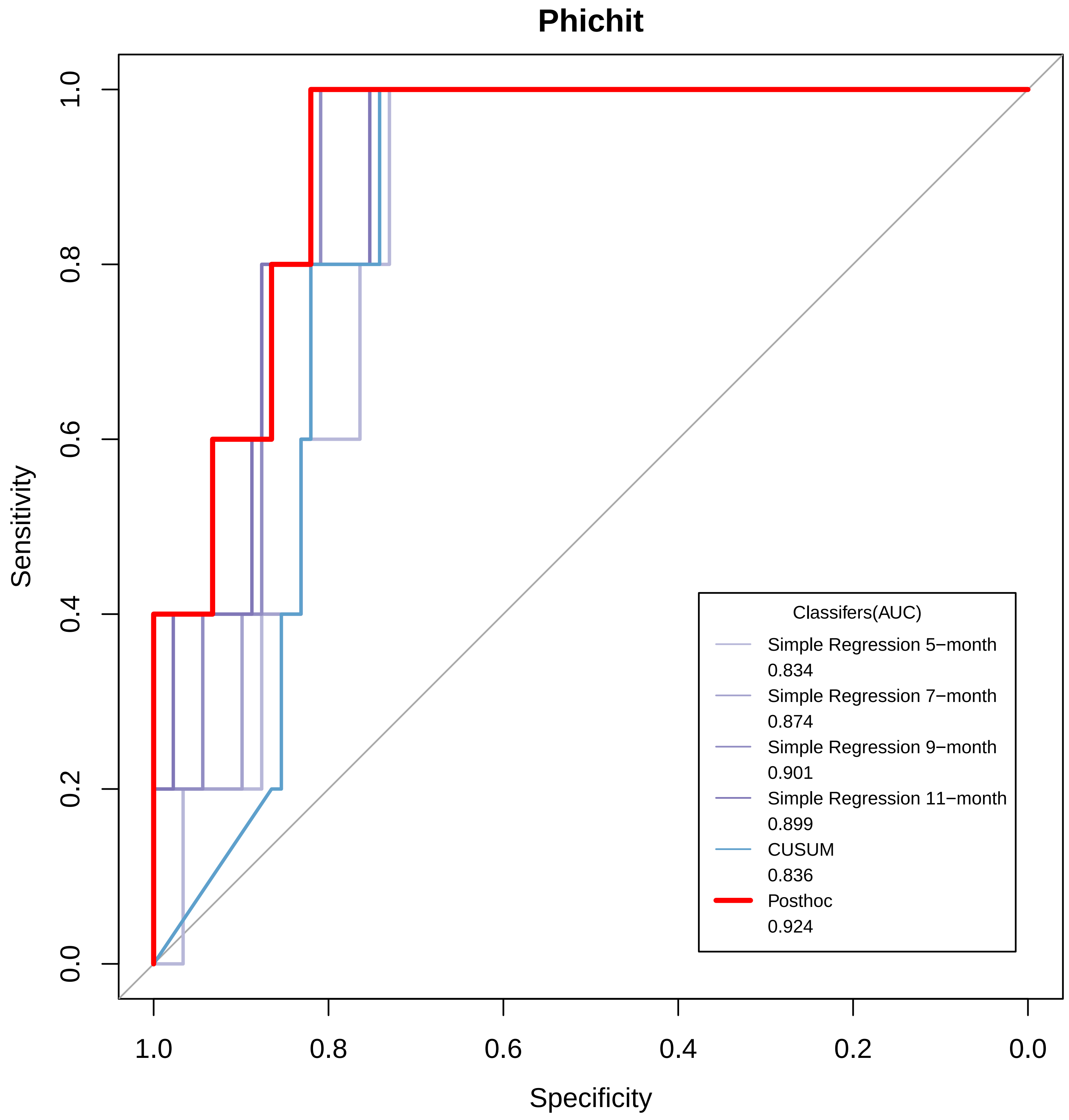
Phichit
Sensitivity
Specificity
1.0
0.8
0.6
0.4
0.2
0.0
Classifers(AUC)
Simple Regression 5−month
0.834
Simple Regression 7−month
0.874
Simple Regression 9−month
0.901
Simple Regression 11−month
0.899
CUSUM
0.836
Posthoc
0.924

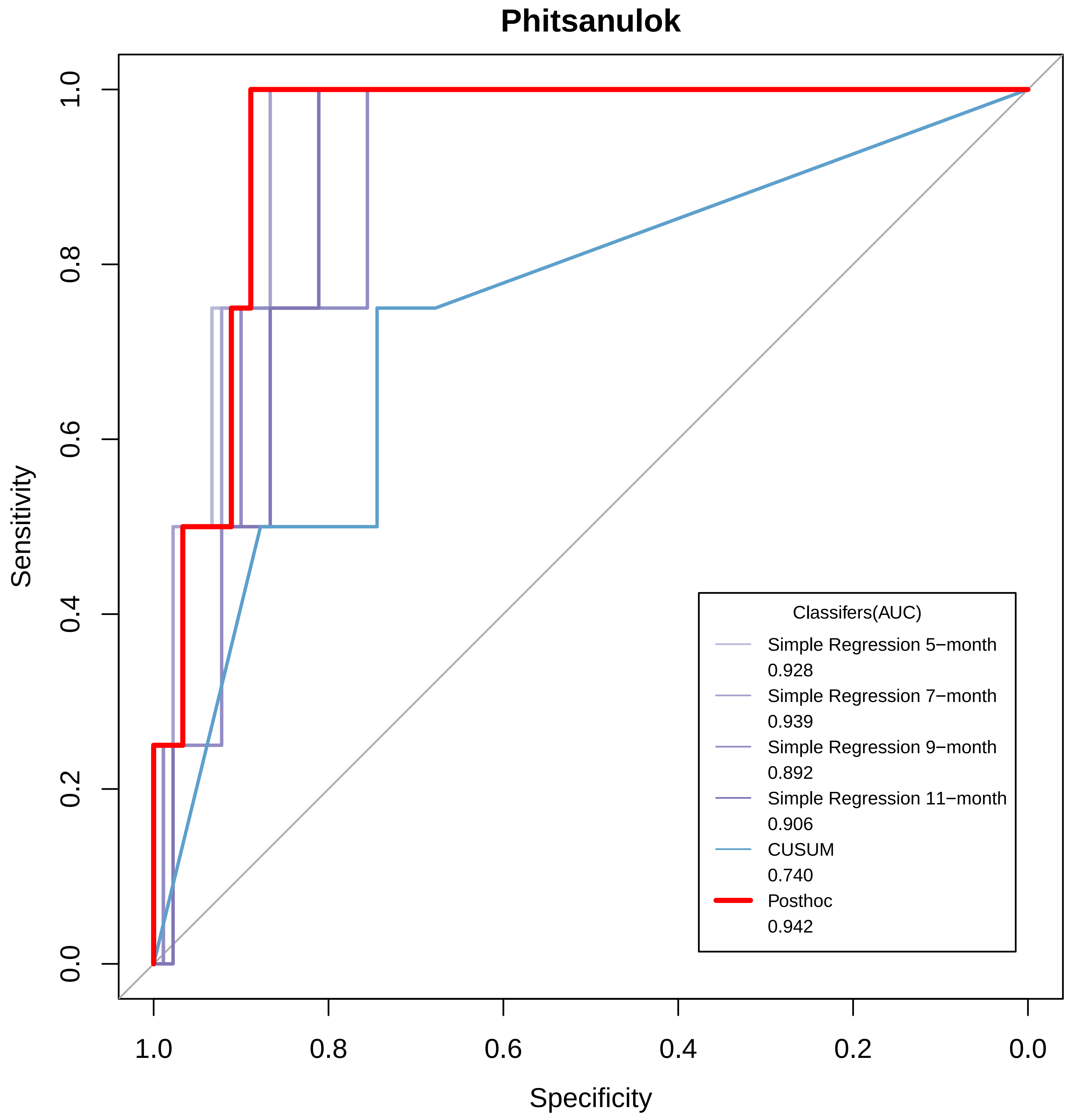

Phitsanulok
Sensitivity
Specificity
0.0
0.2
0.4
0.6
0.8
1.0
1.0
0.8
0.6
0.4
0.2
0.0
Classifers(AUC)
Simple Regression 5−month
0.928
Simple Regression 7−month
0.939
Simple Regression 9−month
0.892
Simple Regression 11−month
0.906
CUSUM
0.740
Posthoc
0.942

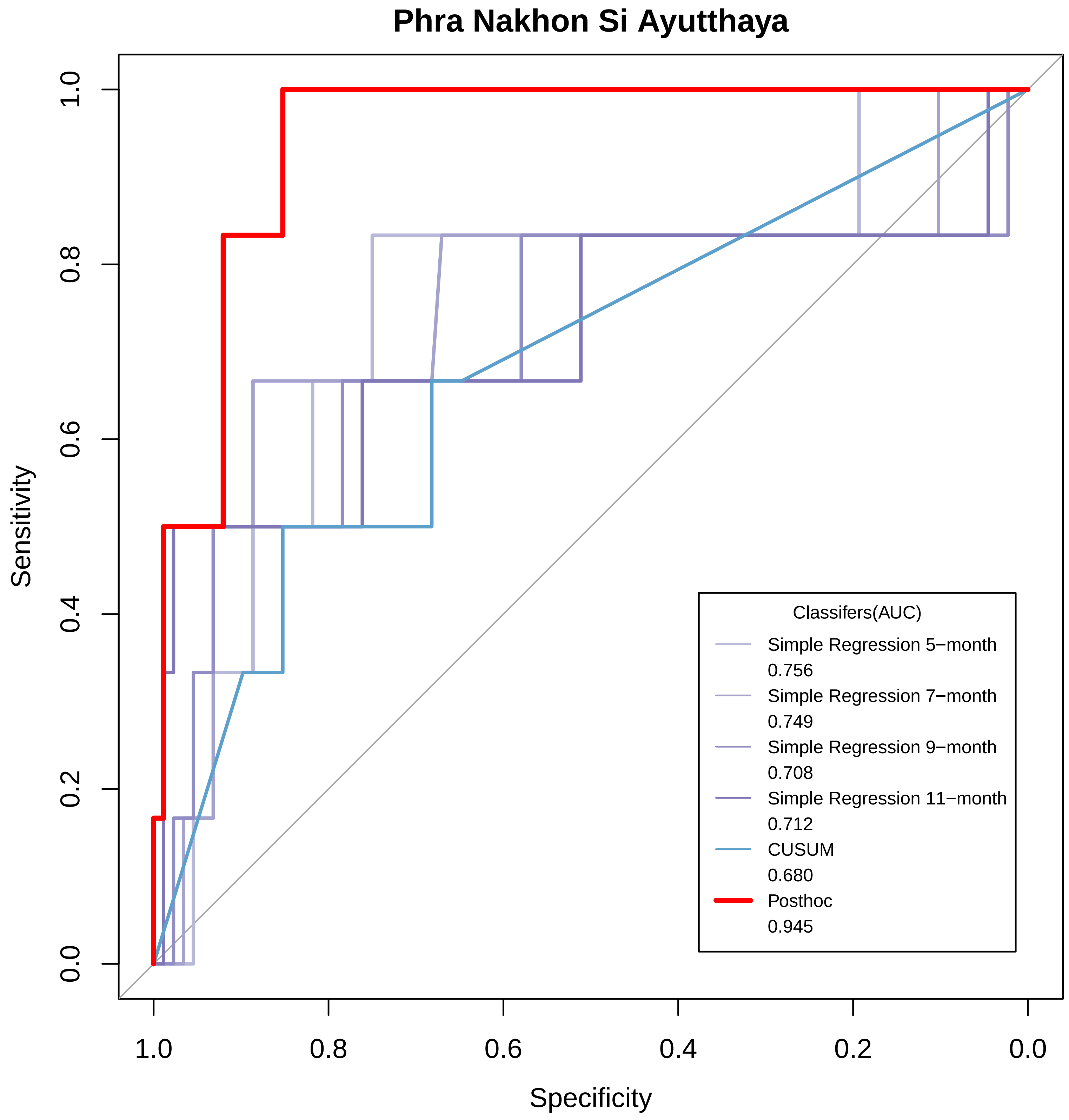
Phra Nakhon Si Ayutthaya
Sensitivity
Specificity
1.0
0.8
0.6
0.4
0.2
0.0
Classifers(AUC)
Simple Regression 5−month
0.756
Simple Regression 7−month
0.749
Simple Regression 9−month
0.708
Simple Regression 11−month
0.712
CUSUM
0.680
Posthoc
0.945

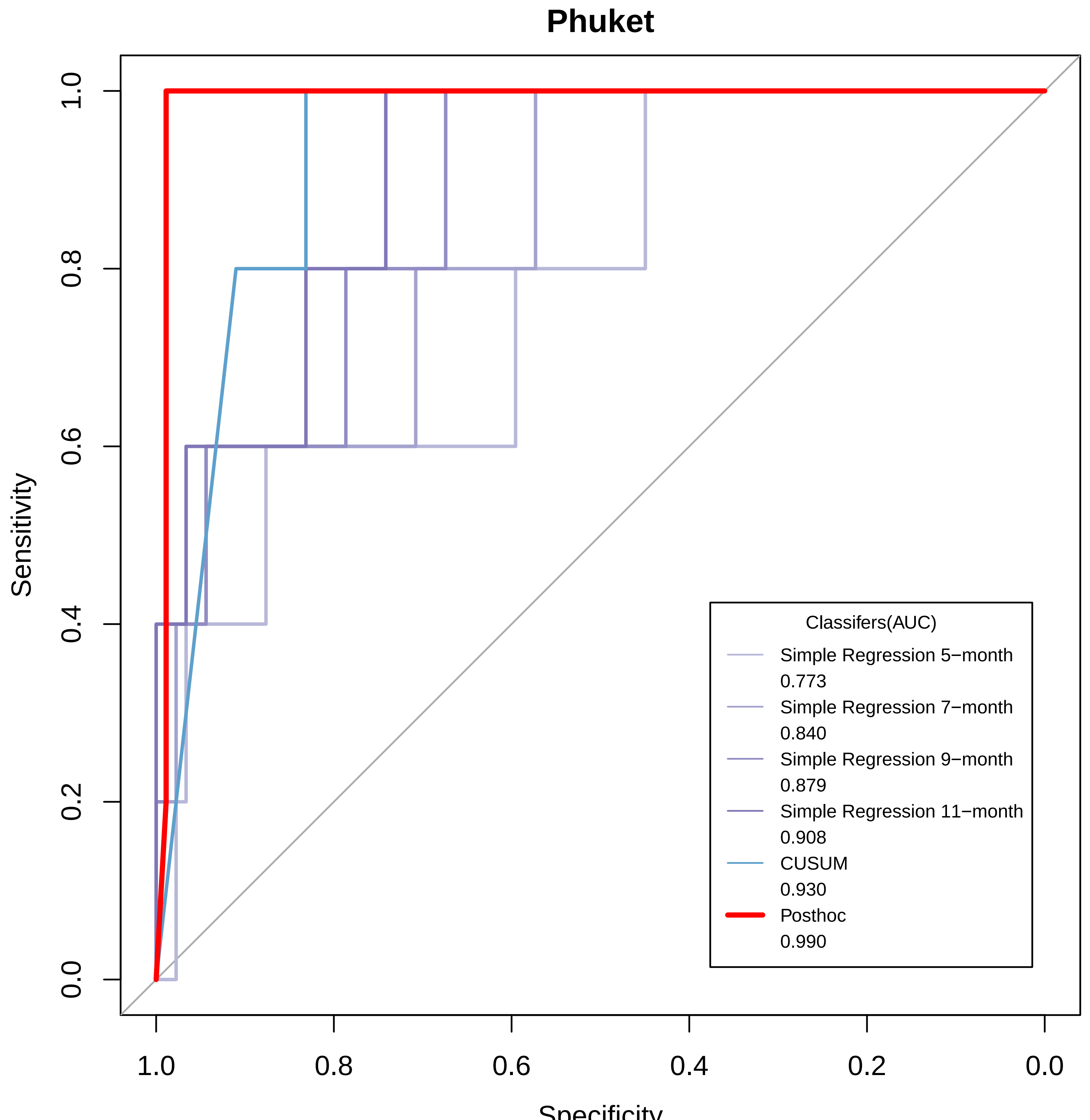
Phuket
Sensitivity
Specificity
1.0
0.8
0.6
0.4
0.2
0.0
Classifers(AUC)
Simple Regression 5−month
0.773
Simple Regression 7−month
0.840
Simple Regression 9−month
0.879
Simple Regression 11−month
0.908
CUSUM
0.930
Posthoc
0.990

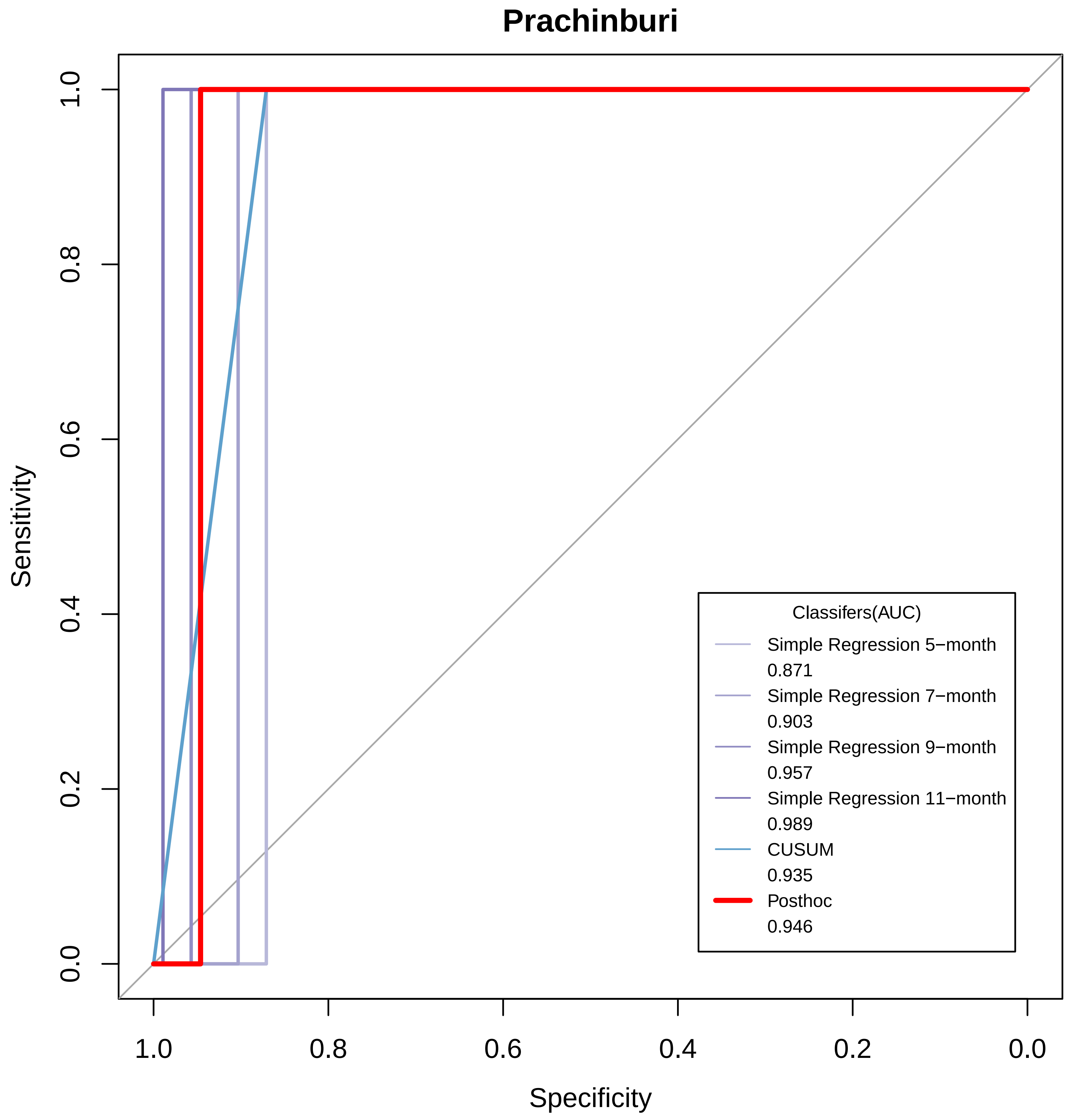
Prachinburi
Sensitivity
Specificity
1.0
0.8
0.6
0.4
0.2
0.0
Classifers(AUC)
Simple Regression 5−month
0.871
Simple Regression 7−month
0.903
Simple Regression 9−month
0.957
Simple Regression 11−month
0.989
CUSUM
0.935
Posthoc
0.946

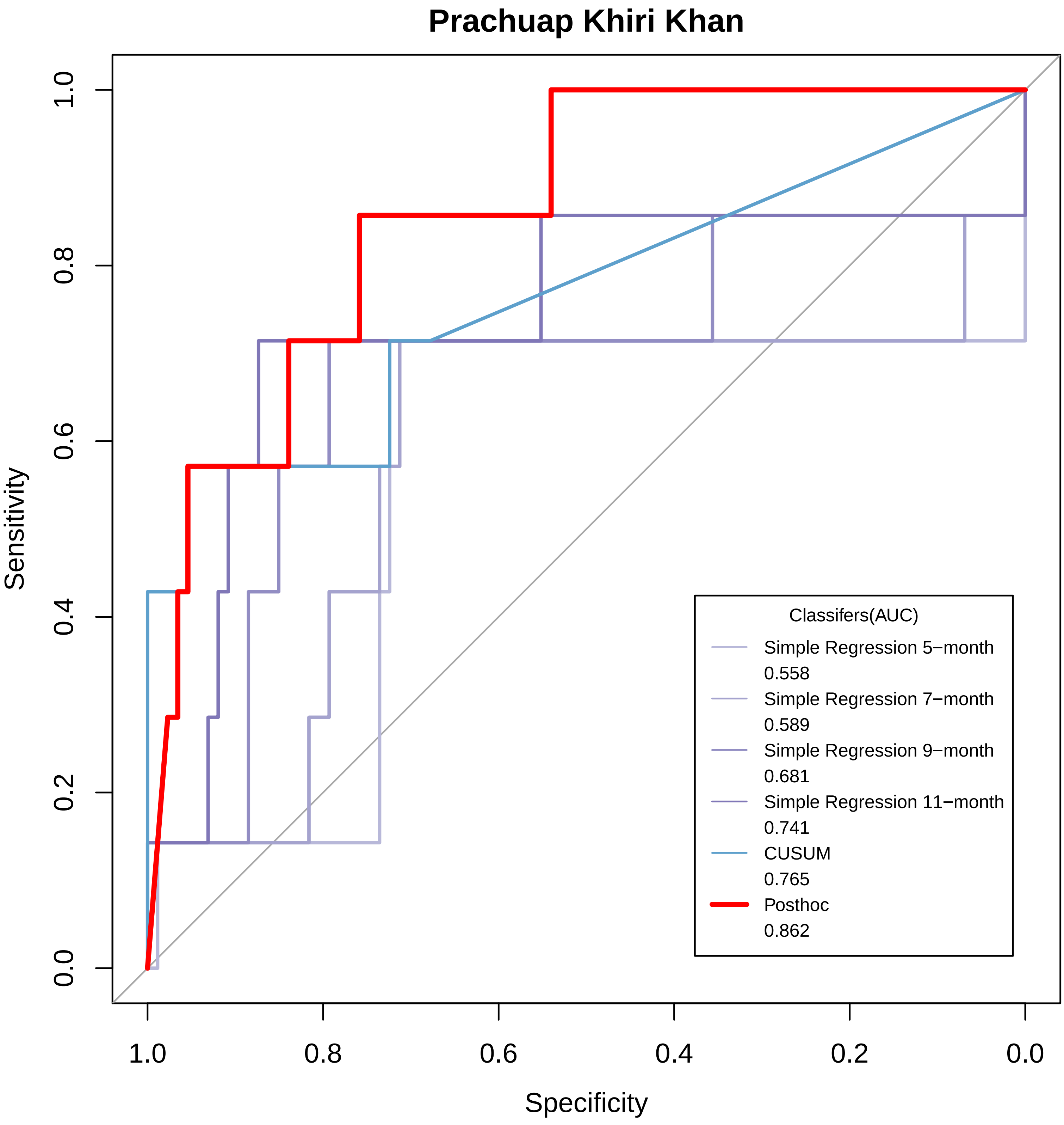
Prachuap Khiri Khan
Sensitivity
Specificity
1.0
0.8
0.6
0.4
0.2
0.0
Classifers(AUC)
Simple Regression 5−month
0.558
Simple Regression 7−month
0.589
Simple Regression 9−month
0.681
Simple Regression 11−month
0.741
CUSUM
0.765
Posthoc
0.862

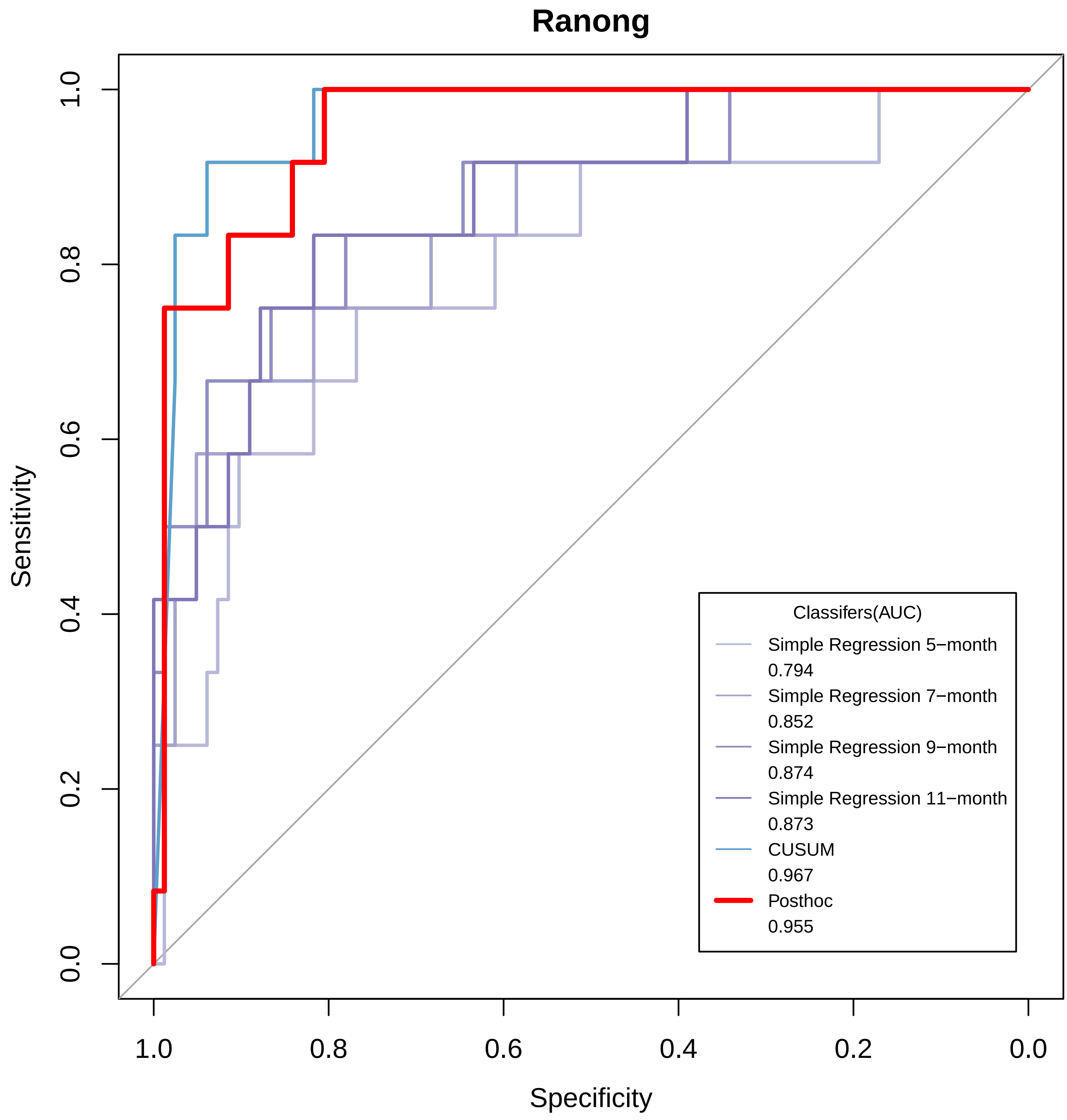

Ranong
Sensitivity
Specificity
1.0
0.8
0.6
0.4
0.2
0.0
Classifers(AUC)
Simple Regression 5−month
0.794
Simple Regression 7−month
0.852
Simple Regression 9−month
0.874
Simple Regression 11−month
0.873
CUSUM
0.967
Posthoc
0.955

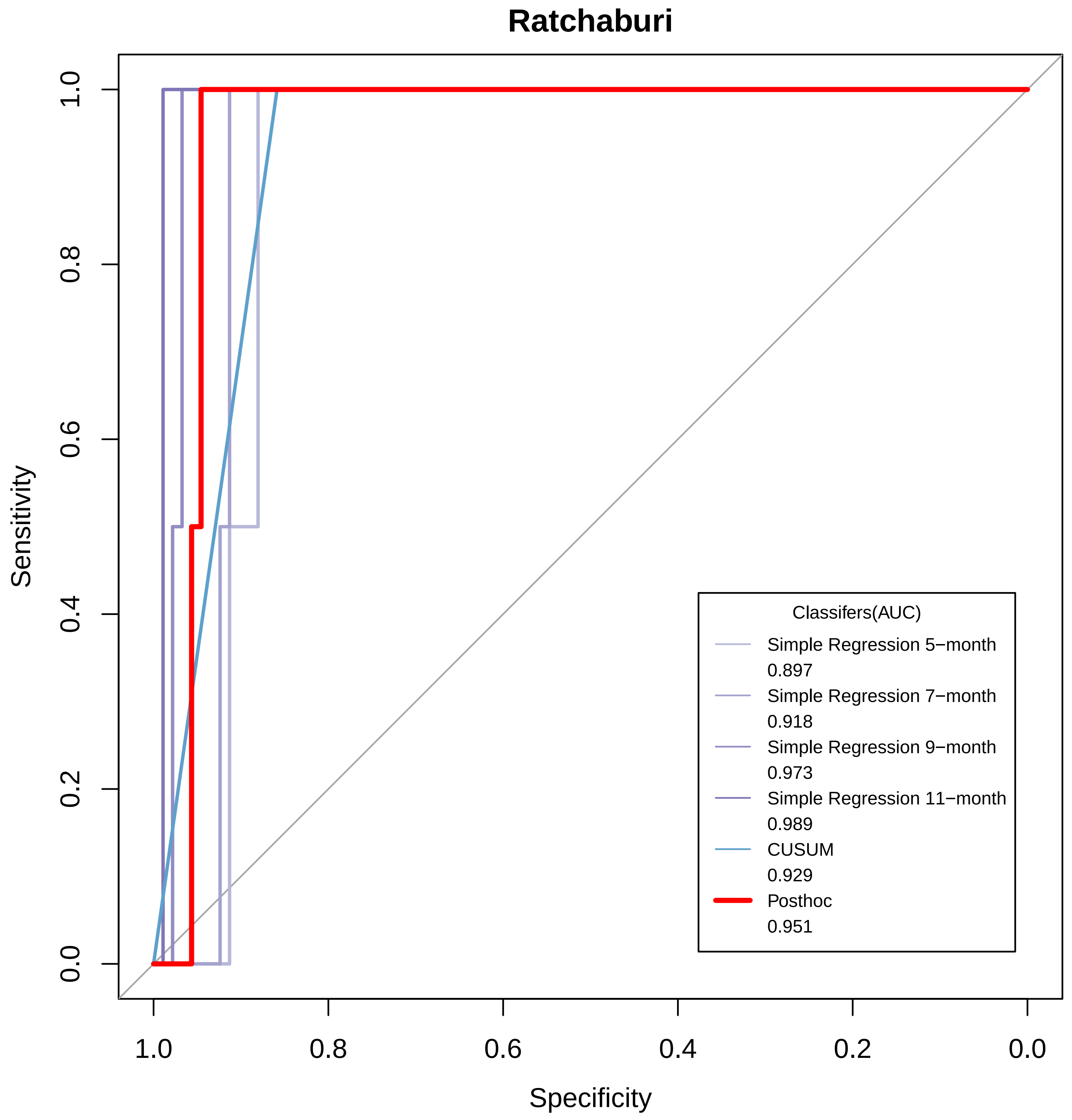
Ratchaburi
Sensitivity
Specificity
1.0
0.8
0.6
0.4
0.2
0.0
Classifers(AUC)
Simple Regression 5−month
0.897
Simple Regression 7−month
0.918
Simple Regression 9−month
0.973
Simple Regression 11−month
0.989
CUSUM
0.929
Posthoc
0.951

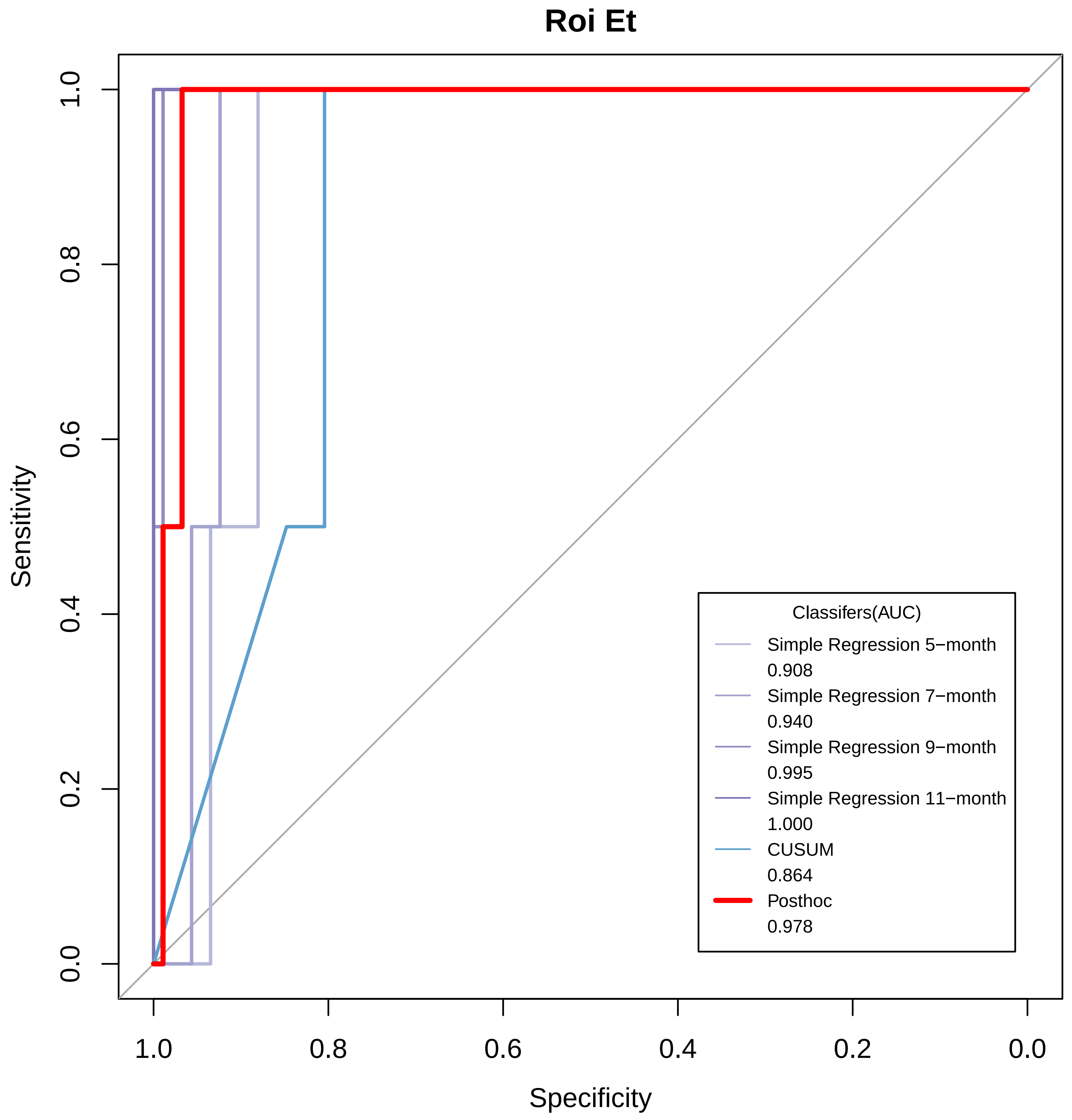

Roi Et
Sensitivity
Specificity
1.0
0.8
0.6
0.4
0.2
0.0
1.0
0.8
0.6
0.4
0.2
0.0
Classifers(AUC)
Simple Regression 5−month
0.908
Simple Regression 7−month
0.940
Simple Regression 9−month
0.995
Simple Regression 11−month
1.000
CUSUM
0.864
Posthoc
0.978

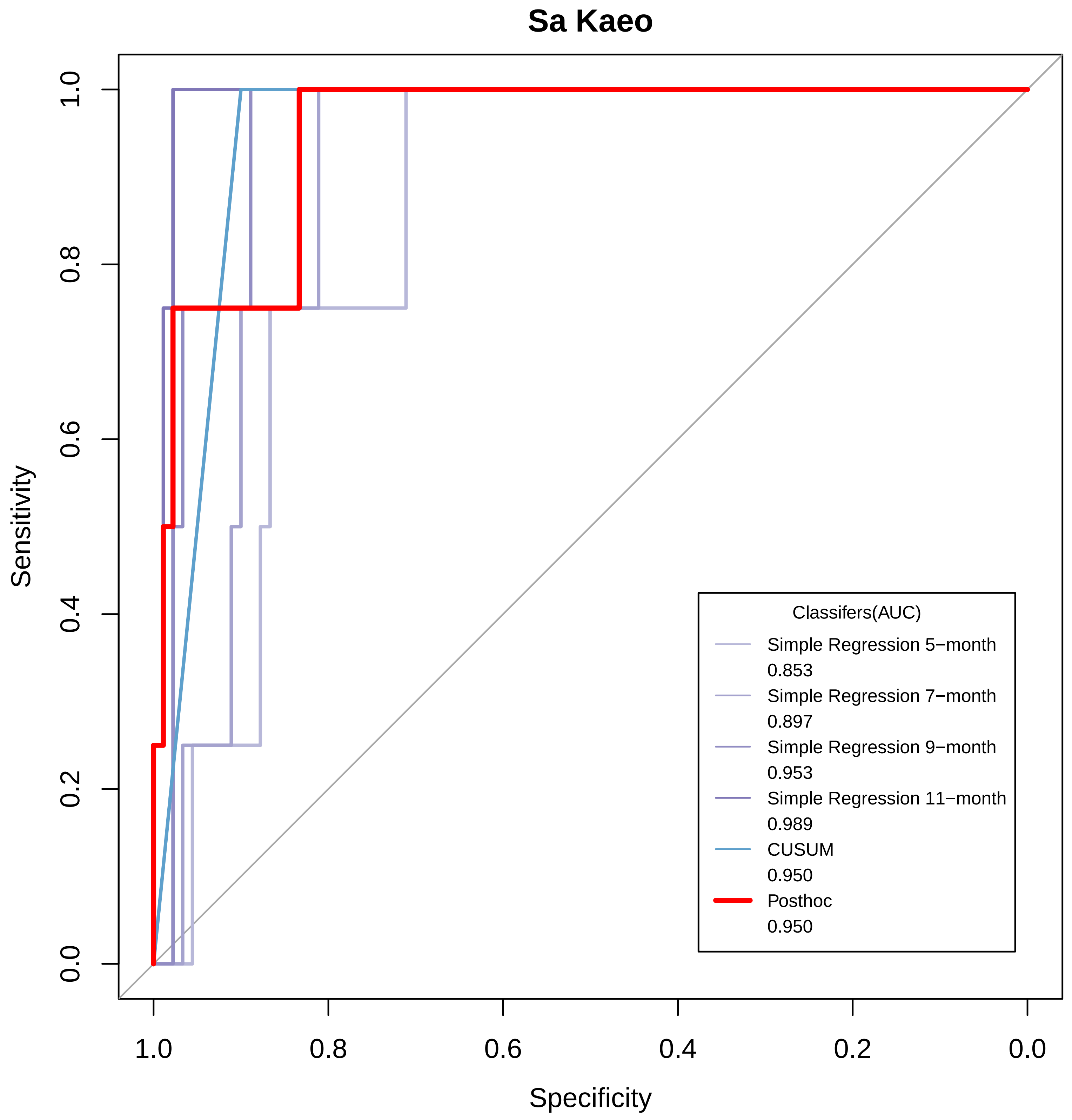

Sa Kaeo
Sensitivity
Specificity
1.0
0.8
0.6
0.4
0.2
0.0
Classifers(AUC)
Simple Regression 5−month
0.853
Simple Regression 7−month
0.897
Simple Regression 9−month
0.953
Simple Regression 11−month
0.989
CUSUM
0.950
Posthoc
0.950

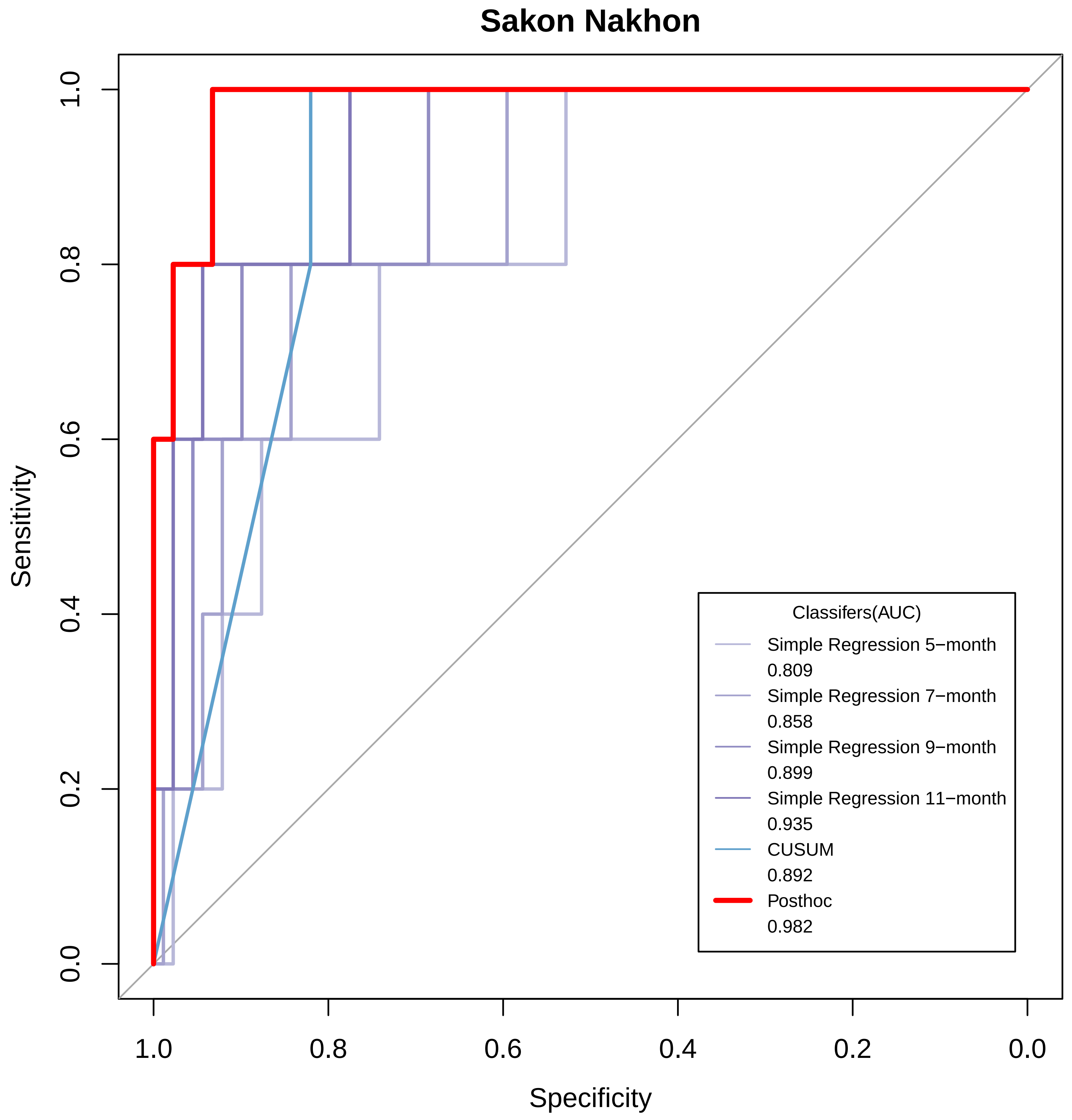
Sakon Nakhon
Sensitivity
Specificity
1.0
0.8
0.6
0.4
0.2
0.0
Classifers(AUC)
Simple Regression 5−month
0.809
Simple Regression 7−month
0.858
Simple Regression 9−month
0.899
Simple Regression 11−month
0.935
CUSUM
0.892
Posthoc
0.982

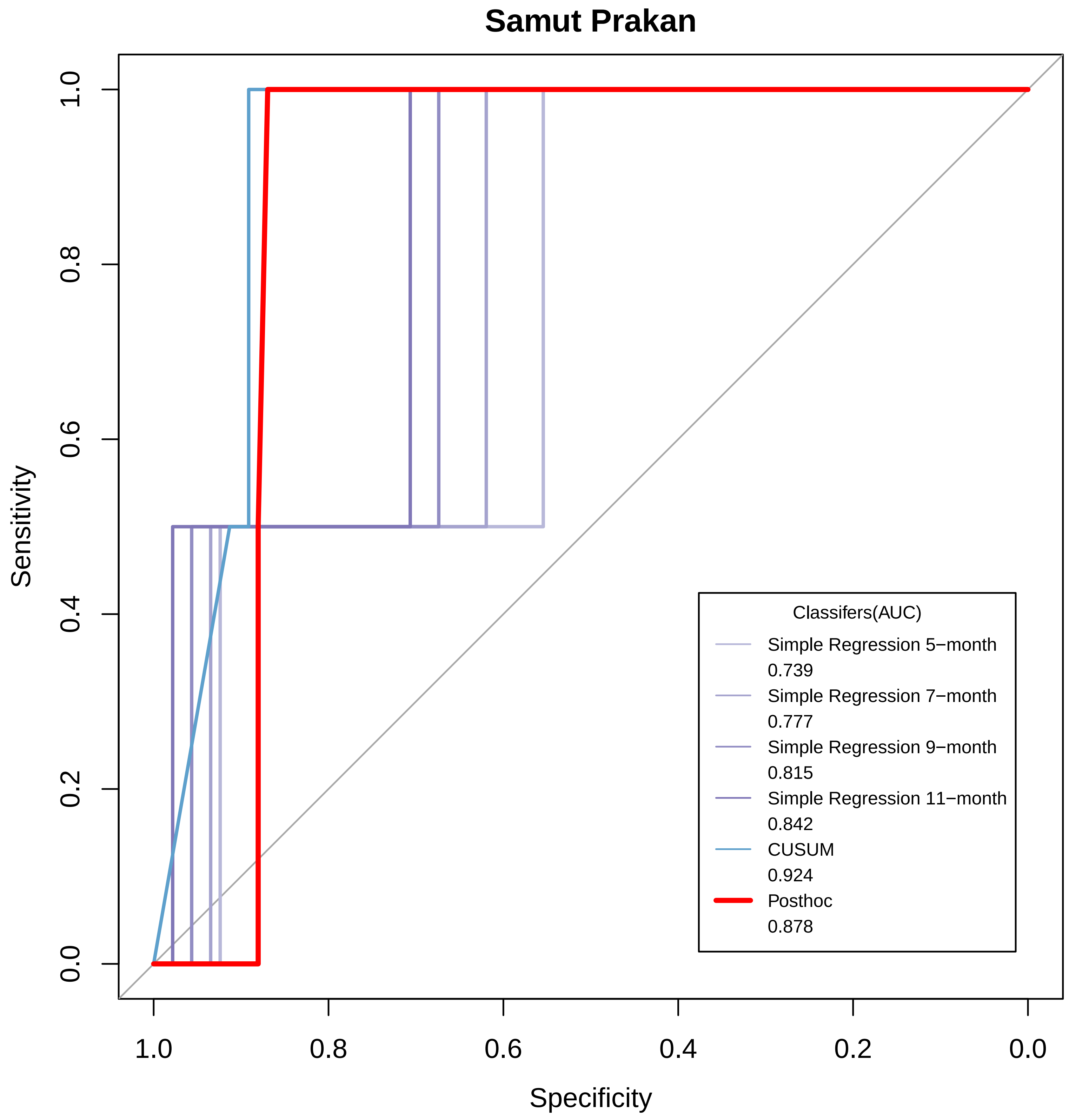
Samut Prakan
Sensitivity
Specificity
1.0
0.8
0.6
0.4
0.2
0.0
Classifers(AUC)
Simple Regression 5−month
0.739
Simple Regression 7−month
0.777
Simple Regression 9−month
0.815
Simple Regression 11−month
0.842
CUSUM
0.924
Posthoc
0.878

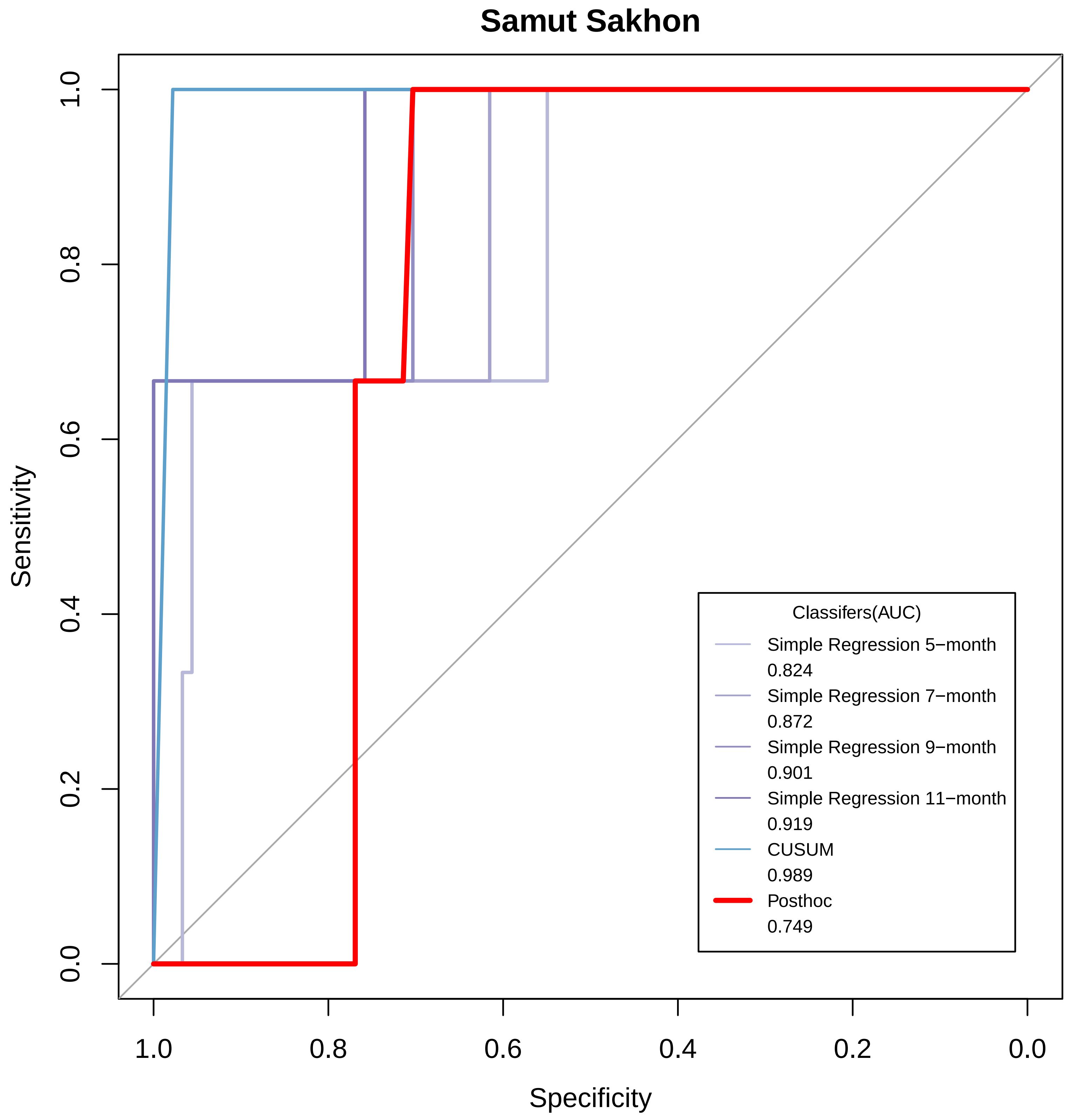

Samut Sakhon
Sensitivity
Specificity
1.0
0.8
0.6
0.4
0.2
0.0
Classifers(AUC)
Simple Regression 5−month
0.824
Simple Regression 7−month
0.872
Simple Regression 9−month
0.901
Simple Regression 11−month
0.919
CUSUM
0.989
Posthoc
0.749

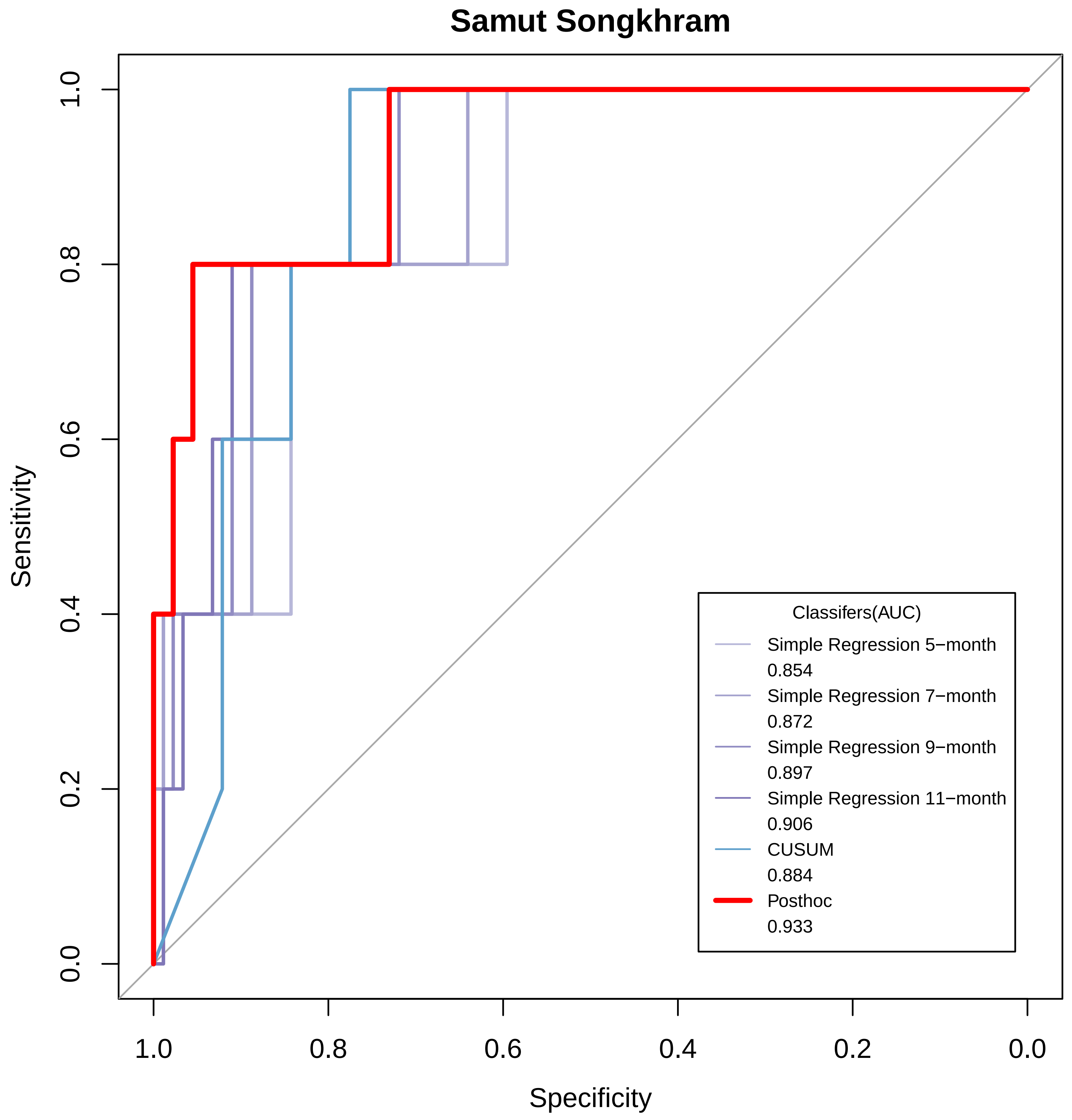

Samut Songkhram
Sensitivity
Specificity
1.0
0.8
0.6
0.4
0.2
0.0
Classifers(AUC)
Simple Regression 5−month
0.854
Simple Regression 7−month
0.872
Simple Regression 9−month
0.897
Simple Regression 11−month
0.906
CUSUM
0.884
Posthoc
0.933

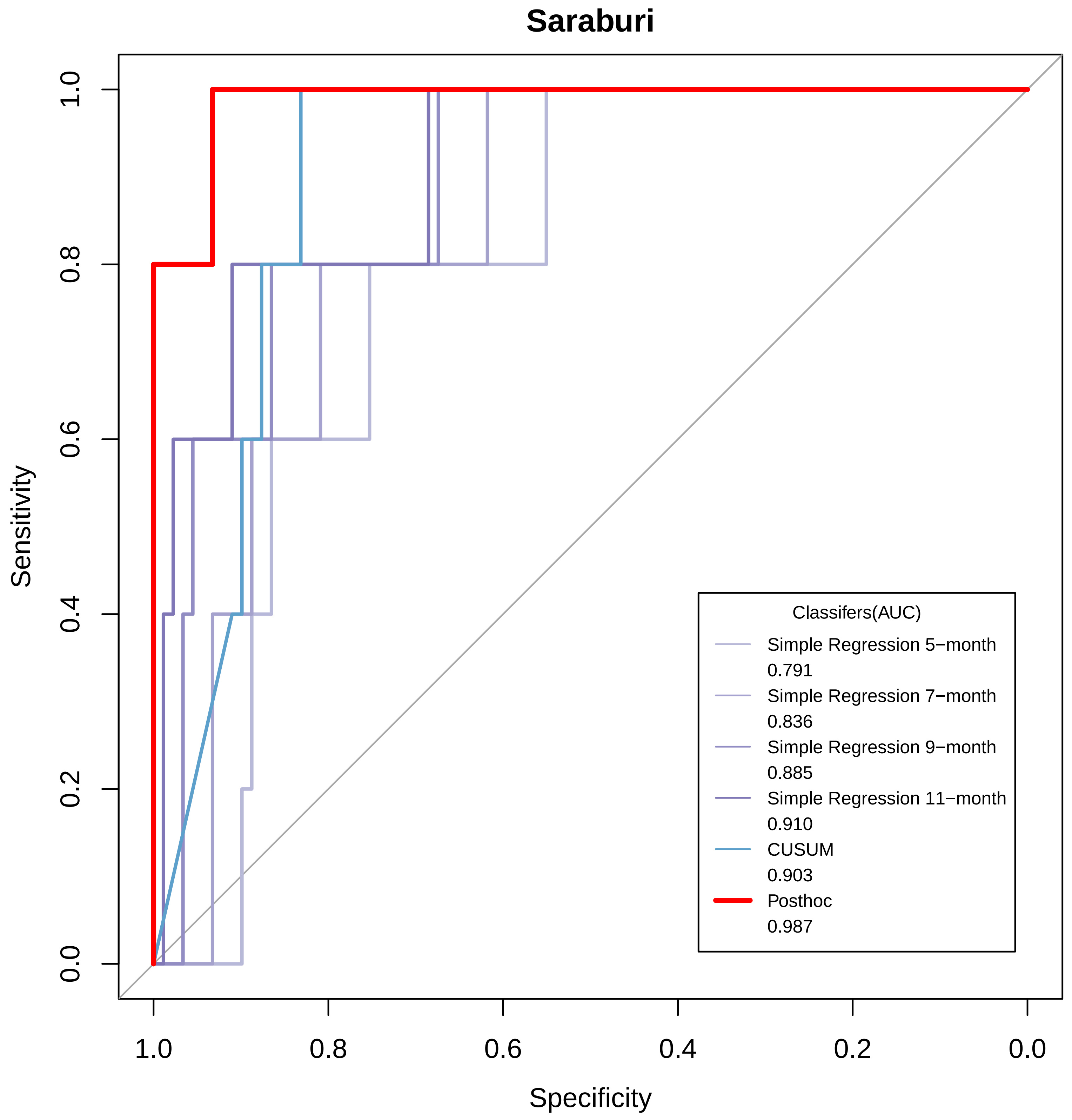

Saraburi
Sensitivity
Specificity
1.0
0.8
0.6
0.4
0.2
0.0
Classifers(AUC)
Simple Regression 5−month
0.791
Simple Regression 7−month
0.836
Simple Regression 9−month
0.885
Simple Regression 11−month
0.910
CUSUM
0.903
Posthoc
0.987

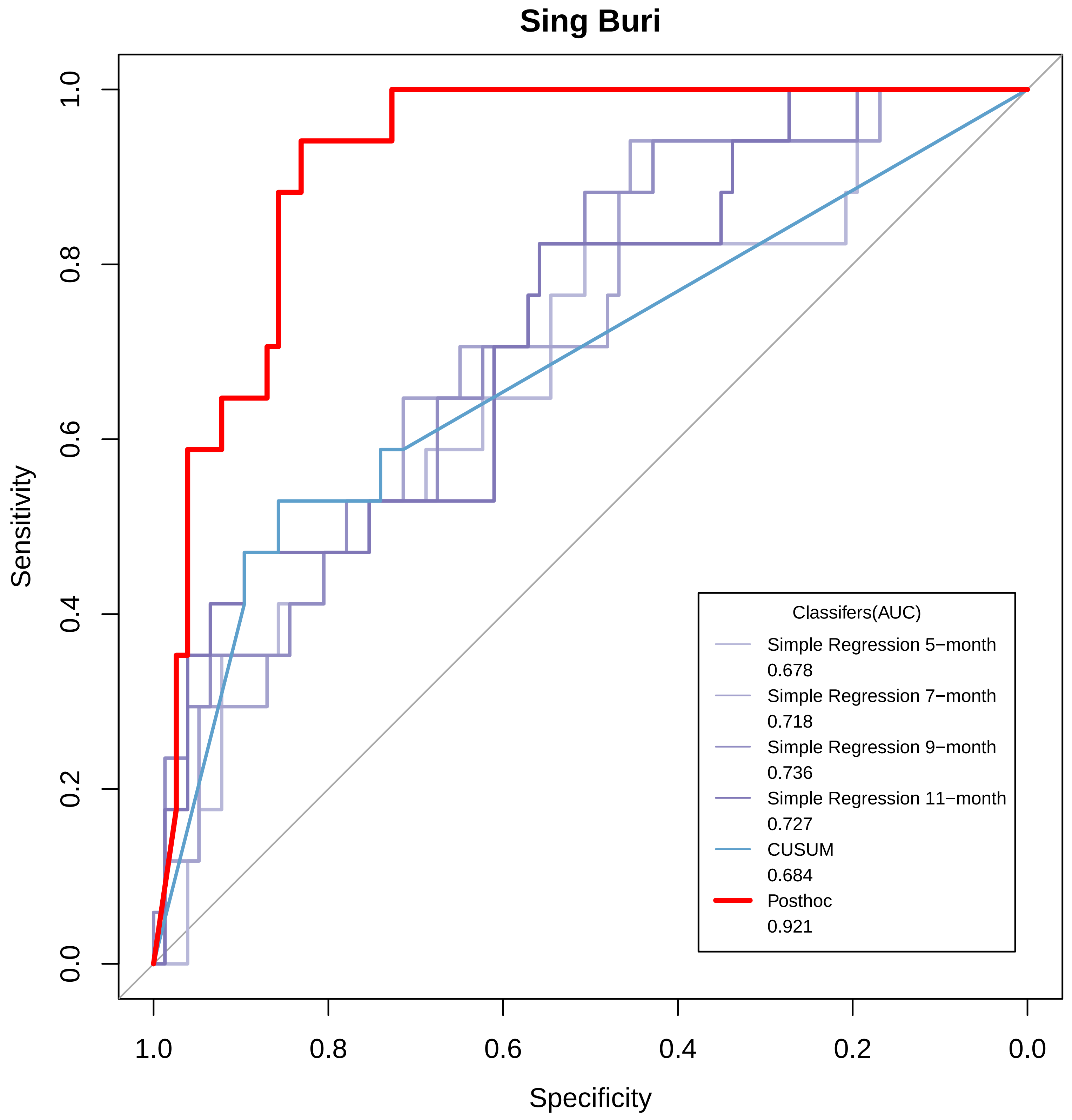
Sing Buri
Sensitivity
Specificity
1.0
0.8
0.6
0.4
0.2
0.0
Classifers(AUC)
Simple Regression 5−month
0.678
Simple Regression 7−month
0.718
Simple Regression 9−month
0.736
Simple Regression 11−month
0.727
CUSUM
0.684
Posthoc
0.921

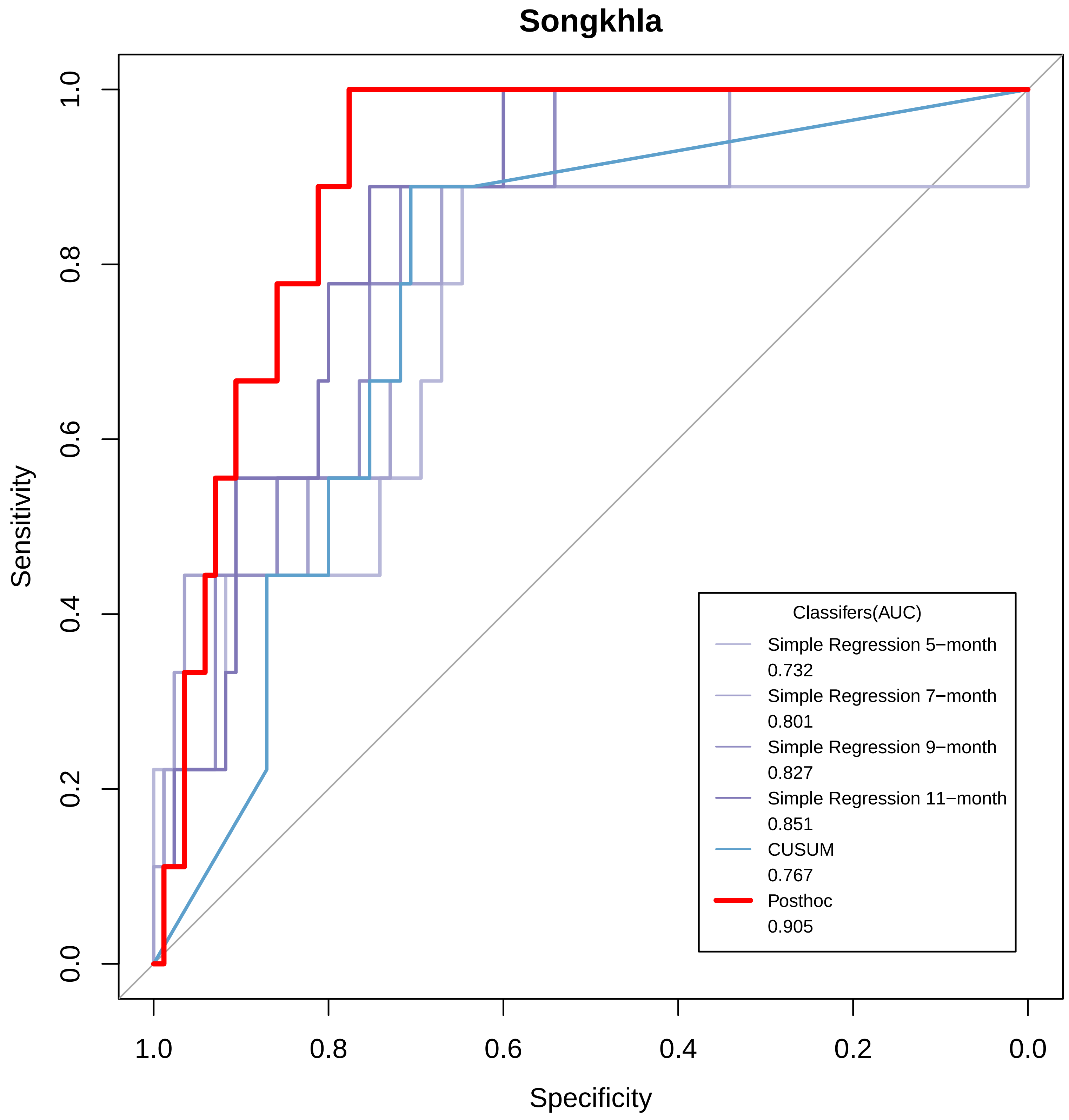
Songkhla
Sensitivity
Specificity
1.0
0.8
0.6
0.4
0.2
0.0
Classifers(AUC)
Simple Regression 5−month
0.732
Simple Regression 7−month
0.801
Simple Regression 9−month
0.827
Simple Regression 11−month
0.851
CUSUM
0.767
Posthoc
0.905

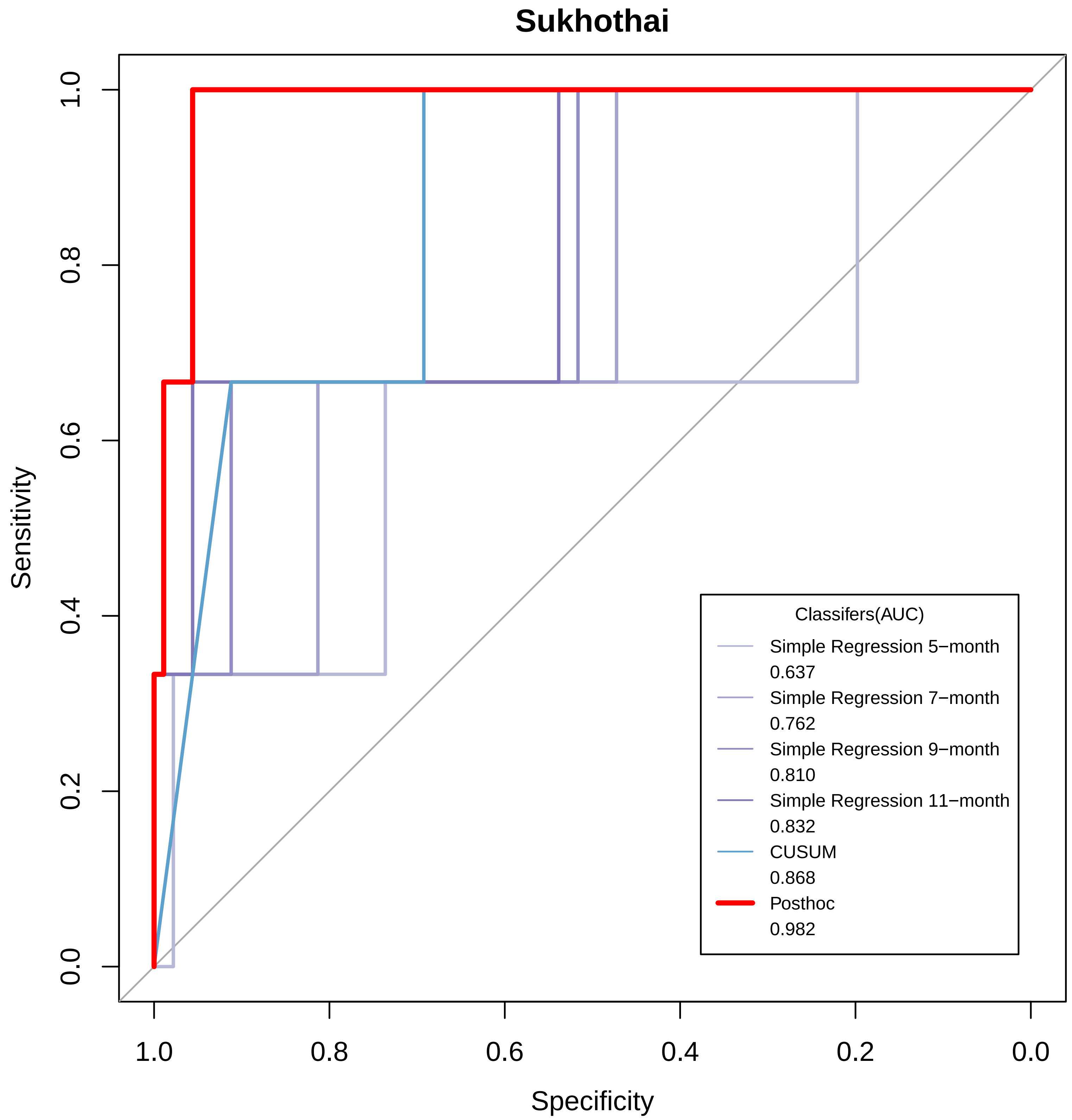
Sukhothai
Sensitivity
Specificity
1.0
0.8
0.6
0.4
0.2
0.0
Classifers(AUC)
Simple Regression 5−month
0.637
Simple Regression 7−month
0.762
Simple Regression 9−month
0.810
Simple Regression 11−month
0.832
CUSUM
0.868
Posthoc
0.982

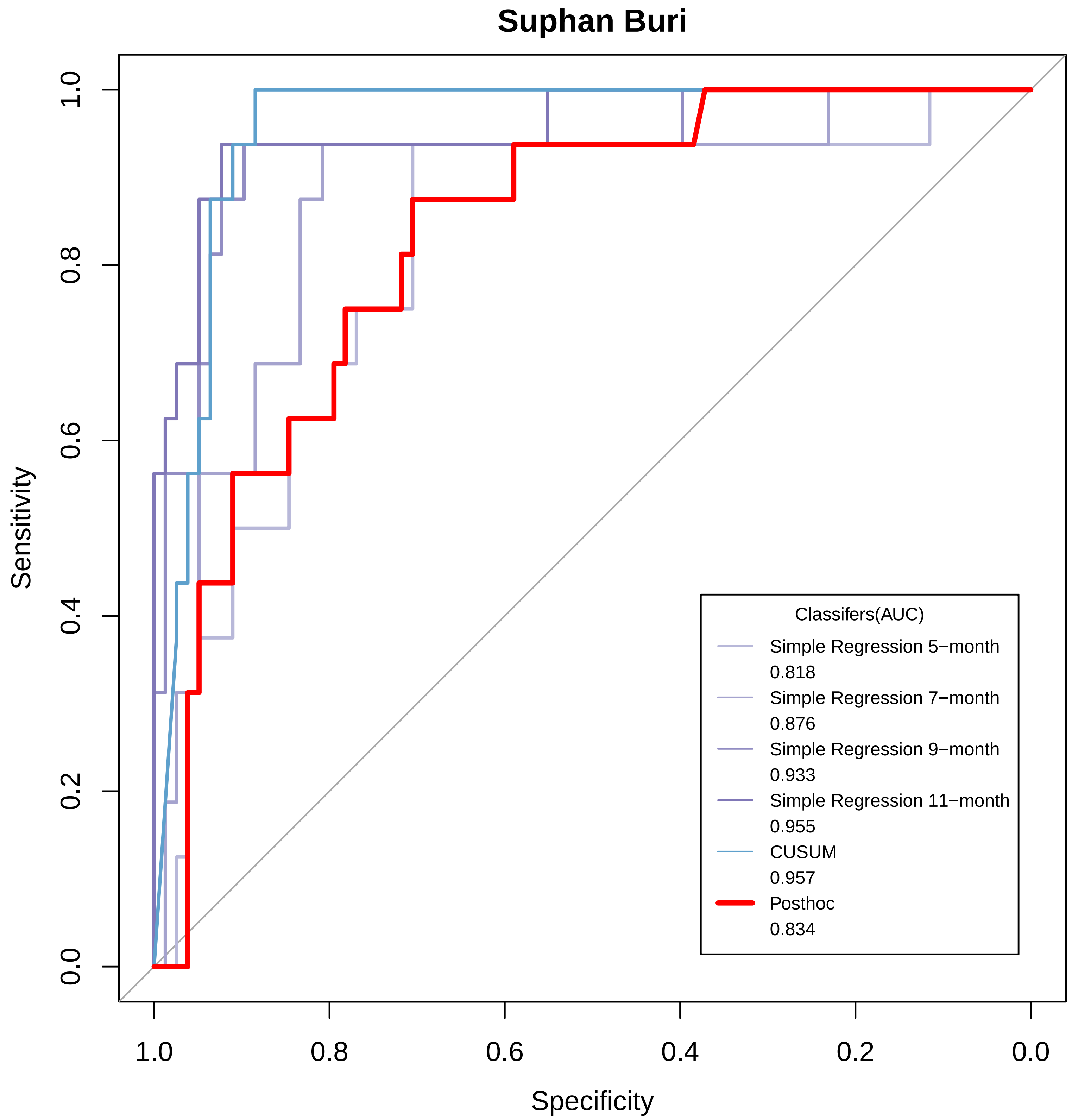

Suphan Buri
Sensitivity
Specificity
1.0
0.8
0.6
0.4
0.2
0.0
Classifers(AUC)
Simple Regression 5−month
0.818
Simple Regression 7−month
0.876
Simple Regression 9−month
0.933
Simple Regression 11−month
0.955
CUSUM
0.957
Posthoc
0.834

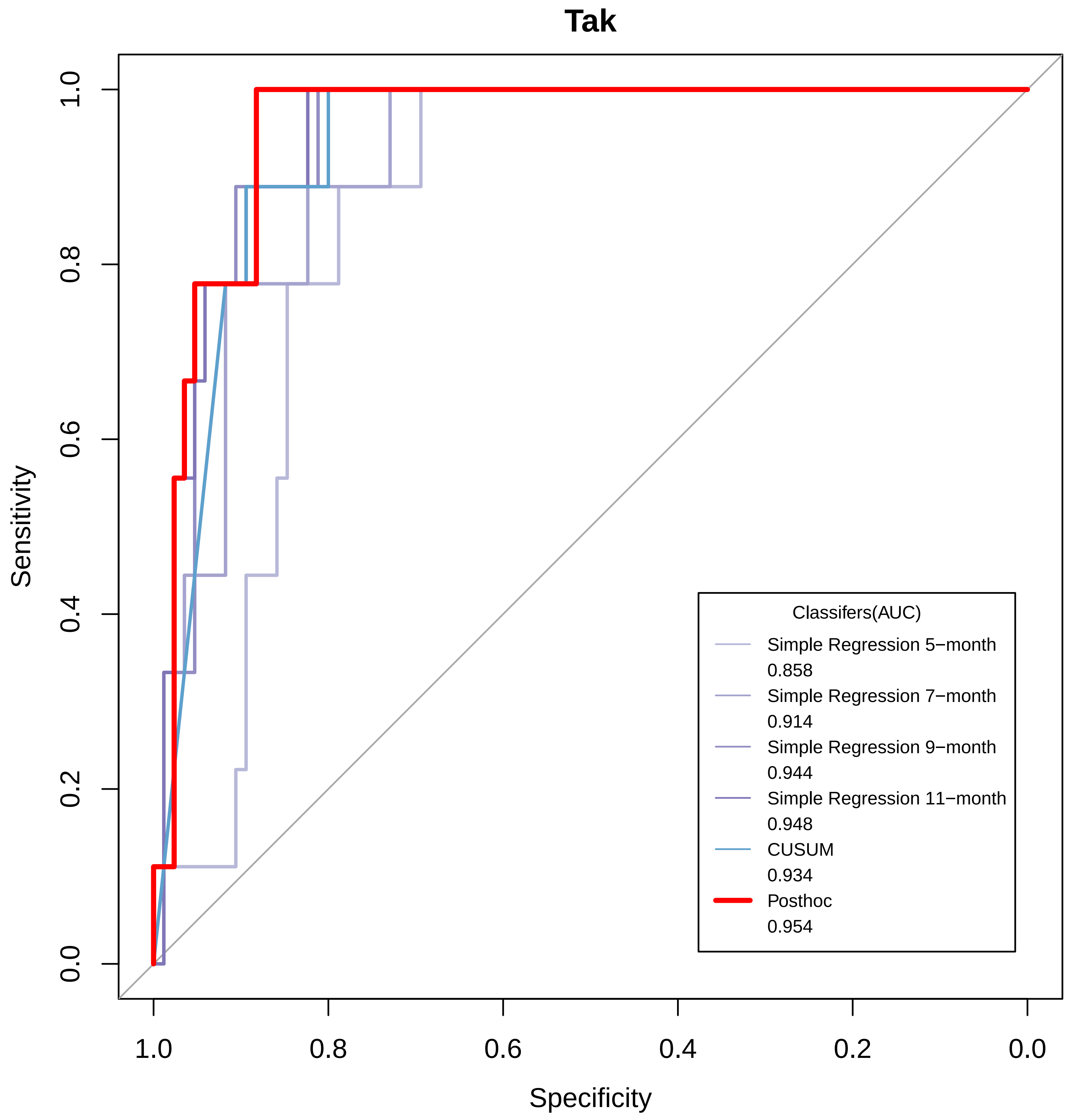

Tak
Sensitivity
Specificity
1.0
0.8
0.6
0.4
0.2
0.0
Classifers(AUC)
Simple Regression 5−month
0.858
Simple Regression 7−month
0.914
Simple Regression 9−month
0.944
Simple Regression 11−month
0.948
CUSUM
0.934
Posthoc
0.954

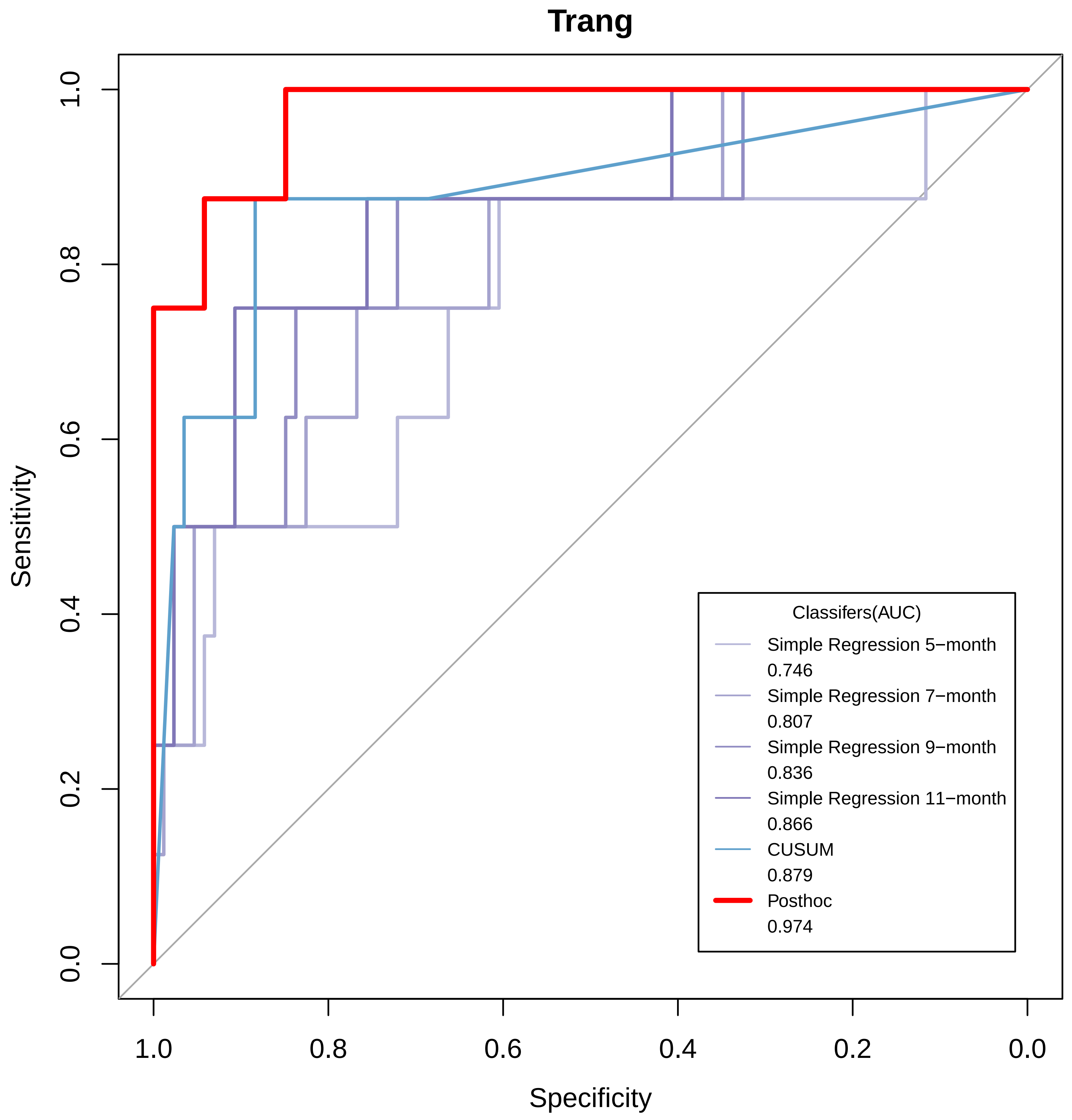

Trang
1.0
0.8
0.6
0.4
0.2
0.0
Sensitivity
1.0
0.8
0.6
0.4
0.2
0.0
Specificity
Classifers(AUC)
Simple Regression 5−month
0.746
Simple Regression 7−month
0.807
Simple Regression 9−month
0.836
Simple Regression 11−month
0.866
CUSUM
0.879
Posthoc
0.974

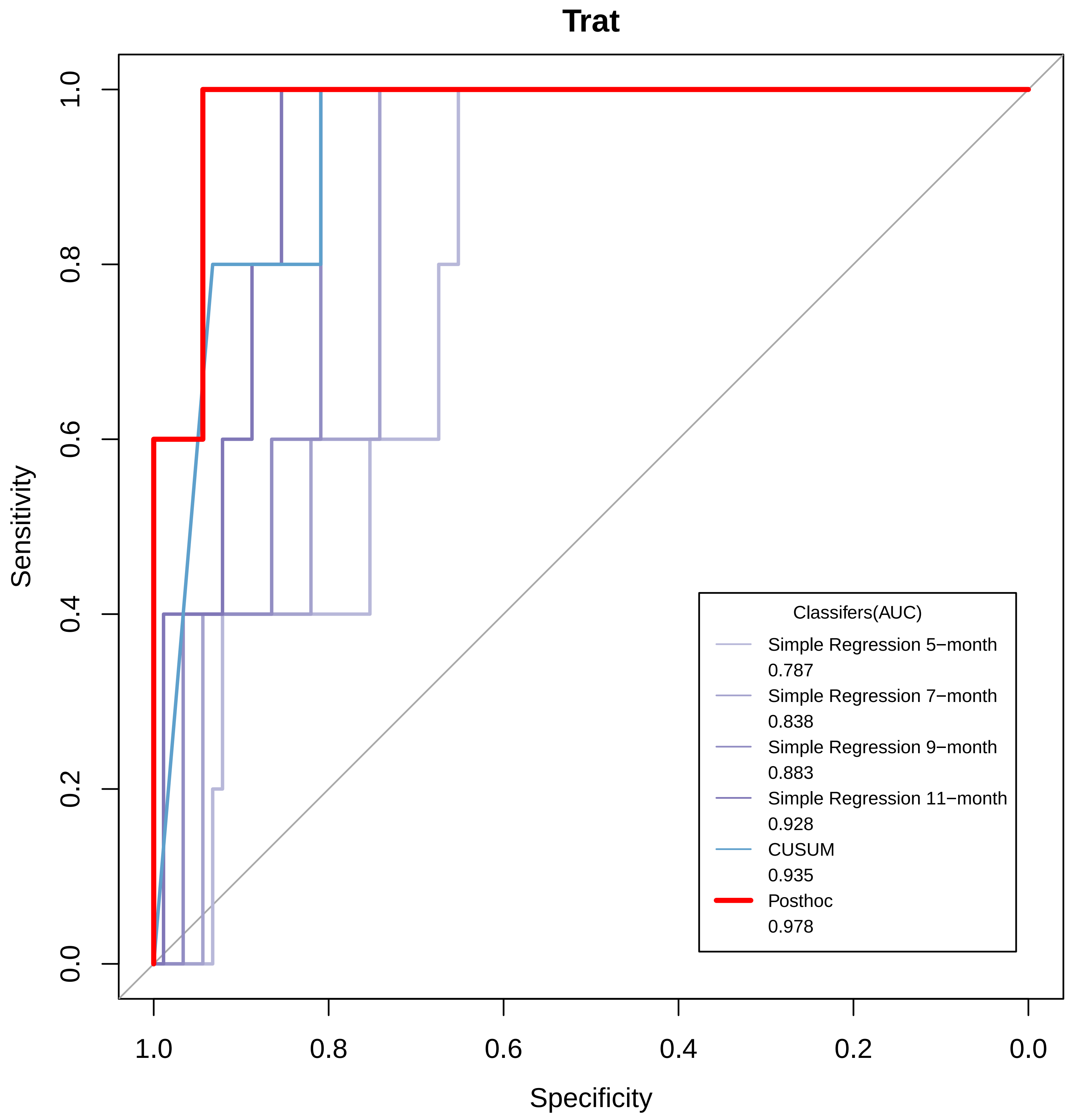
Trat
Sensitivity
Specificity
1.0
0.8
0.6
0.4
0.2
0.0
Classifers(AUC)
Simple Regression 5−month
0.787
Simple Regression 7−month
0.838
Simple Regression 9−month
0.883
Simple Regression 11−month
0.928
CUSUM
0.935
Posthoc
0.978

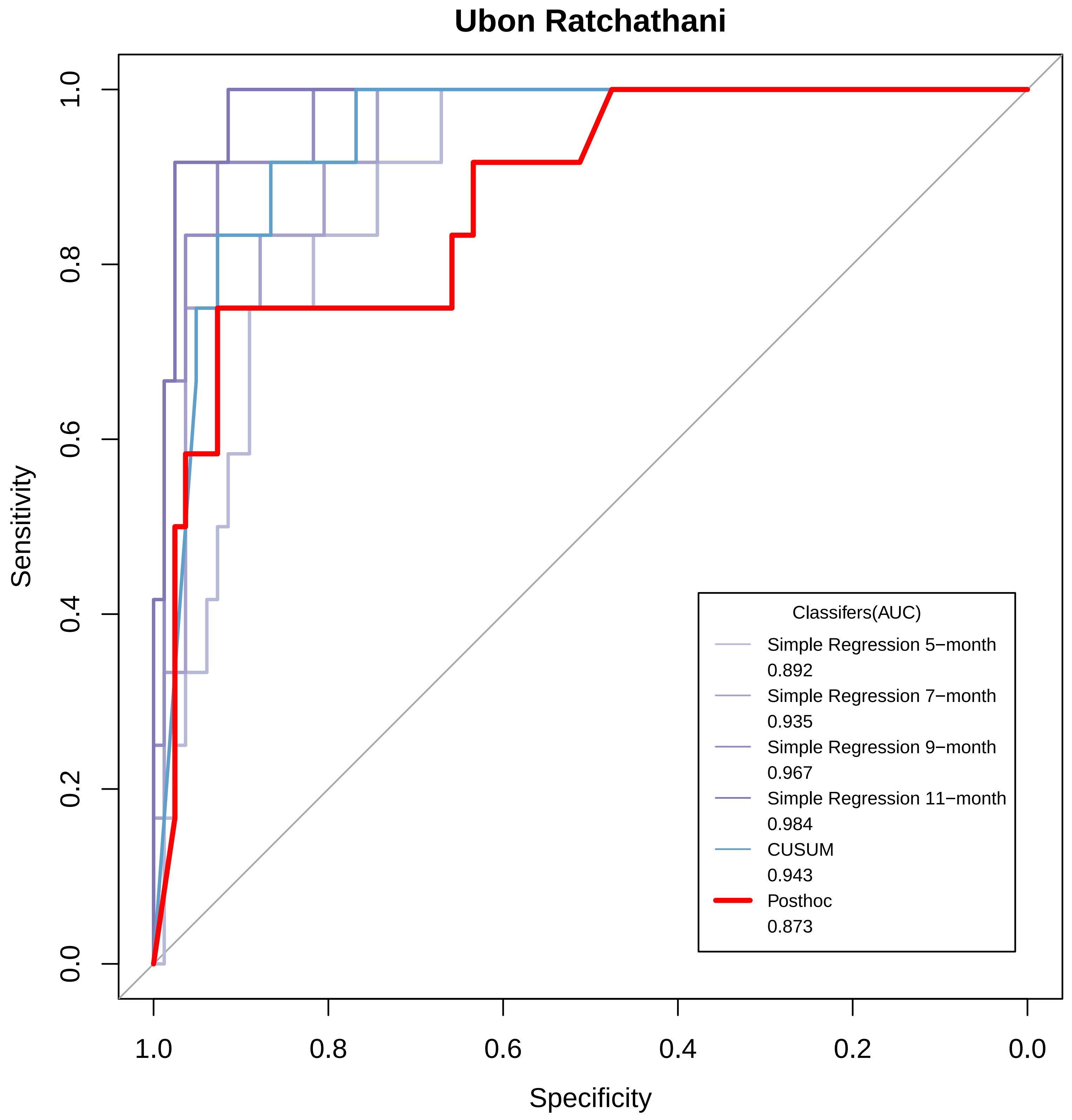
Ubon Ratchathani
Sensitivity
Specificity
1.0
0.8
0.6
0.4
0.2
0.0
Classifers(AUC)
Simple Regression 5−month
0.892
Simple Regression 7−month
0.935
Simple Regression 9−month
0.967
Simple Regression 11−month
0.984
CUSUM
0.943
Posthoc
0.873

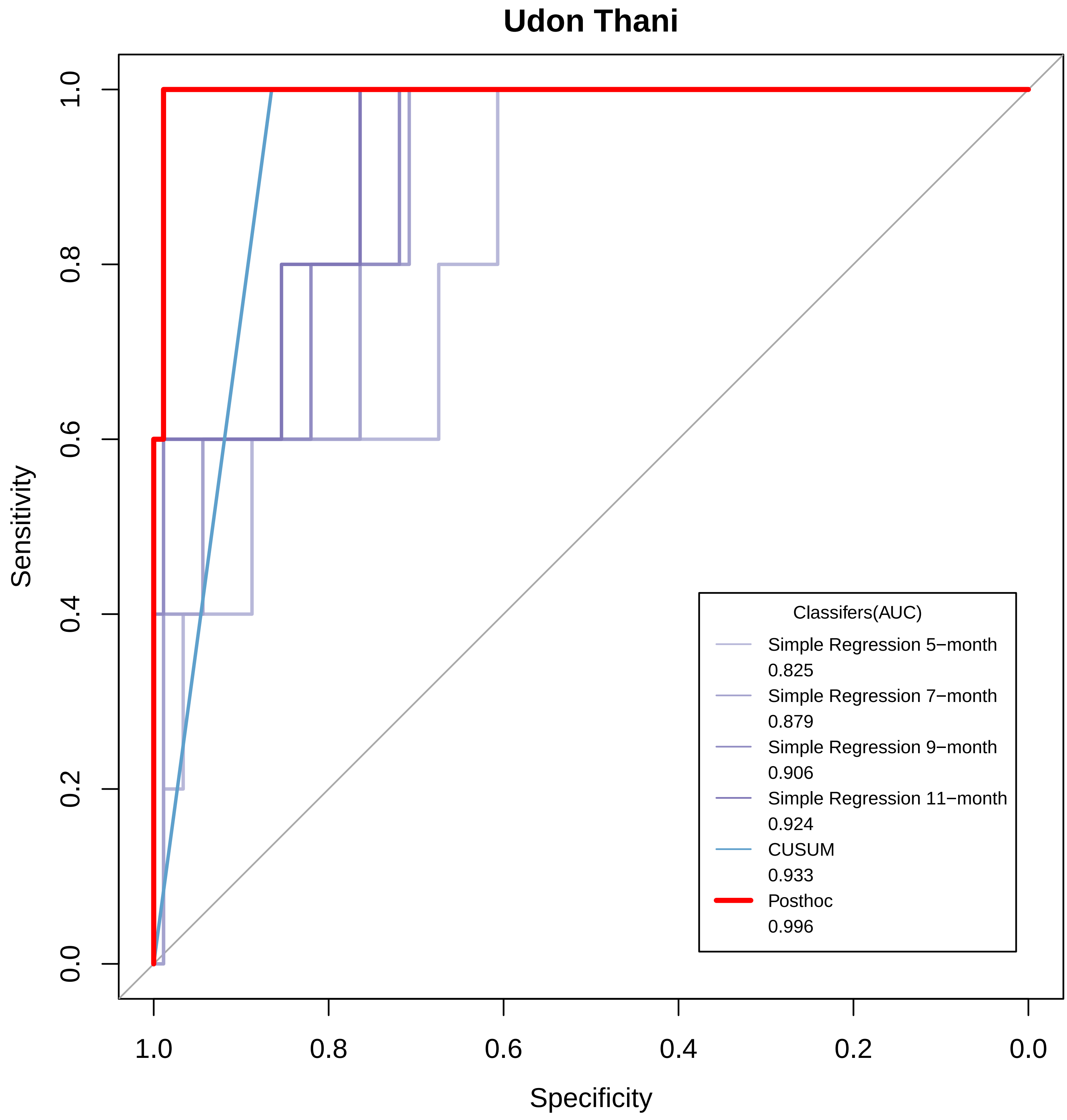
Udon Thani
Sensitivity
Specificity
1.0
0.8
0.6
0.4
0.2
0.0
Classifers(AUC)
Simple Regression 5−month
0.825
Simple Regression 7−month
0.879
Simple Regression 9−month
0.906
Simple Regression 11−month
0.924
CUSUM
0.933
Posthoc
0.996

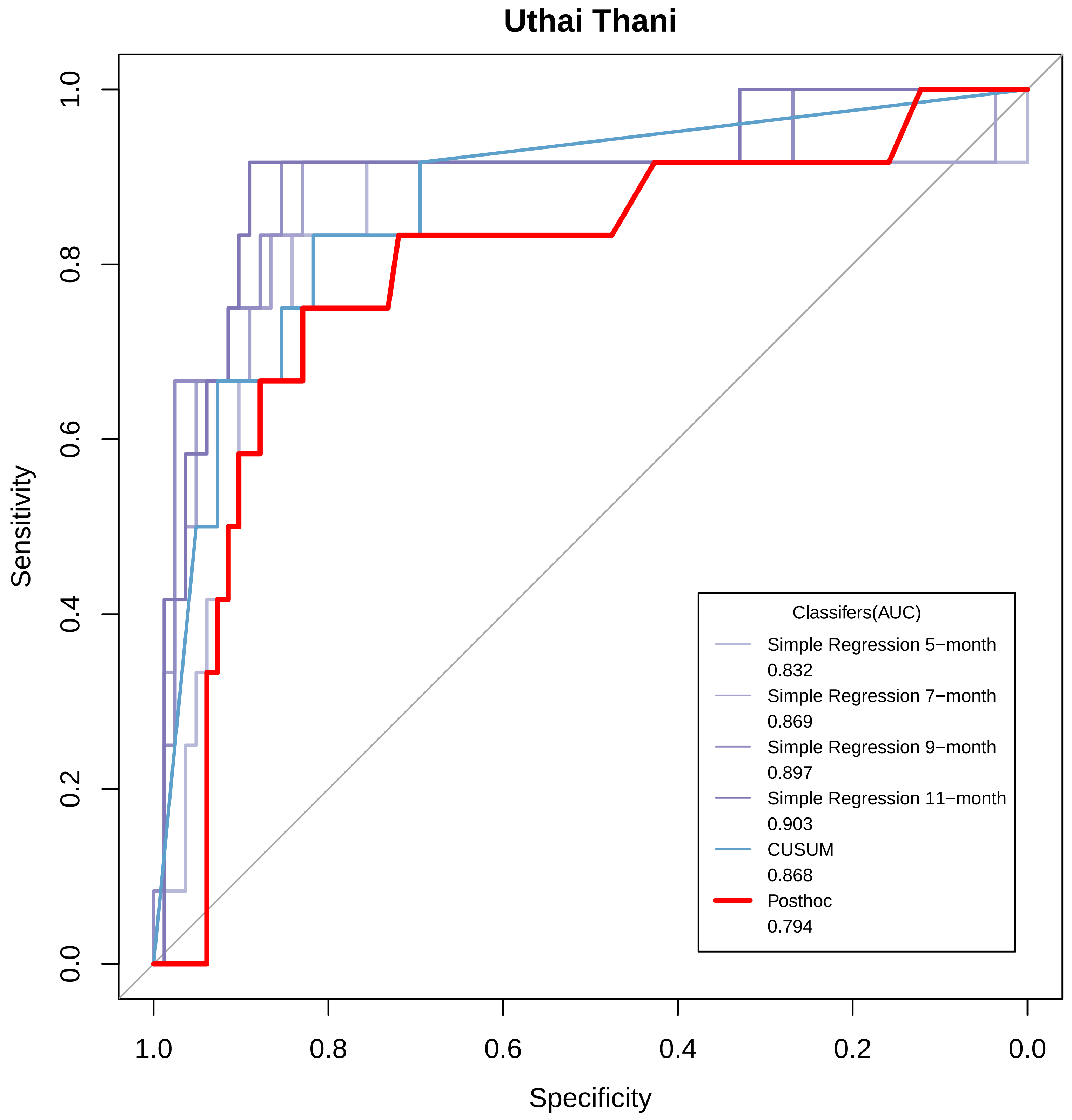
Uthai Thani
Sensitivity
Specificity
1.0
0.8
0.6
0.4
0.2
0.0
Classifers(AUC)
Simple Regression 5−month
0.832
Simple Regression 7−month
0.869
Simple Regression 9−month
0.897
Simple Regression 11−month
0.903
CUSUM
0.868
Posthoc
0.794

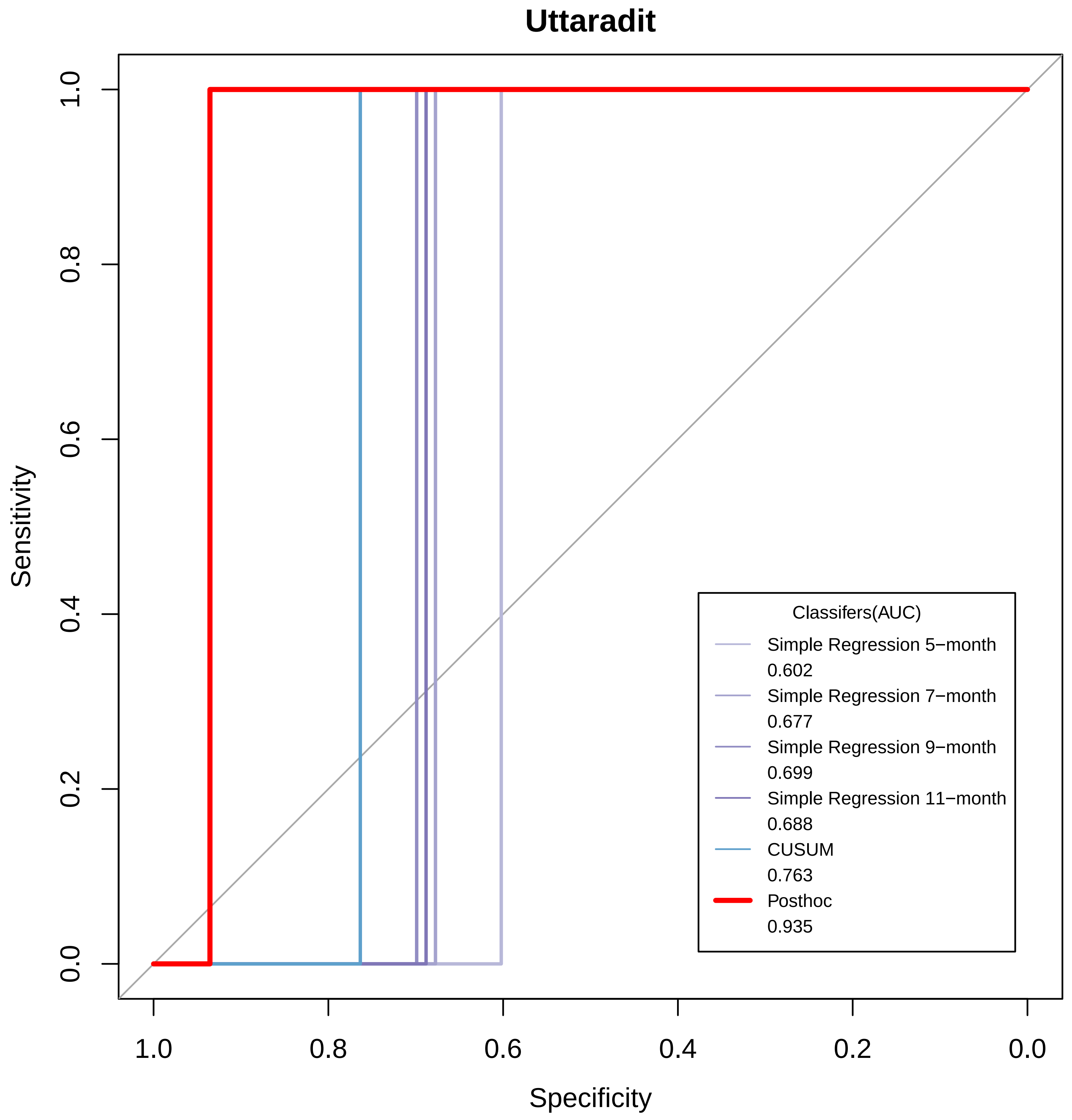

Uttaradit
Sensitivity
Specificity
1.0
0.8
0.6
0.4
0.2
0.0
Classifers(AUC)
Simple Regression 5−month
0.602
Simple Regression 7−month
0.677
Simple Regression 9−month
0.699
Simple Regression 11−month
0.688
CUSUM
0.763
Posthoc
0.935

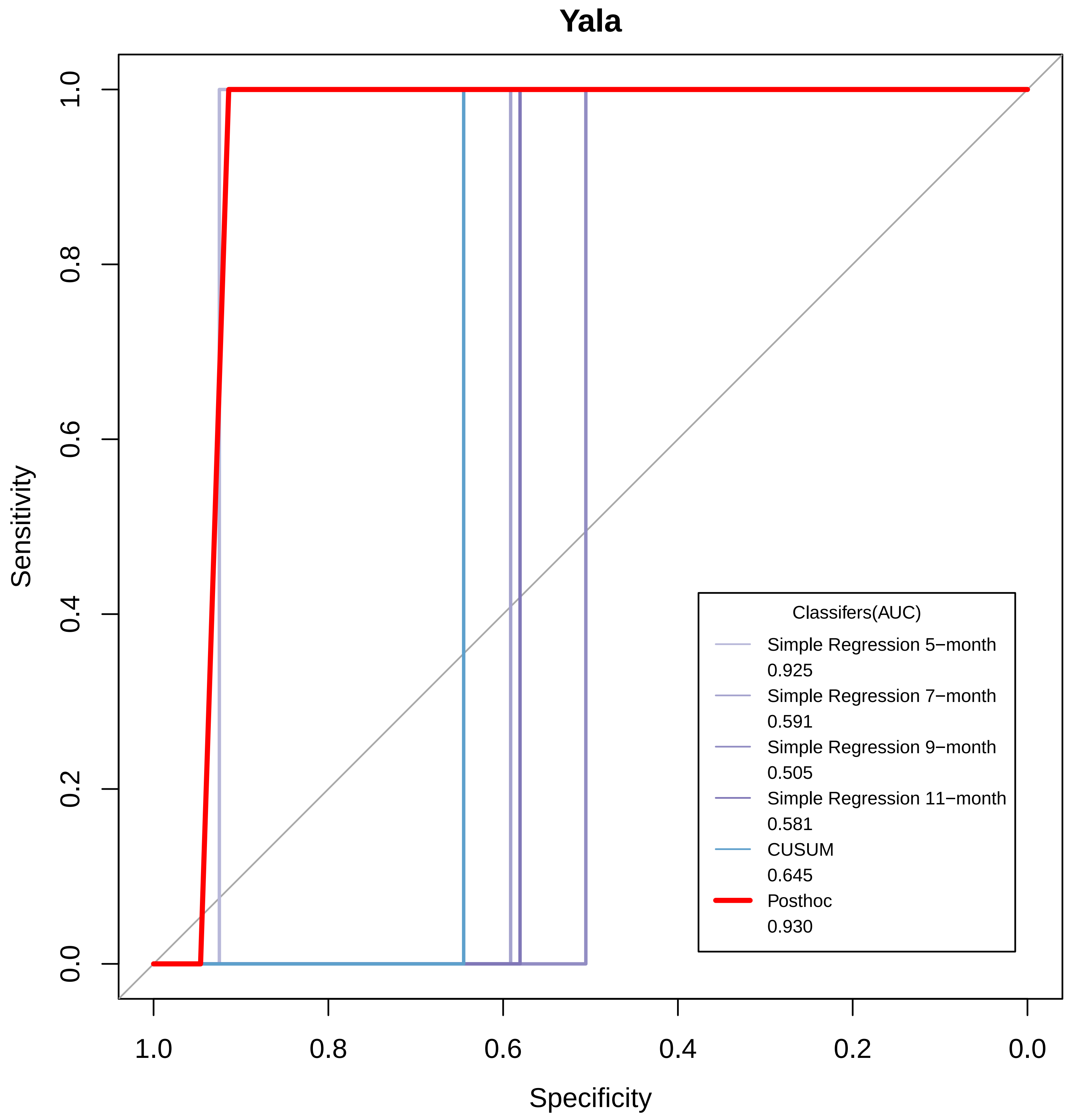
Yala
Sensitivity
1.0
0.8
0.6
0.4
0.2
0.0
Specificity
1.0
0.8
0.6
0.4
0.2
0.0
Classifers(AUC)
Simple Regression 5−month
0.925
Simple Regression 7−month
0.591
Simple Regression 9−month
0.505
Simple Regression 11−month
0.581
CUSUM
0.645
Posthoc
0.930

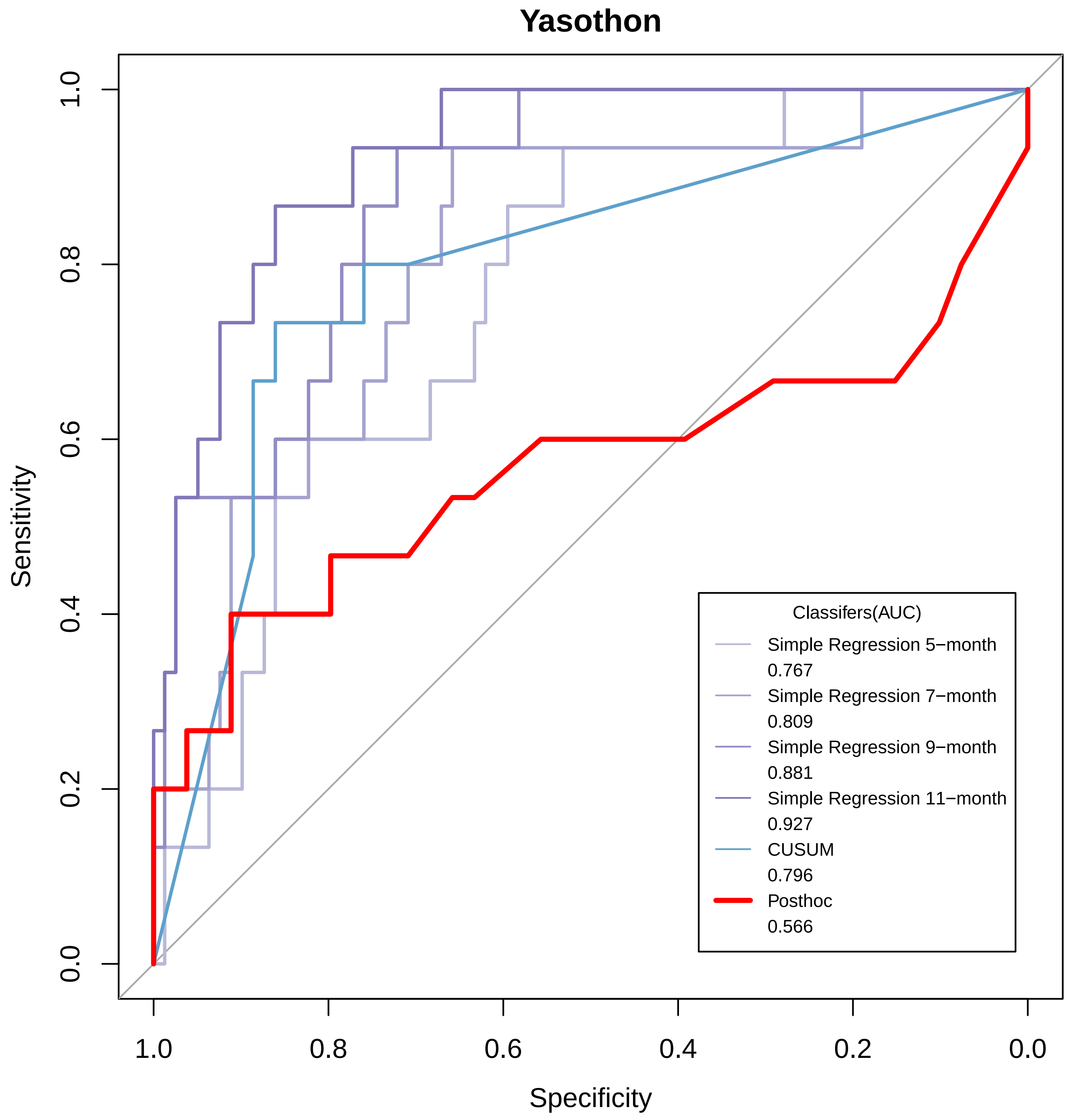
Yasothon
Sensitivity
Specificity
1.0
0.8
0.6
0.4
0.2
0.0
Classifers(AUC)
Simple Regression 5−month
0.767
Simple Regression 7−month
0.809
Simple Regression 9−month
0.881
Simple Regression 11−month
0.927
CUSUM
0.796
Posthoc
0.566

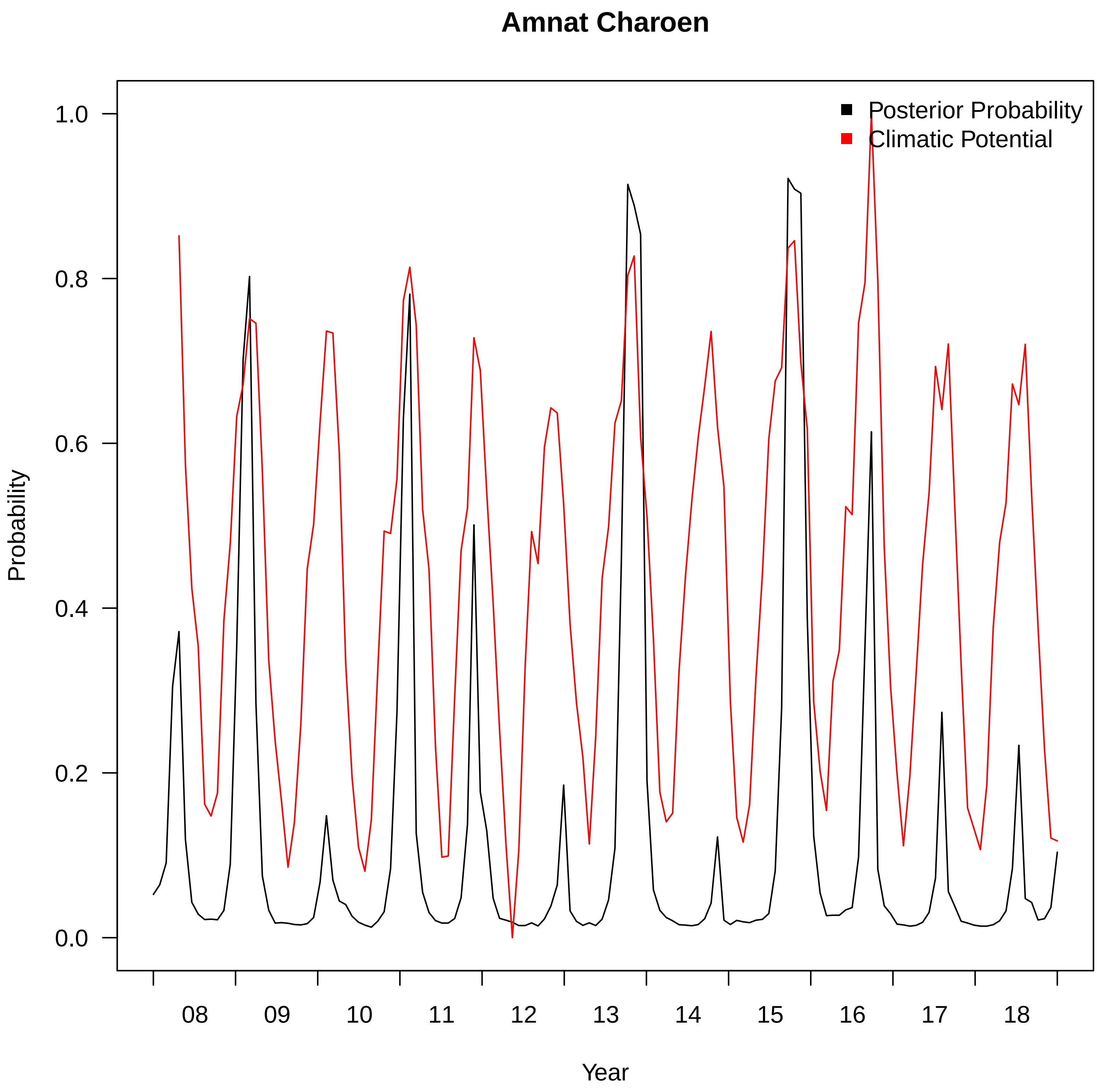

Amnat Charoen
Posterior Probability
Climatic Potential
Probability
1.0
0.8
0.6
0.4
0.2
0.0
08
09
10
11
12
13
14
15
16
17
18
Year

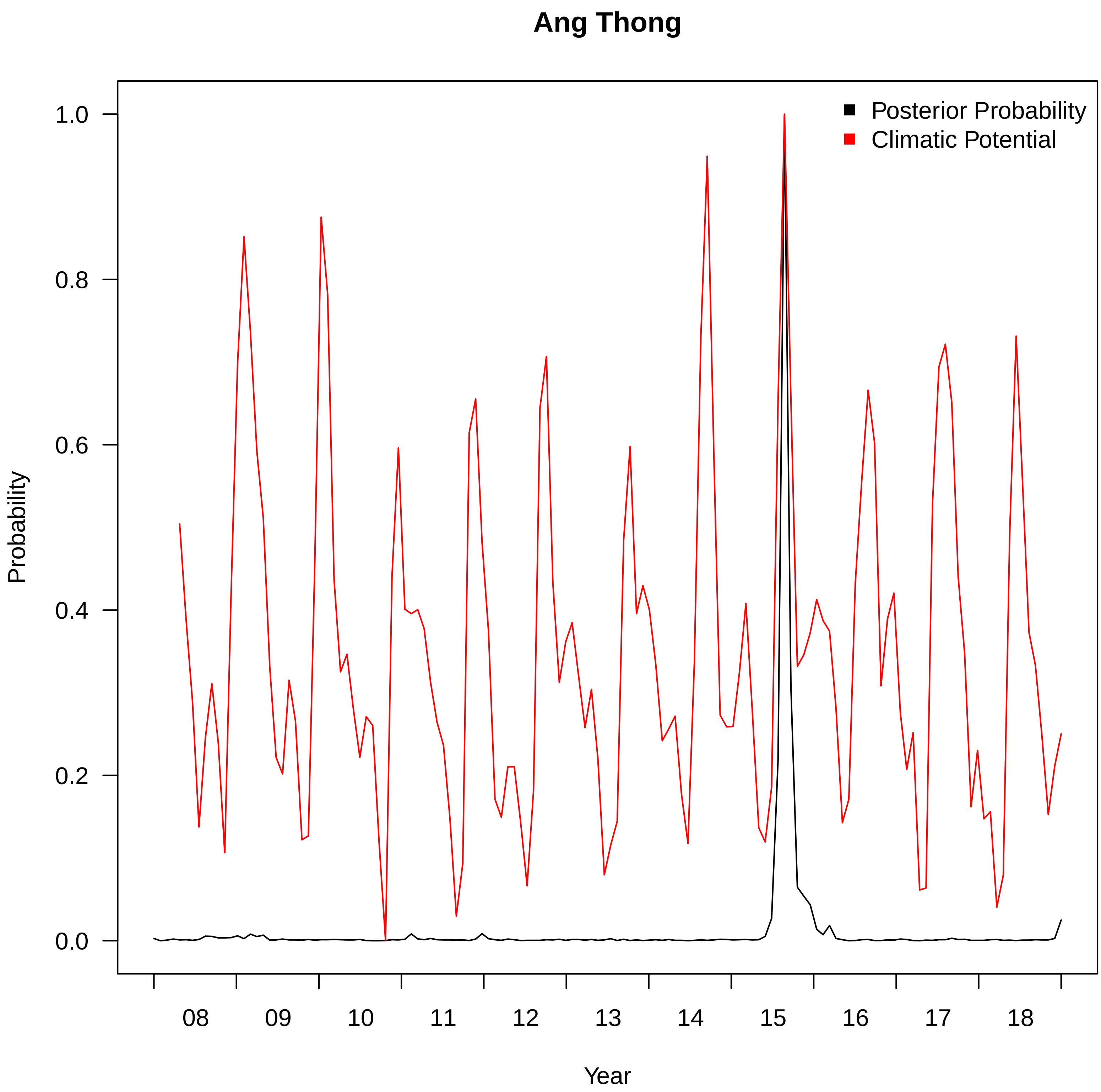

Ang Thong
Posterior Probability
Climatic Potential
Probability
1.0
0.8
0.6
0.4
0.2
0.0
08
09
10
11
12
13
14
15
16
17
18
Year

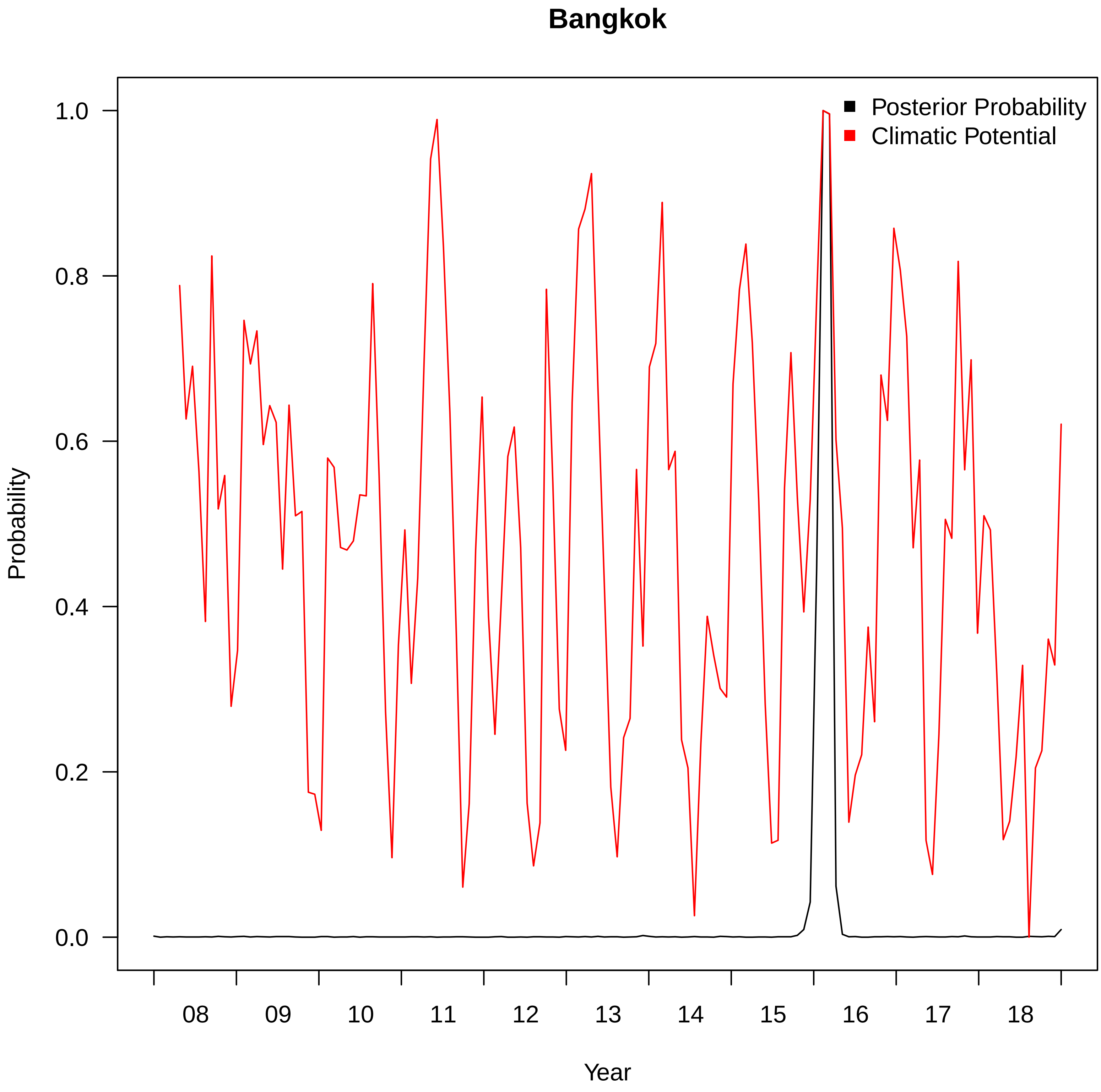

Bangkok
Posterior Probability
Climatic Potential
Probability
1.0
0.8
0.6
0.4
0.2
0.0
Year
08
09
10
11
12
13
14
15
16
17
18

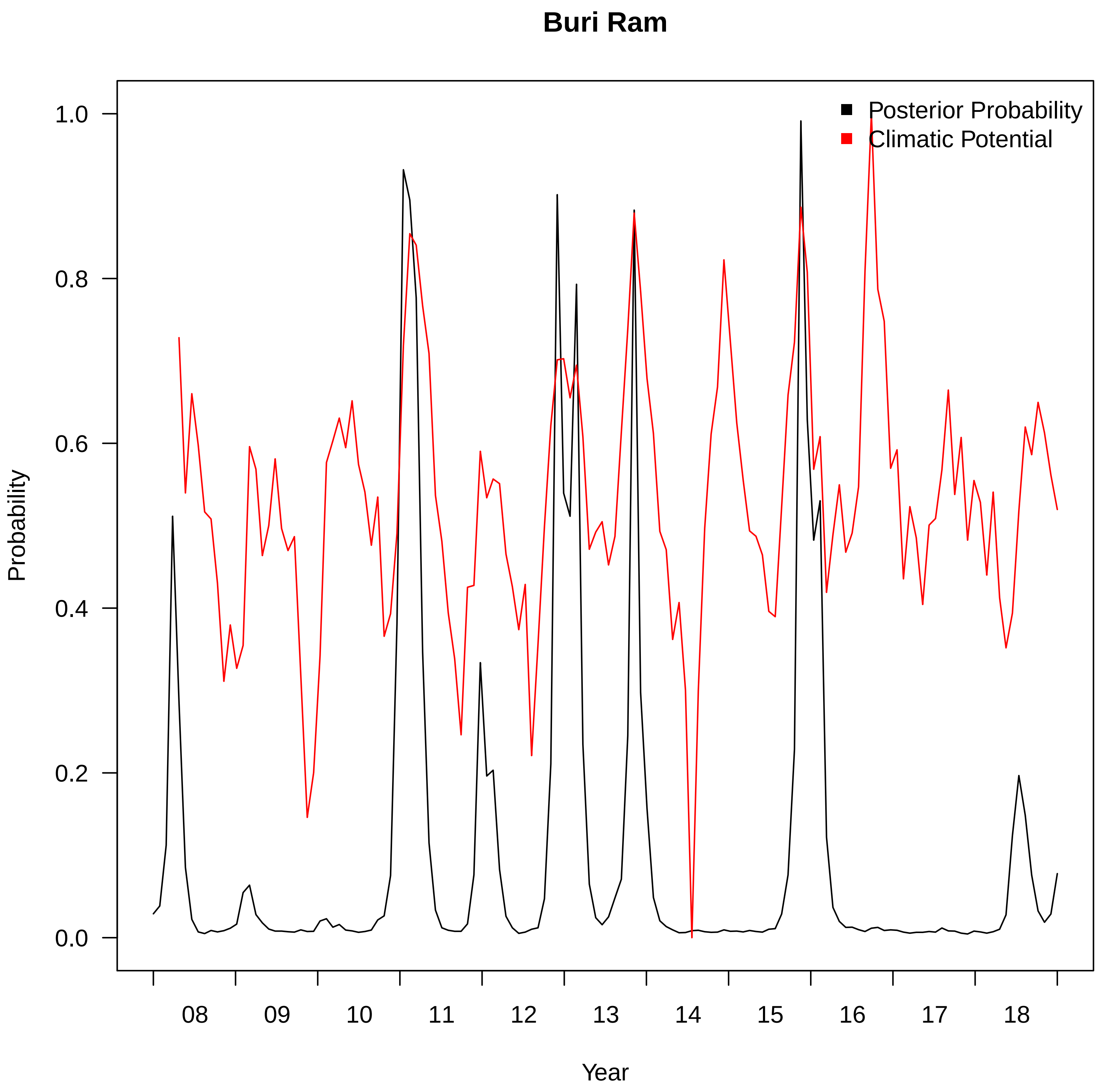
Buri Ram
Posterior Probability
Climatic Potential
Probability
1.0
0.8
0.6
0.4
0.2
0.0
08
09
10
11
12
13
14
15
16
17
18
Year

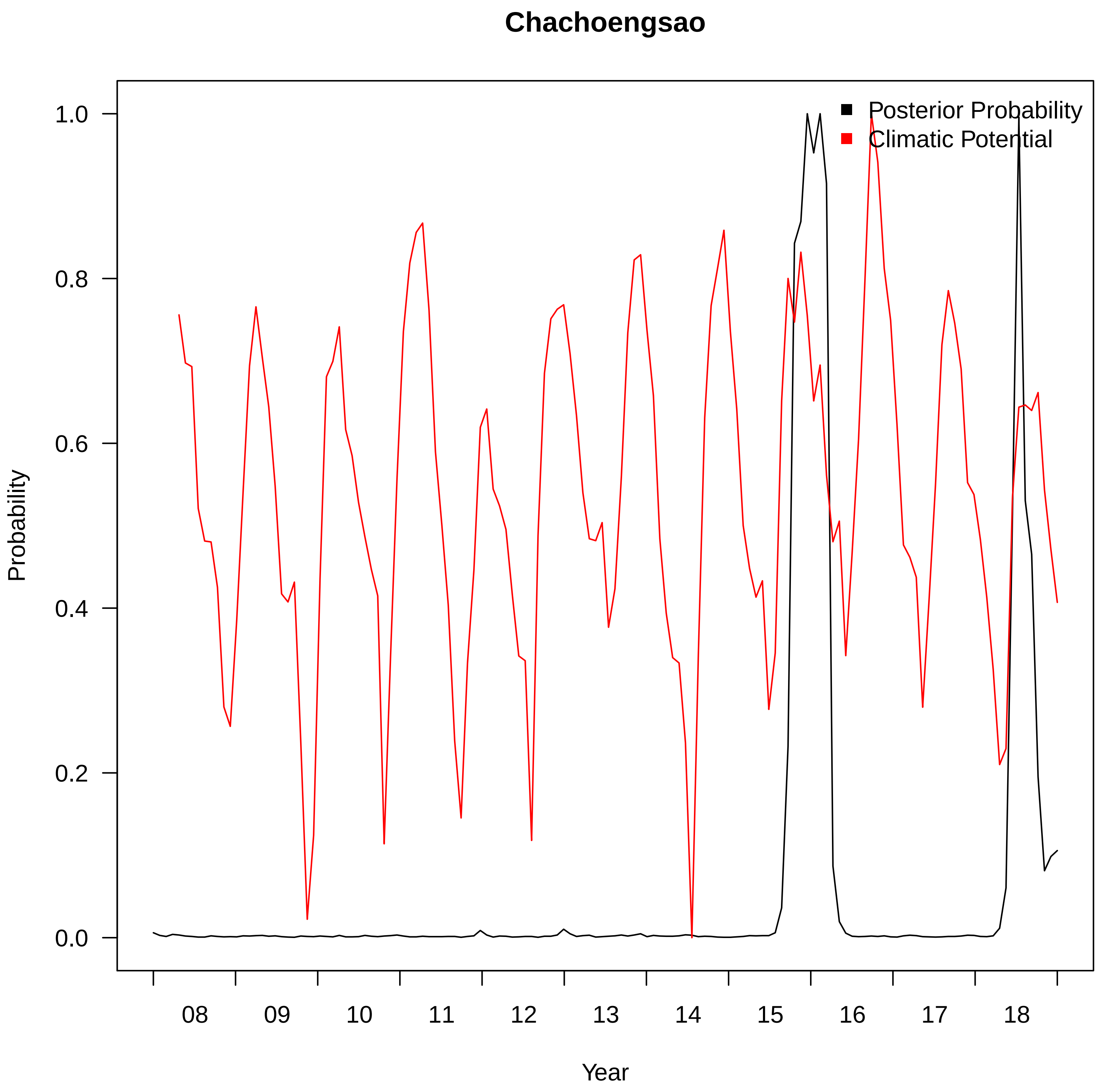

Chachoengsao
Posterior Probability
Climatic Potential
Probability
1.0
0.8
0.6
0.4
0.2
0.0
08
09
10
11
12
13
14
15
16
17
18
Year

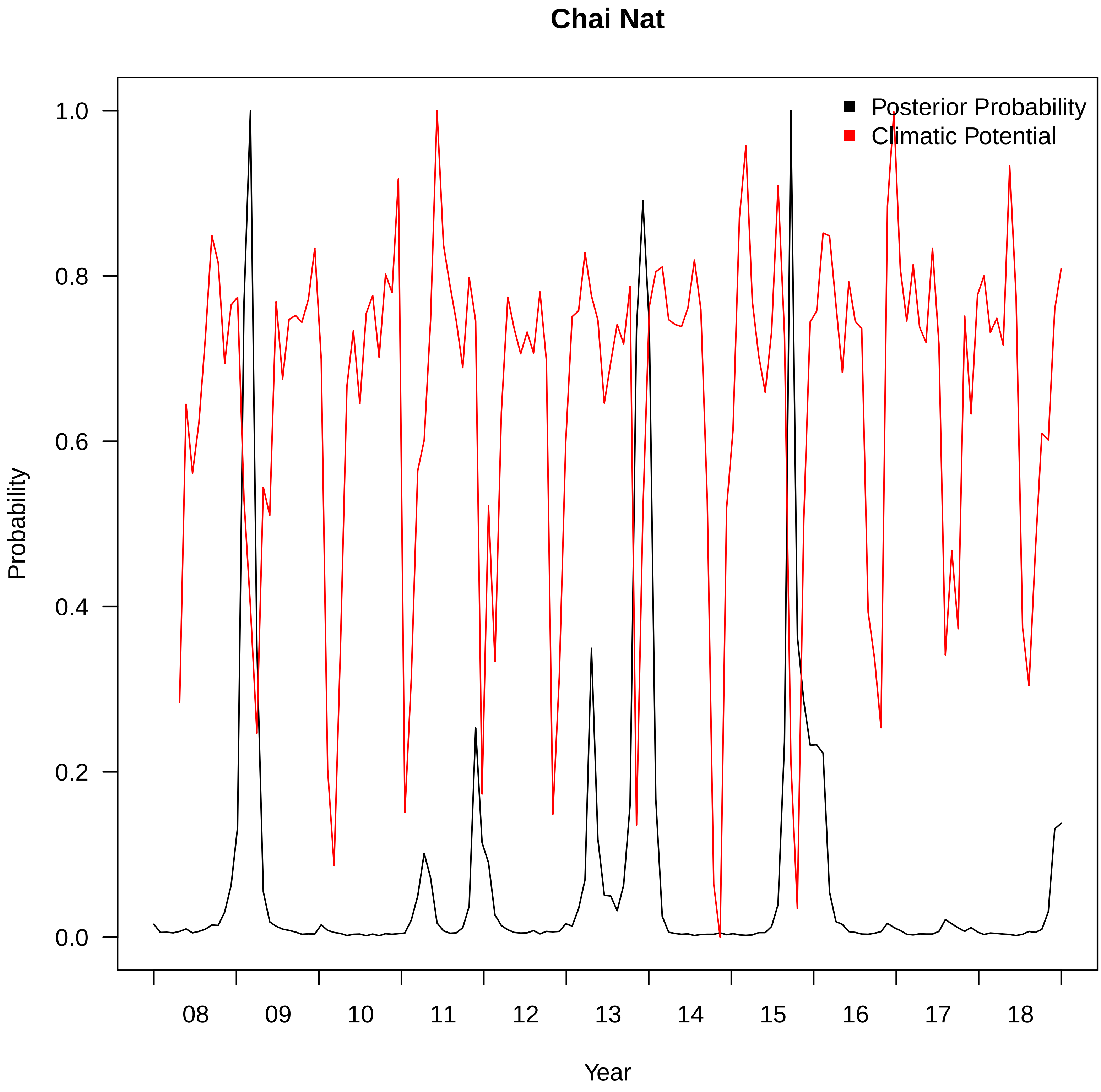

Chai Nat
Posterior Probability
Climatic Potential
Probability
1.0
0.8
0.6
0.4
0.2
0.0
08
09
10
11
12
13
14
15
16
17
18
Year

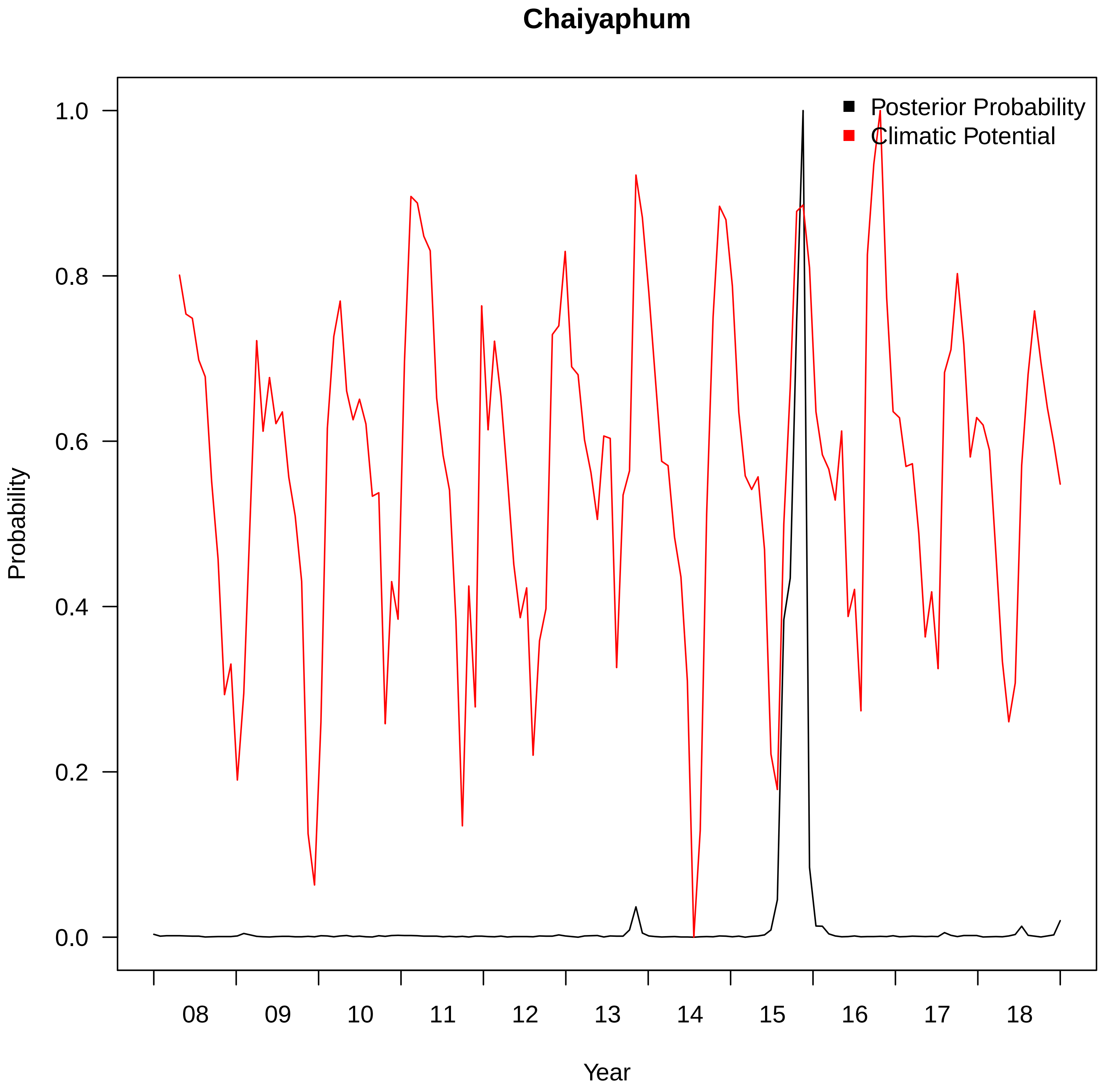
Chaiyaphum
Posterior Probability
Climatic Potential
Probability
1.0
0.8
0.6
0.4
0.2
0.0
08
09
10
11
12
13
14
15
16
17
18
Year

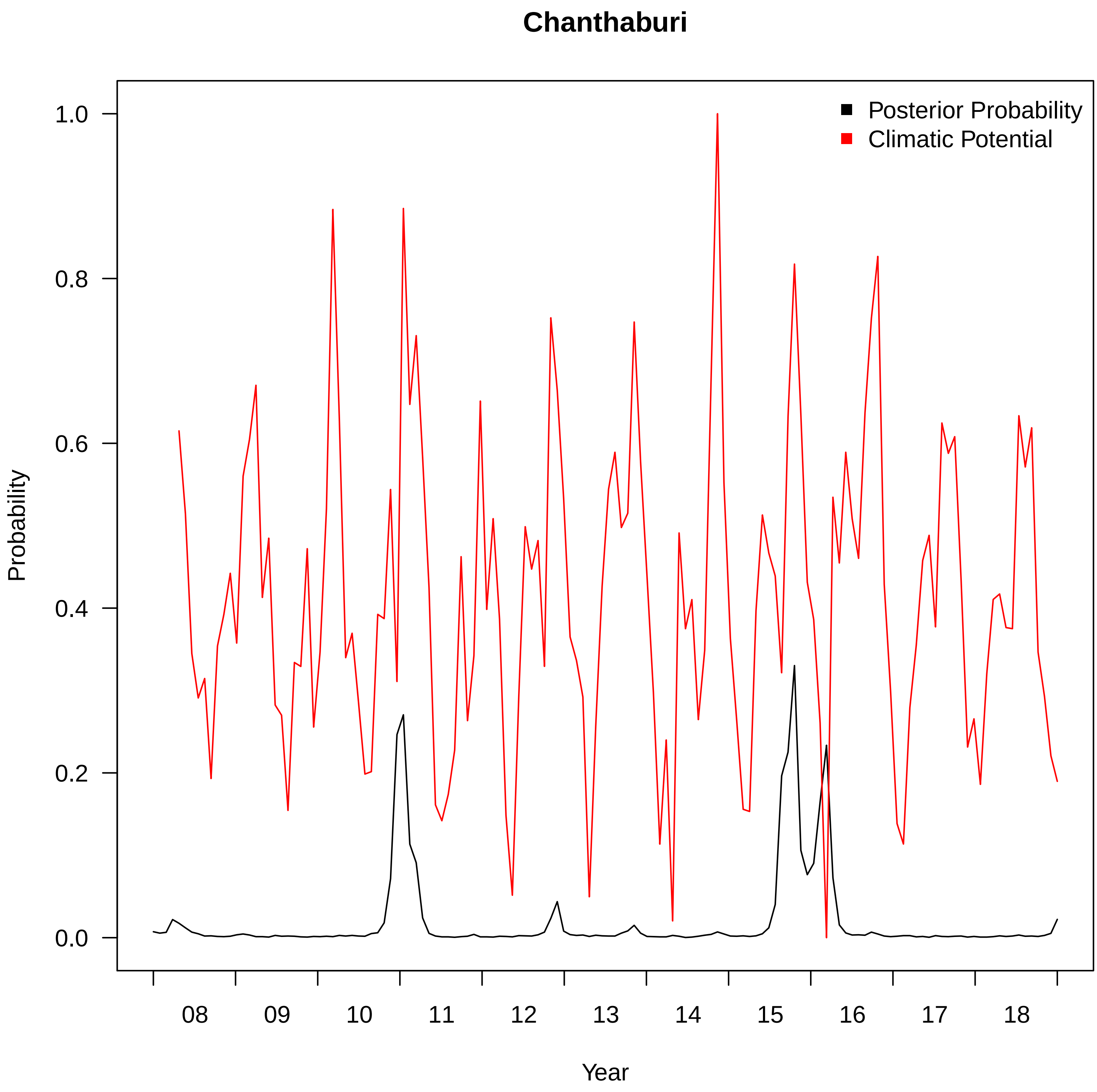

Chanthaburi
Posterior Probability
Climatic Potential
Probability
1.0
0.8
0.6
0.4
0.2
0.0
08
09
10
11
12
13
14
15
16
17
18
Year

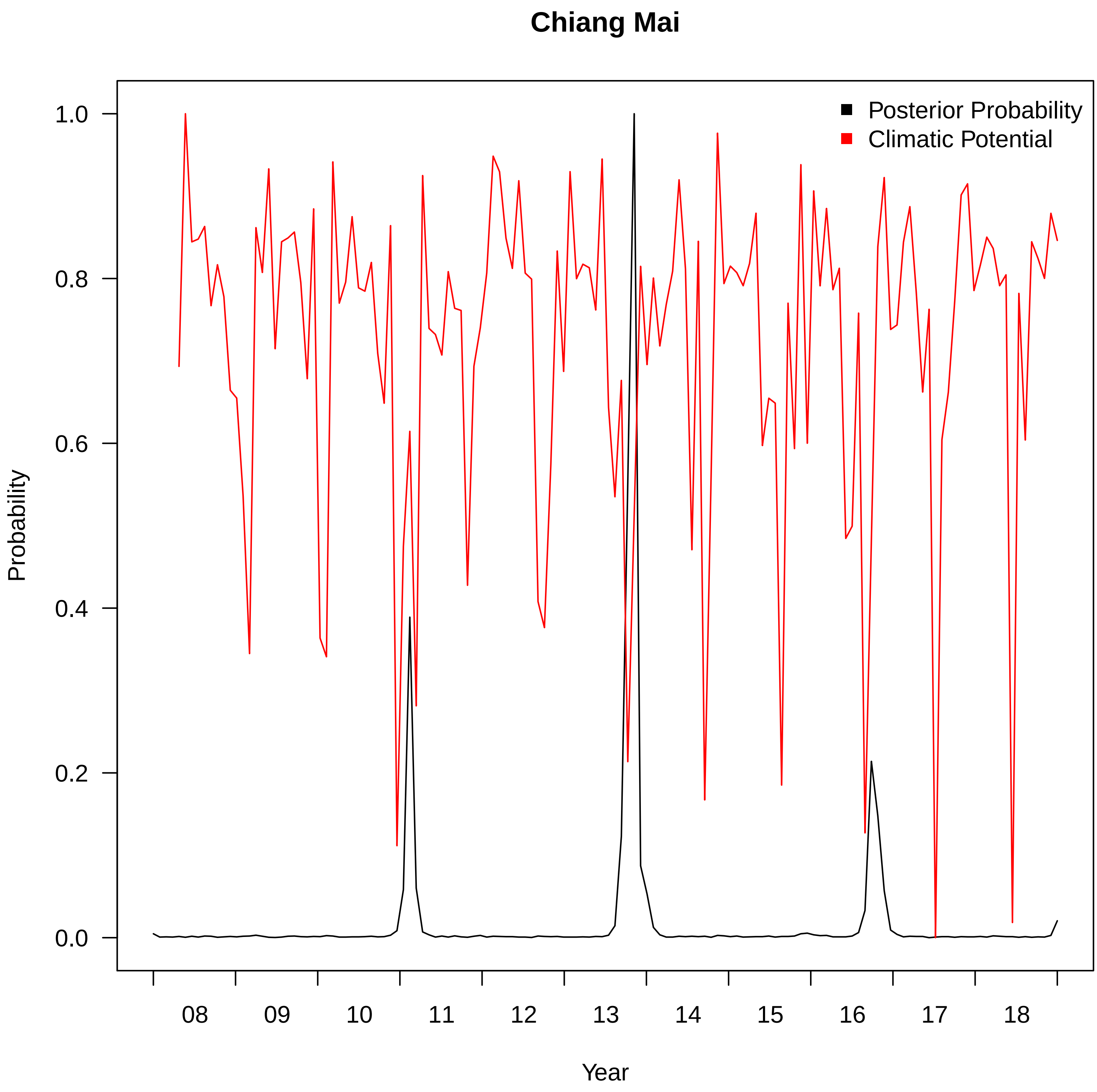
Chiang Mai
Posterior Probability
Climatic Potential
Probability
1.0
0.8
0.6
0.4
0.2
0.0
08
09
10
11
12
13
14
15
16
17
18
Year

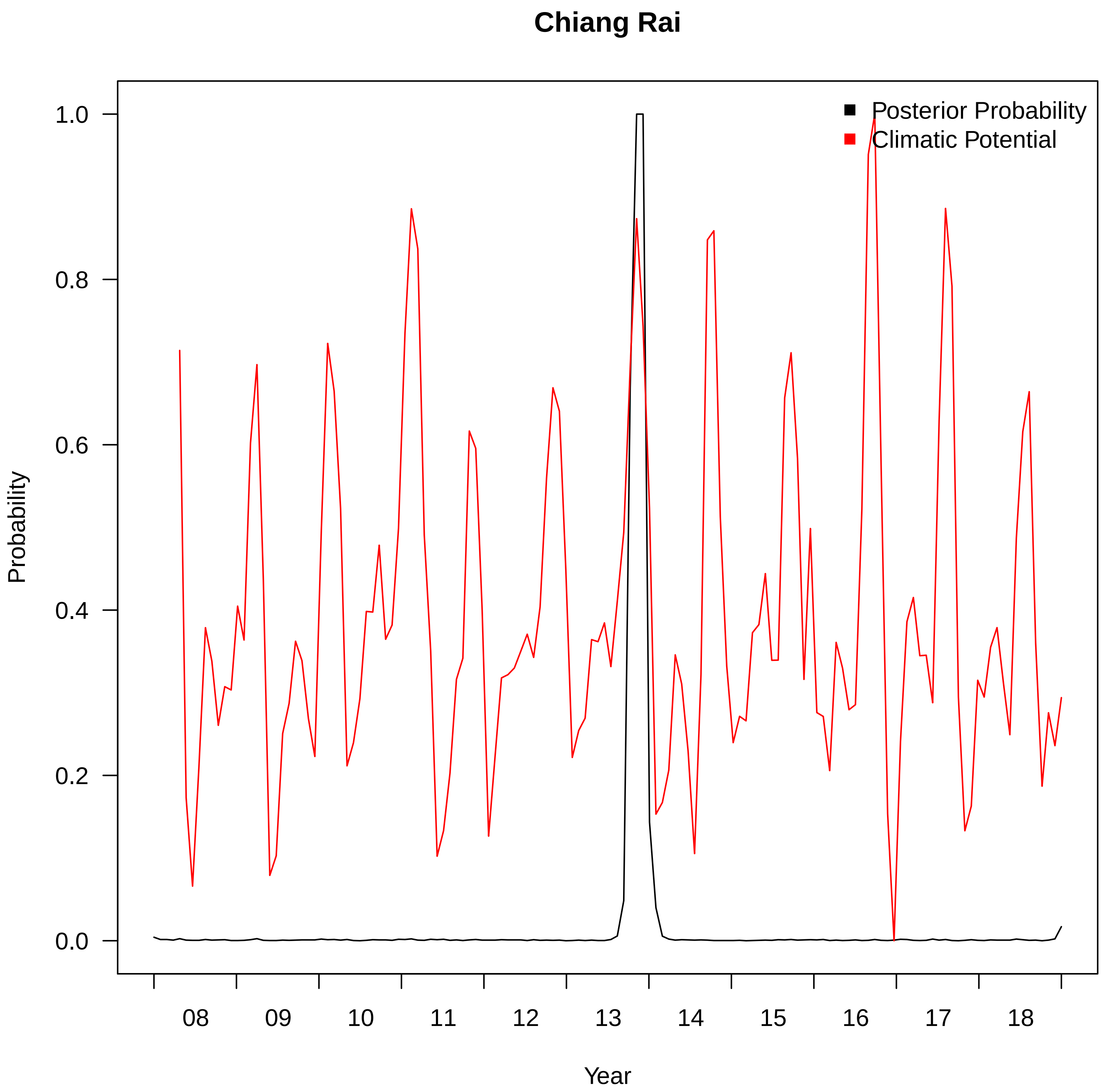

Chiang Rai
Posterior Probability
Climatic Potential
Probability
1.0
0.8
0.6
0.4
0.2
0.0
08
09
10
11
12
13
14
15
16
17
18
Year

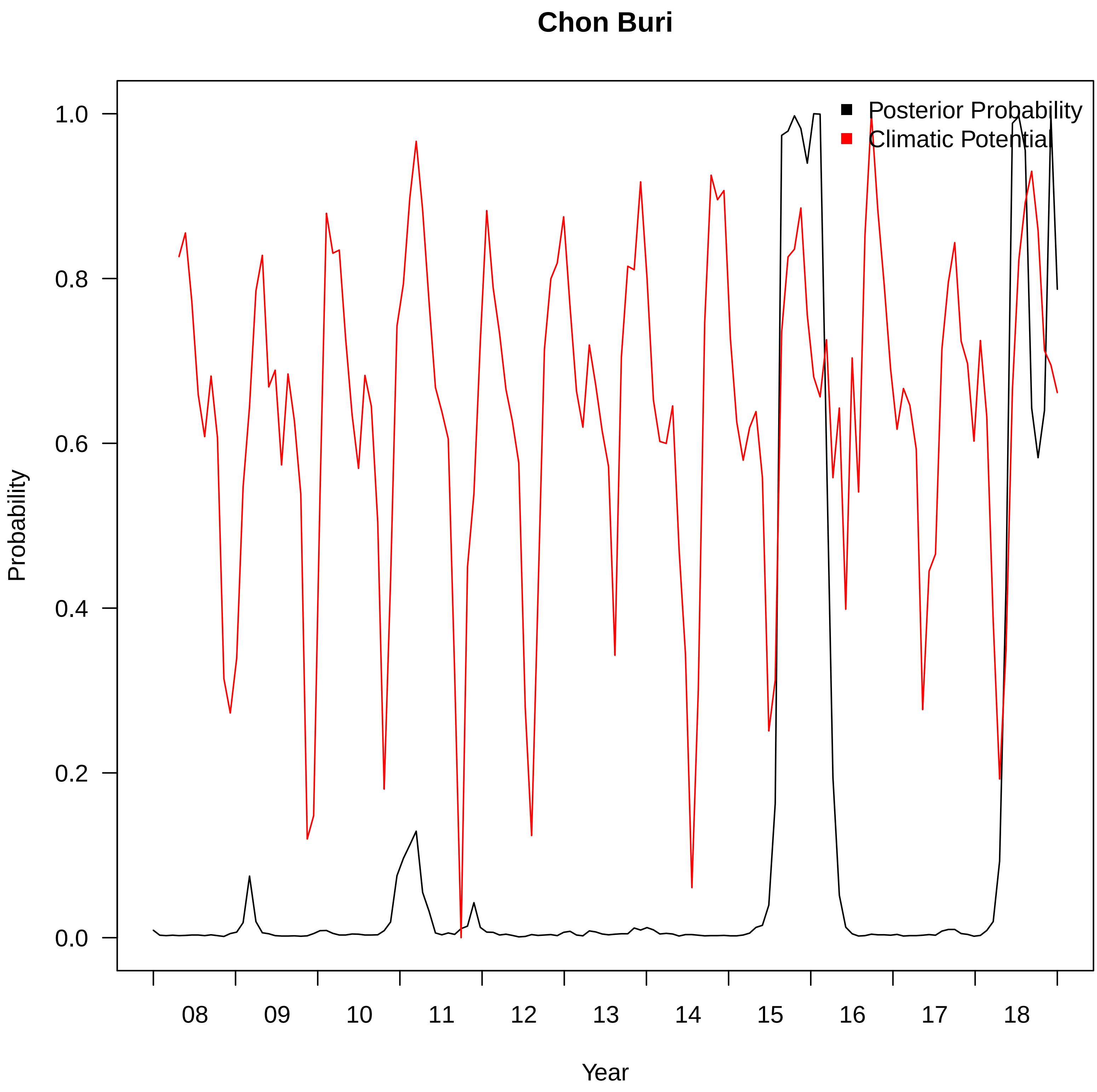

Chon Buri
Posterior Probability
Climatic Potential
Probability
1.0
0.8
0.6
0.4
0.2
0.0
08
09
10
11
12
13
14
15
16
17
18
Year

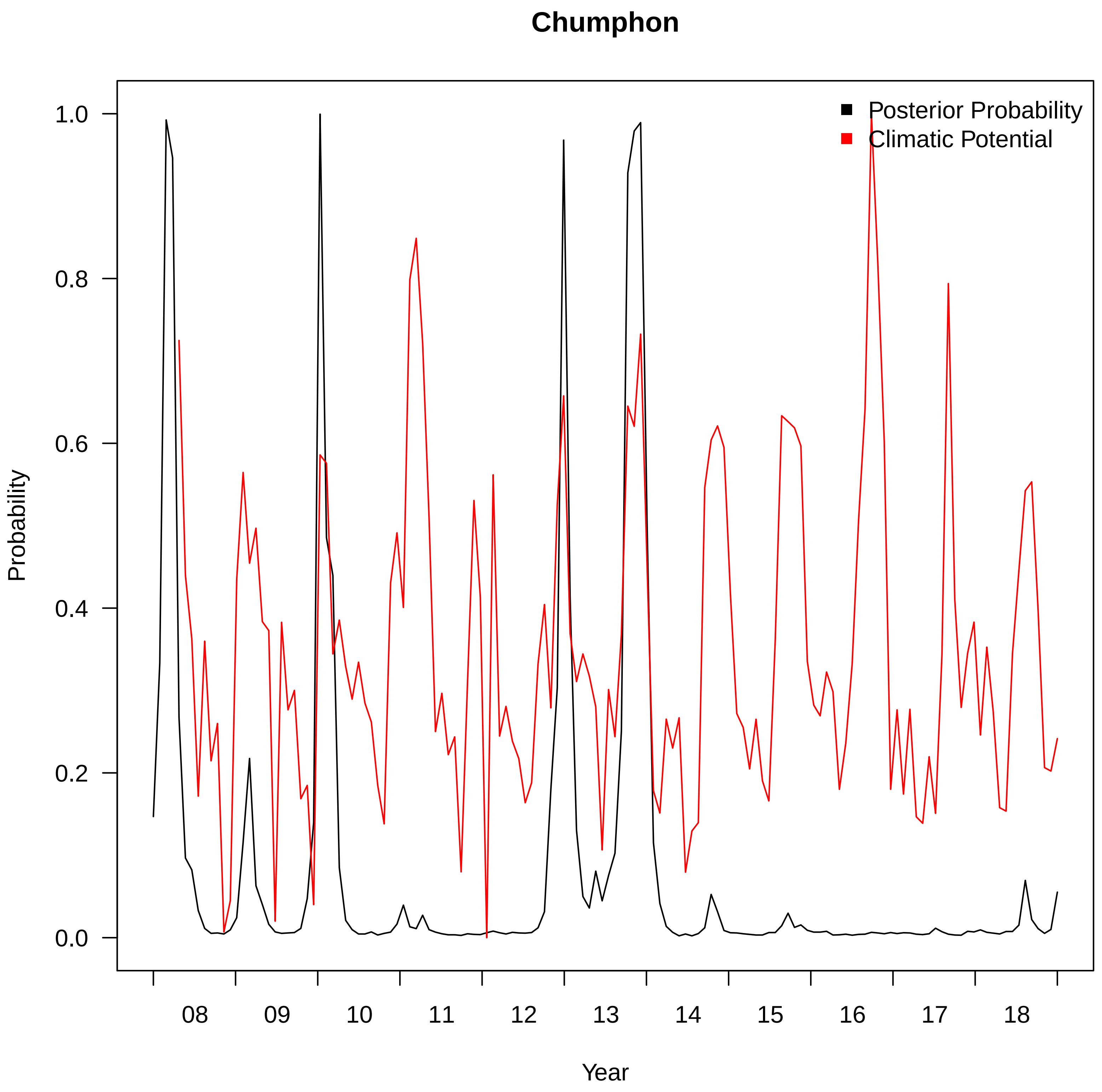
Chumphon
Posterior Probability
Climatic Potential
Probability
1.0
0.8
0.6
0.4
0.2
0.0
08
09
10
11
12
13
14
15
16
17
18
Year

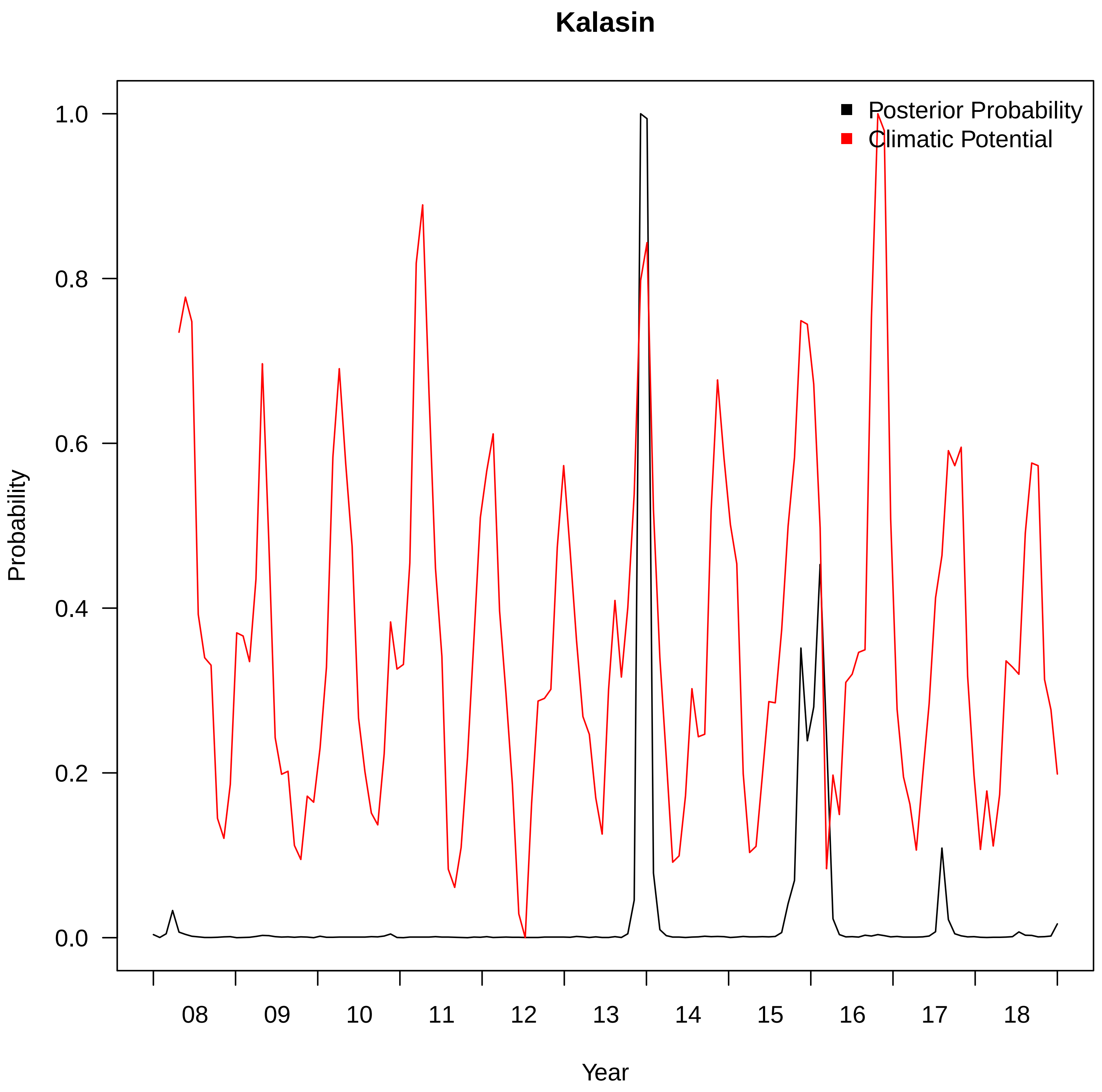
Kalasin
Posterior Probability
Climatic Potential
Probability
1.0
0.8
0.6
0.4
0.2
0.0
08
09
10
11
12
13
14
15
16
17
18
Year

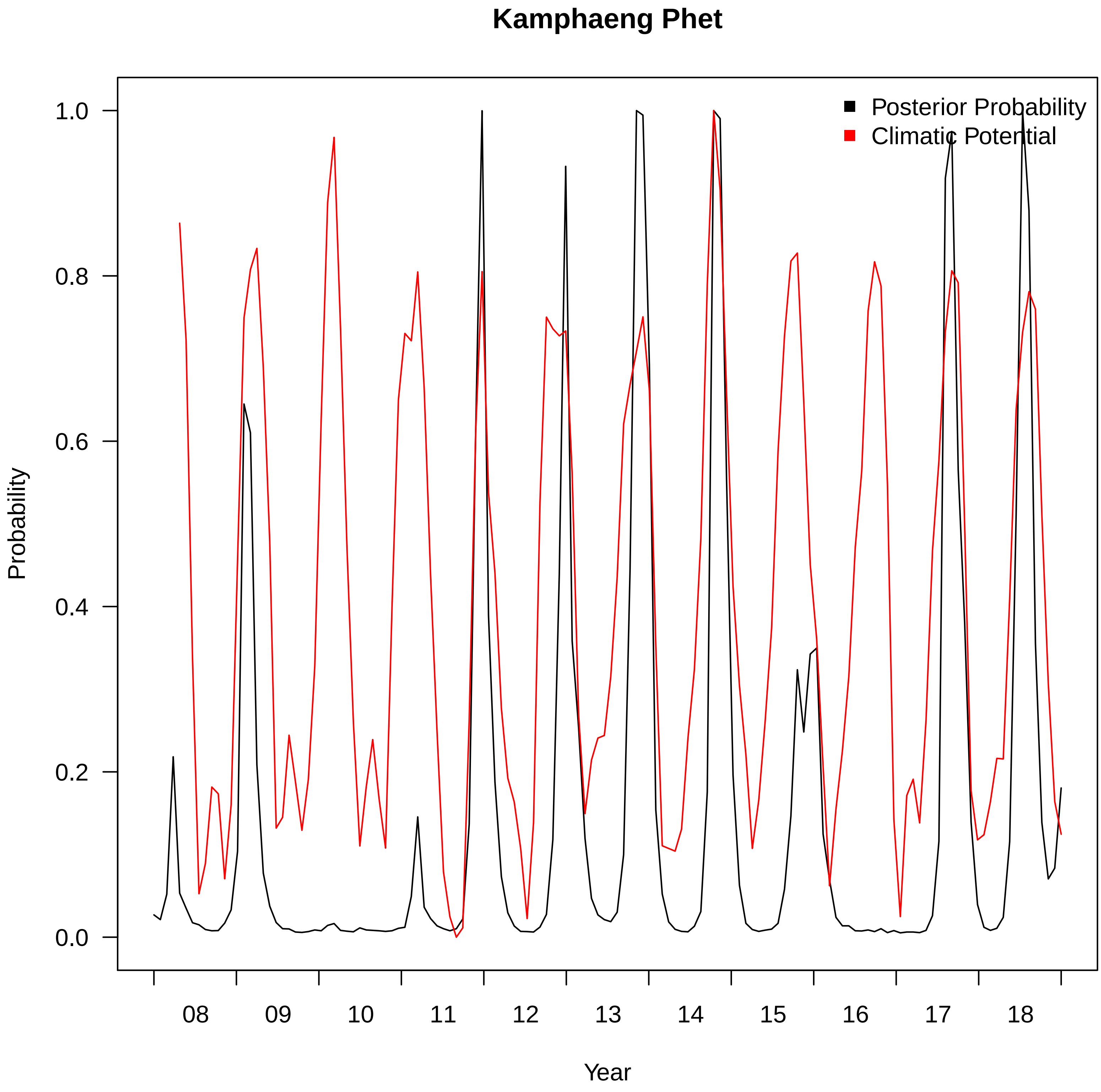
Kamphaeng Phet
Posterior Probability
Climatic Potential
Probability
Year
0.0
0.2
0.4
0.6
0.8
1.0
08
09
10
11
12
13
14
15
16
17
18

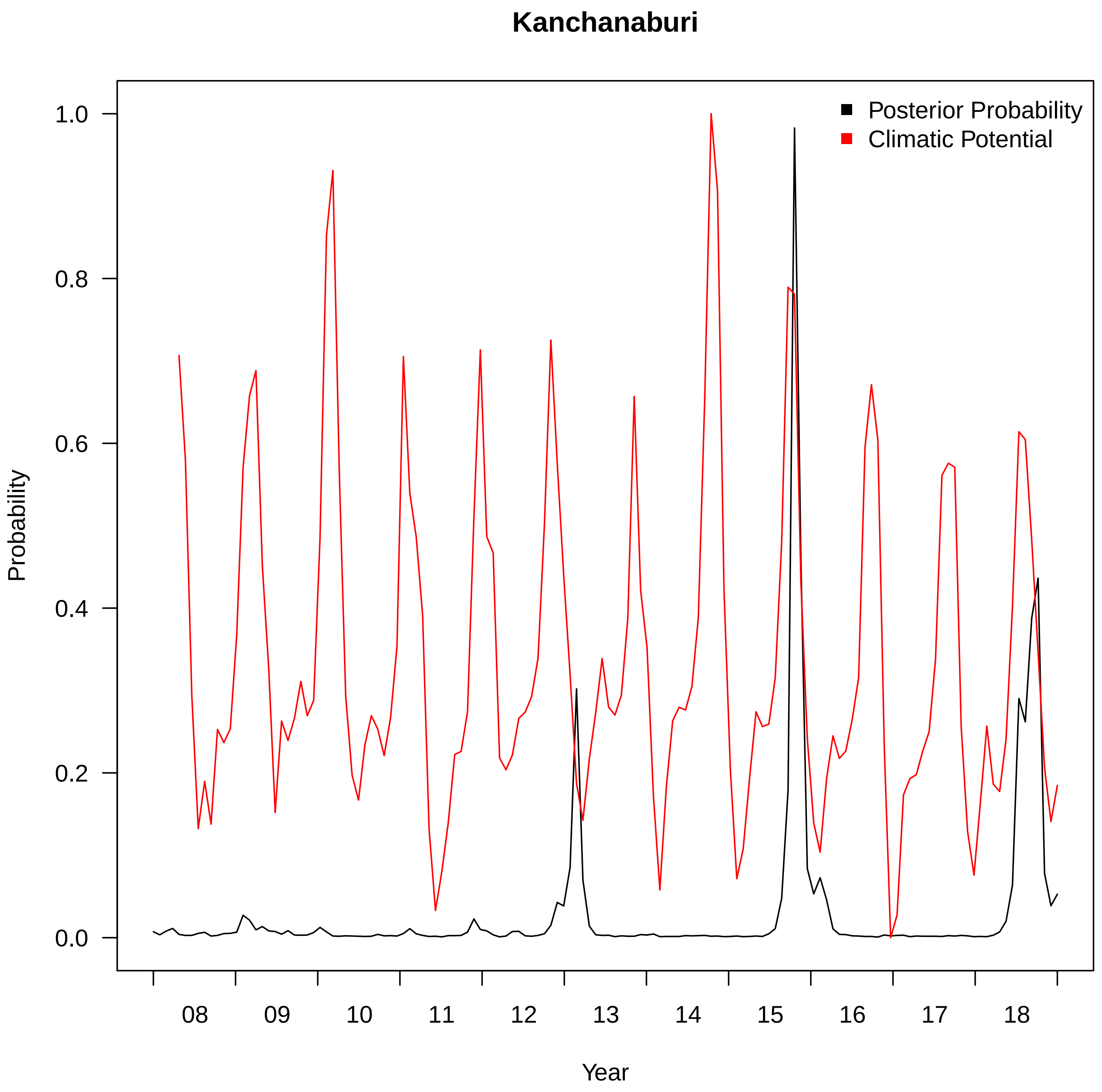

Kanchanaburi
Posterior Probability
Climatic Potential
Probability
1.0
0.8
0.6
0.4
0.2
0.0
08
09
10
11
12
13
14
15
16
17
18
Year

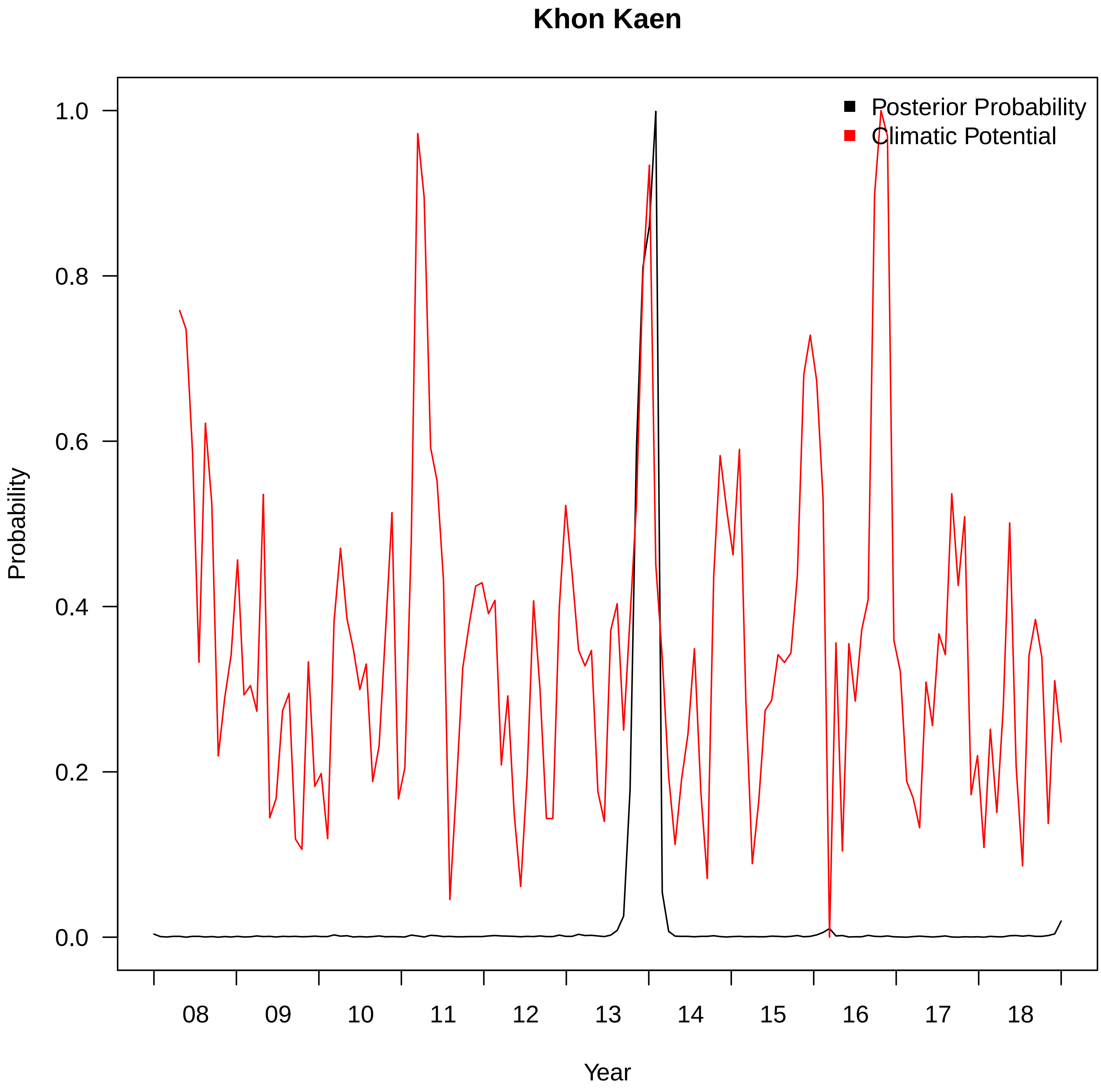

Khon Kaen
Posterior Probability
Climatic Potential
Probability
1.0
0.8
0.6
0.4
0.2
0.0
08
09
10
11
12
13
14
15
16
17
18
Year

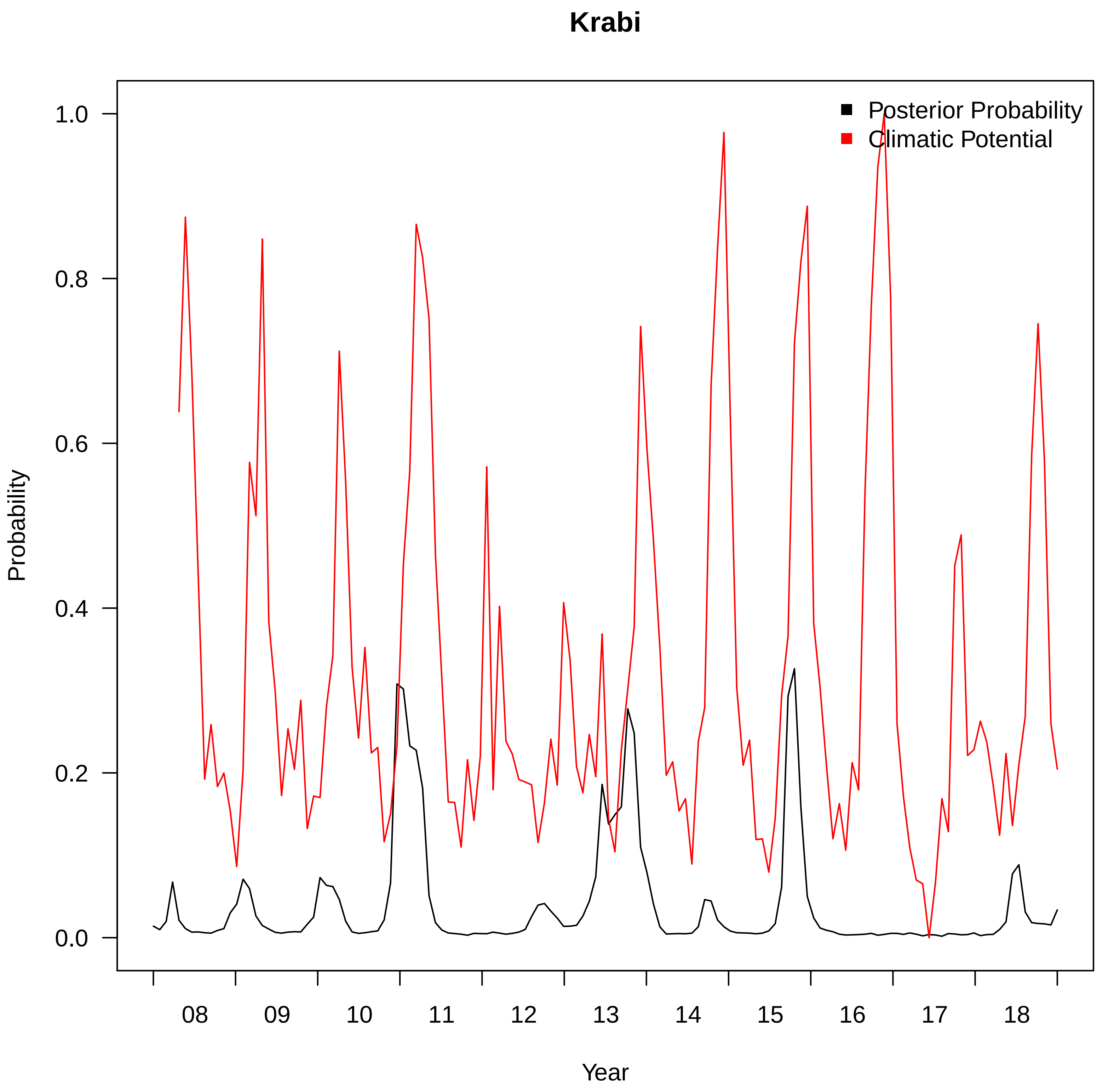

Krabi
Posterior Probability
Climatic Potential
Probability
1.0
0.8
0.6
0.4
0.2
0.0
08
09
10
11
12
13
14
15
16
17
18
Year

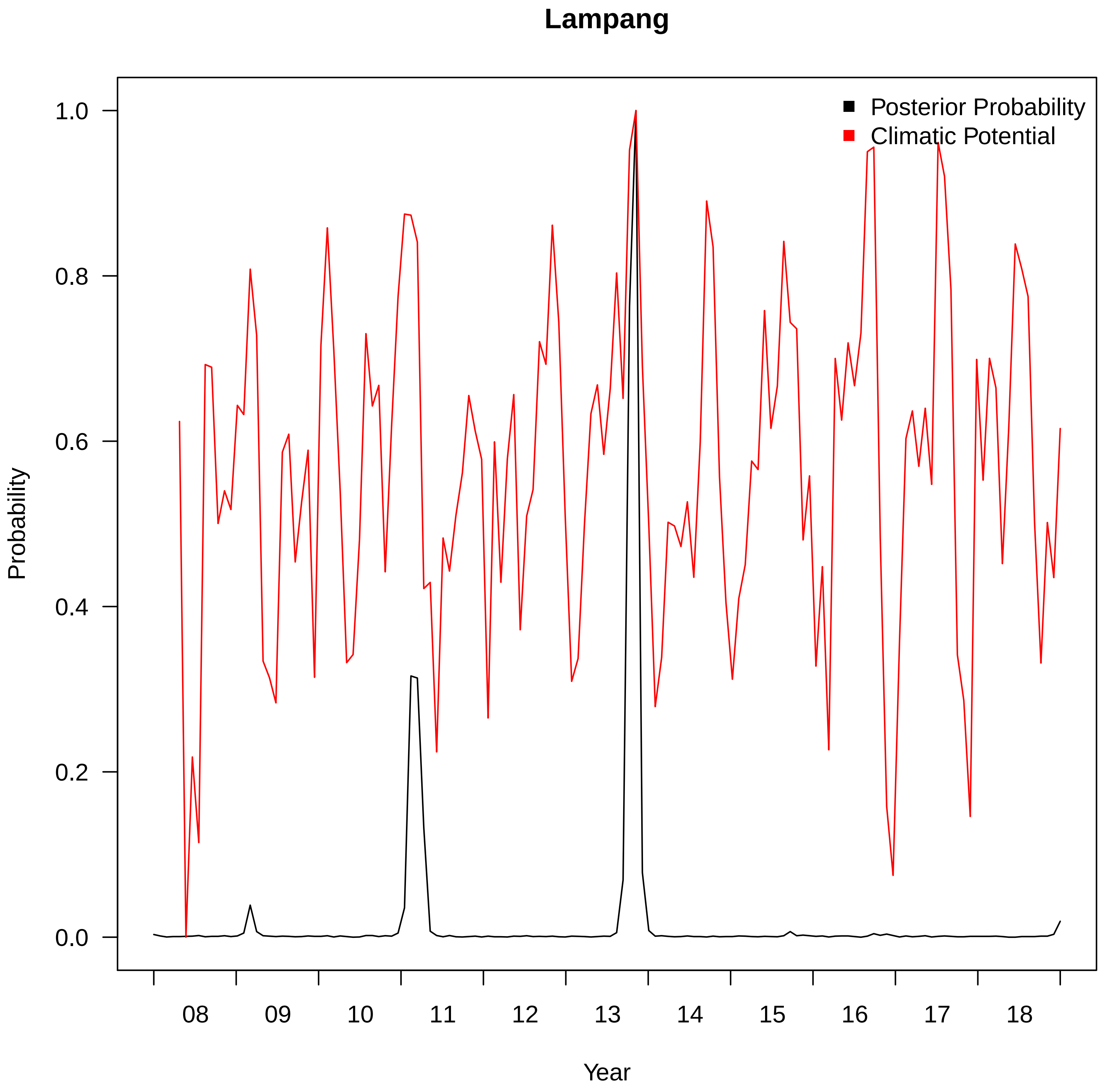
Lampang
Posterior Probability
Climatic Potential
Probability
1.0
0.8
0.6
0.4
0.2
0.0
08
09
10
11
12
13
14
15
16
17
18
Year

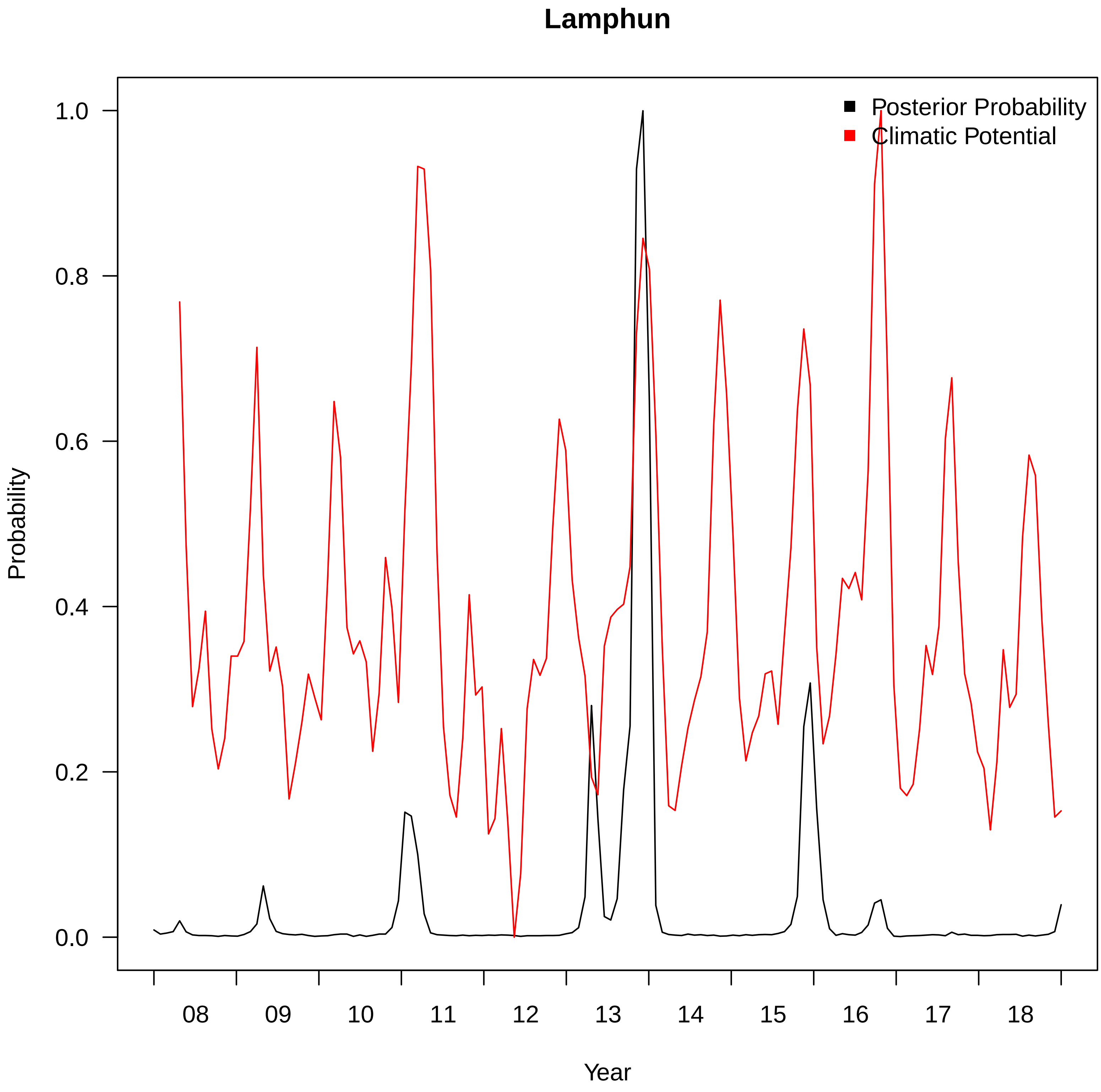

Lamphun
Posterior Probability
Climatic Potential
Probability
1.0
0.8
0.6
0.4
0.2
0.0
08
09
10
11
12
13
14
15
16
17
18
Year

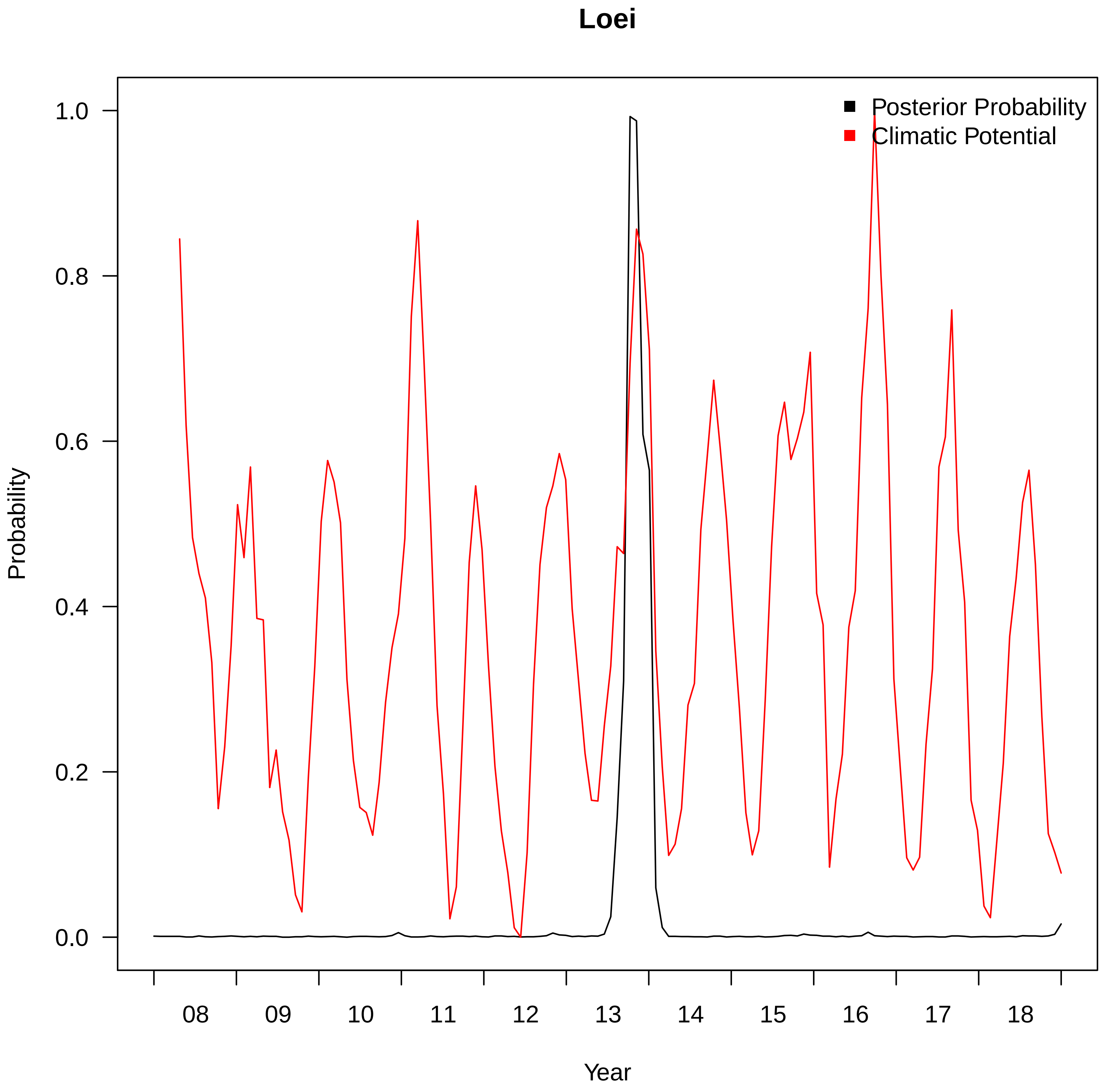

Loei
Posterior Probability
Climatic Potential
1.0
0.8
0.6
0.4
0.2
0.0
Probability
08
09
10
11
12
13
14
15
16
17
18
Year

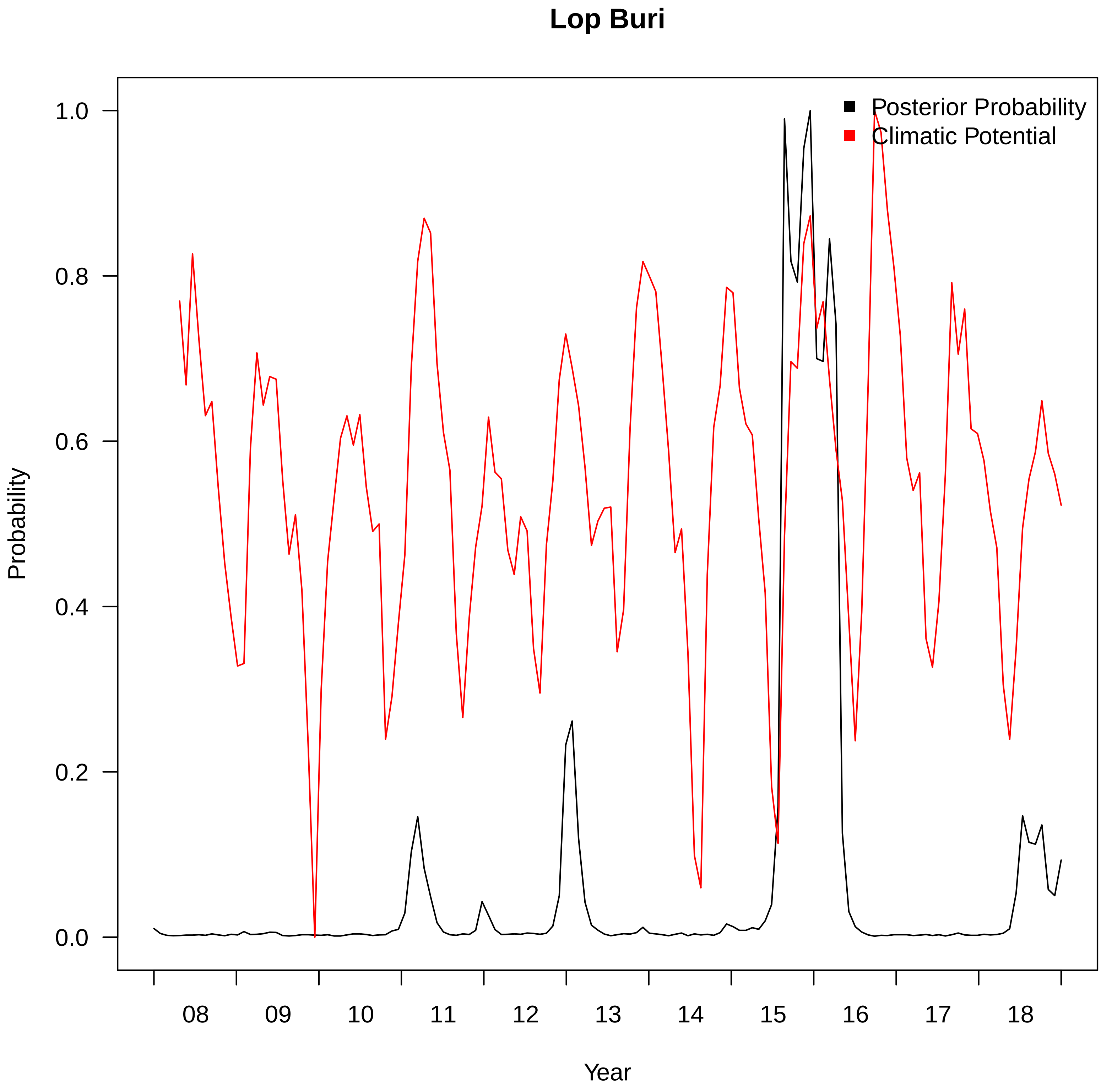

Lop Buri
Posterior Probability
Climatic Potential
Probability
1.0
0.8
0.6
0.4
0.2
0.0
08
09
10
11
12
13
14
15
16
17
18
Year

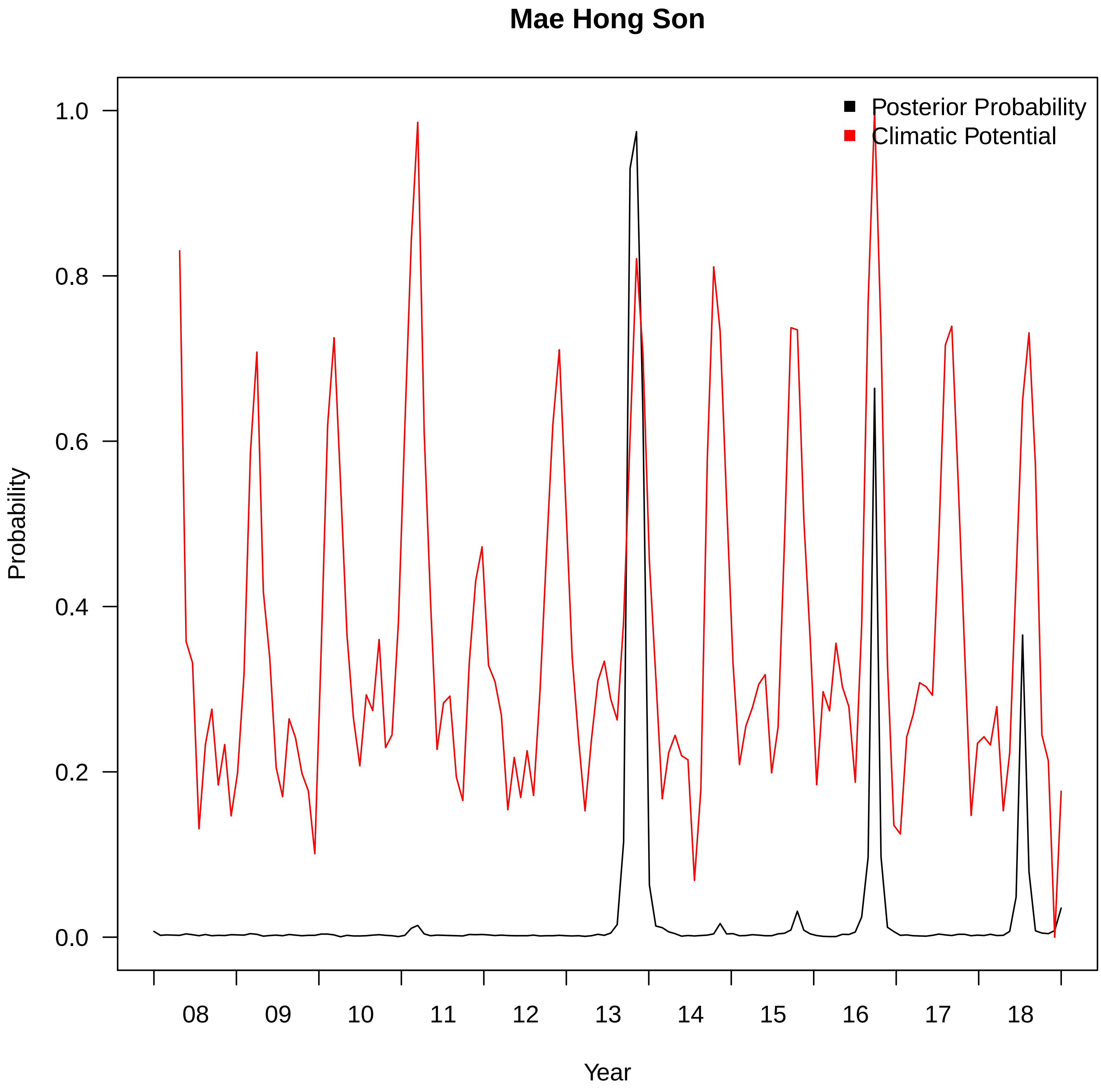
Mae Hong Son
Posterior Probability
Climatic Potential
Probability
0.0
0.2
0.4
0.6
0.8
1.0
08
09
10
11
12
13
14
15
16
17
18
Year

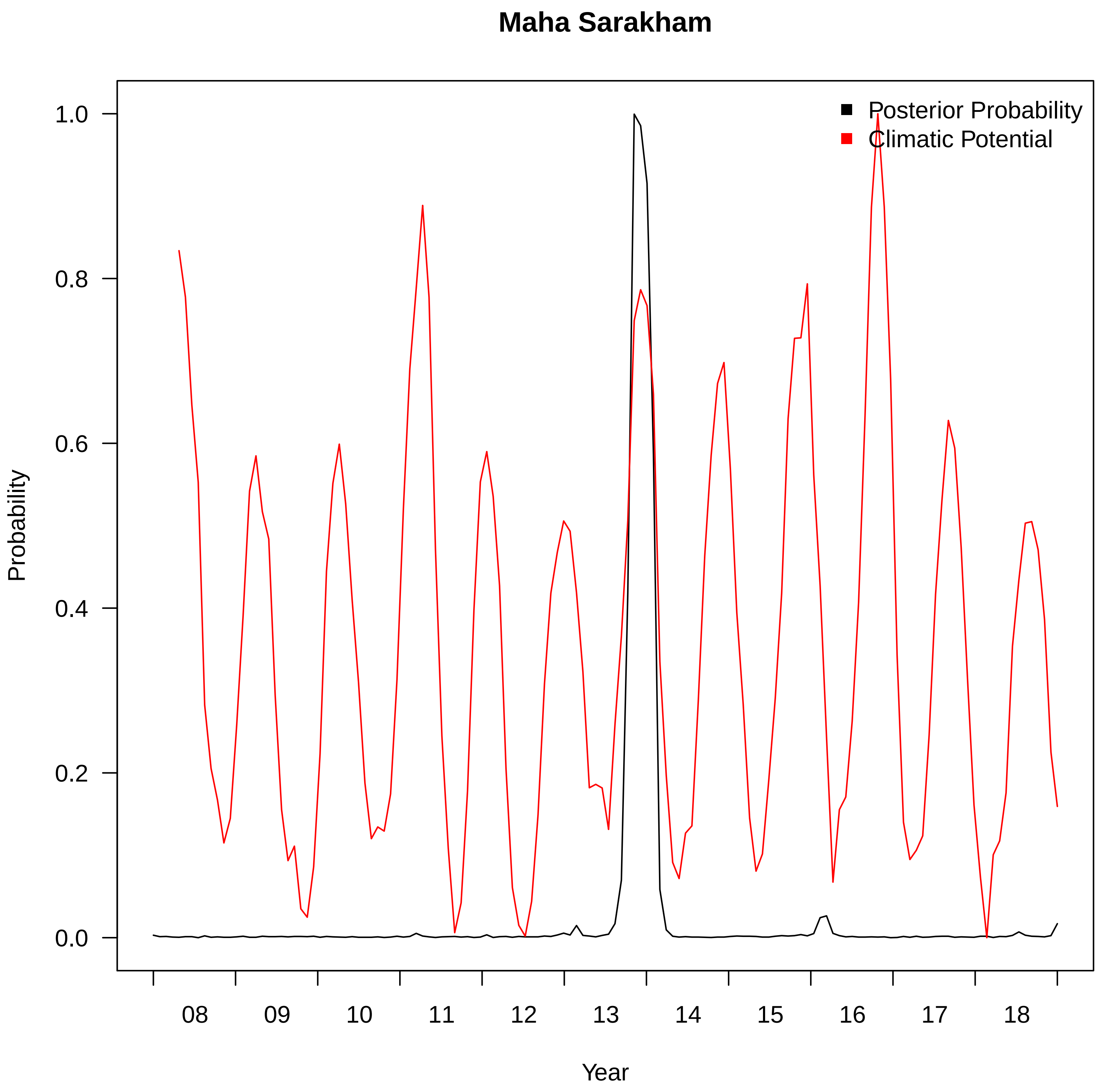
Maha Sarakham
Posterior Probability
Climatic Potential
Probability
0.0
0.2
0.4
0.6
0.8
1.0
08
09
10
11
12
13
14
15
16
17
18
Year

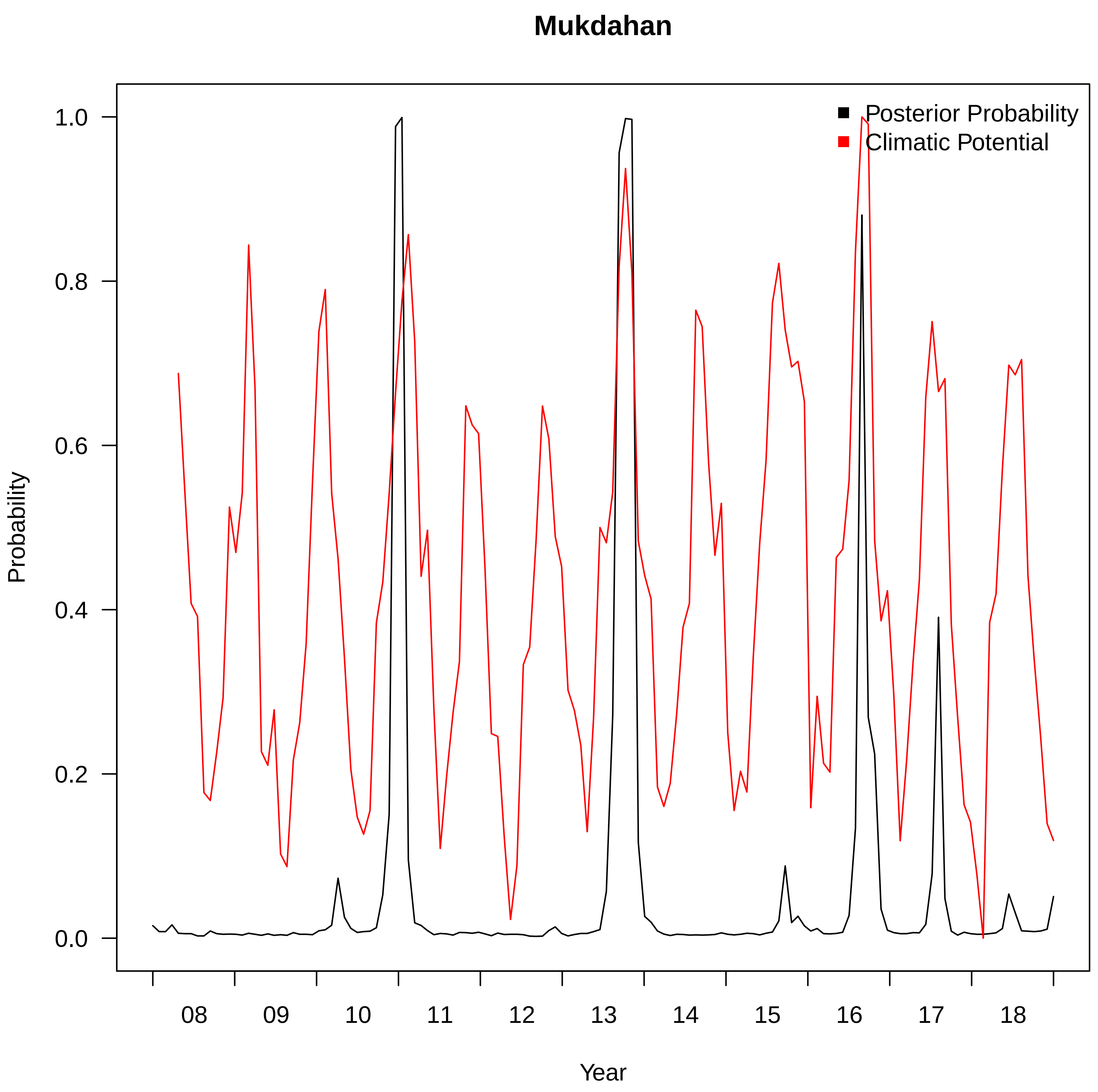

Mukdahan
Posterior Probability
Climatic Potential
Probability
Year
1.0
0.8
0.6
0.4
0.2
0.0
08
09
10
11
12
13
14
15
16
17
18

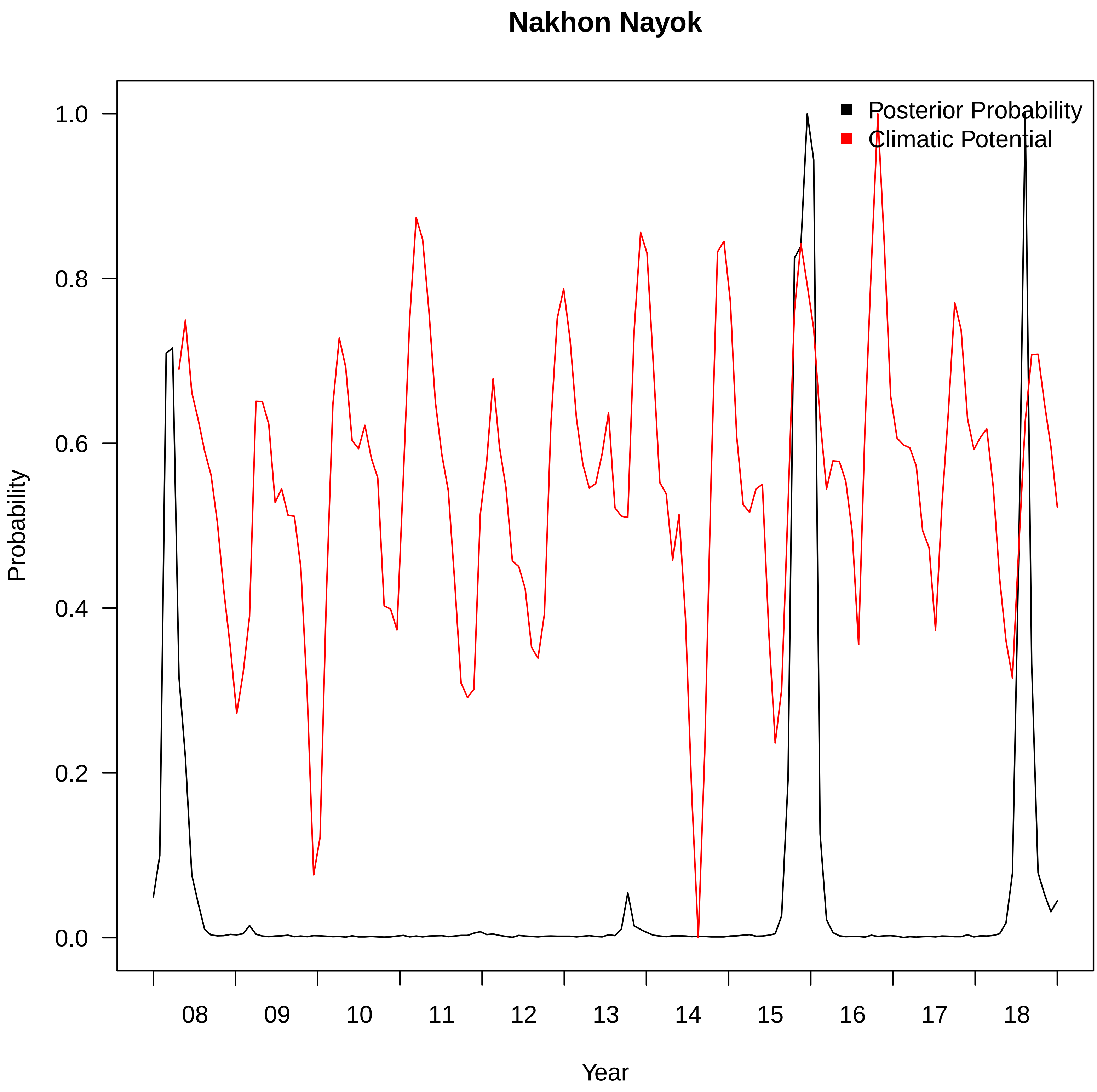
Nakhon Nayok
Posterior Probability
Climatic Potential
Probability
1.0
0.8
0.6
0.4
0.2
0.0
08
09
10
11
12
13
14
15
16
17
18
Year

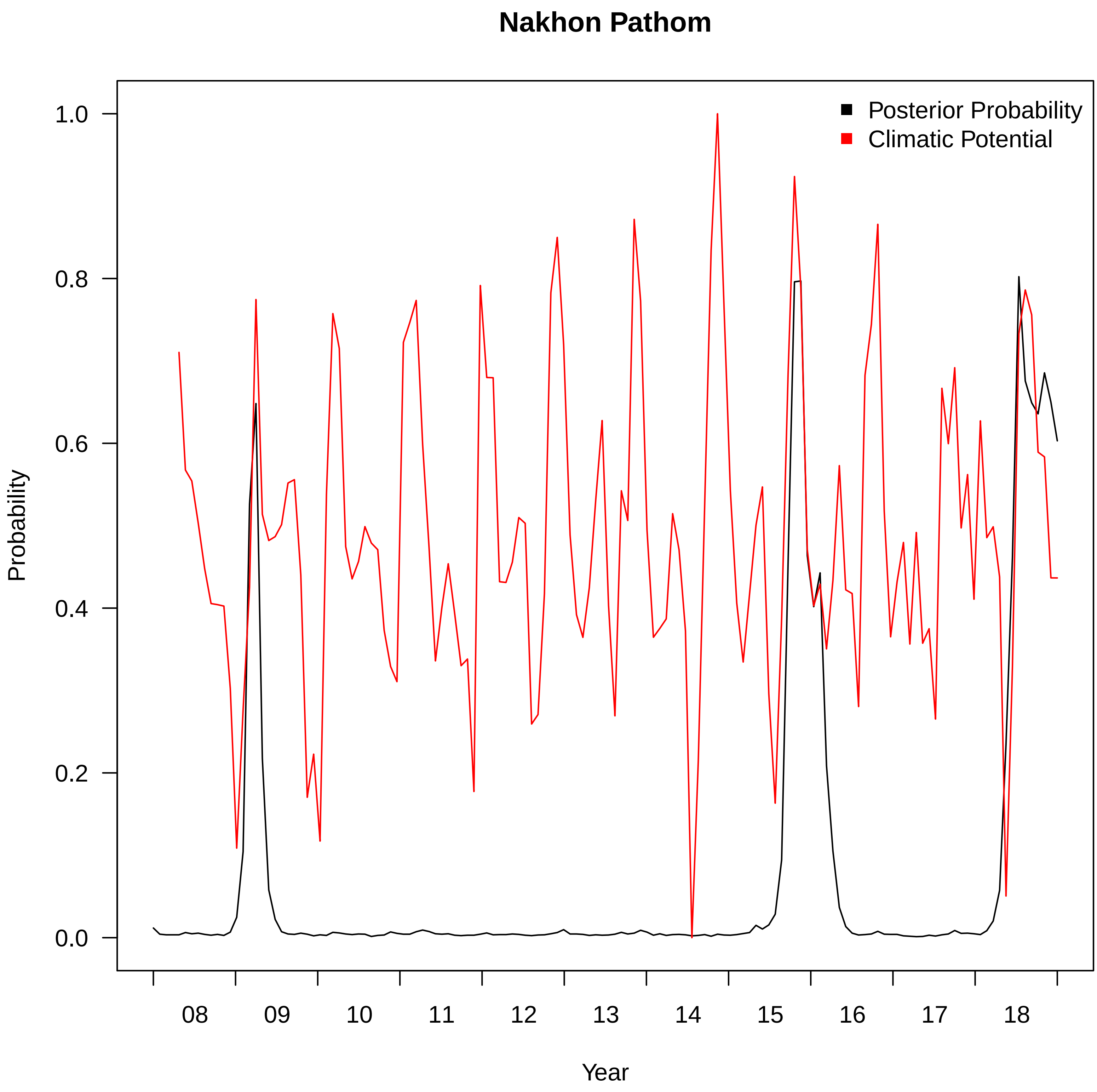
Nakhon Pathom
Posterior Probability
Climatic Potential
Probability
1.0
0.8
0.6
0.4
0.2
0.0
08
09
10
11
12
13
14
15
16
17
18
Year

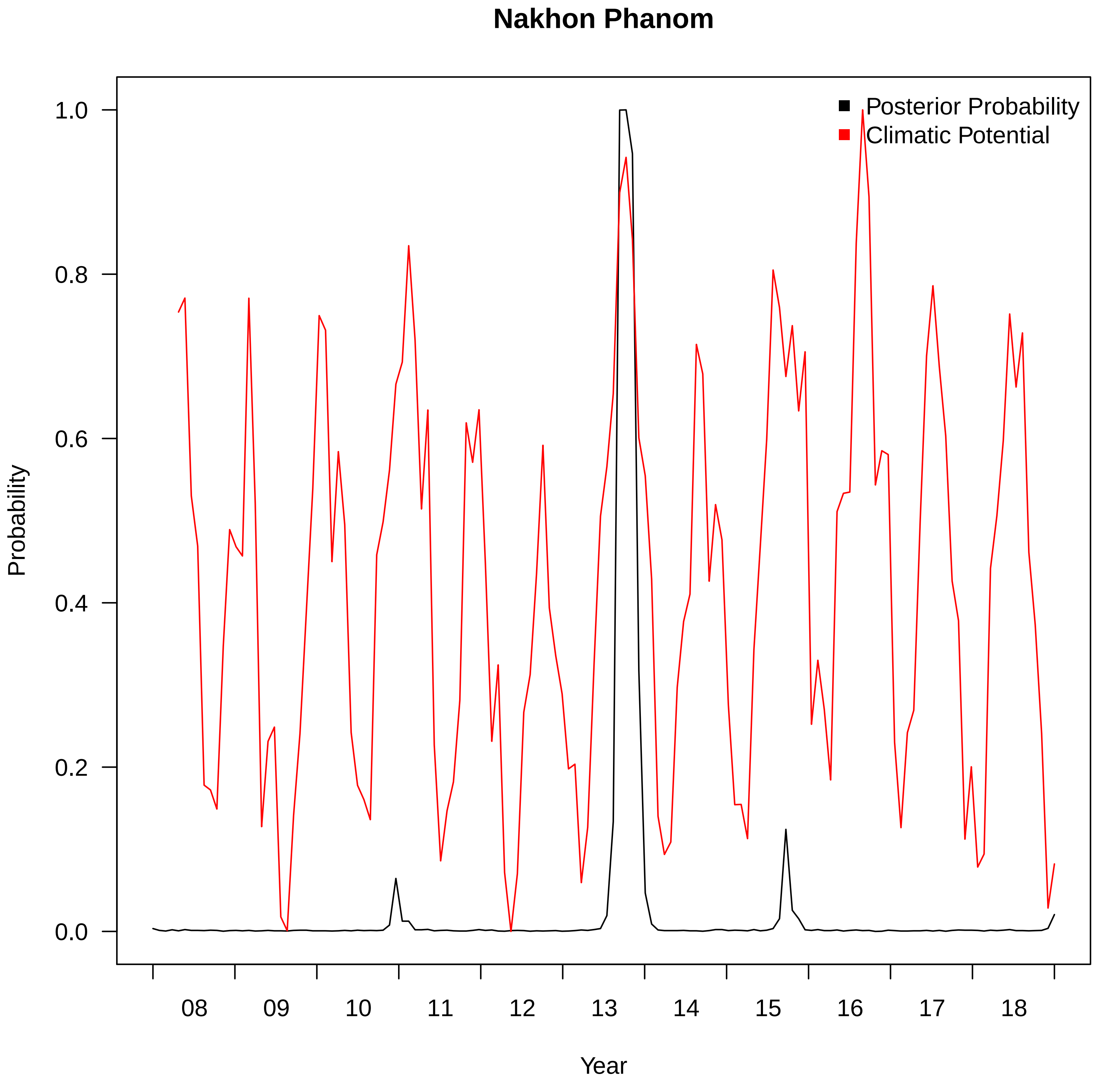

Nakhon Phanom
Posterior Probability
Climatic Potential
Probability
1.0
0.8
0.6
0.4
0.2
0.0
08
09
10
11
12
13
14
15
16
17
18
Year

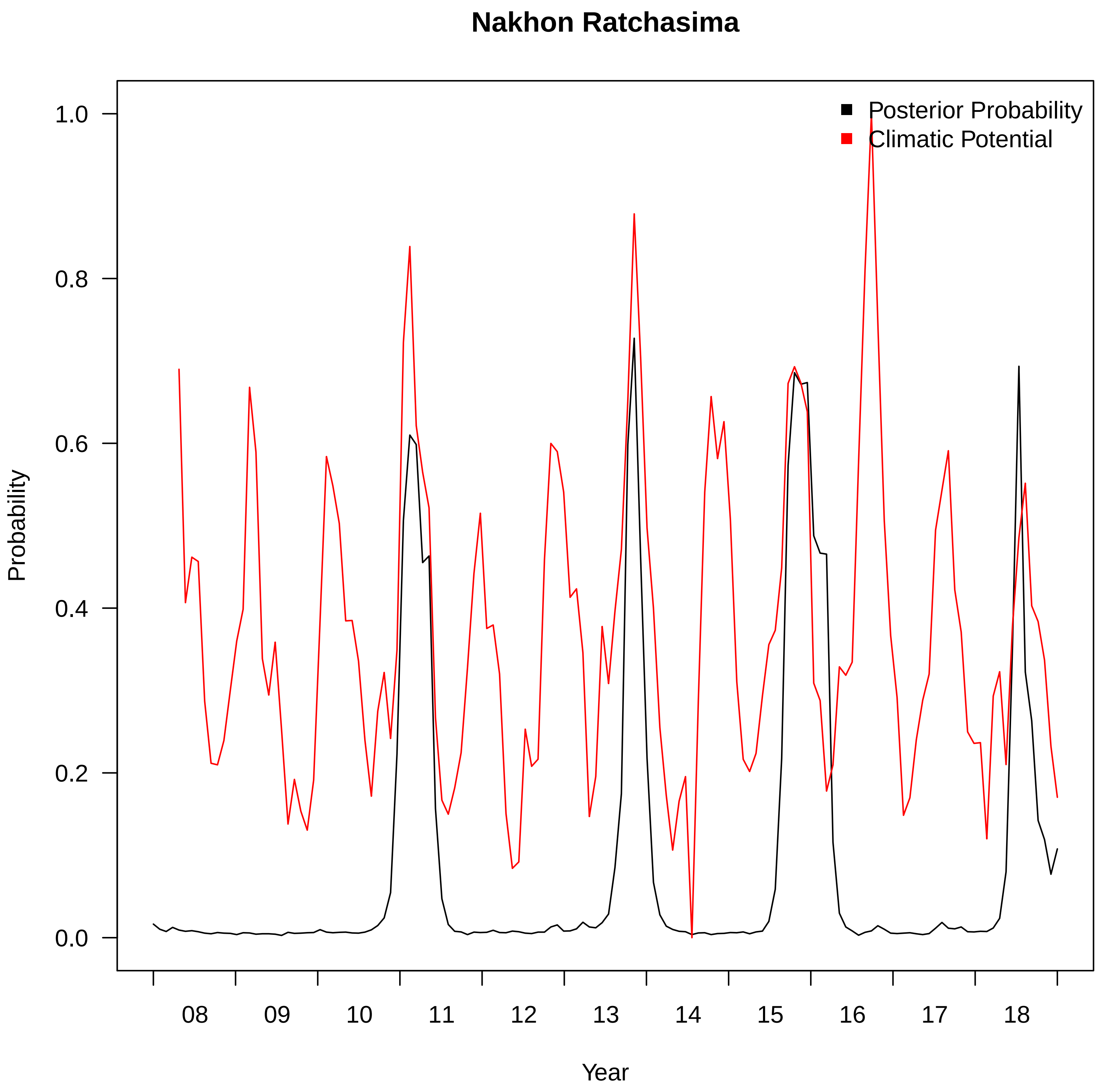
Nakhon Ratchasima
Posterior Probability
Climatic Potential
Probability
1.0
0.8
0.6
0.4
0.2
0.0
08
09
10
11
12
13
14
15
16
17
18
Year

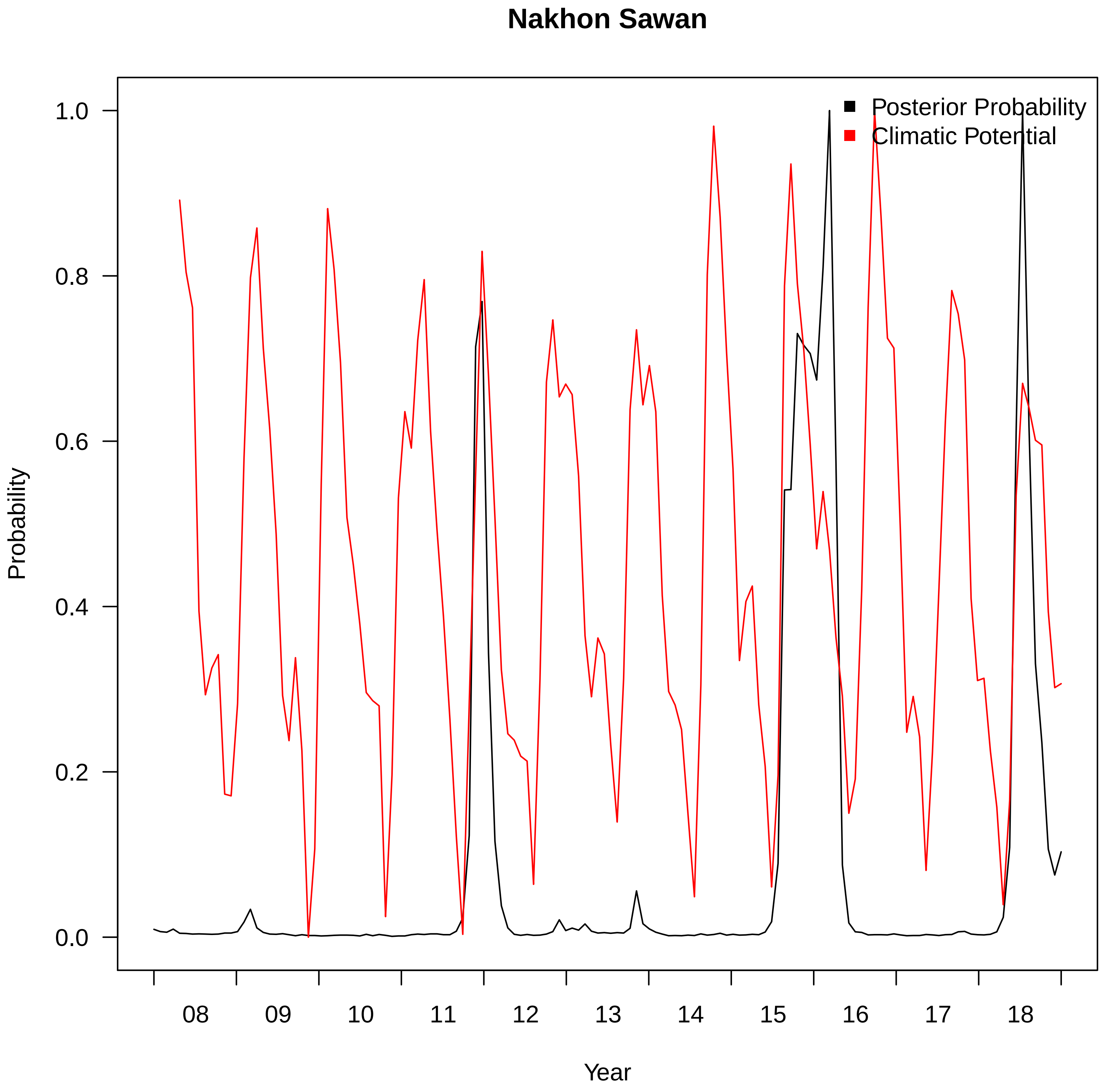
Nakhon Sawan
Posterior Probability
Climatic Potential
Probability
1.0
0.8
0.6
0.4
0.2
0.0
08
09
10
11
12
13
14
15
16
17
18
Year

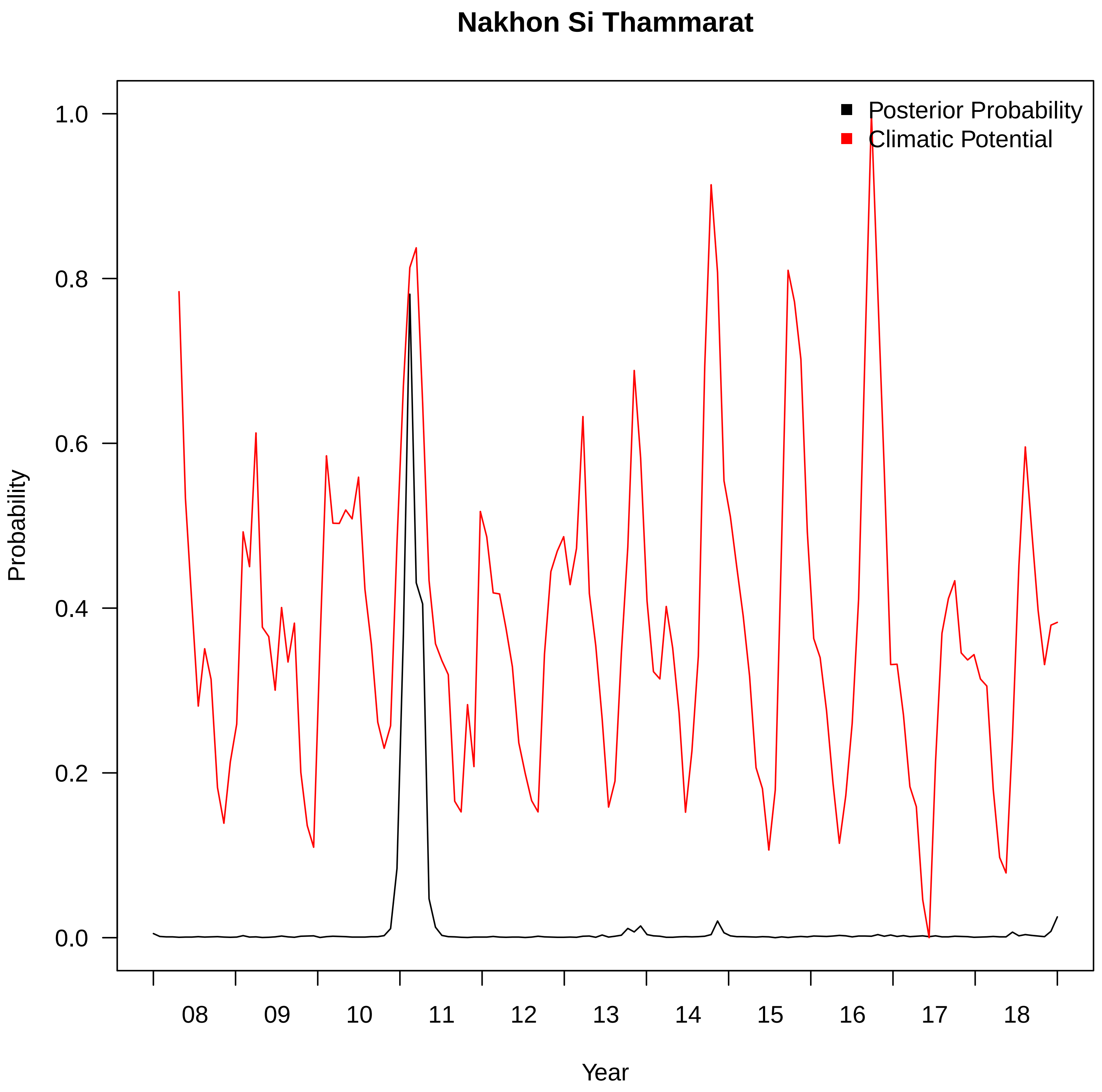
Nakhon Si Thammarat
Posterior Probability
Climatic Potential
1.0
0.8
0.6
0.4
0.2
0.0
Probability
08
09
10
11
12
13
14
15
16
17
18
Year

Nan

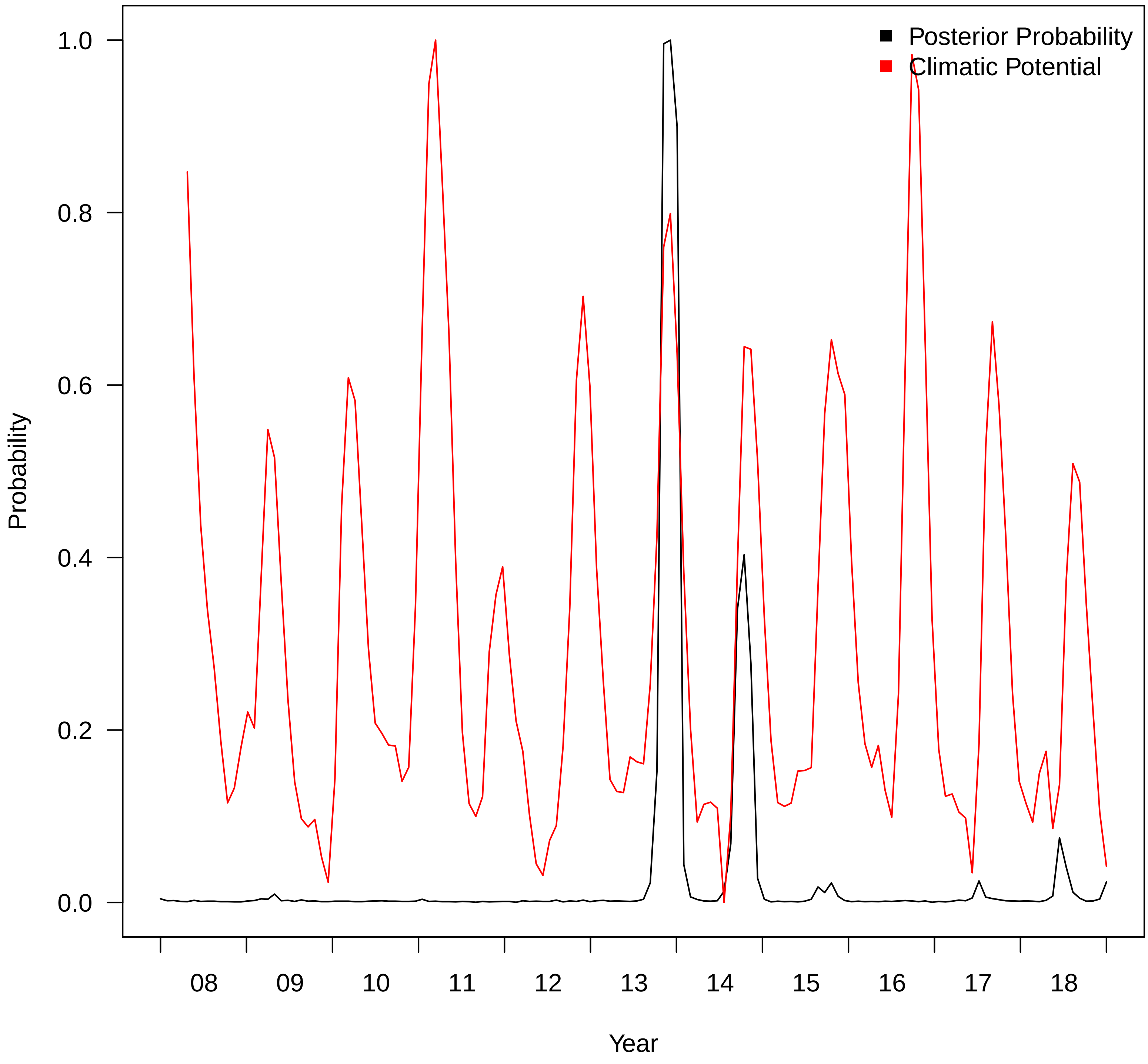

Posterior Probability
Climatic Potential
Probability
1.0
0.8
0.6
0.4
0.2
0.0
08
09
10
11
12
13
14
15
16
17
18
Year

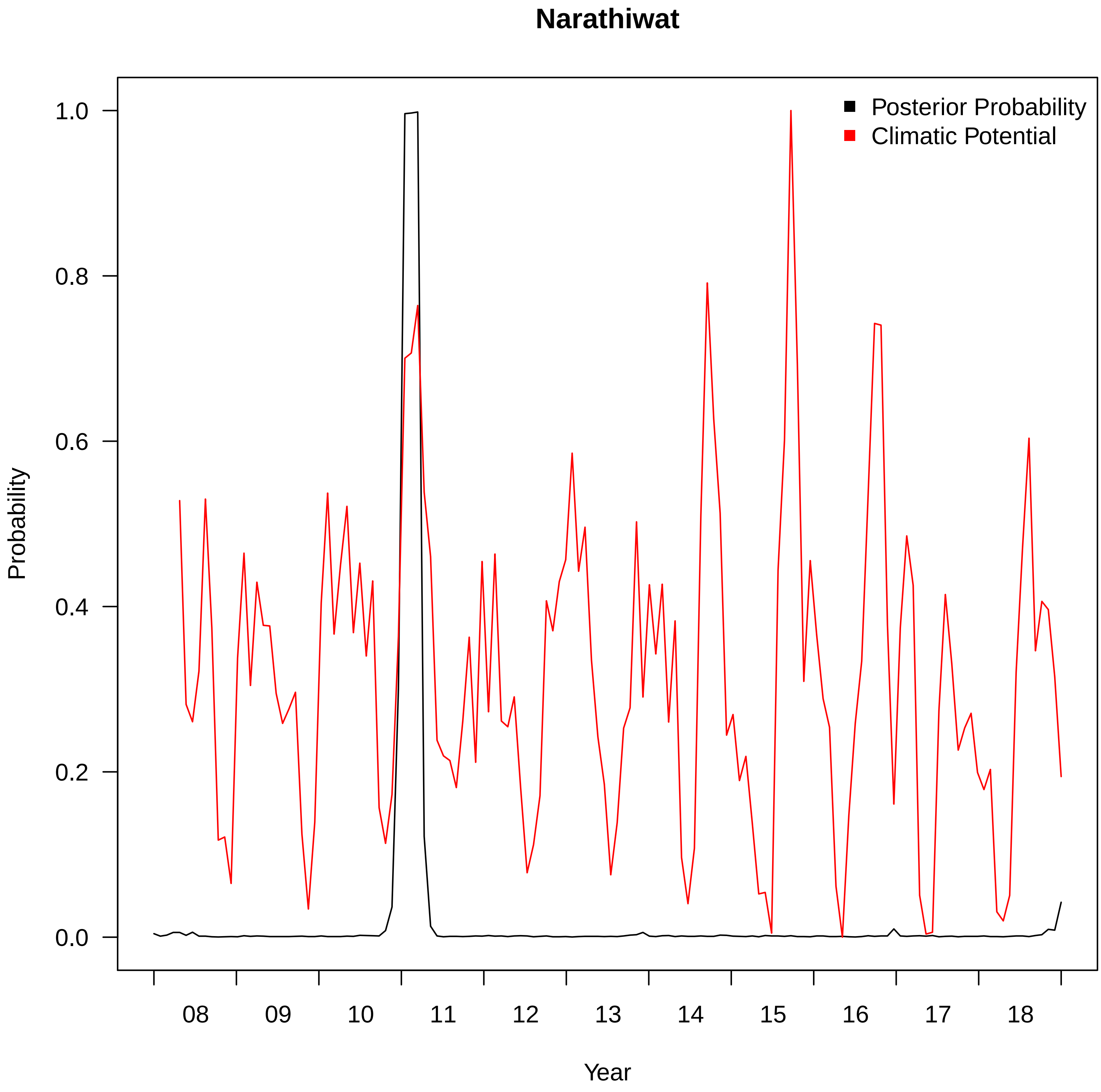
Narathiwat
Posterior Probability
Climatic Potential
Probability
1.0
0.8
0.6
0.4
0.2
0.0
08
09
10
11
12
13
14
15
16
17
18
Year

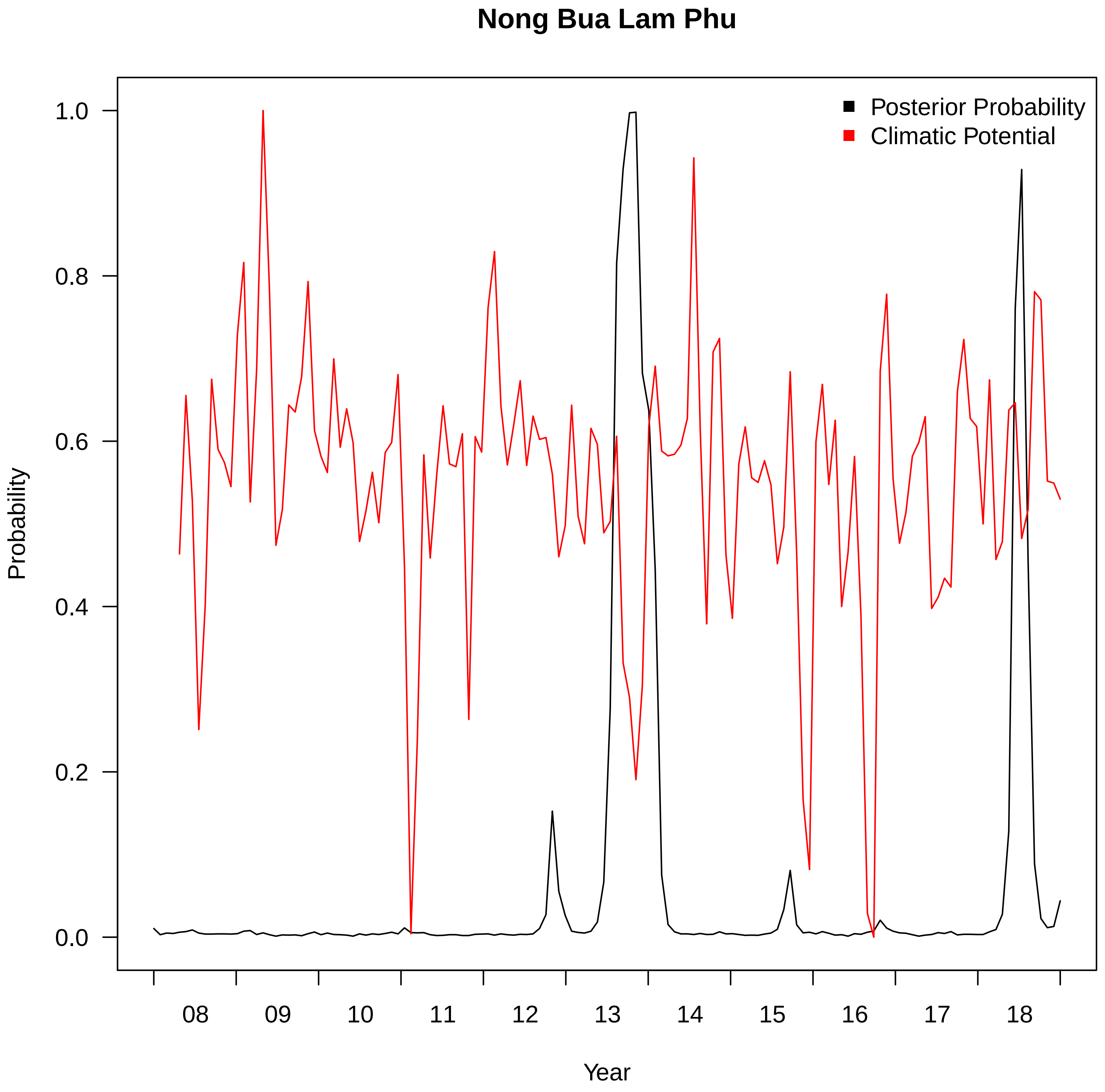
Nong Bua Lam Phu
Posterior Probability
Climatic Potential
Probability
1.0
0.8
0.6
0.4
0.2
0.0
08
09
10
11
12
13
14
15
16
17
18
Year

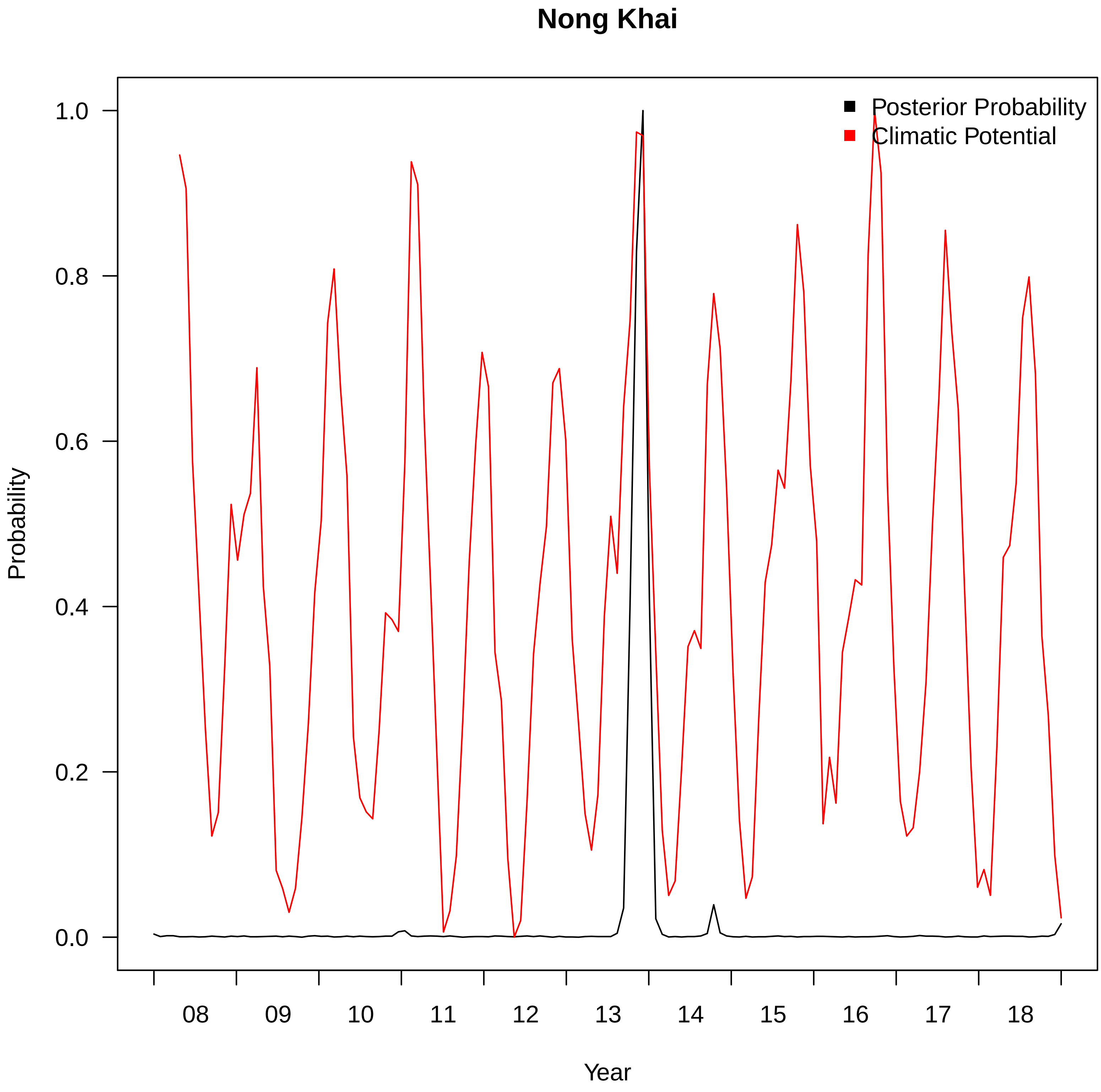

Nong Khai
Posterior Probability
Climatic Potential
Probability
1.0
0.8
0.6
0.4
0.2
0.0
08
09
10
11
12
13
14
15
16
17
18
Year

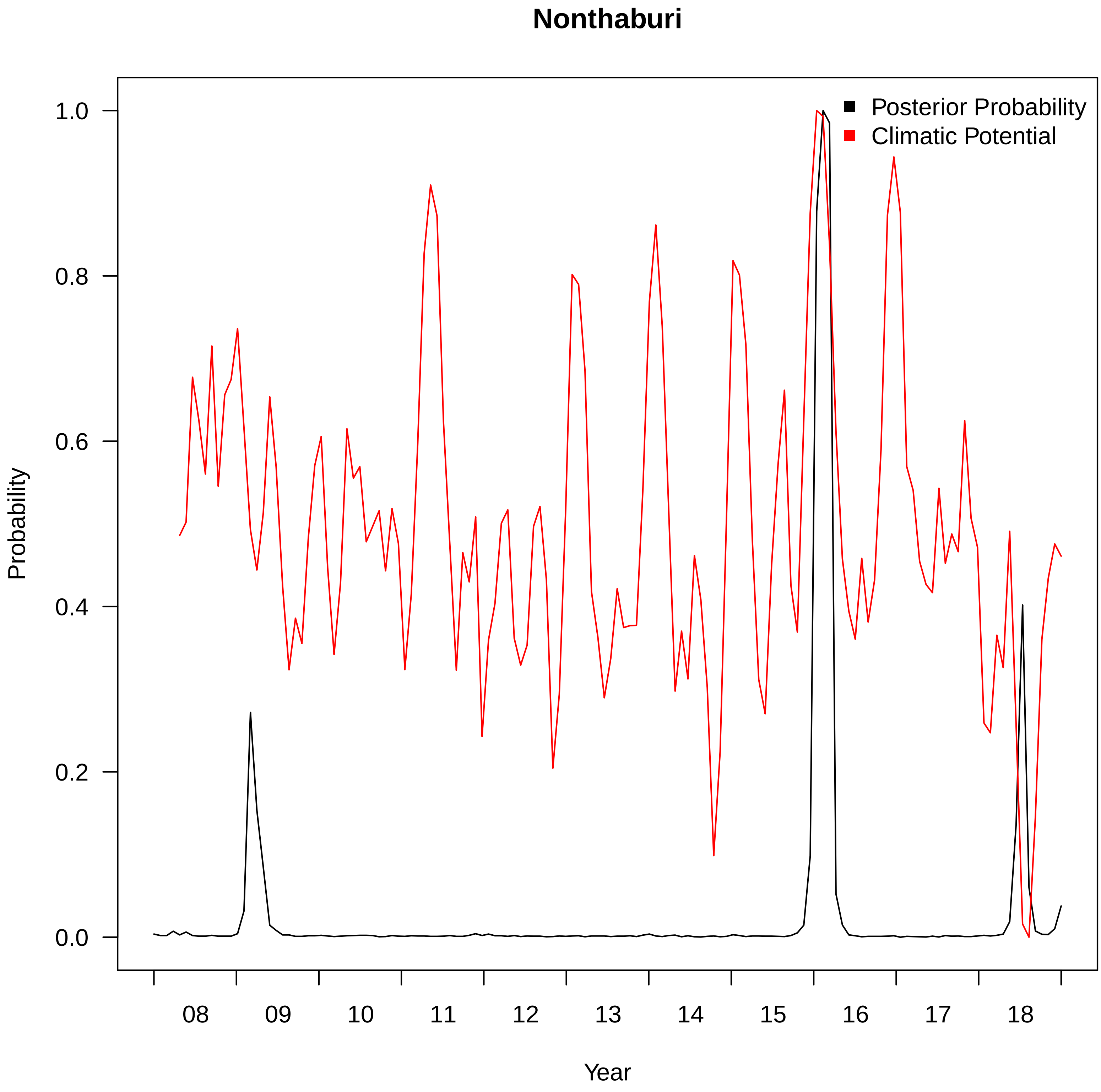
Nonthaburi
Posterior Probability
Climatic Potential
Probability
1.0
0.8
0.6
0.4
0.2
0.0
08
09
10
11
12
13
14
15
16
17
18
Year

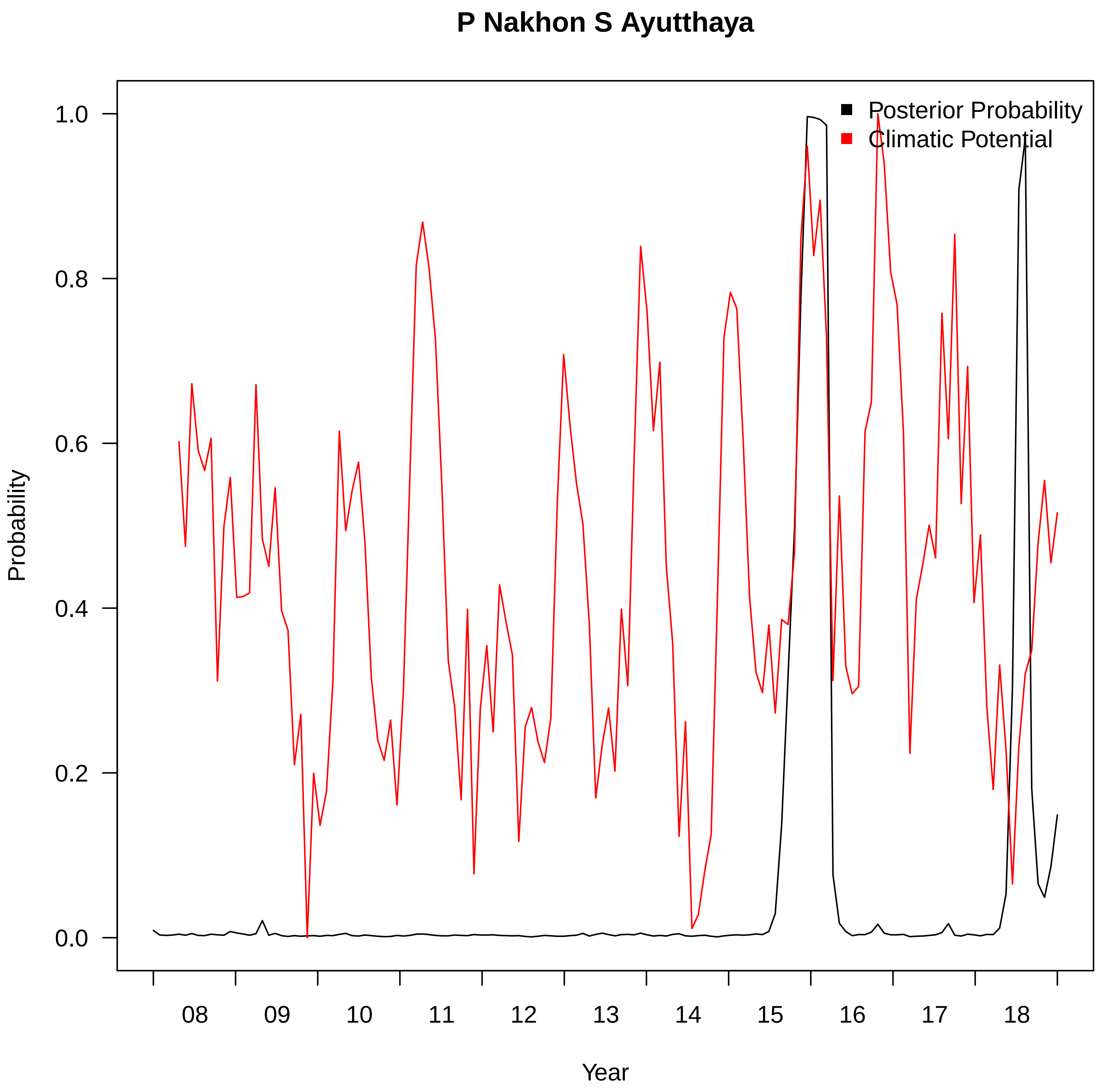
P Nakhon S Ayutthaya
Posterior Probability
Climatic Potential
Probability
1.0
0.8
0.6
0.4
0.2
0.0
08
09
10
11
12
13
14
15
16
17
18
Year

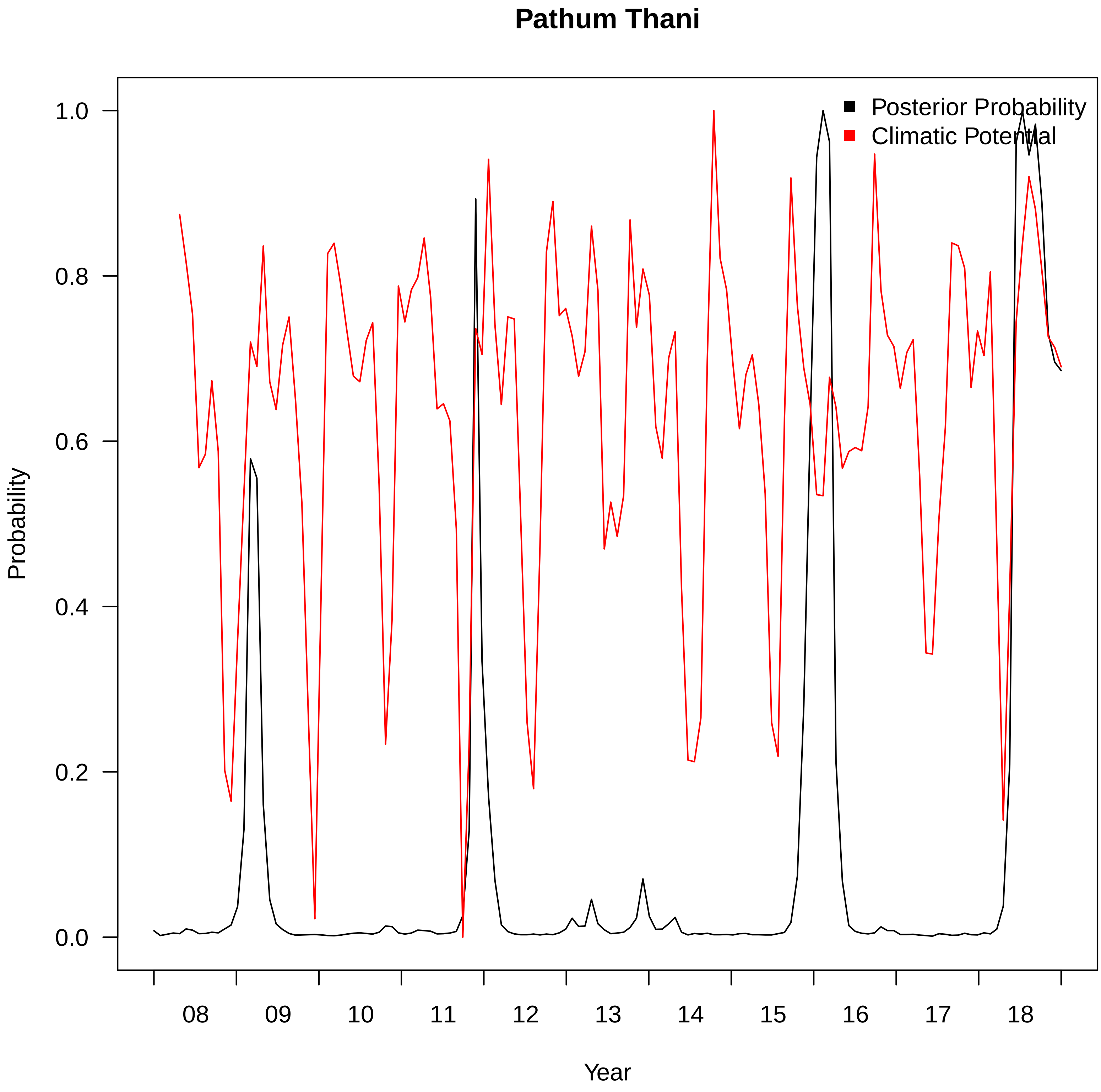
Pathum Thani
Posterior Probability
Climatic Potential
Probability
1.0
0.8
0.6
0.4
0.2
0.0
08
09
10
11
12
13
14
15
16
17
18
Year

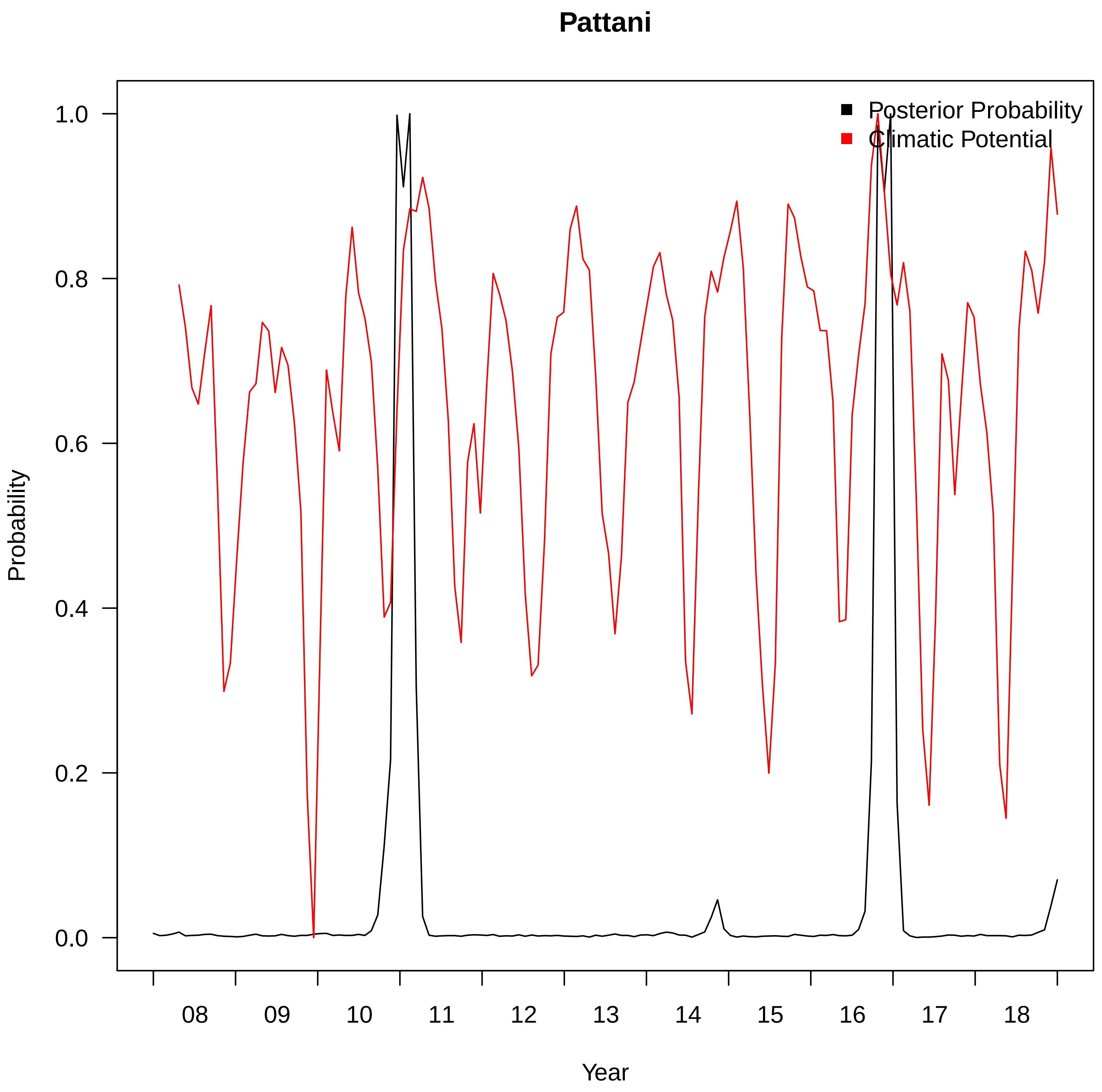

Pattani
Posterior Probability
Climatic Potential
Probability
1.0
0.8
0.6
0.4
0.2
0.0
08
09
10
11
12
13
14
15
16
17
18
Year

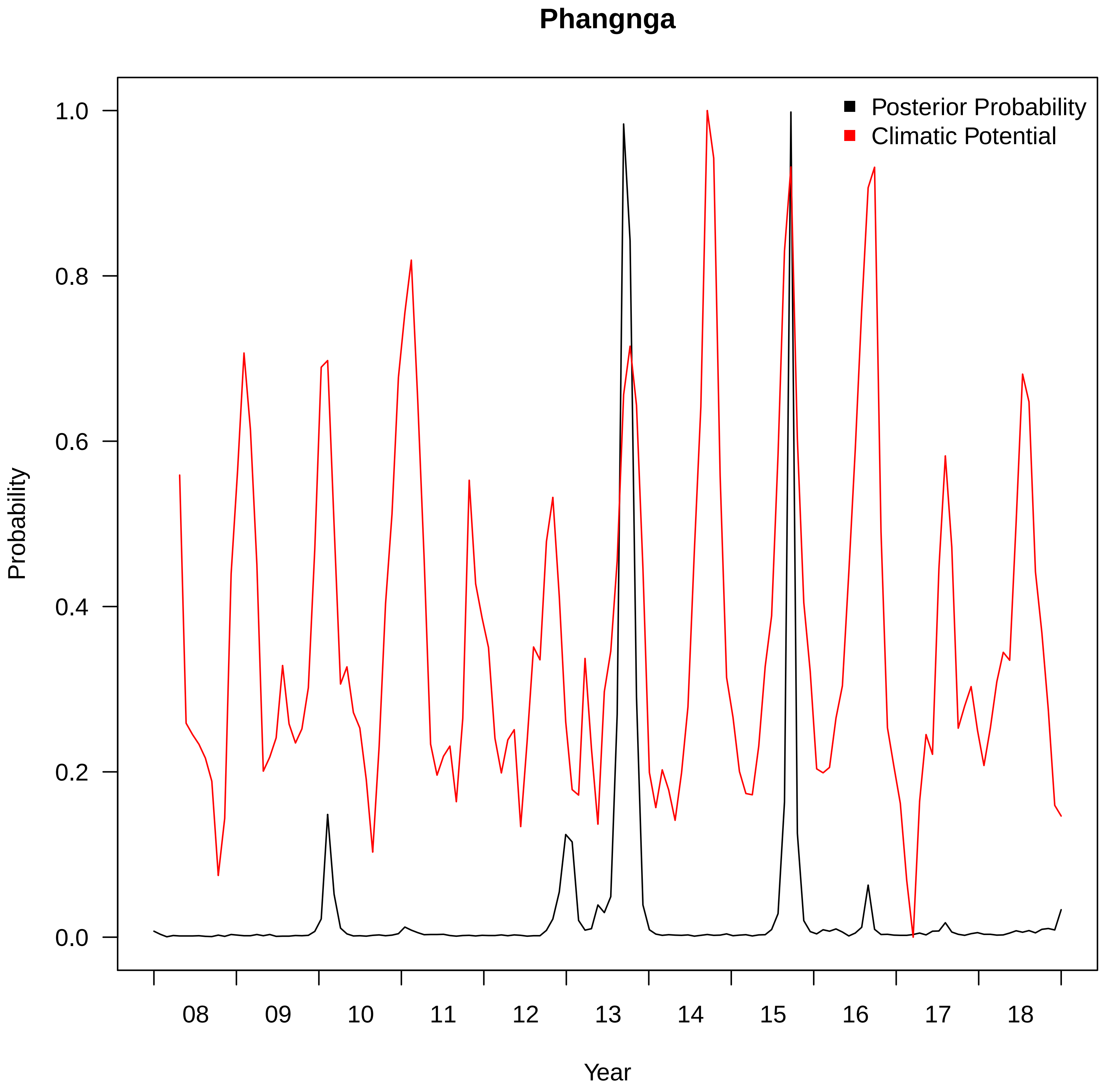
Phangnga
Posterior Probability
Climatic Potential
Probability
1.0
0.8
0.6
0.4
0.2
0.0
08
09
10
11
12
13
14
15
16
17
18
Year

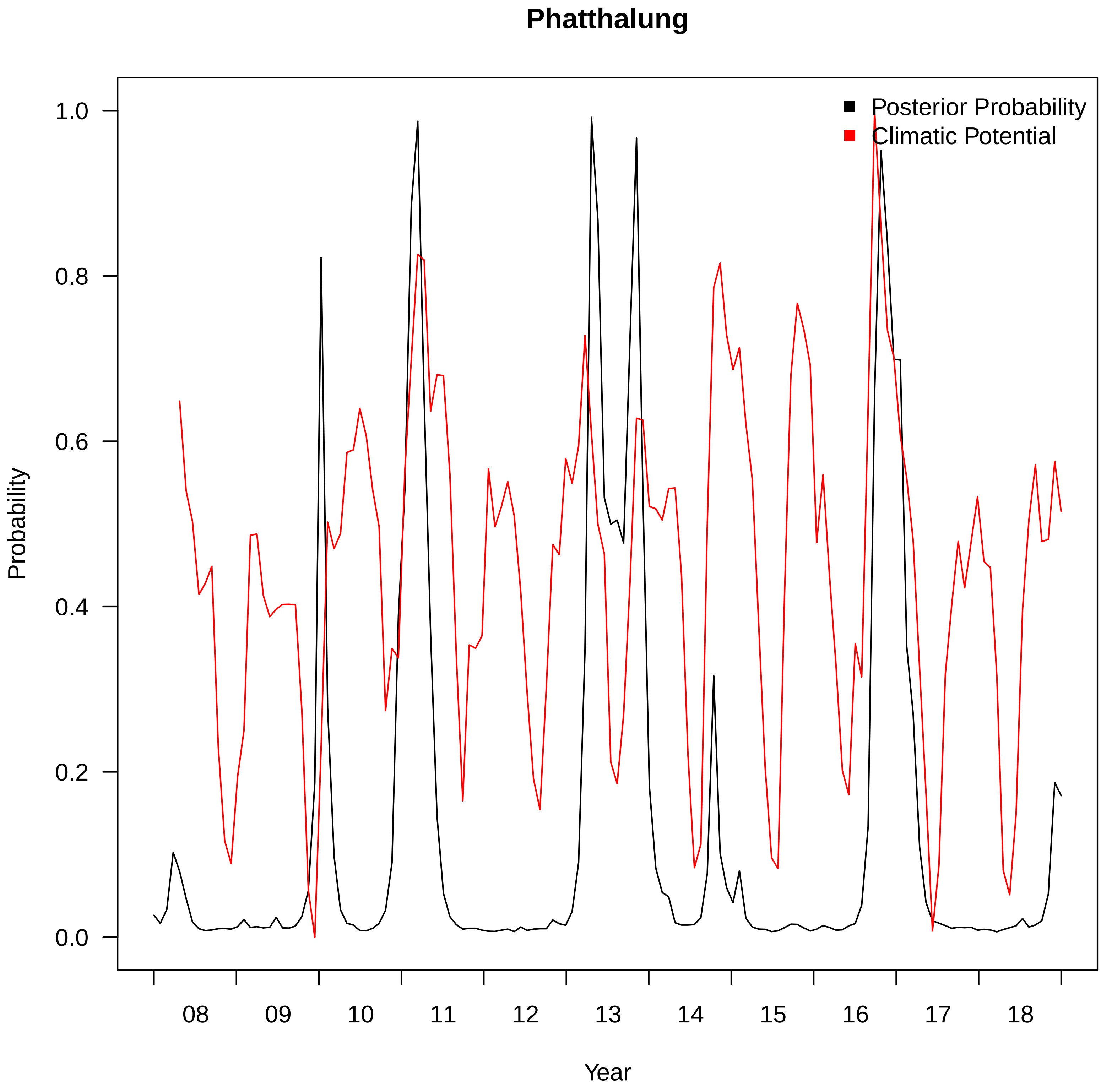
Phatthalung
Posterior Probability
Climatic Potential
Probability
1.0
0.8
0.6
0.4
0.2
0.0
08
09
10
11
12
13
14
15
16
17
18
Year

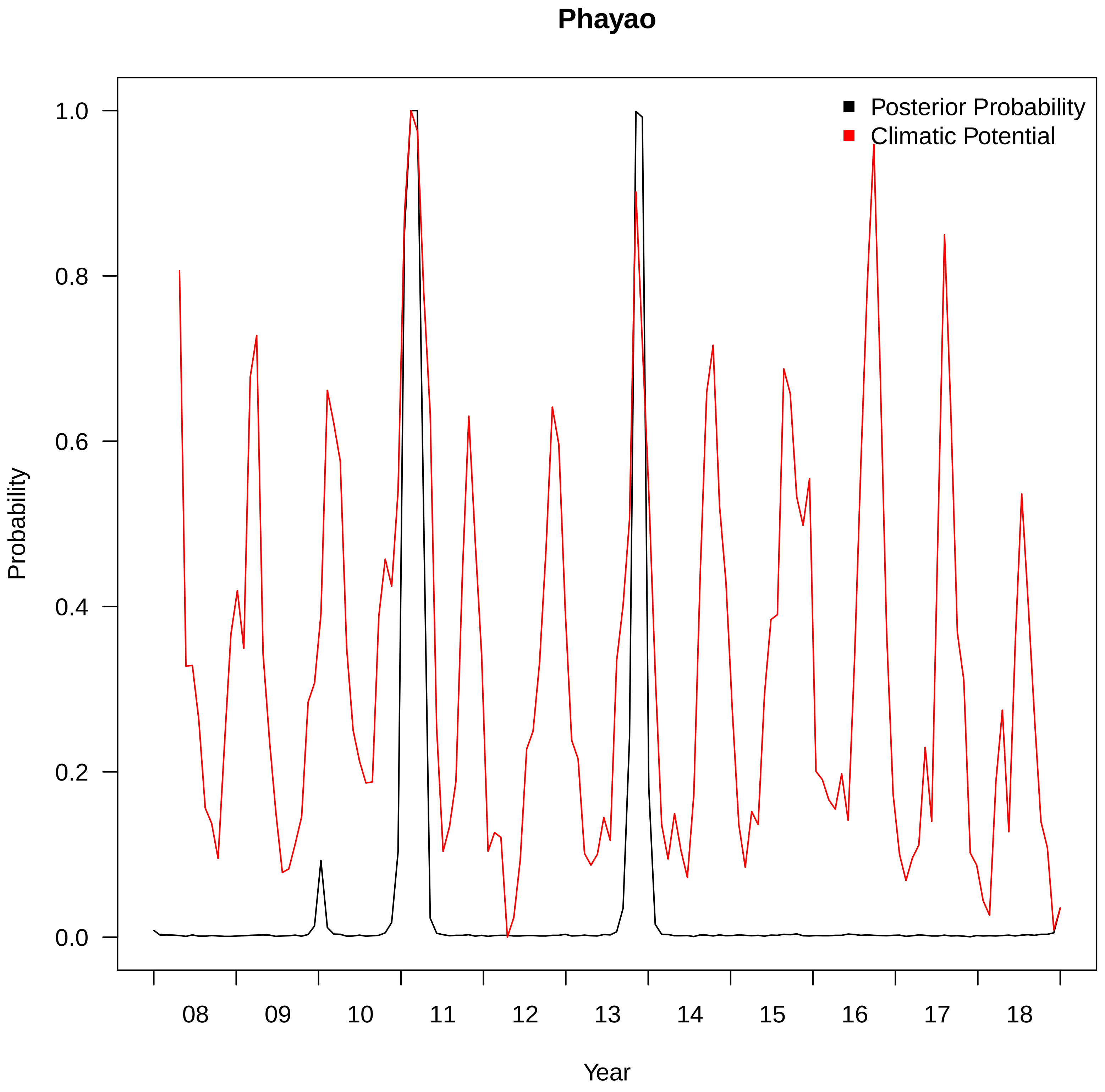
Phayao
Posterior Probability
Climatic Potential
Probability
1.0
0.8
0.6
0.4
0.2
0.0
08
09
10
11
12
13
14
15
16
17
18
Year

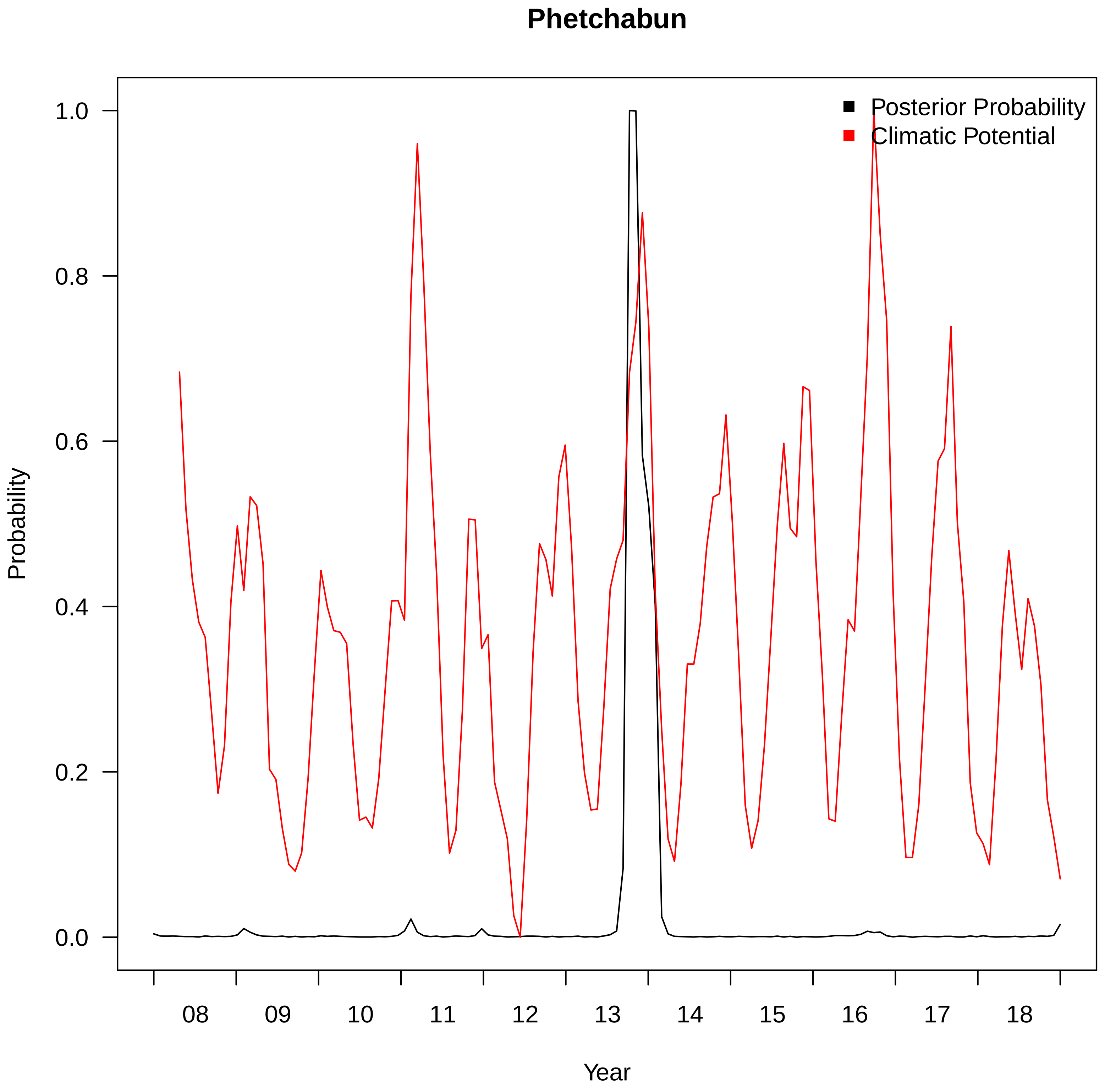

Phetchabun
Posterior Probability
Climatic Potential
Probability
1.0
0.8
0.6
0.4
0.2
0.0
08
09
10
11
12
13
14
15
16
17
18
Year

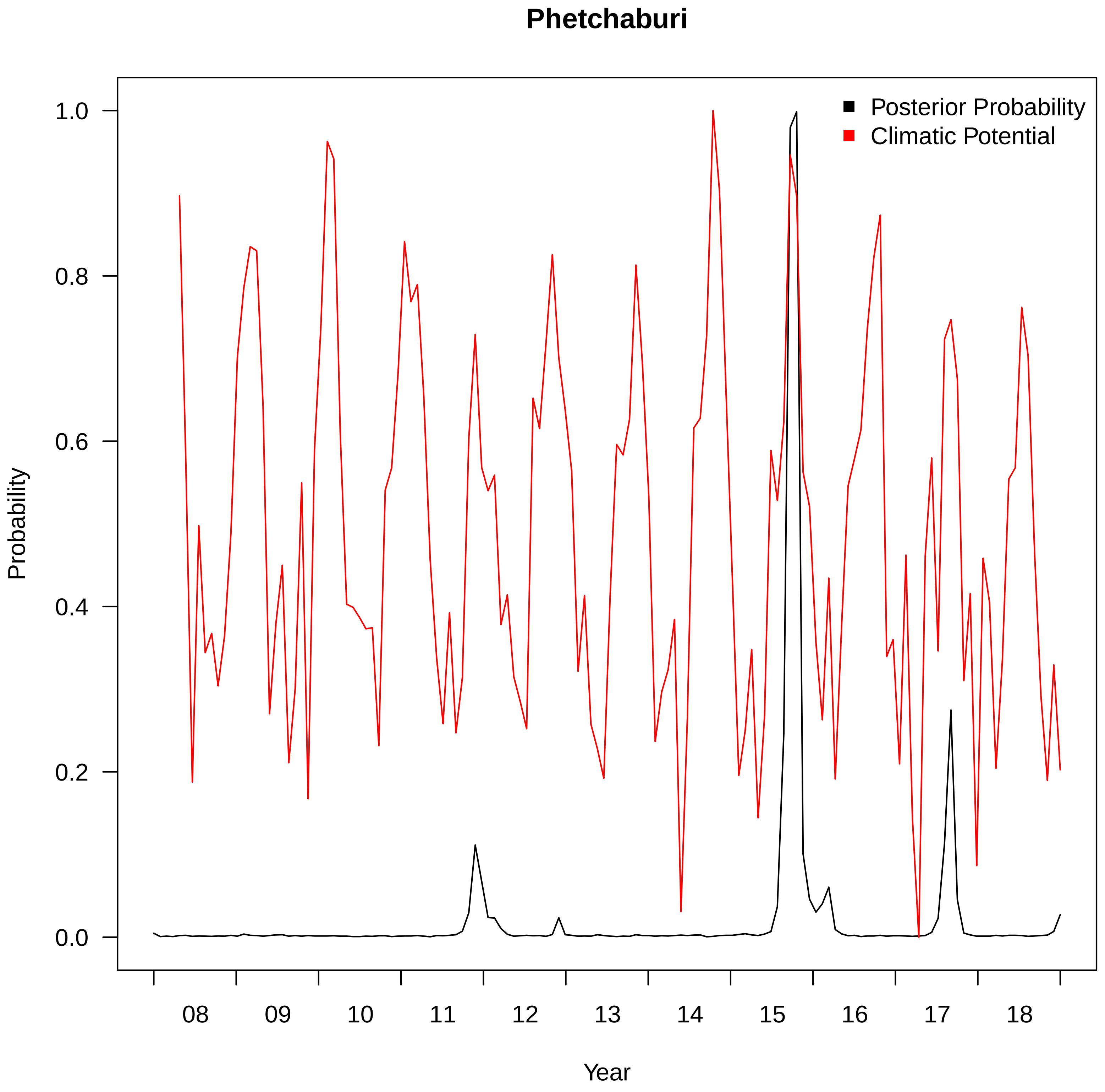
Phetchaburi
Posterior Probability
Climatic Potential
Probability
1.0
0.8
0.6
0.4
0.2
0.0
08
09
10
11
12
13
14
15
16
17
18
Year

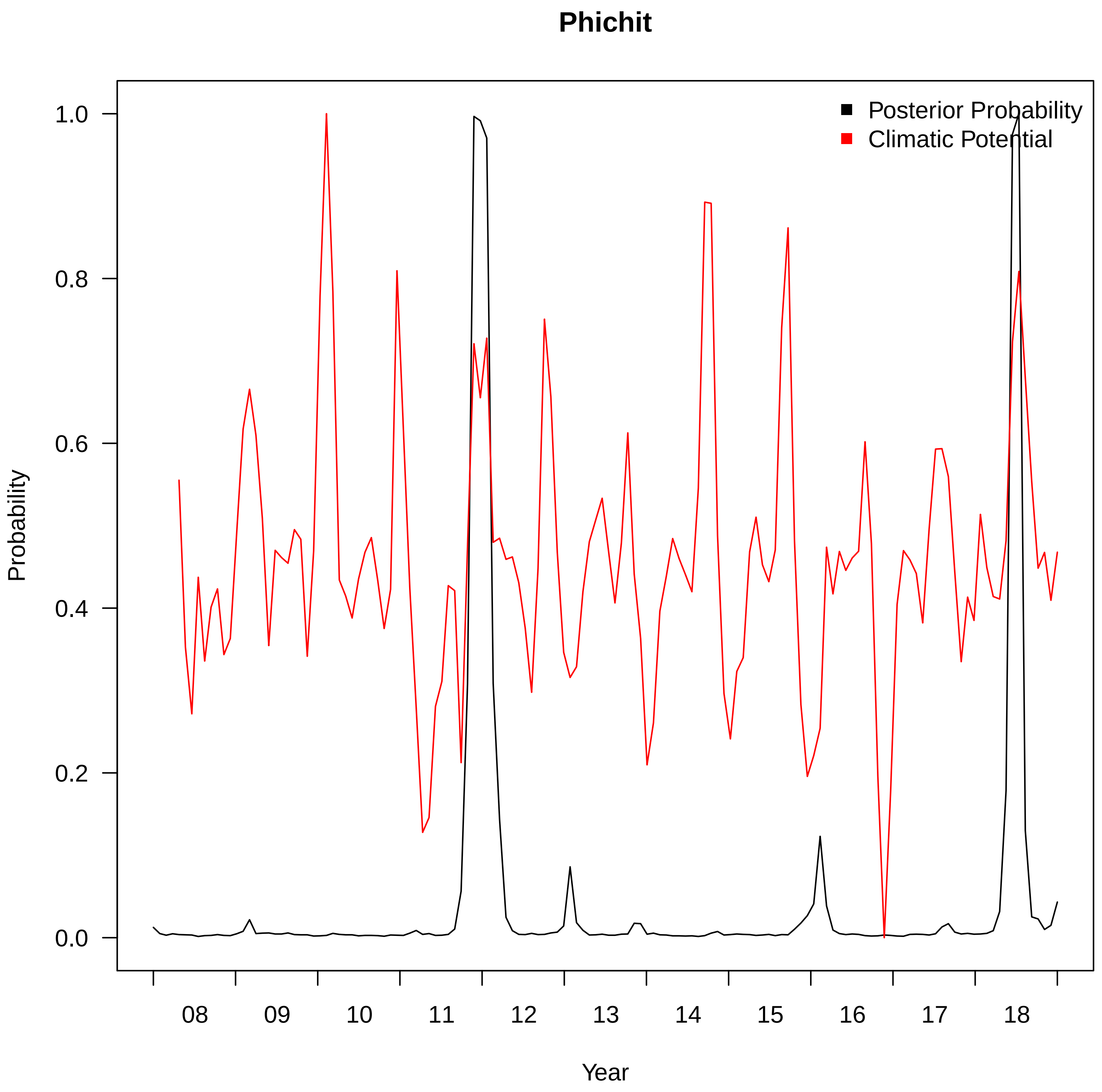
Phichit
Posterior Probability
Climatic Potential
Probability
1.0
0.8
0.6
0.4
0.2
0.0
08
09
10
11
12
13
14
15
16
17
18
Year

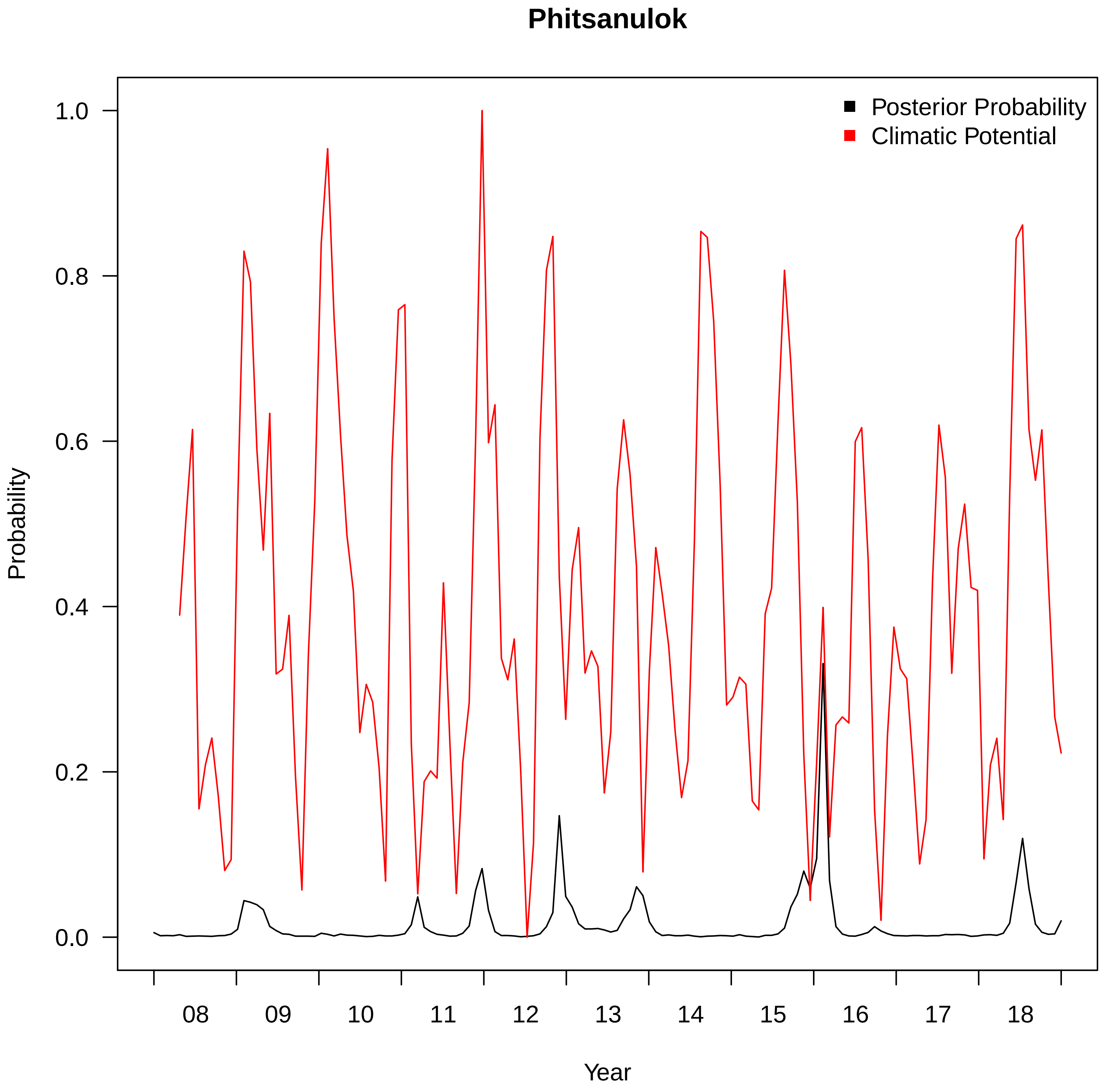

Phitsanulok
Posterior Probability
Climatic Potential
Probability
1.0
0.8
0.6
0.4
0.2
0.0
08
09
10
11
12
13
14
15
16
17
18
Year

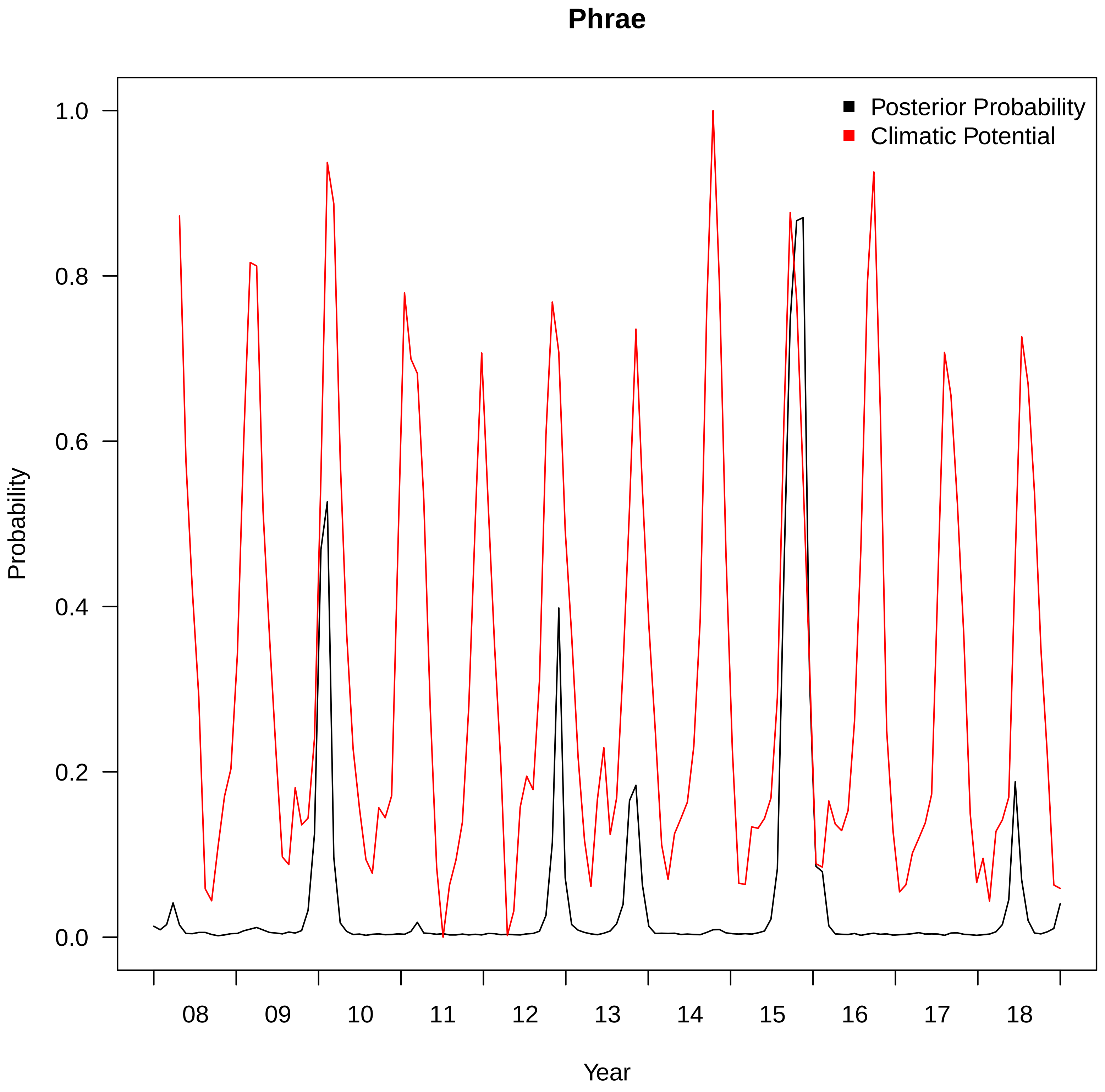
Phrae
Posterior Probability
Climatic Potential
Probability
1.0
0.8
0.6
0.4
0.2
0.0
08
09
10
11
12
13
14
15
16
17
18
Year

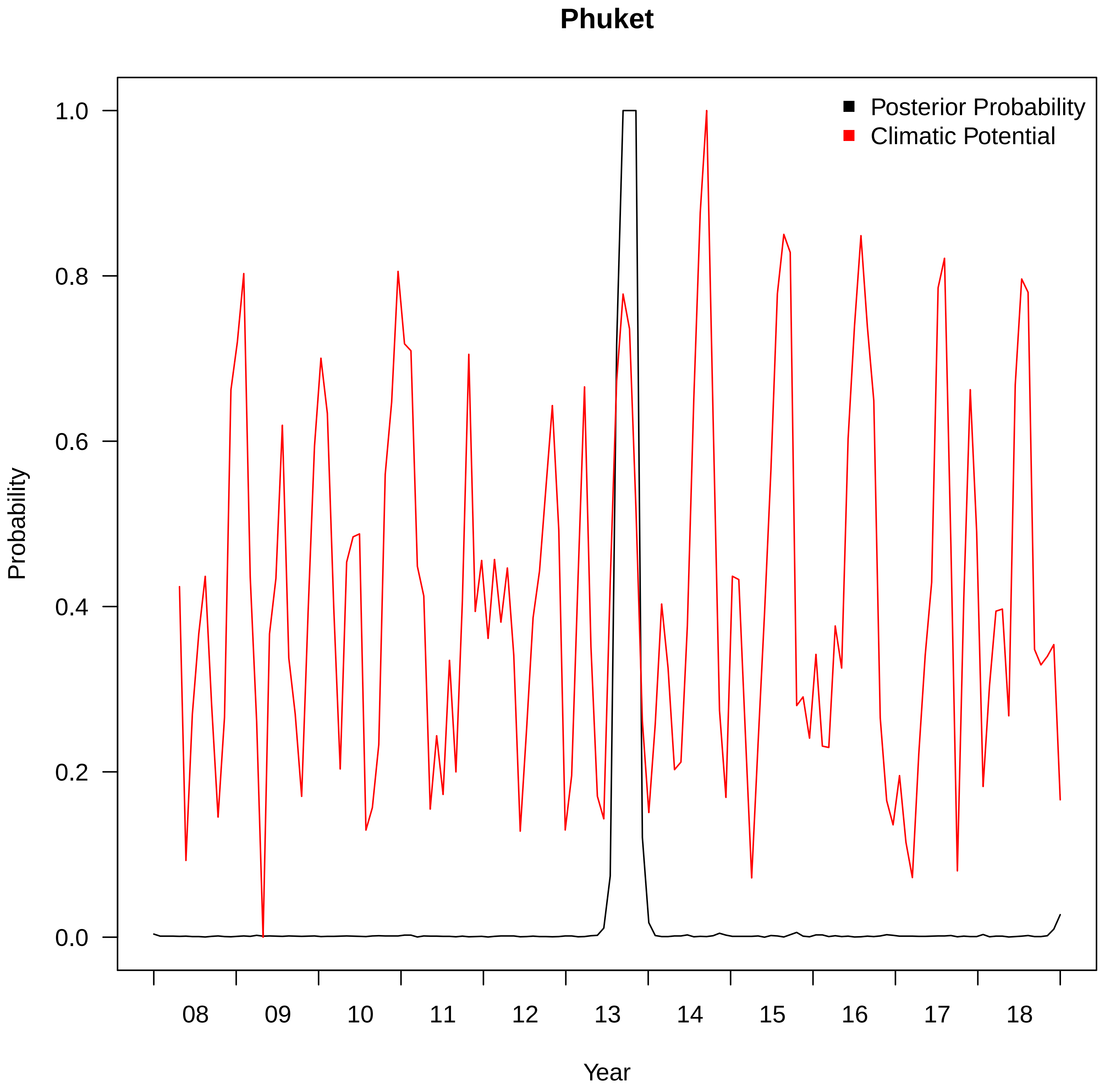
Phuket
Posterior Probability
Climatic Potential
Probability
1.0
0.8
0.6
0.4
0.2
0.0
08
09
10
11
12
13
14
15
16
17
18
Year

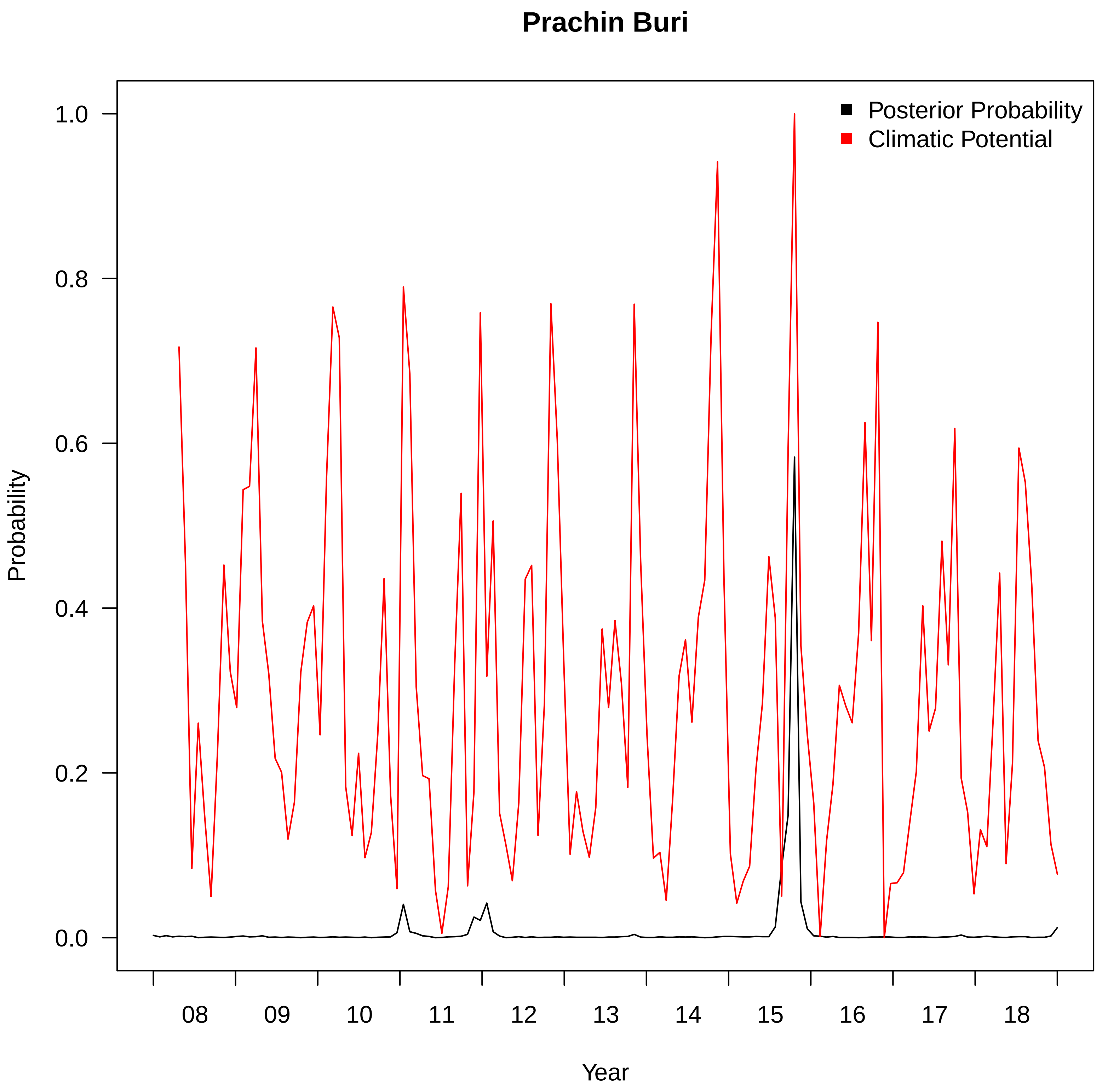

Prachin Buri
Posterior Probability
Climatic Potential
Probability
1.0
0.8
0.6
0.4
0.2
0.0
Year
08
09
10
11
12
13
14
15
16
17
18

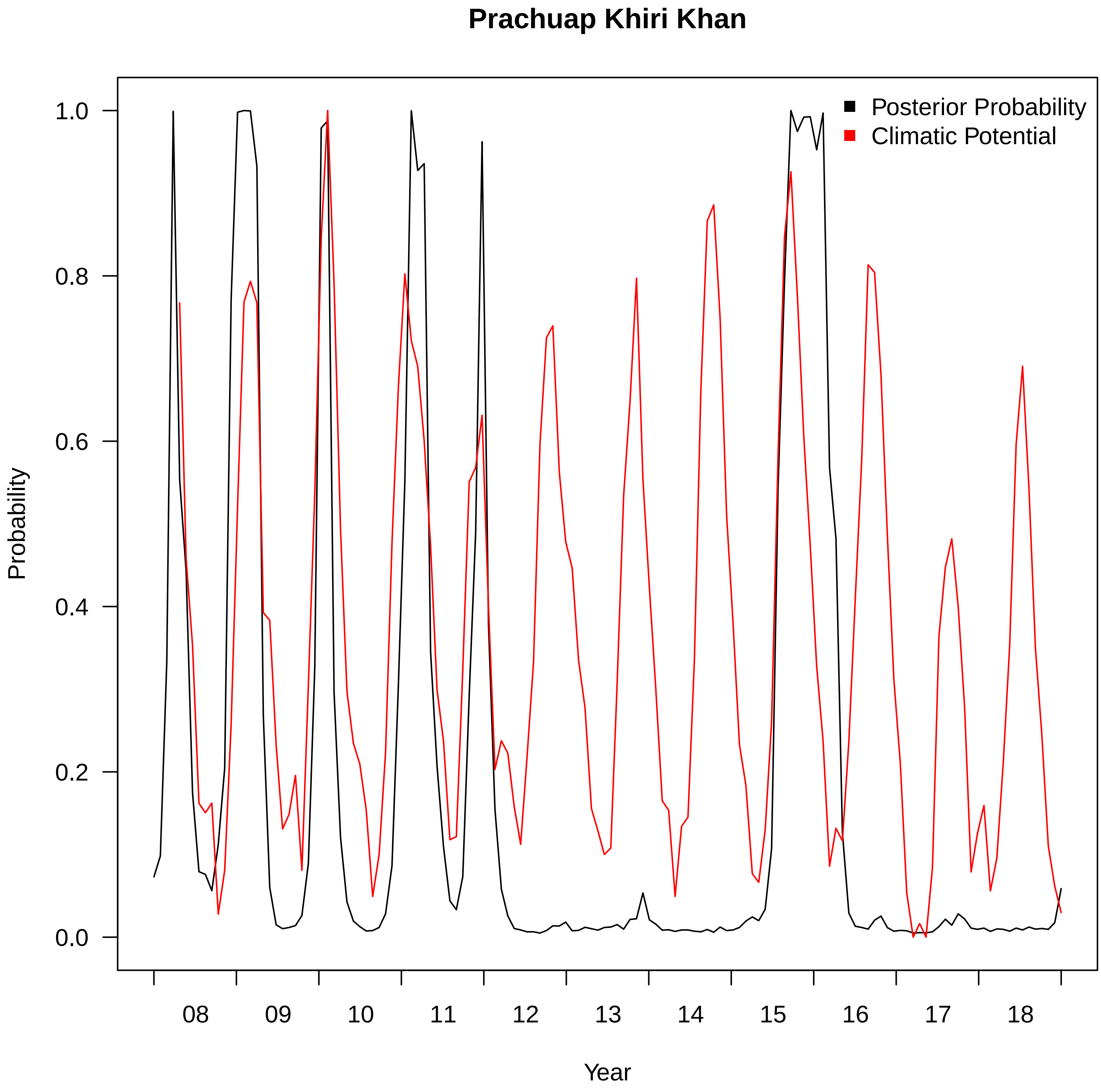

Prachuap Khiri Khan
Posterior Probability
Climatic Potential
Probability
1.0
0.8
0.6
0.4
0.2
0.0
08
09
10
11
12
13
14
15
16
17
18
Year

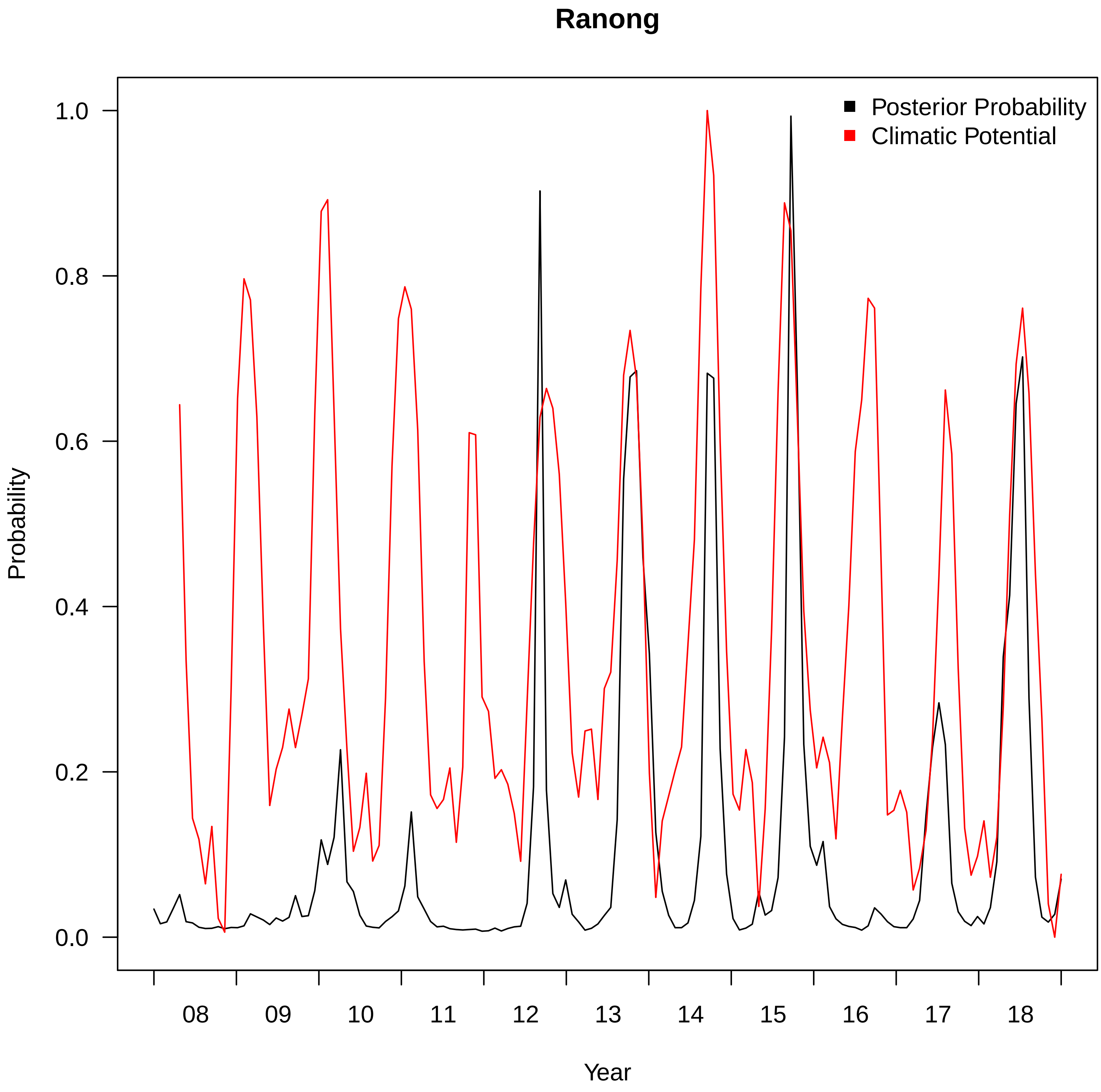
Ranong
Posterior Probability
Climatic Potential
Probability
1.0
0.8
0.6
0.4
0.2
0.0
08
09
10
11
12
13
14
15
16
17
18
Year

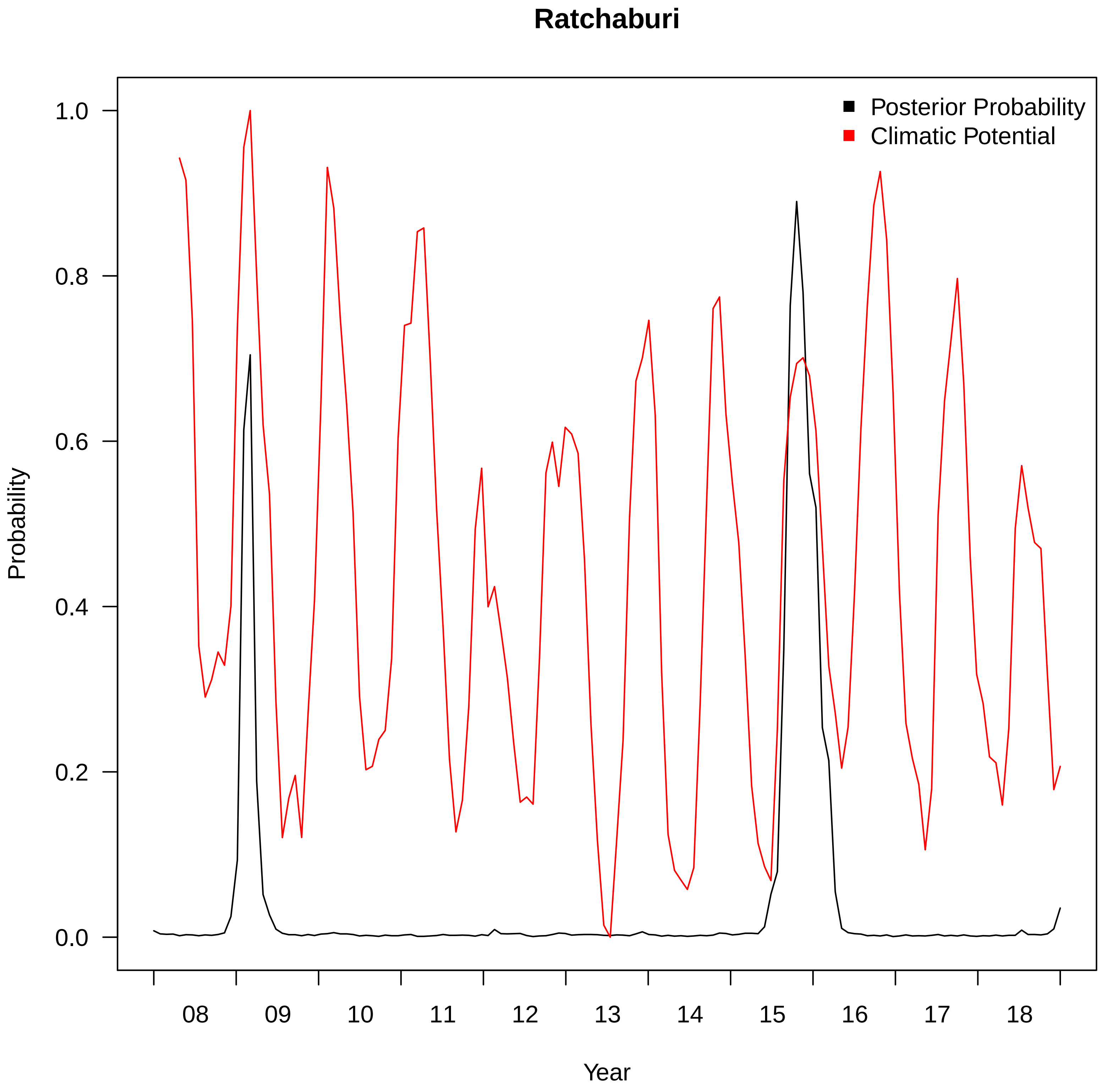

Ratchaburi
Posterior Probability
Climatic Potential
Probability
1.0
0.8
0.6
0.4
0.2
0.0
08
09
10
11
12
13
14
15
16
17
18
Year

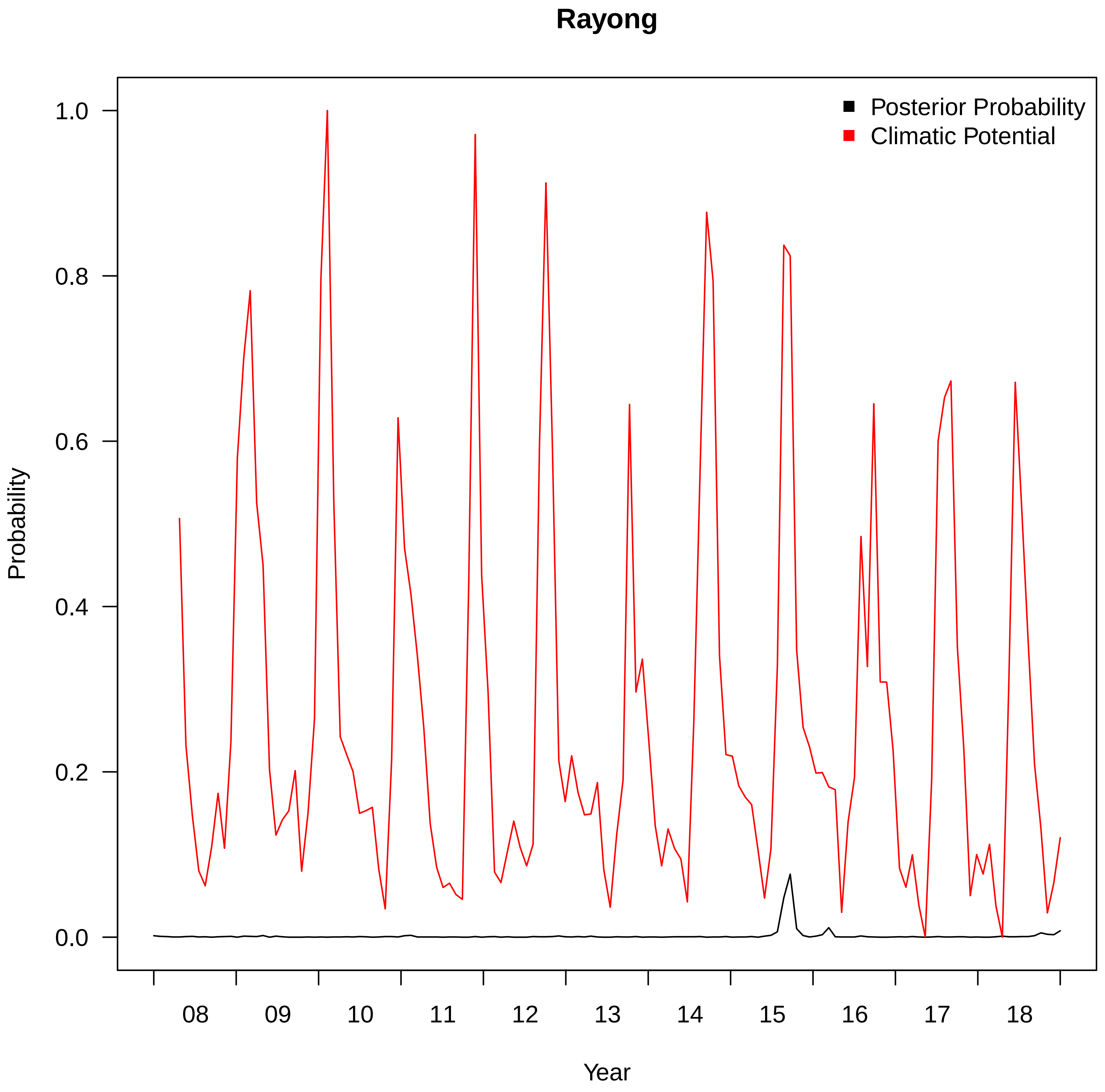
Rayong
Posterior Probability
Climatic Potential
Probability
1.0
0.8
0.6
0.4
0.2
0.0
08
09
10
11
12
13
14
15
16
17
18
Year

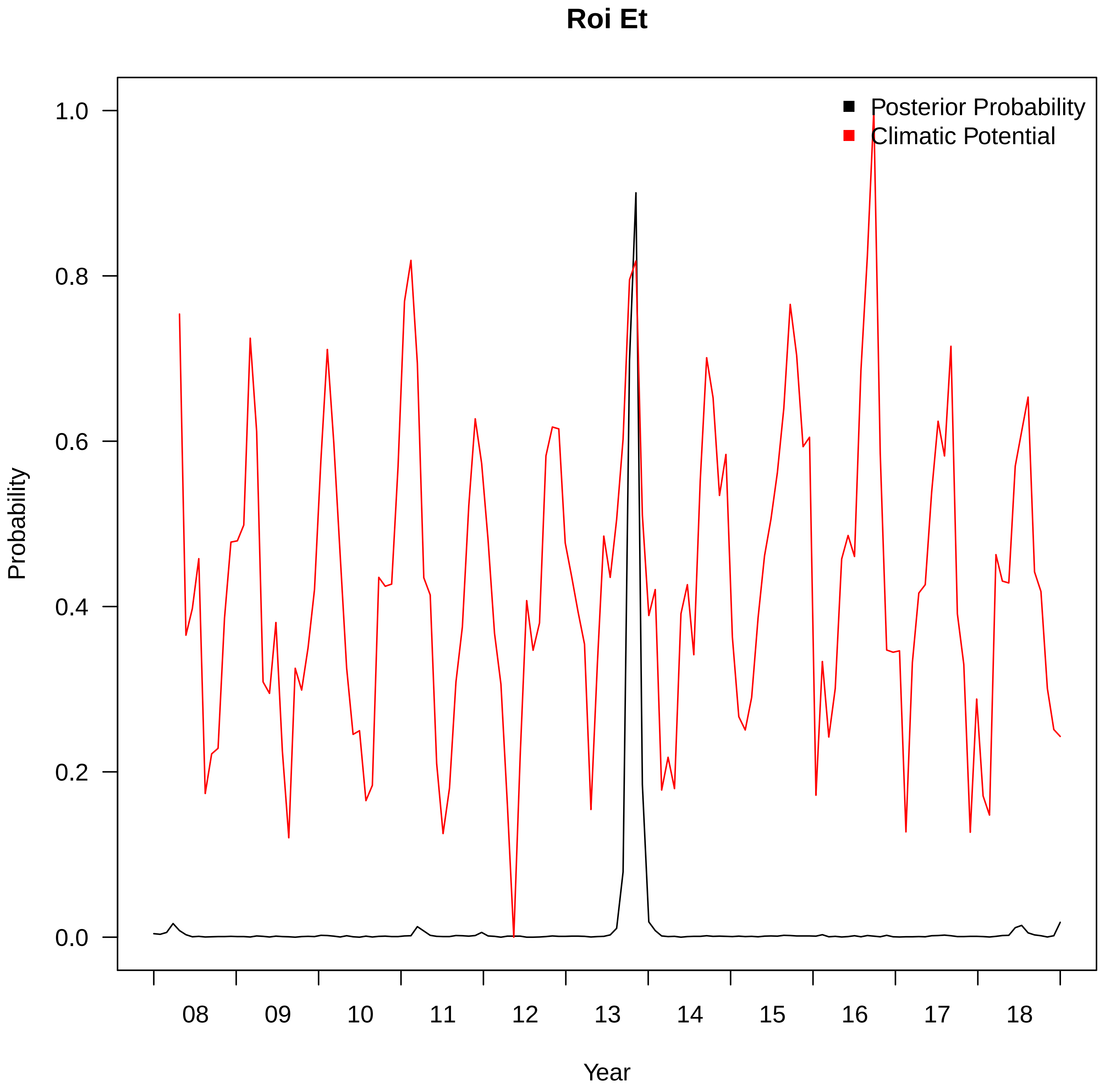
Roi Et
Posterior Probability
Climatic Potential
Probability
1.0
0.8
0.6
0.4
0.2
0.0
08
09
10
11
12
13
14
15
16
17
18
Year

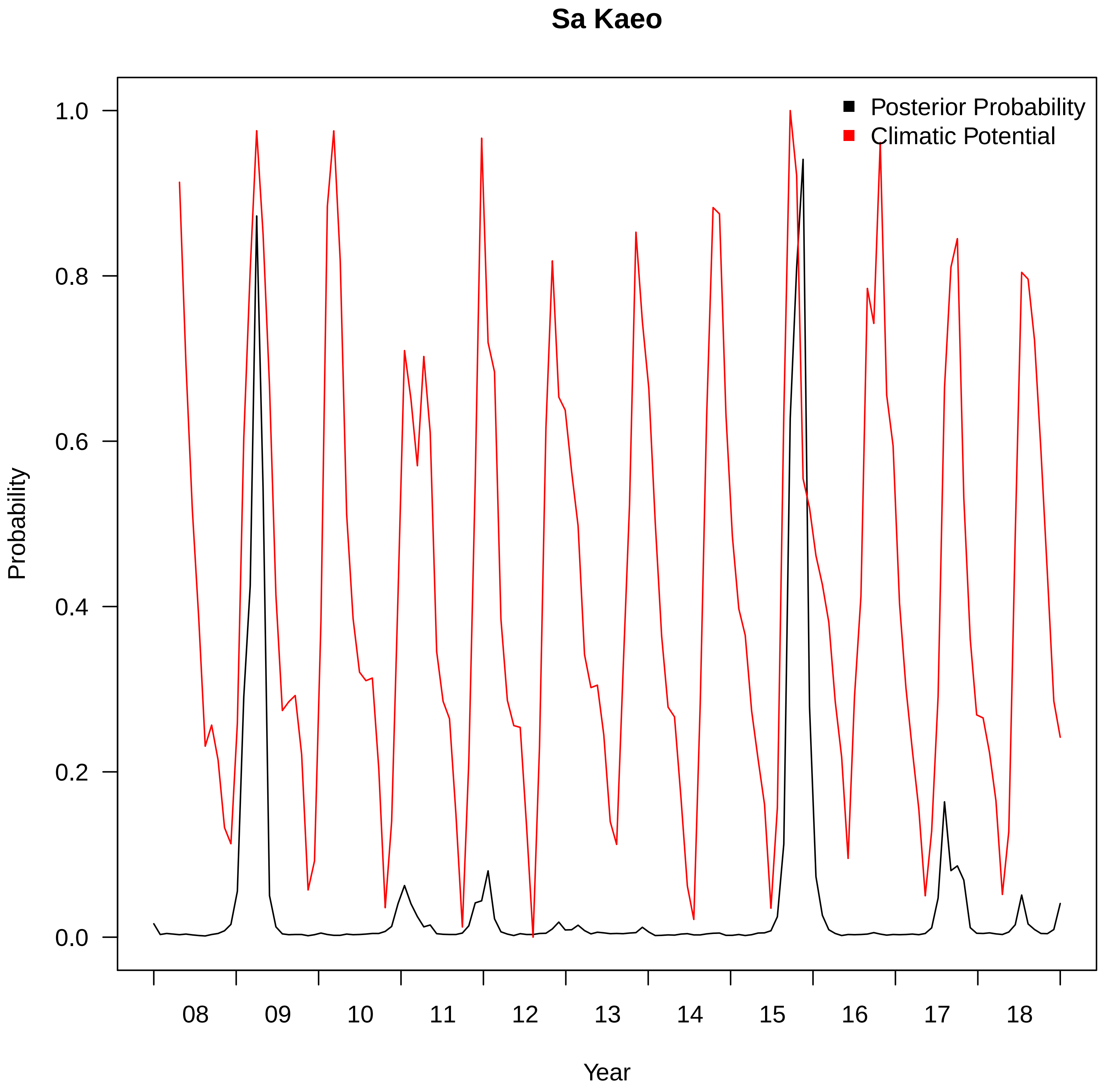
Sa Kaeo
Posterior Probability
Climatic Potential
Probability
Year
1.0
0.8
0.6
0.4
0.2
0.0
08
09
10
11
12
13
14
15
16
17
18

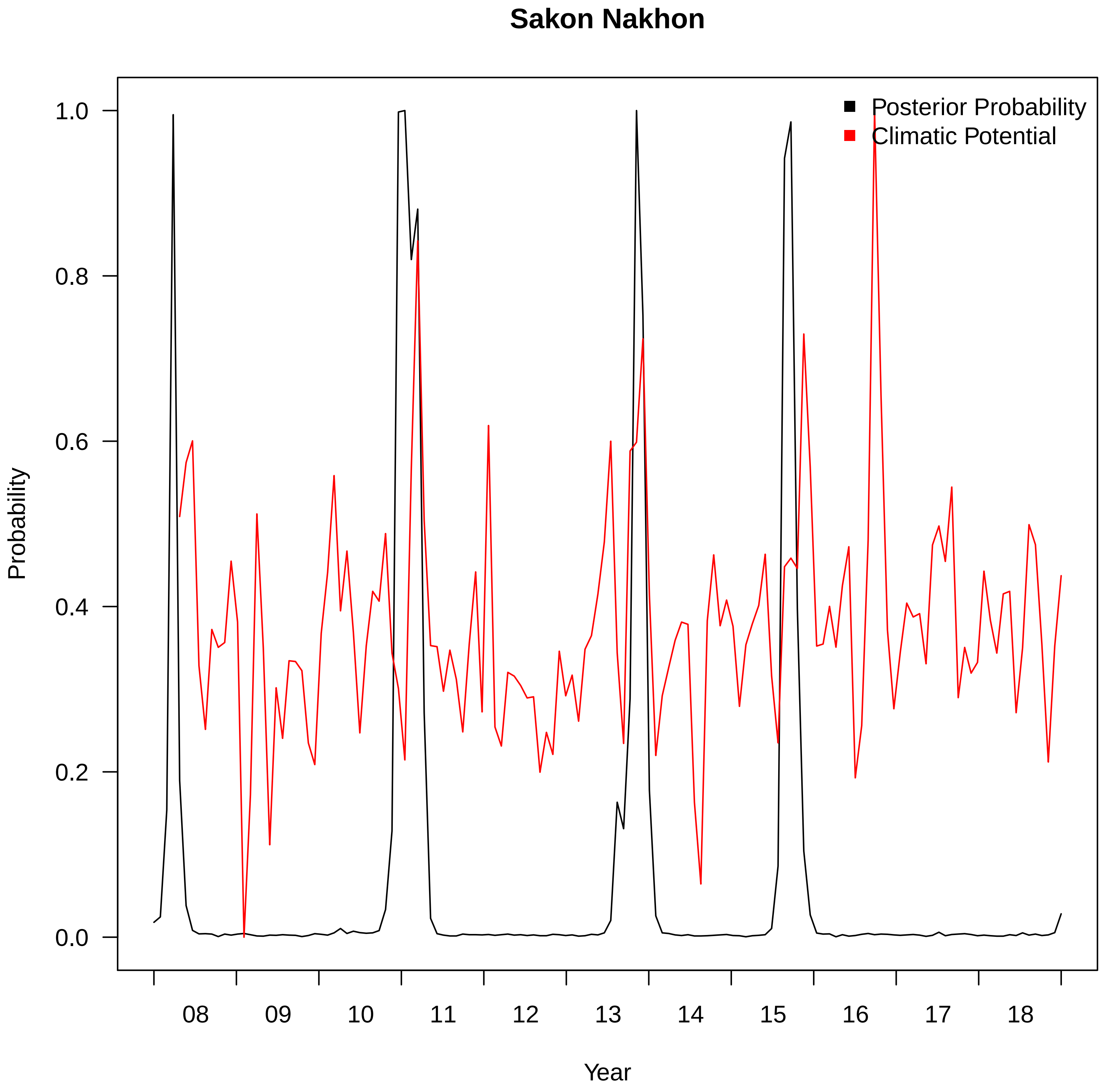

Sakon Nakhon
Posterior Probability
Climatic Potential
Probability
1.0
0.8
0.6
0.4
0.2
0.0
08
09
10
11
12
13
14
15
16
17
18
Year

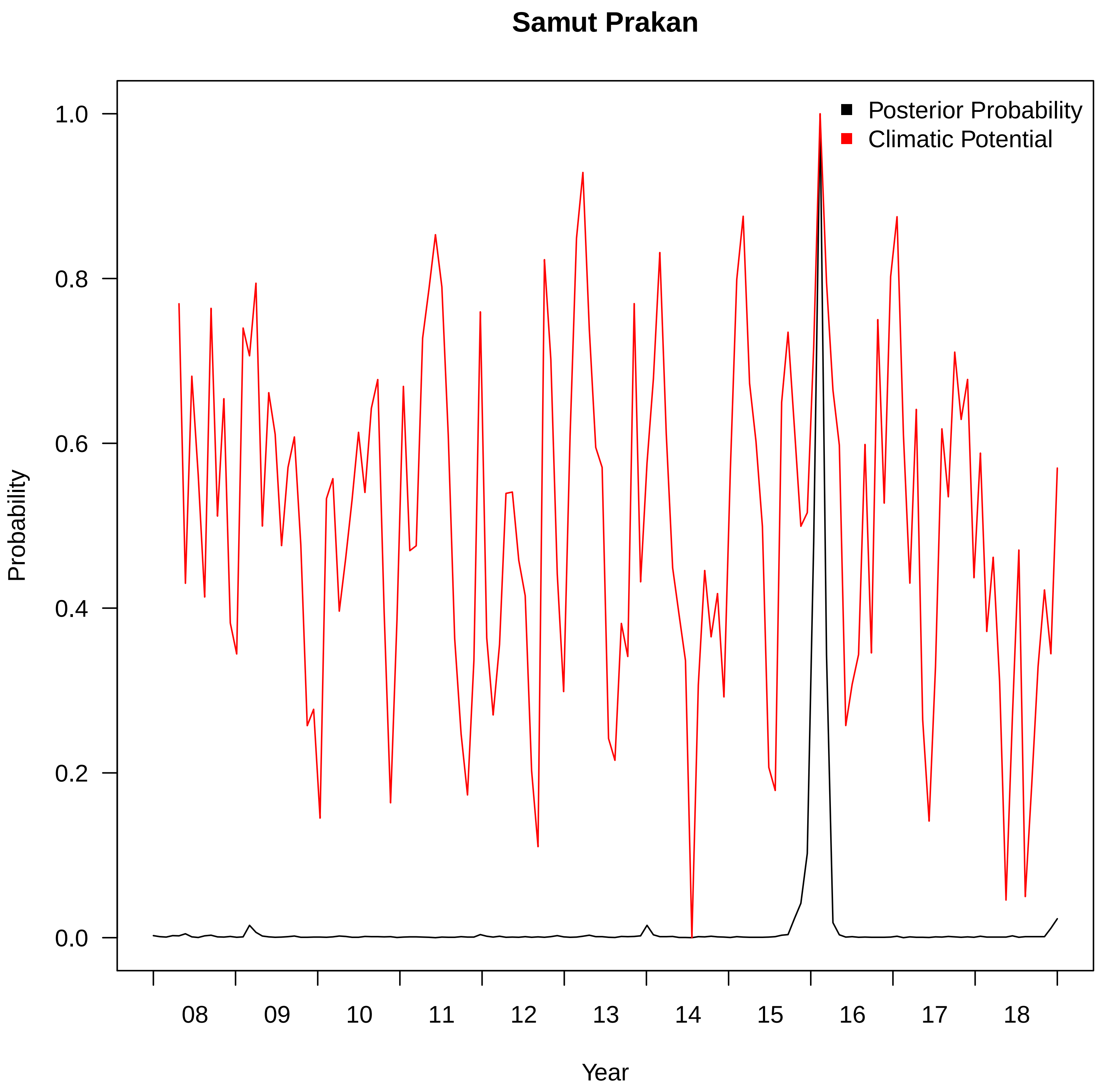
Samut Prakan
Posterior Probability
Climatic Potential
Probability
1.0
0.8
0.6
0.4
0.2
0.0
08
09
10
11
12
13
14
15
16
17
18
Year

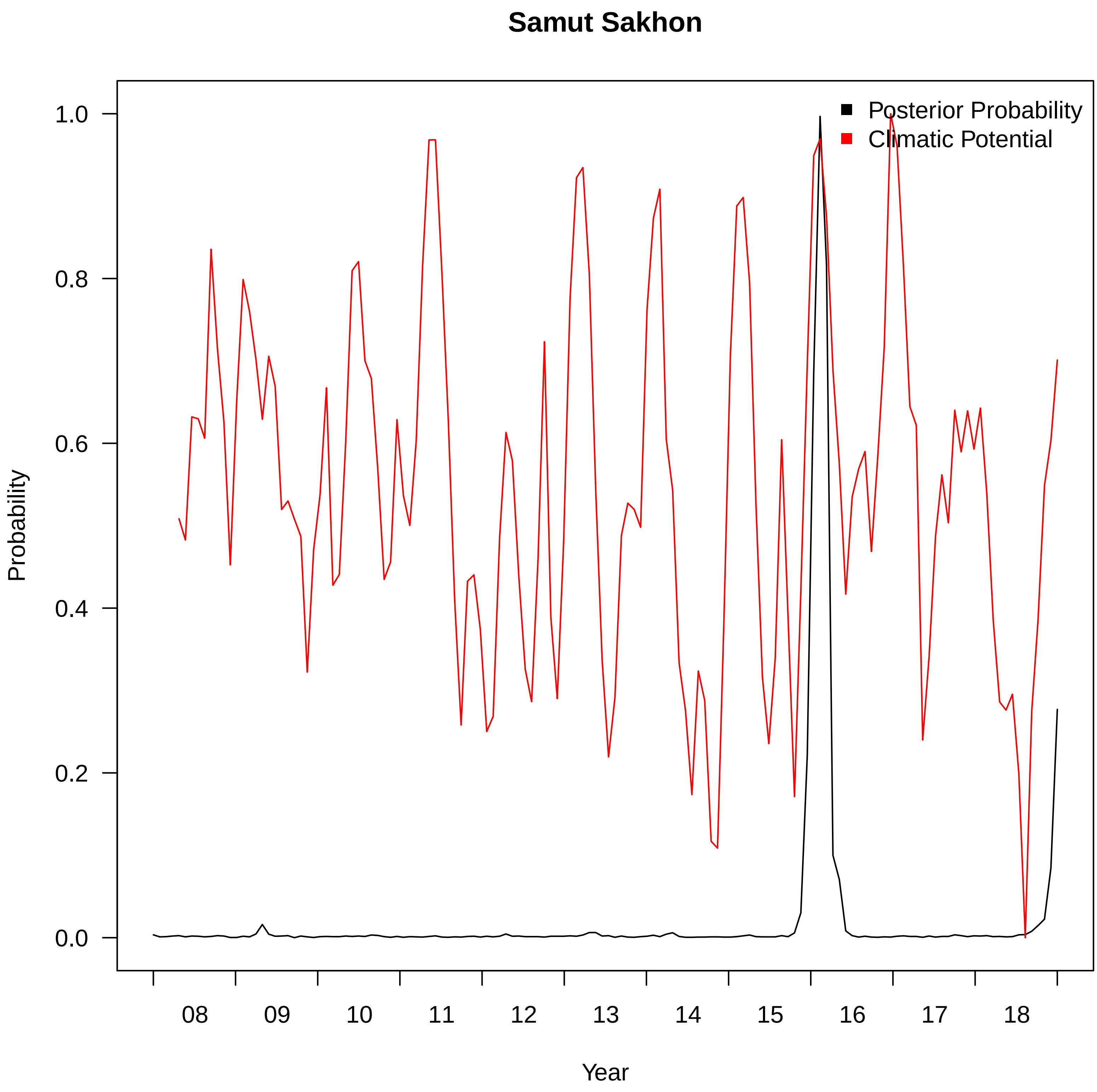

Samut Sakhon
Posterior Probability
Climatic Potential
Probability
1.0
0.8
0.6
0.4
0.2
0.0
08
09
10
11
12
13
14
15
16
17
18
Year

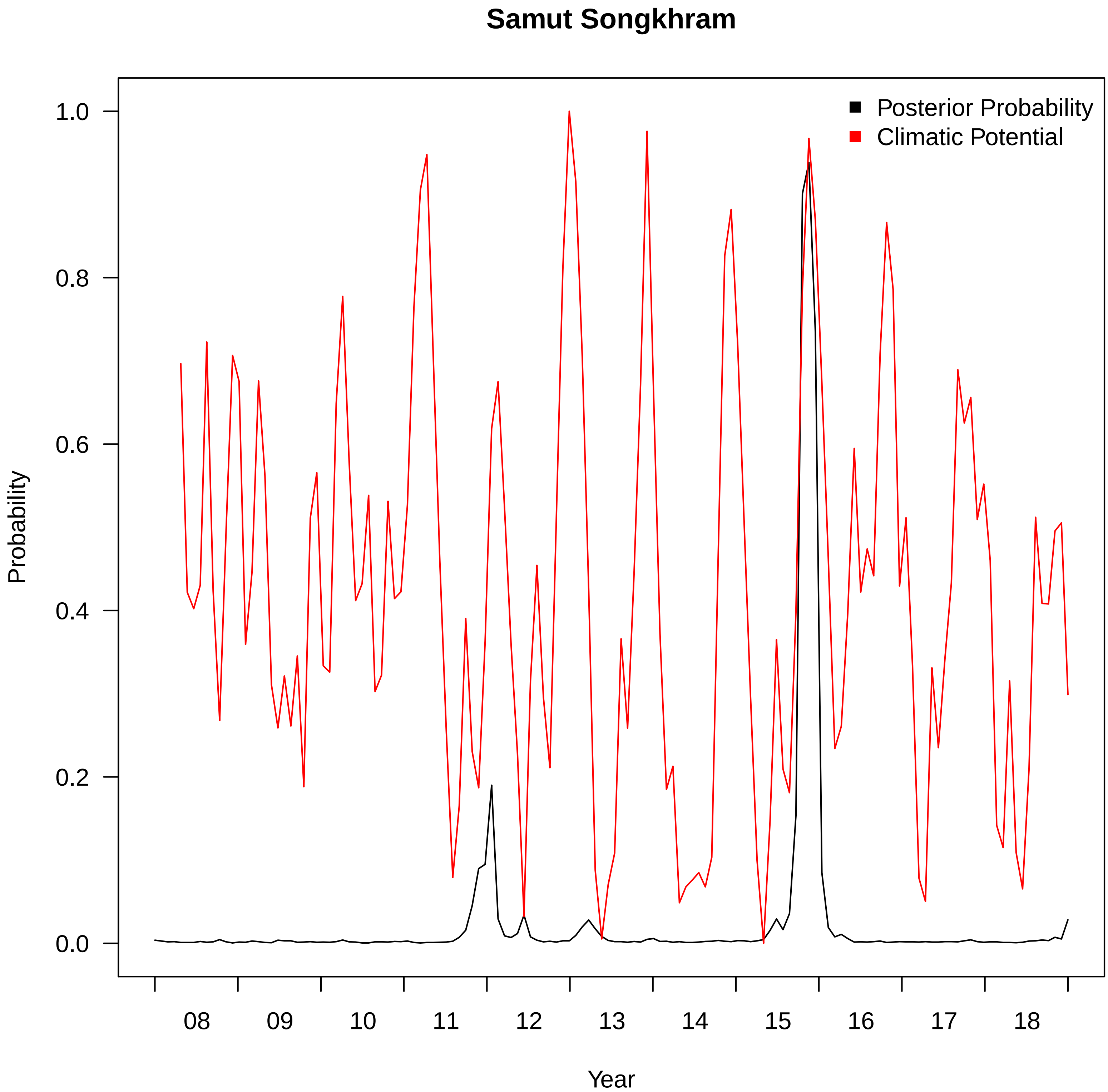

Samut Songkhram
Posterior Probability
Climatic Potential
Probability
1.0
0.8
0.6
0.4
0.2
0.0
08
09
10
11
12
13
14
15
16
17
18
Year

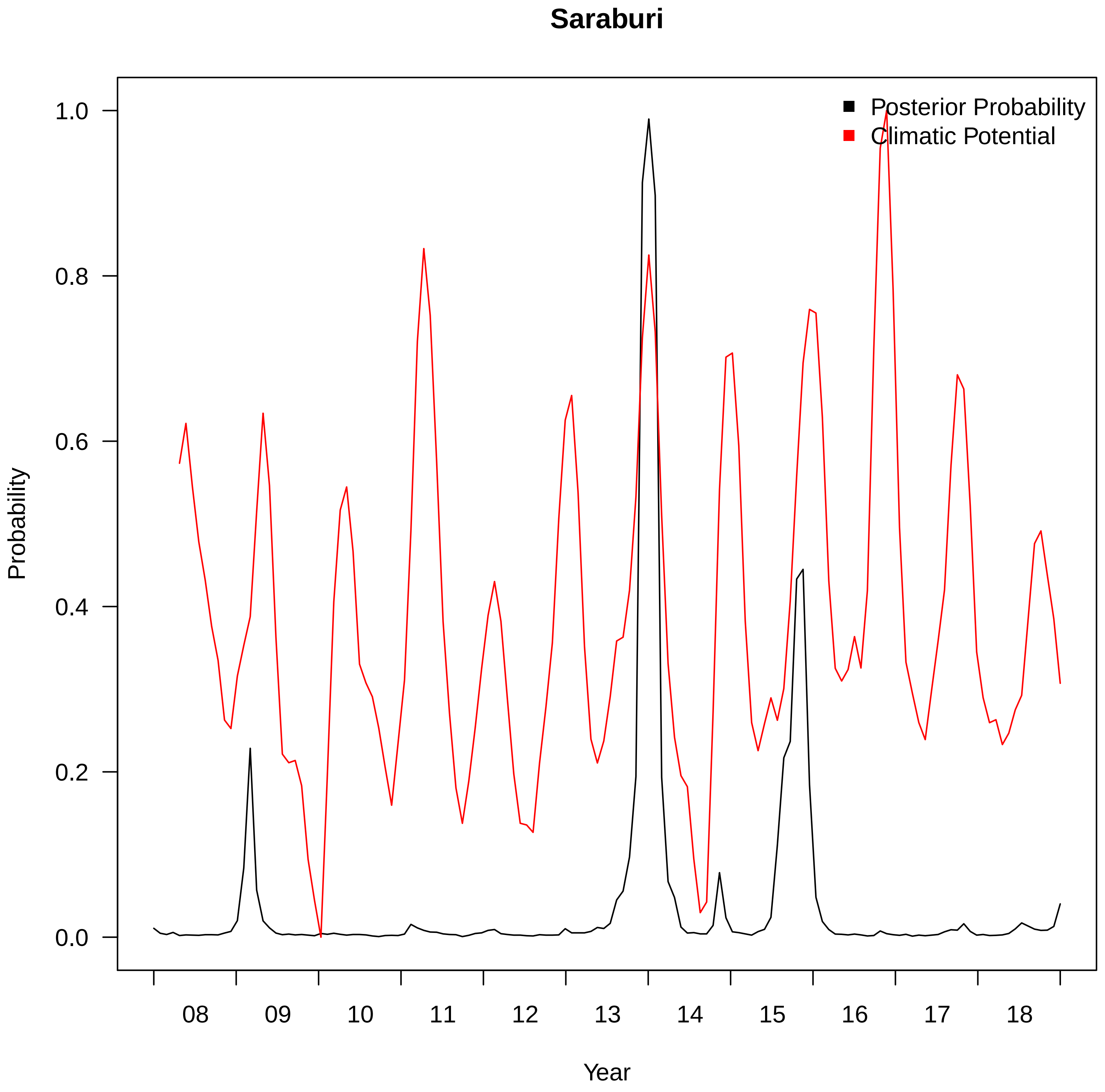
Saraburi
Posterior Probability
Climatic Potential
Probability
1.0
0.8
0.6
0.4
0.2
0.0
08
09
10
11
12
13
14
15
16
17
18
Year

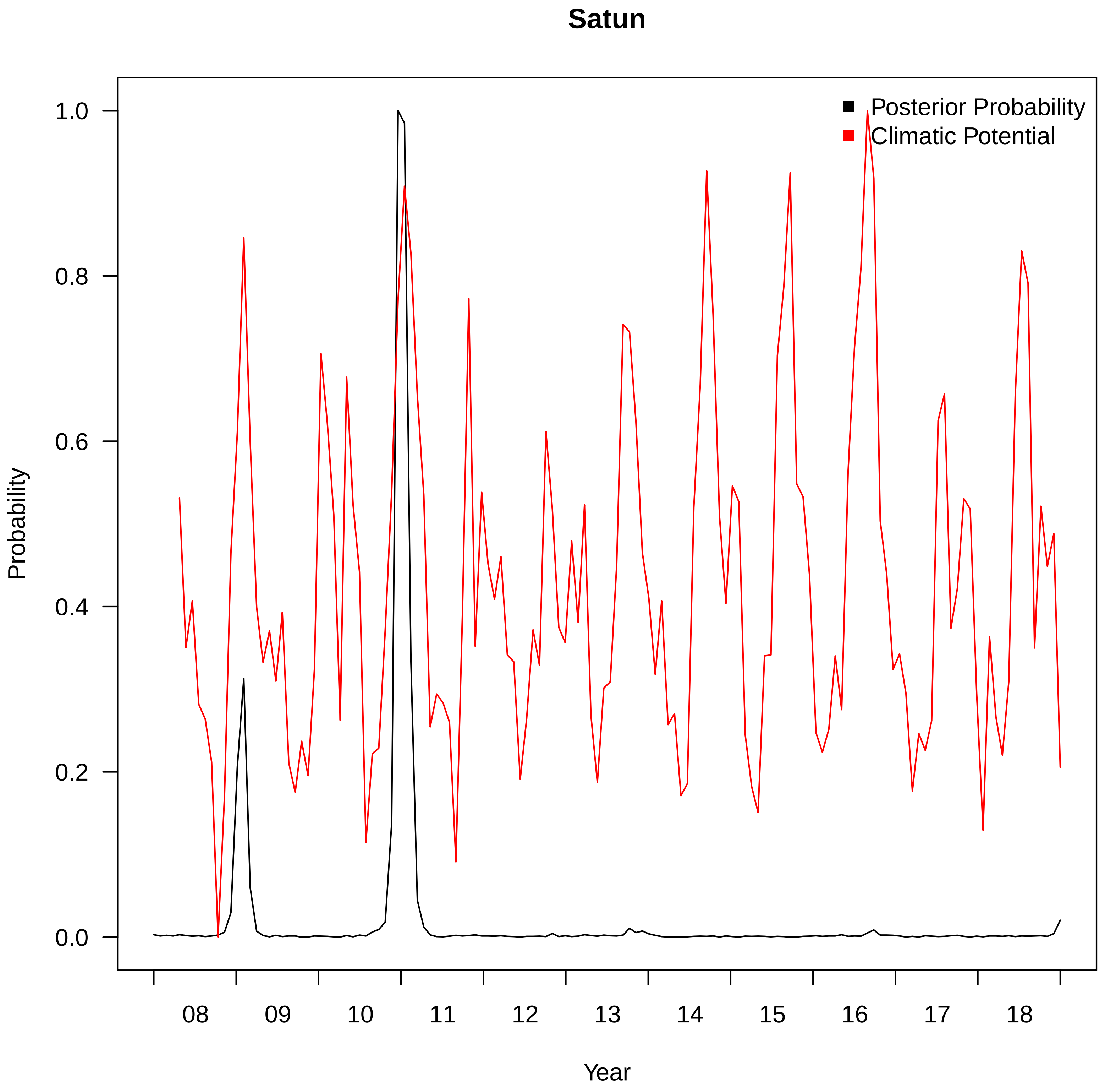
Satun
Posterior Probability
Climatic Potential
Probability
1.0
0.8
0.6
0.4
0.2
0.0
08
09
10
11
12
13
14
15
16
17
18
Year

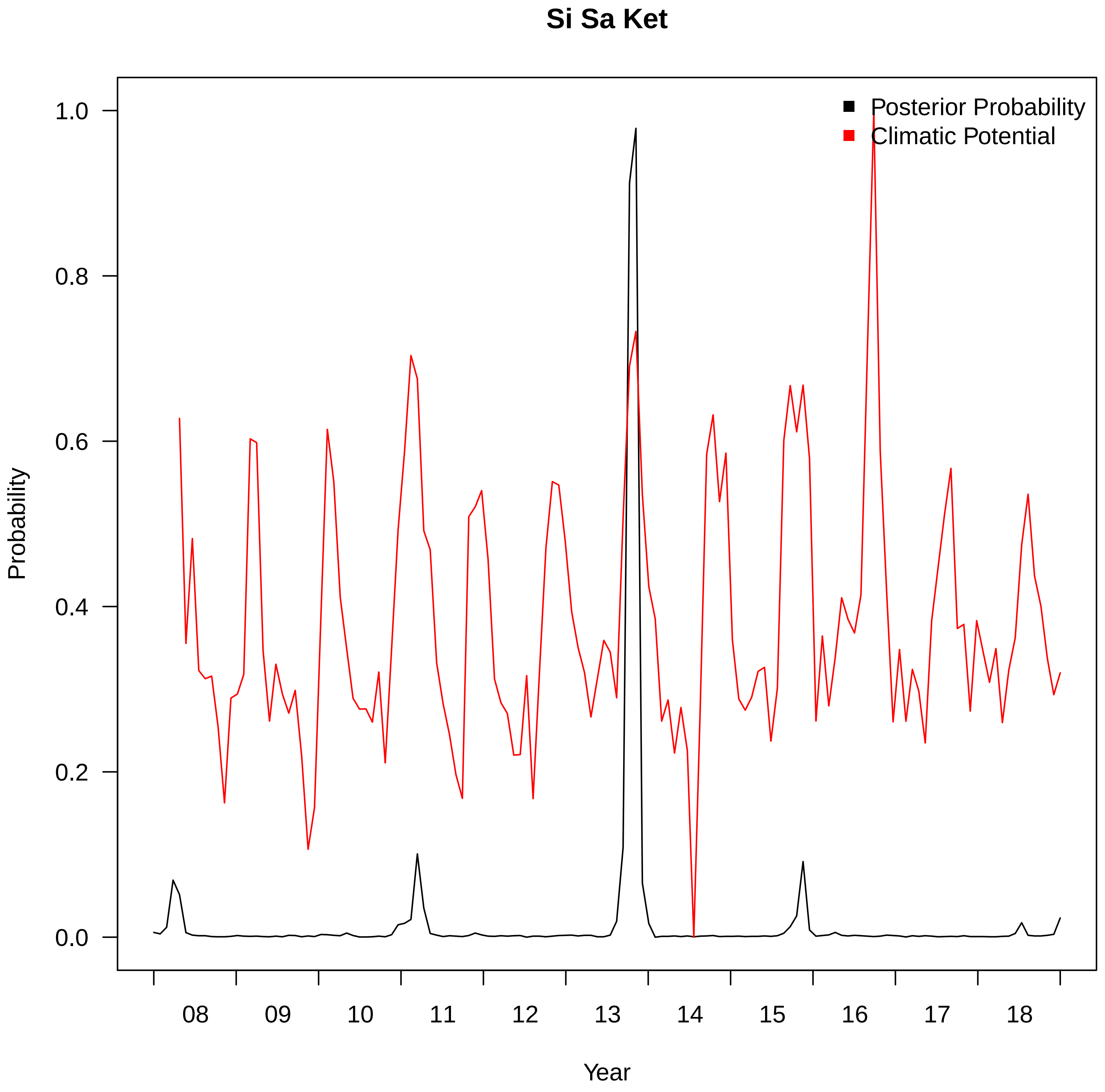
Si Sa Ket
Posterior Probability
Climatic Potential
Probability
1.0
0.8
0.6
0.4
0.2
0.0
08
09
10
11
12
13
14
15
16
17
18
Year

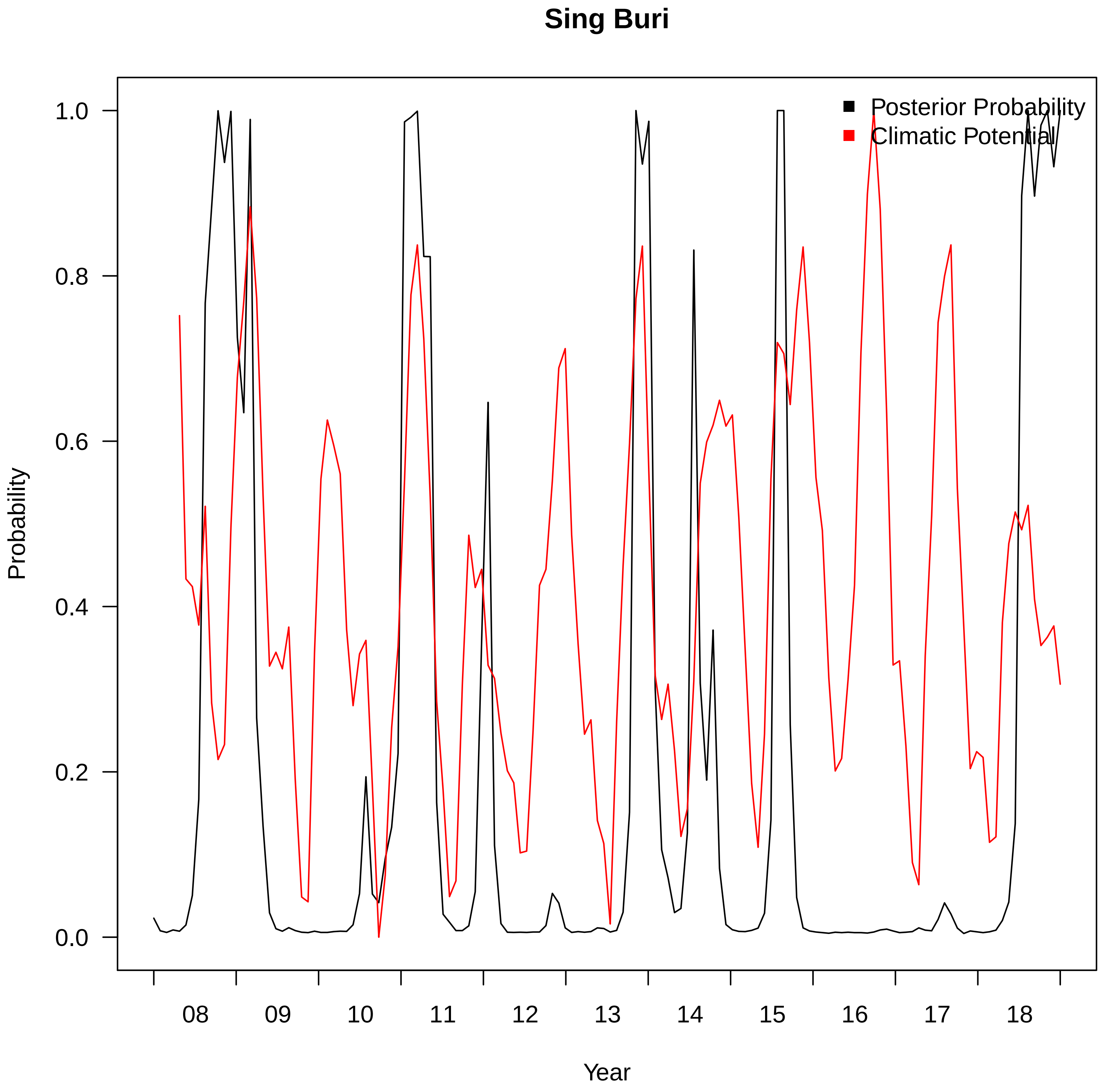
Sing Buri
Posterior Probability
Climatic Potential
Probability
1.0
0.8
0.6
0.4
0.2
0.0
08
09
10
11
12
13
14
15
16
17
18
Year

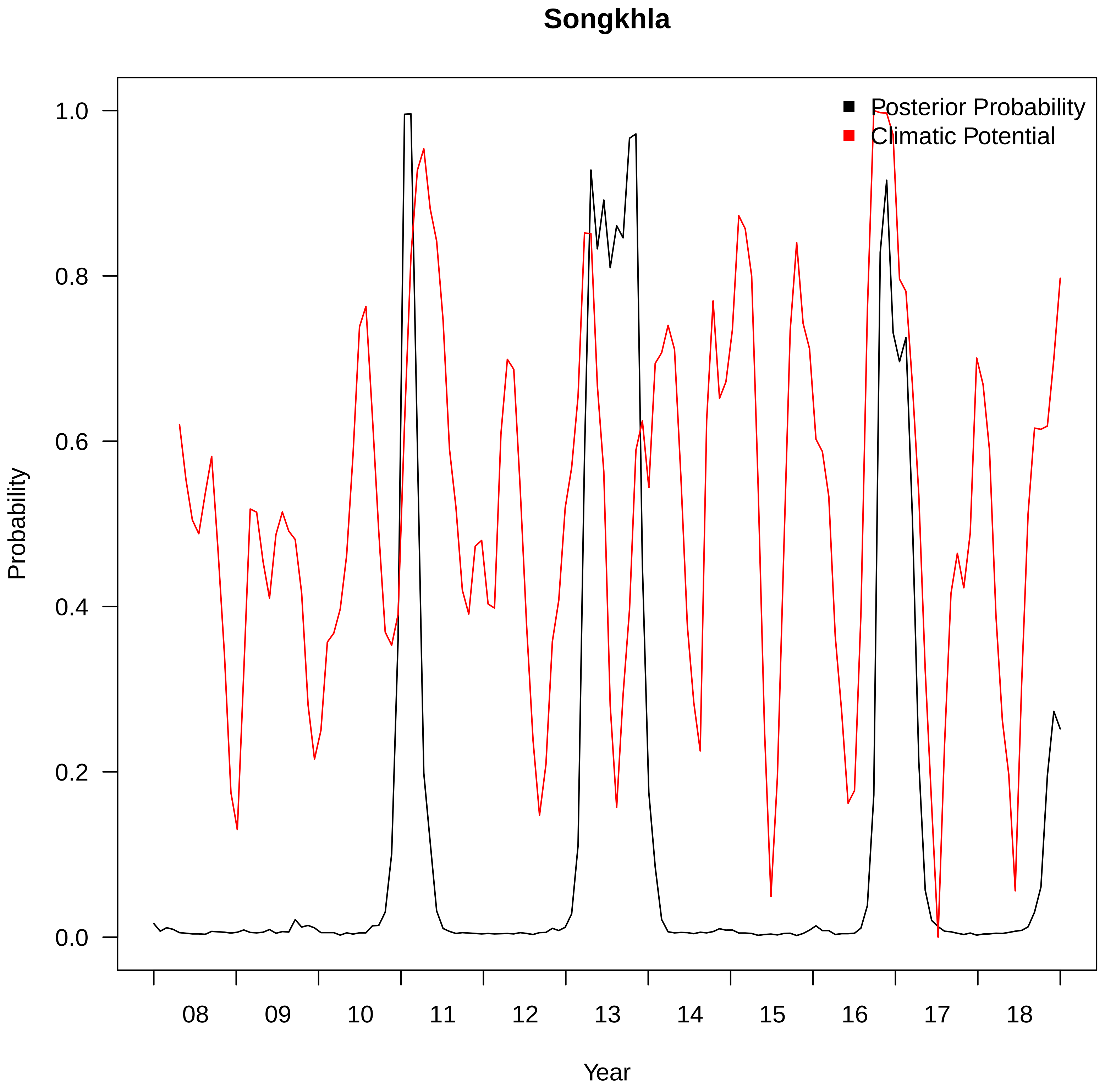

Songkhla
Posterior Probability
Climatic Potential
1.0
0.8
0.6
0.4
0.2
0.0
Probability
08
09
10
11
12
13
14
15
16
17
18
Year

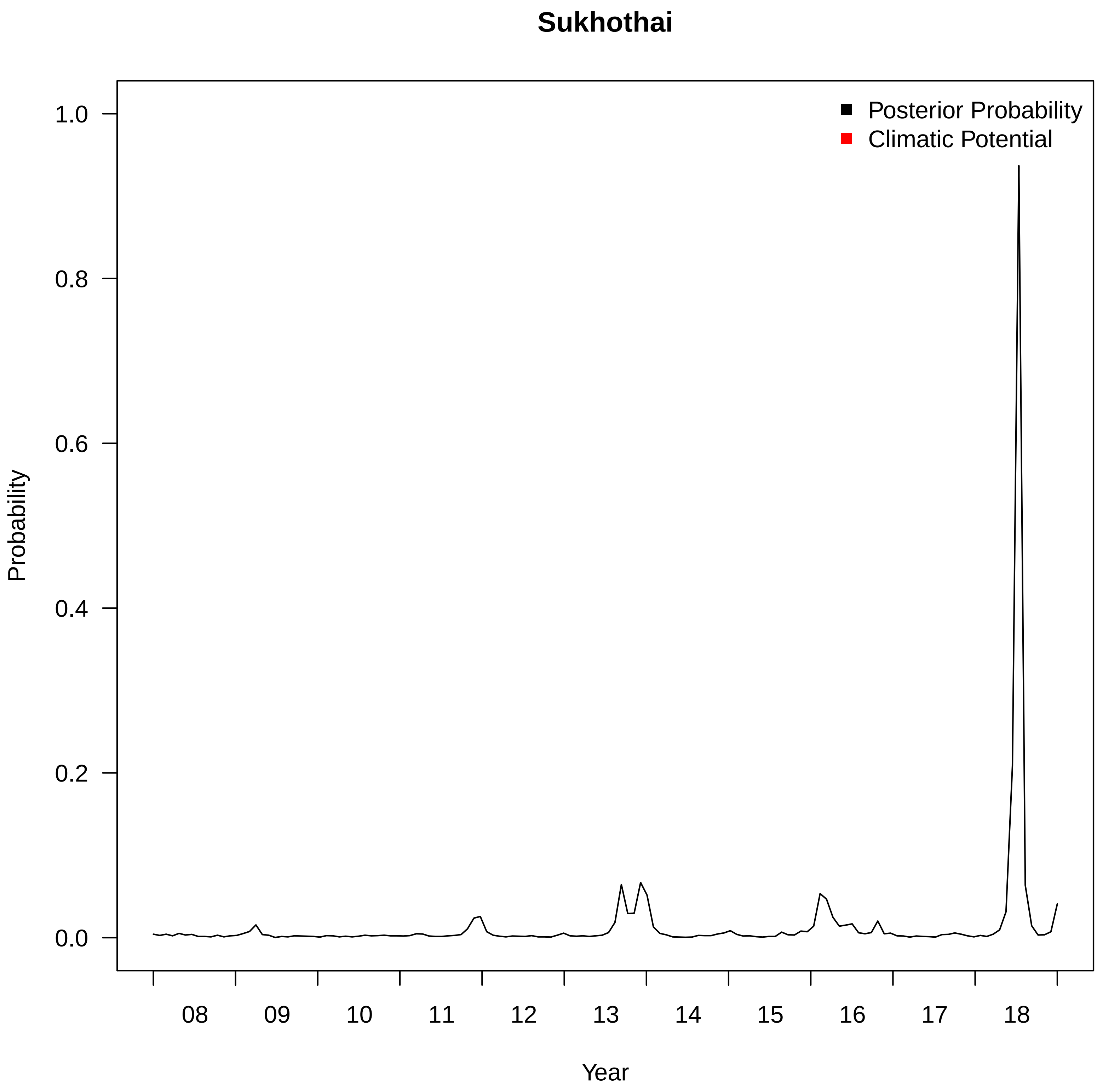
Sukhothai
Posterior Probability
Climatic Potential
1.0
0.8
0.6
0.4
0.2
0.0
Probability
08
09
10
11
12
13
14
15
16
17
18
Year

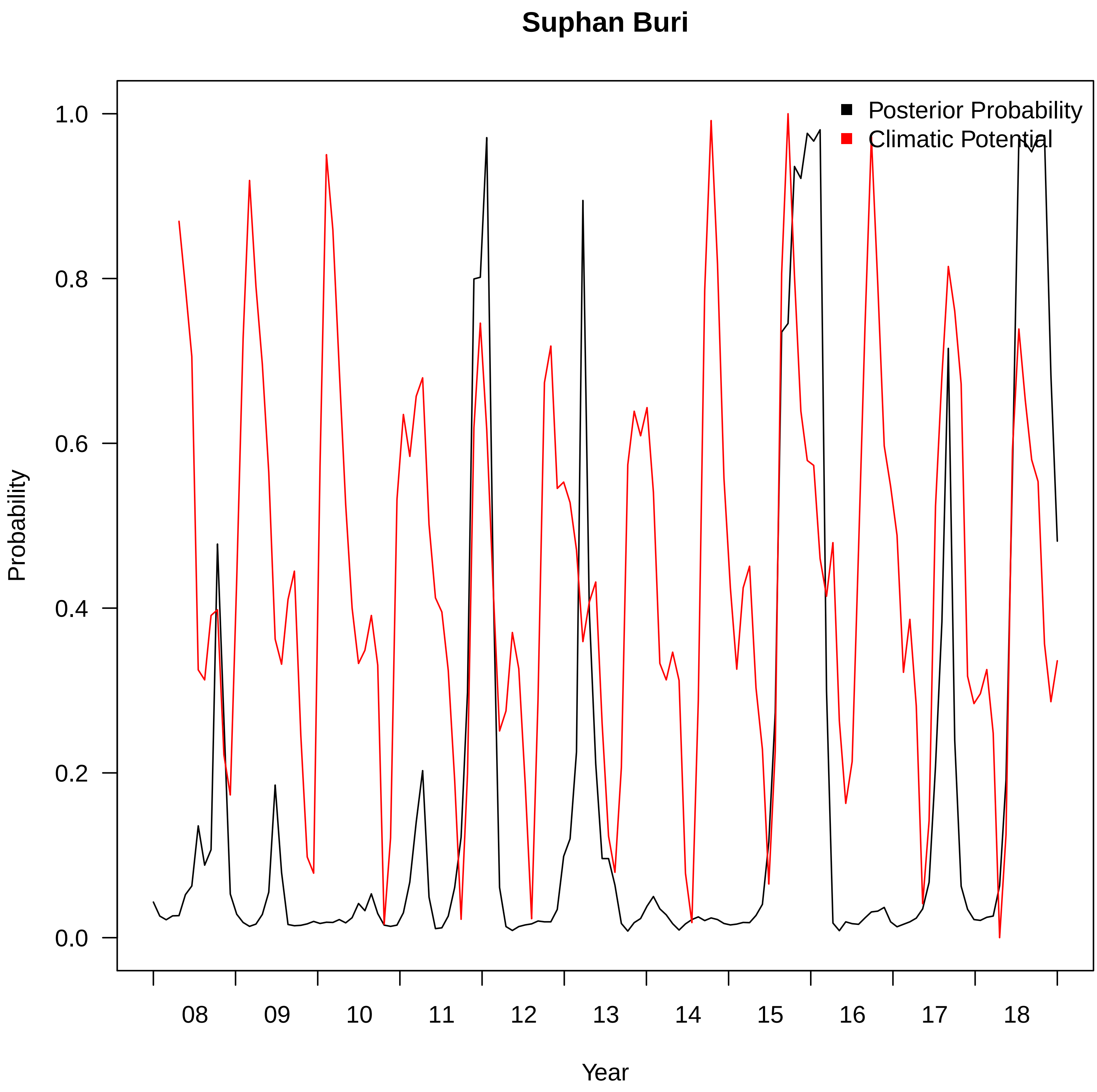
Suphan Buri
Posterior Probability
Climatic Potential
Probability
1.0
0.8
0.6
0.4
0.2
0.0
Year
08
09
10
11
12
13
14
15
16
17
18

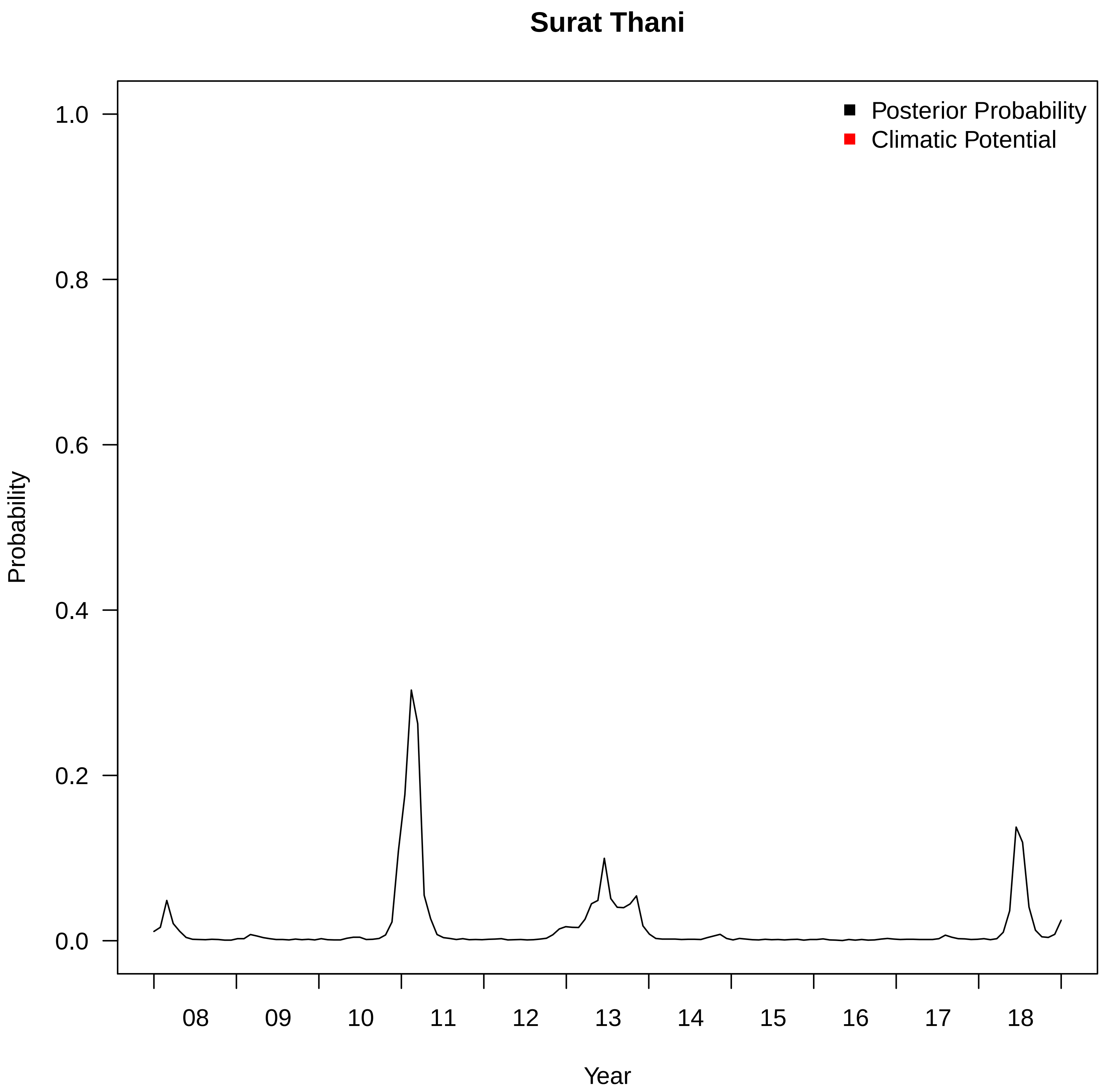

Surat Thani
Posterior Probability
Climatic Potential
Probability
1.0
0.8
0.6
0.4
0.2
0.0
08
09
10
11
12
13
14
15
16
17
18
Year

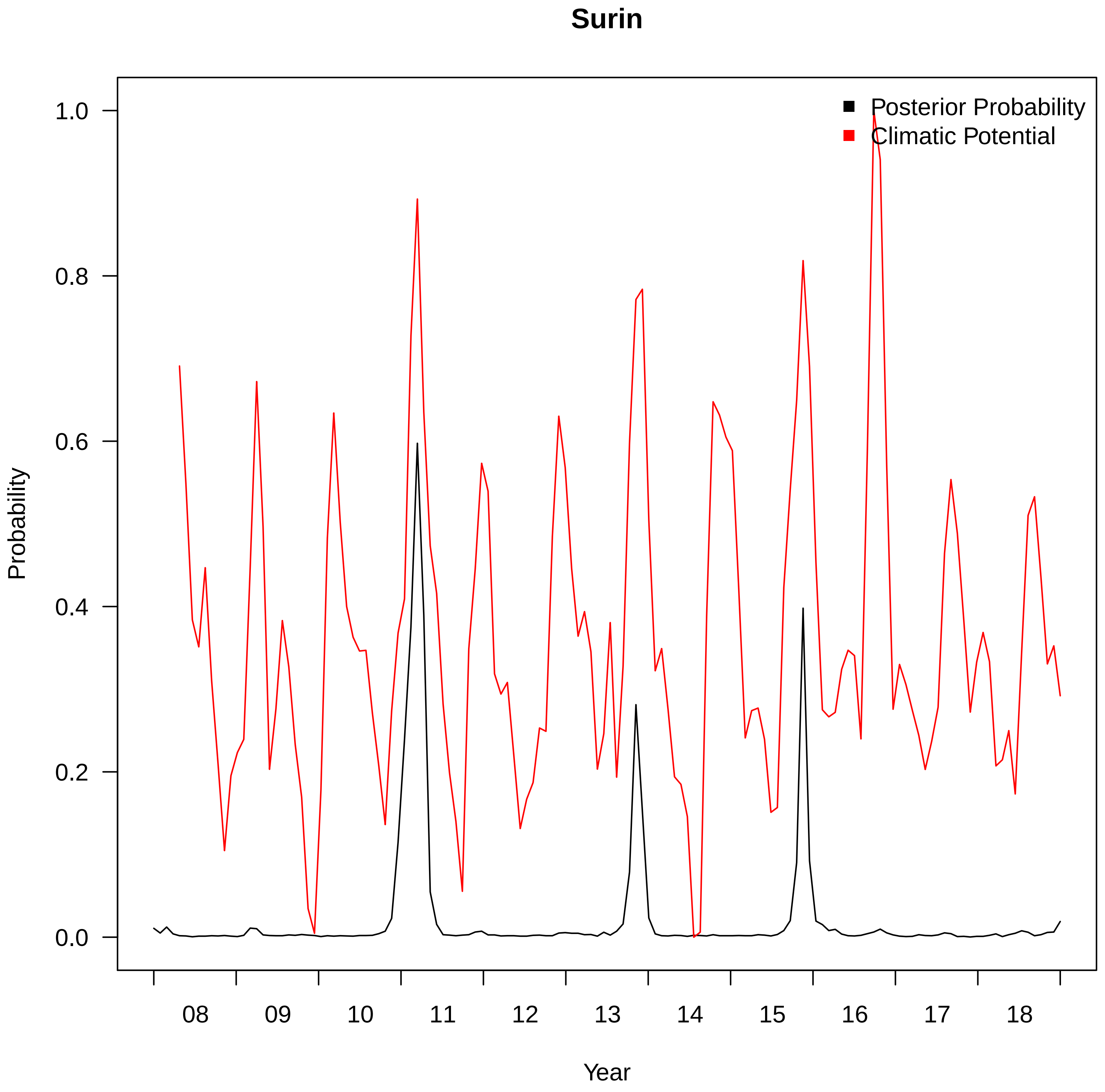
Surin
Posterior Probability
Climatic Potential
Probability
1.0
0.8
0.6
0.4
0.2
0.0
08
09
10
11
12
13
14
15
16
17
18
Year

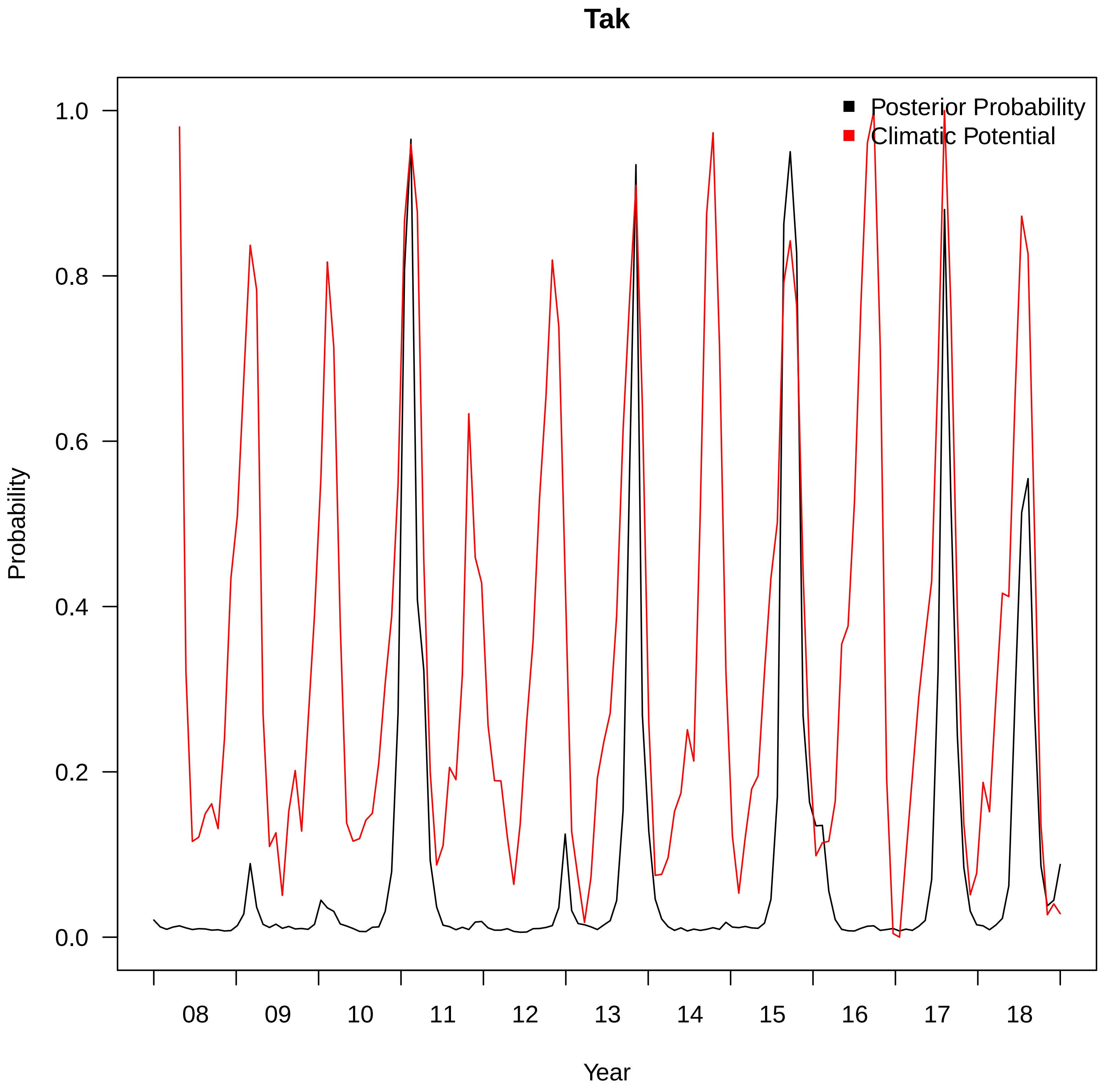

Tak
Posterior Probability
Climatic Potential
Probability
Year
0.0
0.2
0.4
0.6
0.8
1.0
08
09
10
11
12
13
14
15
16
17
18

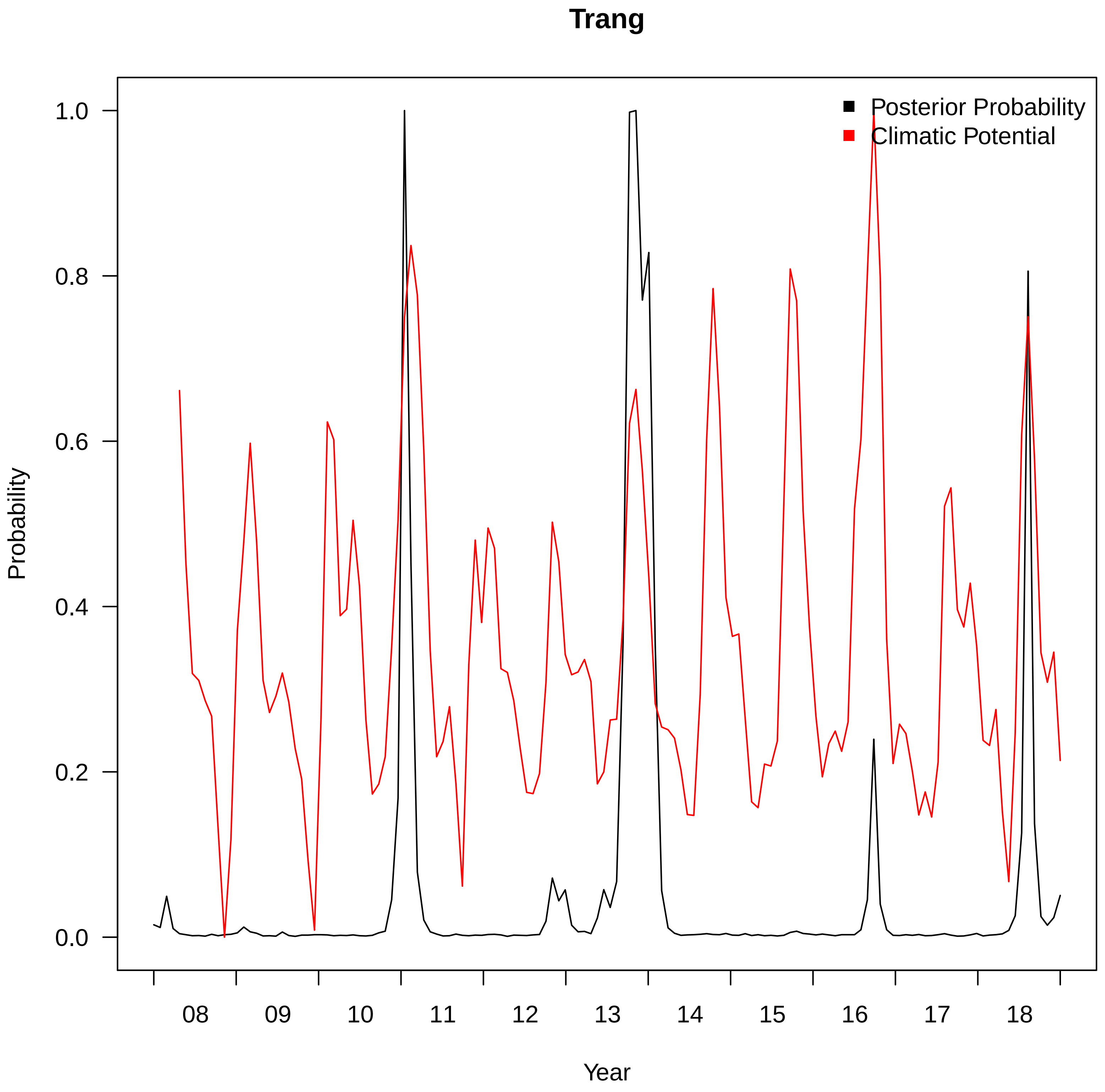
Trang
Posterior Probability
Climatic Potential
Probability
1.0
0.8
0.6
0.4
0.2
0.0
08
09
10
11
12
13
14
15
16
17
18
Year

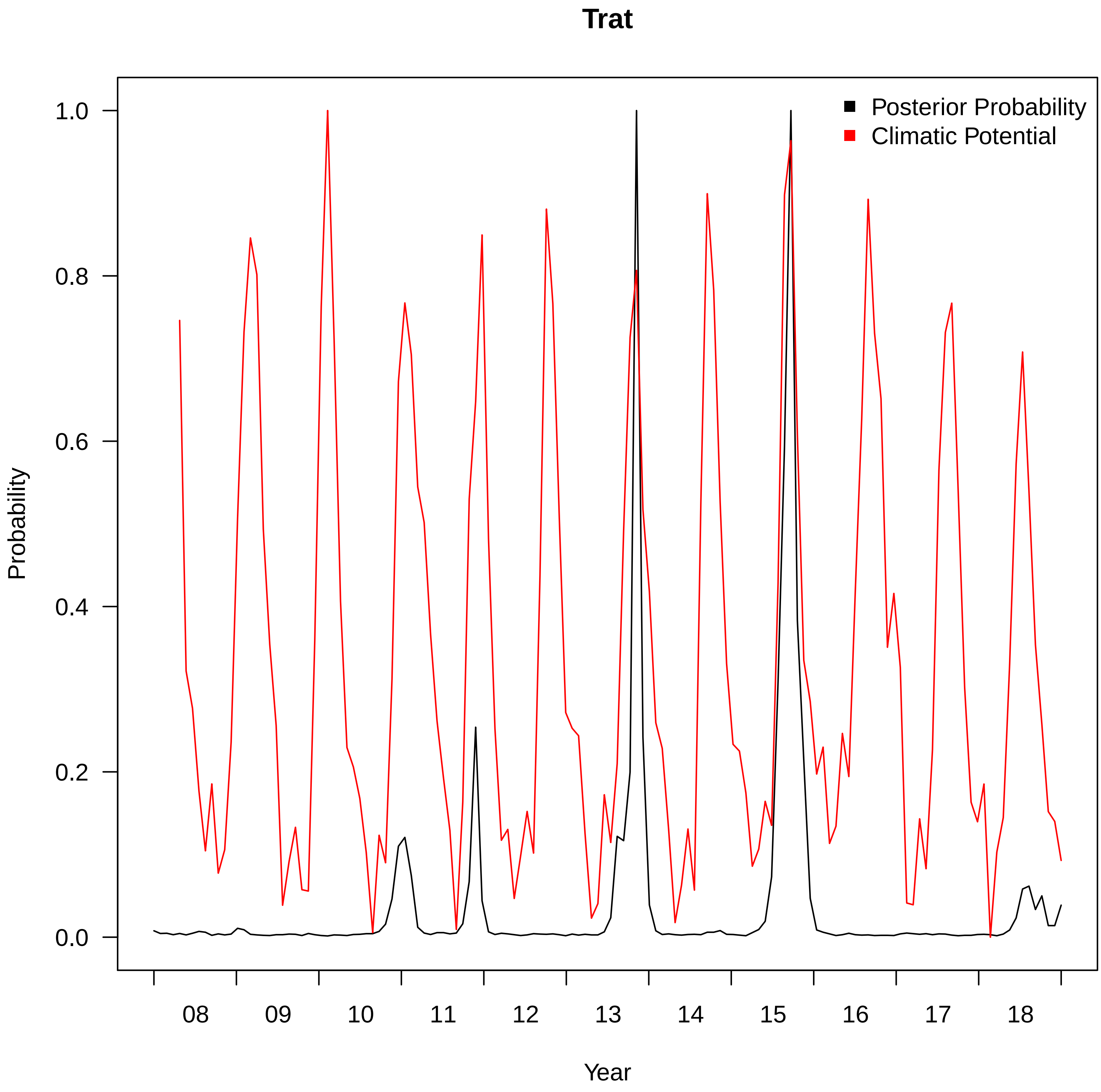

Trat
Posterior Probability
Climatic Potential
Probability
1.0
0.8
0.6
0.4
0.2
0.0
08
09
10
11
12
13
14
15
16
17
18
Year

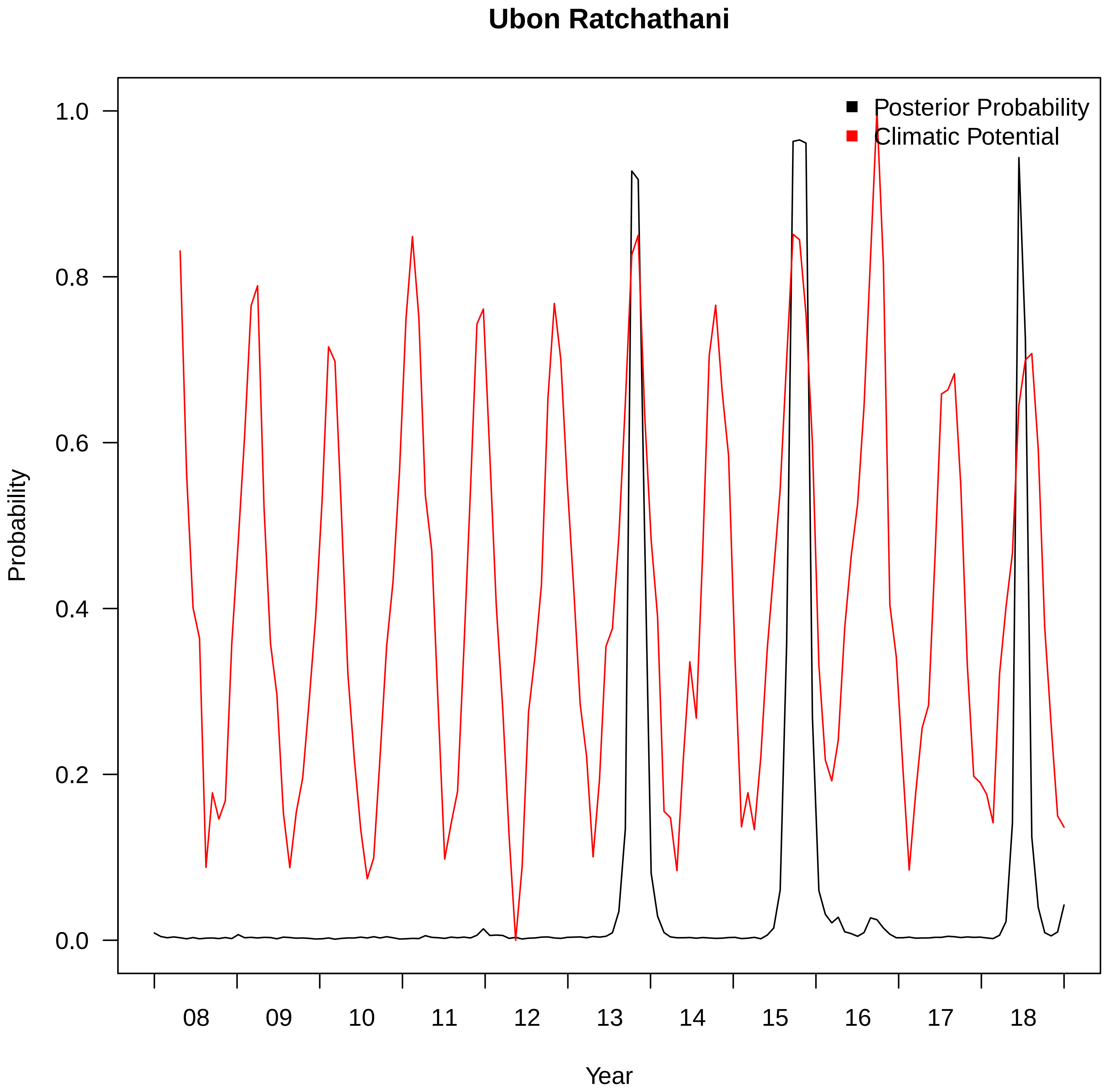
Ubon Ratchathani
Posterior Probability
Climatic Potential
Probability
1.0
0.8
0.6
0.4
0.2
0.0
08
09
10
11
12
13
14
15
16
17
18
Year

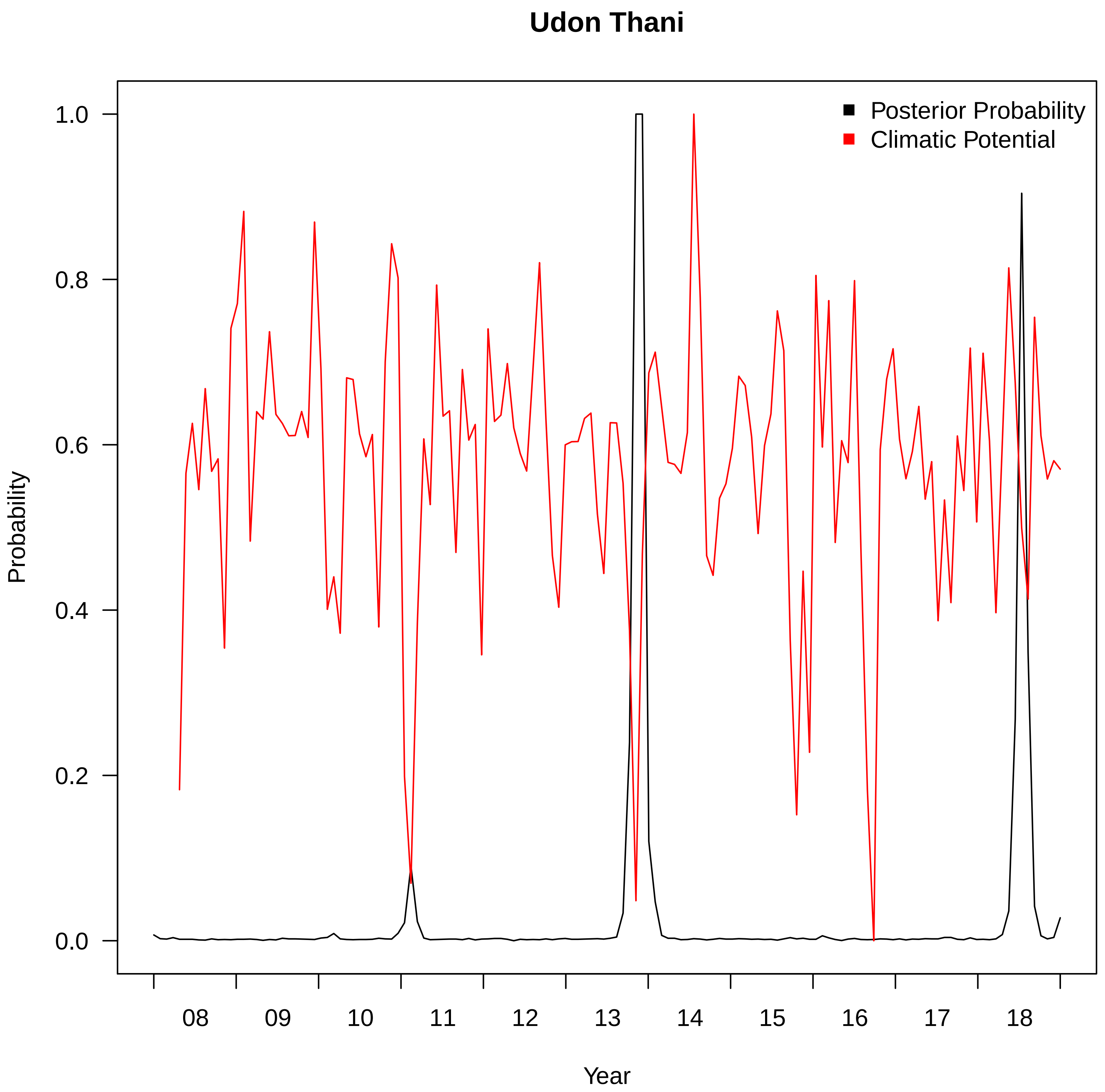

Udon Thani
Posterior Probability
Climatic Potential
Probability
1.0
0.8
0.6
0.4
0.2
0.0
08
09
10
11
12
13
14
15
16
17
18
Year

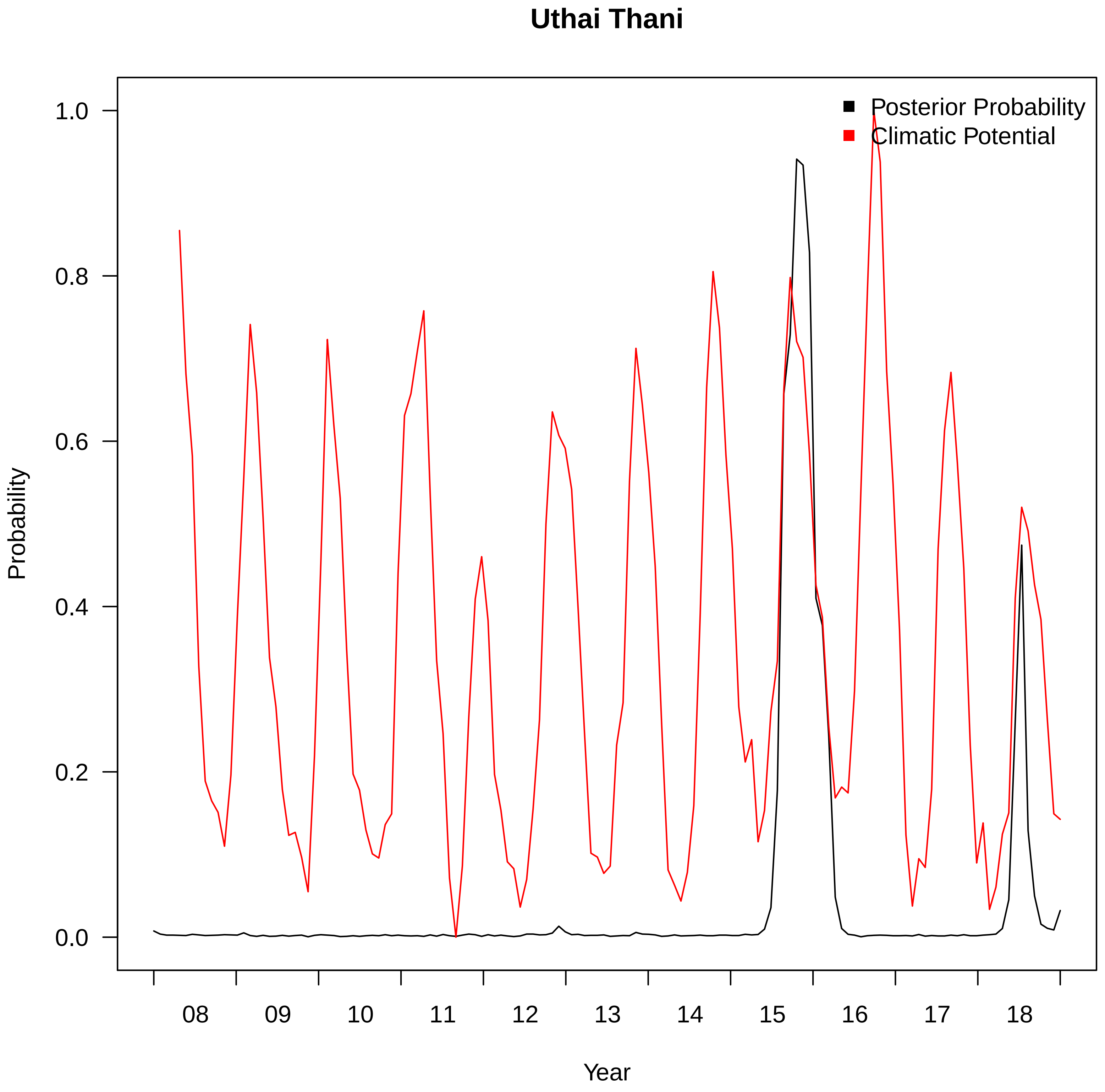

Uthai Thani
Posterior Probability
Climatic Potential
Probability
1.0
0.8
0.6
0.4
0.2
0.0
08
09
10
11
12
13
14
15
16
17
18
Year

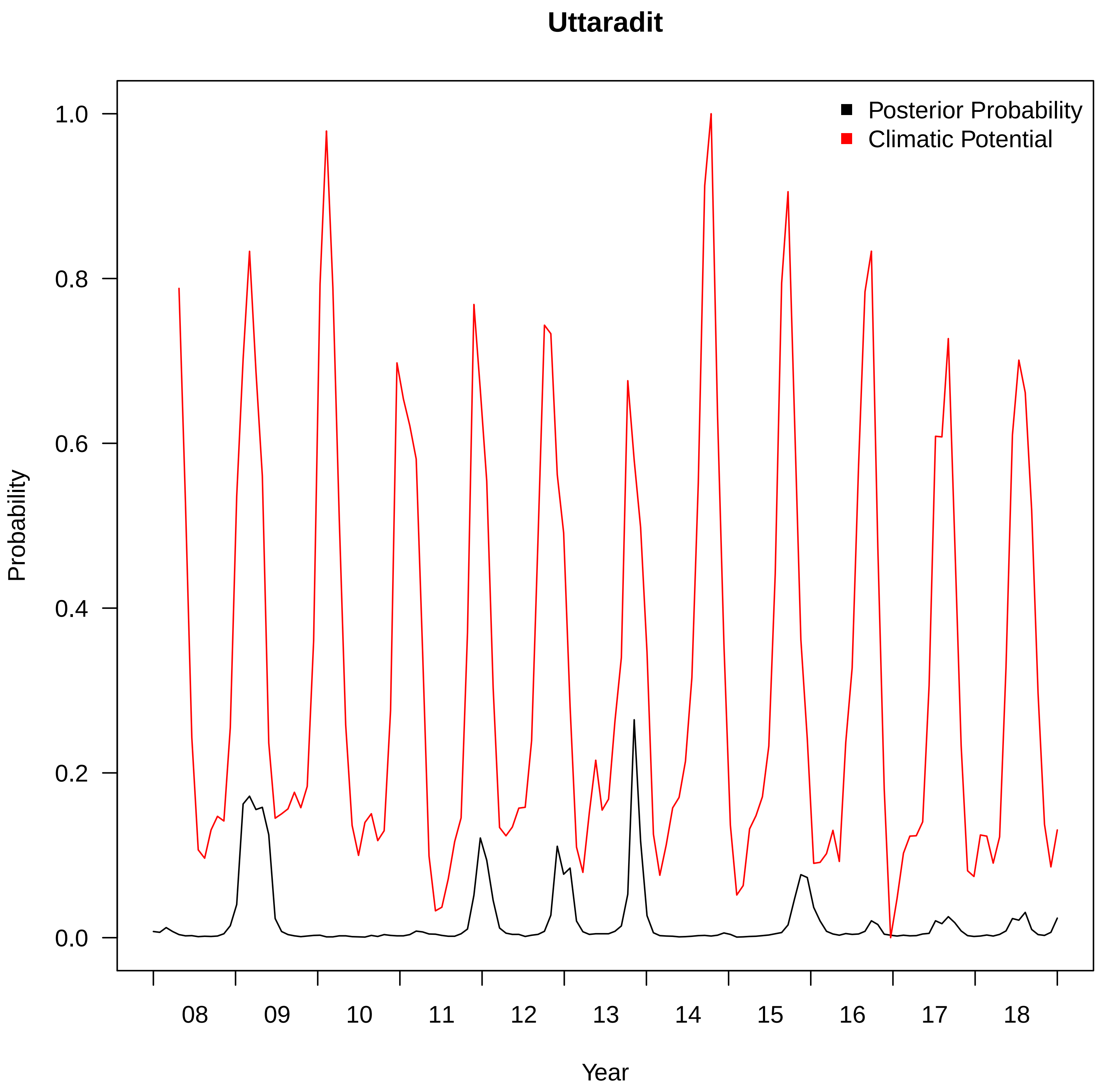
Uttaradit
Posterior Probability
Climatic Potential
Probability
1.0
0.8
0.6
0.4
0.2
0.0
08
09
10
11
12
13
14
15
16
17
18
Year

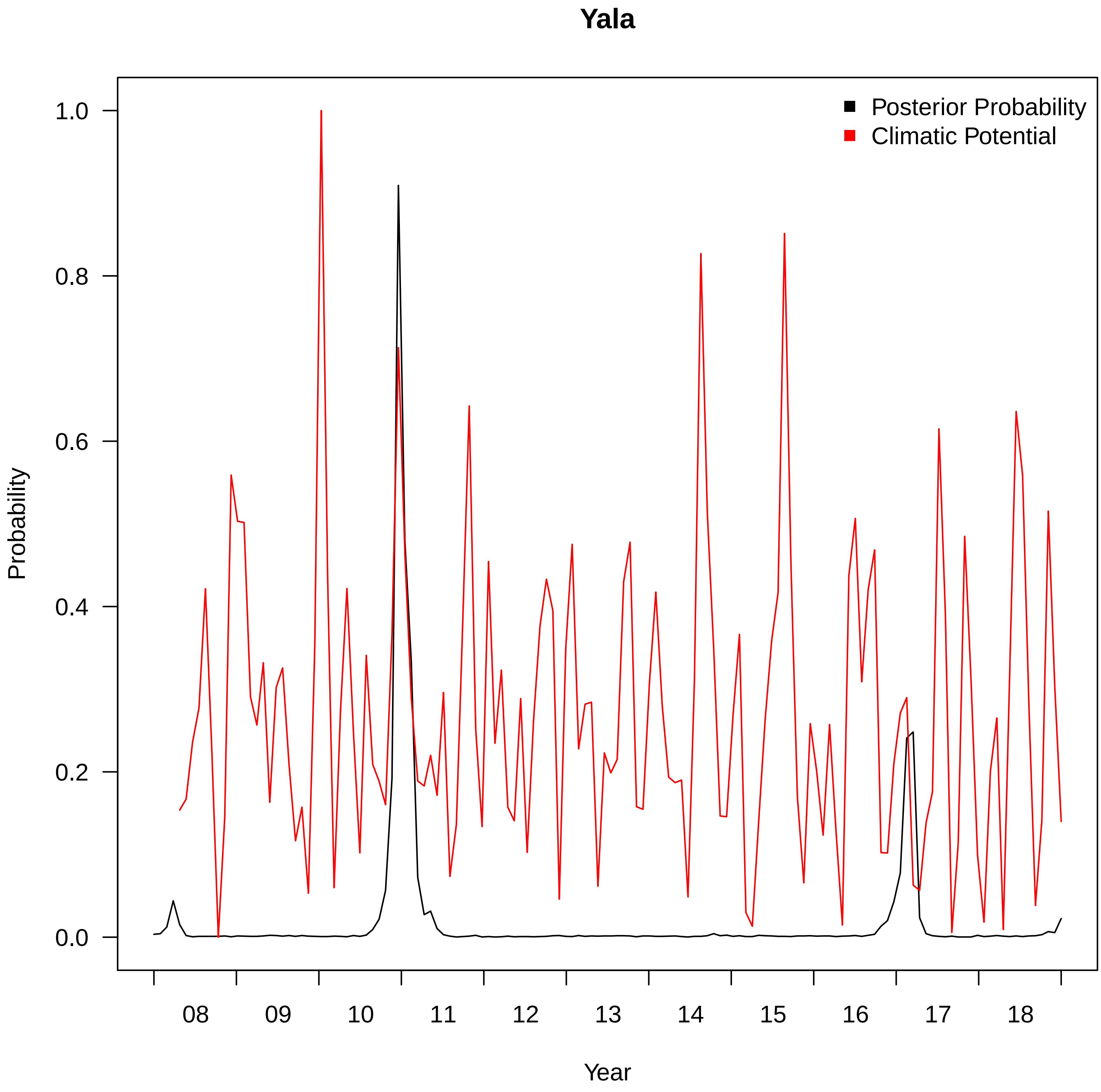

Yala
Posterior Probability
Climatic Potential
Probability
1.0
0.8
0.6
0.4
0.2
0.0
08
09
10
11
12
13
14
15
16
17
18
Year

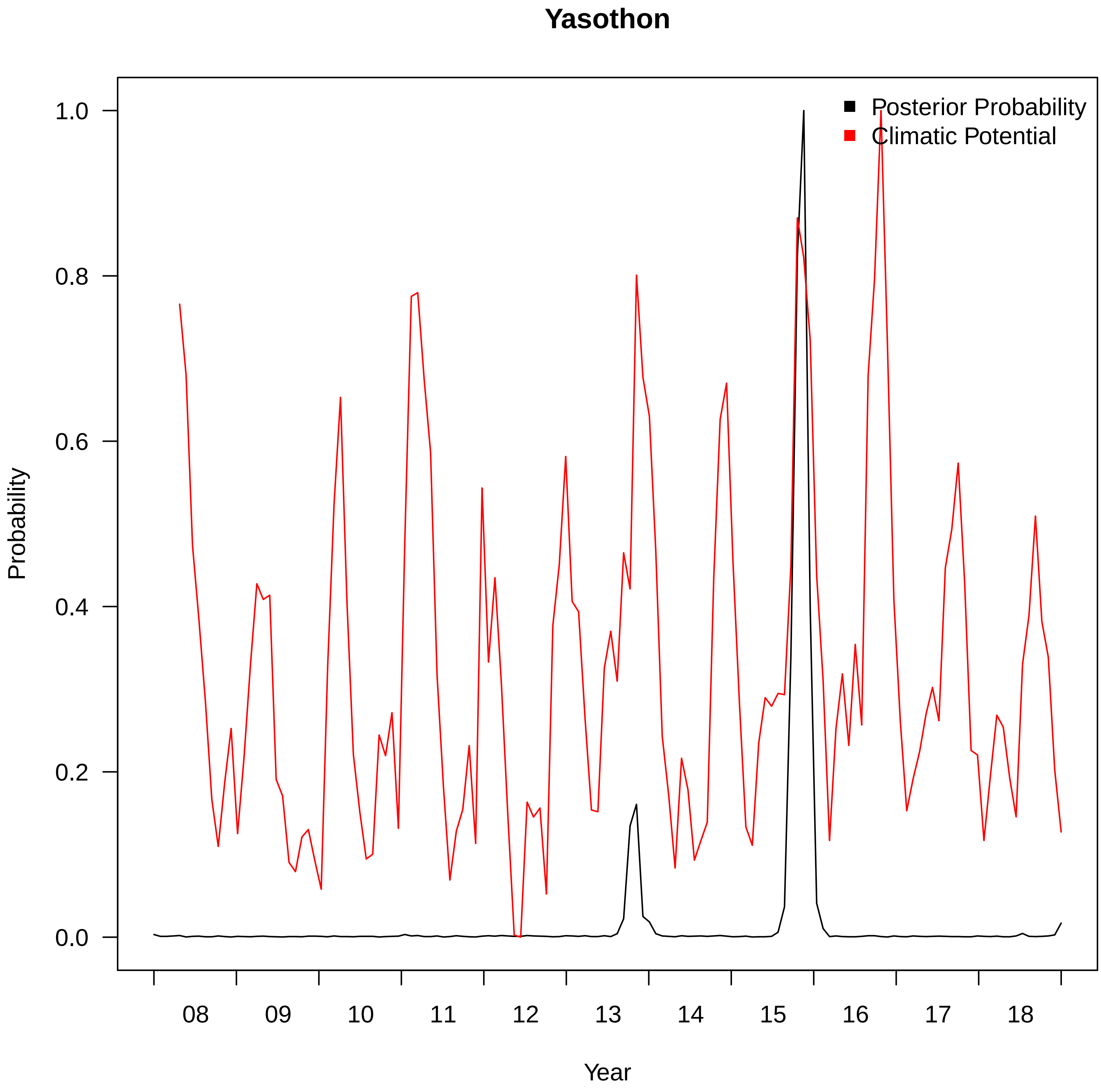

Yasothon
Posterior Probability
Climatic Potential
Probability
1.0
0.8
0.6
0.4
0.2
0.0
08
09
10
11
12
13
14
15
16
17
18
Year

References

Albert, James H and Siddhartha Chib (1993). "Bayes inference via Gibbs sampling of autoregressive time series subject to Markov mean and variance shifts". In: *Journal of Business & Economic Statistics* 11.1, pp. 1–15.

Caliński, Tadeusz and Jerzy Harabasz (1974). "A dendrite method for cluster analysis". In: *Communications in Statistics-theory and Methods* 3.1, pp. 1–27.

Cowling, Benjamin J et al. (2006). "Methods for monitoring influenza surveillance data". In: *International journal of epidemiology* 35.5, pp. 1314–1321.

Davies, David L and Donald W Bouldin (1979). "A cluster separation measure". In: *IEEE transactions on pattern analysis and machine intelligence* 2, pp. 224–227.

Dunn, Joseph C (1973). "A fuzzy relative of the ISODATA process and its use in detecting compact well-separated clusters". In:

Gurrutxaga, Ibai et al. (2010). "SEP/COP: An efficient method to find the best partition in hierarchical clustering based on a new cluster validity index". In: *Pattern Recognition* 43.10, pp. 3364–3373.

Kim, Chang-Jin and Charles R Nelson (1999). "State-space models with regime switching: classical and Gibbs-sampling approaches with applications". In: *MIT Press Books* 1.

Rousseeuw, Peter J (1987). "Silhouettes: a graphical aid to the interpretation and validation of cluster analysis". In: *Journal of computational and applied mathematics* 20, pp. 53–65.
